# Salmonella enterica Serovar Typhimurium SPI-1 and SPI-2 Shape the Global Transcriptional Landscape in a Human Intestinal Organoid Model System

**DOI:** 10.1128/mBio.00399-21

**Published:** 2021-05-18

**Authors:** Anna-Lisa E. Lawrence, Basel H. Abuaita, Ryan P. Berger, David R. Hill, Sha Huang, Veda K. Yadagiri, Brooke Bons, Courtney Fields, Christiane E. Wobus, Jason R. Spence, Vincent B. Young, Mary X. O’Riordan

**Affiliations:** a Department of Microbiology and Immunology, University of Michigan, Ann Arbor, Michigan, USA; b Department of Internal Medicine, University of Michigan, Ann Arbor, Michigan, USA; c Department of Cell and Developmental Biology, University of Michigan, Ann Arbor, Michigan, USA; Emory University School of Medicine

**Keywords:** enteric infection, host response, organoid

## Abstract

The intestinal epithelium is a primary interface for engagement of the host response by foodborne pathogens, like Salmonella enterica Typhimurium. While the interaction of *S*. Typhimurium with the mammalian host has been well studied in transformed epithelial cell lines or in the complex intestinal environment *in vivo*, few tractable models recapitulate key features of the intestine. Human intestinal organoids (HIOs) contain a polarized epithelium with functionally differentiated cell subtypes, including enterocytes and goblet cells and a supporting mesenchymal cell layer. HIOs contain luminal space that supports bacterial replication, are more amenable to experimental manipulation than animals and are more reflective of physiological host responses. Here, we use the HIO model to define host transcriptional responses to *S*. Typhimurium infection, also determining host pathways dependent on *Salmonella* pathogenicity island-1 (SPI-1)- and -2 (SPI-2)-encoded type 3 secretion systems (T3SS). Consistent with prior findings, we find that *S*. Typhimurium strongly stimulates proinflammatory gene expression. Infection-induced cytokine gene expression was rapid, transient, and largely independent of SPI-1 T3SS-mediated invasion, likely due to continued luminal stimulation. Notably, *S*. Typhimurium infection led to significant downregulation of host genes associated with cell cycle and DNA repair, leading to a reduction in cellular proliferation, dependent on SPI-1 and SPI-2 T3SS. The transcriptional profile of cell cycle-associated target genes implicates multiple miRNAs as mediators of *S*. Typhimurium-dependent cell cycle suppression. These findings from *Salmonella*-infected HIOs delineate common and distinct contributions of SPI-1 and SPI-2 T3SSs in inducing early host responses during enteric infection and reinforce host cell proliferation as a process targeted by *Salmonella*.

## INTRODUCTION

Enteric bacterial infections constitute a major human disease burden worldwide, with *Salmonella* species accounting for the most hospitalizations in outbreaks with a confirmed cause. In total, *Salmonella* causes an estimated 1.35 million infections in the United States alone (1). Enteric infections occur in a complex and highly dynamic environment that traverses the distinct landscapes of the gastrointestinal tract. Relevant to understanding infection are the host processes that shape physicochemical properties of the intestine, including regulation of pH and nutrient absorption, the epithelial layer, which establishes a barrier using epithelial tight junctions, mucus and antimicrobial peptides, the microbiome, and the pathogen itself. While animal models are valuable *in vivo* approaches to understanding enteric infections, these models suffer from two major limitations. First, the complexity of the mammalian intestine makes finely controlled experimental manipulation and observation challenging. Second, the physiology of the intestine in different organisms can differ sharply, i.e., mice rarely exhibit diarrhea upon infection by pathogens that would cause diarrhea in humans.

Salmonella enterica serovar Typhimurium infection is a prime example of this disease difference between humans and mice. While *S*. Typhimurium infection is most commonly associated with self-limiting gastroenteritis in otherwise healthy humans, it causes systemic acute disease in C57BL/6 mice (naturally Nramp deficient) or chronic disease in Nramp-sufficient mouse strains (2, 3). To interrogate molecular mechanisms of host-pathogen interactions during intracellular *S*. Typhimurium infection, many previous studies have relied on transformed human epithelial cell lines, such as HeLa cervical adenocarcinoma cells or Caco-2 colorectal adenocarcinoma cells, or primary mouse cells like embryonic fibroblasts or macrophages. These cell culture models have revealed much about *S*. Typhimurium infection but do not recapitulate several key features likely to be important during *S*. Typhimurium enteric infection. These include the continued presence of *S*. Typhimurium in the lumen, known to be an environment that supports robust replication, and interaction with nontransformed intestinal epithelial cells (IEC), which have specific properties, like mucus secretion or controlled cell cycle regulation. Thus, elucidating the cellular and molecular basis of *S*. Typhimurium-epithelial interactions in nontransformed human epithelial cells will advance our understanding of aspects of infection that may be relevant to human disease.

In the last decade, human intestinal organoid (HIO) systems have been developed to enable study of more complex IEC characteristics. These organoids are differentiated from nontransformed human pluripotent stem cell lines such as embryonic or induced pluripotent stem cells (ESC/iPSC) and form three-dimensional (3D) cyst-like structures, delineated by polarized epithelium with a mesenchymal layer surrounding a luminal space (4). HIOs contain multiple epithelial cell subsets, including enterocytes and goblet cells (4). A previous study characterized the global transcriptional profile of wild-type (WT) *S*. Typhimurium-infected HIOs using human induced pluripotent stem cells (hiPSC) and demonstrated that this IEC model could support *S*. Typhimurium infection (5). Their results established that the IEC transcriptional response to WT *S*. Typhimurium infection from the apical or basolateral route was dominated by proinflammatory innate immune signaling pathways. Additional studies have shown that HIOs can support survival and/or replication of both pathogenic and commensal bacteria and that commensal organisms, like Escherichia coli (ECOR2), stimulate cell proliferation, epithelial maturation, and barrier function (6, 7).

Here, we use HIOs derived from the H9 human embryonic stem cell line to define the host transcriptional response to infection by the commonly used laboratory strain *S*. Typhimurium SL1344 compared to isogenic mutants lacking functional SPI-1 or SPI-2 type 3 secretion systems (T3SS), major virulence determinants of *S*. Typhimurium that inject effector proteins into the host for cellular invasion and remodeling of host processes (8, 9). We find that *S*. Typhimurium-infected HIOs recapitulate some known aspects of intracellular infection and report new infection-induced host response profiles that are revealed by the unique features of the HIO model, notably sustained stimulation of the proinflammatory luminal bacteria and cross talk between the host epithelium and mesenchyme.

## RESULTS

### Luminal *Salmonella enterica* serovar Typhimurium replicates within HIOs and invades HIO epithelial cells.

**FIG 1:**
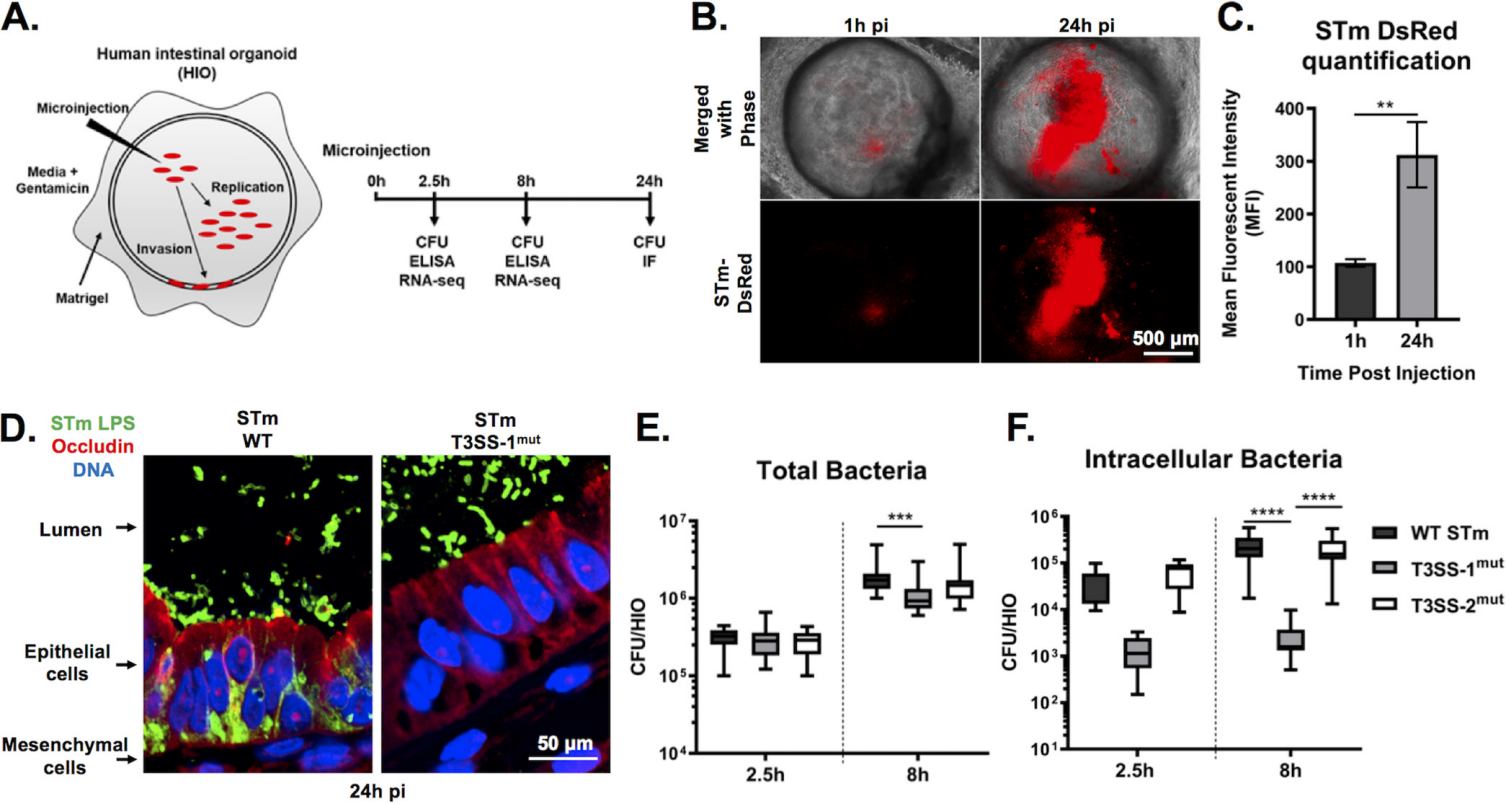
WT *S*. Typhimurium (STm) replicates within the lumen of HIOs and invades IECs dependent on T3SS-1. (A) Diagram of experimental protocol. (B) Fluorescence microscopy of HIOs injected with *S*. Typhimurium-DsRed, a strain that harbors the pGEN plasmid encoding red fluorescence protein (DsRed) (10). (C) Quantification of panel B. *n* = 3 biological replicates. Error bars represent SD. *P* = 0.0047 by unpaired *t* test. (D) Immunofluorescence of HIO sections infected with *S*. Typhimurium WT (left) and *S*. Typhimurium T3SS-1^mut^ (right). LPS, lipopolysaccharide. (E) Total bacteria in HIOs at 2.5 and 24 h postinjection. *n* = 16 biological replicates. Whiskers represent minimum and maximum values. Significance was calculated by two-way analysis of variance (ANOVA). (F) Intracellular bacteria in HIOs at 24 h postinjection. *n* > 31 biological replicates. Whiskers represent minimum and maximum values. Significance was calculated by Mann-Whitney test.

To better recapitulate the *in vivo* human intestinal epithelial response to *Salmonella* infection, we used the 3-dimensional HIO model that allows longer-term bacterium-host interactions compared to traditional cell lines by maintaining the bacteria in the luminal space throughout the course of infection. *S*. Typhimurium was inoculated into the HIO lumen by microinjecting each HIO with ∼10^3^ CFU (low inoculum used for experiments with 24-h duration) or phosphate-buffered saline (PBS) as a control (Fig. 1A). HIOs were allowed to recover for 2 h prior to a 15-min treatment with medium containing 100 μg/ml gentamicin to kill bacteria that were introduced into the culture medium during microinjection. Subsequently, HIOs were cultured in medium containing 10 μg/ml gentamicin for the remainder of the experiment to prevent replication of *S*. Typhimurium outside the HIOs. To confirm that *S*. Typhimurium replication could take place within HIOs, HIOs were injected with *S*. Typhimurium harboring the pGEN plasmid encoding the fluorescent protein DsRed (10), and bacterial burden was monitored by live fluorescence microscopy (Fig. 1B and C). Fluorescence intensity substantially increased by 24 h postinfection (pi), indicating that *S*. Typhimurium replicated within the HIOs. Replication appeared to occur predominantly in the lumen. Histological analysis of HIO sections suggested that luminal *S*. Typhimurium invaded intestinal epithelial cells and migrated to the basolateral side (Fig. 1D). Invasion did not occur uniformly across the HIO, as not all epithelial cells became infected. Additionally, infection was accompanied by increased mucus production on the luminal surface of the epithelial barrier and did not appear to cause major structural damage to the HIO that could be observed by hematoxylin and eosin (H&E) staining (see Fig. S1 in the supplemental material). To further quantify bacterial burden and to maximize invasion for subsequent experiments, we microinjected HIOs with ∼10^5^ CFU (higher inoculum used for experiments with 8-h duration) and enumerated total bacterial CFU per HIO at indicated times postinfection. Comparing results at 2.5 h to those at 8 h pi, we observed an approximately 1-log increase in the number of CFU (Fig. 1E), confirming that bacteria were replicating within the HIO. To test the contribution of the major virulence determinants, *S*. Typhimurium type III secretion system 1 (T3SS-1) and 2 (T3SS-2), to bacterial replication within the HIO, we microinjected HIOs with Δ*orgA* (T3SS-1^mut^) and Δ*ssaV* (T3SS-2^mut^) isogenic mutants. We found that both mutants could replicate within HIOs, since we also observed an approximately 1-log increase in total bacteria from 2.5 h to 8 h pi, although T3SS-1^mut^ did not reach levels as high as those of either the WT or T3SS-2^mut^ (Fig. 1E). Consistent with previous reports, invasion was largely dependent on the *S*. Typhimurium type III secretion system (T3SS) on pathogenicity island 1 (SPI-1) (11), as an in-frame deletion in a structural gene of the T3SS apparatus (Δ*orgA*) drastically reduced intracellular CFU numbers (Fig. 1F). Together, these results demonstrate that the HIO model supports robust luminal and intracellular replication of *S*. Typhimurium and that invasion of HIO epithelial cells is dependent on T3SS-1.

10.1128/mBio.00399-21.1 FIG S1 Histology of HIO sections fixed 24 h pi with PBS (left) or *S*. Typhimurium (right). Sections were stained with hematoxylin and eosin (top) and periodic acid-Schiff (bottom) to detect mucus. Download FIG S1, TIF file, 0.3 MB. Copyright © 2021 Lawrence et al. 2021 Lawrence et al. https://creativecommons.org/licenses/by/4.0/ This content is distributed under the terms of the Creative Commons Attribution 4.0 International license.

### Kinetic analysis of HIO transcriptional profiles defines the acute response to *Salmonella* infection.

**FIG 2:**
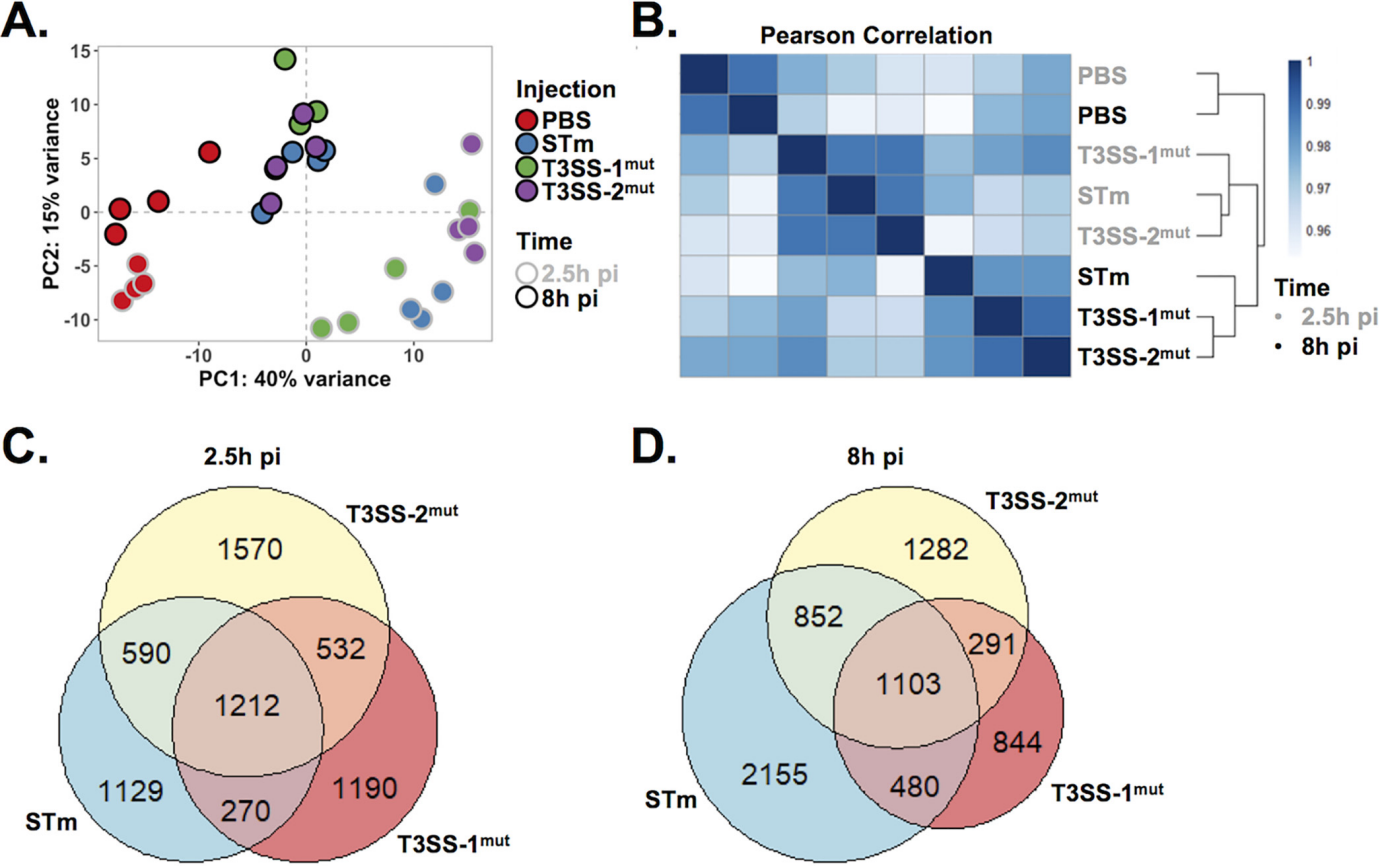
HIOs mount an acute transcriptional response to *Salmonella* infection. (A) Principal-component analysis of HIOs injected with *S*. Typhimurium T3SS mutants. Each circle represents a biological replicate. (B) Sample distance plot of each HIO condition at 2.5 h (gray) and 8 h (black) postinjection. Sample distance was calculated from normalized gene counts across 4 biological replicates. (C and D) Euler diagram comparison of gene changes in each HIO condition relative to PBS-injected HIOs at 2.5 h (C) and 8 h (D) postinjection. Genes were filtered by a *P* value of <0.05.

To gain insight into global HIO transcriptional responses stimulated by *S*. Typhimurium infection and to define the relative contributions of the major virulence determinants, T3SS-1 and -2, we performed global RNA sequencing (RNA-seq) with HIOs microinjected with PBS, WT *S*. Typhimurium, T3SS-1^mut^, and T3SS-2^mut^. RNA was isolated at 2.5 h and 8 h pi to characterize early and intermediate transcriptional responses to infection, including any initial responses that occurred upon immediate recognition of the bacteria. Principal-component analysis (PCA) showed that all infected HIOs displayed markedly different transcriptional profiles than those injected with PBS (Fig. 2A). Notably, sample clustering occurred primarily by time postinfection, because HIOs infected for 2.5 h and 8 h segregated from each other along the first principal component (*x* axis). This difference accounted for 40% of the total variance and suggested that time postinfection is a greater determinant of transcriptional variance than the contributions of the SPI-1 or SPI-2 T3SS. Similar patterns were observed by Pearson’s correlation clustering, which showed clustering of 2.5-h and 8-h samples (Fig. 2B). In addition, the Pearson’s correlation heat map showed that HIOs infected with the invasion-defective T3SS-1^mut^ segregated away from samples infected with WT *S*. Typhimurium and T3SS-2^mut^ at 2.5 h pi, while at 8 h pi, HIOs infected with WT *S*. Typhimurium separated from both mutants. These data suggest that T3SS-2^mut^ is attenuated later in infection than the wild type, a time point at which bacteria have invaded the epithelium, and T3SS-2 is thought to be active in maintaining intracellular infection (9, 12, 13). Using differential gene expression (DEG) analysis, we found that HIOs injected with any of the 3 strains of *S*. Typhimurium resulted in similar numbers of significant gene changes (*P*  < 0.05) at 2.5 h pi compared to PBS controls, suggesting that the early HIO response is driven primarily by luminal bacteria (Fig. 2C and Tables S1 and S2). In contrast, at 8 h both T3SS mutant strains induced fewer significant gene changes than the WT, suggesting that T3SS-1 and -2 effectors are required for *S*. Typhimurium-induced responses later during infection (Fig. 2D).

10.1128/mBio.00399-21.4 TABLE S1 DEGs of *S*. Typhimurium-, T3SS-1^mut^-, and T3SS-2^mut^-infected HIOs at 2.5 h relative to PBS-injected HIOs. Significant DEGs (*P* < 0.05) under at least one infection condition are listed. Download Table S1, PDF file, 2.4 MB. Copyright © 2021 Lawrence et al. 2021 Lawrence et al. https://creativecommons.org/licenses/by/4.0/ This content is distributed under the terms of the Creative Commons Attribution 4.0 International license.

10.1128/mBio.00399-21.5 TABLE S2 DEGs of *S*. Typhimurium-, T3SS-1^mut^-, and T3SS-2^mut^-infected HIOs at 8 h relative to PBS-injected HIOs. Significant DEGs (*P* < 0.05) under at least one infection condition are listed. Download Table S2, PDF file, 2.4 MB. Copyright © 2021 Lawrence et al. 2021 Lawrence et al. https://creativecommons.org/licenses/by/4.0/ This content is distributed under the terms of the Creative Commons Attribution 4.0 International license.

### Immune pathways and cell cycle pathways are inversely regulated during *Salmonella* infection.

**FIG 3:**
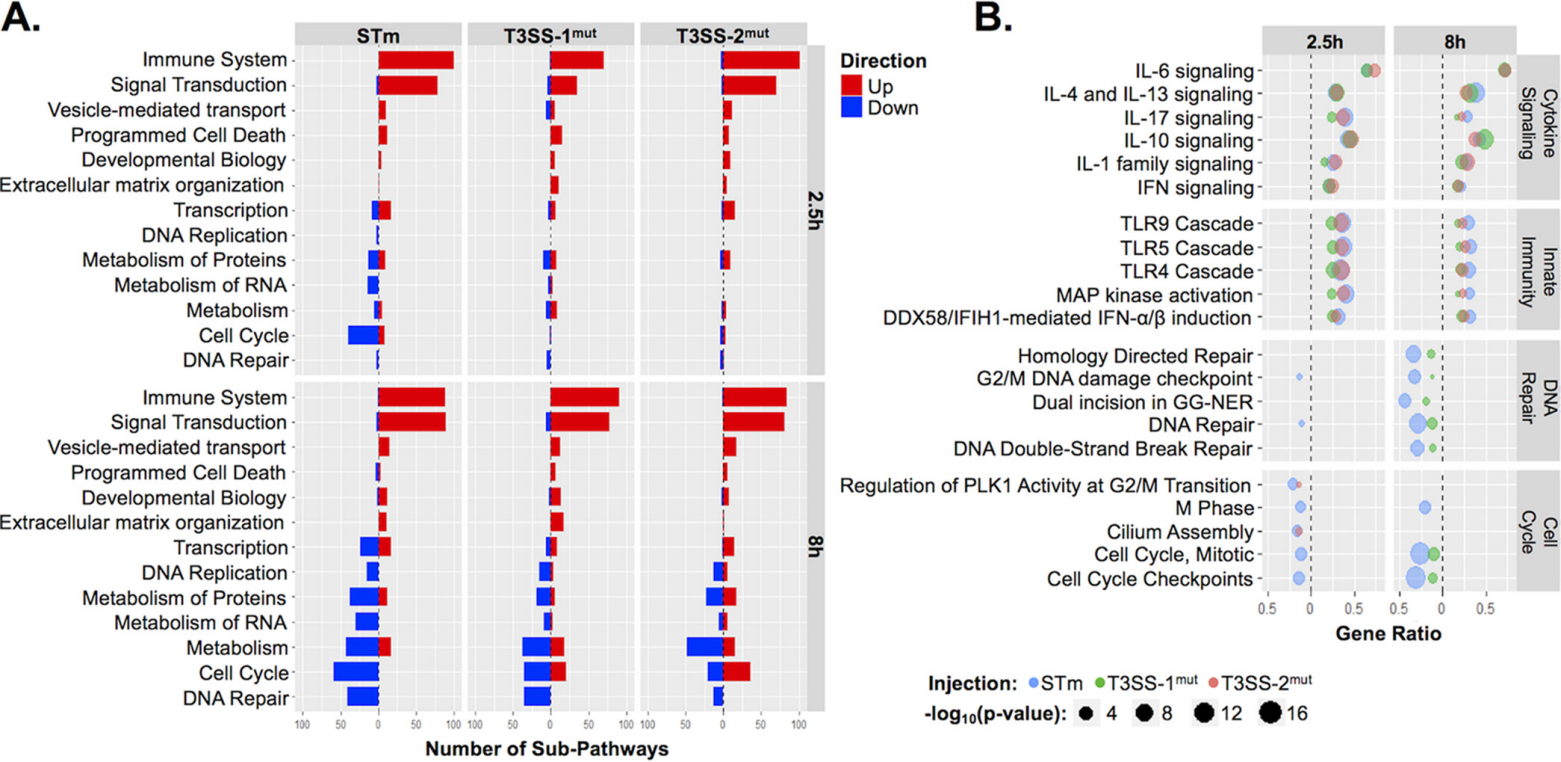
Reactome pathway enrichment reveals upregulation of immune system pathways and downregulation of cell cycle and DNA repair pathways. (A) Number of subpathways clustering into major Reactome pathways. Significantly upregulated (red) or downregulated (blue) genes were analyzed using ReactomePA (41), and pathways were clustered into the major pathways from the Reactome database. Major pathways were filtered so that at least 12 subpathways were significantly enriched under at least one condition. (B) Dot plot showing top pathways enriched from Reactome database. Pathway coverage is shown as a gene ratio. –Log_10_(*P* value) is presented as the dot size, with WT *S*. Typhimurium in blue, T3SS-1^mut^ in green, and T3SS-2^mut^ in red. Upregulated pathways are shown on the right of the dotted line and downregulated pathways on the left.

To determine which pathways drive the HIO response to *S*. Typhimurium infection, we performed pathway enrichment analysis using the Reactome database (Tables S3A and B and S4A and B). Clustering of subpathways into major cellular processes in the Reactome database indicated that the majority of upregulated pathways under all three infection conditions clustered into immune response and signal transduction processes (Fig. 3A). We examined individual pathway enrichment by gene ratio (fraction of genes in a pathway that were significantly changed) and the −log_10_(*P* value) to identify pathways modulated by *S*. Typhimurium infection and dependence on T3SS-1 or T3SS-2. To our surprise, we observed similar gene ratios between infection with WT *S*. Typhimurium and the two T3SS mutants in several cytokine signaling pathways, including interleukin-4 (IL-4), IL-17, and IL-10 signaling pathways (Fig. 3B, top). These results are in contrast to previous reports that T3SS-1-dependent invasion strongly contributes to the inflammatory response, including upregulation of chemokines such as IL-8 (14–16). However, distinct from most tissue culture infection models, the HIO model system features sustained epithelial interaction with both luminal and intracellular *Salmonella*, pointing to a strong contribution of luminal bacteria in triggering early inflammation. Importantly, not all inflammatory pathways were equally enriched under all 3 infection conditions; innate immune signaling pathways, including Toll-like receptor (TLR) signaling cascades, were less enriched in T3SS-1^mut^-infected HIOs at 2.5 h pi and in both T3SS-1^mut^- and T3SS-2^mut^-infected HIOs compared to the WT at 8 h pi, suggesting that modulation of these pathways is enhanced by intracellular infection (Fig. 3B, middle).

10.1128/mBio.00399-21.6 TABLE S3 (A) Enriched Reactome pathways from upregulated genes at 2.5 h pi. Significant Reactome pathways using upregulated DEGs from *S*. Typhimurium-, T3SS-1^mut^-, or T3SS-2^mut^-infected HIOs at 2.5 h. (B) Enriched Reactome pathways from downregulated genes at 2.5 h pi. Significant Reactome pathways using downregulated DEGs from *S*. Typhimurium-, T3SS-1^mut^-, or T3SS-2^mut^-infected HIOs at 2.5 h. Download Table S3, PDF file, 0.3 MB. Copyright © 2021 Lawrence et al. 2021 Lawrence et al. https://creativecommons.org/licenses/by/4.0/ This content is distributed under the terms of the Creative Commons Attribution 4.0 International license.

10.1128/mBio.00399-21.7 TABLE S4 (A) Enriched Reactome pathways from upregulated genes at 8 h pi. Shown are significant Reactome pathways using upregulated DEGs from *S*. Typhimurium-, T3SS-1^mut^-, or T3SS-2^mut^-infected HIOs at 8 h. (B) Enriched Reactome pathways from downregulated genes at 8 h pi. Significant Reactome pathways using downregulated DEGs from *S*. Typhimurium-, T3SS-1^mut^-, or T3SS-2^mut^-infected HIOs at 8 h. Download Table S4, PDF file, 0.4 MB. Copyright © 2021 Lawrence et al. 2021 Lawrence et al. https://creativecommons.org/licenses/by/4.0/ This content is distributed under the terms of the Creative Commons Attribution 4.0 International license.

Few downregulated pathways were observed at 2.5 h pi, with more evident by 8 h pi, largely related to cell cycle and DNA repair. Genes involved in cell cycle processes, including checkpoints and mitotic (M) phase pathways, were more highly suppressed in WT-infected HIOs than in T3SS-1^mut^- and T3SS-2^mut^-infected HIOs (Fig. 3B, bottom). Taken together, our findings show that upregulated pathways primarily consisted of immune-related pathways that were only partially dependent on the two T3SS, while downregulated pathways dominated by cell cycle processes required both T3SS-1 and T3SS-2.

### Luminal *S*. Typhimurium contributes to rapid induction of inflammatory gene expression.

**FIG 4:**
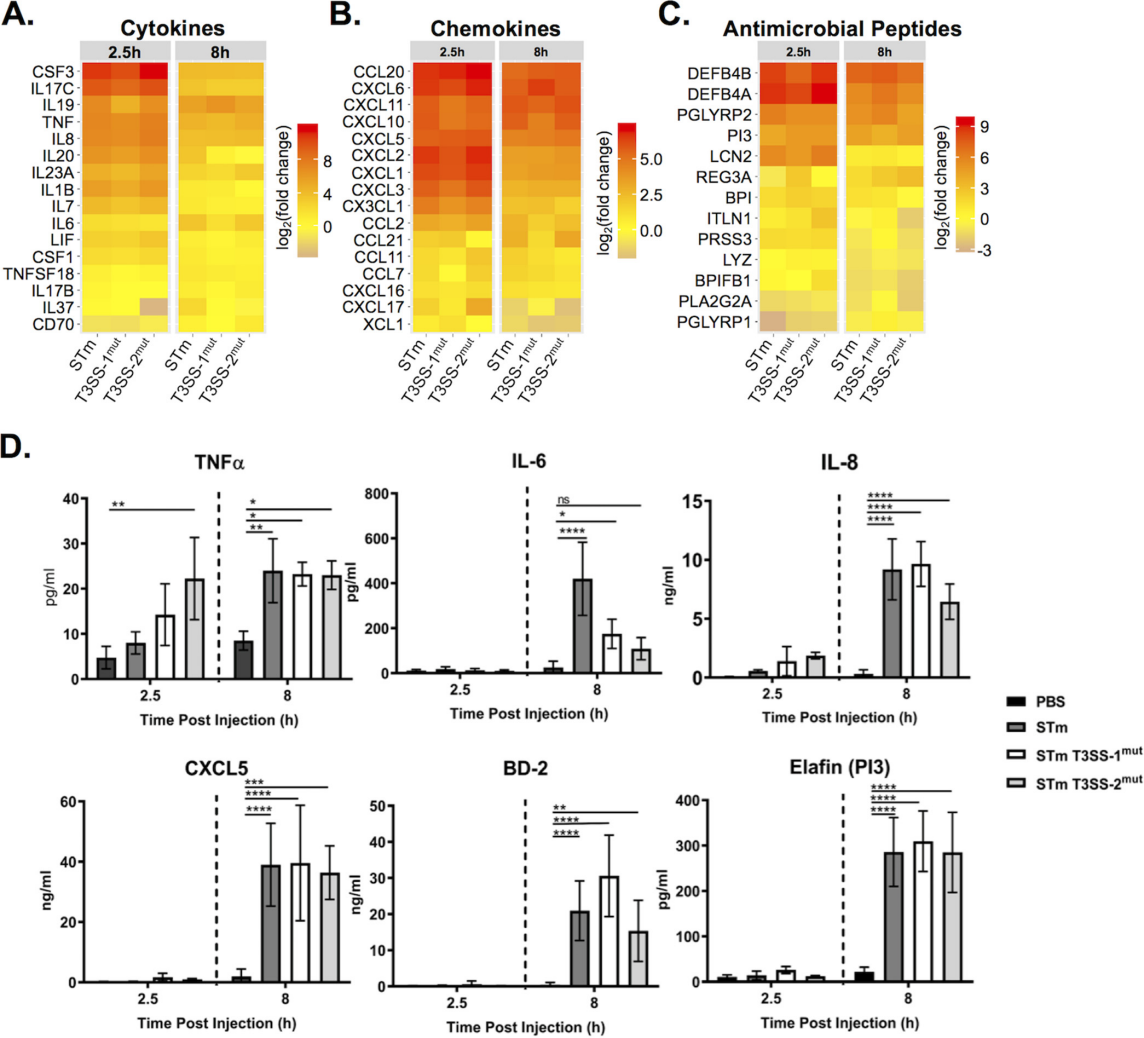
Cytokine, chemokine, and antimicrobial peptide induction is not dependent on T3SS-1 or T3SS-2. (A to C) Gene expression presented as log_2_(fold change) relative to PBS injected HIOs at 2.5 h and 8 h postinjection. (A) Cytokine expression; (B) chemokine expression; (C) antimicrobial peptide expression. (D) Cytokine, chemokine, and antimicrobial peptide levels measured from HIO supernatant at 2.5 and 8 h postinjection via ELISA. *n* = 4 biological replicates. Error bars represent SD. Significance was calculated by two-way ANOVA.

We also analyzed the expression of individual genes, selecting proinflammatory gene sets from the Reactome database (cytokines, chemokines, and antimicrobial peptides [AMPs]), to examine fold change relative to PBS-injected control HIOs (Fig. 4A to C and Fig. S2). Induction of genes in all three categories occurred rapidly, characterized by markedly increased levels of cytokine, chemokine, and AMP transcripts at 2.5 h pi that were reduced by 8 h pi. Global patterns revealed that infection with T3SS-1^mut^ induced weaker stimulation of these proinflammatory mediators than the other infection conditions, although many transcripts were still upregulated compared to PBS-injected HIOs. The strongest responders to infection were cytokines *CSF3*, also called granulocyte colony-stimulating factor (G-CSF), and *IL17C*, and the antimicrobial peptide beta defensin-2 (*DEFB4*). Strong upregulation of *IL17C* and *DEFB4*, genes involved in epithelial intrinsic defenses (17–19), suggests that upon sensing infection, epithelial cells mount a direct antimicrobial response in addition to producing chemokines to recruit other immune cells. Notably, chemokine genes were not induced as strongly at these time points as cytokine and AMP genes (Fig. 4B). Some other responses occurred independently of either T3SS-1 and T3SS-2, including tumor necrosis factor (*TNF*), *IL-8*, and *CXCL5* genes, as fold change was comparable between the three conditions while *IL-6* expression was dependent on T3SS-1.

10.1128/mBio.00399-21.2 FIG S2 Complete cytokine, chemokine, and antimicrobial peptide gene lists from Reactome. Gene expression presented as log_2_(fold change) relative to PBS-injected HIOs at 2.5 h and 8 h postinjection. (A) Cytokine expression. (B) Chemokine expression. (C) Antimicrobial peptide expression. Download FIG S2, TIF file, 0.3 MB. Copyright © 2021 Lawrence et al. 2021 Lawrence et al. https://creativecommons.org/licenses/by/4.0/ This content is distributed under the terms of the Creative Commons Attribution 4.0 International license.

To test whether gene expression level differences were reflected at the protein level, we collected supernatants from infected HIOs at 2.5 h and 8 h pi and measured cytokines by enzyme-linked immunosorbent assay (ELISA). In concordance with the transcript data, release of TNF, IL-8, and CXCL5 was consistent across all three infection conditions (Fig. 4D and Fig. S3). While the degree of transcript upregulation for AMPs varied between time points across the three infections, release of these mediators (Beta Defensin-2 and ELAFIN) into the medium did not significantly differ between WT and mutant infections. In contrast, IL-6 was present at significantly lower levels in supernatants from HIOs infected with either mutant, even though transcripts were increased in *S*. Typhimurium- and T3SS-2^mut^-infected HIOs by 8 h pi. Reduced levels of IL-6 in the supernatant in T3SS-2^mut^-infected HIOs, even though *IL-6* transcript was induced to WT levels, suggest additional posttranscriptional regulation affecting IL-6 production in infected HIOs. Collectively, these results show that the HIOs mount a rapid proinflammatory, antimicrobial transcriptional response to *S*. Typhimurium infection and that invasion-defective T3SS-1^mut^ bacteria, previously reported to have a large defect in inducing an inflammatory response, can signal through the luminal compartment to induce robust inflammation following prolonged interactions with the epithelium.

10.1128/mBio.00399-21.3 FIG S3 Chemokine levels measured from HIO supernatant at 2.5 h and 8 h postinjection via ELISA. *n* = 4 biological replicates. Error bars represent SD. Significance was calculated by two-way ANOVA. Download FIG S3, TIF file, 0.1 MB. Copyright © 2021 Lawrence et al. 2021 Lawrence et al. https://creativecommons.org/licenses/by/4.0/ This content is distributed under the terms of the Creative Commons Attribution 4.0 International license.

### Downregulation of cell cycle pathways during *S*. Typhimurium infection is dependent on T3SS-1 and T3SS-2.

**FIG 5:**
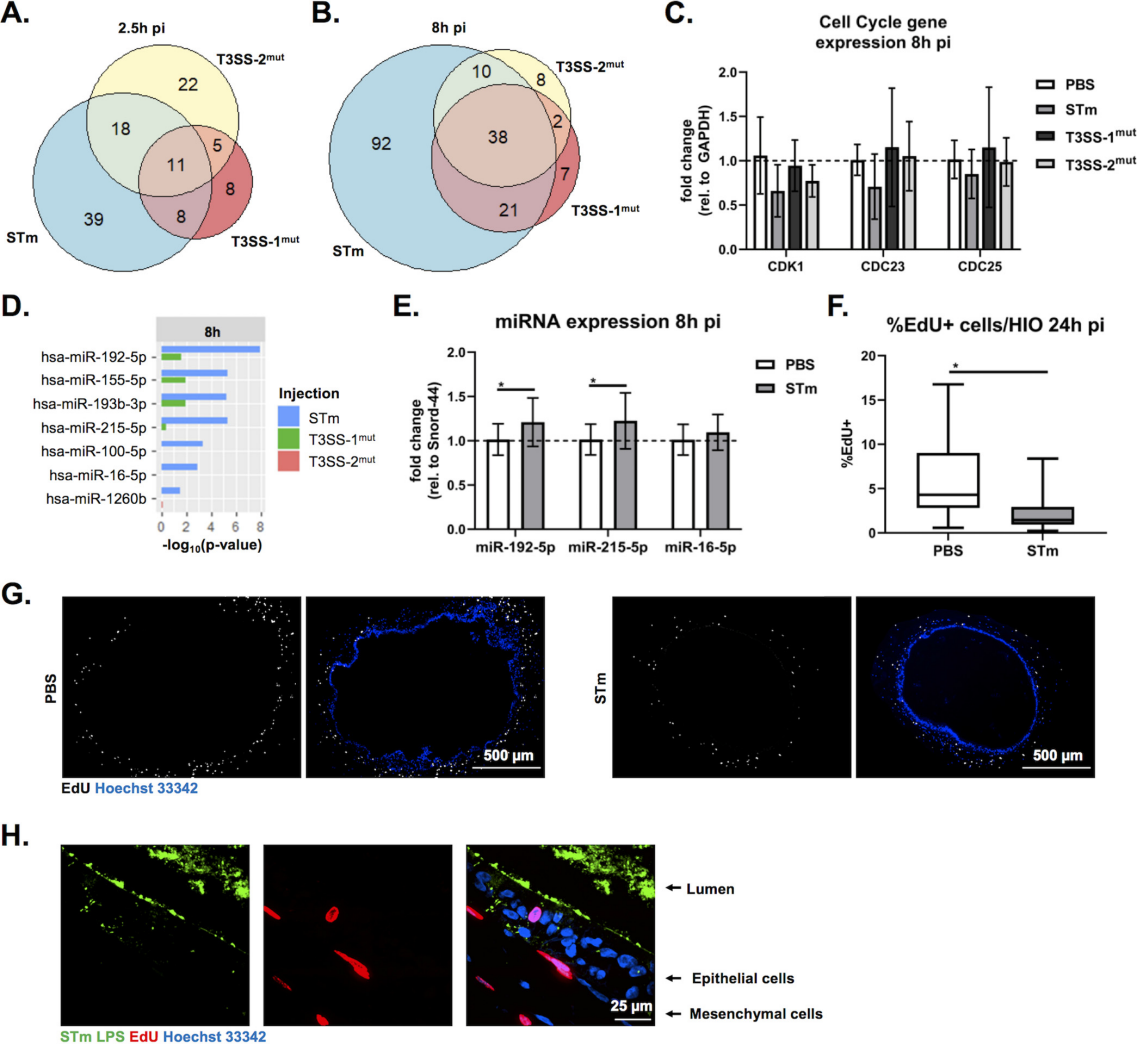
*S*. Typhimurium infection suppresses cell cycle dependent on T3SS-1 and T3SS-2. (A and B) Euler diagram comparison of cell cycle genes downregulated compared to PBS injected HIOs at 2.5 h (A) and 8 h (B) postinjection. Genes were filtered by a *P* value of <0.05. (C) RT-qPCR validation of RNA-seq data testing expression of select cell cycle genes at 8 h pi. (D) miRNA enrichment profiles were calculated using Gprofiler package in R (25) based on significantly downregulated genes compared to PBS injected HIOs. –Log_10_(*P* value) is plotted for each miRNA that is significantly enriched under at least one infection condition. (E) RT-qPCR testing miRNA expression in infected HIOs at 8 h pi. Significance was determined by one-tailed *t* test (*, *P* < 0.05). (F) Quantitation of EdU-positive cells per HIO at 24 h pi. Outliers were removed using the ROUT method, where Q = 0.1%, and significance was determined by unpaired *t* test (*, *P* < 0.05). (G) Fluorescent images of HIO sections microinjected with PBS (left) and *S*. Typhimurium WT (right) exposed to EdU for 24 h. (H) High-magnification image of *S*. Typhimurium-injected HIO assessed for EdU-positive cells at 24 h pi.

We next evaluated genes that were downregulated during *S*. Typhimurium infection. Our pathway enrichment analysis identified cell cycle as the category containing the most significantly downregulated pathways. To assess whether downregulation of cell cycle-related pathways was dependent on T3SS-1 and/or -2, we directly compared genes in the cell cycle pathway that were significantly changed under the three infection conditions. In agreement with our pathway-level analysis (Fig. 3A), we found relatively few genes in the cell cycle pathway significantly downregulated compared to PBS-injected HIOs at 2.5 h pi (Fig. 5A). However, by 8 h pi the number of significantly downregulated genes substantially increased from 76 genes to 161 genes in the WT-infected HIOs (Fig. 5B). Downregulation of gene expression was partially dependent on both T3SS-1 and -2, as only 68 genes and 58 genes, respectively, were significantly downregulated at 8 h pi. To validate these findings, reverse transcription-quantitative PCR (RT-qPCR) was performed on RNA isolated at 8 h pi. While there was some variation across biological replicates, we consistently observed the downregulation of cell cycle genes CDK1, CDC23, and CDC25a in 3 out of our 4 biological replicates infected with WT *S*. Typhimurium but not in HIOs infected with either T3SS-1 or T3SS-2 mutants (Fig. 5C). These observations are consistent with a role for the T3SS in establishing an intracellular niche for *S*. Typhimurium replication.

Decreases in transcript levels can occur through several mechanisms, including halting synthesis of new transcripts or through degradation of existing transcripts by microRNA (miRNA). Evidence for miRNA expression manipulation by pathogens, including *Salmonella*, continues to emerge (20–24), so we used gprofiler2 (25) as the basis for an informatics approach to identify potential regulatory miRNAs associated with our downregulated gene sets. Analysis of infected HIOs with gprofiler2 yielded several miRNAs predicted to be associated with the WT *S*. Typhimurium-downregulated gene sets, while no miRNA was strongly associated with downregulated gene sets from T3SS-1^mut^- or T3SS-2^mut^-infected HIOs (Fig. 5D). Several of these miRNA species, including miR-192-5p and miR-215-5p, which were significantly associated with the WT *S*. Typhimurium-infected HIO gene set, regulate cell proliferation (26, 27), and miR-16-5p overexpression during *Salmonella* infection (20) alters cell cycle progression. To validate whether specific miRNA species were altered during infection in the HIOs, we tested expression of miR-192-5p, miR-215-5p, and miR-16-5p by RT-qPCR. Consistent with our bioinformatics prediction, we observed significant upregulation of the top predicted miRNA species miR-192-5p and miR-215-5p in *S*. Typhimurium-infected HIOs compared to PBS-injected control HIOs but did not observe a significant difference in miR-16-5p levels (Fig. 5E). To evaluate if decreased cell cycle-associated transcripts functionally impacted cell cycle progression, we treated HIOs with EdU for 24 h to monitor cellular proliferation in HIOs that were injected with PBS or WT *S*. Typhimurium. *S*. Typhimurium significantly reduced the number of EdU-positive cells in the HIOs (Fig. 5F and G). Strikingly, we did not observe EdU-positive staining in cells actively infected by *S*. Typhimurium; instead, EdU was primarily associated with the surrounding mesenchymal cells (Fig. 5H). Taken together, these data suggest that downregulation of cell cycle genes during WT *S*. Typhimurium infection, likely driven in part by miRNA-mediated silencing, leads to a decrease in cellular proliferation in supporting mesenchymal cells.

## DISCUSSION

Initial human intestinal responses to Salmonella enterica serovar Typhimurium are still incompletely understood despite the prominent contribution of this species to human disease burden. Here, we used the human intestinal organoid model to analyze transcriptional profiles defining early host responses to *S*. Typhimurium infection, including the contribution of two major virulence determinants, T3SS-1 and -2. We found that HIOs responded rapidly and robustly to all 3 infections by upregulating proinflammatory pathways early and transiently, whereas downregulation of host pathways, including cell cycle and DNA repair, occurred later and only in WT *S*. Typhimurium-infected HIOs.

*Salmonella* infection strongly induces inflammatory responses and exploits the inflammatory environment created during infection to outcompete the resident microbiota and replicate within the lumen of the intestine (28). Accordingly, our transcriptomics analysis found that the dominant response occurring in the HIOs was inflammatory. While this was largely expected for WT *S*. Typhimurium infection, based on studies in other model systems, we had predicted that infection with T3SS-1^mut^ would result in reduced activation of these pathways. Prior studies showed that T3SS-1 strongly contributes to the inflammatory response, with significantly reduced levels of inflammation and colitis in mouse models and little to no upregulation of proinflammatory cytokines in tissue culture models of *S*. Typhimurium infection (14–16). However, we observed largely similar patterns of induction of several proinflammatory mediators in HIOs infected with T3SS-1^mut^. This included IL-8, which in HeLa cells was dependent on T3SS-1 effectors for upregulation (29). This finding highlights the advantage of using model systems that more closely reflect physiologic infection conditions. Mice do not naturally present with the same disease as humans following *Salmonella* infection, suggesting that there are differences in infection progression, and although immortalized cell lines can more easily be manipulated than mouse models, the inoculum is removed after the initial infection; therefore, longer-term interactions between the luminal surface of the epithelium and the bacteria cannot be easily studied. The enclosed lumen of the HIOs naturally limits the extent of extracellular bacterial replication and allows the study of longer-term interactions, revealing a strong contribution of luminal bacteria in inducing upregulation of proinflammatory mediators, as shown by host response to T3SS-1^mut^, which exhibits a >2-log defect in invasion. Prior evidence suggests that gut luminal bacteria can act as a reservoir to continually seed sites of systemic infection (30), and the HIO model may serve to explore the dynamic contribution of luminal bacteria to continued invasion, immune modulation, and remodeling of the luminal environment.

Among our upregulated gene sets, key targets reflected known modulators of *S*. Typhimurium infection. The strongest responder in all 3 infection conditions, *CSF3* (encoding G-CSF), was previously implicated in regulating a supershedder phenotype of *Salmonella* to enhance the spread of the bacteria to other hosts, and injection of G-CSF in moderate-shedder animals recapitulated the supershedder phenotype (29). Additionally, *IL17C* and *DEFB4* contribute to epithelial intrinsic defenses against bacterial pathogens by regulating intestinal barrier integrity and bacterial killing, respectively (17–19). Overall, the transcriptional responses across the 3 infection conditions were similar, with only a slight decrease in upregulation in the T3SS-1^mut^-infected HIOs. Notably, transcriptional upregulation of *IL-6* appeared to be dependent on T3SS-1. Interestingly, while *IL-6* transcript upregulation was dependent on T3SS-1, neither T3SS-1^mut^ nor T3SS-2^mut^ infection stimulated significant IL-6 protein production compared to that of PBS-injected HIOs. These observations suggest a novel function for T3SS-2 in posttranscriptional regulation of IL-6 production. Together, these findings highlight several avenues for future study, including IL-6 posttranscriptional regulation by T3SS-2 and how *CSF3* regulation and function in the early stages of *S*. Typhimurium infection may contribute to a supershedder phenotype, using an HIO system reconstituted with immune cells, like neutrophils.

Downregulation of host gene expression was dependent on T3SS-1 and -2 and notably consisted primarily of cell cycle-related genes. Cell cycle regulation in the intestine affects the rate of cell turnover to shed infected or damaged cells and, therefore, is commonly targeted by bacteria (30, 31). A previous study from our consortium group showed that HIO colonization with a commensal strain of E. coli enhanced cell proliferation and, therefore, could be protective against invasive infections (6). In contrast, Pinchuk and colleagues recently reported that *S*. Typhimurium can block cell cycle progression in mouse intestinal cells and proposed that this enhances intestinal colonization of *S*. Typhimurium (32). This study showed that T3SS-2 effectors regulated cell proliferation through targeting proteins important for cleavage furrow formation rather than exerting regulation at the transcriptional level. Here, we found that both T3SS-1 and T3SS-2 contribute to downregulating cell cycle-related transcripts leading to a reduction in cellular proliferation, suggesting that *S*. Typhimurium can also regulate the cell cycle at the transcriptional level. Consistent with this observation, Maudet et al. reported that transcriptional regulation of cyclin D1 during *S*. Typhimurium infection promoted cell cycle arrest in G_2_/M phase and identified the miR-15 family as key regulators (20). Expression of miRNAs is increasingly appreciated as a mechanism regulating gene expression during bacterial infections, and our results highlight miR-192-5p and miR-215-5p as likely contributors to *S*. Typhimurium-associated suppression of cell proliferation (31). Although our bioinformatics prediction showed significant association of all three miRNAs with our downregulated gene set, we only measured upregulation of miR-192-5p and miR-215-5p. There is known overlap in cell cycle genes that are regulated by each of these three miRNAs (31); thus, increased expression of any one or more of these miRNAs leading to downregulation of common target genes would likely be sufficient to predict associations for all three regulatory miRNAs. Notably, the Maudet et al. study identified miR-16, which we determined to be unchanged in the HIOs in this study, as one of the miRNA species that was downregulated during *S*. Typhimurium infection to regulate cell cycle progression (20). For further validation, we tested miRNA expression in HeLa cells (the primary cell line used in the Maudet et al. study), and although miR-16-5p expression was detected in our HeLa cell experiment, neither miR-192-5p nor miR-215-5p was detected (data not shown). These observations suggest that baseline miRNA expression is different in each model system, underscoring the need to more closely recapitulate physiologic conditions when studying host responses to *Salmonella.*

Finally, our observation that *S*. Typhimurium infection reduced proliferation in supporting mesenchymal cells builds on a previous study demonstrating that commensal E. coli strain ECOR2 stimulates epithelial cell proliferation (6), highlighting the complex interactions that can be revealed in the HIO model. Mesenchymal cells serve many roles in the intestine, including sensing and responding to inflammation, both during initial pathogen recognition and during resolution, as well as modulating cellular proliferation through Wnt signaling (32). The mesenchymal cells also serve as a second-line defense against invading pathogens in the intestine (33). Mesenchymal cells engage in cross talk with nearby cells to limit tissue damage and can also reduce inflammation by secreting antagonizing receptors for IL-1 or TNF-α or through production of anti-inflammatory proteins, such as stanniocalcin-1 (34). Our findings hint at the cross talk between epithelial and mesenchymal cells, implicating epithelial signaling to the mesenchyme to reduce cellular proliferation during *S*. Typhimurium infection. This engagement may further enhance inflammation during *S*. Typhimurium infection due to potential depletion of immunoregulatory cells (32). Future work, including utilizing single-cell RNA-seq technology, will further elucidate the specific interactions between epithelial and mesenchymal cells during *Salmonella* infection.

Collectively, the complex and dynamic transcriptional response in the *S*. Typhimurium-infected HIOs demonstrates the utility of using this nontransformed epithelial cell model to examine what aspects are specific and physiologically relevant to human intestinal disease. HIOs supported both luminal and intracellular bacterial replication while still maintaining overall structural integrity, better mimicking the interaction of both these bacterial populations with the epithelium *in vivo*. This model system allows for the observation of infected cells as well as bystander cells that can be studied using single-cell RNA-seq, and because of the enclosed environment, the entire HIO can be visualized in sections or by live-cell imaging. Additionally, with the enclosed lumen, it is possible to study sustained responses induced by the bacteria from the extracellular environment, an important aspect of *S*. Typhimurium infection biology that has been difficult to study in traditional cell culture models. As further evidence to strengthen this model for future studies, our upregulated gene set for the WT infection was highly concordant with data from an earlier study that looked at the iPSC-derived HIO transcriptional responses to WT *S*. Typhimurium infection (5); 90% of the top 30 genes regulated by *S*. Typhimurium were also significantly changed in our data set. This concordance opens areas for future work, including studying posttranscriptional regulation of cytokine production by T3SS-2 and epithelial-mesenchymal interactions modulating cell cycle processes during *S*. Typhimurium infection. Additionally, the HIO model is well suited to characterize host responses to other Salmonella enterica serovars to elucidate how individual serovars interact uniquely with the host, as well as layering in components such as a simplified microbiome or immune cells to study more complex interactions between *Salmonella* and the human intestine.

## MATERIALS AND METHODS

### HIO differentiation and culture.

HIOs were generated by the *In Vivo* Animal and Human Studies Core at the University of Michigan Center for Gastrointestinal Research, as previously described (35). Human ES cell line WA09 (H9) was obtained from Wicell International Stem Cell Bank and cultured on Matrigel (BD Biosciences)-coated 6-well plates in mTeSR1 medium (Stem Cell Technologies) at 37°C in 5% CO_2_. Cells were seeded onto Matrigel-coated 24-well plates in fresh mTeSR1 medium and grown until 85 to 90% confluence. Definitive endoderm differentiation was induced by washing the cells with PBS and culturing in endoderm differentiation medium (RPMI 1640, 2% fetal bovine serum [FBS], 2 mM l-glutamine, 100 ng/ml activin A, and 100 U/ml penicillin-streptomycin) for 3 days, and media were exchanged each day. Cells were then washed with endoderm differentiation medium without activin A and cultured in mid/hindgut differentiation medium (RPMI 1640, 2% FBS, 2 mM l-glutamine, 500 ng/ml FGF4, 500 ng/ml WNT3A, and 100 U/ml penicillin-streptomycin) for 4 days until spheroids were present. Spheroids were collected, mixed with ice-cold Matrigel (50 μl of Matrigel plus 25 μl of media plus 50 spheroids), placed in the center of each well of a 24-well plate, and incubated at 37°C for 10 min to allow Matrigel to solidify. Matrigel embedded spheroids were grown in ENR medium (Dulbecco’s modified Eagle medium [DMEM]-F12, 1× B27 supplement, 2 mM l-glutamine, 100 ng/ml epidermal growth factor [EGF], 100 ng/ml Noggin, 500 ng/ml Rspondin1, and 15 mM HEPES) for 14 days, and medium was replaced every 4 days. Spheroids growing into organoids (HIOs) were dissociated from Matrigel by pipetting using a cut wide-tip pipette (2 to 3 mm). HIOs were mixed with Matrigel (6 HIOs plus 25 μl of media plus 50 μl of Matrigel) and placed in the center of each well of 24-well plates and incubated at 37°C for 10 min. HIOs were further grown for 14 days in ENR medium with medium exchanged every 4 days. Before use in experiments, HIOs were carved out of Matrigel, washed with DMEM-F12, and replated with 5 HIO/well in 50 μl of Matrigel in ENR medium with medium exchanged every 2 to 3 days for 7 days prior to microinjection.

### Bacterial growth conditions and HIO microinjection.

*S*. Typhimurium strains used in this study are listed in Table S5 in the supplemental material. Strains were stored at −80°C in LB medium containing 20% glycerol and cultured on Luria-Bertani (LB; Fisher) agar plates. Selected colonies were grown overnight at 37°C under static conditions in LB liquid broth. Bacteria were pelleted, washed, and resuspended in PBS. Bacterial inoculum was estimated based on the optical density at 600 nm and verified by plating serial dilutions on agar plates to enumerate CFUs. HIOs were cultured in groups of 5/well using 4-well plates (ThermoFisher). Individual HIO lumens were microinjected using a glass caliber needle with 1 μl of PBS control or different *S*. Typhimurium mutants (10^5^ CFU/HIO for 8-h infections or 10^3^ CFU/HIO for 24-h infections). HIOs were washed with PBS and incubated for 2 h at 37°C in ENR medium to allow for resealing of the epithelial layer. HIOs were then treated with gentamicin (100 μg/ml) for 15 min to kill bacteria outside the HIOs and then incubated in fresh medium with gentamicin (10 μg/ml).

10.1128/mBio.00399-21.8 TABLE S5 List of bacterial strains used in this study, previously described in references 46 and 47. Download Table S5, PDF file, 0.04 MB. Copyright © 2021 Lawrence et al. 2021 Lawrence et al. https://creativecommons.org/licenses/by/4.0/ This content is distributed under the terms of the Creative Commons Attribution 4.0 International license.

### Quantitative measurement of HIO-associated bacteria and cytokine secretion.

Quantitation of viable bacteria was assessed per HIO. Individual HIOs were removed from Matrigel, washed with PBS, and homogenized in PBS. Total numbers of CFU/HIO were enumerated by serial dilution and plating on LB agar. To assess intracellular bacterial burden, HIOs were sliced in half, treated with gentamicin (100 μg/ml) for 10 min to kill luminal bacteria, washed with PBS, and homogenized, and bacterial CFU were enumerated on LB agar. Medium from each well (5 HIOs/well) was collected at indicated time points after microinjection, and cytokines, chemokines, and defensins were quantified by ELISA at the UM ELISA core.

### Immunohistochemistry and immunofluorescence staining.

HIOs were fixed with 10% neutral buffered formalin or Carnoy’s solution for 2 days and embedded in paraffin. HIOs were sectioned (5-μm thickness) by the UM Histology Core and stained with hematoxylin and eosin (H&E). Carnoy’s fixed HIO sections were stained with periodic acid-Schiff (PAS) staining reagents according to the manufacturer’s instructions (Newcomersupply). H&E- and PAS-stained slides were imaged on an Olympus BX60 upright microscope. For immunofluorescence staining, formalin-fixed HIO sections were deparaffinized and subjected to antigen retrieval in sodium citrate buffer (10 mM sodium citrate, 0.05% Tween 20, pH 6.0). Sections were permeabilized with PBS plus 0.2% Triton X-100 for 30 min and then incubated in blocking buffer (PBS, 5% bovine serum albumin, and 10% normal goat serum) for 1 h. Human Occludin was stained using rabbit anti-Occludin polyclonal antibody (ThermoFisher) in blocking buffer overnight at 4°C. Goat anti-mouse secondary antibody conjugated to Alexa-594 was used according to the manufacturer’s instructions (ThermoFisher) for 1 h at room temperature in blocking buffer. *Salmonella* organisms were stained using fluorescein isothiocyanate-conjugated anti-*Salmonella* Typhimurium antibody (1E6; Santa Cruz) in blocking buffer for 1 h at room temperature. 4′,6-Diamidino-2-phenylindole (DAPI) was used to stain DNA. Sections were mounted using coverslips (no. 1.5) and Prolong Diamond antifade mountant (ThermoFisher). Images were taken on a Nikon A1 confocal microscope and processed using ImageJ.

### Cell proliferation analysis.

After microinjection, 25 μM EdU was added to the HIO culture medium and incubated at 37°C for 24 h to allow incorporation into dividing cells. HIOs were then fixed with 10% neutral buffered formalin for 2 days and embedded in paraffin. HIOs were sectioned (5-μm thickness) by the UM Histology Core, and samples were stained using the Click-iT EdU cellular proliferation kit (ThermoFisher) according to the manufacturer’s protocol. HIOs were counterstained using the anti-*Salmonella* Typhimurium antibody and Hoechst to detect bacteria and DNA, respectively, before mounting in Prolong glass antifade mountant (ThermoFisher). Images were taken on an Olympus BX60 upright microscope and processed and analyzed using ImageJ and CellProfiler.

### RNA sequencing.

Total RNA was isolated from groups of 5 HIOs per replicate with a total of 4 replicates per condition using the mirVana miRNA isolation kit (ThermoFisher). Cytosolic and mitochondrial rRNA was removed from samples using the Ribo-Zero gold kit according to the manufacturer’s instructions (Illumina). The quality of RNA was confirmed (RNA integrity number, >8.5) using a Bioanalyzer and used to prepare cDNA libraries by the UM DNA Sequencing Core. Libraries were sequenced on Illumina HiSeq 2500 platforms (single-end, 50-bp read length).

### RT-qPCR analysis.

Total RNA was isolated with 5 HIOs per replicate with a total of 4 biological replicates per condition using the mirVana miRNA isolation kit (ThermoFisher). cDNA was synthesized using random hexamers (Invitrogen), and gene expression was tested using PowerUp SYBR green master mix (Invitrogen). The change in threshold cycle was calculated relative to glyceraldehyde-3-phosphate dehydrogenase (GAPDH) expression. The following primer sequences were used: for GAPDH, F, 5′-CTCTGCTCCTCCTGTTCGAC-3′; R, 5′-TTAAAAGCAGCCCTGGTGAC-3′; for CDK1, F, 5′-CACATGAGGTAGTAACACTCTG-3′; R, 5′-CAAATGTCAACTGGAGTTGAG-3′; for CDC23, F, 5′-CACTGCCTTTCGCTATCTG-3′; R, 5′-TTCCCGGGTATCATTAAATGC-3′; for CDC25, F, 5′-CTGGAGGTGAAGAACAACAG-3′; R, 5′-AGGAGAATCTAGACAGAAACCTG-3′. To quantify changes in miRNA expression, cDNA was synthesized using the miRCURY LNA RT kit (Qiagen) according to the manufacturer’s protocol and detected using the miRCURY LNA SYBR green PCR kit (Qiagen) with the following primers: YP00205702, YP00204099, YP00204598, and YP00203902. SNORD44 was used as a housekeeping control to calculate the change in threshold cycle for each miRNA.

### Statistical methods.

Data were analyzed using GraphPad Prism 7 and R software. Statistical tests for all analyses are outlined in the figure legends. The means from at least 3 independent experiments are presented, with error bars showing standard deviations (SD). *P* values of less than 0.05 were considered significant: *, *P* < 0.05; **, *P* < 0.01; ***, *P* < 0.001; ****, *P* < 0.0001. All statistically significant comparisons within experimental groups are marked.

### Data and software availability.

Data were deposited into ArrayExpress (E-MTAB-10451). Source code for analyses can be found at https://github.com/rberger997/HIO_dualseq2 and https://github.com/aelawren/HIO_RNAseq.

### RNA-seq analysis protocol. (i) Sequence alignment.

Sequencing generated FASTQ files of transcript reads were pseudoaligned to the human genome (GRCh38.p12) using kallisto software (36). Transcripts were converted to estimated gene counts using the tximport (37) package with gene annotation from Ensembl (38).

### (ii) Differential gene expression.

Differential expression analysis was performed using the DESeq2 package (39), with *P* values calculated by the Wald test and adjusted *P* values calculated using the Benjamini & Hochberg method (40).

### (iii) Pathway enrichment analysis.

Pathway analysis was performed using the Reactome pathway database and pathway enrichment analysis in R using the ReactomePA software package (41). miRNA analysis was performed using Gprofiler2 package (25).

### Statistical analysis.

Analysis was done using RStudio version 1.1.453. Plots were generated using ggplot2 (42) with data manipulation done using dplyr (43). Euler diagrams of gene changes were generated using the Eulerr package (44). Cluster heatmaps were generated using the pheatmap package (45).
