# Supplementary figures and images for: Salmonella enterica Serovar Typhimurium SPI-1 and SPI-2 Shape the Global Transcriptional Landscape in a Human Intestinal Organoid Model System
Source: mBio. 2021 May 18;12(3):e00399-21. doi: 10.1128/mBio.00399-21 (PMC8262845; doi:10.1128/mBio.00399-21)

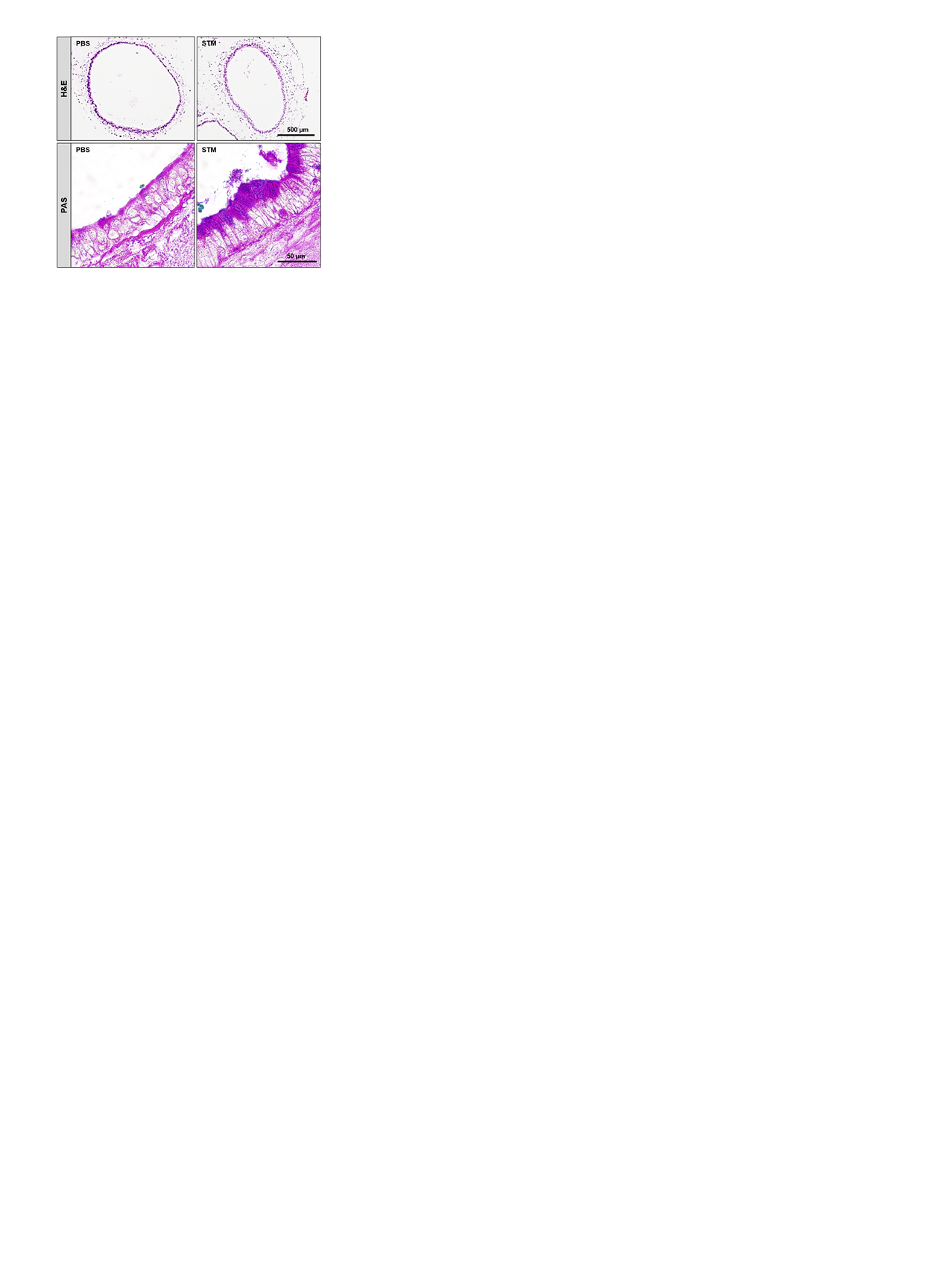

Supplement: FIG S1 [file mbio.00399-21-sf001.tif]

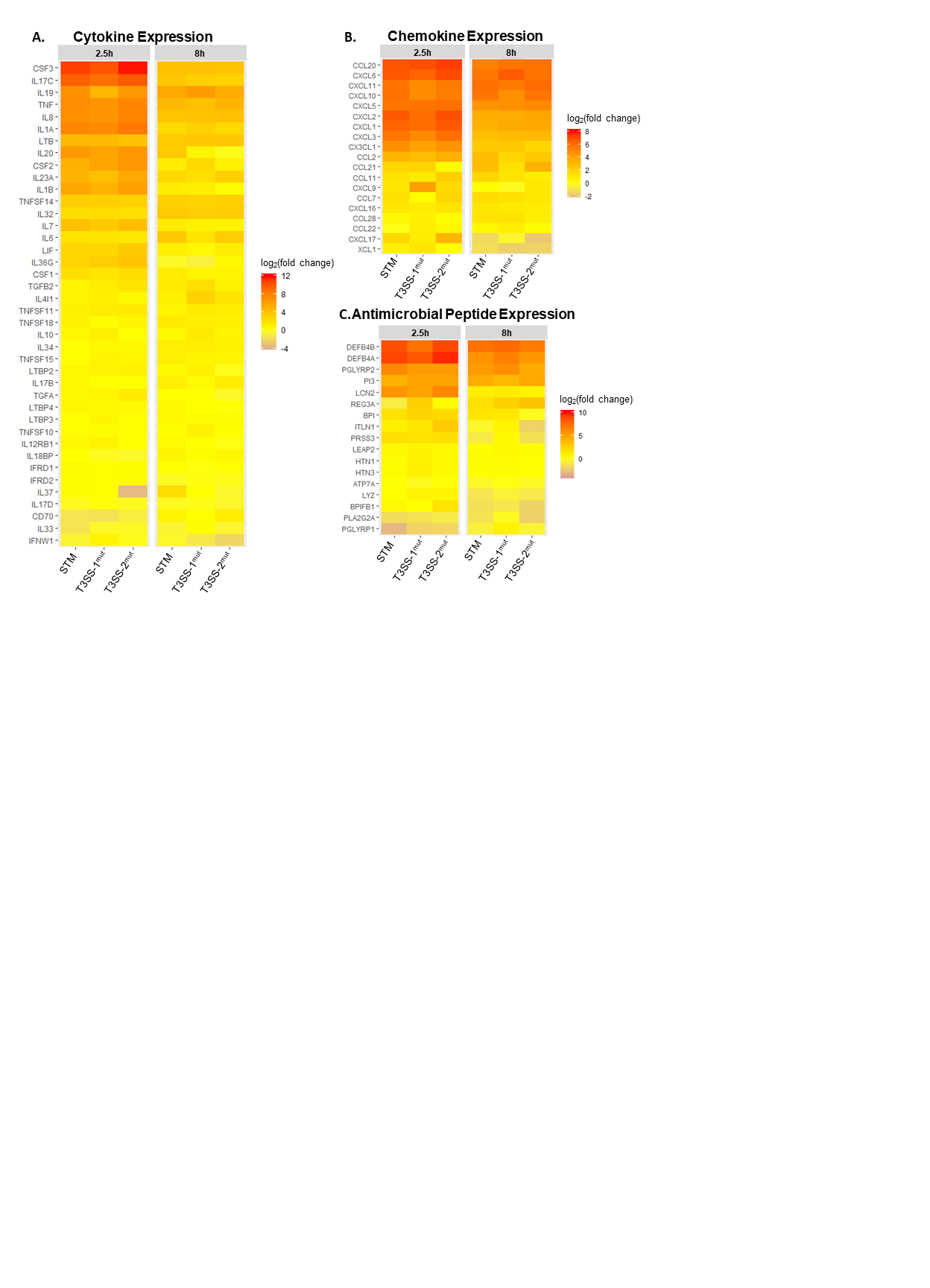

Supplement: FIG S2 [file mbio.00399-21-sf002.tif]

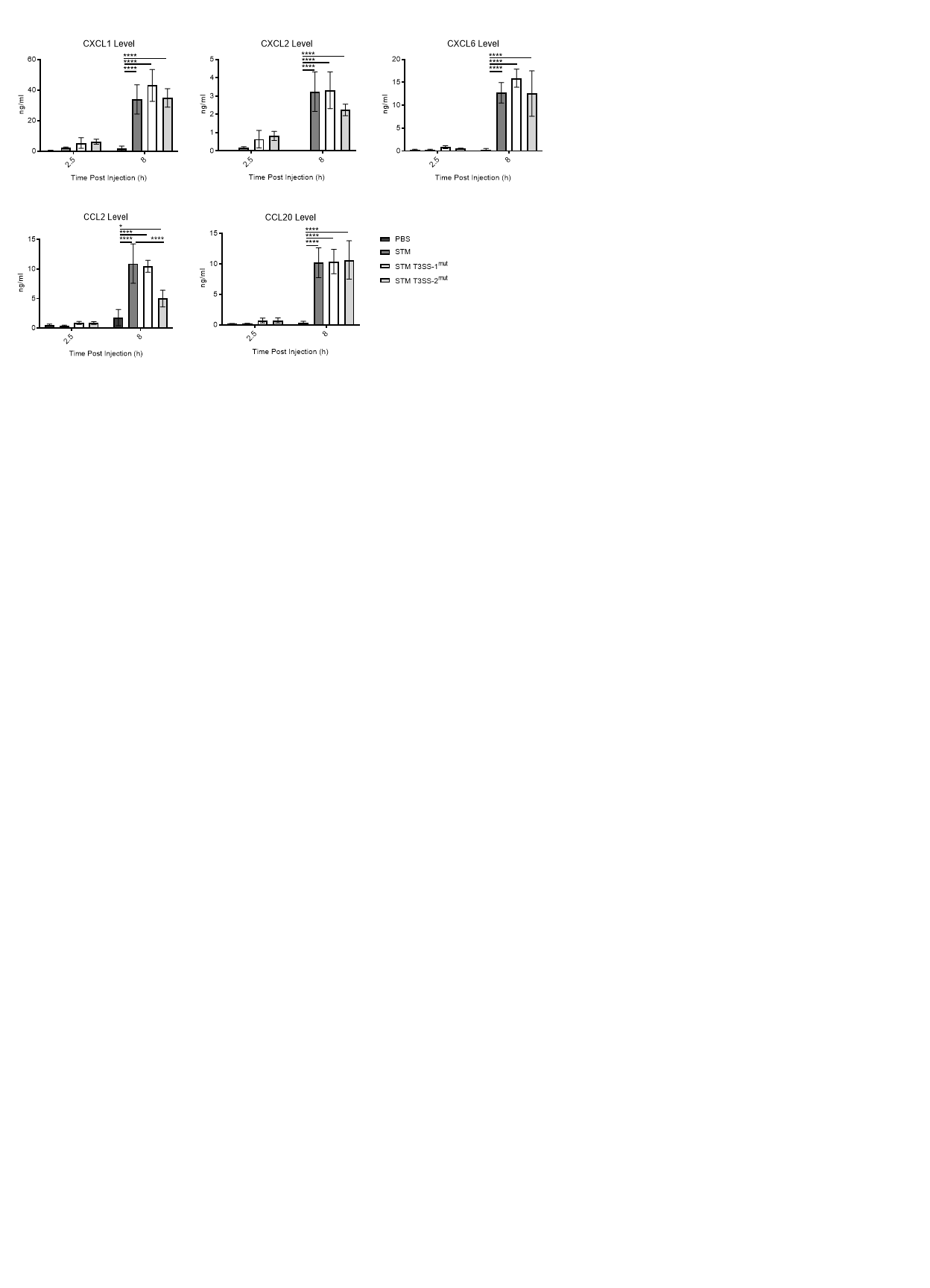

Supplement: FIG S3 [file mbio.00399-21-sf003.tif]
