# Supplementary material for: Salmonella enterica Serovar Typhimurium SPI-1 and SPI-2 Shape the Global Transcriptional Landscape in a Human Intestinal Organoid Model System
Source: mBio. 2021 May 18;12(3):e00399-21. doi: 10.1128/mBio.00399-21 (PMC8262845; doi:10.1128/mBio.00399-21)
Supplement: TABLE S1 [file mbio.00399-21-st001.pdf]

Table S1: Significant genes 2.5h pi

	STM		$T3SS\text{-}1^{mut}$		$T3SS\text{-}2^{mut}$	
**Symbol**	**log_2(fold change)**	**p-value**	**log_2(fold change)**	**p-value**	**log_2(fold change)**	**p-value**
CXCL2	6.615801663	9.63E-103	5.995814411	1.05E-84	6.833470917	1.65E-109
NFKBIZ	2.283908472	3.39E-74	2.060864042	9.56E-61	2.564145149	3.71E-93
CXCL1	6.194937458	4.87E-73	5.947029859	1.86E-67	6.548721662	2.10E-81
NFKBIA	3.975903931	4.06E-73	3.691413604	2.79E-63	4.549870409	3.28E-95
CXCL3	5.652319061	1.85E-72	4.964988877	2.57E-56	5.890110138	1.56E-78
BIRC3	5.882106382	1.39E-69	5.615398099	1.40E-63	6.316795726	5.60E-80
ZC3H12C	2.37057614	1.33E-69	2.014651477	1.12E-50	2.411422919	4.68E-72
ZC3H12A	4.20255591	7.84E-65	3.655866217	1.79E-49	4.325567388	1.33E-68
TNFAIP3	5.508226484	4.62E-63	5.19153728	3.24E-56	5.89952202	4.23E-72
CLUHP3	2.471734213	2.45E-61	1.913275982	2.91E-37	2.305551178	1.51E-53
CCL20	6.71265219	1.91E-55	6.873253041	4.79E-58	7.265895735	1.18E-64
CXCL6	6.620945383	3.59E-55	6.20349225	1.22E-48	6.935831804	2.10E-60
REL	2.536079009	1.81E-50	2.100696003	3.71E-35	2.564948913	1.31E-51
RCAN1	3.85744704	4.71E-50	3.082072188	1.46E-32	3.860944076	3.80E-50
C6orf222	4.474396198	6.83E-46	3.703168651	6.44E-32	4.922244067	3.04E-55
SOD2	2.881461485	1.44E-45	2.840800241	2.39E-44	3.222441562	1.48E-56
TNFAIP2	4.836014333	2.49E-45	4.2455662	2.51E-35	4.914563269	9.28E-47
CX3CL1	4.760616528	2.21E-44	3.946348435	5.77E-31	4.561988956	6.78E-41
MAP3K8	3.362067202	4.40E-42	3.067756941	2.56E-35	3.700260588	1.10E-50
CASP10	3.165107087	6.64E-42	2.392324157	1.37E-24	3.107411907	1.89E-40
IKBKE	2.043267184	2.71E-41	1.569320941	6.97E-25	1.968437373	1.86E-38
SDC4	3.217873309	8.20E-41	2.79016136	4.17E-31	3.5233877	1.39E-48
ICAM1	6.683670443	2.04E-40	6.291701515	5.20E-36	6.966666113	9.19E-44
CFLAR	1.992214216	4.61E-40	1.628968697	2.44E-27	2.173792709	2.11E-47
CXCL5	5.623953884	7.45E-37	5.732039344	3.21E-38	5.852963844	8.92E-40
CDC42EP2	2.022097185	2.53E-36	1.449846451	2.09E-19	1.915532294	8.53E-33
KLF6	2.101878825	2.52E-36	1.554964177	1.32E-20	2.167242559	1.53E-38
TIFA	2.977773533	3.42E-35	2.840566232	3.51E-32	3.296629563	7.67E-43
IL8	7.015250208	6.81E-34	6.765743631	1.21E-31	7.690652387	2.18E-40
NCOA7	2.775844734	5.66E-33	2.329936754	1.05E-23	3.162515264	2.47E-42
NFKB2	3.049506011	7.28E-33	2.43469427	1.61E-21	2.967445084	3.24E-31
ANKRD33B	5.243636897	1.96E-32	4.352997538	1.07E-22	4.66275499	7.48E-26
ABTB2	3.910688729	8.62E-31	3.423552396	5.83E-24	3.678602462	2.00E-27
MAFF	3.069516386	4.79E-30	2.729484993	4.34E-24	3.602199427	7.60E-41
DEFB4B	8.529450849	6.35E-30	7.247387411	5.10E-22	8.763269506	1.66E-31
RND1	5.312025187	2.41E-29	4.598626528	2.16E-22	5.708464338	1.22E-33
SLC6A14	4.009310783	3.70E-29	4.316768253	1.55E-33	4.63707372	1.94E-38
SGPP2	2.779400717	3.61E-27	2.349900241	7.23E-20	2.802115976	1.33E-27
IL1A	7.59718865	2.84E-26	7.267940362	3.50E-24	8.374733915	1.43E-31
NFKB1	2.111039718	1.95E-25	1.898030267	7.22E-21	2.306178457	4.60E-30
BCL3	2.739360509	2.14E-25	1.997196354	4.17E-14	2.405858959	6.60E-20
CEBPD	3.449835774	2.20E-25	2.907407403	1.78E-18	3.536643017	1.31E-26
IRF1	2.043041475	2.23E-25	1.917409367	1.41E-22	2.617667643	7.90E-41
LDLR	1.177490947	2.08E-25	0.85774379	3.28E-14	1.293437906	2.44E-30

IRAK2	3.649313339	3.62E-25	3.232457477	4.44E-20	3.636873406	5.10E-25
HDAC9	3.10336985	1.24E-24	2.713615117	3.42E-19	3.783733153	6.33E-36
SAV1	2.291922907	3.24E-24	2.076529676	3.59E-20	2.981172423	5.98E-40
SGK1	2.600118244	6.30E-24	2.29452685	5.56E-19	2.486597332	5.07E-22
IFNGR1	1.740105298	1.88E-23	1.660597783	1.64E-21	2.024654967	3.34E-31
ZFAND5	0.804447457	1.85E-23	0.445129401	3.37E-08	0.757558747	5.16E-21
VNN3	3.63693001	4.65E-23	2.956549975	1.63E-15	3.869040659	4.24E-26
DUSP5	3.217158659	6.73E-23	2.758163632	3.03E-17	3.001526155	3.88E-20
CD69	4.806380611	1.21E-22	4.796183442	1.44E-22	4.910346182	1.40E-23
LIPG	1.76816937	3.58E-22	1.369567453	6.50E-14	1.743506897	1.30E-21
C2CD4A	3.7901461	4.33E-22	3.240596136	1.54E-16	4.394334358	3.15E-29
JUND	1.341976453	8.32E-22	0.616231799	1.13E-05	1.078270284	1.32E-14
RNF145	1.019456121	1.58E-21	0.854453817	1.37E-15	1.203756122	1.70E-29
TMPRSS2	2.401348235	1.65E-21	2.034702095	7.03E-16	2.508295612	2.51E-23
CACNG8	1.718047724	3.05E-21	1.39484502	1.83E-14	1.829832619	3.51E-24
ADAMTS9	2.168377076	5.79E-21	1.751245438	3.30E-14	2.001807302	4.24E-18
SQSTM1	1.797744679	6.94E-21	1.454706028	3.32E-14	1.886275548	7.69E-23
ITGB8	1.850364788	7.89E-21	1.593186883	7.59E-16	1.926837655	1.80E-22
TICAM1	3.166682874	1.30E-20	2.490350932	2.93E-13	3.364775696	3.81E-23
TNFSF14	3.302721875	1.38E-20	3.06431748	5.45E-18	3.026265337	1.79E-17
NINJ1	1.942390635	1.55E-20	1.467916694	2.36E-12	1.900623672	9.52E-20
PROX1	2.840503868	1.84E-20	2.820695987	3.23E-20	2.901678581	2.64E-21
CXCL10	5.822795639	3.28E-20	4.866426642	1.46E-14	5.354534231	2.53E-17
OAS3	1.446742372	3.66E-20	0.746660091	2.31E-06	1.284356491	3.23E-16
STAT5A	2.992247442	4.05E-20	2.550995087	4.96E-15	3.129833415	6.61E-22
IL17C	9.49583194	5.04E-20	8.8160113	1.80E-17	9.847392627	2.03E-21
BBC3	2.942048984	6.53E-20	2.84412654	9.77E-19	3.384717438	5.72E-26
SLC2A6	2.766167786	7.99E-20	2.396886403	3.19E-15	2.957911657	1.49E-22
CSF3	10.81361385	1.25E-19	10.01623876	4.65E-17	11.82422423	3.65E-23
CYP3A5	1.952226902	2.22E-19	1.394407272	1.30E-10	2.146270338	4.19E-23
TUBB2A	1.375637374	2.33E-19	0.950254549	5.45E-10	1.516212734	2.95E-23
HIVEP1	1.277857282	3.11E-19	0.975524137	8.24E-12	1.15693279	5.04E-16
IL23A	4.783281807	3.29E-19	4.133386066	1.14E-14	5.451023371	1.29E-24
IL7	4.130611548	3.28E-19	3.485005796	4.77E-14	4.177456127	1.03E-19
PLK2	2.029899193	3.39E-19	1.645954817	3.89E-13	2.147615835	2.63E-21
HIVEP2	1.720344629	3.60E-19	1.189534051	6.39E-10	1.427808407	1.14E-13
IL17RB	1.579097353	4.69E-19	0.953895112	8.47E-08	1.178416109	3.26E-11
ZNFX1	0.972698453	4.88E-19	0.605396045	2.98E-08	0.732858952	1.89E-11
BAZ1A	1.208574289	7.15E-19	0.908131392	2.66E-11	1.350951511	3.29E-23
IL1B	5.768983172	8.32E-19	4.973907374	2.31E-14	6.152869922	3.48E-21
ETV3	1.013376981	8.95E-19	0.626763563	4.68E-08	1.036520304	1.24E-19
LIF	2.688049691	1.17E-18	2.548245686	6.32E-17	3.420338284	2.94E-29
BCL10	1.1391055	1.31E-18	0.730812598	1.69E-08	1.073281794	1.03E-16
LTB	4.463330623	1.43E-18	4.528176703	3.85E-19	4.047184601	1.64E-15
TNIP1	1.533268357	2.07E-18	1.319123953	5.09E-14	1.647833987	4.98E-21
EFNB1	1.296846898	3.12E-17	1.031936517	1.86E-11	1.321744305	7.13E-18
B4GALT5	0.80779472	5.47E-17	0.425304768	1.06E-05	0.771714295	1.16E-15

BMP2	2.469997358	2.09E-16	1.772195086	3.80E-09	2.622884854	2.62E-18
PLAUR	1.864478113	2.11E-16	1.339539119	3.81E-09	2.126303282	6.52E-21
SPRR2A	7.137167727	3.74E-16	4.852018191	3.88E-08	7.111741313	4.69E-16
CSF1	2.033777985	4.37E-16	1.741887377	3.44E-12	2.241866236	2.80E-19
PIM2	1.885268375	4.34E-16	1.435874457	6.42E-10	1.889623929	3.46E-16
HS3ST1	2.065059483	5.48E-16	1.398284623	4.29E-08	2.113505853	9.98E-17
EPHA2	1.586905022	8.66E-16	1.327110397	1.72E-11	1.991459165	5.07E-24
OXTR	2.413350757	8.64E-16	2.109295435	2.09E-12	2.504080697	5.68E-17
ADAR	0.445101289	9.00E-16	0.214220207	0.0001081	0.239002553	1.57E-05
NEDD4L	1.357556998	1.33E-15	1.123878736	3.74E-11	1.379243009	4.69E-16
TRAF3	1.158072703	1.32E-15	0.788739693	5.34E-08	1.006912671	3.59E-12
B3GNT5	1.119425574	1.34E-15	0.763780203	5.02E-08	1.237924842	8.56E-19
ZBTB38	1.102221995	1.40E-15	0.801277409	6.33E-09	1.145782876	9.71E-17
TRIM16	1.414374978	1.54E-15	1.135643937	1.57E-10	1.627370751	4.15E-20
IFNGR2	1.436482993	1.70E-15	1.2540248	3.64E-12	1.707774747	2.81E-21
EHD1	1.87520531	1.79E-15	1.491451057	2.64E-10	1.934493989	2.08E-16
ARRDC3	2.514627345	1.83E-15	2.823643728	4.20E-19	3.397792432	5.96E-27
C8orf4	3.119492729	2.10E-15	3.108257851	2.54E-15	3.780162998	5.85E-22
IL18R1	1.899346031	3.13E-15	1.416550349	4.43E-09	2.032623962	2.88E-17
IRGQ	0.698095991	3.17E-15	0.649000823	1.97E-13	0.853556039	3.64E-22
STX11	4.833583142	3.34E-15	4.095553438	3.04E-11	4.830503048	3.26E-15
FAM222A	1.945554799	3.95E-15	1.374464716	3.27E-08	1.852568205	7.16E-14
ICOSLG	2.407164067	5.07E-15	1.896141998	7.63E-10	2.08647827	1.24E-11
ANKRD36C	1.955679169	5.90E-15	1.639283641	6.06E-11	2.41491327	5.16E-22
DCUN1D3	1.071046101	8.17E-15	0.677999624	9.31E-07	0.906070302	5.16E-11
IER3	2.589488192	8.80E-15	2.836606666	1.92E-17	3.257539559	1.65E-22
NFKBIE	2.247033536	9.49E-15	1.889068799	7.76E-11	2.005839457	5.02E-12
PPP1R3B	-1.26599918	9.66E-15	-1.094206136	1.59E-11	-0.695430549	1.73E-05
RAB11FIP1	1.356397558	1.36E-14	0.762980218	1.49E-05	1.73767542	5.60E-23
KYNU	2.657295891	1.66E-14	2.411417956	3.21E-12	3.460928934	1.41E-23
KBTBD7	-0.821056125	2.86E-14	-0.804196347	3.17E-14	-1.116211203	1.91E-24
KLHL25	1.260125494	3.02E-14	0.789250809	2.19E-06	0.742756376	9.07E-06
ZNF217	0.787014183	3.80E-14	0.502040924	1.38E-06	0.759323894	2.58E-13
PPAP2B	1.423781217	4.27E-14	1.219609462	9.79E-11	1.359410267	5.50E-13
TRIM69	1.42042355	4.43E-14	1.01200944	7.66E-08	1.558526559	1.02E-16
NABP1	1.262692142	4.49E-14	1.11462908	2.69E-11	1.231423651	1.81E-13
TNFAIP8	1.686401449	8.89E-14	1.828557562	5.85E-16	2.063157812	6.68E-20
FAM101A	2.634837695	1.35E-13	1.593958496	7.69E-06	2.842051189	1.41E-15
BID	1.61192077	1.47E-13	1.081591523	7.25E-07	1.631741958	7.17E-14
PPAPDC2	1.283805349	1.46E-13	1.020159921	4.35E-09	1.496010025	4.94E-18
FRAT2	-1.373859344	1.55E-13	-0.802601603	8.53E-06	-0.95093707	1.68E-07
OTUD1	1.501738959	1.54E-13	1.264826472	5.01E-10	1.873414242	2.24E-20
TNF	7.16562878	1.70E-13	6.714586769	5.10E-12	7.564796441	6.73E-15
TBC1D10A	1.201586133	2.03E-13	0.749296659	5.03E-06	1.211769378	1.15E-13
ARL14	1.983015286	2.13E-13	1.87303227	4.00E-12	2.732875577	3.49E-24
ZNF607	-0.894342003	2.39E-13	-0.684932104	4.84E-09	-0.82963225	3.60E-12
SPRR1A	5.167015134	2.41E-13	4.373662409	6.35E-10	5.87424361	6.84E-17

NFKBID	1.534520353	3.24E-13	1.056383113	6.97E-07	1.617518544	1.12E-14
PPP1R15A	1.805073916	3.30E-13	1.505804327	1.25E-09	2.261754278	6.58E-20
CT83	4.73743086	3.33E-13	4.265260345	6.34E-11	4.783819691	1.75E-13
BTG2	1.498646555	4.24E-13	0.958905981	3.58E-06	1.789989062	4.62E-18
SLC37A1	0.971662431	4.22E-13	0.757810148	1.54E-08	1.222402675	5.33E-20
ANKRD36	1.125867938	4.95E-13	0.691862672	9.10E-06	1.37486582	8.88E-19
IL32	2.245783163	6.44E-13	2.346714733	5.05E-14	2.190104849	2.30E-12
IER5	1.383515185	7.34E-13	1.036450451	8.12E-08	1.694213855	9.82E-19
NFKBIB	1.401633246	8.77E-13	1.165880519	2.84E-09	1.48888456	2.48E-14
CLEC2D	1.824600999	1.17E-12	1.831161828	7.76E-13	2.418754435	2.05E-21
RAB9A	1.513924455	1.58E-12	0.909961789	2.54E-05	1.291440963	1.73E-09
IFNAR2	1.428858611	1.59E-12	1.368271722	1.30E-11	1.58545147	4.06E-15
MOB3C	1.134721146	1.64E-12	0.754743613	2.82E-06	1.079520009	1.72E-11
PPP1R15B	0.904193555	1.99E-12	0.415025533	0.0012516	0.988892303	1.38E-14
UBQLN4	0.878627957	2.00E-12	0.432085948	0.0005556	0.731674331	4.68E-09
ZBTB10	1.17372463	2.23E-12	0.926670754	2.99E-08	1.285947593	1.39E-14
DEFB4A	8.888424608	2.69E-12	8.33915611	5.49E-11	9.594172673	4.16E-14
ZNF792	-1.089814875	2.96E-12	-0.908642966	3.00E-09	-1.118093968	6.50E-13
PHLDA1	1.463355019	3.12E-12	1.117088502	1.03E-07	1.340050167	1.72E-10
SAT1	1.795936683	3.13E-12	1.37746133	8.94E-08	2.052643046	1.60E-15
RIPK2	1.417438382	3.91E-12	1.175879377	8.43E-09	1.670935001	2.31E-16
KLRK1	2.183328226	3.98E-12	1.66397262	1.49E-07	1.957814546	5.52E-10
RAB4B	1.504651145	4.73E-12	1.082318433	6.79E-07	1.282562721	3.85E-09
ALAS1	1.126055274	5.08E-12	0.771644505	2.24E-06	0.977844969	2.02E-09
₹P11-986E7.	3.28200507	5.65E-12	2.853430792	2.42E-09	3.822380251	5.34E-16
CCNL1	0.864911449	6.33E-12	0.480526949	0.0001364	0.725047953	8.41E-09
IL19	6.968164513	6.56E-12	4.660958545	5.81E-06	6.628798486	6.64E-11
MFHAS1	1.016501617	6.69E-12	0.618748652	2.88E-05	0.822311909	2.66E-08
LCN2	5.63821581	7.06E-12	4.91781109	2.24E-09	6.247922505	2.98E-14
JAK2	1.542833051	7.67E-12	1.275899619	1.50E-08	1.905327545	2.53E-17
IL4R	1.057477395	8.06E-12	0.809209704	1.72E-07	1.237795502	1.06E-15
GJB2	2.244405452	8.62E-12	2.083563037	2.29E-10	3.107120374	2.47E-21
DUSP16	1.335715473	9.59E-12	1.247685915	1.95E-10	1.350296881	5.35E-12
SOWAHB	1.144275144	9.77E-12	0.444596568	0.0085099	1.186744189	1.49E-12
SPRR2F	6.507675014	1.02E-11	4.666644693	1.28E-06	6.97785579	2.79E-13
GCNT3	1.736908116	1.07E-11	1.224461892	1.66E-06	1.70430326	2.57E-11
PTS	0.932116402	1.09E-11	0.826123311	1.50E-09	1.05213571	1.19E-14
TRIM31	2.50337826	1.29E-11	1.993799514	7.10E-08	2.480050721	1.95E-11
RAB3IP	1.072379338	1.35E-11	0.894189737	1.78E-08	1.398492122	9.77E-19
CCSAP	-0.576623256	1.47E-11	-0.258460576	0.0015	-0.477679357	9.99E-09
C3	2.25746585	1.50E-11	2.558049102	2.06E-14	1.948239616	5.79E-09
NFATC2	1.666323776	1.59E-11	1.126197009	5.44E-06	1.422162315	8.56E-09
RND3	1.06639598	1.73E-11	0.973202405	8.37E-10	1.578307685	2.12E-23
DUSP4	1.283231304	1.94E-11	0.782968733	4.29E-05	1.222559908	1.60E-10
MESDC1	0.837385314	2.27E-11	0.662303892	1.16E-07	1.056348954	1.56E-17
ANKLE2	0.824629444	2.56E-11	0.453774674	0.0002422	0.811576083	4.99E-11
FAM43A	3.132223489	3.19E-11	3.183230831	1.44E-11	3.224346591	7.93E-12

LAMC2	1.255188588	4.04E-11	0.899875345	2.21E-06	1.291588986	1.09E-11
CCRN4L	1.529825708	4.10E-11	1.281651273	3.15E-08	1.815460616	3.88E-15
TRAF3IP2	0.974070887	4.28E-11	0.620946029	2.69E-05	0.983507695	2.60E-11
ZBTB21	0.955640064	4.57E-11	0.657961583	6.01E-06	1.066223729	1.76E-13
PELI1	0.905510036	5.15E-11	0.668620227	1.25E-06	1.189503632	5.57E-18
SH2B3	1.425091757	5.39E-11	1.119157794	2.58E-07	1.155153291	1.08E-07
HES1	-0.848260386	5.52E-11	-0.595427281	3.23E-06	-0.69409725	6.46E-08
PPP1R3D	-0.962579607	6.78E-11	-0.66605357	1.56E-06	-0.963250707	3.07E-11
SMAD3	1.28481985	6.99E-11	1.074402871	4.97E-08	1.272329719	1.07E-10
ZFP36	1.677238543	7.05E-11	1.378153754	8.50E-08	2.228898169	3.95E-18
RBBP8NL	-1.918982353	7.75E-11	-2.257535596	3.13E-14	-2.104709877	1.16E-12
NUAK2	2.165806343	7.99E-11	2.219619243	2.18E-11	1.612687794	1.58E-06
GPR37L1	1.660161214	8.24E-11	1.256368307	1.08E-06	1.700059671	2.02E-11
UBASH3A	4.813833876	8.83E-11	4.097832555	3.66E-08	5.207411697	1.83E-12
BTG1	1.088425096	1.06E-10	0.595834498	0.0004066	0.993227405	3.70E-09
CAB39	0.65783769	1.26E-10	0.31879077	0.0018533	0.570594868	2.38E-08
CFB	1.46576135	1.42E-10	1.186378557	2.08E-07	1.377373383	1.67E-09
DUSP6	1.2212528	1.47E-10	1.193204725	3.79E-10	1.514699579	1.82E-15
NEURL3	5.227028624	1.77E-10	5.158655847	2.92E-10	5.630088179	5.40E-12
SLC5A5	2.272959365	2.05E-10	1.499206058	3.60E-05	2.005871844	1.91E-08
FNBP1	0.757392907	2.73E-10	0.605142086	4.72E-07	0.657864321	4.42E-08
TAF4B	1.299543664	2.75E-10	1.156973663	1.79E-08	1.46862062	7.96E-13
F2RL1	1.008342722	2.81E-10	0.621986075	0.0001002	1.082220694	1.25E-11
KLHL5	0.639434179	3.23E-10	0.704646167	3.12E-12	0.676271788	2.41E-11
CCL2	3.204770151	3.25E-10	2.888227769	1.47E-08	3.428222042	1.75E-11
P1-145M24	3.524064556	3.59E-10	3.575541938	1.54E-10	4.365708664	2.81E-15
TMEM217	2.3892582	3.71E-10	1.966553962	2.61E-07	2.668772758	1.53E-12
TNFRSF10E	0.988747421	3.77E-10	0.638679877	5.23E-05	0.935526939	3.09E-09
BACH1	0.856832844	3.91E-10	0.636225289	3.34E-06	0.736417491	7.46E-08
GPBP1	0.457963598	4.86E-10	0.232950743	0.0015424	0.452558665	7.09E-10
AC159540.1	2.614350848	6.20E-10	1.795014171	2.85E-05	3.448331599	1.21E-16
TLE4	0.855814881	6.18E-10	0.39938633	0.0039895	0.757438187	4.44E-08
SOCS3	2.398482444	6.26E-10	2.776440052	7.86E-13	3.091275678	1.49E-15
TNFRSF9	7.655762415	7.55E-10	7.27578424	4.97E-09	7.761165932	4.26E-10
RELB	3.865862186	1.05E-09	3.222935703	3.77E-07	3.997406631	2.71E-10
CTTNBP2NL	0.670808282	1.35E-09	0.489268343	9.83E-06	0.511855271	3.72E-06
TRIP10	0.737189045	1.40E-09	0.382561169	0.0016843	0.604925245	6.48E-07
TRIB1	1.069964371	1.45E-09	0.576748408	0.0011202	0.906990833	2.88E-07
UBD	2.641294127	1.47E-09	3.309822285	2.84E-14	4.224081434	2.38E-22
NFATC1	2.390811127	1.50E-09	2.331744882	3.62E-09	2.286746747	7.50E-09
PPP4R2	0.632660838	1.53E-09	0.498160949	1.92E-06	0.788154027	4.81E-14
CCDC130	0.595720303	1.78E-09	0.242902758	0.0152037	0.344753486	0.000554
MED24	0.780437562	1.83E-09	0.107033723	0.4126955	0.605543496	3.00E-06
PMAIP1	1.665596092	1.98E-09	1.388194733	5.77E-07	2.020772713	3.05E-13
CLIP2	1.035375455	2.14E-09	0.694729574	5.94E-05	0.917532095	1.13E-07
PRRG1	1.389641439	2.17E-09	1.119496328	1.45E-06	1.359707986	4.77E-09
TAPBP	0.7437541	2.25E-09	0.250906562	0.0439722	0.562078979	6.22E-06

CPEB2	1.16870669	2.28E-09	0.906131287	3.60E-06	1.298885246	2.75E-11
RARRES1	1.892233002	2.62E-09	1.817732867	1.03E-08	1.665021437	1.63E-07
ZKSCAN1	-0.665105788	2.62E-09	-0.348228563	0.0014481	-0.555314992	4.60E-07
ZNF697	1.014635296	3.99E-09	1.104461071	1.28E-10	0.938767669	5.06E-08
SGMS2	1.120511235	4.47E-09	0.829473751	1.42E-05	1.319338693	4.81E-12
TXNRD1	0.839416297	4.91E-09	0.834109712	5.92E-09	1.101646781	1.49E-14
JAG1	1.095774712	4.97E-09	0.8746941	3.04E-06	1.306357233	3.05E-12
PATL1	0.724817987	5.47E-09	0.125498865	0.316534	0.422077799	0.000711
TYK2	0.650297657	5.52E-09	0.238917487	0.0327582	0.429939737	0.00012
HIPK1	0.560260926	5.58E-09	0.236424298	0.0139777	0.331306257	0.00057
GRAMD4P7	2.115499727	5.87E-09	1.511243537	4.09E-05	1.9317945	1.11E-07
PHKG2	0.667345954	5.96E-09	0.438670345	0.0001296	0.640342656	2.02E-08
TTC9	1.0829132	6.01E-09	0.758539455	4.71E-05	1.273290235	6.64E-12
BEND3	1.278670641	6.07E-09	0.788811412	0.0003395	1.708615402	6.96E-15
NAMPT	1.846051828	6.18E-09	1.80080379	1.43E-08	2.409131394	3.28E-14
SLC52A3	1.352317058	6.40E-09	1.307636541	1.96E-08	1.062076684	5.50E-06
ING2	-0.830460204	6.55E-09	-0.581003921	3.00E-05	-0.49302613	0.000394
PRDM1	1.582649491	6.57E-09	1.224131755	7.59E-06	1.594915005	5.01E-09
NR4A1	1.835234274	6.64E-09	1.195537697	0.0001624	1.608111427	3.76E-07
VMP1	0.680869159	7.16E-09	0.424680509	0.0003067	0.703953103	2.12E-09
ZSWIM4	1.280901412	9.53E-09	0.910239122	4.78E-05	1.083420927	1.34E-06
MTND4P23	3.805654512	1.00E-08	2.756060518	4.98E-05	4.192298737	1.85E-10
TLR4	1.184060272	1.05E-08	0.354571223	0.0871369	0.911900932	1.05E-05
PLSCR1	0.723644117	1.10E-08	0.416624474	0.0010047	0.923150591	2.69E-13
CXCL16	1.097651813	1.14E-08	0.857618853	8.17E-06	1.190200463	5.59E-10
PRKAB2	-0.972853978	1.15E-08	-0.85271716	4.53E-07	-1.096106068	1.15E-10
DUSP1	2.115196352	1.20E-08	1.384340039	0.0001955	2.672579836	5.57E-13
FRK	0.841036073	1.20E-08	0.498683243	0.0007285	0.808829005	4.20E-08
SLC8B1	1.318449818	1.21E-08	1.055542539	5.32E-06	1.315043526	1.26E-08
ATF3	2.705966196	1.26E-08	1.814395133	0.0001379	2.807778472	3.47E-09
LAMA3	1.298037566	1.31E-08	0.932220463	4.45E-05	1.656557036	3.97E-13
EHF	1.299161433	1.33E-08	1.318125913	8.05E-09	1.622403196	1.23E-12
ITPKC	1.09716854	1.36E-08	0.563327527	0.0036027	1.373723977	1.08E-12
B4GALT1	1.115604832	1.62E-08	0.350566392	0.076336	1.118250318	1.48E-08
MACC1	0.759313775	1.79E-08	0.482325735	0.0003476	1.178303785	2.14E-18
LPIN1	0.82146875	1.82E-08	0.631687233	1.51E-05	0.846032183	6.68E-09
GCNT4	1.230691352	1.84E-08	0.868443489	7.31E-05	1.499285467	6.19E-12
NRARP	1.853718454	2.14E-08	1.43238863	1.62E-05	2.169147339	4.04E-11
ICAM4	2.267458828	2.24E-08	1.847512795	5.95E-06	2.46710959	8.22E-10
RAC1	0.650513785	2.32E-08	0.316889218	0.0065096	0.704796793	1.40E-09
PSEN1	0.490822145	2.55E-08	0.294551889	0.0008256	0.596513973	1.14E-11
NFE2L2	0.71177422	2.63E-08	0.258679319	0.043479	0.617672486	1.36E-06
C22orf29	-0.708600679	2.68E-08	-0.791847032	4.14E-10	-0.958504235	6.82E-14
USP54	0.829382997	2.77E-08	0.150795747	0.3137671	0.989850473	3.19E-11
AASDHPPT	-0.547715944	3.21E-08	-0.133858089	0.1704118	-0.341783537	0.000514
HBEGF	1.916802913	3.41E-08	1.895139381	4.72E-08	2.329537797	1.78E-11
ZNF174	-0.881269003	3.55E-08	-0.539262622	0.0004774	-0.802366138	3.53E-07

CREB1	0.749389686	3.96E-08	0.341271278	0.0123417	0.504252369	0.000218
FUT2	0.879864821	3.99E-08	0.550908894	0.0005916	0.919375763	9.18E-09
SOWAHC	-0.694632914	3.98E-08	-0.396164633	0.0015032	-0.721962007	1.03E-08
BTG3	0.887944717	4.10E-08	0.811685872	5.04E-07	1.246044012	1.02E-14
KBTBD6	-0.702569004	4.32E-08	-0.556316151	1.06E-05	-0.912373864	1.33E-12
MRFAP1L1	-0.496605297	4.38E-08	-0.315207306	0.0004275	-0.336419976	0.000181
FOSL1	1.717539229	4.45E-08	0.979756625	0.0019015	1.486166963	2.27E-06
RASD1	2.596289802	4.45E-08	1.861609769	0.0001113	3.036521242	9.13E-11
HAUS3	-0.561782037	4.89E-08	-0.498939248	9.13E-07	-0.475616581	3.22E-06
LRRC49	1.403713636	4.94E-08	1.206661138	2.58E-06	1.43634038	2.12E-08
PLAU	3.011383875	5.48E-08	3.013552082	5.34E-08	3.715226397	1.97E-11
STEAP2	0.890231049	5.49E-08	0.57300105	0.0004859	0.830116149	3.91E-07
ETS1	0.828584335	5.82E-08	0.766536548	5.12E-07	0.834011362	4.68E-08
DUSP8	2.94146364	6.29E-08	2.85018372	9.22E-08	3.064439498	1.40E-08
KITLG	-0.996424683	6.86E-08	-0.954224965	2.24E-07	-1.167569012	2.58E-10
DYRK2	0.657999406	6.99E-08	0.289929645	0.0175404	0.478167522	8.93E-05
CCDC66	-0.773949121	7.28E-08	-0.197009924	0.1608984	-0.722537048	4.06E-07
SERPINA3	2.978587362	7.66E-08	3.245184925	4.73E-09	3.832527153	4.63E-12
LRRC8A	0.624346602	7.72E-08	0.295648459	0.011055	0.633840784	4.65E-08
MYADM	0.800240417	7.99E-08	0.20401071	0.1735385	0.285141618	0.057176
AHCYL2	1.178923757	8.02E-08	0.82335912	0.0001809	1.158976143	1.32E-07
CFL2	0.832830221	8.04E-08	0.817978806	1.27E-07	0.983586017	2.07E-10
C3orf58	-0.540513232	8.24E-08	-0.518160899	2.33E-07	-0.560842712	2.39E-08
RNF19A	1.249945501	9.21E-08	1.492239371	1.54E-10	1.811394649	6.04E-15
SLFN5	1.430774817	9.41E-08	1.514522129	1.57E-08	2.026438555	3.75E-14
RNF43	-1.490723998	9.51E-08	-1.051631069	0.0001556	-1.619627811	6.86E-09
ZNF267	0.588055518	9.77E-08	0.278048495	0.0117295	0.614726242	2.34E-08
SESTD1	0.743611853	1.02E-07	0.709083993	3.70E-07	1.029678983	1.49E-13
MCMDC2	-0.8322481	1.03E-07	-0.600657708	5.19E-05	-0.561690769	0.000166
ZFP30	-0.825277026	1.04E-07	-0.473723433	0.0019567	-0.815814575	1.21E-07
TPI1P3	2.895633959	1.07E-07	2.314034875	2.77E-05	2.78348557	3.16E-07
USP12	0.614215359	1.09E-07	0.232304758	0.0448499	0.591418999	3.08E-07
ERRFI1	1.619558769	1.10E-07	1.405031856	4.09E-06	2.239093124	2.09E-13
SLC30A1	-0.63295571	1.16E-07	-0.849302127	1.15E-12	-0.853374084	9.34E-13
ZNF253	-0.640727605	1.31E-07	-0.468585958	9.16E-05	-0.78487761	1.12E-10
CYTH1	0.723495406	1.36E-07	0.593597511	1.57E-05	0.871646591	1.94E-10
VPS9D1	1.248689778	1.39E-07	0.431389253	0.0709467	0.699070153	0.003181
SDCBP	1.03531459	1.41E-07	0.803469866	4.42E-05	1.412955875	6.61E-13
CEBPA	-1.300896884	1.52E-07	-1.362752169	3.54E-08	-1.912418737	1.87E-14
SNRPF	-0.768523008	1.53E-07	-0.240108701	0.0961112	-0.094893115	0.510765
IFIH1	0.952291189	1.63E-07	0.69075445	0.0001464	1.110248142	8.17E-10
MAFK	0.868532604	1.73E-07	0.375712898	0.0244493	1.031169817	4.18E-10
ZNF710	0.664737192	1.84E-07	0.278181805	0.029445	0.469342086	0.000237
CCDC51	-0.743184772	2.15E-07	-0.473686585	0.0004982	-0.721481182	2.77E-07
GPX3	1.672737396	2.36E-07	1.230266183	0.0001426	1.415040204	1.29E-05
ELK3	0.984042883	2.61E-07	0.944923943	7.42E-07	1.049892082	3.68E-08
PANX1	0.572156316	2.71E-07	0.41287399	0.0002011	0.641937789	6.78E-09

RDH10	0.903395368	2.92E-07	0.918421962	1.83E-07	1.240904457	1.78E-12
GTPBP2	0.575464255	2.94E-07	0.46704615	3.10E-05	0.81512097	2.63E-13
ZNF766	-0.526620197	3.23E-07	-0.152099941	0.1299776	-0.317348528	0.001714
CXCL11	5.722261787	3.29E-07	4.838381784	1.85E-05	5.479202179	1.05E-06
ETAA1	-0.657238162	3.36E-07	0.020132323	0.8720049	-0.285082317	0.024078
DBF4	-0.591464827	3.45E-07	-0.299862349	0.0088777	-0.264338741	0.021248
TRIM47	1.680509902	3.45E-07	1.348835343	4.11E-05	1.234699242	0.000188
EGR1	1.330791409	3.56E-07	0.995296206	0.0001412	0.891069382	0.000662
PPP2CB	0.473796803	3.58E-07	0.342302414	0.0002333	0.271286697	0.003609
DUSP10	0.897423832	3.59E-07	0.741769928	2.46E-05	0.931084417	1.13E-07
MAPK6	0.426010333	3.60E-07	0.354787006	2.19E-05	0.558950619	2.18E-11
NMNAT2	1.859408589	3.81E-07	1.449724432	7.85E-05	1.894071176	2.16E-07
DENND4A	0.752253966	3.83E-07	0.529649289	0.0003487	0.94623131	1.56E-10
SAC3D1	-2.114226336	3.99E-07	-0.557240975	0.1495915	-0.722900914	0.063599
SPIDR	-0.61576584	4.26E-07	-0.448393014	0.0002339	-0.46277113	0.00014
C5orf45	0.708451767	4.27E-07	0.683451222	9.53E-07	0.586636279	2.77E-05
NEDD9	1.152209049	4.31E-07	1.041572179	4.84E-06	1.620205707	1.09E-12
C2CD4B	2.151849374	4.65E-07	1.447759753	0.0007571	2.47479122	5.68E-09
NCEH1	1.098277895	4.76E-07	0.840640885	0.0001191	1.265996913	6.34E-09
MYCN	-1.784631558	5.03E-07	-1.276923096	0.0002201	-2.131101896	2.82E-09
SERPINB8	1.22442184	5.12E-07	0.981958127	5.69E-05	1.352435815	2.84E-08
PATZ1	-0.568162239	5.27E-07	-0.58581544	1.55E-07	-0.788620952	3.44E-12
CDK17	0.695045642	5.58E-07	0.543480324	8.49E-05	0.524612201	0.00015
FRMD8	1.063175104	5.60E-07	0.630107715	0.0030484	1.027485923	1.28E-06
LRG1	1.157162351	5.69E-07	0.517318757	0.0256991	0.981092876	2.25E-05
S100A3	2.604319402	5.71E-07	2.501505348	1.44E-06	3.023891799	4.21E-09
NAMPTL	1.793967566	5.74E-07	1.881580045	1.55E-07	2.387956768	2.72E-11
BCAR1	0.848444237	6.05E-07	0.633911566	0.0001948	0.856519418	4.73E-07
ZSCAN20	-0.70081908	6.24E-07	-0.512397505	0.0001137	-0.757873842	3.20E-08
C17orf80	-0.540339135	6.28E-07	-0.398706878	0.0002194	-0.622552246	1.02E-08
PLEKHA4	1.049647449	6.65E-07	0.582760567	0.0059292	0.612614823	0.0038
ELF3	1.057401973	6.70E-07	0.722523413	0.0006856	1.592869215	6.85E-14
RAP1B	0.418711202	6.71E-07	0.510589956	1.22E-09	0.593676315	1.58E-12
CYLD	0.661873488	6.84E-07	0.599037668	6.46E-06	0.648540368	1.07E-06
LAMB3	0.963589844	6.91E-07	0.53679573	0.0056951	1.157900506	2.42E-09
ZNF429	-0.632636302	7.56E-07	-0.531802134	2.45E-05	-0.509943863	5.06E-05
CSRNP1	1.229016206	7.72E-07	0.855385291	0.0005867	1.537132028	5.95E-10
DCBLD1	-0.730166756	8.07E-07	-0.378577232	0.0093355	-0.131036384	0.366396
FANCF	-0.615975654	8.38E-07	-0.26778872	0.0259909	-0.543853688	9.99E-06
ZNF530	-0.783813335	8.44E-07	-0.706113867	4.55E-06	-0.863998954	3.10E-08
GPAM	-0.605166562	9.21E-07	-0.473047922	0.000102	-0.682413396	2.89E-08
BCL9L	1.261461903	9.47E-07	0.67747308	0.0086534	0.805959747	0.001761
NONOP2	1.389766752	1.04E-06	0.993517885	0.0005373	1.309582088	3.92E-06
PTPRH	1.094784847	1.09E-06	0.397175397	0.0775974	1.082951069	1.40E-06
RASGRP1	1.463668467	1.10E-06	1.504507172	5.15E-07	2.055818961	6.04E-12
UBE2Z	0.392512562	1.15E-06	0.151281865	0.0611351	0.27414719	0.00067
OSBPL7	-1.13128222	1.16E-06	-1.267797362	5.30E-08	-1.013024942	1.24E-05

PIEZO1	0.975993628	1.18E-06	0.791433153	8.09E-05	0.985505825	9.16E-07
EFNA1	1.666268401	1.18E-06	1.980098845	7.41E-09	2.805211324	2.41E-16
MTERF	-0.604735974	1.24E-06	-0.46598041	0.0001462	-0.652273198	1.53E-07
MAGOHB	-0.617957651	1.30E-06	-0.16682776	0.1800623	-0.186932183	0.135328
ACKR3	1.027456081	1.35E-06	0.98339255	3.69E-06	1.256001199	3.22E-09
HELB	0.687852864	1.52E-06	0.657380299	3.86E-06	0.917023829	1.16E-10
SYS1	0.596501017	1.54E-06	0.399326183	0.0012772	0.698886429	1.56E-08
PI3	4.225509644	1.56E-06	4.92734322	1.66E-08	4.910768884	1.90E-08
IMP3	-0.551758308	1.61E-06	-0.347898364	0.0021128	-0.338127603	0.002873
SELE	6.222952591	1.62E-06	6.505165397	5.31E-07	8.076180689	4.62E-10
RWDD2A	-0.752703538	1.70E-06	-0.516446422	0.0006389	-0.409479014	0.006746
PML	0.84108132	1.72E-06	0.558448457	0.001501	0.579641376	0.000988
GADD45G	1.809134361	1.78E-06	0.865684398	0.0263072	1.73967632	4.23E-06
CCDC38	-2.602234649	1.83E-06	-0.206586748	0.6652521	-0.457936907	0.349823
SLC31A2	1.136162338	1.84E-06	0.895189926	0.0001706	1.062417576	7.25E-06
BCL2L15	1.916282768	1.87E-06	1.068310497	0.0079285	2.413835456	1.84E-09
ZNF189	0.564542189	1.99E-06	0.431067421	0.0002769	0.565487408	1.82E-06
ZBTB14	-2.188054804	2.01E-06	-0.480389719	0.2258254	-0.708076983	0.075827
UGCG	0.90210073	2.23E-06	0.757728288	7.03E-05	0.952002152	5.85E-07
GRINA	0.620048177	2.43E-06	0.224566758	0.0884707	0.092940112	0.481737
NCR3LG1	0.822014913	2.47E-06	0.881895746	4.05E-07	0.972941674	2.23E-08
OSGEP	-0.469300411	2.51E-06	-0.034210583	0.7242071	-0.166363966	0.08975
ZBTB24	-0.624317323	2.62E-06	-0.518976615	7.87E-05	-0.780789202	4.29E-09
SLC12A7	1.34598807	2.78E-06	0.796342953	0.0056333	1.145861185	6.72E-05
ZNF550	-0.661046212	2.89E-06	-0.438583991	0.0013971	-0.510199787	0.000239
NXT2	0.534354501	2.97E-06	0.531790026	2.58E-06	0.720076081	1.70E-10
NUDT21	-0.308728993	3.00E-06	-0.151226616	0.0212418	-0.172313467	0.008921
LIMK2	-0.53530805	3.25E-06	-0.393477167	0.0005865	-0.089047529	0.435051
OPTN	0.748474565	3.28E-06	0.517427094	0.0012944	0.542604433	0.000743
ARHGEF2	0.58200651	3.30E-06	0.477353742	0.0001331	0.50728621	4.97E-05
SLC25A38	-0.506590098	3.46E-06	-0.502973042	3.10E-06	-0.586552124	7.06E-08
FTSJ2	-0.581007299	3.57E-06	-0.630561619	3.78E-07	-0.675110901	6.67E-08
PARP8	0.591247803	3.69E-06	0.622883999	9.40E-07	0.659203894	2.22E-07
KDM6B	1.087005472	3.72E-06	0.610214713	0.0097138	0.635874165	0.006852
LRIG3	0.5319574	3.81E-06	0.128754975	0.2636178	0.351625921	0.002255
RTKN	0.533550977	3.82E-06	0.340952258	0.0031416	0.344504114	0.002885
VPS37B	0.621126937	3.89E-06	0.269773282	0.0453546	0.61785322	4.29E-06
SH3BP2	0.770032226	3.96E-06	0.468715728	0.0049952	0.609364496	0.000262
SOX2	-1.454922717	4.03E-06	-1.737682038	3.73E-08	-1.227196716	9.52E-05
RAD17P1	-1.770969971	4.08E-06	-1.645630934	6.63E-06	-0.845767598	0.012219
DSE	0.98080748	4.18E-06	0.877764913	3.77E-05	1.201955329	1.63E-08
ZNF398	-0.536053712	4.20E-06	-0.435773907	0.0001622	-0.588243134	4.45E-07
TTC13	-0.781162557	4.46E-06	-0.295750235	0.0790029	-0.344928391	0.040362
USP25	-0.562098196	4.54E-06	-0.214207154	0.0788997	0.06351728	0.601508
ZNF197	-0.398591398	4.65E-06	-0.209866222	0.0113534	-0.31783379	0.000186
CSNK1E	0.399673535	4.69E-06	0.343346148	8.03E-05	0.295489724	0.000697
P11-745A24	1.53556437	4.70E-06	1.090409207	0.0013037	1.718951323	1.99E-07

CIPC	-0.425080276	4.81E-06	-0.543307205	4.42E-09	-0.574346558	6.73E-10
TNIP3	1.976863968	5.03E-06	1.946684988	6.01E-06	2.357891385	4.26E-08
TFE3	0.656237073	5.06E-06	0.493285612	0.0006113	0.773544441	6.98E-08
PLK3	0.949667402	5.10E-06	0.678534125	0.0011277	1.075838366	2.23E-07
FRMD4B	0.830378789	5.29E-06	0.322839286	0.077811	0.788445242	1.50E-05
VANGL1	-0.637491104	5.28E-06	-0.52598539	0.0001609	-0.413946791	0.002896
FARP2	0.55512736	5.35E-06	0.237190505	0.0518624	0.278457318	0.022384
TNFRSF10D	0.852242812	5.37E-06	0.702125843	0.0001773	0.984563909	1.44E-07
CRIM1	0.620168563	5.41E-06	0.455979632	0.0008125	0.260759573	0.056292
HMGCS1	0.8511569	5.77E-06	0.698806074	0.0001968	1.146951332	9.90E-10
N4BP3	1.123771154	5.76E-06	0.957880997	0.0001068	1.828515904	4.79E-14
PTGS2	2.647852273	6.04E-06	1.834978401	0.0017174	2.946723485	4.74E-07
TMCC3	1.065224814	6.03E-06	0.46799374	0.0475162	1.494676322	1.64E-10
VNN1	1.666100907	6.03E-06	1.784834396	1.02E-06	1.617919591	1.06E-05
BIRC2	0.614889607	6.26E-06	0.643588761	2.18E-06	0.772131806	1.32E-08
RIPK4	0.813900284	6.43E-06	0.876625801	1.03E-06	1.219817572	8.82E-12
ARID5A	1.028481903	6.52E-06	0.552488636	0.0161417	1.241782012	4.11E-08
GCH1	2.04218145	6.57E-06	1.513593519	0.0008423	2.273249361	5.16E-07
KIAA1586	-0.596341126	6.59E-06	-0.165493272	0.2007274	-0.442418844	0.000763
ZNF92	-0.501146476	6.60E-06	-0.42962164	9.66E-05	-0.499033025	6.46E-06
PDP1	0.678742114	7.16E-06	0.682044753	6.26E-06	0.837388263	2.90E-08
N4BP1	0.435981067	8.61E-06	0.221526248	0.0239543	0.598815422	8.49E-10
CA5B	0.991008147	8.75E-06	0.823614351	0.0002228	1.400870155	3.08E-10
TBCA	-0.445822351	8.75E-06	-0.352217756	0.0003856	-0.135518448	0.171478
CCDC85A	1.820847961	8.80E-06	1.795624625	1.10E-05	1.786675564	1.25E-05
EHD4	0.494230224	8.89E-06	0.335215849	0.0025184	0.313182547	0.004837
ZNF232	-0.617858243	9.01E-06	-0.480048829	0.0004388	-0.197921525	0.14763
KATNBL1	0.758819226	9.41E-06	0.445288449	0.009351	0.698148428	4.57E-05
ZKSCAN4	-0.719796813	9.59E-06	-0.568908573	0.0002552	-0.940982255	1.04E-08
CASP8AP2	-0.428794543	9.71E-06	-0.376661215	8.79E-05	-0.470378185	1.10E-06
RAB20	-1.02066793	1.02E-05	-0.999920646	1.13E-05	-0.546102216	0.014644
TP53INP2	1.161403369	1.04E-05	0.785196235	0.0029975	0.64267921	0.015497
SESN1	-0.882667731	1.05E-05	-1.017061435	3.55E-07	-1.108956187	3.07E-08
ACOX1	0.523716598	1.05E-05	0.266748421	0.0247318	0.562741053	2.14E-06
CLCF1	0.889938038	1.06E-05	0.270592398	0.1914816	0.629876719	0.001865
C9orf69	-0.512830808	1.06E-05	-0.509666026	9.61E-06	-0.396129986	0.000575
CPEB4	0.716336992	1.08E-05	0.264971666	0.1042853	0.752806753	3.65E-06
KDELC1P1	1.411831082	1.08E-05	1.336715168	2.81E-05	1.730619793	4.58E-08
IRF6	0.770042641	1.08E-05	0.313039524	0.0738724	0.713726625	4.46E-05
CDC42EP1	0.859195127	1.10E-05	0.539512869	0.0057911	0.924115381	2.23E-06
FOXO1	0.865587164	1.11E-05	0.787968399	6.21E-05	0.939075565	1.80E-06
MBLAC2	-0.709292198	1.12E-05	-0.339066785	0.0293328	-0.428107503	0.00646
ZFP36L1	0.603325364	1.12E-05	0.321962583	0.0191086	0.441692788	0.001303
DDI2	-0.551249168	1.14E-05	-0.415798085	0.0008698	-0.465828913	0.000197
OAF	0.59184808	1.16E-05	0.720348169	7.61E-08	0.589949739	1.18E-05
EDN1	1.151218842	1.19E-05	0.643417572	0.0147015	1.46332597	2.24E-08
MED15	0.706769956	1.33E-05	0.560934759	0.0005469	0.555992663	0.000612

DNAAF2	-0.612653206	1.33E-05	-0.424880764	0.0018722	-0.474508962	0.000576
RPL15P3	11.39284259	1.34E-05	0.100019845	0.9715913	7.130397975	0.006522
FZD7	0.704339369	1.40E-05	0.500388734	0.002027	0.565760701	0.000485
HIF1A	0.59725814	1.40E-05	0.86389935	3.25E-10	0.884124811	1.24E-10
RNF168	-0.42808997	1.40E-05	-0.596948291	1.23E-09	-0.34885524	0.000382
TRAF1	1.535126745	1.41E-05	1.526412383	1.46E-05	2.023773438	7.76E-09
NEK2	-0.884384468	1.44E-05	-0.275712081	0.1683666	-0.394763333	0.049263
ACAA1	-0.592583019	1.48E-05	-0.378297449	0.0053504	-0.340283265	0.012355
PSMD4	-0.412601112	1.50E-05	-0.210193343	0.0265401	-0.270181599	0.0044
PPP1R3C	-1.610453779	1.53E-05	-1.364526872	0.0002184	-1.301182802	0.000426
ALPK3	0.7902101	1.54E-05	0.207029813	0.25849	0.373196854	0.041455
RFFL	0.53103616	1.56E-05	0.192484661	0.1177499	0.58199364	2.09E-06
GPATCH11	-0.501478929	1.58E-05	-0.362996312	0.0014487	-0.311948116	0.006373
JAK1	0.506751353	1.64E-05	0.388770083	0.0009416	0.616046841	1.58E-07
JUNB	1.46027644	1.66E-05	1.485901577	1.16E-05	2.07570429	8.87E-10
PNRC1	0.565340627	1.66E-05	0.428669158	0.0010882	0.707520464	6.85E-08
ABCF1	0.462975225	1.67E-05	0.179575128	0.0951255	0.380202788	0.000406
PPCS	-0.509123356	1.69E-05	-0.343922859	0.0034037	-0.235297204	0.044691
VRK2	0.503091807	1.72E-05	0.32880892	0.0048521	0.686580362	3.19E-09
UBE2Q2	-0.556608911	1.90E-05	-0.08806811	0.4937567	-0.306902333	0.017747
TATDN2	0.674082072	1.98E-05	0.586666277	0.0002018	0.570330814	0.000295
TGDS	-0.549169193	1.98E-05	-0.385938753	0.0021833	-0.214707208	0.087482
TRIM68	-0.621528313	2.05E-05	-0.412811697	0.0040617	-0.828615496	1.74E-08
ZSCAN12	-0.479618026	2.07E-05	-0.275122951	0.0124779	-0.352379543	0.001511
RIMBP3C	-0.658281871	2.07E-05	-0.402567864	0.0092187	-0.190644162	0.217517
SASS6	-0.736965651	2.13E-05	-0.467368158	0.0063772	-0.485010315	0.00474
IRAK3	1.874468484	2.23E-05	1.860964779	2.48E-05	2.963384804	1.52E-11
PIK3R1	-0.616279207	2.25E-05	-0.416766056	0.0040761	-0.629945862	1.45E-05
ZMYM5	-0.49427064	2.26E-05	-0.364236488	0.0014814	-0.254021346	0.027036
ZNF480	-0.562421743	2.28E-05	-0.357706319	0.0066738	-0.577515787	1.27E-05
KIF18A	-0.569361548	2.34E-05	-0.128064663	0.3336617	-0.438513719	0.001041
SRP9P1	9.110520817	2.38E-05	7.854429033	0.0002723	8.034190088	0.000196
CTNNB1	0.402736685	2.39E-05	0.379988809	6.68E-05	0.291094436	0.002261
GATSL3	1.227875218	2.40E-05	-0.245504794	0.4100734	0.675489189	0.020391
EBI3	1.876634636	2.43E-05	1.650283279	0.0002165	1.651816884	0.000216
ZNF107	-0.726851504	2.43E-05	-0.409311812	0.0153271	-0.35029383	0.037757
TBC1D24	-0.632373537	2.47E-05	-0.456465358	0.0020137	-0.462344706	0.001891
ASF1A	-0.470342602	2.49E-05	-0.308875504	0.0047965	-0.585739162	1.54E-07
CCDC8	-0.587423376	2.52E-05	-0.540026009	9.91E-05	-0.624994204	7.08E-06
ANKK1	2.924847325	2.60E-05	2.405692567	0.0005513	2.698065081	9.86E-05
DKK1	-1.890515494	2.61E-05	-0.930089243	0.0358382	-1.300938528	0.003439
STK40	0.49249537	2.61E-05	0.277930049	0.0176356	0.413134106	0.000413
ZNF57	-1.087387596	2.61E-05	-0.94520253	0.0001973	-1.001890133	9.20E-05
ZNF730	-0.631446801	2.61E-05	-0.094001273	0.5135779	-0.48206762	0.001134
CYP27C1	1.130368129	2.65E-05	0.857404332	0.0014506	0.9650628	0.000338
MSANTD4	-0.560747101	2.65E-05	-0.364448572	0.0056582	-0.71552515	7.80E-08
SBNO2	1.215267519	2.73E-05	1.380396156	1.83E-06	1.285503885	8.83E-06

AP4B1	0.832482343	2.78E-05	0.807013336	4.69E-05	1.209631699	8.75E-10
ZFP36L2	-0.744388441	2.82E-05	-0.670118213	0.0001557	-0.9728231	4.56E-08
RAPGEF3	1.287677369	2.85E-05	0.118928219	0.7050632	0.981413601	0.001491
FBXO45	-0.380592387	2.87E-05	-0.232046938	0.0093841	-0.183995617	0.039882
CSF2	5.056552137	3.06E-05	5.859198776	1.21E-06	6.623712364	3.86E-08
KIAA1217	0.762639118	3.05E-05	0.234039206	0.2010952	0.706299746	0.000113
B3GNT1	-0.55316625	3.16E-05	-0.434304646	0.0008059	-0.781972628	5.18E-09
FAIM3	1.965773595	3.26E-05	1.435101524	0.0027463	2.097613536	8.11E-06
VCAM1	2.587062563	3.29E-05	2.70994123	1.36E-05	2.819205846	6.01E-06
GLRX2	-0.70547441	3.31E-05	-0.34471178	0.0316959	-0.257375084	0.109505
ARIH2	0.345953887	3.33E-05	0.301265101	0.0002825	0.395759142	1.84E-06
ZNF224	-0.466171229	3.34E-05	-0.179133501	0.1048965	-0.496031128	9.94E-06
RRP1	0.593085006	3.38E-05	0.412939712	0.0038475	0.835518045	4.40E-09
SNRPFP1	5.697105126	3.40E-05	6.346452201	3.62E-06	5.955628464	1.42E-05
UQCR11	0.480197555	3.40E-05	0.14274857	0.2179712	0.313649778	0.006745
PGBD2	-0.745057192	3.42E-05	-0.453340457	0.0095414	-0.633543074	0.000354
LIAS	-0.700103365	3.46E-05	-0.374090058	0.0222287	-0.702500804	3.18E-05
PRR15L	-1.508576013	3.57E-05	-1.093314984	0.0026568	-0.901409647	0.013202
MAPKAPK2	0.30644473	3.74E-05	0.219731353	0.0029909	0.257261651	0.000515
TP53	0.984178985	3.81E-05	1.03811617	1.35E-05	1.146638687	1.54E-06
ZNF816	-0.801447004	3.87E-05	-1.052447604	7.63E-08	-1.031085548	1.46E-07
C12orf29	0.486968615	3.89E-05	0.400531185	0.0006851	0.650694422	3.28E-08
MRPS31P4	-1.081802688	3.91E-05	-0.10927649	0.6472265	-0.295201753	0.224402
CTB-50E14.!	0.962822953	3.92E-05	0.470650688	0.0468303	0.880524979	0.000164
EGR3	2.168300097	3.94E-05	1.395103084	0.0084507	1.828798487	0.000531
GATA6	-0.87932755	3.98E-05	-0.995731462	3.21E-06	-1.047753976	9.75E-07
ZNF606	-0.618694356	4.01E-05	-0.289480958	0.0497188	-0.611123907	4.49E-05
SAP30L	-0.799226008	4.02E-05	-0.194335246	0.3149689	-0.293274902	0.130489
AC024592.1	5.265116192	4.14E-05	6.117733319	1.87E-06	3.887745375	0.002541
DEDD2	0.711302868	4.15E-05	0.596688953	0.000562	0.971873897	1.62E-08
FGFR3	-0.697439913	4.14E-05	-0.701738703	3.57E-05	-0.770605286	5.77E-06
SERTAD2	0.707682688	4.14E-05	0.244361386	0.1580531	0.760458229	1.00E-05
SKIV2L2	-0.327708072	4.14E-05	-0.113916861	0.1509436	-0.138347535	0.081818
GPR132	1.804925313	4.18E-05	1.939136287	7.90E-06	2.19541653	4.27E-07
CREB3	0.410849628	4.25E-05	0.231797292	0.0205958	0.329865597	0.000991
CD55	1.236832612	4.45E-05	1.115977259	0.0002295	1.66332132	3.92E-08
ATP5H	-0.31497533	4.56E-05	-0.256098139	0.0008304	-0.124996774	0.102386
FAM213B	0.50204248	4.56E-05	0.167041406	0.1770279	0.365393975	0.003016
LINC00888	-0.542504184	4.55E-05	-0.122988771	0.3327471	-0.244091438	0.060313
RHOG	0.456100227	4.63E-05	-0.181175465	0.1148742	0.046882172	0.680492
ZNF433	-0.735713016	4.84E-05	-0.470981048	0.0069957	-0.646129926	0.000257
COX11	-0.373076639	4.93E-05	-0.263763577	0.0036049	-0.314038284	0.000569
NADK2	-0.40197961	5.02E-05	-0.072014484	0.4590943	-0.126352235	0.196733
DAPP1	2.297192573	5.04E-05	2.748188051	1.09E-06	3.237940999	8.80E-09
SNAPC3	-0.434082031	5.20E-05	-0.13983571	0.1870003	-0.185664043	0.081561
ZSWIM3	-0.704897256	5.23E-05	-0.670668982	6.87E-05	-0.458398494	0.005844
FSTL3	0.97137198	5.36E-05	0.362582982	0.1331934	0.851234791	0.000399

ZNF605	-0.527998835	5.45E-05	-0.471516556	0.0002778	-0.479878203	0.000234
RBAK	-0.350048495	5.59E-05	-0.216190893	0.0115121	-0.295690445	0.000596
TRIOBP	0.358703023	5.61E-05	0.173323985	0.051277	0.344953762	0.000103
FAM83A	-0.640364782	5.68E-05	0.841400195	1.12E-07	0.655058331	3.63E-05
TMEM106A	2.108475374	5.68E-05	1.171157739	0.0275339	2.136560179	4.06E-05
TC-479C5.1	1.062459859	5.77E-05	0.313624687	0.2381162	0.256018701	0.335953
ZNF440	-0.509605365	5.82E-05	-0.480119624	0.0001372	-0.617520909	1.02E-06
DOLPP1	-0.43552029	5.84E-05	-0.375515106	0.0004306	-0.494050088	4.58E-06
UBE2V2	-0.345308722	5.87E-05	-0.154488393	0.0681051	-0.206059405	0.015395
AFF4	0.532636983	5.90E-05	0.232402602	0.0796104	0.441428529	0.000868
CLIC4	0.531626251	5.89E-05	0.554373254	2.77E-05	0.680426912	2.67E-07
TNIP2	0.551417645	6.05E-05	0.354789744	0.0098611	0.498669152	0.000264
MNX1	-1.416890555	6.08E-05	-1.569947016	6.24E-06	-1.6508792	3.14E-06
SORD	0.819886207	6.25E-05	0.424757196	0.0380894	1.009339903	8.09E-07
GGCT	-0.470512137	6.44E-05	-0.2825459	0.0146524	-0.036878345	0.748636
TNFRSF11E	1.442031464	6.52E-05	2.023161359	1.97E-08	2.027764364	1.84E-08
AC112218.1	0.413503314	6.58E-05	0.109613427	0.2909002	0.262539173	0.011297
SREK1IP1	0.651382672	6.59E-05	0.406096397	0.012556	0.45406583	0.005297
BCL11B	-1.214201559	6.74E-05	-0.764411791	0.0103977	-1.295473835	2.03E-05
LYSMD4	-0.716939894	6.73E-05	-0.634054197	0.000364	-0.716453891	6.20E-05
FAM171A1	0.452524944	6.80E-05	0.277126874	0.0145006	0.414836593	0.00025
STAG3L4	0.640329354	6.83E-05	0.326683724	0.0384699	0.272865651	0.088334
C2ORF15	-0.944521221	7.06E-05	-0.819568722	0.0004497	-0.353765854	0.123969
RPA4	1.775722151	7.09E-05	2.001486296	4.90E-06	1.827884018	3.68E-05
FAM217B	-0.797189745	7.12E-05	-1.050592273	1.34E-07	-1.060485922	1.29E-07
SPRR1B	3.244276858	7.15E-05	2.275544953	0.0060167	4.06063084	5.08E-07
PCNXL3	0.443237959	7.19E-05	-0.030863834	0.7830261	0.115891021	0.301278
CTBP2	0.337236058	7.24E-05	0.209262061	0.0135766	0.276952878	0.001087
BCL6	1.061517768	7.32E-05	0.587853663	0.0282112	0.998644789	0.000188
FAM57A	0.471343491	7.36E-05	0.310681223	0.0089007	0.494903545	2.92E-05
EBLN2	0.711195156	7.40E-05	0.661458116	0.0001976	0.810490484	4.83E-06
PTAFR	1.406392449	7.44E-05	0.720076749	0.0429409	1.169560813	0.000991
ZMYND15	1.745401216	7.47E-05	1.53309509	0.0004893	1.921656424	1.07E-05
ATP1B1	0.669426678	7.65E-05	0.657965471	0.000101	0.507896256	0.002693
CYP2C18	1.114048509	7.71E-05	0.810651749	0.0040165	1.912778197	9.37E-12
TSEN54	-0.444833809	7.84E-05	-0.355427015	0.0014542	-0.287482756	0.009479
ZNF761	-0.531116384	7.84E-05	-0.6523526	1.14E-06	-0.511899407	0.000132
EEF1A1P5	0.563303042	7.87E-05	0.268520231	0.0598163	0.273485223	0.05525
NUDT9	-0.542037945	7.91E-05	-0.360923735	0.0077423	-0.430420771	0.001611
SNPH	2.041931067	7.95E-05	1.04491673	0.0447175	1.682327044	0.001169
RAB1A	0.345065418	8.11E-05	-0.020987865	0.8106545	0.191318687	0.028808
ZNF703	-0.70193854	8.37E-05	-1.251947796	5.90E-12	-1.462329943	3.88E-15
OAS1	0.708941885	8.40E-05	0.110267968	0.5413704	0.390881531	0.030257
TSPAN15	0.670245306	8.42E-05	0.278796475	0.1024991	1.117933665	4.76E-11
ALG10B	-0.409767467	8.71E-05	-0.270551106	0.0088173	-0.176243301	0.088699
MACROD2	0.613885123	8.71E-05	-0.04565888	0.7736992	-0.086532176	0.58806
RTF1	0.352676292	8.75E-05	0.027976865	0.7548308	-0.017124156	0.848678

PAPD5	-0.451625305	8.79E-05	-0.285875887	0.0118081	-0.465491538	4.78E-05
PANK1	-0.518850383	8.86E-05	-0.435166854	0.0008978	-0.52329918	7.00E-05
DNAJA3	-0.53640172	9.07E-05	-0.167406396	0.2161501	-0.381035679	0.005213
PFKFB3	0.852932802	9.23E-05	1.401641147	1.23E-10	1.999223851	4.05E-20
AMOTL2	0.846957573	9.25E-05	0.374446164	0.0844629	0.658496217	0.00238
SLC5A2	1.888006477	9.30E-05	2.17873274	5.68E-06	2.361635392	8.97E-07
CEP76	-0.601195167	9.35E-05	-0.160400084	0.2865386	-0.352930251	0.020836
LACC1	0.697153893	9.66E-05	0.572579784	0.0013328	0.776964112	1.23E-05
SF3B2	-0.573621796	9.69E-05	-0.254796003	0.0792412	-0.502649669	0.00059
ZNF112	-0.496746611	9.72E-05	-0.312686357	0.0114071	-0.601403993	2.00E-06
MDM1	-0.880375097	9.99E-05	-0.414495491	0.0639198	-0.738532981	0.000995
GEMIN6	-0.532027076	0.0001002	-0.280743109	0.0348391	-0.381031766	0.004759
BAIAP2L1	0.515354831	0.0001025	0.364857428	0.0059756	0.600248409	5.90E-06
CKAP4	0.31780308	0.0001026	0.155878273	0.0564874	0.282978703	0.000533
LUZP6	1.319060171	0.0001025	0.647612307	0.0604693	0.565637645	0.103762
CSTF1	-0.388058719	0.0001055	-0.345497018	0.0005129	-0.364237077	0.000267
ST5	0.614007116	0.0001053	0.465830352	0.0032141	0.407182598	0.010123
TMEM165	0.456402148	0.0001057	0.354797772	0.0025406	0.654304954	2.41E-08
ADAM8	1.111723537	0.0001093	0.414937125	0.1508081	1.064924057	0.000211
PHIP	-0.300449356	0.0001136	-0.285809792	0.0002301	-0.272521221	0.000451
CTSS	0.918853593	0.0001154	0.592660545	0.0129208	1.488114571	3.60E-10
PDE4B	1.595482424	0.000118	1.26318185	0.0023408	1.232827384	0.002929
MEF2D	0.678690247	0.0001182	0.067686112	0.7020675	0.252581695	0.154979
PTP4A3	1.225997586	0.0001213	1.155448279	0.0002891	0.914455189	0.004231
MEGF9	-0.384799474	0.0001223	-0.16501374	0.0903101	-0.278791117	0.00469
FEM1C	0.542146839	0.0001246	0.300738831	0.0333125	0.632981621	6.99E-06
FAM46C	-1.295878508	0.0001251	-1.992582552	4.19E-09	-1.554873341	4.25E-06
MYO1C	0.412176743	0.0001254	0.115931764	0.2802674	0.25681284	0.016736
CENPE	-0.861174708	0.0001285	-0.281429147	0.2098341	-0.559892268	0.012691
GNPTAB	-0.388767628	0.0001302	-0.047666146	0.6361062	0.05364504	0.595024
VPS29	-0.340995468	0.0001302	0.036547975	0.6763896	0.107240997	0.220598
RELT	0.949527936	0.0001309	0.657316495	0.0079959	0.772397945	0.001726
TRIT1	-0.550193066	0.0001311	-0.328734044	0.0214084	-0.371375816	0.009457
P11-39K24.	1.908327678	0.0001326	1.779738656	0.0003446	2.603792204	8.69E-08
RNF207	0.6849542	0.0001333	0.78061288	1.21E-05	0.66608066	0.0002
SRP9	-0.230184	0.0001335	-0.019628365	0.7431301	-0.011942048	0.842127
ZNF225	-0.577774243	0.0001344	-0.364432322	0.0132886	-0.38321541	0.010056
GPR108	0.519443693	0.0001358	0.497066793	0.0002573	0.603296642	9.09E-06
HS3ST3B1	0.894041924	0.0001392	0.693532484	0.003097	0.646608139	0.006025
WIZ	0.473586733	0.0001409	0.068038695	0.5860796	0.007688654	0.951125
MAPK14	-0.378873259	0.0001411	-0.579136543	5.76E-09	-0.380901164	0.000126
FADD	-0.381180574	0.0001458	-0.351340113	0.0003579	-0.428028117	1.75E-05
UPF2	-0.356893074	0.0001473	-0.267211808	0.0042262	-0.271636489	0.003698
ATP10D	0.673223691	0.0001482	0.123347222	0.4870897	0.433966234	0.014524
CMPK2	-0.899042586	0.0001484	-0.415423338	0.063234	-0.298781333	0.185542
ZNF37A	-0.471154271	0.000148	-0.087376859	0.4729362	-0.540511234	1.22E-05
B3GNT2	0.501352991	0.0001526	0.372917814	0.0047769	0.743871112	1.64E-08

CACYBP	-0.559739936	0.0001535	-0.112938642	0.4394741	-0.011394086	0.937926
TNKS1BP1	0.613162014	0.0001555	0.368404981	0.0230777	0.678806145	2.79E-05
CAPN7	-0.532103215	0.0001561	-0.090099654	0.520119	-0.215410008	0.124356
H3F3B	0.384027485	0.000156	0.181543643	0.0736727	0.302226519	0.002906
CENPC	-0.368061998	0.0001569	-0.235587075	0.013871	-0.31381673	0.001143
RNF44	0.560881345	0.0001576	-0.14301889	0.3409135	-0.204366903	0.175173
TAP1	0.815084849	0.0001599	0.614386915	0.0044168	0.864019719	5.94E-05
GOPC	-0.29785012	0.0001602	-0.246713596	0.0015822	-0.196832509	0.011859
RUNX1	0.641488836	0.0001605	0.201613091	0.2367342	0.668651089	8.20E-05
ZNF514	-0.500241959	0.0001619	-0.173277583	0.1810647	-0.244365586	0.061423
GPR107	0.410526734	0.0001633	-0.060194479	0.5816194	0.140486845	0.197662
KLF3	0.38139209	0.000164	0.002519963	0.9801477	0.249766146	0.013565
TD-2008A1	1.020233411	0.000166	0.539522789	0.0468507	1.294435368	1.65E-06
WDR11	0.376743504	0.0001704	0.407811261	4.60E-05	0.533344569	9.58E-08
FEZ1	1.05811239	0.0001709	0.645466231	0.0218743	0.897163291	0.001456
INO80C	0.633356616	0.0001713	0.770940484	4.19E-06	0.872176441	1.92E-07
TTC30B	-0.64820183	0.0001714	-0.81910583	1.83E-06	-0.761950432	9.63E-06
SPRY4	0.543194838	0.0001826	0.366130662	0.0116396	0.254504248	0.079775
RASA1	-0.321648721	0.0001874	-0.239199127	0.0052862	-0.177919264	0.037894
TRAPPC1	0.383290646	0.0001872	0.005743937	0.9557089	0.056485645	0.586113
BLNK	0.88643212	0.0001892	0.485383332	0.0412948	0.978920245	3.63E-05
STAP2	0.59642359	0.0001924	0.261000324	0.1029782	0.671195831	2.58E-05
CTCF	-0.266606147	0.000194	-0.298156386	2.77E-05	-0.348187695	1.09E-06
LEO1	-0.333764623	0.0001943	-0.222561082	0.0115125	-0.341723019	0.000122
SLC30A7	0.379054257	0.0001939	0.234646573	0.020772	0.418370064	3.73E-05
FOSL2	0.842266111	0.000195	0.386859574	0.0872031	0.888468998	8.47E-05
DBT	-0.471571249	0.0001972	-0.269336544	0.033305	-0.388068633	0.002232
MAST4	0.748321567	0.0002066	0.371383956	0.0660822	1.102156099	4.38E-08
COTL1	0.749442021	0.0002085	0.533569536	0.0082731	0.647201201	0.001359
PEF1	0.369382156	0.0002086	0.055431693	0.5782768	0.191057224	0.055024
SLC20A1	0.496696682	0.0002115	0.375695403	0.0050454	0.370076587	0.005765
ZNF138	-0.547994019	0.0002114	-0.217893281	0.133305	-0.299769886	0.040389
KIAA0391	0.465380227	0.0002172	0.417015915	0.0008614	0.507900182	5.01E-05
MLX	-0.307837076	0.0002173	-0.254790982	0.0019323	-0.257944625	0.001751
ANKRD36B	0.675897587	0.0002186	0.679750036	0.0001977	1.028380383	1.59E-08
FAM136A	-0.46419771	0.0002189	-0.285487624	0.0219794	-0.175361764	0.159432
CCSER2	0.420199074	0.0002225	0.384972635	0.0007044	0.516073935	5.47E-06
SNX30	-0.684247957	0.0002231	-0.158419582	0.3914673	-0.503434013	0.006614
PDE8A	0.513147817	0.0002244	0.441307919	0.0014938	0.627331962	6.13E-06
EGR2	1.309462517	0.0002261	0.941343956	0.0080907	1.136132281	0.00138
PEX1	-0.477754347	0.0002265	-0.314898442	0.0137332	-0.254516803	0.047383
PISD	0.484095358	0.0002286	0.20391571	0.120664	0.494802879	0.00016
HARS2	-0.357988083	0.0002304	-0.203898009	0.0319585	-0.261608675	0.006319
CRNKL1	-0.440238808	0.000231	-0.341705415	0.0040347	-0.366393326	0.00211
ZNF555	-0.576257352	0.0002311	-0.486233551	0.001554	-0.477455395	0.002153
LYPD6B	0.845254952	0.0002326	0.635562118	0.0056033	1.712859273	3.37E-14
UBL4A	-0.501114413	0.0002325	-0.472951195	0.0004017	-0.26518337	0.047104

SLFN11	1.433658632	0.0002341	2.939300377	4.18E-14	2.516312474	1.01E-10
PVRL2	0.433660409	0.0002355	0.262283982	0.026075	0.409542875	0.000505
PRKD3	0.509802861	0.0002371	0.570638634	3.79E-05	0.769509771	2.70E-08
BTN2A1	0.425449022	0.0002378	0.015358882	0.8946087	0.375550678	0.001127
MAP10	-0.69679068	0.00024	-0.542475222	0.0029938	-0.833701353	1.11E-05
SSX2IP	-0.42648532	0.0002421	0.019002866	0.8682843	-0.4302863	0.000199
POLG2	-0.650296541	0.0002445	-0.323682983	0.0541599	-0.389132136	0.02303
ESRP2	-0.461547714	0.0002473	-0.675167106	7.91E-08	-0.425854141	0.000691
XPO1	-0.324701522	0.0002484	0.006345099	0.9428092	-0.179796816	0.042252
ZNF813	-0.673895082	0.0002513	-0.555833254	0.0023612	-0.734090292	6.21E-05
RASGEF1B	0.54072922	0.000256	0.241771139	0.1048534	0.712635908	1.32E-06
RTKN2	-0.626226332	0.0002567	-0.229326013	0.1742283	-0.28751874	0.088755
CAMK2G	-0.666405802	0.000257	-0.441045532	0.0150194	-0.617443807	0.000676
ANKRD42	-0.556102159	0.0002574	-0.369692854	0.0143663	-0.350642567	0.020419
P11-680E19	-1.984623833	0.000262	-1.271201973	0.0108165	-2.148548737	8.18E-05
ZNF593	0.666101988	0.0002648	0.413504819	0.0236058	0.617331096	0.000699
DCTN1	0.533202747	0.0002665	0.208270587	0.154414	0.395982996	0.006778
AP006216.1	-1.398019052	0.0002701	-1.110813998	0.0034271	-1.233485083	0.001211
FAM203A	-0.560117706	0.0002746	-0.430098208	0.0044302	-0.497825096	0.001093
HIC2	-0.475350098	0.0002817	-0.824323374	3.38E-10	-0.786385998	2.19E-09
LRRC37A6F	-1.369028714	0.0002895	0.007601556	0.9816081	-0.054756525	0.87297
FAM185A	-0.668980736	0.00029	-0.278617596	0.1232276	-0.716333688	7.72E-05
SOX21	-1.301640444	0.0002903	-1.803213266	5.63E-07	-1.325349868	0.00022
CCT8P1	-0.97741627	0.0002931	-0.463119288	0.0650165	-0.526531403	0.038469
AC005280.1	-0.4068017	0.0002965	-0.436969297	7.81E-05	-0.561488127	6.12E-07
ATP8B5P	1.736453735	0.0003001	0.76572008	0.1196026	1.030372371	0.035655
MGAT4B	0.372503767	0.0003052	0.084733416	0.4118035	0.257064379	0.012661
PRDX1	-0.473202198	0.000306	-0.180593697	0.1676214	-0.075011283	0.566501
ZSWIM6	0.372017328	0.0003065	-0.126714809	0.2223017	0.015829106	0.87866
KIAA0586	-0.463282663	0.0003081	-0.359709525	0.0045085	-0.420906516	0.000948
LAPTM4B	1.304712454	0.0003087	1.124171432	0.0018683	0.879176551	0.015209
ZNF273	-0.498012516	0.0003106	-0.331201238	0.0142168	-0.532649965	0.000113
CROT	-0.828809982	0.0003126	-0.361757942	0.1111366	-0.584713271	0.010655
ZNF486	-0.543359486	0.0003141	-0.414251435	0.0055153	-0.502797577	0.000791
PARS2	-0.498630594	0.0003162	-0.648746705	2.22E-06	-0.609622481	1.00E-05
PLEKHG3	0.484357959	0.0003175	0.308777332	0.0216461	0.602832661	7.09E-06
MAP1LC3B	0.627144777	0.0003198	0.516570429	0.0030047	0.730743856	2.65E-05
SEPHS2	-0.50639728	0.0003195	-0.255447702	0.0659503	-0.115526341	0.405204
ZNF493	-0.480483658	0.0003191	-0.201495193	0.1268987	-0.393036166	0.003151
PPP2R1A	0.308196269	0.0003209	0.059629738	0.4863072	0.069194562	0.419439
GATA5	-1.6908684	0.0003229	-1.225109048	0.0070161	-0.969207893	0.031911
FAM53C	0.449061626	0.0003267	0.128291589	0.3061352	0.163361419	0.192533
PIM3	1.125785633	0.0003287	0.770497875	0.014002	1.371015104	1.20E-05
ZNF845	-0.654714663	0.0003285	-0.569758311	0.0017222	-0.416736975	0.021944
GDF15	1.343379041	0.0003302	0.905999523	0.0154772	1.954681166	1.73E-07
IKBKB	0.563332862	0.0003306	0.547625726	0.0004758	0.428216421	0.006332
ZBTB41	-0.410689033	0.0003362	-0.467923077	4.06E-05	-0.30202421	0.007997

HNRNPA3	0.478743164	0.0003367	-0.182115702	0.1728632	-0.130236994	0.330021
RPUSD2	-0.531148787	0.0003445	-0.35875913	0.0131218	-0.601762498	4.90E-05
DAGLB	0.436999736	0.000347	0.202255569	0.096905	0.327971722	0.00697
ZNF184	-0.404154915	0.0003466	-0.263573481	0.0171458	-0.426354026	0.000143
WDR5B	-0.506642612	0.000349	-0.240984857	0.0761252	-0.301008687	0.028602
CDK19	0.349542922	0.0003507	0.263903245	0.0068462	0.15920342	0.104304
PHC1	0.855395298	0.0003625	0.417611498	0.0819444	0.456916282	0.057034
GPR180	-0.324824806	0.0003638	-0.207824825	0.0208363	-0.24060736	0.007731
CDV3	0.333954852	0.0003654	0.302692217	0.0012282	0.472226884	4.56E-07
CHMP4B	0.281799001	0.000368	0.167481807	0.033561	0.459640829	4.49E-09
PLEKHA7	-0.689332182	0.0003785	-0.786440264	4.91E-05	-0.42017824	0.029748
TANK	0.410173587	0.0003796	0.261071052	0.0233585	0.624985737	5.25E-08
AGL	-0.299479993	0.000383	-0.149243219	0.0739746	-0.134975984	0.106691
IMMP1L	-0.451894102	0.0003833	-0.365760169	0.003063	-0.669035668	1.71E-07
MARS	0.452688889	0.0003826	0.309206613	0.0150317	0.385105956	0.002447
OSMR	0.864458016	0.000383	0.75787929	0.0018414	1.190806989	9.74E-07
PSMD1	1.039881904	0.0003842	0.659133374	0.0240254	0.857791116	0.003316
TNFAIP1	0.476449653	0.000385	0.434975623	0.0011754	0.562254975	2.70E-05
TGIF1	0.431630958	0.0003885	-0.143740435	0.2389811	0.107271583	0.378443
CLDN12	0.463452802	0.0003926	0.25710986	0.0492679	0.604465622	3.56E-06
NDUFV2	0.422497589	0.0003921	0.404780954	0.00066	0.607816938	3.08E-07
MAL2	2.90596238	0.0003932	2.467252461	0.0026366	2.539088779	0.001964
LSM3	-0.417539922	0.0003968	0.053998455	0.6414212	-0.078884304	0.498234
CCDC138	-0.516299018	0.0003975	-0.329380248	0.0214789	-0.410642002	0.004322
RPUSD3	-0.312747714	0.0003995	-0.206350084	0.0159352	-0.30041873	0.000585
N6AMT1	-0.503828924	0.0004047	-0.502996932	0.0003248	-0.579052082	4.59E-05
PTBP3	0.477295492	0.0004137	0.148097459	0.2731017	0.357375654	0.008159
ISCA1	-0.415623507	0.0004154	-0.207557282	0.0745472	-0.353405305	0.002544
PEA15	0.460831423	0.0004149	0.320668594	0.0138375	0.564283101	1.46E-05
ADM	1.277788895	0.0004215	1.498099737	3.42E-05	2.396673946	2.92E-11
RSBN1L	-0.510296113	0.0004264	-0.451262162	0.0017118	-0.374577254	0.00946
CLDN4	0.640657268	0.0004297	-0.011768925	0.9484714	0.647551728	0.000371
WIBG	-0.329277747	0.0004297	-0.391837451	2.36E-05	-0.413086058	9.81E-06
PPRC1	0.468022784	0.0004356	-0.13130218	0.3250806	0.053515639	0.688182
ZNF724P	-0.470712462	0.0004352	-0.278229079	0.0350263	-0.635730125	1.98E-06
P11-761N21	20.27289824	0.0004393	18.99546353	0.0009896	-20.2031057	0.000561
CWF19L2	-0.37090818	0.0004424	-0.22727168	0.0277734	-0.276714133	0.007854
CBX3	-0.241976388	0.0004462	-0.111280733	0.1038845	-0.113362474	0.097917
MAPK1IP1L	0.615472566	0.0004461	0.185958516	0.288711	0.245440583	0.161561
SLC12A2	0.69515615	0.0004453	0.411356331	0.0377101	0.828551578	2.84E-05
ARL5B	0.359672893	0.0004512	0.224959373	0.0281536	0.481944486	2.53E-06
ZNF706	0.356362741	0.0004544	0.268615977	0.0081384	0.671760341	2.58E-11
FASTKD1	-0.505023594	0.0004565	-0.309111803	0.030037	-0.377761293	0.008049
EEF1A1P9	-0.630351324	0.0004616	-0.409327888	0.0181892	-0.111218296	0.515268
ZNF780A	-0.62635093	0.0004621	-0.367095807	0.0384641	-0.414560121	0.020056
CAPZB	0.261506524	0.0004688	0.156311119	0.0358746	0.344507332	3.56E-06
MLYCD	-0.468052033	0.0004774	-0.488651922	0.000219	-0.521920322	9.39E-05

ITGAV	0.611800241	0.0004786	0.465317789	0.0078938	0.768989625	1.13E-05
BTBD19	0.571481142	0.0004794	0.288782382	0.0783622	0.295758194	0.072586
MGAT2	-0.271310236	0.0004818	-0.176396069	0.0214629	-0.108138325	0.159022
ZNF721	-0.452133916	0.0004878	-0.477525681	0.0002143	-0.371654822	0.003976
C10orf10	0.930496655	0.0004955	0.796713801	0.002815	1.038435851	9.84E-05
PDGFB	2.438046833	0.0004949	2.302208961	0.0010066	2.939378112	2.55E-05
RMDN2	-0.626846439	0.0005011	-0.218907248	0.2091741	-0.392670303	0.026655
SAMD4A	0.842349122	0.000501	0.8979332	0.0002051	0.630819862	0.009122
KNOP1	-0.490809737	0.0005018	-0.536662175	0.0001371	-0.604408841	1.79E-05
GCKR	1.147848316	0.0005058	0.486402782	0.1457687	0.912690736	0.005838
DENND3	0.769778927	0.0005081	0.870987141	7.42E-05	0.650717764	0.003126
LARP6	0.826056723	0.0005076	0.345872406	0.1450711	0.956406062	5.25E-05
GNA15	1.572418424	0.0005116	0.837215517	0.0659114	1.926203862	1.93E-05
CNKSR1	0.823260189	0.0005164	0.001375157	0.9954706	0.575255828	0.015897
C9orf40	-0.6835022	0.0005196	-0.104939864	0.5620607	-0.510545889	0.0072
KCTD11	0.761920816	0.0005251	0.805720103	0.0002302	1.27550694	4.73E-09
PPFIBP2	-0.770584521	0.0005293	-0.648799715	0.0034729	-0.395336065	0.074197
ZNF10	-0.667038281	0.0005313	-0.21529846	0.2519393	-0.452363713	0.018017
PLCXD1	0.704953214	0.0005324	-0.084287375	0.6788972	0.015353923	0.939955
PPIE	-0.320268293	0.000539	-0.338707368	0.0001956	-0.31862088	0.00049
HAUS6	-0.400358976	0.0005472	-0.223998785	0.0511825	-0.245252217	0.033147
MRPL27	-0.417591153	0.0005485	-0.290941358	0.0147578	-0.199633063	0.095067
MRPS18C	-0.413627494	0.0005492	-0.11750844	0.3184408	-0.125190866	0.290488
ERI2	-0.375710937	0.0005499	-0.184174705	0.0832322	-0.096203388	0.366202
ETF1	0.271763431	0.0005529	0.360200317	4.35E-06	0.447689074	1.14E-08
TBCE	-0.362247699	0.0005543	-0.123555493	0.227958	-0.145571751	0.157321
EPS8L3	0.624809146	0.0005579	0.138107927	0.4458389	0.544671763	0.002619
EEA1	-0.432677295	0.0005595	0.001380459	0.9911676	-0.011104411	0.929029
FOXJ1	-1.64901596	0.0005658	-2.77108022	9.72E-08	-1.544498643	0.001023
DENND5A	0.573756233	0.0005684	0.464360326	0.0052394	0.370910657	0.025877
AURKA	-0.616561342	0.0005728	-0.339418255	0.0562456	-0.480675945	0.00699
NR3C2	0.965582672	0.0005794	0.039352629	0.88995	0.675787383	0.0164
RFX5	0.40311504	0.0005826	0.221746216	0.0581753	0.380455379	0.001132
G6PC3	0.392632449	0.0005837	0.032636277	0.7760114	0.176105541	0.124975
ARHGEF19	-0.505579429	0.0005864	-0.638763238	1.33E-05	-0.711205746	1.44E-06
SH2D4A	0.679254648	0.0005903	0.364702399	0.0651717	0.967699059	9.46E-07
DTX4	-0.540505682	0.0005955	-0.497027191	0.0015815	-0.408060884	0.009474
GNPDA2	-0.386197625	0.0005997	-0.112528783	0.3084086	-0.134402832	0.228123
TLR3	1.086675541	0.0006022	0.401428246	0.2062284	1.18506129	0.000146
AGFG1	0.412899566	0.0006046	0.332012868	0.0057855	0.496620464	3.64E-05
CDC23	-0.28910263	0.000605	-0.231882043	0.005335	-0.248037652	0.002985
MAP3K11	0.383283414	0.0006097	0.257511918	0.021901	0.328626905	0.003358
PTBP1	0.4409333	0.0006101	0.200040562	0.1197772	0.27674434	0.031425
NET1	0.448933052	0.0006118	0.214840816	0.1010205	0.762235152	5.71E-09
TNFRSF10A	0.786494428	0.0006169	1.030463548	6.93E-06	1.241968817	5.92E-08
ZNF141	-0.356229212	0.0006289	-0.255564371	0.0128165	-0.390848416	0.000163
DUSP2	1.252281782	0.0006324	0.368372258	0.3181685	0.950373438	0.009536

RCL1	0.621169148	0.0006347	0.418631166	0.0213475	0.705065399	0.000101
ANAPC4	-0.523270365	0.0006357	-0.279679236	0.0644183	-0.367680389	0.015464
DENND4C	-0.41765179	0.0006371	-0.340676301	0.0052853	-0.12960714	0.288329
KANK1	-0.495883851	0.0006415	-0.350268906	0.0148925	-0.309421623	0.031866
CCDC121	-0.664528773	0.0006519	-0.347978002	0.0652373	-0.433517639	0.022597
RNF113A	-0.548719346	0.0006627	-0.319201059	0.0400672	-0.299618318	0.05528
TTC39B	0.422038218	0.000668	0.363294853	0.003343	0.355472556	0.004161
GCN1L1	0.367026098	0.0006758	0.109851952	0.3091377	0.143789533	0.183
COX19	-0.657024436	0.0006793	-0.641150838	0.0008698	-0.646486556	0.000812
GPR137	0.736834637	0.0006806	0.465639243	0.0323732	0.42592306	0.05169
CEBPB	0.832764374	0.0006836	0.553361294	0.0241575	0.719987658	0.003303
GPALPP1	-0.346231655	0.0006894	-0.132426537	0.1873263	-0.099112101	0.326121
STAMBPL1	-0.646195278	0.0006993	-0.102118002	0.5830945	-0.721499641	0.000144
KIAA0040	1.480508214	0.0007009	1.185354418	0.0064706	1.353407558	0.001921
KLHDC7A	-0.85967138	0.0007036	-0.935714409	0.0001972	-0.793306371	0.001557
NELFE	0.28007279	0.0007124	0.131209548	0.1118218	0.186312331	0.024301
MAP2K3	0.79148941	0.0007165	0.929740546	6.80E-05	0.930809597	6.67E-05
P11-1220K2	1.411071573	0.0007201	1.006038564	0.0159218	1.216655209	0.003549
TCEA1	-0.294130024	0.0007231	-0.225905598	0.0090308	-0.134768202	0.119231
DAB2	0.877123997	0.0007369	0.352342447	0.175612	0.238381856	0.359924
EMP3	0.524402443	0.0007407	0.110819605	0.4768032	0.087960707	0.572676
MLEC	-0.37027311	0.000741	-0.268462475	0.0143572	-0.295108507	0.007146
CCDC43	-0.379859029	0.0007525	-0.228675522	0.039801	-0.193041531	0.083009
ROCK2	-0.428215164	0.0007532	-0.20668802	0.1030904	-0.326370728	0.010122
DDR1	0.402532864	0.0007551	0.412022165	0.0005604	0.555908543	3.20E-06
PALB2	-0.583435155	0.0007606	-0.307594972	0.0705862	-0.423806777	0.013363
PER1	1.062475245	0.0007667	0.702687896	0.0264026	0.995693843	0.001636
RCE1	0.442026194	0.0007686	0.002730627	0.9835863	0.101719468	0.44244
AGPAT3	0.336831114	0.0008001	0.22220272	0.0266579	0.283310916	0.004725
GJC1	0.589984732	0.0008071	0.602591481	0.0005992	0.370360284	0.035649
ZNF33A	-0.374811269	0.0008094	-0.127702428	0.2492182	-0.175837463	0.113365
SUZ12P	-0.378698014	0.0008128	0.030876668	0.7762014	-0.248589758	0.02535
AGGF1	-0.314713121	0.0008173	-0.073678023	0.4274636	-0.064612021	0.486839
MRPS14	-0.377572859	0.0008177	-0.1353386	0.2188336	-0.190170286	0.086341
TMEM127	0.308544555	0.0008252	-0.045985566	0.6181714	0.111705507	0.226327
ADIPOR2	0.397476862	0.0008267	0.205412783	0.0838911	0.400177156	0.000751
ABCD3	-0.300127866	0.000833	-0.218381758	0.0146431	-0.086713643	0.331995
BRICD5	-0.677892381	0.0008337	-0.723472814	0.0002935	-0.548221869	0.006138
RAB5B	0.210315998	0.0008333	-0.109126763	0.0840293	0.06625751	0.292646
ZNF69	-0.545526307	0.0008404	-0.72907422	7.67E-06	-0.580188635	0.000362
FAM177A1	0.371512996	0.0008555	0.393394587	0.0003856	0.561530866	3.95E-07
CSRP2BP	-0.449431221	0.0008617	-0.032839622	0.8032035	-0.265486398	0.046594
DDX21	0.456741196	0.0008626	0.59443147	1.43E-05	0.812409973	3.01E-09
MYB	1.24047372	0.0008697	1.189244174	0.0013894	2.121533919	9.87E-09
RANBP6	-0.354858152	0.0008804	-0.257922183	0.0146596	-0.320298536	0.00255
SETD5	0.360308709	0.0008877	0.175848654	0.1048128	0.169532171	0.11803
NECAP2	0.368841596	0.0008888	0.384886857	0.0004936	0.477165638	1.55E-05

SLAIN1	-0.670918892	0.0008907	-0.669024632	0.0008892	-0.273010386	0.173404
PDCD6IP	0.312529123	0.0008946	0.231503507	0.0137496	0.444810408	2.16E-06
HAS3	1.708579977	0.0009021	2.023985959	8.11E-05	2.022752336	8.26E-05
ZBTB7A	0.479236961	0.0009033	-0.001008014	0.9944522	0.24734123	0.087224
TNNT2	1.204416236	0.0009047	0.38512957	0.2905733	0.435029701	0.232584
SFRP2	3.164463058	0.0009068	3.085450024	0.0012012	2.06989537	0.031552
SENP2	-0.518016641	0.0009116	-0.081563423	0.6004313	0.188184197	0.226899
IRX2	-1.333156318	0.0009195	-1.009102247	0.0114049	-1.176401209	0.003294
TBC1D22B	0.441176683	0.0009262	0.301767436	0.0228972	0.50634459	0.000126
VPS26B	-0.275115753	0.0009291	-0.177098797	0.0295895	-0.256460397	0.001769
SLC9A8	0.731189351	0.0009323	0.530205302	0.0164331	0.919138795	2.96E-05
ZNF200	-0.440617186	0.0009483	-0.3341372	0.0108014	-0.512588056	0.000119
GKN1	-4.524989034	0.0009514	-3.039950367	0.0249326	-0.700273544	0.604203
NKRD36BP	0.78476271	0.0009576	0.861368498	0.0002673	1.241901043	1.29E-07
C5orf22	-0.355496453	0.0009574	-0.058184815	0.5817944	-0.251841877	0.017731
RPL38	0.445454384	0.0009706	0.385002411	0.0043434	0.306141523	0.023376
FAM179B	-0.551253108	0.0009732	-0.371689432	0.0256612	-0.325587907	0.051139
ARIH1	0.308785712	0.0009755	0.127639437	0.172048	0.19713935	0.035234
TRMU	-0.434097163	0.0009825	-0.34832445	0.0076367	-0.429466054	0.001069
C1orf131	-0.589556958	0.0009865	-0.322753934	0.0684437	-0.330577471	0.062379
CSNK1G2	0.467903288	0.0009888	0.305260963	0.0315967	0.437889175	0.002019
USP7	0.318353093	0.0010003	0.153761692	0.1115817	-0.01370598	0.887196
ZNF484	-0.38829799	0.0010101	-0.162132378	0.157481	-0.385591325	0.000927
PKDCC	-0.640868821	0.0010113	-0.737809857	0.0001522	-0.934738196	1.64E-06
CCDC112	-0.916749532	0.0010282	-0.257212707	0.3445224	-0.60084136	0.028884
LPHN1	0.407889437	0.0010349	0.178857779	0.1493742	0.088937525	0.474712
MAP3K4	-0.47126016	0.0010339	-0.232125612	0.1046555	-0.281957747	0.049014
OARD1	-0.464560324	0.0010339	-0.382078421	0.0065888	-0.151953474	0.278922
ELOVL7	0.719508745	0.001051	0.830550477	0.0001383	0.892093454	4.45E-05
MEX3C	0.667748378	0.0010563	0.347753811	0.0884037	0.450721397	0.027163
ESRRA	0.414986085	0.0010582	-0.01519479	0.9049107	0.276312278	0.028327
KCNK1	0.687262141	0.0010617	0.321955308	0.1253711	1.036859206	6.80E-07
RAB30	1.002249638	0.0010685	0.518912134	0.0909184	0.494937109	0.107722
MPHOSPH9	-0.44817383	0.0010717	-0.002303638	0.9864326	-0.180046351	0.187013
OLFM4	3.083963121	0.0010737	3.505930393	0.0001989	1.974372351	0.036596
THG1L	-0.335330693	0.0010811	-0.080604798	0.4187118	-0.093791631	0.352067
MFSD8	-0.442958416	0.0010895	-0.265543887	0.0484579	-0.070979367	0.596786
KLHL11	-0.388690875	0.0011057	-0.49539235	2.80E-05	-0.700208208	6.13E-09
NPTN	0.297257595	0.0011117	0.309220597	0.0006657	0.354334444	9.69E-05
C1orf35	-0.519948534	0.0011183	-0.397381452	0.0105219	-0.389945535	0.01328
CRTC2	0.710166418	0.0011181	0.162483426	0.4585018	0.238647055	0.276246
SLC25A46	-0.277118094	0.0011327	-0.084427649	0.3170478	0.014495243	0.863752
R3HDM2	0.568733642	0.0011411	0.388482773	0.0262629	0.24084602	0.16878
SPAST	-0.374250928	0.0011481	-0.280099804	0.0138731	-0.427677228	0.000193
SDCBP2	0.565329592	0.0011501	0.112012489	0.5200831	0.738449974	2.10E-05
LRRC58	-0.256193068	0.0011542	-0.138858277	0.0750489	-0.118636011	0.129074
VEGFA	0.716413096	0.0011629	0.181641405	0.4104265	0.687798628	0.001821

FLRT3	-0.526001336	0.0011661	-0.559761823	0.0005341	-0.790375738	1.10E-06
GATAD1	-0.311208889	0.001165	-0.141682378	0.1351343	-0.177938947	0.061097
CLK3	0.487758578	0.0011698	0.652526093	1.34E-05	0.827730383	3.30E-08
EID3	1.326542477	0.0011699	0.432434814	0.3042128	1.327826703	0.001093
PPP2R5C	-0.301817685	0.0011727	-0.087633955	0.3443588	-0.067452478	0.467075
EPN3	-1.068145067	0.0011794	-1.187261682	0.0002553	-0.974867869	0.002437
ZFP69	-0.616781126	0.0011985	-0.526927752	0.0047439	-0.325613847	0.081437
PLEKHB2	0.291364904	0.0012048	0.14392126	0.1089977	0.399013746	8.62E-06
ACOT11	-0.349387943	0.0012064	-0.432710242	5.25E-05	-0.239628107	0.025209
PHF3	-0.291782298	0.0012351	-0.245572689	0.0063957	-0.235719019	0.008909
PARG	-0.622116878	0.0012476	-0.346962868	0.0692267	-0.472807007	0.013838
SH3TC1	0.784723855	0.0012517	0.399980349	0.1004733	0.750936595	0.002017
RAB32	0.554873144	0.0012693	0.425883412	0.0129297	0.708249992	3.11E-05
SNRNP48	-0.32321586	0.0012691	-0.179454869	0.0684792	-0.201499744	0.041788
ZBTB7C	0.884039544	0.0012677	0.392031954	0.1538595	1.072332773	9.03E-05
REM2	1.237347115	0.0012894	0.680082931	0.080247	1.318330393	0.000539
LONRF1	0.432614676	0.0012977	0.30767494	0.0219767	0.781206808	5.07E-09
AZIN1	0.369693047	0.0013005	0.038246462	0.7391444	0.267024115	0.020057
EYA3	0.363186795	0.0013222	0.18599408	0.0999082	0.272712349	0.015941
KIF21A	0.501630437	0.0013261	0.143661388	0.3585348	0.621996268	6.62E-05
CPOX	-0.494557089	0.0013405	-0.179723914	0.2402157	-0.114693844	0.454409
COIL	-0.272819036	0.0013433	-0.352305904	3.02E-05	-0.463366676	5.65E-08
P11-85G20	1.134059237	0.0013434	0.789212249	0.0269883	0.787668284	0.02864
TOLLIP	0.421828559	0.0013733	0.214250697	0.1049466	0.103058355	0.435652
CSNK1D	0.362864627	0.0013786	0.064928722	0.5678376	0.213452977	0.060036
PLEKHM2	0.433201372	0.0013811	0.261178516	0.0540876	0.413652534	0.002256
NR4A3	2.698989346	0.0013861	2.478807998	0.0033181	2.667272281	0.001557
TEP1	-0.562190106	0.0013911	-0.299366397	0.0881108	-0.291396885	0.097113
TFAP4	-0.67917455	0.0014047	-0.402626918	0.0547865	-0.929071534	1.22E-05
X1A-SULT1	-0.602268019	0.001424	-0.658517272	0.000443	-0.775531054	4.07E-05
BUD13	-0.329691877	0.0014297	-0.399648979	9.80E-05	-0.447921165	1.50E-05
C5orf51	-0.261513181	0.0014278	-0.318923427	8.69E-05	-0.262298021	0.001319
HYLS1	-0.520752699	0.0014311	-0.489906499	0.0022159	-0.540727277	0.000841
MAP2K7	0.850764983	0.0014316	-0.02829657	0.9158689	0.181157363	0.4987
THUMPD1	-0.286707733	0.0014397	-0.315594607	0.0004341	-0.299090149	0.000867
CITED2	-0.636517722	0.0014444	-0.571755677	0.004054	-0.888233464	9.11E-06
NKRD20A5	-1.78778954	0.0014545	0.38073098	0.4498332	-1.273642871	0.01767
ALG6	-0.317096409	0.0014638	-0.212431123	0.0270306	-0.286607972	0.003228
ATP1A1	0.406727267	0.0014646	0.129648739	0.3105112	0.202579431	0.113059
DCLRE1A	-0.483681971	0.0014658	-0.27330643	0.0686992	-0.34942528	0.020356
PRSS22	0.735084683	0.0014623	0.099349294	0.6693625	0.756118407	0.001049
SRD5A3	0.596811536	0.0014656	0.468634975	0.0124652	1.257492693	1.67E-11
ATM	-0.390546105	0.001476	-0.082579869	0.5000725	-0.222326918	0.07003
SPG21	0.304136317	0.0014861	0.118987194	0.2128768	0.30356278	0.001433
AMER1	-0.395320235	0.0014926	-0.44892377	0.00029	-0.428120452	0.000572
BCL2L14	0.616885942	0.0014937	0.120742791	0.5362081	0.857131531	9.09E-06
ZNF419	-0.506233349	0.0014941	-0.295562586	0.0595654	-0.525369466	0.00094

ARF3	0.336479297	0.0014988	0.167021851	0.1149469	0.248418127	0.019032
COBLL1	-0.609696446	0.0015073	-0.721247763	0.0001697	-0.540567434	0.004802
LGALS9C	1.863084575	0.0015123	1.615277427	0.0066182	1.222159065	0.042974
PLEKHM1	0.772668385	0.0015127	0.039338955	0.874224	-0.147897848	0.551178
ZZZ3	-0.287509786	0.0015139	-0.182149883	0.0433711	-0.16021428	0.075729
SERTAD1	0.609671973	0.0015155	0.101975234	0.5983605	0.345842027	0.072863
ASB13	-0.630314329	0.0015273	-0.446445046	0.02268	-0.768822586	0.000109
CDCA7	-0.641520322	0.0015552	-0.195706255	0.3306412	-0.334164361	0.097355
MICAL3	0.497781823	0.0015551	0.47705124	0.0023476	0.26541981	0.090946
RBM34	-0.429336463	0.0015605	-0.224054394	0.0953813	-0.179368695	0.182503
VPS28	0.291133129	0.0015663	-0.028765765	0.7556232	0.049169309	0.594967
FRAT1	-1.143385845	0.0015701	-0.616303192	0.0768514	-0.806232186	0.020163
AREG	1.710369946	0.0015722	1.930028739	0.0003581	1.481864677	0.006176
ARHGAP11A	-0.558856829	0.0015802	-0.234141184	0.183627	-0.355575229	0.043884
IDH1	-0.392659823	0.0015967	-0.236208435	0.0573142	-0.011680231	0.925087
WTAP	0.280583337	0.0016268	0.14447076	0.1041498	0.153649901	0.084318
FOS	0.702604592	0.0016291	0.68826424	0.0020017	1.268657714	1.16E-08
MTRNR2L1	1.298362531	0.0016377	0.277663779	0.5127471	-0.186245875	0.670205
FBXO25	-0.637867377	0.0016531	-0.495301846	0.0141144	-0.200433138	0.321348
CCNI	0.342303844	0.0016596	-0.032510849	0.7652937	0.061981451	0.569151
HEG1	0.836964109	0.0016577	0.857335882	0.0012226	0.17292257	0.518228
ZNF513	0.543655789	0.0016599	0.216115357	0.2126509	0.414120065	0.016378
PIWIL2	-1.567069128	0.0016617	-0.196466207	0.6707332	-0.853688585	0.080312
A16c-17H1.	-0.838996307	0.0016886	-0.514909641	0.0430137	-0.23226874	0.355011
FAM107B	0.537190478	0.0017082	0.391402957	0.0222624	0.815393727	1.86E-06
LRRC47	-0.463909931	0.0017162	-0.264639382	0.0723211	-0.261523323	0.076333
TADA1	-0.324868426	0.0017156	-0.202082871	0.0455909	-0.300156716	0.003381
THBS1	0.910506592	0.0017139	0.964446958	0.0008947	0.663908638	0.022229
ZNF84	-0.347471904	0.0017216	-0.34551069	0.0017388	-0.439375431	7.19E-05
GAS2L3	-0.791527629	0.0017384	-0.312053445	0.2155268	-0.481767675	0.055994
BRK1	0.211150746	0.0017473	0.083605629	0.2135163	0.210576039	0.001712
CDH8	1.036603181	0.0017471	-0.094914594	0.7792456	0.480818075	0.151366
FRG1	-0.304578448	0.0017531	-0.13742619	0.13629	0.003849656	0.966643
ZBTB8B	-0.620332055	0.0017597	-0.462823003	0.0169281	-0.892626587	8.16E-06
RNASEL	-0.392867945	0.0017739	-0.295882332	0.0156092	-0.305269187	0.013303
POT1	-0.501465929	0.0017784	-0.3003862	0.059168	-0.35728495	0.025128
DFFB	-0.608100069	0.0017814	-0.307454689	0.1072964	-0.620236976	0.001326
ELP5	0.285930252	0.0017891	0.00783783	0.9317761	0.170371907	0.062921
PHC2	0.33192635	0.0017991	0.159118347	0.1343576	0.258496455	0.014996
PSMC4	0.379489983	0.0018056	0.19044059	0.1171135	0.456142623	0.000166
MRPL44	-0.301325882	0.0018115	-0.279529972	0.0033597	-0.348624317	0.000286
MTRF1	-0.431979887	0.0018211	-0.317493318	0.0200516	-0.178456976	0.189392
C6orf89	0.265425314	0.0018319	0.021832606	0.7975774	0.207522461	0.014719
ZNF331	-0.431083762	0.0018388	-0.462971393	0.0007567	-0.459753616	0.000904
NME4	0.566702334	0.0018466	-0.207328997	0.2563652	-0.140324868	0.442507
CCDC90B	-0.270094917	0.0018521	0.01033267	0.9032554	-0.192446303	0.025049
IL6ST	0.431463199	0.0018603	0.377134627	0.0065109	0.39879183	0.004018

CIC	0.480397762	0.0018621	-0.000954503	0.995075	0.205290421	0.184164
ARRDC2	0.723689473	0.0018668	0.2884053	0.216933	0.851153111	0.000239
ASTE1	-0.79750942	0.0018651	-0.561411706	0.0273287	-0.789361912	0.001985
KDELR1	0.35656007	0.0019002	-0.189691326	0.0989357	-0.065139814	0.571099
LSR	0.526542401	0.0019026	0.188665263	0.266177	0.561742166	0.00092
POC5	-0.503929324	0.0018999	-0.095508914	0.5418423	-0.028039125	0.858127
RPL14	0.367414259	0.0019033	0.085235498	0.4712592	0.258393151	0.028956
ZNF33B	-0.769250975	0.0019035	-0.543489113	0.0253425	-1.007602412	4.39E-05
FLOT2	-0.471714696	0.0019077	-0.568274615	0.0001813	-0.253347036	0.094182
POU2F3	1.671877112	0.0019104	1.494579937	0.0054004	2.325450938	1.33E-05
ZNF252P	-0.378426907	0.0019172	-0.311832411	0.0099851	-0.269255775	0.026333
TAB2	0.336070573	0.0019211	0.050844936	0.639063	0.236484893	0.028967
HELLS	0.703612363	0.0019229	0.567925505	0.0122716	0.848942299	0.000181
LRRC4	0.916583001	0.0019339	-0.105853714	0.7214696	1.207007851	4.24E-05
UTP14C	-0.236656665	0.0019438	-0.2073258	0.0059153	-0.273125155	0.000323
NDNL2	-0.447474947	0.0019575	-0.455618433	0.0014426	-0.44189007	0.002078
RHOV	1.435377253	0.001962	1.247463084	0.0070937	1.83356413	6.94E-05
C1orf109	-0.475703598	0.0019669	-0.396326167	0.0091278	-0.479198095	0.001661
TTC4	-0.324884013	0.0019783	-0.306884371	0.0030002	-0.309041323	0.003039
INTS6	-0.26159187	0.0019816	-0.327512286	0.0001038	-0.230713313	0.006253
ZSCAN29	-0.293134422	0.0019973	-0.412904023	1.13E-05	-0.417764524	1.04E-05
CA13	-0.486434461	0.0020181	-0.459741179	0.0033527	-0.609602258	0.000108
COL9A2	0.856218267	0.002026	0.59211114	0.0326768	0.762034502	0.005904
ZNF625	-0.773852006	0.0020357	-0.448204998	0.067091	-1.031973074	3.72E-05
JADE2	-0.563647268	0.0020377	-0.599630986	0.001007	-0.65435279	0.000345
CHKA	-0.473313412	0.0020396	-0.625260207	4.38E-05	-0.374433943	0.014169
C2orf44	-0.411340062	0.0020469	-0.372104945	0.004454	-0.586501552	1.07E-05
RSC1A1	-0.547691163	0.0020501	-0.364524496	0.0364644	-0.324180064	0.063469
PQLC1	0.423807889	0.0020611	0.064426183	0.6408565	0.282855402	0.039078
LIN9	-0.486362036	0.0020663	-0.18803561	0.2242388	-0.272381215	0.080344
MAP4K4	0.391677805	0.0020668	0.391337809	0.0020576	0.505677528	6.86E-05
EPB41L2	-0.472774171	0.00207	0.089108546	0.5600319	-0.166577886	0.277057
TMEM184B	0.455731672	0.0021033	0.227303843	0.1251575	0.452049664	0.002247
TMEM57	-0.404603213	0.0021058	-0.172935216	0.1860594	-0.216529272	0.098135
ZHX1	-0.297785411	0.0021182	-0.297577448	0.0020378	-0.219870997	0.022643
EPHA1	-0.500640229	0.0021202	-0.442760303	0.0064297	-0.352464838	0.029522
KLHL21	-0.549262863	0.0021223	-0.449476693	0.0116089	-0.432690935	0.015189
MTATP6P1	0.9675036	0.0021318	0.294248289	0.3521739	-0.010165999	0.974481
SLC7A1	0.521692562	0.002134	0.413246789	0.014869	0.788608627	3.25E-06
GSK3A	0.4539761	0.0021387	0.122507448	0.4082489	0.059707497	0.688164
PARP16	-0.50160129	0.0021379	-0.364720253	0.0226428	-0.319163378	0.046973
TCF20	0.245818644	0.0021436	0.142332421	0.0731597	0.143565528	0.071977
DRAM1	0.58151546	0.0021472	0.553195742	0.003448	0.765188925	5.04E-05
FAM118B	0.384595745	0.0021534	0.537022666	1.55E-05	0.59918088	1.43E-06
FAM83G	0.5188605	0.0021538	0.670691934	6.60E-05	0.77769844	3.70E-06
RPP30	-0.298334708	0.0021816	-0.137528982	0.1469609	-0.114143832	0.232111
SERPINE1	1.625294558	0.0021823	1.119997301	0.0348182	1.158393438	0.02907

MYLIP	-0.446225304	0.0021915	-0.709586457	1.14E-06	-0.61913565	2.19E-05
DNAJB1	0.29572645	0.0021995	0.019149251	0.8430789	0.225105576	0.01973
P11-480I12	-0.898055639	0.0022041	-1.007306013	0.0005583	-1.333661581	7.59E-06
JD7-PLA2G	-1.03749623	0.0022248	-0.590133513	0.0790306	-0.141448109	0.674872
DHX9	0.265677477	0.002232	0.174721921	0.0441592	0.222212682	0.010495
ISG20L2	0.524796046	0.002233	0.383837691	0.0253793	0.362476171	0.034889
B2M	0.42598581	0.0022381	0.384487771	0.0057919	0.65447806	2.63E-06
\M47E-STBI	-0.754804612	0.0022381	-0.684491623	0.0054199	-0.367154923	0.135233
ABHD10	-0.370327719	0.0022402	-0.087209469	0.4634121	-0.260701803	0.029424
CBX8	-0.758763109	0.0022537	-0.91452687	0.0001573	-1.032115321	2.47E-05
ısa-mir-119	-0.82793343	0.0022549	-0.826046184	0.0021528	-0.73369821	0.005411
RASGRP3	0.73921039	0.0022645	0.832351004	0.0005703	1.288371389	8.14E-08
MEIS3P1	0.575803981	0.0022796	0.402689042	0.0322243	0.568648978	0.002401
RAP2B	0.316791959	0.0022766	0.071818765	0.4890888	0.633512595	8.45E-10
TEFM	-0.57249225	0.0022776	-0.224880971	0.2206571	-0.288852044	0.117607
UNC119B	-0.291984987	0.0022788	-0.22416058	0.0179842	-0.428406192	7.57E-06
SAP30BP	0.342461345	0.0022837	0.292553944	0.0090227	0.368866473	0.000988
SMC4	-0.450087247	0.0022995	0.116701799	0.4281834	-0.218041469	0.139134
GDI1	0.326299661	0.0023038	0.095885905	0.370061	-0.013995239	0.896314
ADCY10P1	-1.24168038	0.0023089	-0.788737557	0.0478952	-0.83314182	0.037165
ATMIN	-0.269725831	0.0023109	-0.069583702	0.4282382	-0.248107604	0.004942
RDH14	-0.383515135	0.0023086	-0.347262973	0.0050677	-0.128631101	0.296289
PRUNE	-0.459629331	0.0023263	-0.488727116	0.0012252	-0.465749447	0.002073
ANKRD32	-0.644396796	0.00233	-0.145970966	0.4852779	-0.186482632	0.373626
MAT2B	-0.262218342	0.0023322	-0.165920234	0.0501917	-0.18439513	0.030493
FAM160A1	0.710036743	0.0023446	0.652059435	0.0052731	0.963947696	3.49E-05
MLK4	-0.515383606	0.002347	-0.394491143	0.0188858	-0.157068535	0.348178
C1orf64	1.202766095	0.0023616	1.154391408	0.0031767	0.727966694	0.071967
SALL4	0.703850119	0.0023683	0.704868873	0.002303	0.432359162	0.0621
WRNIP1	-0.290960386	0.0023706	-0.160500238	0.088135	-0.086986375	0.356107
LYN	0.614764397	0.0023839	0.808840635	5.72E-05	1.190392862	2.91E-09
ZFPM1	-0.882372155	0.0024002	-0.808095322	0.0053903	-0.955469751	0.001009
DRD1	1.083521509	0.0024188	1.421830817	5.41E-05	1.03899771	0.003537
FBRS	0.493215206	0.0024259	0.082932166	0.6124523	0.159455604	0.32958
BIK	1.233514101	0.0024382	1.107175027	0.0063904	1.803985287	7.16E-06
SSH2	0.558784377	0.0024373	0.232122702	0.2094408	0.482204426	0.008828
CENPA	-0.766951209	0.0024588	-0.089511829	0.7138123	-0.43271083	0.079166
TOR4A	0.491163581	0.0024684	-0.043104237	0.7917762	0.339445396	0.036461
UFSP2	-0.438204084	0.0024777	-0.061927372	0.6656433	-0.020028132	0.889036
PELI3	-0.66169185	0.0024801	-0.063349242	0.7598512	-0.303592377	0.148609
DERL2	-0.324985212	0.002485	-0.320957741	0.002616	-0.232709966	0.029573
PTGER4	1.182608659	0.0025061	0.750206075	0.0551385	0.596924979	0.127016
CDC40	-0.554241378	0.0025166	-0.290514636	0.1115356	-0.426887318	0.019553
RGS16	1.063261054	0.0025174	1.420884355	4.84E-05	2.003727099	8.90E-09
PDP2	-0.613087565	0.0025305	-0.252574095	0.2088363	-0.553568004	0.006062
TMEM223	-0.358719844	0.0025404	-0.132974817	0.2486047	-0.299428178	0.010513
ZBTB5	0.329808458	0.0025416	0.085622071	0.4333604	0.10686304	0.329469

SLBP	-0.358439855	0.0025583	-0.179999067	0.126841	-0.12765017	0.279313
ZNF700	-0.501075462	0.0025756	-0.403489033	0.0138885	-0.362387999	0.02756
TMEM80	-0.563157656	0.0025852	-0.48369629	0.0088994	-0.390755142	0.034928
OVOL2	-1.103580251	0.0025962	-1.2173262	0.0007826	-0.828594689	0.016757
SHPRH	-0.449457117	0.0025965	-0.265512925	0.0740295	-0.44269319	0.002975
ZNF30	-0.855184335	0.002614	-0.625670784	0.0249706	-0.893549532	0.001621
EBPL	-0.334464634	0.0026549	-0.067490604	0.5335469	-0.122063895	0.263388
SDHAF1	-0.532208685	0.0026543	-0.544197676	0.0017352	-0.627396676	0.000376
SMG8	-0.35870545	0.002657	-0.544213045	4.97E-06	-0.70999087	3.60E-09
ZFP14	-0.539009754	0.0026563	-0.298132697	0.0918889	-0.267630496	0.133264
OXNAD1	-0.35900124	0.0026648	-0.138044578	0.2350104	0.009287557	0.935817
ZNF717	-0.808483252	0.0026785	-0.327207111	0.2159243	-0.69536974	0.009849
P11-206L10	-1.270518461	0.0027023	0.423509937	0.2897837	-0.407045931	0.319307
NAB1	0.335952266	0.0027093	0.36473824	0.0011322	0.503084941	6.95E-06
TPCN1	0.48798022	0.0027251	0.067075205	0.6814621	-0.147319042	0.368003
DNAL1	-0.561940048	0.0027404	-0.324323066	0.0836749	-0.227020968	0.225763
MMAB	-0.363752141	0.0027422	-0.271060772	0.0246133	-0.071319253	0.553824
PRKCZ	-0.504148681	0.0027418	-0.471592903	0.0049197	-0.50514522	0.002614
RN7SL467F	-0.898113217	0.002747	0.39277377	0.1307207	0.067481133	0.800914
MRPS6	-0.264064018	0.0027534	-0.356624798	4.67E-05	-0.369707064	2.69E-05
IFT81	-0.432726871	0.0027646	-0.103284305	0.4679613	-0.105925995	0.460548
TES	0.258211821	0.0027643	0.122889512	0.1538019	0.421542953	9.51E-07
SACM1L	-0.310607871	0.0027675	-0.2197903	0.0327803	-0.162393837	0.115753
AKIRIN1	0.340179985	0.0027761	0.210909922	0.063408	0.170613573	0.133518
BHLHE40	0.754957697	0.0027767	0.926232032	0.0002394	1.78733424	1.29E-12
PHACTR2	0.315596338	0.0027768	0.223315418	0.0341246	0.436117595	3.48E-05
SYNGR2	0.383299269	0.002779	0.137677778	0.2826308	0.567499596	8.91E-06
MFN2	0.340638989	0.0027932	0.171716529	0.1309499	0.137087205	0.228427
KCNE3	-0.989012204	0.0028116	-1.123724966	0.0006798	-0.141561008	0.667745
MPV17L2	-0.424341907	0.0028294	-0.007104071	0.9575627	-0.126220569	0.353272
NDUFC2	-0.308049785	0.0028265	-0.121152052	0.236211	-0.085933244	0.401533
SLC25A32	0.382598312	0.0028278	0.314548653	0.0137441	0.498758603	9.28E-05
CINP	-0.279570451	0.0028357	-0.215582834	0.0179938	-0.072815542	0.425488
WWC1	0.552331378	0.0028532	0.631818743	0.0006236	0.668055794	0.000296
ANKRD49	-0.390061867	0.0028583	-0.389864346	0.0027573	-0.328818148	0.011478
SRPK1	0.437375648	0.0028564	0.483714451	0.0009631	0.699268744	1.81E-06
GEM	0.779213225	0.0028817	1.011776172	0.0001063	0.317633074	0.224957
CHCHD4	-0.56497878	0.002895	-0.377245262	0.0424151	-0.2707504	0.144709
MRPS34	-0.448989991	0.0028935	-0.315742235	0.034441	-0.157966578	0.289105
RAPGEF1	0.437808186	0.0029062	-0.046385825	0.7527385	0.113711727	0.439746
SZRD1	0.306732706	0.0029306	0.012105051	0.9064654	0.087932288	0.393517
EPS8	0.343817035	0.0029339	0.321881826	0.0053005	0.435549867	0.000161
PSTPIP2	0.897238856	0.0029745	0.954272024	0.0015718	1.305935073	1.47E-05
TMEM30A	0.368366806	0.0029762	0.089364231	0.4710789	0.292807168	0.018206
AGO2	0.555968989	0.0030031	0.602907226	0.0012852	0.751988193	5.94E-05
SPPL2A	0.334027921	0.0030065	0.310896287	0.005649	0.469210076	2.93E-05
THUMPD2	-0.424719936	0.0030336	-0.119548914	0.3926035	-0.281725197	0.046216

SLC16A3	0.996644464	0.0030492	0.275908637	0.4118362	0.570214341	0.089958
ZNF439	-0.873603868	0.0030591	-0.499935143	0.0815786	-1.035708855	0.000534
CHEK2	-0.400036708	0.0030699	-0.263896723	0.0480284	-0.116215935	0.382982
SAMD14	0.705187628	0.0030769	0.358800876	0.1340806	-0.478504378	0.054576
TCEAL8	-0.247532175	0.0030778	0.005659853	0.9453718	-0.016193738	0.844963
TMTC4	-0.376827971	0.0030757	-0.173896737	0.1647395	-0.148473658	0.237157
SNRNP35	-0.367301397	0.0030828	-0.236514656	0.0517332	-0.243090212	0.047177
SAMD8	0.330140203	0.0030858	0.212557931	0.0555983	0.347852591	0.001755
NUP93	0.44566212	0.003089	0.473309553	0.0016183	0.326503077	0.029618
NUDT16	-0.314086057	0.0030953	-0.151129214	0.1500677	-0.316378499	0.002793
TOR1AIP2	0.185019267	0.0030945	0.102258281	0.1016848	0.01817589	0.771535
CCNF	-0.541539807	0.003132	-0.391791085	0.031569	-0.536019658	0.003381
FOXO3	0.341337393	0.0031357	0.400136269	0.0005126	0.32913683	0.004318
GRAMD1B	-0.886440892	0.0031383	-1.064499444	0.0004092	-0.885669153	0.003104
MAP4K2	0.571276714	0.0031512	0.0702793	0.7165402	0.348719351	0.072063
CELF1	0.318032693	0.0031721	0.176200683	0.102219	0.286573342	0.007815
RP11-384K6.	0.675500786	0.0031741	0.116132406	0.6164449	0.056478584	0.808877
AACS	-0.346231745	0.003192	-0.284619925	0.0148473	-0.147058926	0.207678
BBS10	-0.412983288	0.0031876	0.055095034	0.6818546	-0.305088583	0.026796
SHISA9	0.599653661	0.0031905	0.811175725	5.56E-05	0.738573479	0.000255
DNAJC21	-0.249307247	0.0032028	-0.086458707	0.3027466	0.008321343	0.921071
PROX2	-1.170684224	0.0032115	-0.340526875	0.3099397	-0.88796309	0.015234
PET117	-0.594517401	0.0032234	-0.395357269	0.0448187	-0.395450027	0.04581
TRAF4	0.33896332	0.0032331	0.042629672	0.711411	0.279390563	0.015165
FAM150B	1.148927013	0.0032502	1.34847688	0.0004587	1.032147644	0.007936
ACTR6	-0.415002024	0.0032708	-0.283794852	0.0398863	-0.314649014	0.022757
RAB11FIP4	-0.566819488	0.0032866	-0.386849155	0.0439602	-0.265014597	0.165836
KDM5C	0.239396722	0.0032929	0.027635342	0.7345108	0.095116384	0.243445
GNL1	0.470931923	0.0033031	-0.036899212	0.8180282	0.080394596	0.616002
ARMC7	-0.464523468	0.0033073	-0.781476084	1.29E-06	-0.887945807	7.16E-08
ARL4A	-0.676211461	0.0033107	-0.702866561	0.0021856	-0.665344018	0.003755
SMAD2	0.198546309	0.0033178	-0.001988174	0.9764996	0.218170016	0.001225
GATAD2A	0.277788733	0.0033329	0.218232041	0.0208063	0.221499742	0.019131
PDHB	-0.215076905	0.0033527	-0.181990526	0.0122397	-0.052501099	0.469977
ZBTB6	-0.285833572	0.0033502	-0.259478106	0.0068248	-0.32784201	0.000711
AC040977.1	0.492566318	0.003357	0.220569115	0.1892945	0.285217945	0.089798
SLC6A15	1.351152831	0.0033612	0.159444311	0.7328577	0.301073366	0.516515
FAM76A	-0.350855882	0.003368	0.116114281	0.3129011	0.071282662	0.539493
WWC3	0.476464367	0.0033758	0.539961798	0.0008258	0.496734649	0.002185
ZNF180	-0.375409812	0.003378	-0.446333067	0.0004514	-0.426851712	0.000817
GSPT1	0.320247509	0.0033906	0.349845251	0.001353	0.506720097	3.44E-06
SNURF	-0.4517386	0.0033919	0.021949802	0.8833471	-0.493438422	0.001292
TMEM64	-0.348486476	0.0033878	-0.073380878	0.5342678	-0.215507922	0.068392
C19orf44	-0.701015891	0.0033948	-0.418796485	0.0645134	-0.754433706	0.001455
ELF2	-0.261461471	0.0034036	-0.271400458	0.0022098	-0.28501434	0.001318
NFXL1	-0.457705534	0.0034032	-0.38421413	0.0132836	-0.487394175	0.001758
SS18	0.287776277	0.0033989	0.340874711	0.0004955	0.353960256	0.000301

ZC3H7A	-0.455627428	0.0034083	-0.263500225	0.0896216	-0.308600501	0.047049
CCDC85B	1.067740167	0.0034128	0.482121843	0.1915414	0.876678494	0.016387
MEGF11	0.888673412	0.0034267	0.295237557	0.3301635	0.500871456	0.097543
DCTN3	0.332753135	0.003437	0.045438331	0.6893909	0.00560617	0.961016
CD3EAP	-0.632902379	0.0034446	-0.594161497	0.0058238	-0.699464948	0.001242
RP11-396K3.	-0.435964267	0.0034467	-0.01876181	0.8994416	-0.523460052	0.000443
CEP44	-0.401366774	0.0034589	-0.321525372	0.0180397	-0.360778809	0.008335
KIAA1143	-0.464973661	0.0034647	-0.347429716	0.0280442	0.003530212	0.982223
SEC24A	0.379251432	0.0034661	0.243285757	0.0606684	0.537192701	3.39E-05
STAT6	0.266605099	0.0034651	0.245217602	0.0071293	0.339768121	0.00019
TMEM92	0.658764542	0.0034919	-0.423461856	0.0630067	-0.120602894	0.594572
MKS1	-0.504100801	0.0035016	-0.271023538	0.1146288	-0.745922747	1.65E-05
NIPSNAP3A	-0.298471024	0.0035113	-0.129645878	0.1885455	0.111344065	0.253369
APC	-0.302921774	0.0035422	-0.265596933	0.010355	-0.411311782	7.51E-05
NCCRP1	1.200694687	0.0035403	0.802219885	0.0528592	0.998352438	0.015555
RCOR3	-0.406279223	0.0035415	-0.244709273	0.0766478	-0.341050589	0.014125
ING5	-0.412072683	0.0035484	-0.281366148	0.0450452	-0.18007917	0.19962
MB21D1	0.852657876	0.0036002	0.505685662	0.0869886	1.342021774	2.87E-06
FBXW2	-0.269882786	0.003628	-0.184993287	0.0449885	-0.151901412	0.100489
MRPL33	-0.277603278	0.0036263	-0.226907583	0.0153019	-0.196843542	0.036023
GPSM2	-0.503889736	0.0036509	-0.11587491	0.5017821	-0.007871419	0.963616
ZNF702P	-0.521063017	0.0036648	-0.715947332	6.62E-05	-0.332282192	0.062454
IL33	-1.381558254	0.0036696	-0.359825256	0.4377789	-0.347675198	0.452247
L3HYPDH	-0.548363624	0.0036936	-0.318966959	0.079426	-0.136934397	0.449388
MTG2	-0.32912038	0.0036949	-0.203702757	0.0670577	-0.317754669	0.00472
IRS4	1.761820394	0.0036999	0.635556058	0.3022248	1.484631398	0.014615
FAM200A	-0.37474615	0.0037126	-0.27831467	0.0268413	-0.387573125	0.002493
IRF2BPL	0.593648008	0.0037087	0.497600337	0.0148841	0.53330711	0.009088
SCRN1	0.234211843	0.0037129	0.079794689	0.322478	0.192138732	0.017168
CH25H	1.351649797	0.0037183	1.549704707	0.0008029	1.962577527	2.02E-05
TBCC	-0.351186628	0.003719	-0.264922694	0.0258202	-0.45119573	0.000187
SIN3A	0.274700358	0.0037539	0.099897753	0.2912907	0.159409637	0.09215
ASB8	-0.282151376	0.0037725	-0.303142205	0.0015192	-0.242747216	0.011057
PIGA	0.675348872	0.0037926	0.817927783	0.000445	1.470259444	2.56E-10
MPHOSPH6	-0.37196316	0.0038033	-0.13023274	0.2994833	-0.295337936	0.01984
ZNF182	-0.430845844	0.0038104	-0.303705222	0.0363404	-0.315069092	0.030517
BCDIN3D	-0.547839331	0.0038245	-0.408415914	0.0278129	-0.397969012	0.033557
YAE1D1	-0.370526525	0.0038263	-0.145334139	0.246049	-0.373829471	0.003267
ERN1	0.617732925	0.0038339	0.808226623	0.0001504	0.786701433	0.000227
CHD2	0.41505816	0.0038444	0.068512643	0.6333495	0.386661524	0.007072
QTRT1	0.492509403	0.0038973	0.323932196	0.057897	0.258356291	0.131079
CASP7	1.290673038	0.0039211	1.019202389	0.0227533	1.219742851	0.0064
MRPL35	-0.325891819	0.0039372	-0.094255863	0.3988029	-0.140575697	0.210713
CTDSPL	-0.28067539	0.003953	-0.324493997	0.0008257	-0.322722825	0.000918
DCP1A	0.511119256	0.0039517	0.321298307	0.0696424	0.098151562	0.583952
PSMC2	-0.245090704	0.0039642	-0.080372707	0.3405726	-0.038002263	0.652436
C9orf152	-0.982848337	0.0039747	-1.199308336	0.0004479	-0.69228403	0.040234

DNAJC24	-0.413295994	0.0040031	-0.035501816	0.799949	-0.223809366	0.1143
HDHD3	-0.372006092	0.0040212	-0.59977939	3.41E-06	-0.301011358	0.019303
JUP	0.470444118	0.0040148	0.259519559	0.1125293	0.616847813	0.000161
KRT15	-0.804783078	0.0040209	-1.138155132	4.75E-05	-0.707037013	0.011058
ORMDL1	-0.244326141	0.0040197	-0.016787717	0.8404732	-0.065940653	0.431554
MAD2L1	-0.398709758	0.0040458	-0.000295713	0.9982822	-0.222258164	0.106797
TPBG	0.58380522	0.0040815	0.221355171	0.2767034	0.748597213	0.000221
KLF9	0.976545664	0.0040909	0.653690851	0.0548535	0.68834344	0.043352
FANCM	-0.502005589	0.0040992	-0.332881738	0.0537507	-0.198780515	0.250774
ZFP62	-0.437907286	0.0041024	-0.301049867	0.0468526	-0.199333342	0.189426
DENR	-0.201141717	0.0041081	-0.003665876	0.9578905	-0.093585069	0.179304
FAM156B	-1.09146696	0.0041387	-0.982269221	0.0093567	-1.013266283	0.007488
UFL1	-0.27965746	0.0041469	-0.231514461	0.0169623	-0.126049164	0.193548
IGF2BP1	0.390192455	0.004178	0.175773342	0.1964568	0.108245462	0.426718
AKIRIN2	0.418152045	0.0042026	0.306552736	0.0343978	0.439980814	0.002493
YWHAH	0.242341517	0.0042034	0.134889472	0.1104033	0.495606773	3.96E-09
CCDC34	-0.447864951	0.004214	-0.363699474	0.0184568	-0.40829691	0.008687
SF3B4	0.281202646	0.0042138	-0.109009714	0.2719295	0.042679249	0.666672
FAM187A	-0.992323245	0.0042202	-0.951597645	0.0057052	-0.559022633	0.100036
ALKBH5	-0.273017	0.0042311	-0.188914123	0.0467607	-0.009389782	0.921159
IL6	1.935042331	0.0042464	1.780636182	0.0085308	1.4944991	0.028158
LIMD2	0.654685283	0.0042434	0.559322116	0.0143276	0.351674355	0.125861
TMEM39A	0.437816131	0.0042455	-0.188822687	0.2181353	0.216272742	0.157256
SNAPC4	0.434426801	0.0042625	0.331666973	0.0285542	0.352689517	0.020127
DBI	-0.338432236	0.0043229	-0.088801179	0.452848	0.071518185	0.545322
RASSF9	-0.709727893	0.0043244	-0.934451196	0.0001711	-0.747124474	0.002603
DMXL1	0.329998915	0.0043494	0.462949527	6.08E-05	0.42263292	0.000251
ELK4	0.397189727	0.004341	0.088068912	0.5271894	0.185054331	0.183895
GJA1	0.711543304	0.0043443	0.507310037	0.0420024	0.693607536	0.005427
SAP30	-0.440625165	0.0043435	-0.144382506	0.3207408	-0.30755438	0.04004
TMEM70	-0.367229963	0.0043497	-0.372391626	0.0034069	-0.246765619	0.05218
FGF23	0.816637857	0.0043677	0.501889968	0.0805874	-0.04800151	0.870828
DHRS13	-0.423242546	0.0043756	-0.491697363	0.0009139	-0.184672975	0.204157
LTBP4	0.600436524	0.0043843	0.458424876	0.0296241	0.30453316	0.148862
F816-ZNF32	-0.600452753	0.0043855	-0.389365936	0.0624132	-0.071913118	0.729698
TFF3	0.796413756	0.0044142	-0.102553238	0.7139889	0.905209248	0.001212
ZCCHC4	-0.440224542	0.0044143	-0.127974702	0.3945305	-0.175825076	0.246603
C1orf172	-1.064498739	0.0044267	-1.989172298	1.54E-07	-1.229926396	0.001012
NR0B2	-0.869581826	0.0044363	-1.182145253	0.0001114	-1.036150983	0.000701
KCTD10	0.320545431	0.0044468	0.187912568	0.0941731	0.220306368	0.050308
FBXO9	-0.326491618	0.0044601	-0.113916229	0.316486	-0.151830894	0.182477
CBR4	-0.339700628	0.0044676	-0.186243478	0.1130237	-0.23008754	0.051309
SMARCAD1	-0.219006232	0.0044656	-0.010656329	0.8888571	-0.143149011	0.061629
GPR39	-0.660724267	0.0044713	-0.825429454	0.000377	-0.582838339	0.011809
LRP11	-0.302251637	0.0044782	-0.313408428	0.0030326	-0.146108023	0.166666
KIF20B	-0.419857755	0.0045089	-0.064575437	0.660178	-0.16347519	0.266478
C3orf38	-0.245986871	0.0045442	-0.095252134	0.2625073	-0.221383798	0.009999

CYCS	0.284337836	0.0045824	0.388613556	0.0001024	0.576790545	8.05E-09
CASZ1	0.861284957	0.0045986	0.570481811	0.0615679	0.813924594	0.007483
FBXO18	-0.250000935	0.0046257	-0.078434399	0.3706965	-0.159746708	0.069027
LRR1	-0.453342299	0.0046413	-0.329228842	0.0384279	-0.60479568	0.000154
RP4-669L17.	0.865988536	0.0046472	-0.239961897	0.4447079	-0.085731627	0.784358
AB019441.2	-0.844548969	0.0046524	-1.068446171	0.000337	-0.899469458	0.002356
YWHAZ	0.222275531	0.004658	0.011671954	0.8818558	0.325854143	3.31E-05
ZNF420	-0.620153912	0.0046553	-0.755002913	0.0005394	-0.911008855	3.00E-05
BMPR1APS	6.388509824	0.0046687	6.134064621	0.0066024	6.391125651	0.004635
FBXO5	-0.393807383	0.0046637	-0.196143315	0.1548401	-0.428485345	0.002024
USP47	-0.237178108	0.0046658	-0.072524761	0.3854394	-0.022034852	0.792078
FBXO34	0.32662844	0.0046981	-0.017254349	0.8814797	0.311463322	0.006941
TLR7	1.378022641	0.0047247	1.533924373	0.0013682	1.845354543	0.000115
ZNF181	-0.410664477	0.0047331	-0.183071995	0.195574	-0.239816989	0.095802
KIAA0556	0.542665528	0.0047394	0.264250638	0.1707547	0.852287365	8.70E-06
NR2F2	-0.790224147	0.0047442	-0.589077081	0.0347933	-0.783345363	0.005078
PEX2	-0.246713883	0.0047582	-0.082827489	0.3381658	-0.06812405	0.432021
MYO10	0.459936388	0.004777	0.537619934	0.0009605	0.334771838	0.039971
GLIPR2	0.629541774	0.0048054	0.187058383	0.4032648	0.630952355	0.00463
NEFM	1.948356525	0.0048251	0.537533429	0.4407872	0.233825154	0.739752
TMEM2	0.340956891	0.0048384	0.118351344	0.3280574	0.40552464	0.000801
USP1	-0.261350733	0.0048597	-0.202169465	0.0286659	-0.048185313	0.601765
RBM26	-0.268301281	0.0048723	-0.164054964	0.0836201	-0.315176109	0.000923
PDS5B	-0.363547849	0.0048879	-0.056969572	0.6573473	-0.11684467	0.363916
ZNF14	-0.48990459	0.0049011	-0.616909903	0.0003653	-0.527943687	0.002299
PFDN4	-0.397196103	0.0049113	-0.067581748	0.6200255	-0.162326227	0.240447
CCDC104	-0.375221797	0.004923	-0.234262729	0.0764639	-0.116078912	0.380401
GADD45B	1.018060446	0.0049474	0.997496883	0.0057433	1.36135887	0.000154
ATG3	-0.378261028	0.0049613	-0.018817487	0.8876488	-0.020566665	0.877519
ZNF782	-0.528593035	0.004981	-0.319498597	0.0811638	-0.373251618	0.044227
CHD9	-0.319060242	0.0049964	0.016333179	0.885479	-0.06952591	0.539993
PLA2G6	0.588432436	0.0050003	0.069594164	0.7416565	0.047252938	0.824343
KLHL29	0.434020856	0.005053	0.421935813	0.0060929	0.070973365	0.649004
POLR3B	-0.379686329	0.0050581	0.047782709	0.721179	0.044758939	0.738144
COX6C	-0.262807083	0.0050729	-0.025197429	0.7852596	-0.002283234	0.980344
UBTF	-0.337954597	0.0050706	-0.230037708	0.0556117	-0.308750906	0.010298
RELA	0.669475271	0.005077	0.703368605	0.0032167	0.851288248	0.000362
PRSS3	1.695188593	0.0050928	1.554362818	0.0098122	1.811618533	0.002477
AHDC1	0.70533095	0.0051047	-0.152092482	0.5483428	0.284953046	0.259189
DNAJA1	0.375460735	0.0051069	0.180659115	0.1778053	0.457383309	0.000643
LPAR2	0.410213515	0.0051012	0.168390935	0.2508216	0.351511755	0.016174
LZTFL1	-0.37456437	0.0051106	-0.259566181	0.0513676	-0.132794257	0.318214
ITPR1	0.627536209	0.0051238	0.266783262	0.2344389	0.331159919	0.139548
NKX3-1	1.186571732	0.0051361	1.668311024	7.03E-05	1.616275888	0.000121
PI4K2A	0.311320904	0.005142	0.02636148	0.8129822	0.078682193	0.48076
CD248	0.582200443	0.0051481	0.204569682	0.3262912	-0.078582727	0.707429
NXF1	0.422999682	0.0051735	0.250191844	0.0989211	0.097755014	0.519355

PRR15	-0.474693393	0.0051786	-0.675492097	6.82E-05	-0.272129052	0.105787
RFT1	-0.377418797	0.005192	-0.208447265	0.1178291	-0.046119638	0.729622
NSMAF	-0.318270388	0.0052026	-0.045795864	0.6837294	-0.190756479	0.090393
CUL4B	-0.286300279	0.0052157	-0.304948149	0.0028751	-0.322806326	0.001612
CYP2R1	-0.554759059	0.0052259	-0.34635184	0.0758722	-0.463021946	0.017746
SLC38A9	-0.362836896	0.0052301	-0.29053921	0.0235887	-0.003442981	0.97848
HIBCH	-0.530442775	0.005243	-0.343403476	0.0696699	-0.468722098	0.013295
RFC4	-0.527607201	0.0052551	-0.41097572	0.028782	-0.326908811	0.08229
CNOT6L	-0.283998664	0.0052816	-0.226648772	0.0245565	-0.205529793	0.042024
CUTC	-0.481731461	0.0052785	-0.196368449	0.2417892	-0.243198382	0.147797
OXA1L	0.295580049	0.0052808	-0.09871068	0.352896	-0.119169061	0.263019
SENP5	0.345325545	0.0052704	0.33030812	0.0075312	0.227630799	0.065694
SRP14	-0.432004708	0.0052812	0.127235296	0.4099947	0.091560074	0.553394
CA8	0.928937961	0.0053349	0.455498821	0.1718624	0.739560969	0.026118
IPCEF1	1.413762155	0.0053319	0.78701071	0.1198279	0.875606152	0.081717
SRSF10	-0.232651152	0.0053388	-0.034276758	0.6806434	-0.195210978	0.019372
CYTIP	-1.431651507	0.005354	-1.410803014	0.0063381	-0.716554733	0.153334
ELAVL1	0.240024663	0.0053726	0.209561009	0.014964	0.136565907	0.113286
ZNF441	-0.508448251	0.0053831	0.260983485	0.1351356	-0.559793277	0.001963
PNKD	0.293643763	0.0053888	-0.085175642	0.4217754	0.175914685	0.095097
CASP6	-0.360348288	0.005398	-0.259327172	0.0423033	-0.150721285	0.237042
RUNX2	0.714437029	0.0054334	0.384917513	0.1348684	0.899522793	0.000404
SLC9A6	-0.304578027	0.0054439	-0.274069913	0.011247	0.005389307	0.959914
LSM6	-0.351197241	0.0054553	0.080357478	0.5129983	-0.171093476	0.168532
STAT3	0.356578938	0.0054704	0.194174956	0.1302097	0.367580752	0.004162
TCAIM	-0.317611843	0.0054711	-0.273271504	0.0163254	-0.148124893	0.192226
POLR3K	-0.548633512	0.0054796	-0.481690811	0.0127521	-0.428460447	0.026827
ZNF675	-0.35134744	0.0054787	-0.298622156	0.0166273	-0.350389365	0.005281
AGPS	-0.346132931	0.0054911	-0.22345414	0.0723242	-0.024231407	0.845329
ZMYND19	-0.366690224	0.0054988	-0.447662668	0.0006477	-0.363074637	0.005692
C1QL1	1.178424504	0.005513	1.077987466	0.0106217	0.121253366	0.787501
DHX16	-0.363623772	0.0055359	-0.175446568	0.1782353	-0.2795134	0.032325
FKBP1A	0.379985798	0.0055228	0.182069109	0.1837943	0.359712122	0.008567
GIT2	-0.33164416	0.0055202	-0.219181324	0.0638366	-0.394301381	0.000953
HCCS	0.339486626	0.0055357	0.128085124	0.2915461	0.39617937	0.001072
PCDH19	1.480056172	0.0055289	0.10952095	0.8389652	1.305665103	0.013765
PLK1	-0.755379944	0.0055238	-0.224095615	0.4074037	-0.383860681	0.156812
RAB34	0.349569879	0.0055077	0.154520412	0.2194607	0.144334435	0.251919
UHMK1	-0.18010135	0.0055311	-0.232374837	0.0003223	-0.196980545	0.002323
PEX19	0.27052002	0.0055548	-0.137276481	0.1586357	0.024475432	0.801704
ZCCHC9	-0.342379823	0.0055653	-0.168733984	0.1664526	-0.250859412	0.040968
HOXB7	-0.820276024	0.0055837	-0.68601015	0.019208	-1.442362202	1.86E-06
ZNF260	-0.324476698	0.0056122	-0.351273752	0.0026098	-0.516655407	1.06E-05
BORA	-0.681167755	0.0056187	-0.675931859	0.0057228	-0.772693597	0.001667
ZNRF3	-0.46622953	0.0056219	-0.04681942	0.7767949	-0.31356589	0.059864
AMMECR1L	0.191814011	0.005635	-0.070889751	0.3069116	0.167112473	0.015243
CDC42	0.208807533	0.0056547	0.13601824	0.0712644	0.235201079	0.001806

ZYX	0.529488336	0.0056507	0.309560042	0.1058227	-0.001269152	0.994721
CCNG2	0.503833084	0.0056968	0.434906613	0.0168346	0.71340786	8.72E-05
DTNB	-0.556769621	0.0057007	-0.136148196	0.4960434	0.060942723	0.760219
ADTRP	1.223618377	0.0057192	0.977363643	0.0256345	1.759965141	3.18E-05
BLOC1S5	-0.384216716	0.0057163	-0.738744806	1.03E-07	-0.346093353	0.012357
P11-261C1(	0.915660998	0.0057118	0.265001709	0.4324156	0.031876824	0.926153
G0S2	1.014751849	0.0057696	0.379770389	0.3050521	1.372187179	0.000171
C12orf44	0.37414745	0.0058019	0.221237859	0.1026954	0.334671296	0.013475
TJP1	0.363112029	0.0058343	0.290768857	0.0271344	0.465374381	0.000403
ID1	-0.601733632	0.0058537	-0.371321559	0.0882383	-0.213416822	0.327012
ATXN2L	0.415652341	0.0058639	-0.12557785	0.4069021	-0.133667902	0.378555
ZNF329	-0.335317681	0.0058625	-0.534720133	1.03E-05	-0.588376294	1.54E-06
MDH2	0.361542388	0.0059023	0.090842708	0.4886633	0.128773708	0.326512
PRDX3P1	1.209971026	0.0059074	1.173610367	0.0068976	1.27246105	0.003395
AP1G1	0.196203377	0.0059232	-0.159306503	0.0252857	0.035570848	0.616978
AMN1	-0.355347777	0.0059365	-0.214848458	0.0909403	-0.286995871	0.024879
RNF135	-0.537533989	0.0059374	-0.041057293	0.8275716	-0.083718214	0.657877
TMEM163	0.614325522	0.0059291	0.08576546	0.7016939	0.582989804	0.008949
CREB5	1.047966471	0.0059462	0.568156625	0.1364244	1.011200935	0.00796
RPP25	-0.650532268	0.0059671	0.081033551	0.7228213	-0.126798585	0.582226
WDFY1	-0.190548795	0.0059852	-0.154355755	0.0253349	-0.054474811	0.429593
WSB2	0.296138307	0.0060017	-0.017073285	0.8741522	0.10407683	0.334414
LRRC45	-0.497672903	0.0060175	-0.591413962	0.0010534	-0.338254338	0.058018
CCDC169	0.767276939	0.0060236	1.122446907	3.96E-05	0.721853242	0.0088
GOLPH3	0.348772224	0.0060423	0.164579731	0.1950006	0.326276376	0.01017
ICA1L	0.766630723	0.0060383	0.861468166	0.0018309	0.365674147	0.189699
RCN1	0.354504241	0.0060753	0.098691457	0.4445309	0.159008767	0.21821
SF3B5	0.304347184	0.00607	0.042559918	0.701375	0.147042616	0.185123
UPF3A	-0.349902695	0.0060748	-0.065359096	0.604468	-0.21503021	0.089566
ABHD13	-0.234297136	0.0060966	-0.268306513	0.0015205	-0.298663659	0.00045
GRB14	-0.745182107	0.006135	-0.501203934	0.0613616	-0.546063003	0.042663
KPNA4	0.238002196	0.0061682	0.240739957	0.0055466	0.341395074	8.39E-05
ZNF131	-0.319754336	0.0061666	-0.128786904	0.266623	-0.28185695	0.015392
CERKL	-0.597084546	0.0062152	0.266949612	0.1993285	-0.201860514	0.346409
DOT1L	0.465853932	0.0062289	0.059903217	0.7254939	0.284194212	0.095704
NRAS	0.187655581	0.0062514	0.112492877	0.1001957	0.268194207	8.74E-05
ABHD15	-0.512345941	0.0062716	-0.638816513	0.0006163	-0.203997241	0.268099
CLDN18	0.761330035	0.0062737	0.077066276	0.7820537	1.104804273	7.30E-05
FA2H	0.640297308	0.0062906	-0.09121376	0.6980704	0.457372181	0.050815
TNFRSF19	-0.632598477	0.0063062	-0.524838199	0.0228714	-1.183427423	3.81E-07
TRMT13	-0.409031397	0.0063124	-0.046853301	0.7516002	-0.043794304	0.767515
P11-691N7.	1.691180635	0.0063497	1.766336204	0.0041283	1.440128796	0.020432
LRRC37A3	-0.741930917	0.0063572	-0.153145477	0.5677736	-0.323557495	0.230365
PSPC1	-0.405577638	0.0063828	-0.073057916	0.6176388	-0.133127226	0.36558
STEAP4	0.909614228	0.0064106	1.10898926	0.0008345	1.847205388	2.06E-08
NFE2	-1.312270306	0.0064404	-1.313846567	0.006108	-1.712774484	0.000407
VWA7	0.760003078	0.006439	0.540415685	0.0544961	0.517728355	0.065008

C16orf46	0.702934828	0.0064522	0.26434964	0.3188449	0.19752865	0.458263
CTU2	0.605856252	0.006484	0.268946063	0.2298683	0.388619716	0.080886
EIF4G3	0.225374226	0.0064898	0.17795925	0.0307556	0.132345582	0.10941
.36A-HNRNI	-1.882975584	0.0064952	-0.396780189	0.5351991	0.19499779	0.758043
DNASE1L1	0.653453887	0.0065081	0.001128872	0.9962445	0.314172373	0.188701
CSTB	0.615567212	0.0065133	0.56911039	0.0119019	1.171683133	2.19E-07
CRLS1	-0.292013851	0.0065258	-0.106771314	0.3143027	0.012471971	0.906524
FER1L6	0.666447604	0.0065566	-0.058063839	0.8128783	0.913857957	0.000192
NSMF	0.475116175	0.0065711	0.063706503	0.7164501	0.03964168	0.821524
ARHGAP31	0.953798153	0.006591	0.822122879	0.0191183	0.693692771	0.048352
TMEM167A	-0.364604798	0.006602	-0.152272913	0.2559725	-0.238704168	0.075144
DCP1B	-0.40955645	0.0066156	-0.314296094	0.0350393	-0.621611796	4.39E-05
RPL28	0.39934328	0.0066164	0.168133648	0.2529142	0.358270296	0.014827
JOSD1	0.378215899	0.0066353	0.048472642	0.7277019	0.421070164	0.002463
RNF170	-0.305178174	0.006672	-0.206415196	0.0637351	-0.217678431	0.052103
IRX5	-0.938893629	0.0067091	-1.353870898	0.0001062	-0.3888526	0.247615
GRAMD3	0.505794554	0.0067453	0.303708319	0.1037191	0.768862662	3.28E-05
SPRTN	-0.392109957	0.0067673	-0.469808183	0.0011158	-0.609394391	2.50E-05
MST1R	0.651905517	0.0067952	-0.171352067	0.4783885	0.782564627	0.001081
TSEN2	-0.664453881	0.0068117	-0.234723466	0.3355023	-0.189945071	0.436079
ZNF436	-0.461957278	0.0068655	-0.264066909	0.1207379	-0.247972243	0.145349
IRF3	0.325048055	0.0068979	0.212409064	0.0773103	0.286825518	0.016753
KCMF1	0.36963595	0.006904	0.16392969	0.2299165	0.5206689	0.000135
LRCH4	0.528494099	0.0068996	-0.035622443	0.8563399	-0.057618955	0.770061
STAG3	-0.647371766	0.0069141	-0.347916189	0.1387469	-0.684062465	0.004272
SLC15A4	0.492900006	0.0069225	0.489126221	0.0069664	0.90671461	4.34E-07
DOPEY1	-0.325287624	0.0069403	-0.18255039	0.1285756	-0.213046221	0.076344
ZNF41	-0.329557396	0.0069735	-0.200623034	0.0954768	-0.175139951	0.144326
CXorf40A	-0.490179297	0.0069934	-0.402563404	0.024731	-0.347013495	0.053913
DIDO1	-0.205555332	0.0069981	-0.151548196	0.0457098	-0.220783603	0.003708
RANBP3	0.339881142	0.0069936	0.201590656	0.1088742	-0.029924759	0.812245
PTCD3	-0.204291415	0.0070067	-0.074027145	0.3253686	-0.177373106	0.019031
AC005042.2	1.127131805	0.0070611	0.909470909	0.0295868	1.215588711	0.003373
MANEA	-0.47996455	0.007079	-0.360610243	0.0415612	-0.261302178	0.140527
MAP1S	0.622632832	0.0070976	0.083538041	0.7183289	-0.035196744	0.879224
ZNF250	-0.466373643	0.0071028	-0.151977559	0.375331	-0.245252858	0.155425
RAP2A	-0.200258774	0.0071169	-0.155942584	0.0340821	-0.244057395	0.000981
AP1B1	0.279291738	0.0071599	0.138669983	0.1811639	0.349512642	0.000739
SPAG7	-0.19003017	0.0071681	-0.091592502	0.1876687	-0.028166078	0.685356
IKZF3	0.590524488	0.007206	0.892708917	3.70E-05	0.861351056	6.96E-05
MICALL1	0.345429177	0.0072054	-0.047062325	0.7133948	0.309779888	0.015506
PDLIM5	0.377833916	0.0072176	0.238263791	0.0900571	0.405968289	0.003873
TMEM65	-0.217460264	0.0072307	-0.112322658	0.1565008	-0.121915404	0.126389
NR4A2	0.958830541	0.0072381	0.244733476	0.4969287	1.07598347	0.002499
NDUFB3	-0.289720043	0.0072451	0.060671184	0.5647196	0.06826753	0.518364
GAREM	0.421594321	0.007251	0.372501954	0.0176859	0.420534243	0.007353
BRMS1	-0.290809414	0.0072785	-0.242674962	0.0229505	-0.059773049	0.573136

C2orf69	-0.253062719	0.0072747	-0.299251085	0.0013201	-0.230559814	0.013486
PRR22	0.676090737	0.0073053	0.008342975	0.9745661	0.480286219	0.057439
P11-466H18	0.371591801	0.0073049	0.305864007	0.0271381	0.338785276	0.014413
PRKD2	0.428955171	0.0073139	0.250188542	0.1180372	-0.01107415	0.944962
ACSL4	0.525602075	0.0073266	0.581073517	0.0030214	0.71186178	0.000279
F3	1.300030341	0.0073304	1.363922343	0.0048828	2.357685055	1.12E-06
RPS19BP1	-0.338837234	0.007331	-0.496432648	7.85E-05	-0.371546233	0.003229
P1-130H16.1	0.602303535	0.0073591	0.182296281	0.420978	0.470724684	0.03613
P11-274B21	0.96286762	0.0073684	-0.230696843	0.5460088	0.395365026	0.282462
ZNF286A	0.32263333	0.0073695	0.259121737	0.030995	0.370969673	0.002045
TTC30A	-0.334547121	0.0074047	-0.155301878	0.203908	-0.193458964	0.115853
USP22	0.234203897	0.0074018	0.176123448	0.043855	0.155710739	0.074873
DHX15	0.185131176	0.0074211	0.22400269	0.0011726	0.249497519	0.0003
DUT	-0.42147162	0.0074976	-0.275389887	0.0786477	-0.145350238	0.351651
NSD1	-0.23613176	0.0075117	-0.098526817	0.2628096	-0.114571971	0.193612
DGAT2	0.612289658	0.0075206	0.608689205	0.0075624	0.440371585	0.054775
ZIK1	-0.454414356	0.0075376	-0.556437125	0.0010425	-0.558937074	0.001024
SMAD1	0.575568074	0.0075487	0.99885357	3.19E-06	0.862741288	5.94E-05
ANKRD65	-1.549980581	0.0075542	-1.875799797	0.001254	-0.453940944	0.398186
ARL15	-0.550457832	0.007574	-0.007723591	0.9696886	0.150067367	0.460578
APEX2	0.306472538	0.0075975	0.141828041	0.2159002	0.052670446	0.646475
ACTR2	0.195869519	0.0076227	0.024124199	0.7420763	0.183945412	0.012036
LSM11	-0.223935703	0.0076901	-0.321169749	0.0001046	-0.330354102	7.98E-05
C19orf53	0.205541241	0.0077057	-0.028643109	0.7102802	0.043967982	0.568874
MIA2	-0.885805783	0.0077102	-0.018286696	0.9548258	-0.601857496	0.066998
NOC4L	0.565773323	0.0077287	0.403900669	0.0567657	0.572467414	0.006805
IL17RE	-0.63728658	0.0077875	-0.535580445	0.0251761	-0.123679951	0.604042
CAPRIN1	0.186056845	0.007798	0.187760112	0.0071864	0.186913235	0.007486
CCNB1	-0.542368931	0.007822	-0.296595353	0.1449092	-0.418616296	0.03979
FAM213A	-0.276293816	0.0078214	-0.205688876	0.0456945	-0.13138486	0.202337
HSBP1L1	-0.422225792	0.0078226	-0.292370666	0.0617055	-0.227796036	0.14585
AC004057.1	1.048926111	0.0078378	1.227655869	0.0018488	1.086969767	0.005851
CCNA2	-0.461677657	0.007865	0.034939692	0.8389249	-0.115060324	0.504514
FAM46B	-0.781081989	0.007863	-1.209985322	5.48E-05	-1.381486918	8.13E-06
SRF	0.262831741	0.0078707	0.00231585	0.9813291	0.018577945	0.851418
GMPR2	-0.301793169	0.0078906	-0.241729094	0.032653	-0.306735395	0.006861
ARMC1	-0.236990956	0.0079117	-0.275915774	0.0017961	-0.168019989	0.057425
AUH	-0.479800056	0.0079353	-0.242352434	0.1739447	-0.132183248	0.460026
ZNF470	-0.350522021	0.007943	0.02530675	0.8455889	-0.251702859	0.054025
SMEK1	-0.191632466	0.0079679	-0.154904989	0.0314756	-0.161119136	0.025389
STOML1	0.589946436	0.0080063	0.081805918	0.7129642	0.60248535	0.006663
SNRPB2	-0.253612858	0.0080184	-0.015449446	0.8703002	-0.015326018	0.871374
XDH	0.7384313	0.0080673	0.632801583	0.022784	0.548209919	0.048201
AC024560.3	-0.377146549	0.0080735	-0.329951167	0.0185815	-0.401449507	0.004581
PPWD1	-0.294188159	0.0080817	-0.223017567	0.0426964	-0.201882705	0.067389
DNMT3B	-0.483019041	0.008178	-0.215261017	0.221009	-0.476463394	0.008086
CBX4	-0.712455461	0.0082241	-0.552819	0.0387459	-0.722942628	0.007256

CPHL1P	0.912580225	0.008229	0.291136503	0.3992921	1.292524399	0.000155
FNDC1	1.479176984	0.0082242	0.784102418	0.1638672	1.598414856	0.004178
TTK	-0.497779795	0.0082334	-0.114759821	0.5399086	-0.143117297	0.445571
SLC25A6	0.327079216	0.0082533	0.111956624	0.3657186	0.178998073	0.148223
DCLK3	1.667263214	0.0082624	1.516964615	0.0154675	2.12595926	0.000656
THAP3	-0.489541139	0.0083115	-0.337240713	0.066095	-0.635055561	0.000783
MALSU1	-0.291252428	0.0083298	-0.091599939	0.3935933	-0.274352476	0.011005
ZNF146	-0.21017967	0.008332	-0.190370141	0.0165363	-0.094348106	0.234835
CLN8	0.421439124	0.0083386	0.056614741	0.7231201	0.325862995	0.041428
TD-2623N2	-0.881252106	0.0083612	-0.704949143	0.02849	-0.701860096	0.030501
TMEM179B	0.331657933	0.008358	0.015946434	0.8986281	0.277046157	0.026228
UTP6	0.279144525	0.0083647	0.080579112	0.4440857	0.112340219	0.286314
HRSP12	-0.348186173	0.0083855	-0.046270927	0.7172024	-0.121668978	0.346081
THUMPD3	-0.333917657	0.0083967	-0.162465059	0.1964029	-0.182174883	0.148579
ZNF655	0.179125859	0.0083974	0.091715709	0.1754595	0.21645023	0.001384
IPO8	-0.436792971	0.0084386	-0.182312133	0.268919	-0.398636219	0.015813
DACT2	-0.922161453	0.0084529	-0.543694355	0.1137201	-0.517287264	0.132986
AOC2	0.867898889	0.0084723	0.010564289	0.9753381	-0.092628193	0.789116
CYP3A7	1.189132771	0.00848	0.942671103	0.0376995	1.654566442	0.000181
ANKRD50	0.38442838	0.0085325	0.229601183	0.1159492	0.280127136	0.055193
ZNF549	-0.380375765	0.0085296	-0.35758518	0.0126466	-0.557483507	0.000117
TOMM34	0.283538031	0.0085482	0.379163775	0.0003833	0.480840257	6.66E-06
MCL1	0.394799511	0.0085717	0.26634966	0.0761197	0.630638475	2.64E-05
ZNF140	0.327046065	0.0085878	0.208572178	0.0929938	0.40728745	0.001012
MED29	0.94860575	0.0085963	0.239866795	0.5114315	0.737407882	0.039763
RIMKLBP2	-1.094427313	0.0086068	-0.278774125	0.4369549	-0.521708839	0.162993
ZNF510	-0.347929822	0.0086217	-0.355460875	0.0067293	-0.471835081	0.00037
USF2	-0.325554222	0.0087492	-0.111237493	0.3661102	-0.06345195	0.606525
TTC3	-0.200335154	0.0087615	-0.313935219	3.95E-05	-0.203623884	0.007683
HMMR	-0.544415944	0.0087747	-0.116150656	0.572726	-0.221968733	0.282402
SH2D3A	0.403717614	0.0087765	0.227122052	0.1401638	0.604533774	8.09E-05
POP7	-0.309322224	0.0087906	-0.326647894	0.0049526	-0.330042698	0.004771
USP45	-0.463481727	0.0087867	0.057435236	0.741234	-0.192622223	0.270123
NEDD1	-0.275880486	0.0088062	0.092762333	0.371006	0.082609824	0.42649
FOSB	1.432174216	0.008838	1.065448231	0.0515262	0.296709708	0.592913
ARHGAP12	-0.263227546	0.0088748	-0.372810903	0.0002051	-0.186779345	0.06251
BYSL	0.456300929	0.0088604	0.052950187	0.7623415	0.188318925	0.282169
RUNDC1	-0.280983829	0.0088651	-0.415235182	0.0001059	-0.419532347	9.56E-05
SERF2	0.26628851	0.0088636	-0.03835377	0.7062956	0.343869642	0.000717
ZNF214	-0.752286636	0.0088729	-0.397845811	0.1555727	-0.526445923	0.06936
ZNF704	0.628784961	0.008868	0.543982131	0.0234481	0.732949734	0.002258
MTIF2	-0.380613083	0.0089155	-0.175727514	0.223048	-0.297697427	0.039566
PLTP	0.451965329	0.0089327	-0.129355348	0.455659	0.278783985	0.106873
RCN2	-0.245562467	0.0089456	0.106203228	0.2533145	-0.086982002	0.352001
ALDH7A1	-0.396428014	0.0089818	-0.09561005	0.5256927	0.020575334	0.891537
ATP5SL	0.35501379	0.0089926	-0.001733508	0.9898464	-0.00753476	0.955975
IFT172	0.417975292	0.0089925	-0.03047615	0.8486746	0.06573001	0.68172

VGLL4	0.233519552	0.0089835	-0.005425651	0.9513867	0.091429792	0.304123
FOXL1	0.746240007	0.0090453	1.470750263	2.02E-07	1.290905402	5.49E-06
GKN2	-3.563529996	0.0090413	-2.609252282	0.0541715	-0.882312377	0.513684
NDUFA4	0.260354633	0.0090568	0.1851605	0.0630304	0.157415277	0.114352
TAF10	0.360795815	0.0090602	0.429479995	0.0017657	0.515845019	0.000173
TP53BP2	0.384051329	0.0090631	0.510657872	0.0005115	0.741213906	4.46E-07
ZMIZ2	0.379441006	0.0090614	0.046626445	0.7492356	0.048723521	0.739112
CKAP2	-0.395869339	0.0090725	-0.189722542	0.2098714	-0.428978652	0.004653
TWF1	-0.248521939	0.0090755	-0.076330645	0.421876	0.05528225	0.560617
MTERFD3	-0.391200522	0.0090841	-0.333209022	0.0242413	-0.315354421	0.033411
SYDE1	0.645407937	0.0090994	0.240010335	0.3319505	0.091315914	0.713209
PDLIM3	0.781355277	0.00912	-0.348113376	0.2533074	0.249638201	0.407939
DIAPH1	0.48072616	0.0091668	0.043306577	0.8146898	0.429354659	0.01992
TOR1B	0.361440859	0.009168	0.186387289	0.1789919	0.303427261	0.027843
ZNF24	-0.191546842	0.0092028	-0.379379473	2.42E-07	-0.284498335	0.000108
HLA-A	0.401013842	0.0092594	0.015029724	0.9223363	0.070324561	0.64829
WBP5	-0.248049812	0.0092664	-0.072919773	0.4390485	0.076726064	0.414988
ZNF641	0.422023154	0.0092728	0.167594699	0.300686	0.479501852	0.003085
FHL3	0.492533363	0.0093297	0.240282177	0.2048408	0.372200479	0.049867
RRP36	-0.238742494	0.0093384	-0.031732308	0.7249103	-0.014775539	0.870205
RHBDD2	0.326726961	0.0093659	0.179220999	0.1553216	0.494065646	8.38E-05
ADAMTS1	0.353803829	0.0093861	-0.022318836	0.8701273	-0.144179667	0.291159
HSPA1A	0.469554103	0.0094147	0.421212611	0.0197968	0.546919342	0.00248
PRKX	-0.40932822	0.0094341	-0.173280839	0.2693382	-0.114505522	0.466494
PSMB10	0.335007612	0.0094919	-0.119752104	0.3579253	0.368570679	0.003848
IFI35	0.431801162	0.0095052	0.327742644	0.0475352	0.319648903	0.052876
MRPS35	-0.343340504	0.0095019	-0.316445459	0.016523	-0.058988612	0.654381
CDCA8	-0.47836373	0.0095298	-0.37598029	0.0405261	-0.319361219	0.082085
MTUS1	-0.419727371	0.00952	-0.505709976	0.0017782	-0.474925513	0.003337
ZNF688	-0.623721535	0.0095266	-0.235087428	0.3164667	-0.137954669	0.559647
MED25	0.389691047	0.0096263	-0.132548681	0.3827034	-0.048604185	0.75031
METTL17	-0.241215547	0.0096343	-0.185646917	0.043854	-0.355436161	0.000125
SOWAHA	-0.723340289	0.0096476	-0.272738274	0.3144315	-0.242542214	0.372166
FBRSL1	0.658559018	0.0096646	0.306654068	0.2283265	0.568258721	0.025735
SLC43A2	0.707225931	0.0097115	0.35145244	0.1983862	0.215385242	0.43331
ARMC10	-0.248397876	0.0097517	-0.247002331	0.0092155	-0.281680339	0.003237
DFNA5	-0.676280083	0.0097317	-0.032157259	0.9003077	-0.433895188	0.094998
GK5	-0.400844225	0.0097549	0.027883244	0.8557699	-0.183013862	0.233534
GPATCH2L	0.269432619	0.0097664	0.05586624	0.5918323	0.156953446	0.132673
RBM25	0.232557623	0.009776	0.00012312	0.9989083	0.072394181	0.421143
RHBDF1	0.429028021	0.0097745	0.157217358	0.3445757	0.417097322	0.011943
UNKL	0.875445843	0.0097513	0.785047548	0.0197768	0.242517489	0.473686
UTP20	0.259057367	0.0097699	0.214856788	0.0312508	0.302379152	0.002437
ZADH2	-0.399689862	0.009751	-0.23083584	0.1316755	-0.359271696	0.019688
ZBTB44	-0.210814155	0.0097357	-0.057204258	0.4790936	-0.018337524	0.820853
PRPSAP1	-0.330737933	0.0098347	-0.137685254	0.2774392	-0.191064473	0.134099
ATXN1L	0.209915197	0.0098644	0.026963632	0.740603	0.036496176	0.654547

STRIP1	0.334233307	0.0098751	0.259616732	0.0448668	0.235986522	0.068757
ASUN	-0.372989824	0.0099194	-0.130187077	0.3646327	-0.060356555	0.674655
MOB4	-0.297618267	0.0099394	-0.298283388	0.0093818	-0.129543199	0.258766
CMC1	-0.489839901	0.0099537	-0.469669884	0.0117345	-0.278842064	0.138726
P11-644F5.:	-0.631044915	0.0099892	-0.125917959	0.5957796	-0.277252352	0.245862
ZNF12	-0.27247704	0.0100066	-0.031608108	0.7632561	0.00696799	0.947128
MTX3	-0.373915523	0.0100415	-0.26495766	0.0637146	-0.284939456	0.047423
SELK	0.472544211	0.0100438	0.089241041	0.6276321	0.367106309	0.045462
CAMK1G	0.865241452	0.0100745	0.400945493	0.2433277	0.641717263	0.054111
EXOC1	-0.311303264	0.010104	-0.286016902	0.0177739	-0.297802139	0.013744
LGMNP1	-0.737066936	0.0100914	-0.194349587	0.4696228	0.156677471	0.553794
SCYL3	-0.235114979	0.0100919	-0.173251597	0.0509252	-0.109580576	0.219857
ZNF415	-0.443139937	0.0101032	-0.111610851	0.5098727	-0.3113824	0.068861
AUNIP	-0.517380529	0.010127	-0.547689137	0.005982	-0.382129161	0.054684
C1orf159	0.445603539	0.0101544	0.232978377	0.1750001	0.271322513	0.115506
HINT3	-0.23622471	0.0101692	-0.062281817	0.4906484	0.061446257	0.496072
PTX3	1.637801543	0.0101596	1.281191005	0.0444005	1.439912119	0.023847
PUS7L	-0.33624083	0.0101687	-0.250648084	0.0546532	-0.185051402	0.156746
ZNF786	-0.427774935	0.0101453	-0.370193814	0.023175	-0.519379616	0.001735
DYRK4	-0.337176779	0.0101943	-0.108873081	0.3901126	-0.15927788	0.214521
FAM167A	0.771071237	0.0102001	0.083210474	0.7864487	0.380694034	0.211388
TPRN	-0.462698151	0.0102253	-0.499451062	0.0056366	-0.692793932	0.000125
MFSD2A	0.76225284	0.0102316	1.071476335	0.000267	1.482561736	3.64E-07
ICMT	-0.279700397	0.0102531	-0.168115591	0.1211945	-0.090510961	0.404261
NFATC3	-0.300234385	0.0102406	-0.20125032	0.0839088	-0.418266181	0.000342
SEMA7A	1.288465183	0.0102586	1.177670947	0.0189337	1.869676319	0.000191
VN1R2	-0.956255643	0.0102502	0.037508201	0.9077474	-0.182236491	0.58442
TBC1D2B	0.40807464	0.0102697	0.416715402	0.0087119	0.260526705	0.10145
CLINT1	0.311843487	0.0103185	0.049593442	0.6832599	0.422777598	0.000501
ACPP	0.619434874	0.0103753	0.165662208	0.4938914	1.084146941	6.06E-06
TUBB6	0.536822369	0.0103727	0.601370266	0.0040341	0.440579854	0.035354
ALG1	-0.34257616	0.0103819	-0.25347417	0.0556125	-0.191676058	0.147427
ENGASE	-0.387613103	0.0103919	-0.696481137	4.57E-06	-0.647597651	1.95E-05
ZSCAN16	-0.392044559	0.0104067	-0.469393724	0.0018311	-0.405056343	0.007355
AARS2	-0.250270671	0.0104546	-0.276623012	0.0041381	-0.386877866	7.44E-05
MLF2	0.211962693	0.010455	-0.092525736	0.2643848	-0.040708071	0.623838
NACC1	0.503980598	0.0104609	0.173542507	0.3780479	0.183423373	0.352078
EPRS	-0.311014103	0.010478	-0.099377245	0.4124507	0.138855505	0.2519
TAF13	0.362793757	0.0104918	0.087926046	0.5357244	0.48812602	0.000536
SCAI	-0.54737379	0.0105433	-0.147297839	0.4900159	-0.174948837	0.413586
GDPD3	-0.621164667	0.010586	-0.395396203	0.0934976	-0.18313142	0.430222
PTP4A2	0.299034995	0.0105865	0.278045203	0.0174259	0.480961269	3.90E-05
FMNL3	0.685035861	0.0106136	0.586427155	0.0285689	0.223186828	0.406153
ZNF211	-0.447167757	0.0106101	-0.421865363	0.0153295	-0.515367815	0.003106
XRCC2	-0.431290601	0.0106289	0.02219905	0.8941569	-0.184545408	0.270599
YEATS2	-0.260055303	0.0106901	-0.146522941	0.1471586	-0.277477855	0.006387
EXO5	-0.428877656	0.0106982	-0.730873082	1.33E-05	-0.460372518	0.006395

CRY1	0.440338462	0.0107475	0.213405362	0.2163213	0.272219461	0.114713
IFRD1	0.296620094	0.0107962	0.037508032	0.7473422	0.198135349	0.088328
ANKRD9	-0.427423205	0.0108281	-0.45042112	0.0068749	-0.09613127	0.563859
LSM1	-0.272614454	0.0108449	-0.137915931	0.1892501	-0.038210046	0.716266
MAN2B1	0.335518535	0.0108789	0.118750984	0.3672167	0.437563975	0.000878
PNMA1	-0.373168852	0.0108812	-0.020256247	0.8883431	-0.331167013	0.023067
ATG16L1	-0.272681814	0.0109172	-0.129104978	0.2190292	-0.373006898	0.000458
CXCL17	1.665904883	0.0109192	0.816300581	0.2168549	3.175089454	8.53E-07
DIS3L	-0.299124578	0.0109316	0.08510791	0.4629742	-0.09282193	0.424727
CHPT1	-0.533216187	0.0109476	-0.108848984	0.6002006	-0.28397385	0.173831
PLGLB1	-0.455148576	0.0109531	-0.14618206	0.3866019	-0.193659964	0.254857
ANXA2	0.277122338	0.0109741	0.211991679	0.05164	0.55990484	2.73E-07
MRPL19	-0.290456665	0.0109677	-0.088253438	0.4350426	-0.023976595	0.832186
TEX30	-0.392674888	0.0111183	-0.170043769	0.2537743	-0.212797002	0.156957
SLC30A4	0.575309974	0.0111369	0.656050364	0.0036219	1.028691472	4.66E-06
GAL	1.604347377	0.0111636	1.709114559	0.0062984	1.409295291	0.026089
SPR	-0.422069561	0.0111871	-0.197317933	0.2275667	-0.118380863	0.46937
KIAA0753	-0.367939482	0.0112028	-0.175763695	0.2206361	-0.218188681	0.129623
VKORC1L1	-0.321501768	0.0112144	-0.122093979	0.3337756	0.075028689	0.552111
ZNF649	-0.300689358	0.0112132	-0.194445567	0.0968189	-0.345787179	0.003443
TBX15	0.563634992	0.0112339	0.469165067	0.0341941	0.483212594	0.029574
NSUN6	-0.416723903	0.0112544	0.091978592	0.5648496	-0.090410189	0.57361
CEP170B	0.430903486	0.0112849	0.161504305	0.3424736	0.266756701	0.11662
ABCA10	1.451254552	0.011318	1.281781268	0.0258594	1.615857312	0.004778
DNAJB14	-0.226649833	0.0113238	-0.008723995	0.9217995	-0.099045593	0.266113
PHPT1	0.241537597	0.0113137	-0.118280269	0.2164517	-0.018620791	0.84568
AMPD3	0.724862749	0.011332	0.430582244	0.1326523	0.29298958	0.306246
AP3D1	0.292548523	0.01135	0.117560275	0.3080075	0.142554545	0.216554
ATP5EP2	-3.785649668	0.0113465	-1.497378697	0.2796714	-1.145754556	0.406801
CALM1	-0.269304195	0.0114019	-0.231341399	0.02956	-0.061117543	0.565363
CAMKK1	-0.501184411	0.011429	-0.703543159	0.0003684	-0.752307315	0.000175
METTL14	-0.34318499	0.0114224	-0.184262824	0.171769	-0.270228897	0.045579
ZNF677	-0.349545235	0.0114287	-0.251062767	0.0684882	-0.358173543	0.009674
VASP	0.271222188	0.0114397	-0.004604786	0.9657973	0.169924122	0.112826
PCYT2	-0.312409312	0.0114591	-0.396197306	0.0012637	-0.182313845	0.137406
CC2D2A	-0.383903345	0.0115211	-0.073610708	0.6233221	-0.312901121	0.038545
CDH13	1.036557753	0.0115249	0.676716183	0.0993707	0.398082651	0.333881
CHML	-0.263465792	0.0115079	-0.257909113	0.0128516	-0.188467189	0.069231
GOLGA2P5	-0.565026427	0.0115266	-0.503152876	0.022732	-0.963549161	1.99E-05
P11-22B23.	-0.496393272	0.0115242	-0.560574045	0.0039627	-0.585956856	0.00278
RSAD1	-0.379915334	0.011511	-0.702936798	2.68E-06	-0.803767673	9.76E-08
FAM58A	-1.700315286	0.0115726	-1.674390685	0.0116917	-0.689463408	0.295408
INHBA	1.008792844	0.0115943	1.252193941	0.0017042	1.360227681	0.000653
TOP2B	-0.256929263	0.0115894	0.12952706	0.2019493	0.128414821	0.206355
CHTF8	-0.299487477	0.0116384	-0.288348536	0.0151948	-0.158716854	0.18143
SCO1	-0.274657903	0.0116357	-0.151753058	0.1603727	-0.034333505	0.751318
TBC1D31	-0.337330198	0.0116363	-0.047464466	0.7143022	-0.216945327	0.097125

ZNF75D	-0.344313187	0.0116685	-0.409951989	0.0023393	-0.148543621	0.268069
ZNF449	-0.359791381	0.0116912	0.1166913	0.3963015	-0.002992347	0.982767
AC068279.3	-1.318736025	0.0117341	-0.553301669	0.2606916	-0.959447047	0.057564
SRRT	0.499615497	0.011732	0.296509576	0.135318	0.266170341	0.180828
ZNF239	-0.447448859	0.0117249	-0.298246653	0.0862255	-0.380375118	0.030221
METTL9	-0.407125551	0.0117555	-0.214762369	0.1826155	-0.216164981	0.180021
EXOSC3	-0.349112501	0.0117638	-0.14076045	0.3026049	-0.189395683	0.16804
A2M	0.627981273	0.0117788	0.717332138	0.0040093	0.183755463	0.461187
REPIN1	0.366998758	0.0118037	0.083403079	0.5673483	0.351387517	0.015777
F2RL2	0.560835415	0.0118773	0.269986566	0.2262308	0.37242058	0.094955
DSP	-0.326361399	0.0119121	-0.174622074	0.17818	0.015502811	0.904845
LARS2	-0.306929096	0.0119189	-0.20422595	0.0916822	-0.280434972	0.021271
THAP5	-0.345369923	0.0119129	-0.026081053	0.8477797	-0.155107046	0.255131
LRRC27	-0.585389961	0.0119492	-0.294929828	0.1881749	-0.512033981	0.024268
1-Mar	-0.484710101	0.0119523	-0.211325962	0.26318	-0.388240597	0.041822
LIPT2	-0.584097473	0.0119839	-0.082861148	0.7018928	-0.392768594	0.079409
YLPM1	-0.343668525	0.0119803	-0.181265383	0.1833873	-0.269301469	0.04862
DNAH14	-0.280999141	0.0120158	0.096924485	0.3752343	-0.095948396	0.386355
SEC11C	-0.329171616	0.0120166	-0.193625913	0.135573	0.099170401	0.442429
MICU2	-0.206850146	0.0120305	-0.099958245	0.2174155	0.036250112	0.653809
SCNM1	-0.282568512	0.0120246	-0.277164817	0.0124078	-0.076825658	0.486518
RNF222	0.892095146	0.0120521	0.901317818	0.0095009	0.302103476	0.415694
DDX39B	0.215266133	0.0120792	0.207284872	0.0155172	0.221194848	0.009848
H6PD	0.262912417	0.0120802	0.049498638	0.6362702	-0.038926103	0.710555
PLK4	-0.387763965	0.0120866	-0.152263713	0.3218622	-0.073085851	0.634622
TPRKB	-0.290180635	0.0120847	-0.054104713	0.6302375	-0.078992511	0.484983
CSTF3	-0.192059223	0.0121262	-0.053497495	0.4735773	-0.078195786	0.299436
DEPDC1	-0.532503325	0.0121187	-0.226302442	0.2830406	-0.351587664	0.096193
HSPB11	-0.274533119	0.012132	-0.069565535	0.5134614	-0.133204818	0.214921
ZNF234	-0.389214869	0.0121336	-0.161967919	0.2922232	-0.469612486	0.002479
COX6B1	0.260305306	0.01215	-0.063064302	0.5436881	0.13816113	0.182935
NCBP2	-0.30758963	0.0121544	-0.02271602	0.8526237	-0.030075596	0.805864
GRTP1	-0.527021444	0.012165	-0.513404848	0.0134986	-0.348778823	0.091916
ADPGK	0.304735793	0.0122077	0.082893502	0.4948539	0.153805368	0.205809
TAF5	-0.342093492	0.0122264	-0.105136369	0.42724	-0.349950925	0.009681
NIF3L1	-0.286408905	0.0122459	-0.057481644	0.604655	-0.070087073	0.532092
NDC80	-0.382843931	0.0122698	0.140754654	0.3479431	-0.085036095	0.573216
ARHGEF11	0.32292036	0.0123062	-0.070667434	0.5839207	0.020597351	0.873265
METTL18	-0.348215004	0.012312	-0.093666729	0.4800025	0.004382596	0.973687
TRIM32	-0.252005029	0.0123248	-0.389769957	9.30E-05	-0.497542543	9.18E-07
EID1	-0.334916708	0.012356	0.079486832	0.55176	-0.159528144	0.233014
ITGB6	0.709189584	0.0123448	0.225264448	0.4269202	1.046068934	0.000222
SCNN1G	-1.588503943	0.0123624	-1.439990571	0.0214241	-0.922469795	0.135811
ZNF44	-0.335320589	0.0123584	-0.42598919	0.0013948	-0.361198664	0.006817
HDDC3	-0.577822431	0.0123728	-0.393970748	0.0821926	-0.419862169	0.065632
MTF2	-0.279795767	0.0123806	-0.306446465	0.0059926	-0.33299518	0.002912
SMIM22	0.456770459	0.0123879	-0.127722192	0.4881281	0.610711971	0.000766

TLDC1	0.251077316	0.0124033	-0.056080122	0.5776882	0.087129976	0.387463
HSPA4	-0.243908857	0.0124525	-0.026489674	0.7850931	-0.04394901	0.651919
TP53INP1	-0.288087901	0.0124575	-0.174288088	0.127053	-0.367222804	0.001417
EFHD2	0.516103759	0.0125358	0.214092458	0.3005996	0.759271959	0.000233
GK	0.753382104	0.0125306	1.013075823	0.0007456	0.473375642	0.116247
PIWIL4	-0.630266417	0.0125449	-0.827114897	0.0009888	-0.147966419	0.548286
ZNF808	-0.431698038	0.0125452	-0.693728523	5.74E-05	-0.470154295	0.006362
RUSC1	0.268772497	0.0125561	-0.109502021	0.3128567	0.076515046	0.478991
TYSND1	-0.478493603	0.0125619	-0.40365637	0.0324047	-0.45493923	0.016839
DTWD1	-0.287748707	0.012605	-0.108025285	0.340206	-0.37179337	0.001288
UBR2	-0.171658971	0.0126272	-0.202201242	0.003059	-0.185663336	0.006677
SLC22A23	0.51665456	0.0126748	0.295130041	0.1544914	0.731470256	0.000405
FAM73A	-0.288418893	0.0127021	-0.155718605	0.1741145	-0.09909308	0.387985
STOX1	-0.476611339	0.0126988	-0.347394233	0.0626138	-0.148110682	0.423754
KLHL8	-0.250376282	0.0127124	-0.10657462	0.2796657	-0.112844856	0.25403
MLXIPL	-0.917817916	0.0127441	-0.341600215	0.3417391	-0.73292888	0.046437
SSPN	0.856722381	0.0127398	0.847630807	0.0132116	0.672489788	0.049841
ZNF701	-0.306106036	0.0127565	-0.39252498	0.0013142	-0.369374704	0.002687
TMEM170B	-0.354776617	0.012793	-0.341109109	0.0157864	-0.231662291	0.101015
CLDN10	-2.453889662	0.0128007	-1.031525933	0.2696423	0.032409199	0.971984
NAT1	-0.442375978	0.0128219	-0.587171008	0.000918	-0.476434683	0.006595
RFC5	-0.351712366	0.0128352	-0.28920951	0.0389777	-0.048909078	0.727066
RCSD1	-1.045119063	0.0128624	-0.132577468	0.7307601	0.201045965	0.601935
LEKR1	1.349908251	0.012878	1.2638115	0.0193343	1.033370911	0.055493
KIAA0368	0.380594784	0.0129382	0.422976084	0.0057172	0.5560396	0.00028
MIER3	-0.291855262	0.0129329	-0.175934616	0.1326071	-0.322824367	0.005947
TCHP	-0.403820986	0.0129387	-0.077101744	0.6289861	-0.250590882	0.121325
TB-50L17.1	1.20361125	0.0129468	0.844643909	0.0832795	0.876004865	0.0736
ANKRD30B	0.598506728	0.0129854	0.880828502	0.0001854	0.369225251	0.126397
DDX27	0.799752283	0.0130282	0.693258304	0.0303641	0.499690147	0.121624
DGKE	-0.500944656	0.0130243	-0.070128746	0.7214593	-0.139017653	0.480883
FAM98A	0.285547246	0.0130078	0.020644224	0.8576268	0.173263162	0.131837
LSM12	0.263694706	0.0129899	-0.023549311	0.8245973	0.162511334	0.125622
MSH6	-0.315851038	0.0130398	-0.182536179	0.1504881	-0.221079292	0.081722
NCK1	0.385554264	0.0130356	0.372834471	0.0161991	0.572572924	0.000215
PHF5A	-0.266568539	0.0130311	-0.072107547	0.493729	-0.067854711	0.52079
TRIB2	0.638508312	0.0130145	0.541555434	0.0351212	0.550648493	0.032192
RWDD4	-0.304612142	0.013058	0.049727033	0.6800093	-0.058176013	0.630986
FAM46A	-0.452709305	0.0130871	-0.829418952	5.49E-06	-1.013234752	2.87E-08
TOMM7	-0.280434635	0.0130871	-0.188847026	0.0915071	-0.118694822	0.289226
C9orf114	-0.378372297	0.0131115	-0.333390926	0.0273686	-0.331396908	0.029554
HIST4H4	-0.413355253	0.0131529	-0.301646842	0.0690847	-0.189826101	0.25244
IFNAR1	0.224988298	0.0131728	0.104468055	0.2489889	0.457414587	4.01E-07
CHD4	0.258874575	0.0132094	0.159357494	0.1269379	0.164059276	0.116216
P11-1023L1	-0.795447815	0.0132247	-0.087577357	0.7755374	-0.598236047	0.057615
TIPARP	0.54258038	0.0132329	0.299091223	0.1721386	0.633464667	0.003801
CMAS	-0.27692471	0.0133149	0.001172202	0.9915544	-0.00792129	0.942945

CSNK2B	0.271379609	0.013287	0.225682072	0.0390294	0.185531355	0.090271
P11-274B21	0.47661307	0.0133036	-0.063633607	0.7438317	-0.109947126	0.574706
TMEM206	-0.696483114	0.0132981	-0.387469322	0.1604482	-0.67502762	0.015745
VSIG1	0.658820967	0.0133101	0.093911689	0.7242213	0.99545648	0.000184
PPP1R7	-0.255264777	0.0133694	-0.285926743	0.0052751	-0.194478764	0.057524
ZNF488	-0.784671345	0.0133944	-1.075791364	0.0007142	-1.817091773	4.43E-08
CDK1	-0.389461267	0.0134818	-0.172636388	0.2715796	-0.225527098	0.151294
ABHD16A	0.26133628	0.013491	0.005180005	0.9609953	0.185318038	0.079557
TMEM248	0.236745455	0.0135048	0.148082525	0.1214915	0.299610553	0.001737
ADGB	1.230595255	0.0135628	0.954956842	0.0487142	0.484033538	0.323033
PYURF	-0.254983754	0.0136545	-0.013838266	0.8911663	-0.127185951	0.212614
OSBPL1A	-0.252262823	0.0136683	-0.113932323	0.2592638	0.051563777	0.609466
LEPREL2	0.80672223	0.013679	-0.014770151	0.9641717	-0.343876773	0.300231
NRDE2	-0.381816648	0.0137051	-0.325354898	0.0348587	-0.31851793	0.039791
ACTR3B	-0.53107191	0.013752	0.208422667	0.3242436	0.343909647	0.104959
EMD	0.333236809	0.0137328	0.275186786	0.0397112	0.395980293	0.003011
ISY1	-0.424354648	0.0137225	-0.227306973	0.1819015	-0.287891889	0.091656
LYPD2	1.1271554	0.0137428	0.09643989	0.834134	1.527731365	0.00081
MOBP	0.957918416	0.0137545	0.811925751	0.0286727	0.536367267	0.15923
PDLIM7	0.560569556	0.0137581	0.348308691	0.1257825	0.141940474	0.533383
TMEM134	0.520248236	0.0137634	-0.157401933	0.4584215	0.262501485	0.216196
SC5D	0.278753617	0.0137968	0.171247447	0.1296693	0.316632853	0.005075
FST	0.723177577	0.0138067	0.368875917	0.2092576	-0.06968266	0.812908
MKNK1	0.320508735	0.0138416	0.103709228	0.4228901	0.239920467	0.063276
ADAM2	-1.006210504	0.0138494	-0.513750192	0.1723691	-0.690468424	0.075834
AP3M2	-0.371497755	0.0138583	-0.309641306	0.0390516	-0.435863156	0.003827
GPR116	0.888294698	0.0138733	0.478453546	0.1830097	1.027463599	0.004206
GPR135	-1.028365174	0.0139855	-0.288653889	0.4632758	-0.220092543	0.57503
R3HDM4	0.324383589	0.0139886	0.022415371	0.8652901	0.364217896	0.005627
ALCAM	0.469404429	0.01408	0.174940203	0.3601182	0.933478101	1.01E-06
U2AF2	0.217527475	0.0140926	-0.044847219	0.61227	-0.008980313	0.919214
NCK2	0.31548618	0.0141148	0.45523815	0.0003663	0.5220347	4.45E-05
MSL3P1	-0.523361608	0.0141251	-0.606047641	0.0038783	-0.536744572	0.010975
NCAPD3	-0.411189165	0.0141442	-0.193887123	0.2450026	-0.272076006	0.102728
ACSF3	-0.345387525	0.0141805	-0.213789411	0.1264511	-0.410688529	0.003659
STAG3L5P	-0.416741913	0.0141829	-0.17545495	0.2913439	-0.293115716	0.081472
ZNF595	-0.374567229	0.0141764	0.00254518	0.9865193	-0.276095217	0.069061
OTUD5	0.308828656	0.0141925	0.172045836	0.1726348	0.229580397	0.068739
RAB11FIP2	-0.480901695	0.0142086	-0.522141735	0.0075845	-0.246657067	0.206913
ALKBH8	-0.332660183	0.0142384	-0.030397138	0.8172514	-0.163665701	0.218828
AC010970.2	-1.245404445	0.0143085	-0.353087929	0.4863956	0.050264194	0.92104
IST1	0.186338215	0.014325	0.067416938	0.3755391	0.216177019	0.004454
PTHLH	1.731925406	0.014318	1.633415412	0.020721	2.740834224	9.59E-05
DEPDC7	-1.038381102	0.0143573	-0.814652482	0.0517923	-2.505947264	2.11E-08
C12orf45	-1.001049942	0.0143667	-0.687691819	0.0865458	-0.670224478	0.094934
GTPBP4	0.362241977	0.0143885	0.292736715	0.0477619	0.423852228	0.004154
YRDC	0.409019616	0.0144129	0.081383124	0.6271983	0.426506914	0.010416

THYN1	-0.287777708	0.0144535	-0.109871104	0.3401812	-0.206037026	0.076595
COL5A3	1.322265027	0.014465	0.943634393	0.0812159	0.404537734	0.457923
MRPS7	-0.227880835	0.0144727	-0.01267259	0.8898287	-0.047506419	0.605654
PPM1D	-0.227512347	0.0144848	-0.266819879	0.0037872	-0.239807536	0.009494
TXNIP	-0.500347792	0.0145502	-0.689781507	0.0007568	-0.686435636	0.000804
LRRC8D	-0.347644238	0.014575	0.157084593	0.2607773	-0.215721065	0.126895
PDE4A	0.811784229	0.0145874	0.199773046	0.5569746	0.251023251	0.452772
CLPX	-0.276567357	0.0146119	0.165541874	0.1410059	0.002405971	0.982954
ELMOD2	-0.284037902	0.0146535	-0.069806775	0.5454015	-0.074970931	0.517325
IER2	0.518636741	0.0147118	0.208419652	0.3271824	0.179732395	0.398627
ZNF106	-0.279918618	0.0147601	-0.083664796	0.4647235	-0.22582631	0.048861
FBXW11	0.236441436	0.0147722	0.090574556	0.349228	0.218723173	0.023686
SRPR	0.262492629	0.0147779	-0.184081807	0.0884567	0.111502933	0.300103
C1orf27	-0.233956757	0.014798	-0.064921825	0.4920593	-0.044920242	0.635597
MFF	-0.224139171	0.0147993	-0.013951061	0.8780671	-0.117754614	0.197485
SLC16A13	0.487663995	0.0148274	0.216902136	0.280494	0.07980332	0.697735
USF1	-0.249575988	0.0148251	-0.151322281	0.1347641	-0.189288226	0.063463
RNF11	0.259146546	0.0148618	0.118877371	0.2626327	0.374496161	0.000414
TUBGCP3	-0.395728917	0.0148602	-0.425783546	0.0084091	-0.534191993	0.000966
CPSF2	-0.250683459	0.0149795	0.083051547	0.4176668	0.016383479	0.873141
PLA2G10	-2.993158146	0.015003	-2.610354073	0.0291091	0.481169843	0.671737
RTN4	0.203851959	0.01504	0.21744869	0.0093365	0.223495724	0.007581
GLE1	-0.268598498	0.0150657	-0.112524812	0.3015426	-0.11868965	0.277324
MT-ATP6	0.643446938	0.0150513	-0.18523895	0.4841809	-0.169303478	0.522586
MZT1	-0.32374717	0.015067	-0.266487092	0.0421893	-0.229925193	0.080505
CDKN2AIP	-0.485653371	0.0150886	-0.636764448	0.0014392	-0.734070425	0.00025
PEX6	-0.306398139	0.015128	-0.273762405	0.0279959	-0.274864567	0.028013
TIMM21	-0.322089359	0.0152775	-0.313614593	0.0168216	-0.262848494	0.045271
CDC6	-0.483193811	0.0152943	-0.273029217	0.1690248	-0.23446344	0.237731
KIAA0355	0.348682214	0.0152927	0.317601647	0.0268798	0.309623441	0.031194
CYR61	0.742392119	0.0153146	0.016211649	0.958041	0.320319906	0.296927
GPATCH2	-0.24416394	0.0153482	-0.123599686	0.2146045	-0.081819541	0.412944
TMEM41A	-0.246693768	0.0153496	-0.208157176	0.0393961	-0.383046509	0.000163
OSBPL11	-0.179760167	0.0153668	-0.176126983	0.0157244	-0.321826506	1.44E-05
URI1	-0.343547648	0.015407	-0.017848302	0.8995702	-0.027446088	0.846142
COQ7	0.346273028	0.0154419	0.333066138	0.0188905	0.208458593	0.142533
ATL3	-0.187672301	0.0154869	-0.149700348	0.0517007	-0.048227495	0.530721
ZBTB22	0.249809576	0.015501	0.002146395	0.9834181	0.014983254	0.885252
TUBA3FP	0.662667166	0.0155329	0.549259925	0.0416492	0.671286581	0.013234
C19orf55	0.50694662	0.0155622	0.026906758	0.898298	0.039547107	0.852756
GCNT1	-0.644608439	0.0155932	-0.682874679	0.0104139	-0.253049196	0.342251
GMEB2	0.315684673	0.0155999	0.15770391	0.2256634	0.355761864	0.006101
GPRC5C	0.403932986	0.0155993	-0.161549764	0.3345331	0.285378183	0.087487
KLHDC2	-0.342274396	0.0155838	-0.223352204	0.1133709	-0.140075697	0.320621
PCNT	-0.362176012	0.0155884	-0.233709746	0.1182001	-0.136348297	0.362081
APH1A	0.243915695	0.0156718	-0.008387471	0.9337661	0.256371243	0.010922
C4orf27	-0.319379139	0.0156394	0.167685671	0.1861529	-0.282095272	0.03075

MFSD9	-0.411303502	0.0156694	-0.70062917	3.81E-05	-0.376637742	0.026552
NFYB	-0.300554242	0.0156747	0.006192107	0.9597856	-0.270335001	0.028831
PREPL	-0.283530052	0.0156538	-0.15477992	0.1851855	-0.349728192	0.002826
TRPC1	0.679225349	0.0156724	0.519244865	0.0625232	0.249079637	0.378433
TRUB1	-0.293316677	0.0156322	0.120599237	0.3153952	-0.012634163	0.916496
FBXO48	0.521030376	0.0157158	0.37798436	0.0776208	0.604246135	0.004416
SSBP1	-0.200877744	0.015711	0.071858157	0.3794072	0.171609222	0.036047
NSL1	-0.374556245	0.0157513	0.214823909	0.1603659	-0.090111142	0.558053
KCNJ13	-0.847873823	0.0159577	-0.050406789	0.8804351	-0.689096726	0.043606
MAP4K3	-0.233297331	0.0159643	0.038316346	0.6903323	-0.118301489	0.220692
UBB	0.183695456	0.0159724	0.066318644	0.3840644	0.322493321	2.28E-05
GUSBP1	-0.42704092	0.016018	-0.27813059	0.1102853	-0.41679519	0.017798
C8orf44	-0.841571559	0.0160322	0.396299728	0.2239135	0.141396155	0.673102
HNRNPU	0.179885974	0.0160356	0.117597771	0.1151866	0.111002695	0.137207
VANGL2	0.390429848	0.0160449	-0.244245224	0.1369322	-0.081592575	0.618019
PPM1N	-0.769700964	0.0160561	-0.085054771	0.776972	-0.300351691	0.326677
DHX32	-0.26087644	0.0160836	-0.20921183	0.0515513	-0.033127142	0.757307
DLGAP5	-0.468956528	0.0161015	0.050466586	0.7947275	-0.190862933	0.326024
ZNF302	-0.345129765	0.0161697	-0.186496082	0.1905242	-0.510920583	0.000374
ZNF354A	-0.371438746	0.0161695	-0.277782609	0.0680289	-0.362685779	0.017943
MPEG1	-0.862646223	0.0161781	-0.73331821	0.0326216	-1.133172048	0.001923
BTN2A2	0.50549212	0.0162039	0.505436711	0.015448	0.578316365	0.005713
PHLPP1	-0.481467469	0.0162601	-0.047013867	0.814356	-0.370385728	0.064291
SHC2	0.443371955	0.0163056	-0.131994773	0.475746	0.182130366	0.323887
DDX19B	0.280748481	0.016327	0.190864919	0.0998419	0.34485148	0.00291
HIST1H4C	-0.426962972	0.0163369	0.015895874	0.9286158	-0.134235934	0.449621
LLGL1	0.407230677	0.0164262	0.097434682	0.5661559	-0.053847595	0.75184
CLUAP1	-0.389307031	0.0164855	-0.312768483	0.0502826	-0.291885766	0.069518
RHPN2	0.455429538	0.0164974	-0.089577674	0.6376489	0.411331156	0.030286
C1orf86	0.58125243	0.016509	0.144933139	0.550067	0.451964545	0.062834
ARPC4	0.265186616	0.0165234	-0.070669672	0.5238039	0.15661543	0.156921
MBNL1	0.236660372	0.0165535	0.062504265	0.5263074	0.212217342	0.031392
FIZ1	0.431901666	0.0165723	0.164423358	0.3621051	0.149098452	0.409816
CAMLG	-0.324507348	0.0166136	-0.221335362	0.0991861	-0.324100168	0.016533
AKR1C2	-0.801102798	0.0166438	-0.256817973	0.4388841	0.258335555	0.434321
NUDT7	-0.860052535	0.0166616	0.145075614	0.6561602	0.245608607	0.450203
SEC61G	-0.278204134	0.0167435	-0.011701318	0.9189437	0.159928303	0.163805
URGCP	-0.326730197	0.0167657	-0.323388191	0.0177119	-0.448211459	0.001042
ZNF438	0.397630321	0.0167842	0.249407691	0.1313332	0.330544721	0.045262
ZFP64	-0.342685059	0.0168539	-0.341714648	0.0160454	-0.354314619	0.013004
9-Mar	-0.379749611	0.0168654	-0.341533495	0.0297193	-0.411082582	0.009034
MSN	0.435111998	0.0169063	0.43906634	0.0158422	0.08411118	0.644504
PTPRJ	0.521622316	0.0170139	0.46966067	0.0309844	0.377124101	0.084364
POU2F2	1.482538918	0.0170241	0.478390747	0.4457354	0.796439191	0.209435
RPL13P12	0.426447471	0.0170638	0.11060525	0.5363277	0.325045359	0.068972
MOSPD1	-0.31746804	0.0171545	-0.175151388	0.1813167	-0.377258255	0.004345
PLEC	0.508261394	0.0171534	0.623593009	0.003443	0.67822509	0.001466

PRPF18	-0.328195945	0.0171367	-0.148977075	0.2715023	0.100108739	0.457724
RPL9	-0.377257118	0.0171531	-0.430363617	0.0065421	-0.470881783	0.002929
SLC16A10	-0.897617301	0.0171272	0.395326356	0.2869913	-0.3952639	0.292596
PTEN	-0.299534801	0.0171873	-0.119181314	0.3427496	-0.035365325	0.778215
ZNF395	-0.384249727	0.0171793	-0.529040419	0.0010339	-0.478883748	0.002976
CCDC18	-0.45294852	0.01722	-0.142364453	0.4471759	-0.357593517	0.057942
SF3A2	0.2784167	0.0172232	-0.111853829	0.3439507	-0.059302685	0.616644
SLC25A40	-0.280940267	0.0173156	-0.213982441	0.0670919	-0.032403745	0.780384
TIAL1	-0.339375783	0.0173517	-0.340554275	0.0167123	-0.297038578	0.037067
SLC25A37	0.304896242	0.0174192	0.394286336	0.0019669	0.306643701	0.016434
CACFD1	0.402487813	0.0174325	-0.039184633	0.8185159	0.111124763	0.512403
ASB2	1.025726658	0.0174674	0.007253379	0.9867963	-0.225565238	0.617653
CRYGS	-1.044363807	0.0174765	-0.230651057	0.565136	-0.174081344	0.662248
KRCC1	-0.325020476	0.0174765	-0.054784279	0.6839228	0.094498962	0.481918
PUS7	-0.426489378	0.0174777	-0.116922013	0.5122261	-0.218469973	0.221014
HIST1H2AI	-0.499139072	0.0175941	-0.160807608	0.4434317	-0.302140135	0.150169
SLC9A2	-0.810628675	0.0176299	-0.763753049	0.025032	-0.080636957	0.812675
RABGAP1L	-0.310286249	0.0177754	-0.309228513	0.0180488	-0.155003443	0.235352
STX4	0.300060968	0.0178206	0.223465376	0.0754196	0.35421972	0.004851
GPR3	0.837461718	0.017846	0.352322156	0.3281691	-0.511890405	0.198996
ZNRD1	0.453413283	0.0178555	0.035671229	0.8525835	0.364275573	0.056553
NCOA3	0.21437339	0.0178663	0.053995565	0.5506092	0.163558257	0.070254
XBP1	0.585705175	0.0178788	0.27015167	0.2747419	0.633849458	0.010354
SET	-0.215579259	0.0178991	-0.064791666	0.4762053	-0.073968411	0.41624
IFITM3	0.329246563	0.0179722	0.078408181	0.5730983	0.205769131	0.13918
HIPK2	0.413808665	0.017986	0.369046073	0.0347389	0.344465894	0.048815
ACOT9	0.279694515	0.0180807	0.376345754	0.0012844	0.472037777	5.41E-05
CYTH4	0.942673125	0.0180773	1.126461353	0.0042644	1.129297537	0.004479
ARHGEF17	0.490809644	0.0181128	0.065567916	0.7521337	-0.330225714	0.113096
SPATA6	-0.428725866	0.0181358	0.083660677	0.6410933	-0.222449538	0.21542
TRNT1	-0.252230804	0.0181242	-0.015615814	0.8819324	0.036921333	0.726101
VPS13D	-1.016789409	0.0181419	-0.514608641	0.2311893	-0.050706125	0.905868
GPX2	0.491452125	0.0182021	0.38745072	0.062607	0.765015843	0.000235
AC004980.9	1.2055639	0.0182341	0.454181037	0.3837982	0.874203937	0.089142
FNBP1L	-0.269751312	0.0182362	-0.21271922	0.0623148	-0.138373477	0.225218
MFAP3L	-1.116068432	0.018229	-0.393665465	0.3959401	-0.990832811	0.035088
TBL1X	0.288798598	0.0183432	0.19493433	0.1103287	0.190396116	0.118694
TNFRSF12A	0.50850634	0.0183333	0.334019636	0.1209807	0.62759383	0.003542
UBXN11	0.331000903	0.0183414	-0.087281548	0.5352599	0.108060172	0.442461
SLC28A3	0.980138619	0.0183782	1.334307663	0.0013037	1.019990185	0.01413
EEFSEC	0.408090644	0.0183894	0.079988291	0.6460846	0.181937727	0.295646
DAPK3	0.322007175	0.0184146	0.212495669	0.1201594	0.207035845	0.130283
NLGN2	0.545788626	0.0184153	0.070381853	0.7625027	-0.347737942	0.138854
GABPA	-0.183680533	0.0184245	-0.141228609	0.0674837	-0.192864513	0.012897
EAF1	0.289392597	0.0184416	-0.014990103	0.9030216	0.217952246	0.076043
DEDD	0.250465161	0.0185302	0.026921567	0.8004583	-0.029165682	0.784315
UGDH	-0.239584542	0.0185945	-0.334383128	0.0010174	-0.141530543	0.16381

ZBTB7B	-0.38684107	0.018612	-0.568903011	0.0005396	-0.466767584	0.004523
APH1B	0.503325155	0.0186451	0.116023319	0.5876245	0.272459152	0.204275
PLCB2	0.984769201	0.0186468	0.400952464	0.3394587	0.381181329	0.364113
RNPS1	0.233470464	0.0186363	0.135378949	0.1720413	0.252084667	0.011007
MRPL43	-0.19560419	0.0186891	-0.135737908	0.0951551	-0.040796939	0.616737
PSMB4	0.19620046	0.0187214	0.046985089	0.5728775	0.079331178	0.341432
ATAD5	-0.365953291	0.0187628	-0.271595728	0.079797	-0.372640273	0.016515
DDX20	-0.289627966	0.018784	-0.260348673	0.0337524	-0.518403958	2.69E-05
RGP1	0.221425067	0.0187911	-0.104656773	0.2671018	0.02154144	0.819297
MIF	0.419453291	0.0188501	0.020839899	0.907182	0.346672929	0.052203
TP5J2-PTCI	-0.681027033	0.0189284	-0.943152327	0.0011594	-0.874383285	0.002619
MID2	0.542151489	0.0189206	0.124909405	0.5907451	0.454261332	0.048764
PIGO	0.347504179	0.0189487	0.114705603	0.4395863	0.199574139	0.177795
P11-575G13	-1.268440782	0.0189334	0.083196224	0.8609881	-0.88852017	0.082676
TRAF6	0.348220767	0.0189492	0.236626578	0.1104054	0.211349298	0.154054
ZKSCAN5	-0.242094218	0.0189374	-0.340217176	0.0008873	-0.387351779	0.000172
G2E3	-0.319697627	0.0189753	-0.169919799	0.2091645	-0.160290908	0.236905
ZNF160	-0.2684227	0.018976	-0.015647173	0.8900471	-0.027726882	0.806896
MOCOS	-0.74158783	0.0189973	-0.533994957	0.0894165	0.006628838	0.983108
RAP2C	0.643794994	0.0189925	0.106028459	0.6991531	0.655548742	0.016786
SERPINA4	0.371991955	0.0190819	0.131265024	0.4081653	0.500903111	0.001577
PLEKHA5	-0.305914697	0.0190988	-0.231654886	0.075324	-0.243640267	0.061577
RAB11B	0.445039228	0.0191113	0.000689453	0.9971303	-0.134842185	0.481921
JUN	0.598045341	0.0191255	0.404819471	0.1126788	1.13448917	8.50E-06
P11-777B9.	-2.20142774	0.0191949	-1.018546871	0.2776913	-0.807734885	0.389282
TSHZ1	-0.450470735	0.0191943	-0.165541178	0.3831779	-0.651358668	0.000732
TLR2	0.527748482	0.0192475	0.211723626	0.3492911	0.713562168	0.001356
DUSP3	0.207641734	0.0192618	0.036901578	0.6771096	0.270483066	0.002224
EIF5A	0.278646952	0.0192963	0.185502561	0.1190666	0.327152341	0.005969
WIPI1	0.293520698	0.0192886	0.324135628	0.0091997	0.529834583	2.04E-05
LAMTOR1	0.19660935	0.0193234	0.033044144	0.6928539	0.151822658	0.069747
POGK	-0.189251168	0.0193269	-0.241200038	0.0027598	-0.155348941	0.053963
DSCC1	-0.503920205	0.0193642	-0.217941449	0.3029615	-0.361665516	0.090108
DYNLL1	-0.206022322	0.0193728	-0.135150676	0.123278	-0.124436535	0.156306
INCENP	-0.435852771	0.0193737	-0.006968681	0.9700627	-0.293988449	0.114388
FKBP14	-0.403705592	0.0193933	-0.236694239	0.1690879	-0.158028481	0.359501
ZBTB40	-0.220536573	0.0193933	-0.346107996	0.000239	-0.406067713	1.75E-05
SPATA21	0.886622687	0.0194456	0.873321322	0.0221922	0.315792338	0.429283
ZNF860	-0.596912636	0.0194424	-0.519441186	0.0358941	-0.396686991	0.111857
AP005901.1	0.708327144	0.0195125	0.909344912	0.0025452	0.600191687	0.047602
HSDL1	-0.424641599	0.0195254	-0.568751555	0.0016785	-0.322128454	0.075186
TCN1	0.688031239	0.0195297	0.418731359	0.1552604	1.123677226	0.000135
CDKN2AIPN	-0.273095086	0.0196519	-0.107114542	0.3428577	-0.184661549	0.107261
HES6	-0.692924704	0.0196771	-0.802163099	0.006894	-0.305186863	0.291783
ZFAND2B	-0.410595556	0.0196796	-0.358150634	0.0402143	-0.277499082	0.112959
SPOP	-0.242816846	0.0197008	-0.1769698	0.087037	-0.211787443	0.040899
TBK1	0.240714668	0.0197368	0.42088618	3.93E-05	0.52924089	2.27E-07

TPM3P9	-0.371912957	0.0197782	-0.38634192	0.0143079	-0.243382713	0.123652
MANEAL	-0.774317313	0.0197885	-0.407478136	0.1976329	-0.127180359	0.686116
FOXA3	0.523525566	0.0198532	-0.162012186	0.4750616	0.447839938	0.046131
KAT2A	-0.410498581	0.019835	-0.594481789	0.000762	-0.618862295	0.000466
OR7E14P	-0.649751521	0.0198469	-0.407291848	0.1292507	-0.37360088	0.166002
FAM195A	-1.0196412	0.0198745	0.292877302	0.4863756	0.011984578	0.977567
DUOXA2	1.160630251	0.0198869	0.827470166	0.096953	1.337675254	0.007255
ETF1P2	0.802532541	0.0199	0.347839065	0.3183369	0.352618486	0.314274
RGN	-0.933058824	0.0199121	-0.056293413	0.8821959	-1.130592908	0.004546
SFR1	0.381268306	0.0200002	0.16184117	0.3243049	0.694217615	1.40E-05
BRF2	-0.376570452	0.0200265	-0.08468304	0.5930183	-0.629521563	0.000106
TOMM70A	-0.204944439	0.0200532	-0.152608226	0.0822631	-0.178084112	0.04279
SGOL2	-0.330583143	0.0201252	-0.185556679	0.1873253	-0.528415452	0.000196
SPRR3	1.771428253	0.0201278	1.124566831	0.1413349	2.845261343	0.000175
HPS3	-0.384108749	0.0201497	-0.09955861	0.5454045	-0.057577283	0.726442
RPP14	-0.223930487	0.0201423	-0.163740854	0.0870272	-0.310726651	0.00123
CNST	-0.263740204	0.0201794	-0.209829007	0.0621238	-0.263794842	0.019604
LPAR3	0.830278893	0.0201601	0.850285927	0.0156241	0.633287499	0.076397
TAP2	0.806802636	0.0201713	0.449174972	0.1975621	0.447128805	0.199236
FPGS	-0.341920411	0.0202207	-0.01896308	0.8955586	0.026324138	0.854916
SH3BGRL3	0.407278123	0.0202371	0.233025447	0.1840528	0.367621262	0.036061
WDTC1	0.293306197	0.020326	-0.077470848	0.5413229	-0.031873047	0.802357
PNISR	-0.256118039	0.0204156	-0.328956056	0.0028763	-0.482520657	1.27E-05
SP110	0.42859577	0.0204814	0.036274877	0.8442527	0.392419756	0.032409
ATP10B	0.587298534	0.0205128	0.738863771	0.0035174	0.29287079	0.248111
POLM	-0.377986175	0.0205877	-0.334999976	0.0355701	-0.345141361	0.031781
UNC13A	1.484630923	0.0206277	1.056910448	0.1003098	1.7331316	0.006926
ELMOD3	-0.360518358	0.0206467	-0.332013036	0.0300215	-0.123992086	0.421435
IVNS1ABP	0.185864447	0.0206479	0.06160376	0.4415652	0.051641502	0.519325
HDAC2	0.239108085	0.0206907	0.114404224	0.2673562	0.15429212	0.134824
ULBP3	-0.522598833	0.0207089	-0.227619389	0.3039882	-0.435040638	0.051427
ZNF629	-0.236038298	0.020712	-0.232686956	0.0217832	-0.321452613	0.001605
P11-735A19	-0.211509358	0.0207874	-0.172232526	0.0577347	-0.182115783	0.045419
RWDD3	-0.304345879	0.0208008	-0.216037112	0.0931488	-0.517597055	8.50E-05
PRRC1	-0.225022479	0.0208146	-0.414317987	2.04E-05	-0.245332117	0.011621
TRIM35	-0.531703742	0.0208298	-0.475808531	0.0384076	-0.629974893	0.006187
MXD1	0.648524877	0.0208972	0.60214583	0.0318835	1.364474803	1.12E-06
MED13	0.22010471	0.0209866	0.203106441	0.0330569	0.301264382	0.001572
DDX58	0.33082771	0.0210216	0.303190313	0.0331843	0.521172479	0.000237
TFEB	-0.46282999	0.0210443	-0.266815677	0.1706917	-0.164184724	0.404612
YEATS4	-0.270013089	0.0210634	0.007619411	0.9466646	0.151440503	0.182559
ZDHHC13	0.315656768	0.0210732	0.422370107	0.001921	0.464922845	0.00061
CYP2B7P	0.912502292	0.0211688	0.750212791	0.0581183	0.963320967	0.015656
RREB1	-0.370903619	0.0211765	-0.277721104	0.0836959	-0.26183866	0.103212
AC241952.1	-0.488285581	0.0211918	0.225822587	0.2653003	-0.527673501	0.012236
WARS	0.322017712	0.0211993	0.39202618	0.0049286	0.527953989	0.000152
ZDHHC8	0.399476704	0.0212448	-0.163554868	0.3508688	0.012443305	0.943296

CRBN	-0.233682422	0.0213264	-0.222651833	0.0266204	-0.094607395	0.34788
MPC2	-0.318801442	0.0213169	-0.367429245	0.0076825	-0.18475449	0.179739
PCDH12	1.239108985	0.0213472	0.738102731	0.1714503	0.585510823	0.281535
HELZ2	0.587809226	0.0214017	0.46962254	0.0664879	0.547582294	0.032038
PHAX	-0.264713287	0.0214657	-0.241528532	0.0351703	-0.290529624	0.011477
MRPS22	-0.2062386	0.0215034	-0.004192803	0.9620943	-0.019791995	0.822986
ARAP2	0.55721003	0.0215416	0.386216609	0.1110568	0.802607457	0.000909
FBL	0.304763948	0.0215591	0.014570751	0.9125916	0.005308762	0.968171
LYPLA2	0.226193554	0.0216296	0.036353047	0.711005	0.138916119	0.157183
PNO1	0.320825175	0.0216027	0.282755701	0.0422461	0.319121302	0.02207
SMURF1	0.231064289	0.0216252	0.317426023	0.0015657	0.442857538	1.00E-05
ZNF204P	-0.424059836	0.0216202	-0.417618403	0.0227516	-0.376720454	0.040216
CARD8	-0.334044674	0.0216563	-0.355415868	0.0136682	-0.451137408	0.002021
KIF11	-0.374119682	0.0216842	0.02898102	0.8582633	-0.124262116	0.444478
PPL	-0.714455647	0.0217078	-0.550942006	0.0763187	-0.244439269	0.431416
MMS19	-0.21515245	0.0217256	-0.237770262	0.0110852	-0.277575702	0.003027
MTCH1	0.20560426	0.0217608	0.138442153	0.1220882	0.218104623	0.014786
NCAPD2	-0.431077997	0.0217409	-0.169340243	0.3666866	-0.228386622	0.22392
TOB1	-0.486828317	0.0217556	-0.462731043	0.0287843	-0.656735521	0.001963
NAA16	-0.364044422	0.0217727	-0.349086612	0.0270644	-0.395992148	0.012288
UHRF2	-0.282239241	0.0217856	-0.076369267	0.5327744	-0.228451441	0.062493
THAP6	-0.297028001	0.0218702	-0.153529941	0.2221527	-0.370718325	0.003658
CNKSR3	0.406816	0.0219399	0.464983053	0.0086916	0.354737697	0.045558
RPS26	-0.302596353	0.0219391	-0.19042698	0.1481789	-0.137627453	0.29619
EBF2	2.284847032	0.0219636	0.828260074	0.4074701	2.975507394	0.002833
KATNA1	-0.364967021	0.0220103	0.034833002	0.8239206	-0.121391615	0.443522
TNRC6C	0.696154239	0.0220329	0.173960669	0.5671357	-0.037218635	0.902726
PRELID1P1	-0.570675531	0.0220602	-0.477138943	0.0511075	-0.532194544	0.029451
TYRO3	-0.310647191	0.0221216	-0.598094963	1.07E-05	-0.48774002	0.000333
SLC7A6OS	-0.346151067	0.0221516	-0.547908849	0.0002936	-0.691465251	5.40E-06
RPS6KB1	0.21920217	0.0221662	0.385913694	5.35E-05	0.400964595	2.68E-05
MGAT5	0.256194249	0.0222577	-0.130611817	0.2445898	0.305952886	0.006282
STRA6	0.928714035	0.0222422	0.43995014	0.2794062	0.746502345	0.06623
ZNF548	-0.289787361	0.0222607	-0.118034478	0.3443635	-0.324177228	0.01016
ANXA10	0.541232918	0.022321	-0.240797421	0.3096143	0.606621317	0.010429
TRMT44	0.313009079	0.0223363	-0.27297453	0.0493818	-0.006992108	0.959642
GPSM1	0.510567172	0.0223655	0.230642858	0.3025238	0.042646734	0.849291
TC-359D24	-0.741261695	0.0224061	-0.101225723	0.7465854	-0.85597803	0.008043
SHF	0.637121484	0.022412	0.421634182	0.129527	-0.031619547	0.910611
WDR66	-0.379086774	0.0224602	-0.532351746	0.0012265	-0.681561095	4.54E-05
TRIM71	-0.746727218	0.0224709	-0.745934212	0.022499	-1.462759603	8.24E-06
SRP19	-0.281310452	0.0224994	-0.009475882	0.9382996	0.038450371	0.753721
ORC1	-0.41117399	0.0225349	-0.382789747	0.0323857	-0.305955313	0.087556
FILIP1L	0.388435282	0.0225858	0.297772229	0.080294	0.224936764	0.186713
LCN9	-0.674655436	0.0225954	-1.052356459	0.0004032	-1.182322054	7.67E-05
ESYT3	-0.868491788	0.0226592	-0.347243704	0.347753	-1.03933333	0.005483
FNDC4	0.424916126	0.0226942	0.436011442	0.0185729	0.330007509	0.07639

SMOX	0.740612585	0.0227174	0.535124705	0.0997699	0.870379482	0.007371
PSD	0.712714092	0.0227635	0.66564974	0.032499	0.19296402	0.542001
SVIL	0.347746662	0.0227593	0.42644599	0.0051602	0.351246926	0.021317
RFX1	0.623300733	0.0228055	0.401890826	0.1416301	0.194384295	0.479315
SAFB	-0.233584203	0.0227969	-0.157651711	0.1219764	-0.153344805	0.133419
RLF	0.203634915	0.0228623	0.328168849	0.0002262	0.565585126	1.93E-10
HDHD2	-0.259058125	0.0230648	-0.137936834	0.2211741	-0.116061126	0.305083
VDR	0.530384972	0.023085	0.276763171	0.2355017	0.711885967	0.002105
HEATR3	-0.35521147	0.0231486	-0.102674707	0.5074262	-0.113528729	0.463416
EPHX1	0.568147654	0.023186	0.309353086	0.217449	0.311064679	0.215631
BSCL2	0.425855259	0.0232403	0.092069127	0.6248748	0.004072532	0.982778
OTUB1	0.177835545	0.0232336	-0.072998759	0.3523009	0.003237334	0.967109
SNX7	-0.22629172	0.0232352	-0.253181657	0.0103053	0.055862803	0.568125
ZNF718	-0.559769708	0.0232129	-0.184245181	0.4427434	-0.41026583	0.091577
MRS2	-0.301515438	0.0233048	-0.007904313	0.952174	-0.204541001	0.122198
TUBE1	-0.33702556	0.0233413	0.209156306	0.1484541	0.107054034	0.465184
RHOB	0.506857177	0.02338	0.239084678	0.2849116	0.658008341	0.003227
RAE1	-0.244363528	0.0233933	-0.174251522	0.1035097	-0.315140784	0.00347
DLEC1	-0.78916697	0.0234398	-0.584991707	0.0935456	-1.224631999	0.000624
FNDC3B	0.401936932	0.0234448	0.362975769	0.0406241	0.407846886	0.021444
MAPK7	0.451104651	0.0234181	0.478950912	0.0158007	0.427685936	0.031474
MTHFD2	0.44768189	0.0234357	0.409792666	0.0379533	0.812260804	3.85E-05
AKAP13	0.268653235	0.023499	-0.05187168	0.6620544	0.372361497	0.001671
KIAA1958	0.327072138	0.0234888	-0.154637263	0.2830211	-0.220883281	0.127869
PSD4	-0.360126565	0.0235845	-0.245145513	0.1215126	-0.144281188	0.363182
MITD1	-0.270299858	0.0237161	0.001955143	0.9865944	-0.036446988	0.755994
CEP290	-0.286551281	0.0238118	-0.099973732	0.4274256	-0.161531013	0.200943
PAXBP1	-0.227762842	0.0238135	-0.258654933	0.0099152	-0.353466021	0.000445
TNFAIP6	1.032932899	0.0239293	0.575251676	0.2108096	1.334469271	0.003408
CERS6	0.234612505	0.0240013	0.035484862	0.732725	0.373932415	0.000311
CCT6P1	-0.430406315	0.0240551	-0.192103959	0.2967252	-0.731074173	0.000147
PSRC1	-0.505390423	0.0240457	-0.302660699	0.1710895	-0.658235261	0.003352
CCDC117	-0.276734418	0.0240807	-0.121220959	0.3203535	-0.206930571	0.090275
RP1-152L7.!	-0.54700809	0.0241511	-0.383059742	0.1124331	-0.457978613	0.058554
ZNF337	-0.228080109	0.024154	-0.160974558	0.1018862	-0.293783624	0.00332
MMP25	1.136965408	0.0241945	0.937088171	0.0627645	0.643885593	0.199968
UBC	0.35094261	0.0241955	0.214686318	0.1679013	0.363009445	0.019714
ARF4	0.235515215	0.024257	-0.018322511	0.8608266	0.286722827	0.006038
EFCAB2	-0.385551184	0.0242554	-0.160174045	0.3326262	-0.322505179	0.055312
FAM129A	0.643759919	0.0242891	0.271974504	0.3431177	1.145355995	5.78E-05
NRBF2	0.227342156	0.0242569	-0.06384989	0.527599	0.119438526	0.236031
ORC2	-0.278186824	0.0242867	-0.07095938	0.5626495	-0.146329592	0.23342
PPP1CC	-0.17130455	0.0242809	-0.082332199	0.277312	-0.198268543	0.008984
SQLE	-0.245432306	0.0242939	-0.006784707	0.9503122	0.103411984	0.342122
MORN2	-0.374724843	0.0243654	0.006010856	0.9698329	-0.060608505	0.706062
NUP37	-0.253245734	0.0245106	-0.043297063	0.6939476	-0.152288247	0.16991
SERPINI1	-0.800028928	0.0245073	-0.504411051	0.1510123	-0.58586954	0.09619

USP53	0.38745617	0.0245903	0.34606771	0.0446555	0.677439762	8.32E-05
CAD	-0.319924105	0.0246417	-0.079103237	0.5765058	-0.187880594	0.186491
DNMBP	-0.249492004	0.0246598	-0.351090025	0.001507	-0.298514651	0.007069
HSF1	0.329888542	0.0246457	-0.051052231	0.7285379	0.134458457	0.3596
KNSTRN	-0.340245054	0.0246545	0.037709807	0.8000666	-0.108243284	0.470046
VGLL3	0.901159076	0.0246346	0.542666025	0.1757615	0.967192927	0.015784
SYF2	-0.245557991	0.0246955	-0.101482156	0.3466695	-0.092694246	0.390943
ZNF75A	-0.313004466	0.0246846	-0.290887689	0.0356734	-0.190186556	0.168483
FKBP11	0.450683277	0.0247101	0.112860136	0.5724194	0.55628664	0.005345
C19orf52	-0.427764186	0.0247619	-0.506501322	0.0070869	-0.514666526	0.006433
DDB1	0.159448185	0.0247751	0.170262698	0.0163345	0.188107508	0.008002
MT-ATP8	0.751311143	0.0247999	0.021208342	0.9495929	-0.06174678	0.854117
PCDHGB4	0.593427016	0.0247962	0.557529964	0.0346328	0.352293923	0.183114
TMEM74B	0.782364659	0.0248427	0.080471868	0.8205055	-0.249281678	0.492335
DECR2	-0.672970619	0.0249438	-0.230479619	0.433497	-0.413132913	0.165686
SPIN3	0.436413615	0.0249912	0.146919695	0.4515219	-0.035931526	0.857013
NAA50	0.337323994	0.0250581	0.301675536	0.0445302	0.382567153	0.010932
LRCH1	0.250681658	0.0251211	0.119070473	0.2845302	0.088872142	0.426165
APOL1	0.907955894	0.0251344	1.083521894	0.0072126	1.927198494	1.56E-06
SCML4	-0.862290344	0.0251542	-1.01347196	0.0080131	-1.503973602	0.000157
EIF4G2	0.338785163	0.0251979	0.463262893	0.0022061	0.380253734	0.01199
HS2ST1	-0.276222997	0.0252583	-0.099673956	0.4179304	-0.144754713	0.240243
RAD54L2	0.211439348	0.0252568	0.070812967	0.4526168	0.043242518	0.647502
CTSE	0.44830057	0.0254127	0.239320678	0.2328006	0.926066373	3.88E-06
DHODH	-0.360782292	0.0254309	-0.077076435	0.623686	-0.422405292	0.008179
ADAM11	1.204622458	0.0254451	0.281025688	0.6062613	-0.179428704	0.74476
ANKS4B	-0.86677357	0.0255225	-0.688326912	0.0755369	-1.500823233	0.000116
NR1H2	0.336301987	0.0255158	0.016053929	0.9152811	0.461757661	0.002022
NBR2	1.216630059	0.025536	0.502650402	0.3526002	0.657190243	0.22651
SPAG9	0.307735784	0.0255617	0.236568773	0.0854055	0.232940976	0.090389
RBMXL1	-0.233158205	0.0255838	-0.249934849	0.0159286	-0.143073944	0.167507
SMPD1	0.426382069	0.0256264	0.239840167	0.2084491	0.052329116	0.784598
SPATA2	0.341680035	0.0256668	-0.205558267	0.1889894	-0.195750695	0.214107
ZBTB9	-0.322261057	0.025721	-0.574405965	7.11E-05	-0.637832412	1.23E-05
EEF1A1P1	-0.611453725	0.0257606	-0.282992024	0.2787015	-0.004448005	0.986242
B4GALT7	0.281615526	0.0257787	-0.012260015	0.9222888	0.189224904	0.134226
SPEG	0.696847733	0.0258002	0.390955454	0.2109308	-0.230799629	0.465621
ASAH2B	-0.416983556	0.0258275	-0.307948964	0.0953126	-0.152122063	0.410468
CEP70	-0.241916449	0.0258528	0.021709483	0.8383616	-0.03355344	0.754367
OXSM	-0.490953775	0.0258884	-0.162811034	0.4402904	-0.129694128	0.539224
ZNF554	-0.325719341	0.0258895	-0.112820243	0.4305493	-0.176118255	0.223022
PPP2R1B	0.26823186	0.0259135	0.047634934	0.692117	0.123636273	0.304718
CTGF	0.754524391	0.0259784	0.089044923	0.792956	0.134685857	0.691407
B3GALT2	-0.762410289	0.0260383	-0.382479114	0.2593042	-0.890656774	0.009301
CDC37	0.237845458	0.0261038	0.131167579	0.2194011	0.302231954	0.004616
SFN	-0.483295496	0.0261289	-0.502287598	0.0201123	0.029411433	0.89081
WDR60	-0.471393584	0.0261427	-0.308426163	0.1450616	-0.401973086	0.057977

AC243772.2	0.452012846	0.0261591	0.423523662	0.0347902	-0.032982126	0.874259
DDX3X	0.438738783	0.0262047	0.448206064	0.0230483	0.654530585	0.0009
HSPE1P3	-1.516515334	0.0262279	0.581486239	0.3755033	0.153954473	0.815287
RQCD1	0.201626608	0.0262423	0.216419996	0.0166712	0.19851077	0.028236
RP11-762I7.	0.784439182	0.0262617	0.297964329	0.4007703	-0.137532158	0.699679
TPMT	-0.259749301	0.026325	0.017395127	0.8794887	-0.096413615	0.40403
TRIM65	-0.378329617	0.0263172	-0.344825292	0.039991	-0.494809306	0.003372
EMC10	0.224023248	0.026343	0.058119483	0.5638955	0.220559012	0.028467
CD101	-0.933764214	0.0264042	-0.070391634	0.8623722	-0.498753669	0.232725
MRPS26	-0.339063421	0.0264793	-0.420087274	0.0056695	-0.355483937	0.019385
XPA	-0.354898153	0.0264736	-0.123076522	0.4360625	-0.362538854	0.02245
TNFRSF14	0.802344582	0.0265288	-0.012199709	0.9741951	0.227515238	0.542057
KCNE1	0.794728504	0.0265988	0.620100666	0.0847258	0.663834783	0.064426
PPAT	-0.327851134	0.0265975	-0.18357641	0.2112733	-0.125823761	0.391839
ARHGAP18	-0.285514561	0.0267689	-0.433739267	0.0007578	-0.086339655	0.501654
CHST1	0.924758861	0.0267734	0.617699439	0.1413624	-0.457855471	0.296696
EEF1GP1	0.297135011	0.026702	0.157851572	0.2373279	0.342239659	0.010163
ERBB3	-0.329992539	0.0266888	-0.420582894	0.0047213	-0.337625523	0.023301
FAM174A	-0.378007292	0.0267172	-0.128536966	0.4407456	0.050072787	0.760843
ISG20	0.585516081	0.0267297	0.170284293	0.5202766	0.799399741	0.002418
MCTP2	-0.522179835	0.0267285	-0.24927313	0.2871501	-0.299050937	0.202788
SEZ6L2	0.978048746	0.0267133	0.528023968	0.2319304	0.481216298	0.276117
STRBP	-0.276488959	0.026739	-0.146519716	0.2369382	-0.092757221	0.454743
TPX2	-0.38550881	0.0267727	-0.404167954	0.0201241	-0.510697308	0.003343
AFM	-1.355400414	0.0269505	-1.49215764	0.0156372	-1.058362295	0.081667
TB-134H23	1.056634535	0.0270072	0.278941069	0.5531471	0.380236331	0.418764
EFNA4	-0.397354249	0.0269693	-0.367216438	0.036135	-0.502049732	0.005187
GATA4	-0.678833283	0.0270188	-1.138695674	0.0002165	-1.009315483	0.001018
IFT74	-0.30548953	0.0269189	-0.048188352	0.722773	-0.13033049	0.339833
KDM1A	-0.163762043	0.0269236	-0.027750683	0.7061765	-0.027834764	0.705737
KIF14	-0.901973019	0.0269116	-0.382602378	0.3434994	-0.26457011	0.512358
LMAN2L	-0.223697273	0.0269915	-0.33582161	0.0008509	-0.268900522	0.007646
LYSMD3	-0.359992061	0.0269483	-0.291114632	0.0728248	-0.274196605	0.091217
MCEE	-0.444437084	0.0269197	-0.512204576	0.0102096	-0.151375931	0.446007
MEOX1	1.698311037	0.0268382	1.040906502	0.1753675	2.050260692	0.007459
NDUFAF6	-0.350420693	0.0269624	-0.059871361	0.6949087	-0.242484664	0.119995
NUF2	-0.405715749	0.0269069	0.18328772	0.3073839	-0.16995796	0.348529
RHOQP1	-0.875421946	0.0270143	0.320953999	0.3594203	-0.850096894	0.028682
RPS17L	-0.359276816	0.0269619	-0.024982151	0.8776714	0.10217199	0.529006
SH3GL1	0.236706546	0.0269904	-0.00525888	0.960821	0.072599549	0.497843
STARD3NL	0.172054262	0.0270206	0.163384411	0.0333888	0.274693498	0.000354
TMEM245	-0.195881062	0.0269147	-0.351388103	7.13E-05	-0.225006735	0.010936
UBALD1	0.46165248	0.0270283	-0.212606216	0.3160773	-0.06745343	0.750276
EIF1	0.381779668	0.0271107	-0.048062905	0.7809769	0.204785177	0.23586
ADNP	-0.34679237	0.0271409	-0.302870475	0.0533161	-0.411441171	0.008718
LGR4	-0.26557916	0.0271572	-0.336512241	0.0050857	-0.186333486	0.120754
PRPF8	-0.364568321	0.0271622	0.054397377	0.7415308	-0.0796441	0.629167

TXNL4B	-0.350205167	0.0271359	-0.296839163	0.0574784	-0.360942343	0.021961
GLP2R	0.742777577	0.0271901	-0.043543597	0.8976692	0.371562076	0.270364
KBTBD3	-0.642959623	0.027267	-0.852866073	0.0031032	-0.975492808	0.000805
ACN9	-0.390546821	0.0273049	-0.244849613	0.1599134	-0.180422244	0.302355
CCDC134	-0.37599814	0.0273156	-0.24359408	0.141618	-0.361570635	0.032297
TNNC1	-0.676516236	0.0273027	-0.184991448	0.5339159	-0.492839302	0.102606
JMJD6	0.308204091	0.0273475	0.118787439	0.3930268	0.422967767	0.002298
TD-2561J22	-0.487940415	0.0274137	-0.577622456	0.0084636	-0.710167207	0.001403
SPTSSB	-0.904066637	0.0274241	-0.984797808	0.0163921	-0.057153332	0.887704
C14orf142	-0.38269604	0.0274457	-0.206523448	0.2088569	-0.144278374	0.38212
BFAR	-0.288438163	0.0274731	-0.186264103	0.1532657	-0.081442693	0.53242
SF3B14	-0.1985233	0.0274883	0.122181944	0.1673097	0.042365935	0.633734
BAIAP2L2	0.812751677	0.0275333	0.870325017	0.0178776	-0.068856232	0.853352
RP11-438J1.	0.51164207	0.0275507	0.294542208	0.2042775	0.837495186	0.000259
RC3H1	0.311299749	0.0275723	0.158643748	0.2611692	0.117890052	0.404061
U2SURP	-0.216851975	0.0275973	-0.099406793	0.311737	-0.108174367	0.271239
APLP1	0.664491239	0.0276282	0.213904416	0.479432	0.479543901	0.112674
FAM53B	-0.318771408	0.0276222	-0.287830209	0.0455591	-0.385592448	0.007621
GPR50	1.41110208	0.0276641	0.945912356	0.1405069	0.364510852	0.572405
STX8	-0.234911169	0.02767	-0.284608268	0.0066291	-0.151961552	0.148115
MCAT	-0.310090058	0.0277481	-0.290970471	0.0356147	-0.169471176	0.221105
PSMC5	-0.222618636	0.0277614	0.019816119	0.8423959	-0.071028506	0.478704
FAS	0.585235471	0.0277929	0.62299302	0.0188323	0.226517855	0.394969
CDC42BPB	0.189479632	0.0278087	-0.011910022	0.8898484	0.067112809	0.435564
CDKN2B	0.762973838	0.0278247	0.625779965	0.0710306	1.094368591	0.001581
USP11	0.324761702	0.0278335	0.051228693	0.728765	0.161985407	0.272645
CD59	0.364208282	0.0279109	0.231986243	0.1611801	0.470758025	0.004459
TNFRSF21	0.275085337	0.027908	-0.007183387	0.9542287	0.151268873	0.226609
TUBA1C	0.341675672	0.027905	0.362173477	0.0197428	0.58425803	0.000169
AP001885.1	-0.794186173	0.0280073	0.290341479	0.3489883	-0.436691147	0.195842
FAM178A	-0.320624848	0.0280002	-0.006363029	0.9649648	-0.220921005	0.128656
FNIP1	-0.172462336	0.0279831	-0.094598394	0.2229855	-0.046694603	0.548944
PRUNE2	-0.690526047	0.0280054	-0.496514995	0.112105	-0.595533226	0.06149
SLAIN2	0.390687693	0.0280239	0.227745178	0.2000371	0.18459954	0.299022
C6orf47	-0.229789587	0.0280631	-0.182335998	0.0768818	-0.125984274	0.222508
SERAC1	-0.275221059	0.02807	-0.114504758	0.3538381	-0.084928292	0.491489
NRIP1	0.180161635	0.0282303	-0.088937555	0.2787847	0.006139389	0.940397
ZBTB46	0.72200242	0.0282461	0.690344224	0.0355517	0.307150288	0.350916
FHDC1	0.487292915	0.0282636	-0.241358548	0.2804471	0.405804813	0.067533
ASPM	-0.408436587	0.0283113	0.10706838	0.5643097	-0.128691275	0.488843
FAM129C	-0.374063885	0.0284053	0.047988521	0.7716542	-0.198193145	0.237649
USP30	-0.48865175	0.0284285	-0.376651618	0.0899571	-0.649355895	0.003686
CDHR3	-0.754953035	0.0284469	-0.377179896	0.2585754	-0.145362599	0.663102
TD-2521M24	-0.845151991	0.0284548	-0.352713955	0.3287845	-0.388424565	0.287142
RAB4A	-0.274276817	0.0284971	-0.160989683	0.1956442	-0.177460892	0.155731
PTPRC	-0.631483987	0.0286815	-0.020146237	0.9415267	-0.350232154	0.21248
STX3	-0.433208826	0.0286785	-0.467330839	0.0180532	-0.138556641	0.482973

EMILIN3	1.042154132	0.0286991	0.750284199	0.1157395	0.643294411	0.179813
SAPCD2	-0.516593695	0.028723	-0.398127129	0.0897777	-0.302937411	0.196738
PI4KB	0.250216929	0.0287549	-0.142439525	0.21442	0.135862663	0.235736
RAB7A	0.403225434	0.028763	0.500604082	0.0066218	0.565337928	0.002162
TTLL4	0.210293991	0.0287645	-0.020204076	0.8337807	0.076186361	0.429716
ADAM17	0.234584952	0.0288322	0.098410701	0.3585477	0.32041882	0.002755
RPL36A	-0.396611833	0.0288821	-0.475907143	0.0086923	-0.101001161	0.57724
KDELC1	-0.414680834	0.0289148	-0.234739783	0.2108221	-0.399362097	0.034267
GOSR2	-0.204668145	0.0289323	0.006477144	0.9440261	0.038128576	0.680159
ATP2B4	0.284716885	0.0289662	-0.118722354	0.3626415	0.02673258	0.837568
MMP7	1.522311111	0.0289746	2.635274731	0.0001544	2.986506287	1.80E-05
USP20	-0.443953922	0.0289768	-0.222866896	0.2712181	-0.199385558	0.325334
FAM63B	-0.222994533	0.0290251	-0.125790666	0.2160801	-0.215267809	0.034855
C6orf211	-0.240353927	0.0290694	-0.12254572	0.2611714	-0.248927797	0.023244
DEK	-0.292969595	0.0290697	-0.011433776	0.9319962	-0.06928669	0.605189
ZNF83	-0.210494516	0.0291237	-0.027170344	0.7756415	0.05131667	0.59068
CCDC25	-0.268067937	0.0291886	-0.184626039	0.1316637	-0.068903544	0.57355
PIGM	-0.316863929	0.0292142	-0.280156061	0.0511661	-0.29299531	0.042293
SERPINB5	-1.824333267	0.0292542	-0.79196418	0.3176601	0.876469439	0.257233
ACAD11	-0.501430545	0.0292756	0.136579466	0.5478467	-0.327810158	0.153908
HJURP	-0.534250013	0.029288	-0.348013737	0.1531056	-0.460433353	0.059295
ABHD6	-0.392771882	0.0293797	-0.170948743	0.334789	-0.367550378	0.040153
SLC35D1	-0.386611873	0.029393	-0.047608679	0.7877143	-0.212438826	0.230262
PEAR1	0.930647461	0.0294204	0.263216569	0.5381247	0.612505989	0.150934
SETD4	-0.324059821	0.0294367	-0.479253071	0.0011646	-0.288065899	0.051392
SYTL1	-0.554101578	0.0294296	-0.327479876	0.1951222	-0.16903807	0.503137
SLFN12L	0.615951481	0.0295327	0.761047032	0.0060183	0.370683012	0.190541
CNPPD1	-0.267239459	0.0296205	-0.194984186	0.1062018	-0.075389625	0.531229
FGD3	-1.50373223	0.0296363	-1.229444286	0.0718974	-1.972793234	0.005394
ABRACL	-0.277639212	0.0298343	-0.025244877	0.8397736	0.113270862	0.363491
KRT5	-3.34410056	0.029831	-1.570075983	0.3017764	1.382237335	0.362043
LIMD1	0.228633267	0.0298531	-0.077690346	0.4576238	-0.271019217	0.010096
PDIK1L	-0.683474734	0.0298145	-0.979183617	0.0018777	-0.804561718	0.010442
TPD52L2	0.215932145	0.0298419	0.123791877	0.2118219	0.200740067	0.043026
TRIM8	0.31425408	0.0298377	0.024156431	0.8674031	0.062094357	0.667817
SP6	-0.916623391	0.0299404	-0.387831847	0.3456678	-1.490367553	0.000545
SLC13A3	0.62936715	0.029975	0.291846143	0.3186287	0.394600262	0.173548
C4orf46	-0.323235571	0.0300226	-0.152563288	0.3024984	-0.177654941	0.230994
KHDRBS1	0.191470707	0.0300298	0.151079745	0.0860695	0.160483689	0.068569
SPSB2	-0.439368078	0.0300227	-0.453443281	0.022865	-0.4329459	0.031678
BCAM	0.477750704	0.0300563	0.314564425	0.1524234	-0.327072946	0.139103
RFWD2	-0.2051336	0.030093	-0.067596998	0.4714069	0.149652284	0.110302
C11orf48	-0.249222095	0.0301155	-0.056833151	0.6155733	0.005764767	0.959464
ZNF638	-0.22591212	0.0301353	0.026463462	0.7992994	-0.173735803	0.095405
CDK11B	0.378270745	0.0302163	0.145731906	0.4045887	0.078775403	0.652563
SLC38A2	0.278144551	0.0302174	-0.033679107	0.7930367	0.146156142	0.254794
SNX2	-0.253703735	0.0302417	-0.221044249	0.05828	0.081211434	0.485631

NAIF1	-0.505830401	0.0302562	-0.729817821	0.0014707	-0.782835411	0.000748
RBM12B	-0.415577744	0.0302671	-0.09292543	0.6264791	-0.225494358	0.239084
NDUFA10	0.654310272	0.0302958	0.5716053	0.0574743	0.501322513	0.096359
FUT10	-0.313534386	0.0303291	-0.161665244	0.2593162	-0.135975362	0.344336
NSUN2	0.169394131	0.0303362	0.129401597	0.0963504	0.119043723	0.125962
ZNF532	0.320869808	0.0303329	0.673572176	5.15E-06	0.029583106	0.841874
PIP4K2A	-0.359506819	0.0304523	0.351538082	0.032997	-0.34072137	0.040253
RBM38	0.549308824	0.0304779	0.62435287	0.0135804	0.40367029	0.112452
NPC2	0.204004188	0.0306218	0.189077926	0.0447628	0.432347587	4.22E-06
TOX4	0.262155915	0.0306604	0.215676559	0.0750254	0.287589623	0.017605
VCL	0.235539591	0.0306651	0.099289616	0.362135	0.049601514	0.649065
TLN1	0.294767701	0.0306829	0.306730921	0.0244873	-0.092351257	0.498683
MTO1	0.295535336	0.0306981	0.275169351	0.0424007	0.107077088	0.431753
ACE	0.846179186	0.0308081	0.581727457	0.1262277	0.678647096	0.084464
AC010240.2	0.485147309	0.0308429	0.711960848	0.0010098	0.352193452	0.116886
RBL2	-0.168357938	0.0308342	-0.203863253	0.0087155	-0.28052522	0.000306
BNIP3P1	-1.936824543	0.0308723	0.066445711	0.9403333	-0.379464425	0.670213
HHIP	0.714280839	0.030908	0.719850626	0.0294801	0.421653392	0.20272
ZNF608	-0.332747367	0.0309361	-0.207099165	0.1781561	-0.374773904	0.015088
ZNF594	-0.409911858	0.0309574	-0.034427952	0.8525173	-0.253369539	0.175315
FBXO22	-0.302540539	0.03101	-0.249165961	0.0738354	-0.332518853	0.017463
PIGY	0.69647308	0.0310101	0.125632936	0.7007334	0.07451099	0.820815
STYK1	0.642935999	0.0309882	0.159732816	0.5939465	1.531829164	2.31E-07
MRPL2	-0.308107206	0.0310442	-0.140929422	0.3150303	-0.083076168	0.556001
UHRF1BP1	0.160752746	0.0310395	0.072079868	0.3300517	0.238807863	0.00122
NUP188	0.300166159	0.0311184	0.251533224	0.0706284	0.040050602	0.773722
ZNF598	0.416512241	0.0311311	0.042506817	0.8261462	0.246022684	0.202541
CCDC124	0.398864647	0.0311611	0.21005776	0.2555373	0.39746275	0.031145
EMC8	0.247737643	0.0311614	0.242022646	0.0334176	0.246040096	0.03075
ZNF570	-0.37674228	0.0312661	-0.303113997	0.0800846	-0.875059362	6.45E-07
RNASE10	0.675109463	0.0313978	0.0036566	0.9909646	-0.21131535	0.523495
C6orf163	-0.632751713	0.0314802	-0.004466927	0.9871481	-0.614752768	0.034214
SMTNL2	-1.225091735	0.0314674	-2.009837865	0.000415	-1.529920607	0.007346
C6orf62	0.234758763	0.0315952	-0.063304184	0.5619931	0.250921705	0.021302
GCSH	-0.324139559	0.0316599	-0.062701119	0.6747783	-0.109869652	0.463682
NUDT6	0.583669003	0.0316638	-0.037200438	0.8899386	0.148979323	0.585181
PLAG1	-0.316423945	0.0316139	-0.258995026	0.0766369	-0.355345503	0.01571
PLRG1	-0.193307063	0.0316652	-0.281451964	0.0016145	-0.198277758	0.026781
YBX3	0.305272008	0.0316349	0.290998974	0.040188	0.471038306	0.000892
KCNK5	-0.520712752	0.031682	-0.745400053	0.0020989	-0.35247863	0.144825
GFRA1	0.802577307	0.0317037	1.455976757	9.07E-05	1.27861542	0.000596
BAG2	-0.454775402	0.0317848	-0.018386888	0.9296927	-0.044764042	0.830331
EID2B	1.078304763	0.0317646	0.738150815	0.1299119	0.379130006	0.44727
KIF12	-0.61122548	0.0317795	-0.723648072	0.010946	-0.62406712	0.028426
NPC1	0.259942215	0.0317819	0.248688328	0.039752	0.395417042	0.001053
PIF1	-0.690302729	0.0318087	-0.062815047	0.841211	-0.233652096	0.462671
SIMC1	0.26550562	0.0318047	-0.055894718	0.6525654	0.068291596	0.58127

SPOCD1	0.783674972	0.031808	-0.016946979	0.9631206	0.296654781	0.417487
THAP11	-0.336421892	0.0318216	-0.51641993	0.0009558	-0.483564872	0.002037
SKA2	-0.314068411	0.0318985	0.144521292	0.320802	0.024626772	0.865935
ESD	-0.189354954	0.0319541	-0.088047097	0.3149599	-0.197193493	0.025016
KLLN	-0.418323556	0.0320287	-0.176619307	0.3360781	-0.513923642	0.008084
KTN1	-0.185940181	0.0320117	-0.122344743	0.157524	-0.005130055	0.952734
P11-835E18	-0.498230739	0.0320188	-0.105530416	0.6280938	-0.405815574	0.073115
MEGF8	-0.53026695	0.0321104	0.097582116	0.6930104	-0.482768528	0.051018
MRPS5	-0.202745134	0.032109	-0.103524714	0.2669825	-0.045246324	0.628662
PIK3R3	-0.360350408	0.0321145	-0.271256317	0.1065325	-0.59122325	0.000445
TMEM259	0.235176392	0.032176	0.062685954	0.568237	0.021183819	0.847164
HAPLN4	0.99367012	0.0322137	0.806613009	0.078798	-0.080135205	0.86636
ZNF93	-0.236498362	0.0322194	-0.133774127	0.2193508	-0.346099858	0.00172
ASXL1	-0.311733805	0.0322773	-0.093503657	0.5199845	-0.167384375	0.250094
DHX8	0.170228954	0.0322986	-0.00737497	0.9258802	0.126554723	0.109667
MND1	-0.494181536	0.0323918	0.245038687	0.2679871	-0.147463962	0.511275
EXPH5	-0.482580558	0.0324372	-0.521957475	0.02077	0.008787378	0.968823
ANTXR2	-0.313029314	0.0324662	0.007847175	0.9570997	-0.581041868	7.49E-05
POLR2H	-0.250047117	0.0325297	-0.064351943	0.5804963	0.055759491	0.631489
ATF7IP2	-0.345673563	0.0326114	-0.592341852	0.0002515	-0.278837779	0.084512
STXBP4	-0.366002836	0.0327048	0.176472553	0.2991692	-0.453443624	0.008179
BDKRB2	0.949437492	0.0327501	0.793128274	0.0743407	0.641529248	0.149634
CDK9	0.305962173	0.0327629	-0.264713983	0.0647852	-0.166803868	0.244462
TSG101	0.265888167	0.0327645	0.076357005	0.5395596	0.276503184	0.026093
ZNF578	-1.021077135	0.032767	-0.920233942	0.0524792	-0.181049954	0.701784
BZW2	0.239666092	0.0328555	0.087443306	0.4358549	0.320096274	0.004292
C1orf226	-0.567203146	0.0328833	-0.948331531	0.0004244	-0.696971186	0.009162
C3orf70	0.437482319	0.0328408	0.176052873	0.3907919	0.053882724	0.794304
TMEM161B	-0.23530858	0.0328749	-0.206457987	0.0570765	-0.127168249	0.241601
TSPYL4	0.261262928	0.0328855	-0.100914492	0.4123162	-0.221877864	0.074363
ZNF836	-0.346846391	0.0328953	-0.375099791	0.0190343	-0.252812527	0.113167
MRPL16	-0.229636223	0.0329167	-0.139758615	0.1885507	-0.182296738	0.087767
AC010620.1	-0.283211868	0.0330162	-0.324402163	0.0141918	-0.31114859	0.018869
GNAQ	0.225424095	0.0330267	0.098824401	0.3492913	0.475335867	6.56E-06
NLGN4X	0.536101485	0.0330309	0.076605167	0.7614295	0.183607137	0.466427
BBS7	-0.467236603	0.0330832	-0.175389499	0.420364	-0.227323188	0.299259
ARHGAP42	0.222611321	0.0331717	0.109565961	0.2945126	0.579528477	2.58E-08
BUB1	-0.390748463	0.0332019	-0.042048429	0.8177815	-0.273695975	0.134676
FAIM	-0.39307656	0.0331908	0.01288366	0.9429517	0.079234547	0.658695
PXDC1	0.293428214	0.0332669	0.328997374	0.0165221	0.430985596	0.001685
ATP5D	0.768805667	0.0333889	0.218839512	0.546901	0.673223206	0.063156
PITX2	-0.902551595	0.0334769	-0.891988398	0.0353719	-1.898833339	1.04E-05
SH3PXD2A	0.282685318	0.0334869	-0.104154871	0.4337333	-0.00125482	0.992479
C11orf95	-0.303709172	0.0335413	-0.453293883	0.0014938	-0.732826963	3.27E-07
MRPS10	-0.190264419	0.0335327	0.018937087	0.8289257	-0.018437537	0.834317
ABT1	-0.191554107	0.0337592	-0.238963351	0.0075748	-0.165925479	0.06397
GSTP1	0.209214838	0.0337446	-0.039818083	0.6861244	0.320615237	0.001129

PARVA	0.274588697	0.0337417	0.217205738	0.091959	0.060258168	0.641007
SIX5	0.645202166	0.0337567	0.333116587	0.2747242	0.422795387	0.165428
TD-2328D6	1.10791064	0.033805	0.162565241	0.7558222	0.665171526	0.202814
SLC39A8	-0.636591893	0.0337924	0.032314509	0.9118675	0.2560047	0.384537
ARNTL	-0.528072255	0.0338924	-0.632299586	0.0109671	-0.203006017	0.41206
PPIL4	-0.172895119	0.033902	0.028082794	0.7258714	0.034895326	0.664116
ART5	0.995234575	0.0339473	0.635046211	0.1780982	0.195610093	0.683274
PTPN2	0.223992419	0.0339353	0.35266092	0.0007607	0.554135219	1.17E-07
TMED9	0.236435242	0.0340252	-0.136124709	0.2230557	0.037328033	0.737948
TP53TG5	0.759504449	0.0340516	0.439750275	0.2201626	0.753616654	0.034287
VMA21	-0.180714669	0.0340854	-0.115818626	0.1715342	-0.23622241	0.005488
C15orf48	0.796017166	0.0341614	1.001363135	0.0073973	1.938462118	1.81E-07
SHROOM2	0.502382403	0.0341969	0.441615589	0.0614264	0.33253577	0.160929
TRIQK	-0.342218675	0.0341949	-0.290998	0.0710898	-0.030583189	0.849502
C21orf2	0.26398645	0.0342705	-0.044051544	0.7260671	0.29216885	0.019255
ZNF692	-0.358757946	0.0342819	-0.55363722	0.0010803	-0.488824724	0.003905
ENDOD1	-0.269702367	0.0343072	-0.117529761	0.3525805	-0.177722601	0.161038
SAE1	-0.218591717	0.0343197	-0.021922736	0.8311329	-0.053592043	0.602591
ITGB1BP2	-0.898902411	0.0344493	-0.265266562	0.4921621	-0.34412932	0.379563
C8orf48	-0.621441406	0.0345052	-0.437794205	0.123958	-0.695781302	0.017318
ZNF708	-0.236837549	0.0345417	-0.182980178	0.0997059	-0.413510757	0.000228
C4A	0.938877604	0.0346201	0.147237228	0.7430565	0.664951886	0.132978
CABIN1	-0.321573151	0.0346186	-0.413248784	0.0066042	-0.362687183	0.017169
KCNJ6	0.601356349	0.0345926	0.813462895	0.0036644	0.334316693	0.242666
PIM1	0.67118045	0.0346253	0.442028851	0.1641145	1.214105796	0.000125
ZNF304	-0.250224786	0.034578	-0.271945854	0.0202944	-0.344816037	0.00351
ZSCAN9	-0.348349542	0.0346487	-0.028301134	0.8598212	-0.108831039	0.500603
PPME1	0.271312377	0.0347087	-0.156858927	0.2244237	-0.093525756	0.469147
DUS4L	-0.375401418	0.0347701	-0.104688642	0.5403562	-0.062170145	0.717227
NOLC1	0.373079613	0.0347606	0.51425402	0.0035901	0.612933545	0.000518
GXYLT1	-0.258108936	0.0348174	-0.193365029	0.1127842	-0.361091785	0.003157
SEMA4B	0.470499095	0.0348332	0.454490474	0.0413886	0.764319036	0.000598
ZNF681	-0.402587511	0.0348369	-0.482803016	0.0107506	-0.438422231	0.020857
ZNF714	-0.682778309	0.0348074	-0.22718621	0.4593921	-0.694084901	0.029913
PRKRIP1	-0.303892685	0.0349084	-0.158516176	0.2664812	-0.230053983	0.109828
ZNF468	-0.382875121	0.0349022	-0.29458968	0.1041051	-0.303109364	0.094515
KIF3A	-0.351436791	0.0349299	-0.385392659	0.0197551	-0.436072753	0.008876
RAB8A	-0.312792931	0.0349657	-0.162898124	0.2711993	-0.137308276	0.353768
P11-460N11	0.854327055	0.0349934	1.247451298	0.0017933	0.698014415	0.08507
S1PR2	0.573325892	0.0352252	0.302338709	0.2669324	-0.024035046	0.92998
ZNF90	-0.380150748	0.0352213	-0.421573137	0.0184691	-0.375035243	0.035929
DNAL4	-0.309954478	0.0352555	-0.389459169	0.0075916	-0.271061459	0.062926
RBM7	0.264317265	0.0352525	0.097336438	0.4371755	0.276168551	0.027384
LGALS9	0.625734763	0.0352807	0.041293658	0.8899746	0.763139993	0.009988
WDR3	-0.326294349	0.0352969	0.005768486	0.970222	0.020364274	0.895248
PARD6B	0.309101261	0.0353218	0.205613818	0.1608556	0.425723122	0.003653
FZD6	0.353928669	0.0353788	0.191885853	0.2532719	0.583339399	0.000496

YWHAG	0.162390286	0.0353807	0.125010914	0.1047487	0.202881897	0.008478
EEF1A1P12	0.383692815	0.0355234	0.368321034	0.0408435	0.437052657	0.015403
RNF219	-0.294974359	0.035545	-0.271742454	0.0522324	-0.273616396	0.050827
VPS33A	-0.294238129	0.0355359	-0.089352365	0.5188319	-0.084975155	0.541658
ACTG1	0.300918597	0.035617	0.03920443	0.7842717	0.239205721	0.094822
HDGFRP3	-0.245999973	0.0356138	0.201829968	0.0828216	0.04645989	0.6904
LAMA5	-0.401736393	0.0355961	-0.571667285	0.0027991	-0.452694297	0.017918
MRPL51	-0.213972533	0.0355926	-0.021002606	0.8342828	-0.021714155	0.829211
BCKDHA	-0.874013509	0.0356996	-0.153430152	0.7033376	0.425757775	0.289565
HOXB9	-1.597640659	0.0357314	-1.236299411	0.10171	-3.522176185	1.02E-05
CBX2	-0.394194552	0.035796	-0.347917214	0.0619964	-0.488562637	0.009104
PSMG2	-0.176581637	0.0357955	0.027526106	0.736934	-0.02187172	0.790918
DZIP1L	0.439778483	0.0358109	0.237347504	0.2563258	0.18888942	0.367803
OLR1	0.742044742	0.0358863	0.517898542	0.1427964	0.471107275	0.182982
ECT2	0.334315768	0.0359327	0.377224791	0.0178281	0.500474166	0.001666
ALDH3A2	-0.365754283	0.0359821	-0.139133364	0.4237085	-0.327455385	0.059911
CTSV	-0.369192883	0.0359926	-0.192318912	0.2677371	-0.072991132	0.673947
P11-570P14	-1.199764802	0.0360069	0.086936173	0.8702315	-0.034163362	0.949157
P11-38O23	-0.934268326	0.0360742	-0.214562084	0.6037863	-1.088167124	0.014793
DDX26B	0.268971809	0.0362296	0.232367788	0.0698408	0.208937887	0.104534
FABP5P7	-0.471610773	0.0362123	-0.131206539	0.5579156	-0.011798561	0.957972
KCTD2	-0.299939271	0.0362517	-0.330959925	0.020588	-0.287267985	0.04465
MGRN1	0.358391283	0.0362444	0.077824485	0.6498559	0.182301408	0.287144
PURA	-0.265345423	0.036251	-0.564543907	7.11E-06	-0.500288967	7.41E-05
RAP1GDS1	-0.230429242	0.0361944	-0.197513682	0.0705757	-0.051262648	0.637806
ARF6	-0.194048934	0.0363259	-0.216766811	0.0189777	-0.111309202	0.228282
CNNM1	-0.601962969	0.0363266	-0.346158534	0.2242386	-0.389332197	0.172532
DNTTIP1	0.244221885	0.0363251	0.148060608	0.1996475	0.370175987	0.001263
PYGL	-0.319649334	0.0362936	0.077554163	0.6075223	-0.011717139	0.938298
TARSL2	-0.314017543	0.0362959	-0.156873162	0.2925462	-0.061985304	0.677286
P11-254B13	0.879636463	0.0363558	0.903980483	0.0291477	1.224039216	0.002838
ST20	-0.477119444	0.0363613	-0.251815966	0.2588305	-0.100540937	0.650598
MRPS31	-0.226678021	0.0363846	-0.112838237	0.2874551	-0.19723892	0.065553
ST8SIA3	-1.638451651	0.0364136	0.314670155	0.6829215	-2.381768312	0.002966
MRPL22	-0.260763213	0.0364282	-0.240763053	0.050658	-0.253776637	0.041129
ZBTB4	-0.270155369	0.0364493	-0.344360375	0.0073892	-0.328761505	0.010728
ACBD5	-0.245569064	0.0366302	-0.283191506	0.0156255	-0.153495927	0.189229
BBS12	-0.459596724	0.0366046	-0.174144851	0.4149177	-0.089013302	0.67961
GNB1	0.238495009	0.0366329	0.231399943	0.0424368	0.282158313	0.013362
C1QTNF6	0.291383155	0.0366501	-0.031700697	0.820777	-0.05361904	0.705607
B9D1	0.342939544	0.0367326	-0.0655151	0.693415	-0.145417077	0.386468
BSDC1	0.235138181	0.0367184	-0.044320618	0.6938391	0.108435119	0.334998
IQSEC3	0.857705136	0.0367263	-0.401223498	0.3441301	0.018975559	0.963175
ITM2A	1.439791426	0.036705	-0.183494234	0.7918441	2.433923516	0.000394
TTC7A	-0.380072646	0.0367238	-0.143346761	0.4245676	-0.165085265	0.35831
CCDC157	0.414195366	0.0367747	0.059822361	0.7626471	0.419952167	0.032298
HTR1B	-0.945986836	0.0368054	-0.991752149	0.0282018	-1.059460406	0.019319

GNAS	0.163926593	0.0368903	0.176272626	0.0247161	0.143349721	0.0679
ENTPD4	0.24431233	0.0369557	-0.112322747	0.3369979	0.126575596	0.27928
FAM86EP	-0.589471067	0.0369524	-0.384054994	0.161258	-0.781531072	0.005118
INTS12	-0.480556642	0.0370605	0.205794333	0.3545337	-0.180223726	0.423895
AC004381.6	0.566586431	0.0371138	0.732504584	0.0052996	0.641474541	0.015552
DECR1	-0.204011126	0.0371195	0.215307507	0.0252275	0.03758147	0.69746
RAB21	0.224880068	0.0371215	0.165374533	0.1241645	0.386771861	0.000322
VHL	0.2096988	0.0371778	0.15562706	0.1207138	0.211201776	0.035374
TACO1	-0.287788171	0.0371991	-0.186954483	0.166194	-0.452152337	0.001076
ETFA	-0.215751135	0.0372655	-0.051835333	0.6145203	-0.140397178	0.173296
GLYCTK	-0.425721693	0.0372467	-0.492451219	0.0155778	-0.490993732	0.015955
IRGM	0.57079102	0.0372669	0.145146278	0.598991	0.1044719	0.708471
KIAA1731	-0.226884928	0.0373386	-0.116251757	0.2826696	-0.146518759	0.177088
NOL11	-0.220070491	0.0373624	-0.112194608	0.2862803	-0.119083682	0.258006
RAC2	0.600810002	0.0373135	0.284366149	0.3253081	0.143557839	0.620667
STK36	0.356398561	0.0373312	0.126457993	0.4594984	-0.107472753	0.531753
TOE1	-0.438931039	0.0373542	-0.465314969	0.0270977	-0.615714042	0.003513
C19orf26	0.880707501	0.0373939	0.496912266	0.243985	0.043979953	0.919722
FAM208A	-0.198244358	0.037452	-0.249220378	0.008695	-0.213854969	0.024512
FXYD6	0.489578032	0.0374377	0.424828226	0.0709281	-0.120053903	0.611842
FBXL3	-0.187840738	0.0374687	-0.240789576	0.0073389	-0.06303126	0.48217
CAPG	-0.250544945	0.0374917	-0.191457721	0.1110518	0.191479902	0.109623
CLDN1	0.555014558	0.0375211	0.403701244	0.1302429	0.574911071	0.031158
CD70	-1.369876204	0.0375419	-1.520176037	0.0206784	-0.885033627	0.170556
PRRC2B	0.256338957	0.0375909	-0.076844095	0.5329472	-0.095357067	0.439656
CCDC102A	0.535716002	0.0376357	0.421974272	0.0985631	0.349125187	0.174352
CCDC12	-0.23759969	0.0377101	-0.367787687	0.0012316	-0.320811795	0.004934
PCF11	-0.180724427	0.0377231	-0.277275792	0.0013783	-0.155120405	0.07341
FXN	0.34616442	0.0377541	0.146600594	0.3745398	0.108392771	0.516074
RHEB	0.15992578	0.0377584	0.364201827	1.53E-06	0.420832466	2.91E-08
DDX18P1	-0.838893013	0.0377933	-0.594736972	0.1237887	-0.70180092	0.074346
EBF3	2.093786179	0.0378262	2.002791293	0.046788	3.15403857	0.001706
GLUL	-0.289978679	0.0378652	-0.30127334	0.0309008	-0.00189196	0.989182
COG6	-0.346273319	0.0379315	-0.392233587	0.0186253	-0.333559294	0.045311
SLC35B4	-0.289438774	0.0379245	0.012316152	0.9291796	0.044809768	0.747106
ZNF207	0.157646374	0.0379874	0.162371189	0.0322642	0.262748324	0.000533
ZNF669	-0.250592457	0.0380178	-0.265689377	0.0253647	-0.440730795	0.00028
ZNF592	0.180998982	0.0380403	-0.07387211	0.3974427	0.017666151	0.839788
HNRNPA2B1	0.224098066	0.0380773	-0.033250976	0.7583135	-0.071859704	0.506262
FAM105A	-0.815701105	0.038111	-0.441130472	0.2601172	-0.650721677	0.097171
GOLIM4	-0.354812257	0.0381666	0.122259047	0.4736588	-0.178267769	0.296914
MRPL4	0.283695059	0.0381875	0.112353555	0.410921	0.110146139	0.421551
NMUR2	0.706721556	0.038181	0.840921004	0.0129599	0.215655178	0.532379
SLMAP	0.262420571	0.0381885	0.297429264	0.0186116	0.416503923	0.000976
IFI6	0.626372529	0.0382384	1.096642843	0.0002579	1.136168997	0.000154
NEK4	-0.209292111	0.0382323	0.005455165	0.9564112	-0.020495965	0.837944
ADAMTS4	0.597162489	0.0383277	0.480328239	0.0931187	0.279244133	0.331448

HNRNPK	0.142099427	0.0383253	0.030203235	0.6595828	0.114635173	0.094501
IDH3A	0.242768452	0.0382943	0.317050833	0.0062482	0.451956527	9.48E-05
MNS1	-0.478703284	0.038326	-0.142363584	0.5267806	-0.283860445	0.208697
NOG	0.696079337	0.0382907	0.509539173	0.1286568	0.555570026	0.098211
ETV6	0.318611926	0.0383567	0.159714165	0.2992291	0.279468146	0.06909
SMAP2	-0.366691872	0.0383874	-0.569571175	0.0013073	-0.545445601	0.002078
RPL26L1	-0.240999867	0.0384071	-0.009333301	0.9336571	-0.068915605	0.543376
FASTKD3	-0.384412524	0.038491	-0.332081267	0.0718265	-0.549797219	0.003045
VARS2	0.369010937	0.0384974	0.069809555	0.6957601	-0.005090283	0.977329
MAFB	1.594579027	0.038589	0.011470608	0.9882645	1.021288588	0.18638
SMIM5	-0.569588499	0.0385866	-1.223918536	1.06E-05	-0.390728835	0.152148
TM4SF1	0.387114734	0.038598	0.317214346	0.0900387	0.737385235	8.10E-05
TMEFF2	1.740815759	0.0385734	1.154505477	0.1663032	1.573188021	0.058831
ZC3H11A	0.139059036	0.0385837	-0.071547647	0.2867805	-0.00572277	0.932133
ZNF367	-0.479124762	0.0385684	-0.47154443	0.040787	-0.357709008	0.12059
ZGLP1	0.894367085	0.0386411	1.367896874	0.0009607	0.555419329	0.200557
BAHD1	-0.260112085	0.0386575	-0.551694397	1.16E-05	-0.431908359	0.000598
BCL7A	-0.294197327	0.038687	-0.024117001	0.8607865	-0.375867135	0.008932
P11-139H15	-0.418450353	0.0387085	-0.14191705	0.4638046	-0.120109852	0.537578
ATG7	0.345372554	0.0387538	0.538337698	0.0012126	0.374053508	0.024533
PL17-C18orf	-0.333860112	0.0387622	-0.141693928	0.3784879	-0.042304862	0.792581
ZNF684	-0.5229873	0.0387428	-0.4442232	0.0730711	-0.267892688	0.282139
PBDC1	0.289537865	0.0388758	-0.05423729	0.6994431	0.337124697	0.015796
SMIM7	-0.173163686	0.0390553	-0.103165337	0.2152208	0.083087238	0.318549
UBR4	0.275316355	0.0390889	0.08117363	0.5427865	0.251419762	0.059539
MSANTD2	-0.295055946	0.0391851	-0.057609444	0.6817707	-0.080041999	0.570803
PRPF4B	-0.187299924	0.0391725	-0.259751913	0.0041693	-0.172717793	0.056803
PRRC2A	0.252947326	0.0391909	0.249657952	0.0417508	0.105433566	0.390282
KBTBD2	0.234136678	0.0392386	0.108305372	0.3391958	0.280456705	0.013164
RBBP5	-0.176162682	0.0392177	-0.160388994	0.0561978	-0.230401368	0.006673
RGMA	0.609899487	0.0392394	0.159169621	0.5908825	0.056558887	0.848707
AC000367.1	-0.60607375	0.0392576	0.233251606	0.3915189	0.150751441	0.58346
DCTN4	-0.180246716	0.0392926	-0.027093155	0.7556407	-0.281624698	0.001284
PXMP2	-0.381841522	0.0393268	-0.056500017	0.7551847	-0.196631807	0.280305
ABCA9	0.776471127	0.0393941	0.822454651	0.0279927	0.633194626	0.091128
HLA-E	0.287108576	0.0394023	0.137853106	0.3223679	0.366713405	0.008416
PPFIA1	0.234751393	0.0394002	0.2269591	0.0454391	0.314309843	0.005623
WBP2	0.376062023	0.0394193	-0.027370557	0.8811214	0.262496144	0.15051
MEN1	0.218999143	0.039469	-0.135299703	0.2031221	-0.237540338	0.027086
RFXAP	-0.585881647	0.0395771	-0.693432092	0.0144227	-0.413874499	0.143041
RNPEP	0.246244394	0.0395673	-0.122724752	0.3066239	0.132725667	0.266732
CDKN3	-0.42657923	0.0396549	-0.049469025	0.8088545	-0.096998889	0.636278
LPIN2	0.247135297	0.0396816	-0.078702213	0.5127662	0.065042335	0.58866
FBXL15	-0.474528526	0.0396976	-0.412655712	0.0699981	-0.421433528	0.063272
LIN7C	-0.189231031	0.0397393	-0.161801445	0.0776687	-0.021043308	0.818418
SLC10A5	-0.683900251	0.0397324	-0.527157344	0.0981502	-0.374399006	0.237664
MAPRE1	0.215927766	0.0397777	0.17964512	0.0863517	0.294804311	0.004879

RPLP0P6	-1.091825807	0.0398732	-0.749110212	0.144833	-1.299853963	0.014889
CCNJL	-0.394606859	0.0399507	-0.373583051	0.0502416	-0.733610845	0.000145
PRTG	-0.651193689	0.0399696	-0.100897137	0.7495307	-0.563433535	0.075592
BOLA2B	-0.340425354	0.0399956	-0.217104541	0.1854537	0.126517261	0.437722
CARD6	0.265566492	0.0400252	-0.08543606	0.5109964	0.278570472	0.030441
AP3S1	-0.19428375	0.0400798	-8.35E-05	0.9992887	0.089468021	0.339007
ZNF557	-0.213979706	0.0400848	-0.19776424	0.054954	-0.402241793	0.000118
PCDH17	-0.515903857	0.0401448	-0.476981498	0.0569169	-0.580777169	0.02072
TWISTNB	0.316507114	0.0401418	0.117196143	0.4464137	0.43533895	0.004636
RANBP1	-0.24477004	0.0402138	0.078383592	0.5083014	0.061138732	0.606338
RAD52	-0.501687967	0.0403124	-0.117004734	0.6292037	-0.510483968	0.038645
SIK2	0.352424013	0.0403383	0.377596451	0.0280115	0.453990541	0.008254
SLC41A2	0.35357145	0.0403381	0.236895537	0.1689366	1.002436098	5.08E-09
TBC1D2	-0.438301928	0.0402956	-0.458927733	0.0301309	-0.258040904	0.2213
ZNF292	-0.284355438	0.040284	0.004801979	0.9723136	0.095162535	0.49168
ILK	0.239879662	0.0403913	0.045491608	0.6972826	0.167709131	0.151672
SSBP4	0.373793437	0.0403869	0.198319225	0.2765366	0.162961139	0.371469
YME1L1	0.159649386	0.0403908	0.064011108	0.4100235	0.20095795	0.009685
FGFR1	0.505988683	0.0404072	0.549407388	0.0259145	0.214501922	0.385306
HIST1H4A	-0.43781599	0.0404392	-0.291837076	0.1715463	-0.20382375	0.33963
AKT1S1	0.240882919	0.0405147	-0.091826599	0.4349743	-0.050906622	0.666787
EBAG9	-0.298872608	0.0405252	-0.117260985	0.413743	-0.168136518	0.242912
NIPAL2	-0.369479999	0.0406408	0.055823007	0.75409	0.355339339	0.045912
C17orf107	0.747806337	0.0406903	0.544643763	0.1343321	0.705410723	0.055433
DPY30	-0.227672103	0.0406893	-0.314884943	0.0044279	-0.303182872	0.006416
ACADM	-0.188262004	0.040794	0.066434976	0.4641071	0.112524144	0.215605
MLH3	-0.205502207	0.0408024	0.025317663	0.797937	-0.21054613	0.035178
TMEM200A	-2.240807355	0.040784	2.599827775	0.004528	1.243978839	0.178626
TMEM11	0.264270881	0.0408405	-0.055231494	0.6696248	0.047757391	0.712073
CDCA2	-0.487430823	0.0409167	-0.119177166	0.6152561	-0.429751536	0.070739
GTPBP1	0.310958945	0.0409282	-0.02907268	0.8483322	0.315614554	0.03805
MTERFD2	-0.323451772	0.0409763	-0.083717093	0.5928454	-0.379778517	0.016082
SERINC2	0.280205913	0.0410025	-0.134026447	0.3294603	0.276529078	0.043447
DCTPP1	0.350321256	0.0411236	0.280292865	0.1014085	0.519695534	0.002308
STC1	0.863758175	0.0411091	0.724773407	0.0865455	0.492248367	0.245308
AKTIP	-0.300119336	0.0411525	-0.346909395	0.0168985	-0.248837868	0.086101
UBAP1	0.245016262	0.0411658	-0.07288747	0.5441435	0.125904472	0.29387
UBR3	-0.273934614	0.0412742	0.044885392	0.7369886	0.042491179	0.750797
ANO8	0.393513556	0.0413329	0.056305977	0.770356	-0.105412225	0.587443
SMAD4	0.192765732	0.0414101	0.039313184	0.6768121	0.283342007	0.002669
CDS1	-0.303215322	0.0414346	-0.428394607	0.0039377	-0.109010422	0.462601
INSIG1	0.331187218	0.0415305	0.111958599	0.4907811	0.379112567	0.019605
GREM1	0.808238017	0.0417384	-0.11201323	0.7784544	0.640911341	0.106581
MED14	0.363652466	0.041896	0.023481301	0.8955011	0.072904743	0.683131
RARS2	-0.192979125	0.0418891	-0.13869638	0.1402237	-0.017958215	0.848469
MCMBP	-0.166621996	0.0419791	-0.133369246	0.1017302	0.043261831	0.595214
MFAP4	0.431857507	0.0419551	0.278097585	0.1903309	-0.137715279	0.517508

ONECUT1	-0.885037	0.0419853	-1.335123313	0.0022369	-0.727766113	0.09383
ZNF665	-0.39226118	0.0419391	0.070932297	0.7062131	-0.104180709	0.585592
ARHGAP33	0.882509289	0.0420418	0.028273797	0.9487907	0.684674159	0.117556
SLC25A16	0.447359065	0.0420545	0.432788868	0.0487683	0.516413852	0.018671
DPP9	0.384623763	0.0421754	0.311637957	0.099428	0.131870745	0.486522
MEGF10	1.521056874	0.0421836	1.393096932	0.0624576	1.527685391	0.040778
TMEM261	-0.21083775	0.042175	0.013944216	0.8915764	0.102682038	0.316211
P11-641D5.	-0.261893124	0.0422188	-0.185940622	0.1487784	-0.141782325	0.270994
OSGEPL1	-0.337869784	0.0422379	-0.347784072	0.0334323	-0.394493162	0.017102
PIK3CD	1.175130176	0.0424051	1.530043626	0.0085969	0.98723302	0.088774
SCAF8	-0.120891776	0.04239	-0.175335881	0.003083	-0.226827512	0.000136
MPC1	-0.237269688	0.0424778	-0.025093881	0.824891	0.059735928	0.598808
SIRT2	0.244254211	0.0424867	0.030078752	0.8025839	-0.058553236	0.62798
LEPREL1	-0.555707296	0.0425177	-0.322169595	0.2387768	-0.037830774	0.889986
ELP4	-0.215563137	0.0426516	-0.186448701	0.071576	-0.130003489	0.212767
MBD4	-0.246975631	0.0426489	-0.376949886	0.0019334	-0.118509869	0.328466
PAX5	1.198355141	0.0426871	1.743509179	0.0022749	0.889380438	0.129734
SGSM1	-1.387809783	0.0427053	-0.694028233	0.3020957	0.283039116	0.672014
COQ3	-0.471139248	0.0427313	0.091366803	0.6790962	-0.081054279	0.719365
PDE7B	1.419826242	0.0427584	1.040130241	0.1406563	1.354745753	0.055462
MRE11A	-0.223628349	0.0428106	-0.16758468	0.1255864	-0.327578689	0.002924
CCRL2	0.890933973	0.042858	0.92302063	0.0348609	0.265634612	0.550681
CNOT7	-0.241184854	0.0428425	-0.043746203	0.7126915	-0.179634134	0.130866
P11-459D22	-0.96901497	0.0429374	-0.413752733	0.3427206	0.242948298	0.559129
TB-147C22	0.893440097	0.0430578	0.169365929	0.7080658	0.787780918	0.074013
DDX11	-0.282996588	0.0430185	-0.485173829	0.0005199	-0.48095063	0.000597
ELAC2	-0.23711401	0.043043	-0.198494975	0.0891704	-0.138181421	0.236485
ENPP4	-0.394272625	0.0430361	-0.437457801	0.0243534	-0.160096764	0.408846
POM121	0.767703027	0.0430288	0.692840767	0.0678728	0.192982407	0.611822
PMPCB	-0.196047466	0.0431324	0.010077253	0.9163551	0.015465148	0.872302
SUPT7L	0.193745401	0.0431243	0.059121979	0.5353888	-0.013127349	0.891147
CYB5R3	0.306890546	0.0431571	-0.062606398	0.6802791	-0.0461326	0.761591
FAM86DP	-0.375926512	0.0432088	-0.245175635	0.1817777	-0.577019635	0.001936
SLC39A7	0.44367071	0.0432098	-0.057553643	0.7933555	0.293705115	0.180523
SNRPA1	0.234551656	0.0432031	0.017489661	0.8800359	0.206275605	0.074058
ZDHHC3	0.210517867	0.0432392	0.005768548	0.9556722	0.213616352	0.039247
NSUN5P2	-0.820864399	0.0432682	-0.71101581	0.076966	-0.997495197	0.013976
TMBIM6	0.185514679	0.0433183	-0.011168101	0.9031879	0.324013095	0.000415
NPM1P25	0.621513241	0.0434081	0.180972188	0.5613401	0.549745717	0.072906
HHEX	-0.878911005	0.0435233	0.465336572	0.2799112	-1.091442623	0.012168
CBR3	0.934734825	0.0435405	0.865031335	0.0594227	0.892399193	0.052686
RALGAPA1F	-0.754277294	0.0435959	-0.300640232	0.4081303	-0.376000512	0.303747
AC098826.5	0.901385671	0.0436178	1.169503989	0.008111	0.587766568	0.190346
CCDC84	-0.417409755	0.043665	0.020948103	0.9153366	-0.319688292	0.114646
NIT1	0.223564649	0.0436574	-0.055817887	0.6137276	0.135666894	0.21778
ADAT2	-0.384176059	0.0437357	-0.229616569	0.2189633	-0.24426352	0.194634
SPRED1	0.28126576	0.0437863	0.260172591	0.0617244	0.138016037	0.321955

UBALD2	0.576189606	0.0438224	0.360115424	0.2078125	0.699070917	0.0141
CCDC142	-0.492221648	0.0438388	-0.270458852	0.2630992	-0.50673098	0.038441
GPRC5A	0.514660863	0.0438774	0.214133101	0.4017584	0.297012916	0.244865
ADRA2A	-0.73391469	0.0441191	-1.628950469	1.13E-05	-0.553349274	0.126736
C16orf62	-0.211240258	0.0441121	-0.253275443	0.0155056	-0.185797856	0.076157
IGLON5	0.512489263	0.044104	0.368059649	0.1472632	-0.376717728	0.148969
CHD6	-0.278995975	0.0441789	-0.142196983	0.3051414	-0.175874639	0.204759
PNPO	-0.406722146	0.0441634	-0.01532167	0.9383224	-0.317211369	0.114678
APBA3	0.363950534	0.0442539	0.192571041	0.2821493	0.195395169	0.278213
FGA	-1.394838157	0.0442526	-0.077819013	0.909966	-2.160545365	0.001942
BTF3L4P2	0.317938631	0.0443168	-0.147349368	0.3562031	0.10052541	0.526218
OGFOD2	-0.278468543	0.04431	-0.542323924	8.68E-05	-0.574120501	4.81E-05
LIG1	-0.279246088	0.0443349	-0.116590345	0.4005043	-0.166899311	0.228114
CSTF2T	-0.192978439	0.0443893	-0.236065145	0.0134156	-0.318138492	0.000913
C3orf52	0.239086463	0.0444541	-0.02903929	0.8071306	0.483015925	3.86E-05
TD-2287O16	-0.357570047	0.0444528	-0.442874181	0.0127116	-0.392155421	0.027401
FAHD2A	0.338350076	0.0444427	0.092558422	0.5826203	0.353678234	0.035597
ARHGEF34P	0.329966829	0.0445838	-0.118649979	0.4709753	0.168472783	0.30519
EIF5B	0.468659646	0.0446343	0.163263488	0.4843398	-0.042348331	0.856347
PCDP1	-0.595293705	0.0446499	0.509961955	0.0708971	-0.240295372	0.402787
RANGRF	-0.305738732	0.0447332	-0.101366167	0.4923824	-0.087931846	0.552109
S100A6	-0.31324682	0.044725	-0.213305422	0.1715174	0.050509009	0.746069
SIKE1	-0.193936395	0.0447262	-0.143648045	0.134904	-0.136024618	0.157566
UBE2L6	0.445875009	0.0447237	0.402845256	0.0685872	0.460766563	0.037519
TMEM5	-0.249486433	0.0447675	-0.275322828	0.0244274	-0.123868277	0.310553
ZNF544	-0.2160008	0.0447529	-0.290539781	0.0064987	-0.351674948	0.00104
DAZAP2	0.243425305	0.0448233	-0.159110138	0.1898338	0.209164631	0.084368
HDAC11	-0.402536106	0.0448091	-0.480246376	0.0156639	-0.422132317	0.034755
HECTD3	-0.288510657	0.0449013	-0.156748094	0.2721332	-0.485993223	0.000752
NAT6	0.477545696	0.0448942	0.382427128	0.1059516	0.322296706	0.174716
PIN4	-0.336642065	0.0449321	0.092770997	0.5732951	0.030595885	0.853535
DACT3	0.419826387	0.044984	0.56628558	0.0065536	0.178749526	0.393946
RTN2	0.51539915	0.0449698	0.553273972	0.029249	0.502323904	0.049969
ZNF77	-0.3130084	0.0449597	-0.571376299	0.0002677	-0.56197778	0.000369
TIMP2	0.36546508	0.0450165	0.364541789	0.0452049	0.143655753	0.43102
DDX47	0.176150562	0.0450598	0.10010174	0.2529383	0.292372531	0.000817
LACTB2	-0.365515445	0.0451214	0.172523776	0.3351706	-0.006743996	0.970078
BRINP3	0.969911751	0.0451493	0.340610086	0.4831195	0.376516389	0.437907
AC242988.1	-0.372278342	0.0452046	-0.2884809	0.1185892	-0.155350633	0.40038
EIF2AK1	0.186190657	0.0452205	-0.189828803	0.0410302	0.008437919	0.927545
KLC4	-0.330238133	0.0451763	-0.36616574	0.0260565	-0.281371793	0.086974
ZHX3	-0.30066694	0.0452202	0.092362971	0.5332573	-0.088742267	0.55137
FAM153C	-0.959394391	0.0453001	0.127315818	0.7804247	-0.559603884	0.237258
FAM161A	-0.426963782	0.0452911	-0.038886893	0.8521824	-0.195505073	0.35457
GINS1	-0.315662096	0.0453442	-0.059680225	0.7031607	-0.013660823	0.930486
UBE2K	0.232597412	0.0453421	-0.072986488	0.5293659	0.106909595	0.356321
C14orf28	-0.659326917	0.0454151	-0.358848366	0.2648874	-0.598397998	0.064985

FAM115A	-0.146850838	0.045432	-0.10355985	0.1563366	-0.239075255	0.001114
NCLP1	-0.648477891	0.0454554	0.395546543	0.1800091	-0.258199876	0.40388
CTNNA1	0.158649951	0.0455366	0.13507512	0.0885386	0.28562175	0.000313
GSTM2	0.398376949	0.0455268	0.145184785	0.4653403	-0.206758941	0.306465
PDK1	0.315451638	0.0455153	0.163724272	0.2987511	0.469659664	0.002854
TRIM2	-0.204637092	0.0455155	-0.200133175	0.0500804	-0.103740618	0.309928
MANBAL	-0.198601352	0.0455639	-0.104705072	0.277413	-0.068118324	0.483167
ASB14	-0.670569003	0.0456117	-0.154869322	0.623641	-0.737422158	0.024411
APOD	1.901490945	0.0456565	1.252583933	0.1895348	3.340807329	0.000404
IER3IP1	-0.235963697	0.0456485	-0.218919082	0.0624599	0.001943257	0.986803
IFT80	-0.31492479	0.0456617	0.020002905	0.8983304	0.181121699	0.247772
RPAP1	-0.39264606	0.0458654	-0.232619269	0.2352734	-0.316984331	0.107174
ABCC6P1	-0.866180714	0.0459328	-0.34546745	0.4225854	-0.979850562	0.024577
AC243756.2	0.547123671	0.0461222	0.177679658	0.5205516	0.266929388	0.334692
BCL2L12	0.381064111	0.0461222	-0.019587324	0.9184322	-0.094351028	0.625031
MORC2	-0.264193387	0.0461046	-0.199498689	0.1307097	-0.073662302	0.576613
NUDT4P1	0.547123671	0.0461222	0.177679658	0.5205516	0.266929388	0.334692
TFAP2A	-1.333743143	0.0461096	-0.362937431	0.5725515	-0.643696801	0.326455
PFN2	-0.162694688	0.0461489	-0.085100843	0.294345	-0.075355074	0.353694
APIP	-0.217836882	0.04638	-0.021135811	0.8420078	-0.042513551	0.69019
C5orf56	0.65532606	0.0463482	0.51724073	0.1111817	0.309737091	0.353227
RNF24	0.350301	0.0463947	-0.044221367	0.8016925	0.022293428	0.899321
SIRT7	-0.321169388	0.0463566	-0.559261408	0.000525	-0.521344274	0.001233
SNX10	-1.542405401	0.0463862	-1.029942098	0.1564856	-0.72301667	0.315731
SRSF12	0.467619341	0.0463554	0.920591349	7.11E-05	0.675843135	0.003707
STAT5B	0.247428401	0.0463159	0.253933571	0.0400846	0.074179373	0.550586
TGFBR2	-0.238671385	0.0462746	-0.553743439	3.82E-06	-0.58450662	1.10E-06
ZCRB1	-0.225288777	0.0463595	0.039898967	0.7202459	0.141814431	0.202966
ZNF354C	-0.386748077	0.0462709	-0.275770428	0.1533114	-0.585771841	0.002563
UBA52	0.201897115	0.0464576	-0.017914846	0.8597461	0.194677548	0.054726
CKMT2	-0.963824042	0.0465916	-0.259648303	0.5747801	-0.944231307	0.051473
ENSA	0.211965569	0.0466145	0.185638248	0.0808419	0.485995954	4.76E-06
UBE2V1	0.19398115	0.0468129	0.089412333	0.3589953	0.042658238	0.662122
CDAN1	-0.402234166	0.0468771	-0.255001497	0.206134	-0.475593363	0.019098
LTB4R	0.406998117	0.0468668	0.088014086	0.665846	-0.031821659	0.87693
EPM2A	0.930405587	0.0469653	0.932539331	0.0463299	0.366479998	0.438995
ERF	0.318452746	0.0469941	-0.011201972	0.9443632	0.116437191	0.468914
RNF138	-0.252298947	0.0469739	-0.176997732	0.1607951	-0.026646156	0.832711
SPACA4	-0.837313559	0.0469816	-0.948517471	0.0221872	-0.926990226	0.026912
HDAC1	-0.149657278	0.0470611	-0.183481376	0.0146204	0.002189299	0.976722
TMEM14A	-0.262942131	0.0470249	-0.236176726	0.0690007	0.091814801	0.473182
TMX1	-0.189231731	0.0470681	-0.205600148	0.0300334	-0.126071742	0.183811
TNK1	-0.648963203	0.0470675	0.15526	0.6325058	-0.123763797	0.703107
POGZ	0.244569335	0.0471836	-0.024578025	0.8420596	-0.235803179	0.056658
KIF7	0.517847713	0.047263	0.458186048	0.078295	0.039597836	0.880693
PEX11A	-0.39344212	0.0472635	-0.756747362	0.0001107	-0.687282707	0.000555
THNSL1	-0.322583024	0.0472644	-0.104746686	0.5036968	-0.15286414	0.334817

ERCC4	-0.344564922	0.0473682	-0.113606281	0.5095314	-0.209198025	0.226687
ZNF844	-0.385782652	0.0473698	-0.114385842	0.5503682	-0.23887751	0.217758
C11orf52	-0.444702754	0.047465	-0.591471394	0.0086007	-0.31213792	0.161327
METTL23	-0.270783538	0.047497	-0.182115868	0.175162	-0.175301624	0.191749
NKX6-3	-2.717998006	0.0475098	-3.432976598	0.0133127	-0.378793439	0.777532
RNF125	-0.682984422	0.0474796	-0.525166215	0.125479	-0.564712768	0.098562
EEF1A1P3	0.765076857	0.0475621	0.6069904	0.1138806	0.717513413	0.06141
APOPT1	-0.231328635	0.0476643	-0.279649348	0.0151683	-0.340566207	0.003316
CEP57	-0.289193945	0.0477086	0.022551546	0.8768446	-0.075898833	0.602458
DUOX1	-0.700627856	0.0477031	0.52312489	0.1288741	-0.123089568	0.723689
DYNLRB1	0.239937637	0.0476762	0.163464227	0.1762665	0.209952002	0.082644
RNF8	-0.224991322	0.0476518	-0.201215534	0.0718098	-0.310407311	0.006129
SKIL	0.348444666	0.0477217	0.310463316	0.0776666	0.627445927	0.00036
EM56-RWD	0.681491503	0.0477263	0.325454681	0.347468	-0.412427234	0.274928
ARL8B	0.557576339	0.0477678	0.74314344	0.0077995	0.659512304	0.018397
NOL6	0.256690103	0.047792	0.247627797	0.055711	0.277613539	0.032031
TMEM106B	-0.195585138	0.0477949	0.001854675	0.9849585	-0.067623139	0.492796
ZNF767	-0.422302659	0.0478242	-0.211013223	0.3183305	-0.435985387	0.040442
PSMD5	-0.223635743	0.0478619	-0.115994497	0.2976218	-0.277921561	0.013221
TRMT11	0.265967985	0.0479489	0.528777012	6.90E-05	0.272683644	0.041138
C11orf83	-0.311908947	0.0480174	-0.463035803	0.0031198	-0.141370877	0.360377
VDAC3	-0.156242707	0.0480013	-0.087410617	0.2647532	-0.087823156	0.263472
ZNF671	-0.412204464	0.0480037	-0.524797582	0.011472	-0.643994798	0.002211
AKR1B10	-1.414445938	0.0481033	-0.347738757	0.6240937	1.01627952	0.150438
HOOK3	-0.308540182	0.048113	-0.189895815	0.2230898	-0.032584301	0.834489
ZBTB33	-0.181437207	0.0481285	0.000245786	0.9978406	-0.124811698	0.171669
PCBP1	0.238665187	0.0481942	0.06074471	0.6147479	0.145092302	0.229379
COG3	-0.219756997	0.0482726	-0.242931896	0.0285105	-0.085012486	0.443469
KBTBD4	-0.292045573	0.0482286	-0.415374119	0.0046657	-0.432827133	0.003378
RPUSD4	0.187124378	0.0482591	0.03955496	0.6751073	0.137349786	0.14407
TMEM185B	0.236805625	0.0482708	0.138915289	0.2433114	0.210322215	0.077688
ARFGAP1	0.291678547	0.048386	-0.133425287	0.367017	-0.138801899	0.348162
DOCK8	-0.519700511	0.0483802	-0.704227933	0.0067507	-0.421460084	0.102324
SDK1	-0.704961516	0.0483214	-0.733330244	0.0384832	-0.771687333	0.030173
SURF2	-0.348477416	0.0483731	-0.126184922	0.4510225	-0.105472632	0.5337
TOP2A	-0.358185816	0.0483664	-0.026509803	0.8837372	-0.309421893	0.087991
AC005517.3	-0.685010656	0.0486752	-0.120833956	0.709257	-0.062373557	0.847799
NARG2	-0.214918665	0.0486798	0.089593506	0.4056888	0.149996904	0.165835
ABCD1	0.415207834	0.0487214	-0.028564179	0.8921172	0.122485028	0.563234
ARHGAP32	0.251922004	0.0487568	-0.015932602	0.9008663	0.322007538	0.011533
COG2	-0.257078499	0.0487323	-0.282642222	0.0297548	-0.14537026	0.263073
FBXO33	-0.193536153	0.0487432	-0.380355714	9.63E-05	-0.239549508	0.013948
FAM199X	-0.211732901	0.0488015	-0.141843932	0.1850159	-0.033197983	0.756391
SAMD4B	0.325692707	0.0488278	0.095714627	0.5591332	-0.035944047	0.82766
LATS2	0.36200741	0.0489775	-0.082978514	0.6522605	-0.164931245	0.371524
OPA3	0.223702973	0.0489784	0.244985987	0.0306316	0.137027891	0.228321
SCOC	-0.264307183	0.0490302	-0.375826708	0.0051014	0.048362611	0.717648

ATP9A	0.193275836	0.0491787	0.010326852	0.9162588	0.30841254	0.001658
PLA2G7	1.433137177	0.0492237	1.500183063	0.0381029	0.970759992	0.187357
PBK	-0.509513453	0.0492857	-0.085239408	0.7401998	-0.100026634	0.697815
ACSM2B	-0.549311409	0.0493708	0.258111197	0.3374534	-0.090981185	0.738773
AFAP1L1	-0.78230063	0.0493708	-0.757783678	0.0525005	-1.149454818	0.00414
MYO5C	-0.328263958	0.0493539	-0.324340917	0.0518222	0.147689469	0.373846
COA7	-0.273640267	0.049492	-0.317530187	0.022066	-0.347386373	0.012549
KRT13	-2.284348613	0.0495648	-1.250344402	0.2802269	1.092271993	0.343395
DOK1	-0.338727861	0.049599	-0.01350935	0.9347396	-0.424961551	0.013032
ACVR1B	-0.29989494	0.0496344	-0.345253132	0.0232554	-0.392210713	0.010081
ZNF799	-0.34774588	0.0496847	-0.409400959	0.0202965	-0.142569553	0.416394
ZNF136	-0.355037697	0.0497643	-0.594542916	0.0010525	-0.329598748	0.069112
ZNF28	-0.281049155	0.0497489	-0.352723159	0.0138212	-0.196951732	0.168754
BICC1	0.544903499	0.0498893	0.593975311	0.0323426	0.683714717	0.013729
RAB5C	0.185194307	0.0499199	0.157610278	0.0939729	0.260823524	0.005575
ZNF501	-0.679417092	0.0499192	-0.390504052	0.2427705	-0.817125286	0.016098
AC003989.4	2.013637948	0.0205907	1.352167043	0.1308562	0.687297978	0.474448
AC005037.6	3.605849814	0.0071136	3.067522311	0.022975	2.998255974	0.026926
AC005488.1	-2.539495518	0.0422948	-1.489005811	0.1786402	-0.156353759	0.863101
AC006548.19	-3.832014766	0.0108876	-0.477245276	0.6831145	-2.654346567	0.05501
AC007272.3	3.939941069	0.0013378	3.130988487	0.0139588	3.648385329	0.003234
AC010733.5	2.656517205	8.87E-06	1.556463171	0.0122798	2.465719454	3.84E-05
AC011737.2	-4.614755928	0.0203022	-1.110546911	0.5298955	-0.403864273	0.817191
AC011933.2	2.146057047	0.0480153	1.888317649	0.0821938	1.453177327	0.193893
AC092338.5	-2.15830943	0.0082621	-0.618037595	0.3106106	-0.523878823	0.390329
AC092798.2	1.633288943	0.0350926	1.367528863	0.078147	1.130284371	0.153897
AC106827.1	-2.443551536	0.0320973	-1.253265852	0.1786452	-0.994580009	0.27822
AC112229.1	-1.789686905	0.0023517	-1.482777532	0.0088524	-2.024842165	0.00064
AC133644.3	2.479327836	0.0469631	1.248158913	0.3348526	-0.232586088	0.87381
AC139452.2	-2.524945525	0.0421638	0.397989303	0.6332129	0.952518658	0.235082
ACTBP2	1.519094627	0.0452345	1.24809086	0.1007933	0.417270917	0.614956
ADAMTS7P3	3.181054061	0.0100114	1.481111421	0.2242952	0.460137281	0.717982
ADAT3	1.283811605	0.0152407	0.058793342	0.9152959	0.746177709	0.162539
AGXT	1.289003065	0.0286808	1.435574809	0.0128749	0.810807346	0.17408
AIRE	1.453183246	0.0189614	0.73076554	0.2357375	0.411624461	0.519122
ALG1L	1.495697295	0.0460287	0.896091981	0.2403322	0.587280964	0.452992
ALG1L2	-1.047889477	0.044616	0.017887404	0.9687758	-0.650854532	0.183335
AOAH	-1.552664784	0.0294059	-0.610546403	0.3763546	-3.360342203	2.64E-05
AP000619.5	3.821942669	0.0290225	1.649071903	0.3770376	0.497898998	0.795933
AP001258.5	3.00974155	0.0048261	1.318020793	0.2533171	1.214576823	0.300565
AP004289.2	-1.90827877	0.0463008	-0.243971101	0.7402458	-0.239375961	0.747045
APOBEC2	0.945208878	0.0346667	0.015767157	0.9739559	0.228153374	0.631833
ATP6V1G1P	-3.576184027	0.0147094	-1.343940233	0.290954	-2.992369359	0.04123
B3GAT2	2.555854065	0.0438102	2.158633683	0.0893515	2.899583718	0.021462
BCL2A1	4.061528171	0.0003104	4.093959175	0.000258	5.261103646	2.21E-06
BLID	3.649364338	0.0108387	2.51521839	0.0969993	0.919984937	0.582958
C11orf87	4.339792573	0.0128207	2.420691008	0.1714819	3.7397266	0.032429

C12orf74	1.748719637	0.003867	0.908941491	0.1449379	1.117838354	0.07075
C14orf178	-1.260873201	0.0342638	-0.449550233	0.389803	-0.48828652	0.356042
C15orf43	1.534830644	0.0325959	1.328929344	0.0631742	1.683003066	0.017703
C17orf99	1.202992646	0.0320552	0.736347152	0.2026066	-0.530116618	0.371578
C19orf73	-1.528605151	0.021514	-0.15444444	0.7721403	0.284093855	0.583341
C1DP1	-2.843480913	0.0383891	-1.370443711	0.2945632	-2.36962848	0.077167
C2orf74	-4.500961707	0.0119789	0.800938187	0.5896146	-0.152082676	0.919143
C5orf49	-1.575724329	0.0200663	-1.803255045	0.0074448	-0.82976654	0.195802
CACNG7	1.38717585	0.0251944	0.535572879	0.3846851	-0.405527585	0.538063
CADM3	-3.234743221	0.0382254	0.298780345	0.8075649	-4.158096673	0.007722
CAMK2N2	-2.944062281	0.0231345	-0.651922031	0.5006384	-1.358415429	0.207039
CCDC135	-1.809618455	0.0104138	-0.315833868	0.6342356	-0.532425898	0.411481
CCDC155	1.919234498	0.0193497	2.006312238	0.0121794	0.168104947	0.863132
CCDC96	-1.134543111	0.0199636	-0.988675183	0.0335919	-1.316980287	0.007268
CCL11	1.336618227	0.042417	0.921937776	0.1648013	2.084509161	0.001134
CD274	3.928974123	4.48E-07	3.371943821	1.89E-05	3.416125327	1.24E-05
CD33	1.913151829	0.0044991	1.759962339	0.0075113	1.571549267	0.021171
CPN2	1.222879554	0.0155589	1.497248567	0.0023156	1.347743894	0.00677
CPNE5	1.406260531	0.0088954	0.384359847	0.4841795	1.245469428	0.020603
CRH	3.250780598	0.0115404	3.743999712	0.0027747	4.217991531	0.000662
CRYBA1	-2.410088037	0.0358426	-0.876454429	0.2692151	0.111531071	0.873557
CSNK1A1P1	-1.812751369	0.0471724	1.064496072	0.0847453	0.17657016	0.796366
CTAGE3P	-1.49066348	0.0112746	-0.608544405	0.2498844	-1.404168882	0.014274
CTBP2P2	4.431846306	0.0135926	3.166259595	0.0858167	2.626630321	0.165347
TC-260E6.1	-1.991362913	0.0188018	-1.364929903	0.0610043	-0.856024416	0.213629
CTC-459F4.(	3.044718167	0.0166397	2.807019289	0.0272741	1.535970394	0.261365
TD-2349B8	-4.346836483	0.0002507	0.217808854	0.7540796	-1.049100504	0.180045
TD-2372A4	-2.919283867	0.0499028	-1.699777201	0.2500351	-0.285934728	0.820632
CYP4A22	-2.958407842	0.0072587	-0.162918936	0.799904	-1.203535306	0.111398
CYP7A1	3.453665236	6.38E-05	2.776214283	0.0014567	3.553400295	3.61E-05
DDX10P1	-2.718228573	0.0236097	0.454107118	0.545076	0.450214671	0.552701
DOC2B	3.939288474	0.0011789	3.332553097	0.006737	3.328155205	0.006761
DUTP1	-2.852290336	0.0065658	-1.101910571	0.1499985	-1.116594496	0.149497
EIF4BP6	-2.757411499	0.0413716	-0.010463545	0.9915225	0.103971286	0.915851
ETV7	2.26515219	0.0258421	2.162286936	0.0328644	2.538020283	0.011993
FBLL1	2.604735537	0.0216571	1.847284109	0.1119844	2.226141101	0.052082
FCF1P8	1.624095211	0.0107145	0.952631024	0.1429361	2.353245332	0.00014
FDPSP3	2.190800175	0.017571	1.619935793	0.0844465	1.889104184	0.04225
FGF19	2.825055022	0.0490078	3.435326796	0.014554	2.647819873	0.065347
FTH1P16	-2.223938957	0.0156066	-0.281597203	0.6710823	-1.169394167	0.115887
GABRB3	1.934221952	0.0383847	2.301251124	0.0124922	1.525979871	0.107225
GAPDHP21	-1.593455087	0.0329575	0.016714248	0.9781262	-0.277986359	0.657937
GAPDHP40	-2.779925868	0.0322877	-0.970847628	0.3459204	-0.402564454	0.678895
GAPDHP49	4.270600648	5.48E-07	4.52830539	8.92E-08	5.227974475	5.32E-10
GAPDHP61	-1.706609445	0.0328337	-0.005362447	0.9931055	-0.346327498	0.591842
GBX2	4.577063989	0.0202835	3.07908041	0.1260759	3.947879982	0.046542
GJB7	-1.158376816	0.0484447	-0.24942648	0.6515372	-0.82505549	0.14185

GLDN	-1.640788874	0.0304991	0.016441645	0.9803099	-0.668162685	0.317184
GUCA1A	1.006449066	0.0341165	0.131301023	0.7905001	0.28275381	0.566529
H3F3C	-3.399232913	0.0231477	0.668051022	0.5643358	0.717100956	0.536829
HGFAC	2.554148837	0.0161833	1.130825469	0.3067637	0.603063182	0.6051
HIST1H1T	-4.092160719	0.0016315	-0.297486188	0.7425087	-2.781888794	0.031209
HLA-J	1.870107801	0.0076651	-0.102493998	0.897397	1.043846112	0.148641
INRNPA1P1	3.217559066	0.0074372	3.788588033	0.0012794	3.965531416	0.000735
INRNPA1P2	-1.763602507	0.0436782	-1.424593806	0.0774987	-0.497802004	0.501964
INRNPA1P3	2.156529961	0.0222442	2.268818304	0.0153068	2.629352668	0.004734
HSD17B14	1.239655511	0.0053679	0.644170826	0.1490245	0.198870113	0.669171
HSD17B6	-1.970604386	0.0255945	0.123587809	0.8740161	-0.873821525	0.301399
HSFX1	1.414727562	0.0156288	0.588582946	0.3278017	-0.390235626	0.575036
HSPD1P2	5.910700673	0.000227	4.943952456	0.0021421	5.611083522	0.00047
HSPD1P3	5.323482882	0.0004701	5.122687225	0.0007597	6.039741299	6.26E-05
HSPE1P10	-4.148997062	0.0005863	0.108616641	0.8769457	0.438295912	0.527292
HSPE1P13	-2.016964539	0.0072425	-2.086509493	0.0043733	-1.06953975	0.101526
HTR3B	-0.936923431	0.0447862	-0.164896911	0.685305	0.009170698	0.981865
ICAM5	2.589929449	0.0006896	2.112909222	0.0058531	1.710137518	0.027677
IGHEP2	3.35871414	2.77E-05	1.901316837	0.0215231	2.881240285	0.000353
IL10RA	2.524276358	0.0003341	0.818200207	0.2564188	2.256885139	0.001337
IL20	6.374034775	4.36E-05	5.837499756	0.0001896	6.622167361	2.09E-05
IL36G	2.813134258	0.031608	3.21342894	0.0123221	3.828362195	0.002562
IMPDH1P1C	3.586381567	4.19E-05	1.793807472	0.0449775	2.824066773	0.001345
INSRR	3.064862508	0.0343157	1.905973116	0.1983359	3.009290737	0.037418
IRX3	-3.225373867	0.0028794	-1.002617166	0.3034394	-1.361741571	0.155999
JSRP1	2.897335722	0.0268976	0.797654729	0.5777168	0.28716147	0.854917
KARSP1	-2.976904285	0.0379335	-0.192467318	0.8691558	-0.689635153	0.578789
KCNE1L	1.648500219	0.0311672	1.590681536	0.0352944	1.236331221	0.113748
KDM5D	-2.032856547	0.0376144	-0.238844088	0.7474858	-0.9544983	0.21113
KIF25	2.530749714	0.0359952	1.561958597	0.2149233	2.583086303	0.030171
KRT18P19	-3.494803936	0.0152259	-1.289640234	0.3008417	-0.007049502	0.994994
KRT18P34	1.507084367	0.0178127	0.778220706	0.235089	0.875120367	0.181852
KRT8P9	3.548075476	0.0004235	2.947703691	0.0037661	4.056749128	4.37E-05
LGALS9B	1.668138528	0.0202899	0.27551034	0.7091061	1.185481755	0.096692
LRRIQ3	-1.939422996	0.0147328	-0.980006821	0.1717767	-1.695919551	0.031642
MADCAM1	2.068298794	0.0375263	1.608393386	0.1098512	-0.16512142	0.88531
F2BNB-MEI	-1.977040528	0.0442396	0.521692147	0.5456996	-0.213135475	0.807755
MIA-RAB4B	5.696158624	0.0008036	5.629471227	0.0009104	5.211945488	0.002236
MKRN3	-2.055392856	0.0343217	1.1569596	0.0877231	1.044402924	0.139185
MOG	1.887253261	0.0144486	2.022835343	0.0059711	1.964632856	0.011003
MRPL35P3	-4.512279973	0.0056972	-0.428312022	0.7471485	-4.649811701	0.004386
MSC	2.666362269	0.0407703	2.330044022	0.0742014	1.505813262	0.253534
MSMP	3.671961121	0.0393062	-0.587540624	0.7657456	-0.951531371	0.640407
MTND4P14	1.701990866	0.0351217	2.117162449	0.0069905	2.720786235	0.00043
MTND5P14	2.239490869	0.0034505	1.759659897	0.0230153	1.663198637	0.033381
MTND5P25	3.759142871	0.0004405	2.960092076	0.0063337	4.112666145	0.000105
NANOGP2	5.348988834	9.47E-06	4.489828849	0.0002317	5.943465216	7.14E-07

NOTO	-1.098911464	0.0234459	-0.023892653	0.9537368	-0.019829687	0.961932
NPHS1	2.680468365	5.19E-05	3.095230698	1.75E-06	1.993812915	0.002833
NPIPA5	4.893695199	0.0370346	2.896850543	0.2287874	4.91632431	0.035947
NPM1P21	-2.722714143	0.0422642	-0.431875399	0.6740498	-0.555568971	0.596957
NPR1	1.36038779	0.0330848	1.215016685	0.0558002	1.207656553	0.058703
NT5C3AP1	-1.755526133	0.0398511	0.010624335	0.9868849	0.099924803	0.877227
OR13H1	1.654709967	0.0050854	1.293691412	0.0298622	2.433282364	1.60E-05
OR2I1P	2.088552085	0.0144785	2.349401219	0.0050957	3.332104911	4.98E-05
OR52N1	3.775244192	0.0046262	2.884458958	0.0377069	2.625025767	0.066239
OR52N3P	4.628029175	0.0002778	3.152096879	0.0193721	4.095957232	0.001507
OR52N5	2.908000492	0.0324363	2.145743233	0.1268208	1.94136182	0.177471
OR52W1	3.284389478	0.0246336	2.379876151	0.1139679	1.932563399	0.215012
OR7E11P	3.751584246	0.0038977	2.560973294	0.0658144	3.963192012	0.001924
OR7E62P	2.15604065	0.0098593	0.633810903	0.4809857	1.187943797	0.172395
OR7E7P	-1.00460887	0.0426513	-0.581699581	0.2018319	-0.228399844	0.606902
OR9Q1	3.600673871	0.0198538	1.50802865	0.3925997	0.919985723	0.603393
P2RY2	-1.069190635	0.0321116	-0.541376763	0.2501276	-1.099228309	0.027367
P2RY8	-1.704452593	0.0240808	0.207724445	0.7264947	-0.548200822	0.382224
PABPN1L	-3.095620961	0.0351976	-1.909423047	0.1905106	-0.735256181	0.566876
PDE6A	-4.078043736	0.0112195	0.677953705	0.5956836	-4.215575474	0.008759
PENK	3.767219525	0.0186105	2.742336644	0.0903175	2.714677099	0.09262
PES1P2	3.650783588	0.0004447	2.516134129	0.0178054	2.252547254	0.036191
PGLYRP1	-3.020063201	0.0323783	-1.84086695	0.1892651	-1.711894635	0.222925
PGLYRP2	6.228305428	2.75E-06	5.370112758	5.18E-05	5.371873796	5.75E-05
PIH1D3	1.731032967	0.0243021	1.366588659	0.0768556	1.060437756	0.182744
PLAC8L1	-1.580874339	0.0344963	-0.563338041	0.3466491	-0.067826398	0.904494
PLP1	2.921371282	0.0246732	2.985485974	0.0212376	3.625797276	0.005019
PPP1R14D	2.183145347	0.0078242	2.422723204	0.0027125	1.160594603	0.176382
PPP4R4	1.266174412	0.0147893	1.095880985	0.0330891	1.09313711	0.032202
PRSS30P	-2.455815815	0.0132579	-0.567677751	0.5059992	-1.28169502	0.140906
PSG1	-3.221753607	0.0372849	0.760040775	0.5414805	-0.644251199	0.630613
PTCHD2	1.953800882	0.0380949	0.838140539	0.3904266	-0.541391688	0.666585
PTGDR	-3.51183332	0.0455774	-3.118551194	0.0758334	-0.810366547	0.607273
PTGER1	3.403705664	0.0221589	3.344939188	0.0233142	1.646830737	0.333239
RAET1K	1.650340336	0.0488227	1.417653972	0.0907697	1.07642285	0.208168
RANP1	-1.607333449	0.0255289	-0.050195796	0.9335856	-0.362645015	0.558182
RBMS1P1	6.248107474	2.09E-06	5.699115879	1.61E-05	6.18683819	2.58E-06
RCVRN	1.379001486	0.0114157	1.061418452	0.0537333	1.492984185	0.006165
RGSL1	-1.904486837	0.0281863	-0.288955462	0.7210337	-1.740218	0.042032
RP1-131F15.	2.761374595	0.0001681	2.160773385	0.0036532	2.795777466	0.000128
RP1-228H13.	3.334920085	0.0412015	4.493640362	0.0040846	2.25517821	0.192465
RP1-232L24.	-3.523033182	0.0456313	-2.293578603	0.190716	-2.227429275	0.205256
P1-273G13	-0.882343438	0.026653	-0.106351457	0.7631176	-0.130883509	0.713955
P11-100G15	3.076333442	0.0289386	2.640397804	0.0633013	2.920957599	0.038226
P11-137L10	2.434219743	0.0213572	2.396826999	0.022187	3.109501023	0.002412
RP11-17P16.	2.500744665	0.0448312	2.286078376	0.0663632	2.011174446	0.113884
P11-182I10	3.019359213	0.0185696	2.145262406	0.1035217	3.674251998	0.003419

RP11-1J11.1	-1.306382818	0.0198017	-1.198160744	0.0263326	-1.68585497	0.003562
P11-210K20	1.755711947	0.0361686	1.386131981	0.100707	0.705607183	0.430458
RP11-236F9.	2.597175487	0.0058846	2.364377749	0.0124226	2.242555887	0.018448
P11-247I13	-3.345598802	0.0120784	0.432062505	0.6508559	-0.454249147	0.662438
P11-267C16	3.015924725	0.0136388	1.913663466	0.1289114	2.478955365	0.045126
P11-288E14	-2.684442994	0.032846	-1.582171582	0.114607	-0.833693154	0.367777
P11-311B14	2.125102933	0.0214296	2.54713328	0.0043535	2.290559434	0.011668
RP11-34E5.4	1.911245497	0.0229774	1.850191811	0.0266401	0.337971453	0.714342
P11-366M4.	-1.54235084	0.0467928	-0.734204787	0.3218046	-0.659960674	0.373503
P11-380G5	-0.994219021	0.0256662	-0.163348306	0.6725103	-0.236100881	0.548744
P11-382A18	2.885706545	0.0355228	1.39780265	0.3294214	2.009011752	0.151932
P11-397P13	-1.911743924	0.0334558	-0.316409464	0.6303641	-0.717242173	0.307215
P11-39K24.1	2.959279527	0.0122409	2.428573811	0.0415911	3.846465394	0.000885
P11-39K24.	2.925094119	0.0020564	1.398602819	0.1670898	2.182681103	0.024778
P11-39K24.	2.298704754	0.0320775	1.995775622	0.0632961	3.026387231	0.003951
P11-39K24.	3.644864255	0.004784	3.606013342	0.0050664	3.404190788	0.008566
P11-432A8.	-3.461755682	0.0046844	-1.977398345	0.0455392	-0.305789263	0.70067
P11-432N13	1.050691889	0.0256091	0.641395718	0.1799311	0.447166651	0.364374
P11-462D18	1.759106532	0.0374841	0.290920212	0.7543382	-0.02066609	0.983083
P11-467H10	-2.169503026	0.0140192	-1.336518653	0.0901826	5.31E-05	0.999942
P11-470B22	5.870842698	4.06E-06	5.402912012	2.36E-05	6.932679865	3.78E-08
P11-480I12	-2.762187786	0.0477759	0.258977166	0.803769	0.651077581	0.525806
P11-529H22	1.547028444	0.0238442	0.732324464	0.3025794	1.216398374	0.07885
P11-537I16	-3.397684698	0.007395	0.511712547	0.5487622	0.239078985	0.785967
P11-561C5.	4.129128297	0.0464745	4.631865142	0.0252109	3.804196088	0.066986
P11-568G11	-1.05747689	0.0366156	0.013118494	0.9748649	0.330043615	0.417139
P11-655M14	3.14761817	0.0393739	3.388751022	0.0229639	2.219497688	0.170955
P11-655M14	3.52462685	0.0015169	2.862397612	0.0106123	2.874233902	0.010439
P11-734J24	-3.556337011	0.0259129	-0.304844302	0.8182988	-0.725248018	0.595844
P11-74E24.	3.417569028	0.0417527	3.003347317	0.0749249	2.515689785	0.143595
P11-777B9.	2.213991944	0.0333608	1.979170128	0.0571714	2.423008729	0.017642
RP11-79D8.2	2.692341713	0.0186435	1.817350393	0.11625	2.112685302	0.066781
P11-813N20	-2.638434532	0.041969	-1.575474688	0.1733124	-2.058533594	0.110752
P11-829H16	1.737561593	0.0092029	1.851311264	0.0047962	1.369793366	0.04261
RP11-83J16.	-2.108882906	0.0066636	-0.241402648	0.7051919	-0.081007691	0.89866
RP11-8H2.1	2.620441231	0.0070983	1.83883604	0.0656117	3.196949988	0.000776
P13-401N8.	-1.860408211	0.02045	-0.641850902	0.362739	-0.766283306	0.283918
P3-342P20.	3.397124247	0.0211462	2.854334652	0.055396	3.111232709	0.035659
P3-468K18.	-3.08408933	0.0468659	0.168939749	0.8943859	-0.180258066	0.89039
P5-1041C10	1.36968293	0.0464406	1.377921718	0.0420744	1.121746796	0.105419
P5-1049G16	1.106706278	0.0399537	0.818094732	0.1306675	0.751587156	0.171406
P5-1059H15	-3.050966293	0.038979	-1.954497467	0.1479125	-1.881043635	0.165135
P5-1073F15	3.718901718	0.0464026	0.100259686	0.9612341	2.241070229	0.256522
RP6-24A23.0	1.982185917	0.0327888	-0.023832631	0.9816222	2.523583907	0.005594
RPL12P2	-2.728550427	0.0484311	-0.520952714	0.6586349	-0.102523051	0.927999
RPL13AP20	-2.665824839	0.0041863	-0.786227	0.3068486	-0.189143336	0.801808
RPL13P4	-3.490097335	0.048717	-0.764313414	0.6259062	-2.906282952	0.100725

RPL21P23	3.940403245	0.0014784	3.921355208	0.0014524	4.572171539	0.000169
RPL23AP5(	-2.464071956	0.0332887	-1.120080674	0.1801621	-0.423144944	0.578406
RPL31P20	-4.003287582	0.0006754	-0.768517351	0.2775564	-0.844810722	0.241773
RPL37P1	2.723601569	0.0346657	0.675284316	0.6464792	2.221400003	0.090345
RPL4P3	-1.645235614	0.0185259	0.169103125	0.761135	-0.324908704	0.576472
RPL5P9	-1.57015913	0.0471584	0.404747493	0.493116	0.435937821	0.462735
RPL7L1P10	2.196834461	0.0424641	2.084470749	0.0526116	1.94616068	0.07418
RPL7P4	-1.973769408	0.0115601	0.004896759	0.9926522	-0.334028426	0.551628
RPL7P6	-2.418664953	0.0074679	-0.427635859	0.5032552	-0.835379102	0.215575
RPL7P7	-1.684399857	0.0483491	0.308112698	0.6618766	-1.315415305	0.100376
RPS15AP3{	3.766820486	0.0219986	1.526999734	0.4113471	2.221183388	0.209882
RPS4XP10	-2.648661892	0.0474538	-0.163217432	0.8706637	-0.753339407	0.485011
RPS6P16	2.685390653	0.0003534	2.344975426	0.001896	2.839243547	0.000134
RTEL1P1	1.942911527	0.0437086	1.738895411	0.0705269	2.308204046	0.013882
SAA1	2.943114899	0.0450985	0.100263583	0.9531585	0.198642887	0.907353
SEPT7P9	-4.258364125	0.0060318	-1.571126341	0.2136771	-3.574088414	0.020008
SETP6	3.399652569	0.0241401	3.341017652	0.0253919	2.233912191	0.16791
SH2D1B	2.048087289	0.0013553	1.076436124	0.1003141	1.886899829	0.003294
SIRPB2	1.388766827	0.0329093	0.911839896	0.1651672	-0.34396666	0.640602
SLC22A16	-1.057509389	0.0478239	-0.64591058	0.1842721	-0.205924665	0.665086
SLC6A6	-1.741225713	0.0370597	-0.84780494	0.2988491	-0.998311939	0.223707
SNRPGP5	2.605353731	0.0320631	3.160126358	0.0072882	4.046769406	0.00046
SNX25P1	-2.630315481	0.0119253	-0.851711111	0.2589311	-0.762591389	0.312593
SNX5P1	-1.112990078	0.0198515	-0.302699574	0.4762618	-0.2800938	0.512624
SPHKAP	-3.867143699	0.0476734	-0.604849809	0.7272898	-2.67966416	0.168038
SPINK13	1.735426109	0.0082472	0.158056705	0.8235464	1.066310321	0.112033
SPRR2D	5.019423825	7.19E-05	3.029981099	0.0223304	4.679440204	0.000224
SPTA1	-2.85206089	0.0277378	0.326339416	0.7804026	-2.121444722	0.087314
SRGAP2B	4.669795821	0.0276219	5.311937112	0.0116553	5.780575361	0.005956
SULT1A2	-1.399255698	0.0402457	-0.049595893	0.9366511	-1.071274951	0.108465
TBX10	-1.426929485	0.0205279	-0.451265078	0.3887879	-0.091719431	0.85749
TCAP	-1.38623861	0.0052682	-1.078555477	0.0193621	-0.85804812	0.0692
TCP10	-3.440682954	0.0407458	-1.030939971	0.48878	-2.213265309	0.186171
TDRD6	-2.474056791	0.0404071	0.661367941	0.5546781	0.839452233	0.457708
TFAP2B	-18.63118294	1.35E-22	-0.282602068	0.8647241	-1.614682549	0.360046
TMIE	2.098730744	0.0208019	-0.432516148	0.6798505	1.392702633	0.132714
TMPRSS11E	-1.560575756	0.0443155	-0.080722083	0.9045728	-0.01279754	0.984883
TNFRSF18	2.931341059	0.0249962	1.841673815	0.1784396	2.751972622	0.034812
TNNI2	-1.519814645	0.0146253	-1.39559233	0.0196407	-0.98377613	0.087116
TPM3P6	-1.955872055	0.0351171	-1.939519861	0.0306059	-0.823093169	0.308284
TSPAN32	1.959131288	0.0145586	1.695596065	0.0431401	0.683777102	0.416514
TUBB4A	-2.616478682	0.0382478	-1.448818782	0.1848944	-2.474001959	0.060652
TYRO3P	-1.306110969	0.0224135	-0.698800963	0.1876934	-0.904746235	0.095708
UBA52P6	3.070037365	0.039099	2.095407619	0.1849347	2.243551771	0.153801
UBE2QL1	2.358930141	0.0317391	-0.222300438	0.8517296	2.257557165	0.039722
ULBP2	3.107399165	0.0359418	2.965263606	0.0445371	3.214918639	0.028523
USP2	-1.574307282	0.0298407	-0.371450479	0.5679594	-1.416282981	0.043377

USP26	-2.691686902	0.0356135	0.319003139	0.7714833	1.098211311	0.318799
WNT10B	2.456355844	0.0381962	3.317082918	0.0037754	2.211249998	0.06369
YBX1P10	-2.844573245	0.0481332	1.440989144	0.1798993	0.783463687	0.481578
ZNF300P1	1.756872901	0.0380322	0.912340859	0.2985604	1.025429693	0.240058
ZNF587P1	-1.440403506	0.0413819	-0.769141942	0.2301184	-0.395402067	0.528359
ZNF831	3.52997655	0.0162486	4.554689495	0.0011988	2.206165589	0.167223
ZNF962P	-4.081942535	0.0032379	0.448077884	0.6517504	-0.453424964	0.663997
ZSWIM5P2	-3.020861539	0.0272394	-0.054228895	0.9571265	-0.282756605	0.784075
RECQL5	-0.264104706	0.0594779	-0.767110351	5.31E-08	-0.364269231	0.009135
STK35	0.041073544	0.5522343	-0.373795093	8.34E-08	-0.184581742	0.007796
HEXIM1	-0.191122424	0.1420521	-0.684183471	1.76E-07	-0.722990491	3.85E-08
PAIP2B	-0.210484403	0.0831565	-0.624940573	3.49E-07	-0.521472073	2.11E-05
TBC1D20	-0.100027086	0.1594486	-0.340093738	1.68E-06	-0.303748841	2.01E-05
RAB40C	-0.236775666	0.1073179	-0.702757632	2.10E-06	-0.400355891	0.006416
PDRG1	-0.261739096	0.140666	-0.850458126	2.19E-06	-0.636722095	0.000372
MYO1B	0.160110657	0.1628985	0.530277651	3.43E-06	0.022325146	0.845573
RP11-583F2.	0.282471834	0.3996408	1.441214894	3.44E-06	0.85989758	0.007265
DPY19L1	0.25788627	0.1044201	0.732499551	3.62E-06	0.41723176	0.008419
NAA30	-0.038304116	0.7113357	-0.471499156	4.85E-06	-0.501024989	1.38E-06
ZMYM3	-0.137376033	0.1467432	-0.43267668	5.22E-06	-0.594018861	5.34E-10
PIK3C2B	-0.279625245	0.0954764	-0.732393209	1.30E-05	-0.565950014	0.000747
DOCK9	0.398413385	0.0724418	0.954787213	1.60E-05	0.491466372	0.026634
SPRY2	-0.269904517	0.1364365	-0.783471292	1.61E-05	-0.709623135	9.42E-05
CYHR1	-0.156223951	0.1852423	-0.502364554	2.19E-05	-0.238220439	0.044169
PRMT2	-0.042601396	0.5256266	-0.283400549	2.35E-05	-0.184401954	0.005968
HM13	-0.136973501	0.1411123	-0.392878939	2.46E-05	-0.337101541	0.0003
ZNF227	-0.315667812	0.061716	-0.715000624	2.55E-05	-0.641072362	0.00016
OVOL1	-0.094551863	0.6000538	-0.75779145	2.95E-05	-0.182130708	0.31191
TNNI3	-0.348322978	0.1747178	-1.118550978	3.52E-05	-0.320567176	0.208728
FPGT	-0.28323521	0.0671665	-0.63052027	3.73E-05	-0.527215559	0.00064
YTHDF2	-0.119002531	0.2918244	-0.465693781	3.80E-05	-0.320352586	0.004593
METTL3	-0.157703964	0.0702549	-0.356669741	3.90E-05	-0.338795283	9.98E-05
GORASP1	-0.044024764	0.6710733	-0.41918914	5.18E-05	-0.447003404	1.63E-05
CLK1	0.191135649	0.2476753	0.665202115	5.31E-05	0.850373598	2.37E-07
SPINK5	0.006132083	0.9730971	-0.733231674	6.23E-05	0.454994913	0.011781
PLAGL2	0.05917904	0.7110046	-0.643396846	6.45E-05	-0.40317972	0.012045
ZBTB42	-0.095898127	0.5403233	-0.636025686	6.55E-05	-0.071939212	0.642222
NARF	0.002635537	0.9795028	-0.408166135	7.36E-05	-0.136833446	0.18213
CBLN2	0.128272456	0.7328261	-1.514712556	7.41E-05	-0.050183382	0.89376
ELF1	-0.234301931	0.0628592	-0.497223268	7.81E-05	-0.314140449	0.01249
RGL3	0.013688157	0.9349362	-0.666934184	8.01E-05	-0.45278678	0.007168
MBOAT1	-0.044921769	0.7802056	-0.638754814	8.28E-05	0.110504666	0.490054
EPB41L3	-0.085830543	0.7690609	1.131943602	8.92E-05	-0.021818977	0.940636
PYGO2	-0.175465191	0.2395185	-0.583996986	9.00E-05	-0.633097424	2.49E-05
FER1L4	-0.332148815	0.3059502	-1.263200415	0.0001092	-0.139289074	0.667363
CEBPZ-AS1	0.410597642	0.1475704	1.075626891	0.000112	1.306299947	2.73E-06
SIM1	-0.361082416	0.4121846	-1.738940531	0.0001169	0.095958814	0.82597

TMSB4XP6	0.23463737	0.1976222	0.699743891	0.0001182	0.738067784	4.91E-05
ETV1	0.331967706	0.1219728	0.818797079	0.0001264	0.089340725	0.677607
SPIB	0.600234556	0.1584459	1.606240995	0.0001264	0.730054007	0.084584
TSPAN14	-0.133984254	0.3984856	-0.608002262	0.0001283	-0.350975604	0.027195
ZDHHC18	-0.169009614	0.2182402	-0.524342928	0.0001419	-0.432452187	0.001658
MARK2	0.049146243	0.6882949	-0.465473797	0.0001627	-0.218259202	0.076061
SFMBT2	-0.040067267	0.861962	0.849683624	0.0001636	-0.175407041	0.445509
ARID5B	-0.152032681	0.251732	-0.500330966	0.0001659	-0.81199358	1.16E-09
ZNF416	-0.057048104	0.7266977	-0.62129365	0.0001838	-0.482622281	0.003586
CPNE3	-0.085965134	0.2815901	-0.297172803	0.0001888	-0.101254931	0.202907
FAT1	0.2226055	0.0993137	0.502924785	0.0001953	0.182777449	0.17593
TOB2	0.021619558	0.8240684	-0.362794844	0.0001975	-0.230375091	0.018031
FAR2	0.156622648	0.363363	0.633222869	0.0002083	-0.101519916	0.555386
ZNF846	-0.302712469	0.0635435	-0.604147182	0.0002093	-0.416759951	0.010483
TMEM219	-0.192330952	0.083113	-0.409736013	0.0002184	-0.129998689	0.23886
HPS6	-0.144093336	0.2241252	-0.438275795	0.0002273	-0.25040588	0.034324
C10orf55	0.761168388	0.0866165	1.53816633	0.0002353	2.025754009	8.77E-07
COL27A1	-0.137330988	0.3476225	-0.53750041	0.00024	-0.478586018	0.001073
TMEM101	-0.16382598	0.1726691	-0.441268655	0.0002558	-0.301477454	0.012054
RGS10	-0.004342382	0.9818831	-0.718217268	0.0002632	-0.146503183	0.444609
IKZF2	-0.074054944	0.6970933	-0.696402428	0.0002689	0.264953323	0.162304
CDH10	0.16431335	0.5658007	-1.079188472	0.0002739	0.064953947	0.82031
FGF7	0.260501824	0.3256655	0.959106411	0.0002828	-0.068548942	0.796086
TRMT2A	-0.04726407	0.7059589	-0.45537063	0.0002855	-0.314791869	0.012105
PWWP2B	-0.21764675	0.3244446	-0.811219926	0.0002916	-0.361225892	0.101986
ALB	0.529175004	0.4152668	2.322479423	0.0003198	2.839073576	1.07E-05
NME7	0.210299174	0.342213	0.791499912	0.0003266	0.297756612	0.177911
CCBE1	0.545161131	0.1979874	1.507142263	0.0003305	0.166339838	0.695925
MRPL54	-0.097694979	0.3974198	-0.416831879	0.0003395	-0.031160954	0.783468
TRIML2	1.066470034	0.161154	2.526483726	0.0003524	1.03498517	0.166397
ZNF551	-0.180732996	0.1922606	-0.495399574	0.0003542	-0.319870662	0.021332
SLC44A2	-0.014744669	0.9174646	-0.5083987	0.0003563	-0.03275153	0.817585
P11-736N17	1.561377834	0.0511731	2.81921142	0.0003665	2.801136139	0.000383
MAPK15	-0.494664373	0.0714111	-0.992727449	0.000369	-0.925612176	0.001002
USP38	-0.120262019	0.1484695	-0.295457557	0.0003716	-0.270918495	0.001136
BCL2	0.492449992	0.2112407	1.389232787	0.0003796	0.585038082	0.137524
ZEB1	0.325821204	0.1739075	0.846680344	0.0003985	0.208819906	0.383575
ZBTB34	-0.020657539	0.7954036	-0.281917202	0.0004066	-0.125707206	0.114116
WHAMM	-0.359483868	0.0591432	-0.675312074	0.0004095	-0.261950489	0.1688
FUT4	-0.03307108	0.8498636	-0.619407505	0.0004113	-0.177070623	0.310814
STON2	-0.032626524	0.8561439	-0.637124435	0.0004179	-0.178849058	0.320059
KIFC2	-0.040108689	0.8352315	-0.683621612	0.0004192	-0.267821978	0.164398
IP6K2	-0.073964017	0.3660255	-0.287760305	0.000434	-0.303132526	0.000216
MAMDC4	-0.257592867	0.1229534	-0.592050252	0.0004367	-0.613033068	0.000267
HDX	0.237865477	0.2950684	0.78874895	0.0004394	0.534315833	0.018122
CDKN2A	0.336378475	0.5241428	1.800070624	0.0004402	0.304682921	0.562673
MAVS	-0.08764105	0.2705624	-0.279225453	0.0004425	-0.292998949	0.000234

PLCD1	-0.291395272	0.1152443	-0.650484194	0.0004448	-0.715600204	0.000108
PLCH1	-0.155631229	0.4910326	-0.796005633	0.0004555	-0.242920909	0.281478
DEPTOR	0.033439502	0.8868685	-0.82434966	0.0004637	0.145929055	0.534374
FAM83E	-0.160237885	0.4307766	-0.715926714	0.0004646	-0.038683694	0.848471
LDLRAD3	0.375365666	0.1470391	0.891175235	0.0004712	0.177016701	0.490984
FAM156A	-0.295164047	0.893618	-8.485771056	0.0004772	-1.229990189	0.577675
STK38	-0.132792382	0.1136376	-0.29005997	0.0005331	-0.002164044	0.979285
HERC2P8	0.113035428	0.739358	1.161444602	0.0005464	-0.070985331	0.834575
C19orf33	-0.04287016	0.7907449	-0.560763359	0.0005492	-0.019854183	0.901984
MMD	0.12206291	0.4079254	0.504661341	0.0005509	-0.014029055	0.924186
LNX2	-0.274255655	0.0772913	-0.536041273	0.0005561	-0.278756766	0.072179
ITGA1	0.023258071	0.9062183	0.679206382	0.0005642	-0.136357604	0.489779
CD46	-0.131442113	0.1980644	-0.351133566	0.0005841	-0.136323333	0.181746
SLC23A2	0.236102481	0.0945219	0.481211238	0.0005843	0.266966482	0.057289
MED18	-0.177392118	0.1520184	-0.424316942	0.000594	-0.171808555	0.162353
PABPC1L	-0.119541665	0.5076297	-0.62437104	0.0005983	-0.35749919	0.050216
SEPHS1	-0.018902318	0.8552457	0.352780534	0.0005978	0.044768963	0.664653
HERC2P5	0.105034177	0.7569889	1.150477942	0.0006095	-0.081862869	0.809536
CLP1	-0.219102657	0.0775043	-0.419964199	0.0006658	-0.328020088	0.007922
CYP2W1	-0.230658054	0.463356	-1.079294145	0.0006751	-0.59089374	0.060238
CEP112	0.110007852	0.4569184	0.492454749	0.0006769	0.384733211	0.008863
LUZP2	-0.699213732	0.1351823	-1.614852193	0.0006792	-0.906265187	0.047922
LNX1	-0.071994843	0.7004176	-0.641126908	0.0006891	-0.356493559	0.057038
C16orf54	0.81232993	0.059628	1.363047096	0.0007191	0.46578794	0.291373
EXT1	0.133017934	0.259363	0.397045133	0.0007215	0.326429883	0.00552
SULF1	0.111043852	0.6967289	0.962483241	0.0007205	0.367994475	0.196339
HMOX1	-0.24539086	0.212691	-0.671417205	0.0007232	-0.689767862	0.000541
GC	-0.252292124	0.5337646	1.32562607	0.0007498	0.35117185	0.378281
C14orf79	-0.179054028	0.2590149	-0.532754994	0.0007682	-0.343302694	0.031534
ZFYVE21	-0.225821054	0.052275	-0.389919852	0.0007713	-0.222937185	0.054156
HTR2A	0.589800631	0.1216268	1.26326519	0.0007813	-0.314191059	0.418425
BZW1	0.062749288	0.4760245	0.295385946	0.0007846	0.462643353	1.44E-07
MAPKAP1	0.059775593	0.4807418	0.281814316	0.0007864	0.166394679	0.048403
KCNMA1	1.000804113	0.0761669	1.866362996	0.0008115	0.964776445	0.087603
MMP9	0.381310757	0.6943003	3.113757191	0.0008256	1.706419222	0.069998
DDX55	-0.217731559	0.0809128	-0.412648511	0.00085	-0.49110233	8.18E-05
TTC31	-0.013332229	0.8787228	-0.293722777	0.0008486	-0.395899513	9.40E-06
MIEF1	-0.184851843	0.097815	-0.370433307	0.0008882	-0.563200024	5.08E-07
PAN2	-0.266571338	0.1433701	-0.606142753	0.0008945	-0.487349849	0.007479
PDCD5	0.040424961	0.7194496	0.368721459	0.000906	0.201110884	0.071938
KIAA1147	-0.136128685	0.1042336	-0.276997119	0.0009134	0.017522953	0.832939
ACAP3	-0.149515488	0.4326315	-0.633476993	0.000923	-0.486483647	0.010671
KIF21B	0.257596902	0.2100003	0.666473738	0.0009246	0.810134214	5.50E-05
PLA2G4F	0.044373187	0.9006647	-1.177503559	0.0009274	0.206692195	0.554388
MFSD6L	0.028915333	0.9075249	-0.855027914	0.0009453	0.002345799	0.992441
SLFN13	0.210177757	0.4853765	0.993112927	0.0009508	0.855308156	0.004376
WBP1L	-0.07924495	0.3040382	-0.253995881	0.0009555	-0.075920139	0.321842

MAP6	0.71275637	0.2379241	1.842310782	0.0009691	1.208290364	0.044898
ABCF3	-0.204149438	0.102221	-0.4120531	0.0009805	-0.19178406	0.12407
KALRN	-0.207193801	0.1258755	-0.44517454	0.0010055	-0.201802115	0.135571
C1orf174	-0.12151017	0.1826226	-0.298205313	0.0010295	-0.249540883	0.006201
DAPK1	-0.059035059	0.7588261	-0.631641423	0.0010348	-0.0730599	0.70392
RHOQ	0.211103011	0.2143081	0.554168075	0.0010366	0.175844955	0.299437
ZNF417	-0.117128303	0.3835109	-0.440516908	0.001047	-0.312560137	0.020607
TPM1	0.223934924	0.0990966	0.444584643	0.0010496	0.445982317	0.001014
ERLIN1	-0.00120536	0.9943949	-0.562725594	0.00106	-0.215800941	0.208577
XRCC5	0.05625623	0.6489087	0.403981239	0.0010695	0.299691177	0.015235
CD44	0.68416828	0.0530788	1.155756192	0.0010724	0.871443697	0.013701
SNCAIP	-0.026435311	0.9149781	0.801138863	0.0010865	-0.071952944	0.771855
ZSWIM1	0.107408235	0.3375342	-0.36838686	0.0010984	-0.236060257	0.037027
TPD52	0.008252154	0.9555539	-0.481534321	0.0011443	0.235092393	0.111327
CLDN9	-0.44650832	0.1331409	-0.993539832	0.0011601	-0.817576534	0.007074
STYXL1	-0.013102253	0.9116394	-0.384226255	0.0011739	-0.059964614	0.609244
CTDSP1	0.037335333	0.7227332	-0.342035821	0.0011771	-0.020411138	0.846053
FANCG	-0.349214092	0.0739258	-0.631608694	0.0011809	-0.502807276	0.009988
USP48	-0.166390588	0.1700752	-0.392215947	0.0011931	-0.318400716	0.008556
EPOR	-0.038080291	0.8175769	-0.541973984	0.0012018	-0.39866105	0.01688
SUMO1	0.052029714	0.6674439	0.390541704	0.0012062	0.348971482	0.003847
CTC-471F3.4	1.8467305	0.1036683	3.570008732	0.0012103	3.459681913	0.001739
CARKD	-0.144626297	0.1568694	-0.329998729	0.0012214	-0.336863126	0.001021
PTPN11	0.138937827	0.1418084	0.305111704	0.0012275	0.265576802	0.004894
ZNF581	-0.23497764	0.1878909	-0.574969293	0.0012673	-0.50526979	0.004772
C1orf106	-0.033496751	0.8143782	-0.460046853	0.0012773	-0.163044094	0.252667
ECE1	0.456960732	0.0579394	0.77528762	0.0012821	0.755190581	0.001713
FN3KRP	-0.135567243	0.188608	-0.330791818	0.0013026	-0.265950935	0.00991
C1orf116	-0.228356767	0.2291901	-0.610325589	0.0013189	-0.09274256	0.624999
KIF13B	-0.113951216	0.3817688	-0.419034579	0.0013173	-0.065840731	0.613116
RP11-61L23.	-0.364655832	0.1477009	-0.810883869	0.0013386	-0.167487532	0.493384
ARHGEF10L	-0.088360818	0.6175493	-0.567230548	0.0013582	-0.307232925	0.082688
COLEC12	0.203361873	0.4276424	0.816976708	0.0013645	0.381246407	0.136188
DDIT4	0.040715474	0.8875915	0.919861591	0.0013633	1.220453673	2.14E-05
TM9SF4	0.147207311	0.0902495	-0.27896526	0.0013635	-0.191199961	0.028142
ZNF20	-0.402485666	0.1348137	-0.87158746	0.0013856	-0.677786704	0.013018
TMEM184A	-0.325647257	0.0735478	-0.581011343	0.0013901	-0.32582527	0.072712
FAM179A	0.082930837	0.698636	0.663340436	0.0014349	0.331865184	0.11598
NBEAL2	-0.165597197	0.2936974	-0.502315763	0.0014596	-0.154485792	0.327052
PTPRR	-0.478527528	0.1099878	-0.953899462	0.0014586	-0.84214883	0.004966
ME3	-0.088673261	0.5691683	-0.498124059	0.00147	-0.09239261	0.549911
MAP3K14	-0.307998641	0.061069	-0.52193725	0.0014775	-0.578753685	0.000499
CWC22	-0.147194234	0.0762001	-0.261940177	0.001485	-0.166513675	0.043463
TTI1	-0.044498268	0.6620121	-0.323553741	0.0014877	-0.374466695	0.000263
C15orf52	-0.04933889	0.8924584	1.09808932	0.0014899	0.769277052	0.026395
RNF34	-0.162404932	0.1466864	-0.353326109	0.00154	-0.23249032	0.03752
RP11-551L14	0.355193516	0.473059	1.54975037	0.0015412	-0.26593462	0.593405

ELMO3	-0.110245356	0.3736417	-0.392925326	0.0015444	-0.083526567	0.496114
TMEM9	0.054434976	0.5849955	-0.316488291	0.0015565	-0.025803064	0.795325
MEPCE	0.019034077	0.8668908	-0.359780739	0.0015827	-0.294922352	0.009678
DNAJB6	0.153864656	0.1273229	0.31694838	0.0015929	0.373921637	0.000197
CRELD1	-0.104573851	0.5636425	-0.572178035	0.0016191	-0.504915588	0.005413
APLF	0.015988432	0.918449	0.47777706	0.001636	0.185931932	0.227374
JTB	-0.160853017	0.1178756	-0.322904061	0.0016468	0.067894104	0.504403
C5orf63	-0.390605355	0.3682726	1.291340546	0.0016548	0.048676337	0.90922
WASL	-0.117260424	0.1453109	-0.252529816	0.0016692	-0.132200423	0.099665
PFKFB4	-0.397966177	0.1617229	-0.894917835	0.0016771	-1.175038719	3.78E-05
PRKAB1	-0.18321689	0.105986	-0.356440222	0.0016807	-0.349128871	0.002141
VPS4A	-0.12889947	0.1073862	-0.249802849	0.0017166	-0.200173315	0.012032
WDR53	-0.353875845	0.1717385	-0.807126974	0.0017289	-0.464275408	0.073513
TNMD	0.501358029	0.481779	2.209586907	0.0017323	0.765527402	0.281062
FAM110C	0.479124113	0.0560436	0.774305262	0.0017789	1.17310136	1.95E-06
MYH11	0.930177912	0.0858393	1.689772724	0.0017811	0.578684717	0.285682
SRC	-0.147239936	0.2678909	-0.415041484	0.0017978	-0.162793011	0.219808
KHDRBS3	-0.083571635	0.7835446	0.936320902	0.0018005	-0.023892473	0.937459
TCF12	0.212526845	0.1273653	0.433701955	0.0018227	0.019097874	0.890994
ANAPC16	-0.126952073	0.2430156	-0.337830609	0.0018427	-0.146361465	0.176254
RCC2	0.202553699	0.0556106	0.32733499	0.0018687	0.395894886	0.000169
RNF223	-0.487628049	0.0746741	-0.856642678	0.0018829	-0.126784877	0.63509
TM9SF1	-0.047424008	0.6024691	-0.282327517	0.0018828	-0.124546088	0.169153
PDXDC1	-0.102133943	0.283405	-0.295045384	0.001935	-0.085026232	0.37105
P11-460N11	0.469647155	0.0691797	0.794200238	0.001976	0.417587329	0.105694
GANAB	0.14904148	0.0937016	-0.275495275	0.0019805	-0.196290276	0.027544
DCAF5	-0.153502072	0.0751665	-0.265051076	0.0019952	-0.243515305	0.004593
RALGPS2	-0.165819671	0.2391815	-0.434615849	0.0020039	-0.201700544	0.151637
TAOK2	0.0349283	0.7182565	-0.299682761	0.0020042	-0.218221366	0.024524
RPS12P26	0.560023083	0.2722679	1.455367919	0.0020083	1.860740972	6.19E-05
HDAC10	-0.185421175	0.3998817	-0.686335924	0.002021	-0.433920351	0.050242
TRIM36	0.651584705	0.1415828	1.359915679	0.0020248	1.45186737	0.000758
UCP2	0.30571271	0.2897162	-0.901988517	0.0020582	0.159751739	0.58062
CBWD2	0.207717945	0.0789382	0.359273572	0.0021342	0.272725254	0.020343
SLC23A1	-0.523399664	0.3390482	1.478261054	0.0021658	0.012946271	0.980173
TRIP12	0.022339102	0.805312	0.277433578	0.0021695	0.274741098	0.002399
HTN3	0.16256266	0.5510971	0.811324991	0.0021984	0.309108792	0.251168
POLL	-0.072863392	0.5839599	-0.409449567	0.0022012	-0.290023451	0.029554
FAP	-0.181859354	0.7558995	1.75288152	0.0022229	0.921042903	0.110684
KLHL12	-0.13319322	0.1872393	-0.30681332	0.0022209	-0.158858025	0.113271
SGSM3	-0.01851532	0.8874126	-0.401505079	0.0022227	0.02728639	0.834637
PCDH20	0.02963267	0.8983264	-0.715974841	0.0022272	-0.468406746	0.044756
C9orf172	-0.283703483	0.2224904	-0.722531796	0.002234	-0.479604102	0.040044
MTUS2	0.998793941	0.1519515	2.097716749	0.0023058	-0.090348668	0.897276
SULF2	0.047161423	0.7578646	0.465298266	0.0023161	-0.379149832	0.013305
DNALI1	-0.246816661	0.1120864	-0.472666434	0.0023206	-0.362884485	0.019534
ARAP1	-0.014067623	0.923765	-0.448567292	0.0023338	-0.391629597	0.007871

CREBZF	-0.058351635	0.5463274	-0.293614741	0.0023625	-0.102225039	0.289406
SALL2	0.925963038	0.0677518	1.514930802	0.0023623	0.723665334	0.14927
CLMP	0.20643652	0.422649	0.77908793	0.0023981	0.422184857	0.100644
KRT6B	1.52888667	0.1635497	3.32507437	0.0023934	2.830273733	0.009773
RFX6	-0.807984485	0.1485082	-1.840683094	0.0023967	-1.947170883	0.001756
CCDC85C	-0.046638963	0.7585959	-0.459262475	0.0024927	-0.086384424	0.568977
CDC42SE1	0.083027739	0.4163865	-0.309354556	0.0024928	-0.027683174	0.786391
KRT18P59	0.587006671	0.3009948	1.609420941	0.0025095	1.007052569	0.066486
ZNF263	-0.158751375	0.0929925	-0.285122339	0.0025133	-0.081406688	0.3876
GRHL2	-0.250711542	0.1140955	-0.478576426	0.002537	-0.294057101	0.06301
ATP9B	0.178286006	0.3244358	0.544215962	0.0025476	0.1198012	0.507576
SNAI1	0.146445439	0.5053428	0.644926632	0.0025761	0.225114186	0.301309
TMEM38A	-0.282592563	0.2555091	-0.750799783	0.0026042	0.078761098	0.749615
MKX	0.797612496	0.2949648	2.232165833	0.0026245	0.766135313	0.312861
PDGFA	-0.398560524	0.0805794	-0.685587969	0.00263	-0.254422065	0.259722
TOP3B	-0.175182199	0.1357294	-0.351667948	0.002645	-0.369822662	0.001663
RUNX1T1	0.621415192	0.068192	1.022194507	0.002667	0.26899733	0.430446
NAA38	0.022122792	0.8803799	0.437807038	0.0026911	0.483190441	0.000936
ABO	-0.538111362	0.0612757	-0.864568619	0.0027087	-0.26252254	0.356359
MMP13	0.00200678	0.9981575	2.480665419	0.0027286	3.642732391	9.75E-06
SUSD5	-0.164184529	0.6816426	1.118090497	0.0027266	-0.349878128	0.383744
C4BPB	0.350239673	0.2134949	0.820106426	0.0028317	-0.120010535	0.67302
CAB39L	-0.273463706	0.1154838	-0.519109616	0.0028742	-0.112468761	0.516058
SRSF3	0.229884158	0.0841915	0.396216361	0.0028711	0.468179569	0.000428
RITA1	-0.232165485	0.0856031	-0.399773273	0.0029253	-0.26665208	0.046452
POLH	-0.199559449	0.1940541	-0.455056007	0.0030017	-0.317570829	0.038192
ALKBH4	-0.213872098	0.2826067	-0.591315255	0.0030101	-0.593669365	0.003196
EEF1DP3	-0.054462186	0.8634981	-0.967889716	0.0030165	0.052479201	0.865977
SSXP10	-0.215147026	0.4048775	-0.769773916	0.0030177	-1.005452779	0.000118
DHX29	0.188505113	0.2222206	0.455890224	0.0030254	0.305029818	0.047698
KCND3	0.58269293	0.1183428	1.097703996	0.0030656	0.12061883	0.747312
PAGR1	-0.176090573	0.0880724	-0.304306864	0.0030413	-0.220628551	0.031599
P11-726G1	0.288812803	0.2026796	0.655270056	0.0030448	0.251528133	0.264936
SCGB3A2	-0.008010506	0.9909181	2.02088695	0.0030456	-0.157688796	0.82259
SEPSECS	0.286699908	0.1077065	0.519589428	0.0030561	0.416839704	0.018687
SPRN	-0.050852511	0.8394399	-0.780054049	0.0030627	-0.230747943	0.359666
USP21	-0.103361176	0.5265625	-0.486699083	0.0030588	-0.588442557	0.00042
ARHGAP22	0.316883919	0.2663486	0.825409851	0.0030895	0.223774258	0.431798
TRMT5	-0.256719293	0.1692859	-0.549696311	0.0031028	-0.212690516	0.253825
RASSF4	0.284561061	0.1958704	0.641927631	0.0031418	-0.073930681	0.737572
PLXNA4	0.450282862	0.2125625	1.049895983	0.0031568	0.704270385	0.051182
NCOA6	0.090782104	0.5663674	-0.46893973	0.0031674	-0.121886748	0.441928
PDE4D	0.297277956	0.1011228	0.533664926	0.0031767	0.598074986	0.000944
CCDC6	-0.196409391	0.0692505	-0.318481435	0.0031818	-0.172486361	0.10975
CYP4V2	0.168129788	0.4955509	0.715086907	0.0032238	0.241165471	0.326651
C1orf210	-0.377473445	0.0729014	-0.619449481	0.003253	-0.486813111	0.020572
PROC	-0.212772021	0.4004052	-0.7490777	0.0032881	-0.445721996	0.077282

ZBTB25	0.213224489	0.188057	0.470315192	0.0033023	0.383364062	0.017067
GOLGB1	-0.033147272	0.6125643	-0.192163891	0.0033101	-0.005454565	0.933493
TCTN1	-0.015309981	0.9014	0.357763492	0.0033318	0.057940739	0.637443
GRASP	0.562730657	0.0616485	0.862766705	0.0033784	0.672258216	0.024051
BMPR1B	-0.06117745	0.8288132	0.817737446	0.003393	0.340040316	0.226181
C12orf23	-0.171409034	0.111642	-0.3151205	0.0034203	-0.214428371	0.046368
HNRNPC	0.00270114	0.9775352	0.280446068	0.0034281	0.340111	0.000387
RFNG	-0.314506307	0.1194866	-0.589781938	0.0034347	-0.204069908	0.310337
PIGV	-0.092366398	0.4621764	-0.367858254	0.0034461	-0.100436388	0.421504
ZFP28	-0.239957798	0.195434	-0.541972026	0.0034708	-0.378081418	0.040946
ERLEC1P1	0.068259537	0.8501429	0.958422987	0.0035311	-0.006743638	0.985008
SPIRE2	0.082357729	0.6036825	-0.465335836	0.0035354	-0.133697295	0.400613
CCDC80	0.011405655	0.9666634	0.7942587	0.0035401	-0.35262771	0.196662
RAB14	0.012944004	0.8893238	-0.271458438	0.0035639	-0.100080191	0.281812
CCDC74A	0.09869308	0.7055279	0.737673114	0.0035898	0.031066081	0.90484
RAB3A	-0.712840675	0.0930041	-1.239562181	0.0036168	-1.095153198	0.009179
SLC35A1	-0.188139272	0.0661099	-0.295741287	0.0036303	-0.122051735	0.228198
CTSC	-0.098631411	0.5427079	0.469056912	0.003734	-0.238474364	0.141084
XRN1	0.247836197	0.1880525	0.544758975	0.0037348	0.283903682	0.131004
.RRC37A16I	0.083401323	0.5950017	0.452004674	0.0037431	0.453365579	0.003587
CCNT2	0.095906526	0.2618174	0.245364345	0.0038049	0.247171022	0.003642
SPICE1	0.233719455	0.126156	-0.441832537	0.0038045	0.194983367	0.200553
NAV3	-0.110607663	0.5451375	0.52314967	0.0038696	0.151103213	0.406246
SMIM6	-0.242039081	0.3141166	-0.697650541	0.0038691	0.131420873	0.580623
ADCY5	0.471860213	0.1416382	0.922327347	0.0039041	0.090616402	0.780757
FKBPL	-0.274775492	0.1282884	-0.52192501	0.003907	-0.266176699	0.135175
FBXL14	-0.233083642	0.308261	-0.663452103	0.0039464	-0.096961184	0.670305
GOLGA1	0.074531199	0.7246411	-0.610366245	0.0039444	-0.439427633	0.037542
TMEM170A	-0.169028158	0.0838499	-0.280572622	0.0040068	-0.138867165	0.154447
ZDHHC20	-0.095963025	0.2811295	-0.255659886	0.0040591	0.024488311	0.782824
SMG9	-0.063447126	0.5685235	-0.317262698	0.004076	-0.255967142	0.021102
RTN3	-0.08934073	0.3653448	-0.2831508	0.004102	0.004453595	0.963884
PBX3	0.237272919	0.2891008	0.638450397	0.0042058	-0.031491233	0.888265
ZNF783	-0.053301062	0.7773401	-0.539170548	0.004206	-0.283698194	0.132921
ENO1P4	-2.203873379	0.1719676	-5.27521894	0.0042398	-1.515903303	0.337475
IFIT1	0.09367668	0.7559594	0.851372295	0.0042648	0.409138551	0.171786
ZNF256	-0.243829229	0.0634148	-0.37137702	0.0042691	-0.422147423	0.001336
C19orf10	-0.087334193	0.5021989	-0.372260211	0.0042765	-0.013010791	0.920073
SLC30A3	0.335237107	0.4790378	1.29770288	0.0042809	0.101468034	0.831288
IGFBP7	-0.11592579	0.5710704	0.582828735	0.0042904	0.473564622	0.020361
SRSF5	-0.195680726	0.0784383	-0.317320773	0.0043061	-0.251695391	0.023569
PEX13	-0.167377027	0.0946324	-0.285145326	0.004332	-0.237814455	0.017379
NDUFA4L2	-0.088908398	0.8396486	-1.261353198	0.0043672	-0.332435599	0.449535
TMEM41B	-0.143903447	0.121005	-0.263687586	0.0044073	-0.22530777	0.015102
NFIL3	0.328592929	0.1509245	0.647004351	0.0044916	0.82289435	0.000299
C11orf35	-0.169505779	0.5480702	-0.819737658	0.0045375	-0.456448298	0.105357
KCNK3	0.412610658	0.2824587	1.049282099	0.0045597	0.107798295	0.780882

CXXC1	-0.231522046	0.0924295	-0.389900105	0.0045843	-0.381931814	0.005445
MAK	0.34725092	0.2319195	0.770437522	0.0046004	0.620274146	0.027581
NAGPA	-0.370859892	0.1205246	-0.674931955	0.0046195	-0.400196591	0.092702
IFT57	0.045228749	0.7421979	0.385573634	0.0046606	0.38636724	0.004598
IRF9	-0.242562779	0.1227885	-0.444717798	0.0046734	-0.184724443	0.238895
P11-848G14	0.440233292	0.5313979	1.911972714	0.0046818	1.489598474	0.029179
SLITRK6	-0.013387151	0.9614161	-0.783838357	0.004679	-0.368239205	0.18352
GTF2H2B	-0.306557085	0.0554064	-0.451703308	0.0047195	-0.347139209	0.029872
ZNF542	-0.038945074	0.8300343	-0.511986809	0.0047204	-0.491248684	0.007025
ABCA8	-0.373466756	0.6054449	1.985309187	0.0047459	1.71451735	0.014799
GCC1	0.124143712	0.2684503	-0.318571485	0.004742	-0.028480396	0.799653
SUB1	-0.036461613	0.6750841	0.244608204	0.0047468	0.206614219	0.017186
SMARCC1	0.100013307	0.2384689	0.238682637	0.0047884	0.050916872	0.548131
ZBTB2	-0.11791657	0.2046356	-0.260968415	0.004792	-0.248748609	0.007425
PCDHB2	0.218168239	0.1526433	0.4269245	0.0048118	0.017404068	0.909261
PIK3AP1	0.608124867	0.0835487	0.988343263	0.0048385	0.429931929	0.220854
STOX2	-0.281157064	0.3133764	-0.781098061	0.0048391	-0.198119601	0.474098
11-Sep	0.161078511	0.1681599	0.328506249	0.0049077	0.225563488	0.053508
ABCC4	0.105895603	0.6012794	0.565432505	0.0049316	0.029146675	0.885365
MAP4K5	0.18233396	0.0630877	0.274292748	0.0049719	0.252632613	0.009729
IRF2BP2	0.219655845	0.121967	0.397320484	0.0050494	0.271353441	0.055727
BTBD1	-0.05232332	0.5583302	0.249052073	0.0050541	0.171753773	0.053518
CABLES1	-0.436305127	0.0716882	-0.677561896	0.0050895	-0.287974315	0.229742
MPP1	0.101723007	0.6284189	0.571274908	0.0050748	-0.535691704	0.012576
PGRMC2	-0.304817187	0.0799959	-0.4873054	0.0050793	-0.259234473	0.135873
RPF2	0.150588446	0.3213978	0.423760082	0.0050852	0.531546061	0.000441
HSCB	-0.123328267	0.3627275	-0.379022261	0.005103	-0.299090046	0.026502
SYTL5	-0.255552682	0.1440798	-0.489380211	0.0051497	0.032425246	0.852429
SH3BGRL	0.053825594	0.5524334	0.251807045	0.0051842	0.164866955	0.067979
PDE4DIP	-0.297451982	0.0519775	-0.4259627	0.0052337	-0.386161448	0.011503
VILL	-0.121196197	0.4950284	-0.495995179	0.0052545	0.062767895	0.723451
CLCN7	0.070407914	0.6239008	0.394971094	0.0053537	0.084545486	0.556887
EFCAB4A	-0.098056914	0.6410525	-0.593237965	0.0053543	0.179153884	0.391199
BTBD8	-0.264311116	0.4599642	-1.059348279	0.0053759	-0.691911304	0.061453
ZNF222	-0.381028163	0.0647476	-0.568710165	0.0054512	-0.447343464	0.028596
KLHL28	-0.200608487	0.1254477	-0.361355948	0.0054562	0.002656143	0.983677
HHLA1	0.048229144	0.9257342	1.419594003	0.0054825	0.087964851	0.864713
TIGD5	-0.280637311	0.1023569	-0.475279575	0.0054935	-0.58219253	0.000801
FAM219B	-0.17575847	0.1878656	-0.369106782	0.0055142	-0.350394524	0.008532
SLC26A6	-0.280053943	0.1064241	-0.480025243	0.005556	-0.331593521	0.056169
TMTC2	0.193482717	0.3242767	0.542879367	0.0055586	-0.118000775	0.548176
CBWD5	0.234709693	0.0843794	0.374690108	0.005565	0.266931019	0.048662
CCNDBP1	-0.145009816	0.2659912	-0.361274652	0.0055728	-0.074991784	0.563106
ELF5	-0.30043811	0.2560522	-0.731987338	0.0055748	-0.828502745	0.001906
KTI12	-0.266205809	0.0662091	-0.398249522	0.0055822	-0.448504492	0.002045
KDM4C	-0.205597601	0.1707692	-0.415633332	0.0056086	-0.227750892	0.128751
MEIS1	0.293660289	0.23129	0.678400154	0.0056402	0.068938236	0.778706

UBA2	0.074513752	0.426846	0.258795081	0.0056536	0.360130617	0.000118
WLS	-0.061811724	0.6290326	-0.354350707	0.0056655	-0.165494209	0.195595
IFI44	0.766798406	0.0773081	1.197621942	0.0056778	1.184883339	0.005844
PCNX	0.120417502	0.3986784	0.393905199	0.0056948	0.158208289	0.267305
PPIAL4G	0.424387024	0.4038557	1.362310932	0.0056892	-0.044394866	0.931361
TMEM87A	-0.171787799	0.1065589	-0.293714104	0.0057155	-0.013450281	0.898989
TRANK1	-0.398352335	0.0514088	-0.565418059	0.005714	-0.867231194	2.57E-05
OPHN1	-0.112092717	0.5476929	0.511291589	0.0057391	0.209233282	0.259876
PCYT1A	-0.154348101	0.1263704	-0.277481051	0.0057429	-0.256620908	0.010751
TBC1D10B	0.152632006	0.0914488	-0.25064158	0.0058215	-0.186226825	0.041033
PARP4	0.102957144	0.1671853	-0.205549867	0.0058661	0.050852122	0.494773
ECHS1	-0.176076857	0.0806591	-0.276526546	0.0058849	-0.104963753	0.294891
VIPR2	0.314197105	0.5469035	1.415765395	0.0058815	0.313164784	0.548776
CORO6	0.745877033	0.1325904	1.343675291	0.0059045	1.448183888	0.003153
VSTM4	-0.01078449	0.9653499	0.667930968	0.0059333	0.232051379	0.343874
C2orf47	-0.041932038	0.8075166	-0.475695955	0.0059458	-0.604941667	0.000465
USP19	0.010681492	0.9335083	-0.351209501	0.0059523	-0.430288132	0.000785
ASAP1	0.361104671	0.0632767	0.532766249	0.0059869	0.073474609	0.705553
RIMKLA	-0.357175918	0.1931593	-0.756686133	0.00599	-0.56677243	0.039513
ANTXR1	0.149288581	0.4576348	0.55011875	0.0060026	0.22769767	0.257066
EEF1A1P6	-0.265300623	0.209375	-0.580974789	0.006016	-0.196783463	0.350906
EWSR1	0.172053012	0.0973905	0.28468359	0.0060382	0.367578702	0.000393
HSPA1L	8.98E-05	0.9998966	-1.926003107	0.0060438	0.233775788	0.73561
PPP4R1	0.003057883	0.9771028	0.291514724	0.0060499	0.285055621	0.007243
BLOC1S3	0.037206574	0.8448172	-0.527316043	0.0060638	-0.307329423	0.109742
CSNK1G1	-0.135825028	0.1693272	-0.270431156	0.0060689	-0.187224513	0.057425
AKR1D1	0.041953966	0.9519606	1.789056252	0.0060737	-0.095615965	0.890779
ALAD	-0.322638606	0.0705681	-0.489058878	0.0060877	-0.249140049	0.162085
POLR3H	-0.239295275	0.067759	-0.357413973	0.0060862	-0.229230098	0.078647
HTN1	0.227558428	0.4051779	0.737721326	0.0061708	0.335537651	0.217034
TRIM26	-0.049473634	0.646842	-0.295792261	0.0061782	-0.137858499	0.201392
GAS2	0.103065501	0.83763	1.366585163	0.0062028	0.477475471	0.340629
NCAM2	0.505785575	0.1562324	0.972344388	0.0061982	0.327309341	0.358317
ADCK3	-0.182516994	0.3378024	-0.521205973	0.0062302	-0.202690623	0.286587
ADAMTSL3	-0.034027942	0.8660701	0.549405816	0.0062895	0.048609973	0.809432
SLC25A14	-0.229700102	0.18481	-0.477216684	0.0063119	-0.349829981	0.0426
P11-950K24	1.046688662	0.0588669	1.450410964	0.0063483	1.031837819	0.060171
SLC6A10P	1.074416068	0.0999139	1.680701386	0.0063629	1.503156471	0.01778
NUB1	0.197796359	0.0528063	0.277494872	0.0064031	0.343965087	0.000709
NUGGC	0.424262283	0.1257093	0.730276604	0.0064012	0.54087636	0.047026
RPL9P30	1.085663246	0.1044505	1.732708356	0.0063865	1.501774641	0.020014
MEOX2	0.579583984	0.4226857	1.951407783	0.0064882	0.71409585	0.322184
RSRC2	0.091817754	0.5018295	0.370977397	0.0064885	0.402311135	0.003174
SLC7A5	0.453610601	0.1063164	0.763162983	0.006499	0.907098067	0.001215
HACE1	-0.156828194	0.266278	0.377963634	0.0065229	-0.068016082	0.629239
MID1	0.24663783	0.2236659	0.546770365	0.006529	0.050033643	0.804444
MITF	0.058756807	0.7429894	0.479434925	0.006529	0.040675698	0.819586

USPL1	-0.019944882	0.8428922	-0.273690942	0.0065684	-0.134928295	0.179306
AAR2	0.089799751	0.3843805	-0.282180957	0.006625	-0.236029269	0.023488
ENTPD7	-0.099548909	0.3402587	-0.281763894	0.0066244	-0.189167172	0.068547
GLIPR1	0.513719958	0.0984239	0.8428573	0.0066052	0.258838741	0.405101
LYRM1	0.266377435	0.0922305	0.425833457	0.0065992	0.33398712	0.034358
NEDD4	0.03403925	0.8528591	0.497896107	0.006614	0.157426265	0.390818
ZNF564	-0.324287782	0.0623102	-0.471138156	0.0066162	-0.460862961	0.00816
PLXDC1	-0.470780587	0.0754213	-0.721523828	0.006677	-0.710623196	0.007615
KIAA2018	-0.083515769	0.5810332	-0.410563494	0.0066889	-0.300758041	0.046975
GALNT7	-0.174999645	0.1886367	-0.360167778	0.0067798	0.039660004	0.765077
CD40	1.081679862	0.0506422	1.474374486	0.0067867	1.588703216	0.003494
C9orf64	0.682149628	0.0970787	1.071127257	0.0068522	0.374524151	0.36771
SHANK2	-0.263780275	0.2270827	-0.588530389	0.0069793	-0.393851073	0.070681
MSR1	0.35595753	0.4425982	1.232791016	0.0070044	0.483748188	0.299199
ADO	-0.127606086	0.0949133	-0.204104552	0.0070508	-0.195480989	0.010212
AKAP5	-0.006689872	0.9733866	0.530499919	0.0070471	0.084834898	0.671696
FAM76B	-0.185306007	0.1938427	0.378887921	0.0070793	0.021042474	0.881855
IPO9	0.147725955	0.2777525	0.365718632	0.0071156	0.27250514	0.045151
AFF3	0.033204356	0.8987538	0.697689519	0.0071754	0.025870314	0.921187
FOXP2	0.266983681	0.3810369	0.818073193	0.0071748	-0.136670031	0.654207
MAPK8IP2	0.245486123	0.5501615	1.013923586	0.0072536	-0.078685204	0.850833
FBXO42	-0.105904041	0.5081638	-0.429440864	0.0073034	-0.356194132	0.026557
BHLHE41	1.147575227	0.0667443	1.666112526	0.0073464	1.314385644	0.03459
COG5	0.114463753	0.3182233	0.305685325	0.0073379	0.423229564	0.000208
EME2	-0.159135288	0.2430703	-0.364610748	0.0073468	-0.369034825	0.006979
KIAA1432	-0.045710492	0.6341203	-0.257011474	0.0073483	-0.267743409	0.005371
NTRK3	0.00338621	0.994091	1.194494342	0.007357	0.20451884	0.650587
EXOC8	-0.014173373	0.8579106	-0.211629888	0.0073822	-0.185156993	0.019549
MDFIC	0.222675076	0.3481991	0.633556244	0.0073792	0.286845441	0.226079
SNRPGP15	1.061933442	0.0650351	1.530903483	0.0073919	1.386429517	0.015481
HOXA11	-0.094994851	0.8271915	1.149213047	0.0074051	-0.331953902	0.446203
CCDC137	-0.161239127	0.3364542	-0.450178053	0.0074694	-0.378754003	0.024358
FGF11	-0.011297547	0.9644214	-0.679614302	0.0074808	-0.685806446	0.007066
MRPL50P2	1.199747619	0.0801832	1.81829223	0.0074753	1.824749199	0.007307
GABRP	0.305534434	0.6376125	1.714650911	0.0074896	0.003101795	0.996173
RAB33B	-0.043552274	0.7213699	-0.329291673	0.0075015	-0.251335981	0.040589
VPS13B	0.23972353	0.2449917	0.550589509	0.0075175	0.14551957	0.480262
XRCC3	-0.290937499	0.1169068	-0.493483254	0.0075607	-0.198378508	0.283785
CSMD3	-0.370411746	0.3046509	0.954333704	0.0076065	-0.140425599	0.697484
IARS2	-0.058465383	0.5379577	-0.253109745	0.0076229	-0.008394874	0.929352
ANXA2P1	-1.001097406	0.06441	-1.488967876	0.0076448	-0.60332746	0.239368
LITAF	0.35737838	0.0542311	0.494148048	0.0076663	0.295091954	0.111856
TOX	0.410766741	0.1158155	0.690034378	0.0076679	0.46498649	0.073919
FHOD3	0.482004066	0.4122647	1.473364593	0.0076756	0.87699848	0.113189
PITHD1	-0.181486947	0.0565734	-0.252118104	0.0077132	-0.124759938	0.186839
DBN1	0.124857471	0.5761592	0.594157801	0.0077368	0.226021296	0.311387
LRGUK	0.772551507	0.1787251	1.442966729	0.0077381	0.679885622	0.220724

ZNF696	-0.319042254	0.1928266	-0.6528909	0.0077421	-0.753659659	0.002415
ELOVL5	0.254395047	0.1041722	0.415950681	0.007752	0.133701924	0.393062
NCOA5	-0.04562245	0.7419012	-0.369161862	0.0077689	-0.327061227	0.018452
PARP1P1	0.479911417	0.1463241	0.837667749	0.0077704	0.735951877	0.021196
CD4	-0.293802718	0.3375391	-0.833094849	0.0077926	-1.289692062	8.42E-05
AC010980.2	0.664454158	0.0912898	1.028051243	0.0078243	0.333569189	0.402961
SNAPC1	0.185366179	0.2814379	0.449000902	0.0078249	0.71349158	2.16E-05
ZNF330	-0.185625441	0.0687086	-0.269631624	0.0078485	-0.03093238	0.758885
FAF2	-0.020672621	0.7846823	-0.200775189	0.0079046	-0.075153338	0.319518
P11-355I22	-0.568280776	0.0735578	-0.844043601	0.0079033	0.244117482	0.437126
G6PC	0.290429194	0.6373366	1.457861611	0.0079248	0.40403786	0.495582
GLTP	0.056286428	0.5908588	0.27471011	0.0079228	0.26350872	0.010876
IMPDH1	-0.325581871	0.0923699	-0.513880454	0.007914	-0.048414103	0.801989
ARFGAP2	-0.158756262	0.0720427	-0.233042865	0.0079645	-0.293374243	0.000847
IRS1	-0.137657753	0.3477565	-0.389035295	0.0079682	-0.455005205	0.001956
ITGBL1	1.225504733	0.2855161	2.973103338	0.0079584	2.757086411	0.014317
NR2F6	-0.269339359	0.0658064	-0.388045714	0.0079669	-0.049614623	0.7339
TDRKH	-0.38521147	0.0776021	-0.579448129	0.0079813	-0.388049907	0.074961
ZDHHC12	-0.168489685	0.3027577	-0.433888072	0.0079922	-0.007157406	0.96464
P11-402K9.	0.957977024	0.0780681	1.405494425	0.0080132	0.619647384	0.258929
KIAA1522	0.012471063	0.9475257	-0.503226579	0.0080283	-0.209990011	0.268049
TRAPPC2L	-0.132812579	0.2663923	-0.315766085	0.0080517	0.054278658	0.645278
METTL15	-0.096604568	0.400151	-0.30415288	0.0080872	-0.302461575	0.008868
NDUFA1	-0.135991099	0.1243141	-0.23249014	0.0080955	0.133665149	0.122927
RCCD1	-0.265077948	0.1347203	-0.462350096	0.0080994	-0.432090103	0.014155
UBXN2B	-0.068942354	0.4348229	-0.232260266	0.0080823	-0.058356152	0.506636
SEC14L2	0.35845032	0.2788202	0.862265365	0.0081079	0.444917454	0.176108
KLF2	-0.644748872	0.0680412	-0.933447595	0.008215	0.027702662	0.936087
FAM117B	-0.228469139	0.065287	-0.325550365	0.0082274	-0.419412772	0.000725
SH3GL3	0.188564864	0.572029	0.861958981	0.0082566	0.397560431	0.230711
SHISA2	-0.445112504	0.096098	-0.706555579	0.0082605	-0.851744397	0.001472
LCOR	-0.124541322	0.3143666	-0.326705079	0.0082697	-0.125184509	0.311516
SLC26A9	0.038422402	0.8866977	-0.712695832	0.0082911	-0.043691811	0.871248
EML4	0.011995667	0.8678784	0.189605543	0.0083022	0.156796039	0.029217
GEMIN7	-0.098823199	0.4722158	-0.363598831	0.0083184	-0.215374937	0.116177
CCT6B	0.173946244	0.5487353	0.734028616	0.0083347	0.524110472	0.060609
PAOX	0.26792926	0.2703295	-0.682748778	0.0083711	0.024368576	0.919883
LAMP3	0.312696842	0.4139112	0.973689405	0.0083918	0.760107469	0.04062
CHODL	-0.348376774	0.5277273	1.449656681	0.008426	-0.661580576	0.230618
MOSPD2	0.036108034	0.9074817	0.816605463	0.008456	0.107629884	0.728936
RDH13	-0.18665393	0.2570028	-0.429621121	0.0085401	-0.069429606	0.668697
FOXF2	0.182855108	0.5323662	0.764623707	0.0086144	0.075082837	0.797584
LRRK1	0.357586477	0.4157035	1.150852839	0.0086121	-0.053434606	0.903194
SLC9B2	0.503948659	0.0762752	0.740307198	0.0086038	0.861386694	0.002259
MISP	-0.375880778	0.0528207	-0.509553642	0.0086245	-0.3366461	0.082562
ADAMTS3	0.355546035	0.3359311	0.95785946	0.0087217	0.565334675	0.124118
ARSK	-0.347600746	0.0535324	-0.468826787	0.0087099	-0.627833865	0.000542

ATP6V0C	0.550357793	0.0656835	0.779960064	0.00871	0.85785192	0.003913
CNTROB	0.180286891	0.3040135	0.456908846	0.0087245	0.305188919	0.080691
YTHDF1	0.110039187	0.2338997	-0.243099684	0.0087014	-0.152293433	0.100335
ZFAND6	0.177283452	0.2044606	0.363920832	0.0087191	0.591295641	1.95E-05
SOCS2	0.511848532	0.1330139	0.88528188	0.0087721	0.674079532	0.046981
USMG5	0.035727797	0.7198457	0.258769052	0.0087878	0.253476602	0.010402
COLGALT2	0.234182881	0.5665218	1.045108768	0.0088368	-0.041862445	0.918615
FBXO30	-0.06078576	0.5682803	-0.278715369	0.0088382	-0.401114255	0.000187
ITPKB	0.115731459	0.6008472	0.575557478	0.008834	-0.512507851	0.021323
HSPA8P9	0.568239089	0.2924847	1.400548313	0.0088691	0.606660691	0.260148
MKNK2	0.203017657	0.3173683	-0.53219069	0.0088706	-0.244714071	0.228509
CARD10	-0.310219564	0.0641249	-0.438441793	0.008925	-0.225580345	0.177292
PAPD7	-0.031990991	0.8014445	-0.332189022	0.0089418	-0.134993856	0.287168
TFG	-0.140571351	0.1596595	-0.261037581	0.0089352	-0.136102753	0.172743
ZNF285	-0.483090648	0.0560324	-0.658667026	0.0089366	-0.709619373	0.005134
DCTN6	-0.02792398	0.7329898	0.209585591	0.008959	0.272624434	0.000692
RNF185	0.024228901	0.7969688	-0.246521023	0.0089848	-0.388070055	4.50E-05
CDR2	-0.145217176	0.2678117	-0.343231819	0.0089987	-0.037503337	0.774456
C5orf30	-0.203979345	0.1593661	-0.378005925	0.0090053	-0.034866204	0.808909
ABCA6	0.5410823	0.1270089	0.912747557	0.0090474	0.663911533	0.059456
PDE10A	0.407009674	0.1762459	0.782751598	0.0090757	-0.177625284	0.555355
ERBB2IP	0.161087469	0.2010535	0.328310387	0.0090955	0.425137532	0.000731
DESI1	0.214859612	0.2494011	0.485289627	0.009103	0.52172202	0.005083
C12orf76	0.286855191	0.0658875	0.398988342	0.0091491	0.130281966	0.399764
FGFR2	0.28174921	0.1021231	0.448756441	0.0091471	0.388293094	0.02417
POLR2D	-0.117268388	0.3364215	-0.316964486	0.0091737	-0.18577204	0.12767
REXO4	-0.230871636	0.1380326	-0.402480067	0.009189	-0.334152968	0.030361
LDOC1L	-0.020260009	0.8265748	-0.240616978	0.0092374	-0.17838389	0.053798
MARS2	-0.232407443	0.2427044	-0.5181774	0.0092418	-0.540707992	0.006707
TAX1BP1	0.034543864	0.7257707	0.255379385	0.009257	0.413564769	2.48E-05
BET1L	0.019127994	0.880476	-0.332732515	0.0092852	-0.167521983	0.189499
RFWD3	-0.183421509	0.1767567	-0.352238077	0.0092993	-0.227641724	0.093115
TAPT1	0.170389034	0.2301645	0.366942709	0.0092933	0.395968365	0.004978
RAB3D	-0.128559954	0.4312837	-0.424851056	0.0093139	-0.045152851	0.781794
APOC2	1.075840868	0.2399895	2.348524586	0.0093651	-0.675944822	0.470552
LTBP3	0.04914222	0.8357533	0.612568911	0.0093765	0.116886857	0.621538
MYCBP2	0.245292182	0.0905977	0.376105788	0.0093426	0.211692331	0.143676
REXO1L1P	-0.156083194	0.7577911	1.303438757	0.0093768	-0.315179402	0.533525
SH3YL1	-0.175518276	0.1722103	-0.333844613	0.0093519	-0.141053152	0.272011
GRIK2	0.253018922	0.4723273	0.905157028	0.0094285	-0.062817507	0.858996
SGK2	-0.286940696	0.0984816	-0.450261143	0.0094282	-0.48596613	0.005118
FAM134A	-0.118924467	0.2651614	-0.276106758	0.0094488	-0.228882246	0.031844
INRNPA1P3	0.233774259	0.7054957	1.459781988	0.0095126	0.588512829	0.321799
SP1	-0.057221992	0.5302941	-0.236402283	0.0095202	-0.263130828	0.00392
PTPN6	0.040992524	0.822404	-0.475803721	0.0095726	0.042300324	0.815608
ZNF17	-0.153855531	0.2303139	-0.327268374	0.0095723	-0.376904105	0.003372
FBXO32	0.237747501	0.5622936	1.052742506	0.0095924	-0.075614653	0.854117

TMEM229B	-0.293734994	0.245196	-0.656170171	0.0095998	-0.219231361	0.383941
DIMT1	0.158616913	0.1665957	0.294298634	0.0096533	0.227311264	0.046154
CCDC53	0.224123166	0.0872594	0.332342108	0.0096884	0.298857917	0.020803
C12orf50	0.057548019	0.8540275	0.799004947	0.009714	0.270028428	0.385977
P11-834C11	-0.437872265	0.2286562	-0.939369366	0.0097121	-0.312326594	0.387433
AIMP2	0.083705554	0.5345816	0.339270666	0.0097469	0.362086492	0.005959
GCHFR	-0.118923506	0.4360128	-0.395601058	0.0097833	0.03445497	0.819429
A4GNT	0.848832053	0.0869719	1.276308467	0.0098247	0.698799692	0.158831
CBFA2T2	0.249176862	0.1760621	0.473466746	0.0098397	-0.055192208	0.764675
TFRC	0.204014721	0.244708	0.452530938	0.0098413	0.440242543	0.012033
EMILIN2	-0.301903294	0.2562804	-0.695306307	0.0098581	-0.269219142	0.311191
GRIK1	0.353200166	0.4163049	1.047215845	0.0098496	-0.26103279	0.561418
SOCS6	-0.109893381	0.2916192	-0.267570602	0.0098871	-0.231454201	0.025955
SMU1	0.051967342	0.3429697	0.139168286	0.0099761	0.130905236	0.01582
MYD88	-0.048096227	0.8098933	-0.517387425	0.0100218	-0.429398732	0.032651
1-Sep	-0.11146853	0.7761925	0.927107334	0.0100474	0.27860144	0.457376
ARHGEF7	-0.091802256	0.3814179	-0.269207783	0.0101135	-0.124609673	0.233869
CYB5R1	0.015656085	0.9158049	-0.381481875	0.010148	0.026400941	0.857966
GALNT3	-0.159854625	0.251972	-0.358663772	0.0101544	0.09753189	0.483862
NQO1	-0.209958524	0.2331792	-0.452560739	0.010171	0.138039732	0.432691
PRRG2	-0.233586545	0.2630259	-0.527969708	0.0102079	-0.128658768	0.52919
PDZD8	0.224230811	0.2687322	0.519958513	0.0102215	0.760170753	0.000172
ZBTB39	-0.021553356	0.8532833	-0.299720533	0.0102409	-0.365432272	0.001878
HERPUD2	0.191132228	0.2120787	0.390647545	0.0102869	0.494025436	0.001155
ZNF275	-0.068768847	0.5686748	-0.309483471	0.0103556	-0.326374034	0.007093
CEP250	-0.234678011	0.1327427	-0.399686145	0.0103794	-0.584112826	0.000185
SLC14A2	0.336906425	0.2871586	0.780707405	0.0103784	0.07202503	0.821615
LYRM4	0.153004486	0.2054908	0.305219773	0.010447	0.122804059	0.307868
FAM101B	0.026612738	0.9234892	0.705795892	0.0104687	-0.298618154	0.281877
TMEM136	0.320885931	0.0830258	0.465915454	0.0104818	0.218765125	0.236483
EXD2	-0.166981826	0.1028841	-0.258928907	0.010497	-0.384198243	0.000162
ZNF587B	-0.205946751	0.0909512	-0.310409383	0.0104998	-0.30422234	0.012438
GYG2P1	-0.14593941	0.5731531	0.633002465	0.0105271	-0.052816277	0.836336
PPFIA3	-0.017661289	0.9334482	-0.541632571	0.0105436	-0.270366265	0.202809
CDH11	0.17971303	0.4069152	0.553535959	0.0105977	0.261394493	0.227636
CDK10	-0.140182566	0.2229611	-0.29334024	0.0105965	-0.215299431	0.060905
P11-248J23	0.476141333	0.3790955	1.332019076	0.0105911	0.14695625	0.793792
POLR1D	-0.117865506	0.3535414	-0.324449175	0.0106413	-0.121207446	0.339704
MERTK	-0.167317488	0.2364194	-0.360575457	0.010659	-0.069121827	0.622407
CELSR3	-0.060617148	0.8333891	-0.736287397	0.0106693	-0.606284161	0.03521
NOP58	0.202315985	0.0567327	0.270163461	0.0106885	0.282313954	0.007659
FTO	0.053356101	0.5687048	0.235916288	0.0107051	0.05574865	0.549488
MGAT1	0.038621854	0.6775925	-0.237422897	0.0107111	-0.057016337	0.538903
BCAT2	-0.153993675	0.3363878	-0.408563357	0.0107527	-0.031174789	0.844712
GCA	-0.038430682	0.8315898	-0.462528291	0.0107278	0.040719127	0.821124
SPATA5	0.037875447	0.7709329	0.327290451	0.0107495	0.115158824	0.372728
UBE2M	0.162355491	0.1320129	0.273492616	0.0107395	0.091232513	0.397872

WDR27	-0.303393503	0.0547551	-0.400013087	0.0107789	-0.548573651	0.000524
RPLP2	0.073781836	0.6055581	-0.364377762	0.0107995	-0.104540521	0.464347
ADAMTSL1	0.127904671	0.7484823	1.01252057	0.0108103	-0.005564971	0.988875
ERAP2	0.077919273	0.811867	0.818482821	0.0108507	0.693257234	0.033814
P3-324O17	0.087635365	0.6876761	-0.569154362	0.0108942	-0.44625177	0.045539
ENDOV	-0.068748365	0.5554539	-0.294488398	0.0109296	-0.21724444	0.06057
NUP54	-0.00874702	0.9339127	0.267452243	0.0109237	0.192147951	0.067568
ABCB5	0.311488974	0.295988	0.714802043	0.0110001	0.052293186	0.861156
PGPEP1	-0.404424635	0.053139	-0.531684509	0.0110032	-0.573147563	0.006172
CHRDL1	0.859231045	0.0538157	1.127627086	0.0110433	0.452227521	0.311888
MECOM	-0.371935221	0.0819734	-0.543321775	0.0110563	-0.253923007	0.234754
TSPAN13	0.000857155	0.9939787	-0.288796971	0.0110428	0.197032395	0.081759
CDC27	-0.02569213	0.8613987	0.372741673	0.0111349	0.278082674	0.058438
WASH3P	-0.052074055	0.733465	-0.388773042	0.0111758	-0.378742169	0.013329
CAV1	0.53417757	0.0697943	0.74484748	0.011193	0.41660119	0.15667
MIEN1	-0.12921519	0.3273106	-0.333248926	0.0112385	-0.091645375	0.483032
POLR2K	0.005266447	0.9650712	0.300718371	0.0112634	0.293689036	0.013529
TNFSF15	0.031268487	0.9065992	0.670333427	0.0112922	0.595283479	0.02463
ZNF879	-0.290553084	0.179233	-0.544713954	0.0112991	-1.034960843	2.61E-06
ATE1	-0.003281465	0.9819634	0.36566603	0.0114176	0.113470101	0.432893
EPS8L1	-0.013212803	0.9548378	-0.592432326	0.0113909	0.02653726	0.909312
ZNF45	-0.219493287	0.1821697	-0.415340088	0.0114155	-0.270966983	0.098846
NRIP3	-0.135466882	0.6230159	0.681877603	0.011445	0.137541992	0.614943
NEK9	0.178834075	0.3550509	0.487454791	0.0114575	0.094723113	0.62449
ARHGAP5	-0.143530755	0.1790801	-0.269972398	0.0114653	-0.094389668	0.376653
BCOR	-0.003550177	0.9834112	0.428461971	0.0114807	0.174914757	0.303511
AGBL3	-0.132141532	0.6028755	-0.621138634	0.0114884	-0.594940359	0.023289
FICD	-0.032412525	0.8741547	-0.520037895	0.0115389	-0.427050167	0.038898
GJC2	-0.02416705	0.9319477	-0.73482898	0.011538	-0.713796565	0.014651
RP11-313J2.	0.59566469	0.0633065	0.804806293	0.0115492	0.545049199	0.088746
SDC2	-0.031661863	0.8692407	0.483979612	0.0116185	-0.300493228	0.118643
CCNH	-0.018381718	0.8716306	0.28296998	0.0116667	0.311226687	0.005631
FAM114A2	-0.30536689	0.1943194	-0.592483752	0.0116705	-0.280236393	0.231355
XRN2	0.002675243	0.9683565	0.168537638	0.0117215	0.131117527	0.050489
STK10	0.198702016	0.3436924	0.527661149	0.0117524	0.257715888	0.219403
KIAA2013	-0.125370756	0.3540617	-0.340519825	0.0117903	-0.273999107	0.042857
SRRM4	-0.14239086	0.8319512	1.544487068	0.0118416	0.444268345	0.492161
OTX1	-0.264097867	0.6327389	-1.558960165	0.011869	-0.258017975	0.639986
NME1	-0.025071572	0.8710017	0.386168992	0.0118864	0.303500851	0.048342
TD-3074O7.	0.021369327	0.9936336	-7.186696094	0.0119143	-2.372516314	0.376686
GLIPR1L2	0.078657681	0.7752128	0.669698208	0.011913	0.043873639	0.872685
GAN	-0.14896125	0.1591213	-0.265674525	0.0119289	-0.279391703	0.00818
DDR2	0.460029216	0.0694467	0.636292986	0.0119545	0.167863219	0.507736
RPRD2	-0.038478585	0.7419292	-0.293529562	0.0120219	-0.409262412	0.000494
HDGF	0.232662673	0.0502676	0.298064498	0.01204	0.424095286	0.000353
NNMT	0.453315728	0.4418894	1.470331133	0.012089	1.992346886	0.000663
MYOCD	0.586465844	0.12134	0.946265796	0.0121532	-0.455903309	0.230065

C7orf63	0.490623449	0.0610644	0.652345987	0.0121745	0.607910521	0.02003
SEMA4G	-0.486828369	0.0667347	-0.666393923	0.012188	-0.704821979	0.008035
MLPH	-0.061156266	0.8179178	-0.666970401	0.0122704	0.387653628	0.143279
RP4-765C7.:	-0.051625844	0.8046868	-0.524302553	0.0122767	-0.290013946	0.165264
TGM2	0.289265312	0.2131667	0.580304103	0.0122833	0.013372426	0.954127
DVL1	-0.134052882	0.4071145	-0.404405744	0.0123206	-0.14941046	0.35422
C4BPA	0.440138794	0.4943452	1.562550671	0.012367	-0.605248783	0.364446
REEP4	-0.281341287	0.0501039	-0.354656543	0.0124121	-0.366880214	0.009856
SAG	0.320217673	0.3622122	0.845075725	0.0124558	0.340781257	0.329475
PI4KAP2	-0.11478801	0.3574293	-0.311744847	0.0124929	-0.270807125	0.03016
EZH1	0.047798582	0.8024093	0.471157377	0.0125801	-0.015202078	0.936451
PAQR3	0.151138342	0.3098649	0.362620169	0.0126219	-0.096334413	0.518905
STARD5	0.804352177	0.0505397	1.015288959	0.0126325	0.308389415	0.460247
ACTN1	0.182676628	0.0913328	0.269302743	0.0126757	0.062723961	0.562188
ATXN7L3	-0.093883453	0.3716644	-0.262096304	0.0127074	-0.242696855	0.020951
MRAP2	0.084726213	0.7496423	-0.668959743	0.0127108	0.504243312	0.055435
NARS	-0.080768556	0.5124703	0.306573793	0.0126908	0.351618803	0.004251
AASS	0.261833313	0.2660987	0.582869103	0.0127281	0.099746692	0.671927
CDH19	0.563141902	0.4645708	1.790696576	0.0127612	1.084974224	0.139703
ATP2B1	-0.00349188	0.9794281	0.336420862	0.0128003	0.145106186	0.283399
GRPEL1	0.179270663	0.312952	0.440948265	0.0128128	0.264509638	0.135692
ZNF682	-0.363979469	0.0875188	-0.5219931	0.01287	-0.405542391	0.054841
AKAP8	-0.159304845	0.1252294	-0.256965532	0.0129813	-0.240847785	0.020226
C1orf61	0.506831455	0.1285751	0.817349553	0.0129975	0.422492956	0.210853
SMUG1	-0.059005555	0.5822198	-0.267312529	0.0129986	-0.042434438	0.690956
CD28	0.925278726	0.1119905	1.393724221	0.0130511	1.176929777	0.039048
KIAA0020	0.009343425	0.9270457	0.25111891	0.0130451	0.392346538	0.000105
METTL4	-0.094530919	0.6677045	-0.547079636	0.0130324	-0.081905291	0.707912
RNMTL1	-0.121133022	0.3637377	-0.331192086	0.0130446	-0.348987417	0.009302
EHBP1	0.170533115	0.1777384	0.312406797	0.0131031	0.066244762	0.6004
ENC1	-0.115321647	0.4622384	-0.388974188	0.0130962	-0.068880652	0.660155
CD3G	0.586534034	0.1366607	-0.98586402	0.0131412	0.922996165	0.018983
MFAP3	-0.061819123	0.6306047	-0.318254468	0.0131592	-0.223855813	0.081266
GRHL3	-0.683179652	0.1594546	-1.210283132	0.0131917	-0.449319859	0.351699
USP39	-0.09269931	0.5616674	0.393961146	0.0131845	0.108636802	0.495643
ARPP19	0.048455071	0.6467864	0.261665071	0.0132236	0.233891425	0.026804
LRP8	-0.031400041	0.9090006	0.675178266	0.0132901	0.440079043	0.107148
MCAM	0.413779097	0.0782915	0.575562285	0.0132693	0.376641308	0.107018
FAM160A2	0.069790795	0.6664061	-0.401373171	0.0132981	-0.26113352	0.107066
FGG	0.248602058	0.8522167	3.092308937	0.0133885	-0.100754024	0.939984
CASP9	-0.296712091	0.163092	-0.527120708	0.0134196	-0.370227829	0.082147
CTSD	0.23133499	0.1196466	0.36701032	0.0134414	0.266130601	0.0732
MCOLN2	-0.342478146	0.3968075	0.94880664	0.0134543	0.212664918	0.596925
RGMB	-0.320280017	0.0818024	-0.453818941	0.0134577	-0.358773522	0.051003
P11-15H20.	-0.37051133	0.0619645	-0.488248155	0.0134566	-0.536360316	0.007066
PHF12	-0.107302499	0.5028575	-0.393694168	0.013687	-0.529852168	0.000954
TARS	0.052501655	0.6872482	0.321062089	0.0136981	0.492213277	0.000157

ANKRD44	0.443676592	0.1643063	0.783329692	0.0137425	0.86316923	0.006775
DAPK2	0.616110447	0.0883726	0.878469563	0.0137512	0.525247519	0.14576
UGGT1	-0.189315818	0.0573492	-0.24511544	0.0137214	0.014926351	0.880673
PRMT3	0.020321587	0.88123	0.327276041	0.0137706	0.368647636	0.005555
ZNF852	-0.433241715	0.1189918	-0.675198315	0.0138147	-0.704511947	0.012398
AC002543.2	0.633733285	0.1362647	1.000625369	0.0138408	0.664721582	0.112974
ANO1	0.494224943	0.1005749	0.739275818	0.0138703	0.740632007	0.013748
FBXL6	0.070564324	0.7064516	-0.466212511	0.0138933	-0.239080596	0.20868
GALK2	-0.26835155	0.0648842	-0.35627719	0.0139338	-0.392256461	0.006897
TEX15	0.030029375	0.9435465	1.020425704	0.0139842	0.128401715	0.762183
ZFX	-0.114015679	0.1162436	-0.177766122	0.0140157	-0.254514503	0.000449
FOXA1	-0.083388474	0.7120879	-0.555358137	0.0140293	-0.188451875	0.404006
INTS5	-0.053226734	0.6940845	-0.33295487	0.0140323	-0.165813553	0.219963
DNAH7	-0.576895512	0.1006336	-0.849179151	0.0140924	-0.453033946	0.191702
ENPP1	0.308603145	0.1674306	0.544799481	0.0141357	0.634936109	0.004252
KIT	0.189366438	0.4856629	0.662280278	0.0141283	-0.022355212	0.934446
LPXN	0.321575143	0.1742847	0.557458152	0.0141251	0.261748681	0.266607
RPUSD1	-0.119553376	0.4894192	-0.424581678	0.0141292	-0.353615783	0.041009
STARD8	0.313824038	0.1902611	0.58290349	0.014099	0.300519281	0.208194
ZNF431	-0.100098478	0.3081	-0.239815419	0.0141344	-0.227289987	0.020284
RBM15	-0.200289947	0.1031785	-0.300355267	0.0141496	-0.25068978	0.040852
MT1H	0.880143385	0.5514489	3.580177743	0.0142159	0.31968085	0.829766
CANT1	-0.165733067	0.3311218	-0.417690802	0.0142513	-0.168484848	0.321831
MAX	0.153584518	0.1894824	0.284947031	0.0142533	0.271848447	0.019694
ZNF202	-0.254042906	0.1238887	-0.404268416	0.0142333	-0.256921307	0.118772
ZNF558	-0.087311593	0.4660293	-0.293246454	0.0142434	-0.360111887	0.002734
AGMO	0.220335136	0.7978444	2.035009797	0.0143646	1.338465516	0.108379
BLMH	0.036619337	0.8130563	0.37457323	0.0144805	0.185700398	0.228083
ITM2B	0.188486456	0.125686	0.300597969	0.0145017	0.346045493	0.004895
PDE5A	0.064813767	0.7631447	0.525542033	0.0144857	-0.249475853	0.246159
ERMP1	-0.120197381	0.2773051	-0.270154449	0.0145243	-0.112321185	0.309012
SKAP2	-0.142201033	0.5169292	-0.53716492	0.0145476	-0.371557517	0.090245
ZNF543	0.045735733	0.7467258	-0.349647842	0.0145559	-0.33148786	0.021263
HOXC8	-0.050986481	0.9003961	-1.023460423	0.0145791	-0.243493949	0.550642
ODC1	0.017422802	0.9138451	0.391709844	0.0146274	0.301609384	0.060382
RABIF	-0.011675136	0.9187665	-0.279723042	0.014747	-0.000550361	0.996128
POTEC	-0.109057839	0.7665938	0.853887188	0.0147975	0.077637167	0.829892
YAF2	0.04837954	0.7775725	0.411905431	0.0148203	0.23103259	0.174454
TBC1D12	0.16012441	0.3266057	0.395478088	0.0148682	0.1252567	0.44049
ALDH1L1	0.091927865	0.8169186	0.948784798	0.0150281	-0.348313212	0.390055
OPRM1	0.766026858	0.0906105	1.067844493	0.0150117	0.439190462	0.343856
TA-246H3.1	-0.588329951	0.1768852	-1.09428391	0.0150573	-0.330725097	0.429116
ENTPD5	-0.2214415	0.1437568	-0.367914701	0.0151446	-0.299460887	0.048079
INRNPA1P2	0.102983199	0.7701148	0.793097296	0.0152215	-0.609200929	0.106237
TLE1	0.25017593	0.1055168	0.373909298	0.0152297	0.326823672	0.034312
DPT	0.275004151	0.7193526	1.772761986	0.0152554	0.802141716	0.283012
TUBA1B	0.121580247	0.407729	0.356131674	0.0152847	0.353037164	0.016199

SSH3	0.083084377	0.6557996	-0.453187684	0.0153134	-0.070870233	0.703244
ZNF736	0.165290833	0.8069877	1.529574783	0.0153359	0.722258348	0.25673
CCDC141	-0.916139099	0.0570789	-1.162626473	0.0153514	-0.693316632	0.148496
MRPL17	-0.225670453	0.1457193	-0.375253507	0.0153639	-0.167685352	0.278204
C2CD5	-0.264564035	0.0895202	-0.376456911	0.0154608	-0.001267113	0.993488
RPL13A	-0.015838157	0.8975375	-0.297794128	0.0154773	-0.022632479	0.853977
CAPN13	0.377061714	0.1369023	-0.626586914	0.015509	0.739262049	0.003369
HMBOX1	-0.107576451	0.4717599	-0.361471593	0.0154964	-0.244686731	0.101529
RPA3	-0.296159699	0.0813648	-0.404866875	0.0156585	-0.110242214	0.510238
CXCR4	0.351878066	0.3375885	0.88298887	0.015698	1.447602296	7.29E-05
NANOGP1	0.904041129	0.0525619	1.099642669	0.0156995	0.945344532	0.040443
CLASP2	-0.036662713	0.7703948	0.301456634	0.0157097	0.248062457	0.047004
RSU1	0.145373462	0.2225253	0.285640808	0.0157752	0.414273157	0.00046
FBXO44	-0.197873215	0.2850744	-0.443350981	0.0158084	-0.623206969	0.00081
ATAT1	0.133491663	0.3567445	-0.355186356	0.0158417	-0.30009156	0.045699
SERPINA1	0.075609636	0.8128296	0.770398816	0.015829	0.047287995	0.882273
NDUFA8	-0.064990356	0.5507158	-0.261963647	0.0158697	-0.200857587	0.065106
DEGS1	0.11929661	0.4515171	0.379763591	0.0159382	0.039418058	0.803418
PRELID1	0.071635467	0.6928553	0.436311921	0.0159107	0.273982071	0.130324
TEF	-0.161356752	0.5186409	-0.603165974	0.0159231	-0.458585745	0.06664
TMPPE	0.019046189	0.9140219	-0.431876132	0.0159194	-0.140096791	0.427762
PVRL4	-0.186295654	0.5472325	-0.748056332	0.015998	0.427481027	0.163685
COL8A1	0.730468533	0.0893261	1.029705702	0.0161271	1.130115138	0.008156
C9orf142	-0.245355803	0.2062266	-0.465413193	0.0161771	-0.250123064	0.194725
LAMTOR3	0.038093682	0.6940156	0.231049067	0.016197	0.33331579	0.000528
SEPT7P6	0.692345648	0.3132027	1.520019313	0.0162023	1.731241351	0.005858
C1orf51	0.276594675	0.1937618	0.49435231	0.0162174	0.444587049	0.033467
SHC4	-0.302056379	0.2648541	0.629320873	0.0162469	0.358385175	0.175548
CLDN23	-0.159291666	0.5220397	-0.601876116	0.0162903	0.287104656	0.241177
FAM126B	0.196346299	0.1447522	0.322441126	0.0162948	0.384364874	0.004207
RPE	-0.13318436	0.189174	-0.242007559	0.0162809	0.0077858	0.938249
PSPH	-0.233172483	0.0991245	-0.33999786	0.0163056	-0.294432217	0.037693
AKAP2	0.351347868	0.1171984	0.537915223	0.0163307	0.200717588	0.370812
MUC13	1.165852458	0.2582688	2.469465839	0.0163498	0.958268854	0.35287
ROCK1P1	0.718486873	0.2508074	1.479792409	0.0163709	0.194287574	0.754335
MC1R	0.134239255	0.4697671	-0.456031659	0.0163923	-0.469530505	0.014209
NUDT12	-0.234369473	0.0542186	-0.289937075	0.0164814	-0.092791077	0.442569
P11-266K4.	0.212629189	0.7427951	1.44360642	0.0165019	0.438553737	0.489226
GARS	0.260456773	0.0730289	0.347823403	0.0165377	0.624793661	1.65E-05
NTRK2	0.601876782	0.2891666	1.351209423	0.0165624	0.230445957	0.685833
ASRGL1	0.456243908	0.0520768	0.55968436	0.0166107	0.082859379	0.725777
FAM73B	0.017983428	0.9328493	-0.510352277	0.0166076	-0.521088111	0.015041
CHCHD3	-0.10718417	0.2953521	0.242289211	0.016645	0.209981255	0.038415
DPH7	-0.196050483	0.2110275	-0.369506093	0.016763	-0.410266127	0.008472
SLC1A2	0.64589416	0.2133172	1.202320578	0.0167736	1.454874311	0.004301
EPHB6	0.997932412	0.244109	1.979931707	0.0167948	1.750871502	0.036691
TOX3	0.148271394	0.4546351	-0.475154311	0.0168021	-0.072523575	0.714726

NUP98	0.206404409	0.0537383	0.255470335	0.0168695	0.299023481	0.005156
MSRA	0.029552578	0.872546	0.42113991	0.0169119	0.393529066	0.027646
SLC20A2	0.017959422	0.8716295	0.262745865	0.0169053	0.125525587	0.25571
ZNF444	-0.139420034	0.4272586	-0.417124982	0.0169079	-0.28753783	0.103165
ZNF582	-0.175821514	0.4447438	-0.557990345	0.0169477	-0.710366082	0.002237
C12orf55	-0.145008256	0.5481586	0.57415699	0.0170001	0.056424556	0.814939
NUP107	-0.033772484	0.7691782	0.272624212	0.0170257	0.181230053	0.113233
FAM177B	-0.068708673	0.8473834	-0.857664372	0.017091	0.255237979	0.472321
GLT8D1	-0.206722186	0.0914268	-0.291280079	0.0171154	-0.183314665	0.13387
GTPBP8	-0.222751748	0.093936	-0.314821089	0.0171423	-0.041146832	0.75373
DDX25	0.072352869	0.8454172	0.864635876	0.0172339	0.1044907	0.783937
MTMR8	-0.173289277	0.4844364	-0.593637866	0.0173074	-0.031393138	0.897638
PNPLA1	-0.194177875	0.7320311	1.176821534	0.0173245	0.071551868	0.894962
EDN3	0.101455735	0.7942488	0.923287034	0.0173487	-0.411111706	0.291496
FOXP1	-0.095207498	0.4638287	0.307596066	0.017418	0.277695608	0.03191
SH3D21	-0.308369632	0.1683815	-0.53220639	0.0174379	-0.415242498	0.064443
KIAA0232	-0.099231402	0.3327589	-0.243342296	0.0174713	-0.237523855	0.020443
UBASH3B	0.209095744	0.6022977	0.9480363	0.0174631	0.464457368	0.245326
EPB41L4A	-0.026403281	0.9099152	0.543423335	0.0174918	0.185594877	0.420222
ZNF507	-0.242738383	0.1158827	-0.365365577	0.0175026	-0.327737856	0.033519
MYLK	0.530001557	0.10585	0.778007516	0.0175431	-0.080887491	0.805131
PINX1	-0.210392951	0.1887679	0.368599332	0.0175455	0.163474551	0.295768
ACTA2	0.329436378	0.2039147	0.615578406	0.0175642	0.11766708	0.65
PITPNA	0.09589661	0.3797838	0.258159013	0.0175864	0.079402659	0.466463
GPR75-ASB	-0.051714961	0.7085525	0.325893427	0.0176253	-0.030194171	0.826733
SGTB	0.081174183	0.6150815	0.375690842	0.0176214	-0.252580938	0.117171
ZBTB48	-0.126699195	0.5329818	-0.484414834	0.0176128	-0.300997138	0.140505
TMEM216	-0.042834262	0.7826316	-0.369228455	0.0176846	-0.265435395	0.090404
GMPPB	-0.012593809	0.9325469	-0.353567841	0.0177014	-0.064732337	0.663482
MON1B	0.022339309	0.8134994	-0.223805927	0.0177308	0.025464692	0.786828
DPM3	-0.210833874	0.2680203	-0.450965401	0.0177681	-0.065735818	0.726234
RASAL2	-0.151349898	0.2742639	0.324805159	0.0178078	0.277978541	0.043213
SH3BGRL2	-0.113159252	0.4365497	-0.344589641	0.0178203	0.279630182	0.054098
TWIST1	0.238224699	0.6133857	1.094680371	0.0178508	0.170455569	0.716428
FAM109A	-0.197963014	0.1983747	-0.361615453	0.0179004	-0.081473105	0.591109
NIPAL3	0.055468069	0.6262755	-0.270760525	0.0179258	-0.217884466	0.057889
NKX6-2	-0.4665186	0.3703612	-1.236039069	0.0179282	-0.197540184	0.704033
SHE	0.578228654	0.1772709	0.974807392	0.0179617	0.340027843	0.42304
OCLN	-0.136544299	0.5192497	-0.50105117	0.0180782	-0.112987833	0.593539
PDE3B	-0.31991801	0.1898245	0.573559379	0.0181302	-0.420781353	0.084514
UGT1A6	1.149297613	0.1097729	1.652720066	0.0181368	1.141293053	0.103646
EGFL6	2.453253905	0.0668341	3.144862701	0.0181977	3.622678585	0.006449
PDPK1	-0.124969035	0.3579496	-0.320786513	0.0182899	-0.217811703	0.109227
LIPT1	-0.034930008	0.8687834	-0.508920153	0.0183598	-0.04079229	0.847273
TMEM87B	-0.173963058	0.1421389	-0.278857594	0.0183569	0.031878799	0.787094
CYB561	-0.129127474	0.4865597	-0.437987098	0.0184294	0.158675456	0.389255
TRAK2	-0.141209655	0.1507705	-0.230250672	0.0184566	-0.143409762	0.142466

PUSL1	-0.135094829	0.4732321	-0.445580275	0.0184969	-0.092544991	0.619703
NUTF2	0.065635772	0.5312462	0.245752311	0.0185277	0.26927271	0.009922
TCP1	0.114813979	0.2080026	0.214235374	0.0185398	0.244561449	0.007195
SLC29A2	-0.103680454	0.5318603	-0.390690768	0.0185666	-0.102018694	0.537607
HMHA1	-0.219808801	0.2498003	-0.450000771	0.0186022	-0.675295405	0.000451
VEZT	0.093412237	0.3319105	0.225734345	0.0186011	0.345842761	0.000308
UPF1	-0.239970814	0.1226379	-0.365419023	0.0186418	-0.454037924	0.003503
NDUFB1	-0.058016192	0.6134314	0.265529359	0.018653	0.282941079	0.012455
CDH7	0.147954184	0.745922	1.067105086	0.0187252	-0.271291344	0.554974
TACR2	1.267970848	0.179175	2.128695984	0.0187264	2.39474936	0.007923
SCRN2	-0.234538185	0.1600628	-0.390357122	0.0188009	-0.202779459	0.22256
SPECC1	0.230299471	0.1420311	0.368028358	0.0188298	0.330583435	0.034881
TAF5L	0.074516592	0.3992211	-0.208031865	0.018839	-0.066219798	0.454196
C17orf59	-0.390044164	0.1226258	-0.592016423	0.0188613	-0.907828627	0.00046
ALX3	-0.478136801	0.0630966	-0.595278712	0.0189165	-0.461073983	0.067911
CNPY3	0.011929772	0.9024652	-0.228369575	0.0189956	-0.044221391	0.648224
CYTL1	0.091762155	0.7921148	0.810086732	0.0189707	0.121826615	0.725895
FOXF1	0.222296167	0.3817213	0.594940274	0.01896	-0.039026558	0.878016
ZNF175	-0.395873594	0.2262051	-0.75804946	0.0189954	-0.653368223	0.04504
WDR52	-0.368361476	0.0618571	-0.462107779	0.0190792	-0.179207993	0.362456
CDC42EP4	0.010245086	0.9246996	-0.254109737	0.0191572	-0.167566867	0.122468
PTPN23	-0.29348282	0.235597	-0.580685769	0.0191408	-0.18167207	0.463504
TUBGCP4	-0.082492013	0.5639259	-0.334144176	0.0191569	-0.337606002	0.018684
BRWD3	-0.118152652	0.3759038	-0.3123837	0.0192091	-0.289418203	0.030177
WSCD1	0.283613003	0.224529	0.536752048	0.0192271	-0.138888819	0.561199
NANP	-0.159354657	0.2664081	-0.334886387	0.0192526	-0.145345	0.308251
TFCP2L1	0.063082677	0.8488262	-0.776071698	0.0192915	0.2584852	0.43332
KANK2	0.162461513	0.4542123	0.507245398	0.0193077	-0.110349823	0.6115
TXNDC12	0.012291778	0.9192228	-0.283034202	0.0193145	-0.132784519	0.27219
RLIMP1	0.895599104	0.1104677	1.263576156	0.019336	0.997381031	0.070716
ZNF589	-0.319429417	0.0702804	-0.407954974	0.0194244	-0.376391129	0.031588
KCNS3	-0.051169981	0.7806018	-0.431032067	0.0194595	0.267195971	0.141243
TMEM125	-0.268903321	0.3385458	-0.657191626	0.0194758	-0.256368626	0.361068
PBOV1	0.494610048	0.2183678	0.903379635	0.0195067	1.171243077	0.002271
C7orf31	-0.23961787	0.2351811	-0.46781819	0.0195295	-0.237852029	0.230569
CDKN1C	-0.283720908	0.134814	-0.442552155	0.0195445	-0.746750718	9.25E-05
P11-169L17	0.108891579	0.7760418	0.838955981	0.0195424	0.249494504	0.505272
ZNF765	-0.216711567	0.1851802	-0.381705095	0.0195587	-0.308065582	0.059857
C8orf47	0.044860668	0.8950643	-0.814495922	0.0196451	0.399038626	0.229755
SNRPGP18	-0.28996816	0.6963567	1.463219543	0.0196383	0.794073748	0.226416
CBWD3	0.355308483	0.0584471	0.433318357	0.0197103	0.325882142	0.081867
GABARAPL	-0.064441944	0.5431477	-0.246053087	0.0197407	-0.10298835	0.329133
STX12	0.219610084	0.0505986	0.261040295	0.0197623	0.325784143	0.00359
CNR2	-0.274545417	0.4016162	0.68566311	0.019802	0.015303446	0.960974
AP002387.1	0.002843049	0.9955308	1.099923553	0.0198709	0.669341103	0.16154
LPGAT1	0.174943307	0.1135223	0.256653125	0.0199007	0.163946122	0.137536
PAQR7	-0.294276722	0.0507507	-0.347557322	0.0199083	-0.411811171	0.006199

KCNQ5	0.763364032	0.4910669	2.485024534	0.0199538	-0.561879711	0.624247
OST4	0.250496589	0.0517213	0.299019311	0.0199574	0.344856683	0.007292
ZNF772	-0.288365421	0.0924615	-0.397777128	0.0200094	-0.519435991	0.002495
ALDH18A1	-0.14136242	0.317174	-0.328580694	0.0200625	-0.180389615	0.20171
ANKMY2	-0.059185049	0.5743772	-0.244652568	0.0200628	-0.066238663	0.528182
P11-84C13.	-0.289011744	0.0670712	-0.365371162	0.020055	-0.332838559	0.034458
SH3BP5L	-0.118548282	0.2115871	-0.219882582	0.0200294	-0.257368677	0.006674
NCLN	-0.158518089	0.1958281	-0.284599108	0.0201105	-0.145587034	0.234294
PROSC	-0.140107429	0.2144726	-0.261050185	0.0201201	-0.137216105	0.222544
CLIP1	0.117384006	0.2159801	0.218678581	0.02016	0.32109036	0.000636
UBE2G2	0.131940994	0.1494219	0.210488491	0.0202051	0.207023042	0.022541
CCDC160	1.053540467	0.0623751	1.271633364	0.0202435	0.723321933	0.207989
AP1S2	-0.1149304	0.5957466	0.499488223	0.0203199	-0.130389303	0.546893
FABP2	0.318986783	0.6505214	1.628520067	0.020358	-1.171051647	0.098554
RPL41	-0.018626156	0.8441753	-0.219769289	0.0203765	-0.013017428	0.890719
PRSS27	0.122016987	0.713488	0.730406282	0.0204592	0.13329593	0.682913
RGL2	0.051214942	0.6176326	-0.238223253	0.0205501	-0.198378727	0.053453
SMOC1	-0.445968017	0.5762496	1.600835938	0.0205373	-0.027882683	0.9696
ZDHHC7	-0.162361939	0.2366067	-0.317414687	0.0205435	-0.143550875	0.294611
C4orf3	-0.107313126	0.2741017	-0.226816624	0.020583	-0.124561112	0.203525
C4orf32	0.000314468	0.9987104	0.449366405	0.0206709	0.237898573	0.220898
MOB3A	0.183637915	0.2325879	-0.359587468	0.0206675	-0.433944916	0.005511
UBTD2	0.145772933	0.0978291	0.202213871	0.0206859	0.217004658	0.013208
ZDHHC8P1	-0.247550678	0.5059803	-0.860849389	0.0207595	-1.607516443	6.35E-05
DDX46	0.193958035	0.108883	0.279030662	0.0208088	0.263402592	0.029124
CACNB2	0.392153018	0.2316732	0.750805015	0.0208385	-0.360800015	0.277176
IRAK1BP1	-0.210376284	0.3484537	0.505098076	0.0208674	-0.03598326	0.87152
DENND4B	-0.163823508	0.3211923	-0.381222421	0.0209774	-0.315495485	0.056358
KCTD6	-0.232515602	0.1524864	-0.370445787	0.0209821	-0.44314451	0.006693
MKRN2	-0.087521364	0.3680374	-0.223144825	0.0210188	-0.118837726	0.219086
NONO	0.112287237	0.5247122	0.407273149	0.0210148	0.234861403	0.183316
PKP2	-0.279824681	0.0652984	-0.349550706	0.021037	-0.285437743	0.059403
PURB	0.12269042	0.1926047	0.216022266	0.0210468	0.140559382	0.13439
RCAN3	-0.195077438	0.4013028	-0.536659295	0.0210407	-0.032086634	0.890022
TMEM141	-0.155731581	0.3302786	-0.369068197	0.0210023	-0.076968266	0.629812
SIGLEC10	0.119625322	0.6740267	0.624392641	0.0212745	0.379566396	0.170666
CC1L-ADOF	0.38117477	0.2376256	0.733778794	0.0212753	0.557584183	0.08174
MYZAP	-0.268111537	0.1501076	-0.427888962	0.021324	-0.319508519	0.08542
DCBLD2	-0.083323094	0.5707748	0.337582338	0.0213435	0.031281793	0.831292
N4BP2L1	-0.341951433	0.2950049	0.704070818	0.0213623	-0.299716508	0.348503
FPR1	-0.257386614	0.6447814	1.152104681	0.0214339	0.030263185	0.954186
ARHGDIA	0.217643793	0.2341544	-0.421756646	0.0214657	-0.369941364	0.04366
TBRG4	0.019817491	0.8561038	-0.250835208	0.0215218	-0.058924737	0.589078
FAM13A	0.07202439	0.7713732	0.568727766	0.0215777	1.140371483	3.97E-06
ACSL6	0.176863065	0.7035416	1.022710696	0.0215991	-0.311476124	0.515518
GTF2E2	-0.024522704	0.8526376	0.301089696	0.0215952	0.213385071	0.104101
CCDC120	-0.068936973	0.7543139	-0.511705722	0.0216542	-0.294715397	0.184057

FAM117A	-0.172882687	0.5023894	-0.595578784	0.0216477	-0.354228098	0.17057
BLVRA	0.183189427	0.4982537	0.6109106	0.0216735	0.172351797	0.522627
ILF2	0.020124752	0.8131561	0.194629369	0.021729	0.155063539	0.067744
SLC44A1	-0.037665492	0.7864787	-0.319051759	0.0217356	-0.021753332	0.875583
TSEN15	-0.123740232	0.2830561	0.258113475	0.0217443	0.31890806	0.004628
RILPL1	0.03872887	0.8294438	0.411075852	0.0217618	-0.06332852	0.726486
CCDC181	-0.066384489	0.8051704	0.574364336	0.0217743	-0.259796304	0.340706
PSD3	0.279360694	0.0719609	0.355704972	0.0218032	0.416183779	0.007211
FAM175B	-0.135606463	0.1412047	-0.209506639	0.0218202	-0.194715165	0.03382
FGD4	-0.154403642	0.3623817	-0.388255928	0.0219038	0.152033696	0.368603
FAM102B	-0.066617679	0.5791209	-0.274825402	0.0219362	-0.03499429	0.77006
GABPB1	0.184178427	0.1207719	0.270716287	0.0219215	0.401007065	0.000675
KIAA1919	-0.206358027	0.0619509	-0.251116722	0.0219371	-0.456982087	3.93E-05
P11-390M11	0.464476008	0.2648493	0.898099731	0.0219825	-0.005875379	0.989174
FMN2	0.417860713	0.429232	1.169338658	0.0220058	-0.068992212	0.899576
NLRX1	0.09660685	0.5850318	-0.409087656	0.0221148	0.004771235	0.978489
SFPQ	-0.162726419	0.1534651	0.260311125	0.0221971	0.155332017	0.172526
SLC2A10	-0.142285904	0.3447327	-0.344254483	0.0222761	0.044059775	0.769047
EFR3B	0.117968616	0.6880575	0.6649103	0.0224033	-0.040437459	0.890671
USP6	0.008282861	0.9685699	0.466802581	0.0223943	0.311429315	0.133025
EIF4A1	0.100506472	0.3343058	0.237439627	0.0224878	0.295162451	0.004556
PRDM11	-0.146177116	0.7429165	-1.023912324	0.0225425	-0.159227875	0.720047
STIM2	0.168752476	0.1605052	0.271588408	0.0225537	0.026544307	0.824826
ZNF689	-0.373185062	0.0537602	-0.439898635	0.022555	-0.568627912	0.003315
ZNF66	-0.568943212	0.0685101	-0.710180839	0.0226425	-0.485599499	0.119494
C1orf198	0.11573845	0.3034995	-0.256941394	0.0226625	-0.09064416	0.420718
CEP164	-0.185446762	0.1664102	-0.302957508	0.0227446	-0.333038868	0.013053
EEF1A1P10	0.385377546	0.2827741	0.779900726	0.0227112	0.729870281	0.034714
OSBPL3	-0.055406478	0.6715157	-0.297111619	0.0227348	-0.038415181	0.767908
RINT1	-0.016401509	0.8749814	-0.23610746	0.022744	-0.234488153	0.024078
ZIM3	1.03775709	0.0787474	1.304982892	0.0227315	1.058834533	0.069907
EPS8L2	-0.230989053	0.1963854	-0.407199517	0.0228071	-0.56156579	0.00169
CTC-534A2.:	-0.298130812	0.1044054	-0.414966003	0.0228442	-0.099637372	0.579721
EEF1A1P22	-0.19091398	0.4910657	-0.646949748	0.0228928	0.166974766	0.527563
OR11A1	-0.050799153	0.9355277	1.295796505	0.0228902	0.145826681	0.810714
CTDSPL2	0.110863224	0.1439351	0.171355208	0.0230094	0.176409537	0.019396
FAR2P4	0.06634625	0.892924	1.023405474	0.0230231	0.765835767	0.100453
VSNL1	0.862832057	0.20989	1.540447137	0.0230237	2.06474956	0.002189
FAHD2CP	0.017471209	0.9494336	-0.635621619	0.0230576	0.428252739	0.112793
PDXDC2P	-0.271188712	0.0754605	-0.346149458	0.0230813	-0.249493736	0.100365
SUDS3	0.018945493	0.8134332	-0.181715739	0.023084	-0.024447248	0.759695
BTN3A2	-0.174621848	0.2537156	-0.346459614	0.0231276	-0.192060327	0.208191
CAP2	0.419664884	0.1461952	0.641813239	0.0231385	0.071917211	0.805452
ERI1	-0.185645812	0.1091932	-0.26079348	0.0231623	-0.122987085	0.285002
HCG27	0.093293633	0.8821806	1.270344358	0.0231774	0.234118907	0.69847
MIPOL1	-0.058391266	0.7680541	0.448992868	0.0231662	0.08768897	0.657653
PDCD4	0.07760837	0.6838875	-0.432984904	0.023153	-0.074400771	0.696257

AADAT	0.148287412	0.3524241	0.357421715	0.0232583	0.250967113	0.114087
PMM1	-0.233725639	0.226143	-0.43754067	0.0232462	-0.515138462	0.008017
ACTL6A	0.027897009	0.8601434	0.35758188	0.0232821	0.268139833	0.089211
ACBD4	-0.145889877	0.4884907	-0.481102084	0.0233204	-0.485835858	0.02392
GSG1	0.063506064	0.7894332	0.528847784	0.0233159	-0.07086722	0.765845
RPL8	-0.04031011	0.7591337	-0.298074484	0.0233692	-0.105891396	0.420489
TSPAN31	0.065255263	0.6055826	-0.286967874	0.0234354	-0.071000703	0.574466
BEND5	0.575230391	0.0631644	0.690088946	0.0234602	-0.018881091	0.951752
ZNF502	-0.304040533	0.1194395	-0.429290101	0.0235215	-0.366233882	0.058963
LAP3	0.139947585	0.3217064	0.318302853	0.0235526	0.267468438	0.057075
ACAN	-0.041621984	0.9598856	1.7557571	0.0236154	-0.526259018	0.535801
PIP5K1A	0.218475033	0.0597477	0.262157035	0.0235866	0.053046786	0.647386
TIGD4	-0.127235287	0.6938202	-0.760312979	0.023595	-0.508666396	0.123926
C2orf91	-0.253520398	0.2855464	0.506557075	0.0236551	0.100699878	0.660564
P11-74M13	0.517575334	0.2220883	0.930767308	0.0237648	-0.448210069	0.317934
VPS37C	0.044037112	0.6979902	-0.257070875	0.0237943	-0.112979104	0.318243
FAM105B	-0.00596973	0.9725043	-0.39059714	0.023815	-0.17778927	0.303843
SH3BP1	0.086052046	0.5890243	-0.36180486	0.0238329	-0.159913727	0.316373
GPR146	0.049043671	0.9067036	-0.98511286	0.0238525	-0.791404198	0.079206
LPPR4	1.22229974	0.0602063	1.457860125	0.0238764	1.57012441	0.014951
PPM1A	-0.036520389	0.6283747	0.168328417	0.0238884	0.088015218	0.239869
KIAA0247	0.149669546	0.4137666	-0.415547385	0.0239673	0.0173803	0.924388
NSUN5P1	-0.26896259	0.2112577	-0.484927864	0.0239822	-0.929359109	1.66E-05
ZNF526	-0.151416302	0.2550995	-0.298392964	0.0240061	-0.378360364	0.004423
NDUFB11	-0.010026025	0.9326077	-0.26756431	0.0240322	-0.063993249	0.587893
HBD	0.093642922	0.8837357	-1.523439458	0.0240721	-1.337037299	0.050631
BIN3	-0.02426675	0.7852354	-0.200860118	0.024084	-0.009491325	0.914275
DDX28	-0.113516069	0.444674	-0.334588393	0.0241254	-0.287687907	0.053252
ACSL3	0.106792331	0.3915235	0.280611088	0.0241866	0.487902429	8.82E-05
THSD7B	0.285068822	0.6608803	-1.535514152	0.0242079	-1.573639479	0.019463
CASP14	0.687993308	0.2264168	1.232958393	0.02424	0.012174087	0.983242
TRIM22	-0.062623384	0.8093615	0.583086885	0.0242361	0.049763847	0.847786
GPER1	-0.163101022	0.6491437	-0.825759708	0.0244073	0.257202407	0.461163
RARB	-0.195654705	0.2091497	0.342348151	0.0244457	0.154298534	0.31429
TIMM44	-0.086844334	0.3903035	-0.227106033	0.0245308	-0.081559588	0.417139
P11-693N9	0.6147701	0.472306	1.787702659	0.0246512	1.734844301	0.031403
C14orf2	-0.149125367	0.1449465	-0.227803372	0.0247838	-0.111284592	0.273622
COG8	-0.178104634	0.0764967	-0.223783033	0.0248078	-0.413543902	4.23E-05
FES	-0.04618468	0.8800865	0.670052103	0.0248605	0.409431308	0.176916
XXYLT1	0.166693685	0.2176953	0.298793565	0.0248711	-0.022141302	0.870079
PAIP1	0.055846808	0.563678	0.216098107	0.0249651	0.341987419	0.000384
LACTB	0.228182705	0.2722339	0.463801137	0.0250767	0.738667608	0.00035
KIF16B	-0.33449327	0.0976163	-0.451058099	0.0251887	-0.27987045	0.164838
PTPRG	-0.026016995	0.8938758	0.434515617	0.0252389	0.00527848	0.978385
TRIM38	-0.122639885	0.2966465	-0.262124963	0.0252952	-0.374044576	0.00151
ZCCHC10	0.222691472	0.0956329	0.29673233	0.025333	0.413471315	0.001823
GBP1	0.529649678	0.0880052	0.693260307	0.0253887	0.357093261	0.250198

C1orf74	-0.312419209	0.0812228	-0.395936236	0.0254216	-0.066240864	0.704151
CHD3	-0.149138648	0.4552035	0.446127563	0.0254104	0.279562279	0.161539
NBPF1	-0.300291057	0.0516581	-0.343222514	0.0254545	-0.555529249	0.000314
SSTR2	0.008954235	0.9657364	0.455259637	0.0254931	-0.017891929	0.931205
S1PR1	0.085502026	0.82251	-0.861823287	0.0255368	-0.371531485	0.332202
TXLNA	0.008161519	0.9320526	-0.213714315	0.0255379	-0.119721372	0.210935
ZNF324B	0.152352888	0.3647695	-0.376778615	0.025547	0.072503691	0.664363
ST3GAL3	0.309246038	0.1642082	0.488286648	0.0255791	0.158027762	0.477691
KIAA1328	-0.076412304	0.7578291	0.552299168	0.0256403	0.049442831	0.841787
PGBD5	-0.366844029	0.2660397	-0.737832588	0.0256435	-0.338952954	0.306222
SPHK1	0.834131095	0.0610154	0.986796089	0.0256041	0.723857949	0.103213
TM4SF20	-0.008903039	0.990285	1.621811438	0.0256384	-2.204237349	0.003299
SLX4IP	-0.250077802	0.050182	-0.274046978	0.0256737	-0.437700338	0.000554
ECSIT	-0.057132125	0.7380767	-0.380994407	0.0257188	-0.180244852	0.290666
HMGCL	-0.211265398	0.0734669	-0.262123008	0.0257908	-0.143261656	0.222335
UBA3	-0.086618829	0.3456348	0.202487096	0.0258091	0.172922233	0.057632
ITK	0.120302516	0.6852402	0.645647354	0.0258655	0.041415439	0.88869
LRFN4	-0.002832421	0.9922389	0.636666951	0.0259113	0.3918593	0.17359
REM1	-0.280280795	0.6215227	1.205498669	0.0259405	-0.459282591	0.419328
SCO2	0.131178009	0.286241	-0.277383279	0.0259432	-0.068678794	0.578215
ADAM28	0.241957426	0.2327243	-0.451776912	0.0259779	0.574229478	0.004601
P11-255H23	-0.265318122	0.202288	-0.462809023	0.025977	-0.030850871	0.881479
UBAP1L	-0.410191383	0.072743	-0.499103531	0.0259843	-0.591457874	0.00958
ANAPC2	-0.231606438	0.2822591	-0.479950137	0.0260831	-0.314778666	0.143586
BRD1	-0.076709385	0.4815206	-0.241223156	0.0260234	-0.083559481	0.44077
CCDC167	0.017258732	0.9168673	-0.37218985	0.0260752	-0.072510548	0.659869
NT5C3B	0.10323122	0.3592674	0.246556506	0.0260789	-0.04096451	0.715174
PCDHA2	0.173996799	0.5746234	0.648894681	0.0260587	0.190974999	0.525223
TMEM181	-0.004755739	0.9645655	-0.238211545	0.0260581	0.087022161	0.415498
CCDC71L	0.217256226	0.2991507	0.461315381	0.0262011	0.159418472	0.445385
SLCO5A1	0.040804628	0.9564414	1.582201145	0.0262124	1.149697032	0.110243
PDZRN4	0.008550559	0.9835004	0.906597792	0.0262299	-0.702126026	0.093397
OBSCN	-0.044804332	0.7880049	-0.370058391	0.0262648	-0.570643162	0.000631
TAF1C	0.259011406	0.1387914	-0.391488223	0.0263255	-0.676014067	0.000146
ETNK1	0.021926224	0.8834151	-0.33202191	0.0263719	-0.08391698	0.57451
HTRA1	0.073247337	0.819021	0.701590358	0.026369	0.13262131	0.67765
UST	-0.06047661	0.8471272	0.685551547	0.0263495	0.531697889	0.086479
VNN2	0.36228289	0.468551	1.048005304	0.0264351	0.685841362	0.146416
MT-ND1	0.416174942	0.154914	-0.649294619	0.0265706	-0.651110863	0.026165
AGR3	-0.147702808	0.4467303	-0.430630376	0.0266246	0.060775296	0.753759
OVGP1	-0.107079637	0.6668043	-0.558202979	0.0265938	-0.458103581	0.068453
TMEM212	-0.026850255	0.8995329	0.46874427	0.0266181	0.133278227	0.529727
OLFM1	0.41580003	0.3715798	0.962828472	0.0266762	0.455169014	0.309721
SLC1A5	0.045389111	0.765519	-0.33759859	0.0266682	0.053806237	0.723425
AKAP8L	-0.016808972	0.867133	-0.222723425	0.0267161	-0.312205841	0.001977
NDRG1	0.344057055	0.2420635	-0.651654301	0.0267356	-0.253984673	0.387858
RWDD4P2	0.74869994	0.2359302	1.38745803	0.0267253	1.647763262	0.008436

TEX10	-0.098969675	0.4191098	0.269387322	0.0267105	-0.082259661	0.500387
HSD17B1	0.141390146	0.3661876	-0.347334341	0.0268456	-0.253414451	0.108599
PTCH2	-0.039712593	0.8972128	0.677523253	0.0268458	0.691783768	0.023843
TD-2005D2(	-0.230543391	0.707281	-1.543180342	0.0268851	0.291741688	0.61721
TMF1	-0.020116187	0.885529	-0.309126805	0.0269006	-0.013122081	0.925117
MED19	-0.138424946	0.2423101	-0.258631625	0.0270081	-0.061733042	0.59538
EEF1A1P3(	0.404384686	0.2926652	0.821546068	0.0270471	0.55092528	0.145072
FECH	0.247619709	0.1521377	0.379010899	0.0270723	0.278342695	0.105332
PLXDC2	0.042091606	0.8521487	0.498001228	0.0270725	0.135180423	0.549168
SP3	-0.071257689	0.2324631	-0.131493831	0.0271237	-0.020484512	0.730591
ANKRD62	0.213048318	0.6619451	0.991935956	0.0272138	0.208910347	0.663939
CFTR	0.160247614	0.7065382	0.916238747	0.027203	0.499641963	0.231888
SATB1	-0.049496331	0.7915756	0.412232903	0.0272663	-0.158007335	0.399031
ZYG11B	-0.14291551	0.0960159	-0.188664622	0.0273141	-0.16829041	0.049733
MPG	-0.263582135	0.1106211	-0.362004346	0.0274377	-0.061895695	0.704713
NXPH4	-0.122228014	0.8286542	-1.285994987	0.0275729	-0.108474233	0.84713
HOXC6	-0.611139535	0.1578851	-0.956490163	0.0276276	-0.945804773	0.030136
RP1L1	0.059174553	0.9064048	1.023421311	0.0276215	0.45178695	0.347357
RPL7P36	-0.054798732	0.9345991	1.296574735	0.0276371	0.817485051	0.179559
PWP1	0.062610699	0.5035988	0.2042372	0.0277508	0.197368697	0.033748
C17orf70	0.202767514	0.1816463	-0.334780403	0.027865	-0.198300865	0.193549
CA5A	0.002020607	0.9937625	0.551976268	0.0279658	-0.227623052	0.380496
MORC4	-0.114138264	0.5680468	0.438924839	0.0279623	-0.01417075	0.943469
SLC38A11	-0.908728807	0.0516067	-1.026661879	0.0280067	-0.985648505	0.03357
DOK4	-0.290162296	0.0876356	-0.372610044	0.0280437	-0.378233089	0.025866
LDB3	0.454671707	0.3178204	0.985526173	0.0280679	-0.009671902	0.983097
UBE2FP3	0.080750806	0.8509937	-1.015585539	0.028147	-0.747903077	0.100055
JAGN1	-0.002671698	0.9795487	-0.228811422	0.0281717	0.053573902	0.603976
TRIM13	-0.130829544	0.2178842	-0.231129588	0.0282002	-0.037909474	0.719481
PICALM	0.087745646	0.4049952	0.230733746	0.0282998	0.280034042	0.007757
NDUFB6	-0.332068688	0.1673425	-0.524257149	0.028346	-0.143807536	0.546137
SYT15	0.417805214	0.0738008	0.509501628	0.0283541	0.688948879	0.003167
ARVCF	0.203399138	0.3227891	0.446416137	0.0284124	-0.113785196	0.582202
TBC1D14	-0.204050505	0.0679819	-0.244691979	0.0284267	-0.101650251	0.362524
RABL6	-0.143772082	0.1166087	-0.199947651	0.0284765	-0.164038196	0.072605
CAMTA1	0.14901397	0.292629	0.306138523	0.0285302	0.260355347	0.064712
AP1G2	0.027579803	0.8331192	-0.287058125	0.0286438	0.008629288	0.947412
ITFG1	0.126647317	0.318748	0.276906785	0.0286953	0.24209263	0.055947
GMNC	-0.745426965	0.1226942	-1.02916076	0.0287396	-0.782531607	0.09647
CAPS	0.036076989	0.8757804	-0.505173472	0.0288167	0.093098649	0.68615
DPP10	0.094911057	0.6952707	0.527491597	0.0288091	-0.45332587	0.062558
KIFC3	-0.102951417	0.5173009	0.342671523	0.0289467	-0.205535321	0.195713
COG1	0.294449359	0.0516312	0.32893089	0.0290349	0.047999266	0.751821
ARMC4	0.888765485	0.134274	1.266138608	0.0291156	0.835623051	0.159385
LPAL2	0.68983472	0.1481508	1.022596874	0.0290887	-0.123061813	0.801278
P11-82O19	1.018057146	0.0787547	1.222976568	0.0290958	1.109214115	0.051168
SYT2	0.613636494	0.1029732	0.807963059	0.0291255	-0.301074293	0.442799

EPHA5	1.336897139	0.0565244	1.502166201	0.0292558	1.160188525	0.092614
TD-2311M21	0.128481413	0.7630446	0.901508141	0.0293781	0.289249739	0.490842
HAND1	0.686673883	0.0789431	0.848465025	0.0293676	0.081175044	0.836357
PODXL2	0.214450851	0.414922	0.564034754	0.0293688	-0.273833956	0.303901
SERPINA6	0.7083787	0.3382423	1.562151384	0.0294332	-0.760359907	0.35302
TAF9B	0.07932691	0.5853697	0.31413032	0.029423	0.237535378	0.100593
SVEP1	0.1155195	0.5499103	0.420063662	0.0294822	-0.21530844	0.265598
CPAMD8	-0.041629464	0.8251043	-0.410044056	0.0295449	-0.405225251	0.031596
TMEM44	0.174231618	0.2618413	-0.34164322	0.0295374	-0.103978624	0.50391
TP53I13	-0.140651646	0.4105542	-0.371474504	0.0295957	-0.378068239	0.026862
ANKRD35	0.093445143	0.7839542	-0.795173179	0.0296457	0.235646693	0.480411
SMYD4	-0.298168413	0.0940317	-0.383868631	0.0297338	-0.763390034	2.08E-05
TNRC6B	-0.088307367	0.443592	-0.250418022	0.029799	-0.344983598	0.00282
FGFR1OP2	-0.105610649	0.2338399	-0.191576076	0.0298536	-0.090398528	0.305677
P11-752P2.	0.767468918	0.2081054	1.271229261	0.0298553	1.405576807	0.016155
RPS8	-0.104410713	0.3618598	-0.248656256	0.029876	-0.080945626	0.479563
MSMB	-0.13210261	0.8203841	-1.264026527	0.0299096	0.704156665	0.22602
PRKCD	0.013310485	0.9302201	-0.330941394	0.0299121	-0.000573001	0.996984
CNN3	0.109182398	0.4711055	0.328633316	0.0299403	0.235224713	0.120362
ASTN2	0.031925194	0.8901238	0.500696819	0.0299555	0.027724347	0.904463
P11-1114A5	0.286945412	0.6477322	1.25053596	0.0300298	0.300107128	0.627956
ZNF317	-0.112620324	0.31917	-0.244484694	0.0300207	-0.121314084	0.282684
THRAP3	0.003609214	0.9657872	0.182092424	0.0300817	0.18913241	0.024292
PDE3A	0.103438987	0.5685903	0.391615297	0.0301784	0.113314689	0.531965
ADRBK2	0.171264839	0.428245	0.465909419	0.0302125	0.145318125	0.501773
RGAG1	0.13628163	0.7828413	0.991732798	0.0302647	0.09548164	0.845721
C16orf52	-0.118180454	0.3014546	-0.24706233	0.0302828	-0.136086955	0.233061
MED8	-0.21553007	0.0917722	-0.275803919	0.0303393	-0.168834818	0.184954
SCN7A	0.197849685	0.6284935	0.878964984	0.0304098	-0.550682845	0.183692
PDZRN3	0.258152779	0.3968415	0.657504519	0.0305368	-0.062879118	0.836673
VCPIP1	-0.083275317	0.3231795	-0.181466504	0.0305294	-0.089418268	0.286547
WNK1	0.206801388	0.2026099	0.350377521	0.0305618	0.083942302	0.604723
LRP6	0.079560201	0.5341244	0.275565941	0.0306005	0.115425979	0.366043
SLC5A3	-0.224977091	0.0973244	-0.29294274	0.0307186	-0.368707823	0.006584
SNRPC	-0.038014921	0.7382586	0.243278456	0.0306992	0.224065314	0.04705
UBAP2L	0.261335077	0.0764877	0.318411695	0.0307152	0.190673366	0.196076
FBXO41	-0.060117174	0.8446632	-0.66573846	0.0307602	-0.685291011	0.028593
SNAI2	0.264212305	0.3382977	0.594321052	0.0308409	0.320388471	0.24514
TD-2063L20	-0.111298267	0.8239341	0.949380513	0.0308875	0.781313317	0.081673
PATE4	-0.061845918	0.8589291	0.689924808	0.0308805	-0.123568874	0.721067
SMAD7	-0.370030943	0.1319144	-0.529247002	0.0308707	-0.145026705	0.552886
UTS2B	0.515836293	0.087925	0.632097604	0.0309106	0.471076861	0.114115
KLF14	0.38953987	0.4962131	1.182014852	0.030982	0.198211862	0.729934
ERCC1	0.253732523	0.0526701	0.281272688	0.0310162	0.224958501	0.085412
HEMK1	-0.225669981	0.1521126	-0.338474153	0.0310248	-0.387203414	0.014273
OXR1	-0.161020881	0.1841402	-0.260483094	0.0311204	-0.127398995	0.291857
C2orf72	-0.27824545	0.293981	-0.570812872	0.0311451	-0.324962491	0.219798

THBS4	-0.052183944	0.9535089	1.904917614	0.0311919	1.420743616	0.109315
IL10	0.496927361	0.317048	1.004922423	0.0312095	0.096784719	0.849798
TRMT2B	-0.039350139	0.7356169	-0.250161494	0.0312572	0.012702115	0.911974
IFI30	0.496080973	0.2034574	0.828151264	0.0313377	0.720698389	0.062406
PCSK2	0.693000597	0.2864988	1.352619188	0.0313723	0.326406282	0.619818
CLEC4E	0.0649709	0.9144508	1.201135456	0.0314324	-0.365053634	0.563021
ENKD1	0.099444952	0.7188411	-0.592613271	0.031448	-0.58001086	0.036999
SDHA	-0.141672822	0.1071349	-0.188359559	0.0314448	-0.018011081	0.837015
EYS	0.153688067	0.5041978	0.486907814	0.0315046	-0.29382245	0.203389
FREM2	-0.092154431	0.6235027	-0.403891193	0.0315042	-0.056190618	0.764617
MINK1	0.026856687	0.8461875	-0.297808556	0.0315657	-0.211032854	0.127691
ANKRD37	-0.202954759	0.7057345	1.126504121	0.0316097	2.41877588	3.22E-06
ANXA6	0.327641659	0.1176701	0.449534472	0.0316388	-0.04009147	0.848263
RNGTT	-0.136132599	0.2632167	0.255788777	0.0316802	0.171505409	0.152189
ZC3H3	0.193275566	0.2675402	-0.382572179	0.0316885	-0.302550194	0.089082
GOLGA2P7	-0.060062927	0.5646243	-0.22355292	0.0317467	-0.107717921	0.300947
AC016712.2	-0.383672511	0.4676131	-1.195883869	0.0318077	-0.549122783	0.299561
METTL13	-0.224026343	0.135641	-0.321336057	0.0318045	-0.415390023	0.00559
RP11-393I2.	0.087077171	0.873314	1.081805601	0.0318928	0.263823346	0.620067
ANG	-0.036020184	0.8594286	-0.437056628	0.0319391	0.097335006	0.631269
PHLDA3	-0.129807893	0.4277589	-0.351382176	0.0319271	-0.335936216	0.040262
BRD3	0.136814496	0.3762026	-0.331144442	0.0321241	-0.370265353	0.017166
MST1P2	-0.581276824	0.1800909	-0.932473247	0.0322387	-0.93981706	0.031604
NAA11	0.750400494	0.1938026	1.163291926	0.0322428	1.386703416	0.010682
NRSN2	0.756190814	0.1062211	0.962981177	0.0322224	0.657169796	0.144991
PIN1	0.202268888	0.0839548	0.247821074	0.0323653	0.192436934	0.098234
VOPP1	0.016354694	0.876588	-0.224853017	0.0323539	0.008422159	0.936004
FAM134C	-0.130173782	0.1864011	-0.210419841	0.0324468	-0.250584003	0.011057
GCSAML	0.517655312	0.2007103	0.827632077	0.0324358	0.446689989	0.266505
SSTR1	-0.539683953	0.0942634	-0.68673738	0.0325038	0.222780312	0.475478
MYLK3	-0.135274331	0.5980374	0.53670224	0.032527	-0.002588772	0.991919
PBX1	-0.011447533	0.9405866	0.327146615	0.0325307	-0.031143152	0.839198
AIMP1	-0.212096017	0.0586931	-0.23883192	0.0326571	-0.119707986	0.282728
METTL21EP	0.458108297	0.4149153	1.127340917	0.0327131	0.553331809	0.313821
PPP1R26	-0.170846514	0.2458601	-0.313192116	0.0326958	-0.047920161	0.742155
TMEM45A	-0.012904046	0.941189	0.372951317	0.0327209	-0.166005678	0.342517
DOK3	-0.371915732	0.1809566	-0.602626463	0.0327897	-0.599496509	0.032947
RPS6KL1	0.077681298	0.8122982	0.681701475	0.0327919	-0.490390231	0.139352
CCDC115	-0.158847509	0.2573406	-0.297656122	0.0329004	-0.071303719	0.608317
PTPN13	-0.058370528	0.7071019	-0.331176592	0.032978	0.020945371	0.892599
C7orf49	-0.026752192	0.8670842	-0.340295009	0.0330235	-0.2977209	0.06239
HRH1	0.279387503	0.4824347	0.834458866	0.0330599	0.643570398	0.102458
IL7R	0.530234634	0.0805342	0.642398179	0.0331837	0.363642845	0.229676
TRIAP1	-0.266636916	0.0710419	-0.312725886	0.0332497	-0.383026591	0.009452
TTC21B	0.089933233	0.5241257	0.299688263	0.0332528	0.078338276	0.578524
EXTL2	0.102903974	0.4347057	0.278382287	0.0332947	0.036997613	0.778219
LEAP2	0.382621836	0.1863127	0.594815542	0.0333164	0.325064338	0.25387

NRK	0.350332192	0.4030931	0.889357176	0.0333831	0.931150777	0.025924
DHX33	0.32446279	0.0605742	0.365275966	0.0334875	0.17319167	0.315095
IGF2	0.063900639	0.7262299	-0.388025999	0.0334972	-0.263064916	0.149473
UBR5	0.207722425	0.180517	0.329557923	0.0334763	0.295951037	0.056285
SLC26A3	-0.114107364	0.8993196	1.911873443	0.0335552	-2.46168493	0.007133
USP49	0.151106543	0.4652036	0.436218279	0.0336569	0.015967829	0.938459
PPP2R5A	-0.152670392	0.1572025	-0.228278542	0.0336861	-0.080337963	0.453533
C3orf72	0.083547167	0.7846927	0.615165115	0.033777	-0.054722699	0.858191
CDC25A	-0.256976987	0.1273185	-0.357574268	0.0337992	-0.152142889	0.366017
PRPF19	0.17436796	0.2550852	0.323674655	0.0339066	0.179544936	0.239859
DNAJC7	-0.049564096	0.6203134	0.210600903	0.0339218	0.131365441	0.186988
P11-529H20	0.05327941	0.8595338	0.636606441	0.033943	-0.084768924	0.778275
ARG2	0.037536637	0.8613077	-0.454108835	0.0341678	-0.457705934	0.033869
FBLN5	0.350547817	0.1862659	0.56043476	0.0341729	0.30837782	0.244822
ADCY6	-0.017611549	0.9039588	-0.309186795	0.034215	-0.213057664	0.144732
NAE1	-0.034649976	0.7283427	0.208409016	0.0342126	0.106948976	0.278626
NUDT19	-0.329135321	0.067052	-0.376342472	0.0342627	-0.218237743	0.218106
AF131215.1	-0.593980018	0.2798873	-1.210953086	0.03444	-1.258952883	0.03077
CEBPG	-0.032033256	0.7704332	-0.232080687	0.0344117	0.072472431	0.507851
NEK3	0.694485625	0.4155884	-1.926384083	0.0344314	-0.887444364	0.313921
PLAGL1	0.237149926	0.1499771	-0.350108765	0.034428	-0.134765664	0.41505
ZNF485	-0.33858094	0.0821533	-0.407042376	0.034448	-0.413921707	0.032498
GSTK1	0.121225052	0.1406622	-0.17427775	0.0344792	-0.041122336	0.617529
MYH10	0.073602949	0.6435757	0.335901921	0.0345958	-0.106885565	0.501606
LDLRAD2	-0.881456923	0.082653	-1.079343734	0.03461	-1.169560977	0.020463
ARHGAP25	-0.312615377	0.3484588	-0.711493258	0.0346567	-0.5918297	0.077901
HNRNPDL	-0.004961951	0.9489183	0.163123896	0.0348323	0.236123825	0.002257
MINPP1	-0.241661996	0.0701471	-0.277856218	0.0348047	-0.347887036	0.008697
PLEKHG6	0.016073027	0.9419773	-0.466434858	0.0347489	-0.2609059	0.237327
RIC3	-0.035456005	0.9666643	1.749253103	0.0348243	-0.081999455	0.922711
SPNS1	0.141504915	0.2574999	0.259685147	0.0348274	0.210358104	0.08961
SRSF9	-0.045894056	0.6972682	-0.248796993	0.0347276	-0.153886663	0.191733
ZNF584	-0.186522162	0.233197	-0.327704097	0.0347287	-0.279942284	0.073248
SRGAP1	-0.45593048	0.266737	-0.864212032	0.0348924	-0.186854329	0.648923
ABCB6	-0.173753027	0.2301849	-0.305594107	0.0349687	-0.189305025	0.188235
GMPR	0.143220293	0.7190016	0.795886592	0.0349872	0.093559732	0.810654
MED10	-0.049352818	0.7250945	-0.295981069	0.0349883	-0.294135959	0.037008
PEG10	0.093997762	0.6681982	0.462512207	0.0349564	0.261373906	0.234636
MRPL10	-0.011371893	0.9311959	-0.277819735	0.0350795	-0.253990043	0.054381
EIF5	0.056989887	0.7256211	0.342039514	0.0351147	0.352990628	0.029671
MT-ND2	0.195829466	0.5121117	-0.629524782	0.0351718	-0.610920216	0.040957
LINC00493	-0.103597814	0.3234532	-0.219503353	0.0353934	-0.076374655	0.462981
GRK5	-0.016193891	0.9277373	-0.376358591	0.0354283	0.053368131	0.764259
CRMP1	0.433148525	0.2184572	0.737039	0.0355847	0.279457559	0.427197
SKP1	0.074862758	0.317623	0.156688853	0.035604	0.143778578	0.054187
CDR2L	-0.27860481	0.0970529	-0.351180138	0.0356427	-0.16243333	0.330058
DCAF10	-0.21827043	0.1719365	-0.334509333	0.0356486	-0.406071988	0.010862

ARHGEF40	0.361932416	0.0716797	0.421320228	0.0358463	0.33504385	0.095348
ELOVL2	0.386510556	0.4197193	0.971750846	0.0358657	-0.341876533	0.49135
KIAA1430	-0.181312701	0.1101787	0.235884748	0.0358249	0.018879023	0.867109
METAP1	-0.135298611	0.3245001	-0.287406196	0.0358734	-0.139805863	0.30721
P11-536C10	-0.161710627	0.6624142	0.76586957	0.0358915	-0.336343427	0.364127
SREK1	-0.18024427	0.0634885	-0.2034002	0.0357742	-0.337957809	0.000502
SUV39H1	-0.252891466	0.270607	-0.479928735	0.0358555	-0.405836227	0.075973
TMEM238	0.517606233	0.1924095	0.80410502	0.0358866	0.982674993	0.009994
LPCAT4	-0.38104557	0.061058	-0.424348305	0.0359093	-0.051163485	0.7995
TTC14	-0.062830783	0.6026103	-0.252230274	0.0359793	-0.20957092	0.082122
QTRTD1	0.257113892	0.1404829	0.365258452	0.0360035	0.353533545	0.042448
OC1S5-TXNI	-0.433304594	0.1001884	-0.552181553	0.0360515	-0.303957902	0.248156
CCNB3	0.293164434	0.6709164	1.439026876	0.0360428	1.153321575	0.087431
CA7	0.327124606	0.8205055	2.986133358	0.0361407	-0.381035441	0.792574
TAC4	0.611046896	0.2099153	0.981089686	0.0361513	0.765865905	0.106768
MFSD4	0.649435903	0.0709051	0.742191138	0.0362433	0.506243784	0.159512
C5orf28	0.11610351	0.4622578	0.323465662	0.0363426	0.284897582	0.066167
ANXA8	1.48373289	0.0683756	1.700240632	0.0363625	2.420631645	0.002801
ADAMTSL2	-0.229344459	0.5885346	-0.930296186	0.0364002	-0.268407584	0.5243
DGCR6	-0.294318041	0.3302452	-0.63266214	0.0364282	-0.19333681	0.519414
LURAP1	0.656521086	0.2519212	1.143653922	0.0364211	0.36510184	0.529273
XKR9	0.102963823	0.6656093	0.490208204	0.0364073	0.255631182	0.279675
WDR59	0.186865875	0.1954648	0.301343606	0.0365133	0.168445145	0.244313
THRA	0.101277307	0.3854906	-0.244781529	0.0366039	-0.095251523	0.414536
SLC27A4	-0.171843176	0.2807676	0.32189672	0.0366714	0.075193744	0.630299
ASB7	-0.045663494	0.7101338	-0.255101715	0.0367173	-0.28298605	0.020761
FBN3	0.337745675	0.5308273	1.122343317	0.0368599	-0.344269518	0.52329
ZBED4	-0.019989097	0.808983	-0.172108474	0.0368546	-0.13110443	0.112479
SEC14L6	0.446074443	0.0981679	0.547718099	0.0369093	0.402279423	0.132712
AKAP17A	0.033450335	0.783252	-0.254115456	0.0369287	-0.113269096	0.351763
NUPL1	0.146226987	0.0708901	0.168206882	0.0370632	0.351730323	1.28E-05
CMTR2	-0.070612144	0.4749713	-0.204474522	0.0371688	-0.192365801	0.050223
CYGB	0.137608603	0.599756	0.539658976	0.0372487	0.118977854	0.648915
MOGS	0.006231717	0.9649982	-0.295885157	0.0372408	-0.115206319	0.416956
IGF2R	-0.177598279	0.278825	0.340941584	0.0373501	0.170310551	0.298561
KIF6	0.244674933	0.5699298	0.880862012	0.0373391	0.19130111	0.655061
TRHDE	-0.306372996	0.3724601	0.709219649	0.0374084	0.021701724	0.949549
PDLIM1	0.204865238	0.0960106	0.255228265	0.0374356	0.428849818	0.000463
KLHL3	-0.201817912	0.3324335	-0.435681291	0.0375431	-0.400562446	0.055536
RFTN1	-0.241587086	0.2740756	0.452951363	0.0375544	0.106371304	0.627348
THSD4	0.311712466	0.2386684	0.547202969	0.0375613	0.027081196	0.918649
TMEM60	-0.153347882	0.330322	-0.325870109	0.0376093	-0.26198179	0.095509
PIGP	-0.16367944	0.2654675	-0.299684925	0.0376243	-0.153040437	0.29154
PARP15	0.027190421	0.916223	0.532302244	0.0376492	0.153883604	0.550046
P11-153M7	0.055063014	0.8630575	0.654823365	0.0376579	-0.113606958	0.72208
CDH12	-0.61399809	0.1150497	-0.809413748	0.0377251	0.084203258	0.827595
TMEM200C	0.039691236	0.9159836	0.772281542	0.0377866	-0.144328745	0.701046

P11-597D13	-0.219293381	0.6739351	1.025281253	0.0378444	0.125309965	0.806792
ZNF19	0.122001773	0.5789561	0.447070342	0.0378427	0.299593393	0.171926
ZNF37BP	-0.013104443	0.9477588	0.411668037	0.0378687	-0.018918636	0.924473
BDH1	-0.391137023	0.1053934	-0.496149426	0.0379889	-0.359495406	0.133865
COL11A2	0.62902896	0.2308034	1.085031133	0.0380749	0.025375039	0.961544
ACOX2	-0.585243843	0.1064551	-0.752588359	0.038122	-0.657889896	0.072887
COX16	-0.137365824	0.2555561	-0.249781296	0.0381868	-0.052444294	0.662188
KMO	-0.07162951	0.8615336	0.810693973	0.0381911	0.64801309	0.101658
CCL22	-0.078639119	0.8391756	0.746459979	0.0382781	0.375396178	0.310476
NDOR1	0.19828241	0.1775168	-0.310506661	0.0383058	-0.040535143	0.784536
FAT4	0.079737832	0.8009652	0.654533989	0.0384125	0.048754829	0.877506
MFAP1	-0.044871662	0.6688974	-0.216917035	0.0383973	0.006278958	0.952088
PIK3CG	0.67707206	0.1351705	0.911207461	0.0384244	-0.10055226	0.833985
RPS14	-0.108684882	0.3907569	-0.261778904	0.0386881	-0.117687854	0.352603
MSL1	-0.056594299	0.5107505	-0.177511744	0.0387255	-0.069861208	0.416255
ZNF571	-0.205031355	0.1658948	-0.303672464	0.0387406	-0.458171233	0.002117
CYSLTR1	0.022670992	0.9267738	0.508027715	0.0387997	-0.029589861	0.904474
AC004797.1	0.080117575	0.6829031	0.40114194	0.0388928	0.388341064	0.045916
ARPC2	0.091963315	0.3207677	0.191019813	0.0388361	0.284197211	0.002112
HCAR1	-0.249664613	0.2311081	-0.430045603	0.0388767	-0.641589918	0.002228
KLHDC8B	0.010438536	0.960782	-0.439107364	0.0388858	-0.4261335	0.045976
SLC39A5	-0.107253316	0.5413838	-0.362758001	0.0388887	-0.179868312	0.305551
SRRM2	0.059262581	0.7681826	-0.415505099	0.038841	-0.281511192	0.161614
SLC28A2	-0.128768832	0.7824962	-0.966450161	0.0389448	0.692644107	0.136005
KIAA1549L	0.231645754	0.6425274	0.991256005	0.0389796	-0.043289835	0.930507
AC079807.3	0.404656078	0.4845497	1.101900773	0.0390794	0.541737807	0.335989
CARHSP1	0.073239762	0.5955548	-0.284923583	0.0390866	0.145932202	0.287346
CLPP	0.166358675	0.3009522	0.328459071	0.0392093	0.394369209	0.013368
P11-651P23	0.289936228	0.2466964	0.511911376	0.0391913	0.606799856	0.014511
SFSWAP	0.129767913	0.4674378	0.367671335	0.0392072	0.028623587	0.872574
UCHL3	0.046567717	0.6983292	0.244293136	0.0392365	0.489017351	3.40E-05
CCDC144A	-0.218817223	0.2930768	0.421552508	0.0393152	-0.298605036	0.151284
DENND2D	0.152358604	0.5378607	-0.510364584	0.039304	-0.291676331	0.238656
MCUR1	-0.125657888	0.3668354	0.285062547	0.0393702	0.011701161	0.932828
NRBP2	-0.039518115	0.7744122	-0.28417538	0.0393592	-0.059242528	0.666145
P13-33H18.	0.330164317	0.3680144	0.716547715	0.0393937	0.193215005	0.598345
WDSUB1	-0.134927828	0.446982	-0.363752382	0.0394526	0.130454241	0.448956
POLR1E	0.062440631	0.6277508	-0.266019177	0.0395268	-0.227910629	0.078532
INSIG2	-0.144779165	0.5621725	-0.514571495	0.0395831	-0.305881853	0.220826
CIITA	0.807928545	0.0621363	0.884062786	0.0396069	0.596554477	0.169934
SSR1	-0.128091347	0.1237706	-0.170724273	0.0396557	-0.044142885	0.59515
LHFP	0.523583141	0.2156965	0.866174397	0.0398004	0.82268382	0.051046
P11-48B14.	-0.573607171	0.2228896	-0.979427223	0.0398568	-0.708201465	0.132224
MUL1	-0.006600043	0.959471	-0.266995459	0.0399346	-0.208153655	0.109649
DNAJC28	-0.198156171	0.6808373	-1.037455528	0.0399954	-0.025997901	0.956269
GPR124	0.33200053	0.1825456	0.511100423	0.0399659	-0.167537068	0.501654
MRPS18B	-0.121035294	0.1954629	-0.190767646	0.0400092	-0.180656518	0.052404

SCAF11	-0.100408452	0.2873564	-0.19346302	0.0399845	-0.005055815	0.957181
SLC2A1	0.035254399	0.9000204	-0.576294371	0.0400429	-0.303043668	0.280194
CYP26B1	0.422146715	0.1911921	0.656022832	0.0402279	0.73017188	0.022254
ZNF568	-0.178241526	0.5810619	-0.658918192	0.0402378	-0.627678616	0.051441
L3MBTL1	-0.302439183	0.1327709	-0.410446616	0.0402706	-0.583141186	0.003968
FGF13	-0.069993395	0.8496253	0.752487157	0.0403269	0.579316472	0.11596
TUBA1A	0.269794371	0.3170814	0.552754641	0.0403133	0.236186089	0.381075
RNF186	0.103145137	0.8776085	1.362903171	0.0404199	-0.387300021	0.564242
ANKRD54	-0.203830829	0.1599702	-0.294249024	0.040504	-0.193504747	0.179149
CHMP1B	0.19488252	0.0582396	0.209875083	0.040507	0.322118037	0.00166
RRAS2	0.077512798	0.4989298	0.232474065	0.0405272	0.377937914	0.00086
VIM	0.19240077	0.3340598	0.407996125	0.0404732	-0.056963572	0.774899
KIAA1107	-0.105294671	0.5551318	-0.365978662	0.0406534	-0.702658099	0.000158
PAK7	-0.805227048	0.2463262	1.285112562	0.0407008	0.054666422	0.933363
TP53I11	0.179122951	0.2051901	-0.290933161	0.0406879	0.095747149	0.498278
WNT5A	0.273527402	0.2834557	0.521126731	0.0406772	-0.163325842	0.521944
DDX41	-0.031257668	0.7612159	-0.209534353	0.0407457	-0.165763949	0.106395
MAT2A	-0.149605768	0.216197	-0.247328378	0.0407455	-0.310092101	0.010359
LPAR5	-0.235627437	0.4620631	-0.670533852	0.0409428	-0.775575568	0.018942
SIRPA	-0.001692009	0.9941132	0.460704609	0.0409367	-0.731644927	0.001791
NPY2R	0.339511814	0.6361865	-1.559513215	0.0410153	-0.828097769	0.263059
TBC1D1	0.257503699	0.0967804	0.315670835	0.0410444	0.231868892	0.134303
MEST	0.129927957	0.5943566	0.49813257	0.0411156	0.074179631	0.761082
UNC5CL	-0.008312771	0.9677035	-0.419778914	0.0411457	-0.091962239	0.653682
ARRDC1	0.057472063	0.6982475	-0.303768755	0.041199	0.121230528	0.411748
SLC17A9	-0.046460923	0.8174525	-0.411857177	0.0412186	-0.045612841	0.82061
SV2B	0.219727676	0.717066	1.202956733	0.0412178	0.023134588	0.969636
TCF21	0.385353634	0.146475	0.539985831	0.041248	-0.4145022	0.120868
UBE2D3	0.087736538	0.3609513	0.195771394	0.0412334	0.285373531	0.002924
SPATA24	-0.499221441	0.0903423	-0.588814634	0.0412812	-0.339770698	0.236237
THY1	0.442837876	0.0535243	0.467511487	0.0412918	0.070738851	0.758261
ANKRD13D	-0.134983003	0.3500795	-0.29363437	0.0414674	-0.447229797	0.00206
SAP130	-0.113461675	0.3193006	-0.231364477	0.0414797	-0.331722561	0.00371
ADAMDEC1	0.663539986	0.3087009	1.322690862	0.0415086	2.056589003	0.001497
AMOT	-0.087070406	0.6354086	-0.374473192	0.0415181	-0.539060031	0.00339
MT1G	0.831213623	0.4808513	2.393929737	0.0415824	0.439971199	0.709198
TUBGCP2	-0.001794525	0.9820684	0.160667528	0.0416056	0.036180511	0.647402
GLUD2	-0.232599129	0.3924637	-0.555273283	0.0416222	-0.336991452	0.214943
PLA2G4B	-0.243843228	0.6003941	-0.948835401	0.0418068	-0.231152497	0.621875
SURF1	-0.681042355	0.1817959	-1.035923699	0.0418161	-0.180435767	0.711125
P11-262H14	0.419963698	0.543786	1.29253283	0.0419034	0.911413815	0.164244
WDR83	-0.020267823	0.916964	-0.397118091	0.041947	-0.445931856	0.023744
MFSD5	0.03920723	0.7908956	-0.300930167	0.0419797	-0.106106395	0.47197
KLHL15	0.019831622	0.8791192	-0.265188447	0.0420654	-0.140712616	0.280613
MED26	0.024292608	0.8998357	-0.395760682	0.0420678	-0.124818621	0.520151
PINLYP	-0.178032466	0.5406417	-0.587849468	0.0420749	-0.218175862	0.449403
ESRP1	-0.291798151	0.0991638	-0.359576661	0.0421241	-0.094735659	0.592172

KCNMB1	0.274426702	0.3096108	0.536361196	0.0422481	-0.189296936	0.488244
SEC24C	0.020910193	0.8228118	-0.189456663	0.0423502	-0.128256002	0.169552
CIRH1A	0.152328044	0.3330995	-0.31956243	0.0423816	0.099690481	0.525844
FJX1	0.511089222	0.172155	0.751688096	0.0424224	0.388613856	0.298173
COL4A3BP	-0.117741568	0.2802636	-0.220518715	0.0425443	-0.140699242	0.195901
TCTN3	0.027543664	0.7865584	-0.206136556	0.0425879	-0.259762826	0.010881
P11-137H2.	-0.230517521	0.058538	-0.244815727	0.0426639	-0.352203364	0.003821
SLFNL1	-0.44173267	0.1883896	-0.689624247	0.0426518	-0.633152381	0.059874
AGPAT1	0.224359495	0.0691563	-0.250793842	0.042712	-0.200858931	0.10481
NPIPA7	-0.057629138	0.6732065	-0.277775622	0.0427571	-0.215530026	0.11606
FBXW10	0.625550193	0.1983824	0.968199356	0.0428059	0.587751665	0.221938
TGFA	0.555236175	0.0694322	0.616268852	0.0429402	1.347066611	8.65E-06
ANGPT1	-0.089096803	0.7454744	0.552343654	0.0429749	-0.127566332	0.64176
MMADHC	-0.091813371	0.3087087	-0.181999664	0.0429873	0.051965515	0.562204
SCHIP1	0.326497334	0.3846003	0.749520525	0.0429897	-0.213921541	0.570023
LDHAL6B	0.424663045	0.3228156	0.822936061	0.0430259	0.911882184	0.02505
PPP1R21	-0.35618185	0.0844521	-0.415952147	0.0430765	-0.212284285	0.302719
ENPP2	-0.053782501	0.8653765	0.636629267	0.0431328	-0.105653919	0.738861
GAB3	1.081155283	0.0769863	1.204962372	0.0431528	0.791663281	0.195101
PLG	0.201739344	0.7057691	1.075054097	0.0432092	-1.925682128	0.000539
SERTAD3	0.113129658	0.4146677	-0.281130857	0.0432042	-0.258442513	0.063598
WDR92	-0.310563105	0.1212667	-0.401182708	0.0432154	-0.464502548	0.020556
C4orf36	-0.003736905	0.9838093	0.366545231	0.0433407	-0.063954718	0.727766
HEBP1	-0.370162563	0.1092196	-0.46465286	0.0433586	-0.270545651	0.238243
HILPDA	0.46749539	0.3338875	0.975861022	0.0433936	2.275029759	2.42E-06
PFN1	0.200620909	0.1811566	0.302864037	0.0434224	0.438786292	0.003432
PSMC1P1	0.0011644	0.9915799	0.220883902	0.0434685	0.188269435	0.08592
SNX12	0.279783141	0.0562565	0.295318451	0.0434781	0.244716656	0.094275
C9orf47	0.532165993	0.1555985	0.748951035	0.0435257	0.006208514	0.987007
AREL1	0.156430791	0.1741386	0.231708009	0.043666	0.266043709	0.020506
THAP4	-0.097021905	0.5284773	-0.309007921	0.0436803	-0.214561376	0.162123
HMGN2P15	0.247842822	0.7802018	1.696100879	0.043747	-0.171486066	0.849251
AC006011.4	0.822382191	0.0633691	0.887925385	0.0438542	0.529228778	0.233624
MAGEA10	0.575898658	0.2279642	0.917957735	0.0437966	0.097176525	0.844393
PPFIA4	-0.229903324	0.3073222	-0.45408226	0.0438545	-0.26893083	0.234232
MRVI1	0.323061381	0.3065719	0.634868666	0.0438705	0.068420745	0.829193
LAMA4	0.528540609	0.0654059	0.577781066	0.043984	0.59426427	0.038224
SOX12	-0.152132414	0.3427544	-0.322320738	0.0439502	-0.70869661	1.36E-05
EYA4	0.395459154	0.5836412	1.446408103	0.0440283	1.072906594	0.136495
SEMA6B	0.426550538	0.2775746	0.788504869	0.0440896	0.467136334	0.234831
THEM5	-0.455823787	0.5139758	1.250792944	0.0440907	0.897794076	0.14909
FBXO43	0.020390114	0.9634723	0.826616523	0.0441681	0.296955272	0.488271
SLK	0.156697282	0.1669842	0.228061505	0.0441475	0.449286914	7.26E-05
SCD5	-0.001227854	0.9946825	0.367121707	0.0442592	0.155439787	0.39629
MTFR2	-0.513845891	0.1582566	-0.726138994	0.0442914	-0.531189088	0.137933
ATF6	0.155572525	0.1062414	0.193048127	0.0443108	0.162456093	0.090792
CBLB	-0.134864774	0.4178317	-0.334629312	0.0443887	-0.045767363	0.782793

C2orf68	0.001227048	0.9921792	-0.250311067	0.0444747	-0.042364889	0.73439
HOXA1	-0.657611507	0.1018996	-0.802042408	0.0444814	-0.93208323	0.022368
RNF175	0.478696686	0.3256657	0.910102078	0.044573	0.035954287	0.940443
SLC11A2	0.275978972	0.1464582	0.381508044	0.044583	0.396748181	0.036753
P11-419C5.	-0.297428095	0.0959755	-0.357839294	0.0446229	-0.191829631	0.278822
C17orf51	0.010410612	0.9909653	1.739598123	0.044825	0.059886156	0.948008
DNAJB5	0.362497299	0.0836553	0.416611954	0.0448849	0.157708044	0.452645
IRF8	0.114551006	0.7747786	0.785557782	0.0448478	0.706443788	0.072211
MIDN	0.259622656	0.1192771	-0.335999276	0.0448776	-0.199177522	0.234286
PAG1	-0.296687934	0.0767496	-0.336268775	0.0447328	-0.112731384	0.501035
SAMD10	-0.443306832	0.060724	-0.469016157	0.0447856	-0.000876546	0.996968
SLC25A26	-0.18411227	0.1734071	-0.267185049	0.044866	-0.287399624	0.031774
LDHAL6CP	0.438452479	0.203101	0.676246378	0.044909	0.084200154	0.809303
ARHGEF6	0.045714279	0.8629976	0.514543724	0.0449587	-0.06139146	0.816674
TRAK1	0.279110812	0.053179	-0.289711376	0.0449318	0.029644103	0.837214
UBN1	-0.053575001	0.6919666	-0.270967181	0.0449734	-0.297065748	0.028078
GPI	-0.028809719	0.8697483	-0.352226617	0.0450056	-0.120109035	0.49419
RBMX2	-0.067717836	0.6655708	-0.314429561	0.045097	-0.130559454	0.404065
DIS3	0.153096334	0.1061471	0.189317766	0.0451432	0.316118182	0.000822
DUOXA1	0.604323095	0.1727288	0.880977936	0.0451707	1.377592022	0.001432
SCIN	-0.135329228	0.6452416	-0.588825536	0.0452078	-0.248674177	0.397579
OSGIN2	-0.081571257	0.6435554	0.349135415	0.0452503	0.233290304	0.181949
TPTE2P2	0.3187488	0.4508183	0.831548004	0.0453675	0.584984979	0.166597
VWCE	0.807150336	0.0545538	0.844298287	0.0454338	0.874511906	0.03721
RASSF10	-0.128049922	0.6122947	-0.507175531	0.0454583	-0.156314713	0.535071
CCDC178	-0.638858848	0.1104973	0.729274653	0.0454978	-1.222881376	0.002409
PXMP4	-0.369939277	0.0625862	-0.395231474	0.0454841	-0.157954129	0.424178
ZNF619	-0.351082591	0.0869088	-0.402195215	0.0455347	-0.546779662	0.00727
IMPA2	0.032941579	0.8414412	-0.329301522	0.0455758	0.363165915	0.026685
SLC2A4RG	0.033462452	0.8594556	-0.377714696	0.0456121	-0.298538164	0.113457
NEGR1	0.560074729	0.0678837	0.607212225	0.0457435	0.265226304	0.385427
TMEM43	0.045170518	0.6535845	-0.201126991	0.0457307	-0.154697856	0.124618
WDPCP	0.060529255	0.7264608	0.343554501	0.0457504	0.097945608	0.570535
AEBP1	0.279250449	0.2210125	0.455213385	0.0457894	-0.062923516	0.78306
MCOLN1	0.11232579	0.5550799	0.366060581	0.0457762	-0.204507161	0.277848
SORL1	-0.427540589	0.0847974	-0.495154877	0.0458813	-0.513371468	0.03851
TD-3088G3	0.311900118	0.4488408	0.780852296	0.045939	0.208893974	0.607929
FKTN	-0.350663469	0.0561149	-0.364967714	0.046009	0.040665283	0.82411
GOLT1A	-0.195181927	0.4280128	-0.492110156	0.0461576	-0.479367881	0.052561
IL12RB1	0.493701461	0.2000976	0.747966784	0.0461221	0.022932705	0.953564
KRTCAP3	-0.15162704	0.4191591	-0.375211704	0.0461353	0.106509613	0.565322
RXRA	-0.04491285	0.7091633	-0.239701885	0.0461545	-0.110093317	0.359791
SSH1	-0.160964612	0.3596314	-0.349815267	0.0461408	-0.518673267	0.003229
ZNF573	-0.28506198	0.1055915	-0.34167394	0.0461881	-0.080361707	0.642262
SYNJ1	-0.187669229	0.7416484	-1.135052942	0.046219	-0.684540108	0.230289
DYNC2H1	0.396926876	0.1043148	0.485290576	0.0463787	0.045312686	0.852868
ANKRD29	-0.426449928	0.3038969	-0.824631885	0.0465893	-0.088721734	0.827125

CPSF4	-0.03074501	0.8176715	-0.265889302	0.0465963	-0.193373988	0.147889
ULK2	0.376009946	0.0750412	0.41879261	0.046562	0.259776277	0.217903
ZNF276	-0.188647468	0.3219062	-0.377708831	0.0465823	-0.188328306	0.321762
XYLT2	0.05301012	0.689204	-0.263930665	0.0466146	-0.249457645	0.060874
CD99	0.056516018	0.7874313	0.41588689	0.0467312	0.300967552	0.150232
FLNC	0.256854113	0.3412753	0.536052906	0.0467204	-0.304791056	0.259497
RERG	0.176170327	0.3808083	0.394559639	0.0470022	0.32662445	0.103058
SCN8A	0.445324843	0.2982392	0.84028077	0.0470198	0.790081372	0.062674
SLC35C1	-0.092756857	0.6109635	-0.361214407	0.047043	-0.223135963	0.219862
SLC45A1	0.445819639	0.3181042	0.859923509	0.0471127	0.331393339	0.461282
SPATS2	0.17838996	0.303308	0.34247026	0.0471476	0.010603468	0.951303
HOXA10	-0.707358116	0.0977329	0.819729627	0.0471747	-0.194554507	0.642563
HCN3	-0.396653178	0.1839643	-0.592128466	0.0472159	-0.363455101	0.222643
QKI	0.18665646	0.1615523	0.264179932	0.0472479	0.189289986	0.155707
KDM3A	-0.05427463	0.7506266	0.337554035	0.0473035	0.49879748	0.003338
NAALAD2	0.181925303	0.5902378	0.665938247	0.0473374	0.009355957	0.977892
PLD3	0.178657609	0.256965	0.3123283	0.0474661	0.046904702	0.766077
AKAP10	-0.182553251	0.1669685	-0.260710981	0.0475446	-0.189550381	0.150154
ABAT	0.196064575	0.2075649	0.306861158	0.0475903	0.196753238	0.205094
ACER2	-0.001473719	0.9944563	-0.420574826	0.0476401	0.292663404	0.166679
CCDC78	-0.247782197	0.4621341	-0.670632674	0.0476655	-0.336247571	0.320221
AP1S3	0.196585159	0.2370748	0.326296907	0.0477155	0.550434165	0.000814
EEF1A1	-0.182331598	0.1229665	-0.23398974	0.0477628	-0.043241792	0.714504
RAB26	-0.095054231	0.7000813	-0.497577204	0.0479844	-0.33127453	0.186264
STK33	-0.106621603	0.6783193	0.49855563	0.0480518	0.016893515	0.947291
UBAC2	0.077281143	0.4191577	-0.189003375	0.0480791	0.02100124	0.825902
TNC	0.736479088	0.1084261	0.906471147	0.0481386	1.23652162	0.007021
LAPTM4A	-0.091328604	0.4660517	0.24687744	0.0482419	0.133253403	0.286657
PHLPP2	0.246921538	0.1237342	0.31533171	0.0482777	-0.083562869	0.60307
ZNF630	-0.106850306	0.7055174	-0.572599595	0.0483309	-0.196982291	0.488496
LZTR1	-0.185764128	0.2598424	-0.324998233	0.048392	-0.37295683	0.023751
NPLOC4	0.110495624	0.2228174	0.17830109	0.0484111	0.224560277	0.012975
ALG2	-0.057390843	0.6365421	-0.239452858	0.0484405	-0.088708084	0.464468
ZNF600	-0.017192605	0.9315637	-0.395518978	0.0484631	-0.061974012	0.75612
ARID2	0.098532628	0.3070161	0.189307688	0.0486657	0.087950363	0.361088
GRPR	0.321480162	0.6345863	1.29174976	0.0487588	-0.167766068	0.806861
PRKCB	-0.555610056	0.4347272	1.369420459	0.0486872	-1.099066757	0.128341
P11-809N15	0.497123686	0.277649	0.864461843	0.048742	0.244447716	0.597117
RPAP2	-0.045045207	0.8049722	0.359085946	0.0486823	-0.068420139	0.707479
SLC38A6	-0.13521337	0.4776662	0.368872675	0.0487528	0.07327158	0.697896
SLC46A1	0.230986803	0.6223561	0.9062099	0.0486851	-0.282450871	0.549195
CUX1	-0.01463753	0.9157356	0.271752195	0.0489602	-0.019637966	0.887161
DTD2	0.031428255	0.7960418	0.235726907	0.048898	0.161945579	0.17785
FAM114A1	-0.153733594	0.2741384	-0.276609653	0.0489408	-0.126512564	0.367636
FOXC2	1.115481831	0.0630854	1.178023792	0.0489617	1.57774618	0.0082
HMGB2	-0.107676662	0.5179619	0.326876606	0.048891	-0.014542448	0.930321
MAP3K1	0.013565506	0.9002471	0.212003706	0.0489324	0.093264984	0.387442

ACTR5	0.018334207	0.9179048	-0.352517402	0.0490102	-0.321700878	0.073458
NOS2P3	0.14100332	0.7770788	0.905262141	0.0490647	-0.030617252	0.951154
ZKSCAN2	-0.132510858	0.3685399	-0.288586939	0.0491374	-0.347368455	0.018608
PPP1R3G	-0.098747581	0.7086306	0.493714493	0.0492651	0.355335631	0.162092
CACNA2D1	-0.423642217	0.1808634	0.617343371	0.0492992	0.264915833	0.401713
CTDSP2	0.017070634	0.9109389	-0.300232328	0.049385	-0.323630941	0.034231
HLA-L	0.195255757	0.2851313	0.348709549	0.0495823	0.196473723	0.274431
SPTSSA	0.037007071	0.7529308	0.228154868	0.0496321	0.205255216	0.078242
EFEMP1	0.139217482	0.4751357	0.381903784	0.0496682	-0.069893778	0.720021
POU5F1B	0.135836244	0.7759597	0.887133465	0.0497573	0.649309276	0.157161
APOBEC3D	0.124419001	0.6136714	-0.491074759	0.0498142	-0.211408226	0.397402
ADI1	0.060520679	0.741367	0.356469505	0.0498654	0.323022073	0.07673
NEDD8	-0.038705027	0.6151508	-0.149697083	0.0498573	0.069933982	0.358504
DOCK10	0.543919783	0.2113474	0.836550257	0.0499591	0.259308456	0.543766
ABCC8	2.453548832	0.0580895	2.994501297	0.0157239	2.017334585	0.119563
AC002310.1	1.600779598	0.1476478	2.625602559	0.0104724	3.923848612	7.56E-05
AC005077.8	2.947793949	0.0629718	3.435750795	0.0266411	2.226599237	0.171606
AC005884.1	-0.526057982	0.408789	-1.426486007	0.0346477	-1.028563414	0.116899
AC010900.2	2.104987388	0.1525699	3.124003475	0.0224691	2.937206643	0.034007
AC019109.1	-0.361168551	0.714353	1.972312538	0.0164465	0.552793496	0.533616
ALS2CR11	-2.430660322	0.0783663	2.874256661	0.0032337	-0.169802137	0.871304
ANKRD20A8	-0.269540909	0.7877144	1.841568485	0.0417491	-1.372237761	0.196655
ANO2	0.337377874	0.7911266	2.462755381	0.0370507	-0.247281996	0.864175
AP000350.1	-2.34707488	0.1674975	-4.856279629	0.0102276	-1.869769415	0.26102
APOBEC3H	1.620445177	0.1967833	2.416889778	0.0443704	-0.565857289	0.71212
AQP5	1.213969964	0.2775138	2.107985846	0.0475788	1.642137108	0.127472
ARHGAP40	0.813271476	0.538612	2.517956401	0.0411281	1.985317424	0.105482
ASCL1	1.345276338	0.4175382	3.935360506	0.0096225	2.544175782	0.103495
ATP1A3	1.356003974	0.244252	2.237973121	0.0457131	0.332098112	0.789984
BCORP1	1.353510533	0.158263	1.811275811	0.0480452	1.52567167	0.101882
BHLHE22	1.762188743	0.3688663	4.130174233	0.0175408	1.656556984	0.399596
BIN2	0.765411346	0.5753403	2.949673885	0.0093562	2.188587968	0.065922
BPI	1.608653204	0.1621665	2.306568015	0.0358263	2.005824722	0.067331
BTK	1.070572851	0.5807269	4.991275579	0.0045968	2.407165985	0.192005
BTNL2	1.830258607	0.0987094	2.178455621	0.0463079	0.554658446	0.639266
C10orf71	1.824727036	0.1388199	2.47336081	0.0387998	1.06519098	0.402831
C2orf71	0.336161776	0.886906	4.450608604	0.0395036	1.671898718	0.45889
C2orf80	0.8413226	0.2784601	1.649555348	0.0223547	0.710239372	0.360005
CCDC144B	0.326870458	0.7163525	1.646676402	0.0448859	1.288627986	0.122476
CD52	-0.115577711	0.8509417	-1.336025871	0.048078	-1.537544131	0.028482
CDSN	-1.280698788	0.415138	-5.05866203	0.0051352	-0.709282476	0.645442
CELF3	-0.458989279	0.6094371	2.316523157	0.0025494	-0.518365266	0.568769
CHRNA9	-0.273341975	0.910462	4.75454576	0.0326631	1.195010445	0.61573
COX6CP16	1.057514813	0.6063137	3.814339978	0.0381877	1.651514887	0.419735
CPLX1	-0.052822346	0.9301711	1.438746914	0.0075609	0.533015812	0.335732
CTB-1048E9.	0.238016356	0.7490883	1.344845046	0.0425335	0.895466321	0.193396
CTB-167G5.	0.619925564	0.3149467	1.190410364	0.0432303	0.135950498	0.830202

TD-2161E19	1.793233416	0.3405096	3.399950316	0.0437386	3.234786679	0.057493
TD-2194F4.	1.057520682	0.527363	3.871857111	0.0057578	3.232290826	0.025869
TD-2535L24	0.336070421	0.8979567	5.371044542	0.0270228	4.38512918	0.072993
CXCL9	1.057501971	0.6340345	4.05578546	0.0445618	1.629991247	0.462444
CYCSP10	-0.181935962	0.7949962	-2.006251322	0.0184122	-1.466242911	0.065132
CYP2D7P	-0.0791573	0.9403495	-2.326754427	0.0480228	-0.503326676	0.637728
CYP2G1P	2.910046631	0.0768321	3.563999442	0.0241268	2.369392545	0.159822
DCSTAMP	1.694324197	0.2013636	2.681644516	0.0369689	1.591622043	0.232757
DLGAP2	2.674480274	0.3780471	7.342369446	0.0119106	1.449331333	0.636071
DLX6	1.018522037	0.5832949	4.330947312	0.0066558	4.049444591	0.011825
DNASE1L2	-0.256177899	0.6358482	-1.329882629	0.0217	-0.573377692	0.300644
FAM204BP	-2.20608657	0.0606751	-2.427188009	0.0386002	-1.934570413	0.079013
FAM216B	1.210033276	0.1571852	1.60226183	0.0494343	2.135821146	0.007266
FAM35CP	-0.096104416	0.9390551	2.547772759	0.0099074	2.001606756	0.048532
FAM86B2	-0.500916486	0.7802236	-3.893232649	0.0496187	-0.121535479	0.94511
FBXW11P1	2.666471167	0.1479158	3.59003554	0.0409573	3.25205498	0.067931
FPR2	1.769440924	0.240802	3.263612356	0.0202867	2.012631767	0.168937
FRG2	-0.079290594	0.9390311	-3.296729799	0.0128937	-0.173663426	0.86633
GAPDHP39	-0.757073865	0.3287966	-1.815090048	0.0400556	-0.054654553	0.938244
GPR112	-0.58539342	0.6794618	2.852418316	0.0147837	0.301862288	0.814975
GPR141	0.895196767	0.2261313	1.696787726	0.0161628	1.56943453	0.029795
GPR88	2.763948138	0.1185247	3.546858202	0.0362449	3.798941175	0.024234
GRK1	1.610346335	0.3712446	3.715088153	0.0175837	1.991370179	0.247751
GS1-21A4.2	0.33618935	0.8450905	2.980415936	0.0422408	2.521969271	0.09363
GSG1L	0.43142883	0.7619064	2.790314996	0.0269964	1.841443189	0.167088
GZMA	2.395610124	0.2080692	4.582725108	0.008895	6.39904715	0.00021
HMGB1P35	1.641190242	0.2830673	2.982094779	0.0295278	1.505423863	0.324841
INRNPA1P4	2.772501586	0.0948562	3.558956437	0.0241039	2.652122316	0.109678
HSPA8P18	1.953713516	0.1208462	2.422022859	0.0446818	1.204089259	0.365004
IL9RP3	-1.679050174	0.1802151	-3.205350591	0.0203908	-0.200663424	0.848176
KB-1589B1.4	-1.069279262	0.4731685	-3.77825627	0.0227678	-2.228778181	0.177484
KLKB1	1.067171685	0.1870721	1.616765415	0.0417126	1.236902122	0.124032
KRT8P17	1.057514107	0.5839435	4.020021606	0.017756	3.242185969	0.062587
LY6K	1.39028432	0.1290478	2.048809456	0.0154559	1.982953633	0.021948
MAP2K4P1	0.056200173	0.9418409	1.326001491	0.0490476	0.36450911	0.620154
MARK2P9	-0.857685533	0.3471537	-3.366136952	0.0063043	-0.549196258	0.522795
MCF2	1.391509588	0.0977359	1.827773221	0.0215015	0.024246146	0.978567
MIPEPP3	-0.807400025	0.1791328	-1.932707544	0.0055445	-0.136205836	0.807032
MTND2P26	1.768542931	0.27364	3.705647943	0.006442	2.64900201	0.069843
MTNR1A	1.560167708	0.1142989	2.194263606	0.0201304	0.534045334	0.616195
MYCT1	-1.875003669	0.1493241	1.925185977	0.031975	2.179558672	0.016694
NDUFB4P2	0.863216399	0.3055252	1.711318269	0.0305897	2.398091175	0.001985
NPY	1.183287556	0.1340959	1.744022302	0.0216761	1.023815025	0.195087
NPY6R	1.273322399	0.1600403	1.783610866	0.0411393	0.32435456	0.733538
NUDT19P5	1.044443363	0.5485202	2.961924896	0.0494592	-0.522692961	0.765735
NXPH1	0.050889518	0.9689215	2.849882468	0.01537	1.51098003	0.207372
OCLM	1.091185	0.0891451	1.272653146	0.04054	-0.048893015	0.945746

OR2A1	0.6822639	0.4863723	2.303922573	0.0095341	1.21204734	0.194644
PANX2	-0.346288234	0.6534576	-2.060444531	0.0470427	-0.155393424	0.837705
PAX3	2.19645891	0.1686229	3.578778228	0.0194402	1.502799363	0.364002
PNPLA5	0.582175565	0.5250096	1.903827396	0.0179163	1.99849099	0.012971
POU3F4	0.871969745	0.602396	3.22133938	0.0299327	-0.517162102	0.769763
PPIAP3	-0.434581336	0.6990872	-2.992569916	0.0326169	0.005402943	0.995981
PSG5	0.801387282	0.3894172	1.81323119	0.0306999	1.427462248	0.114373
PTMAP4	0.76853797	0.3885179	1.679171062	0.045872	0.923452523	0.291099
RBMX2P3	-0.461203605	0.6698266	-2.763066667	0.0395254	-0.628746293	0.562556
REG3A	-0.808937646	0.5994556	2.646126529	0.0288508	0.155703283	0.908757
RHOT1P3	2.094741716	0.288498	4.226592556	0.0267298	4.710220276	0.013367
RP1-154J13.	2.151768273	0.0866924	2.666459887	0.0272795	2.874423333	0.016842
P1-214M20	2.046095433	0.16064	3.296199035	0.0137324	2.891681996	0.03422
RP1-308E4.:	-2.949520486	0.0534735	-3.185431533	0.0370212	-1.771799978	0.208166
P11-115L11	-2.092845139	0.096261	-3.047326065	0.0171456	-2.225760861	0.079925
P11-129B9.	1.576746044	0.0726847	1.927924378	0.0224353	1.045219536	0.250493
P11-133K1.	1.501673585	0.1746403	2.091280582	0.0482558	1.413543038	0.199864
P11-162A12	-0.430252716	0.4826377	-1.500888053	0.0281777	-0.323112942	0.588809
P11-165H4.	0.615531235	0.5492439	2.046719541	0.0235576	1.490543369	0.112157
P11-16F15.	-1.406591648	0.2713266	1.793079578	0.0377828	1.261411405	0.157796
RP11-17A4.:	0.629686509	0.4280808	1.554902723	0.0301575	1.882929023	0.006651
P11-214N16	0.423733742	0.7119474	-3.710319298	0.0117279	-2.160364353	0.140294
P11-267J23	1.766804708	0.2959952	3.683992302	0.0110375	2.960234723	0.050051
P11-335G20	0.717428971	0.5416626	2.990802178	0.0034726	1.932517641	0.069695
P11-346M5	2.386999177	0.1238149	3.293264456	0.0256917	2.211954187	0.154758
P11-370B11	-2.462994534	0.1095173	-3.420251966	0.0262591	-2.600526293	0.09108
RP11-458I7.	0.357736028	0.7648279	2.331758988	0.0216001	1.000124554	0.364731
P11-466F5.	0.933085459	0.3768832	2.368822993	0.0113903	1.911961017	0.047142
P11-466P24	0.308243956	0.9460123	-18.43169102	7.09E-05	1.040608598	0.818705
P11-475E11	1.139868577	0.1201862	1.72678774	0.0128007	0.598000608	0.432538
P11-495P10	-1.721811885	0.2210958	-3.910846282	0.0097686	-1.373619019	0.302102
P11-506B6.	1.819655357	0.1050302	2.640744616	0.0133558	2.115161978	0.053643
P11-529A4.	0.694208786	0.6087269	2.635569377	0.0277521	0.894579211	0.496545
P11-543P15	3.405835928	0.0860557	4.187997814	0.030904	4.80418632	0.012681
P11-56B16.	0.713510615	0.2450649	1.27814246	0.0268813	0.922913938	0.121434
P11-613C6.	1.334084509	0.1148394	1.872009367	0.0195737	1.093157159	0.200475
RP11-615I2.	1.101598376	0.2884477	1.965888564	0.0436373	1.831228866	0.062836
P11-628J14	-0.385159801	0.8319587	3.309707137	0.0341006	0.931195683	0.606736
P11-655M14	3.087178455	0.0557895	4.051380462	0.0084654	3.216145577	0.043345
P11-680E19	-1.675233048	0.0669674	-2.458421986	0.0139031	-1.215722404	0.151182
P11-682B13	0.950971864	0.277061	1.861001019	0.0198566	0.451192145	0.620913
P11-697N18	0.178183787	0.8181598	1.401407425	0.0335879	1.217967921	0.07113
P11-740N7.	-1.015074996	0.2734957	-2.838804348	0.0214256	-0.926512117	0.303056
P11-820K3.	0.75307516	0.4855772	2.226838105	0.0227106	1.557272827	0.123209
P11-86K22.	0.766152958	0.5636437	2.378917802	0.0422993	2.117183363	0.07509
P11-90O23	0.34183476	0.8169025	2.80294065	0.0159253	2.366233457	0.04738
P11-927P21	0.177996856	0.8772353	-3.107103122	0.034533	-0.498099524	0.676071

P11-95M15	0.873826848	0.5886306	2.985793494	0.0367779	1.97936559	0.182424
P13-395E19	1.425514727	0.2597123	2.408775816	0.0457141	-0.141588807	0.920898
RP3-406A7.	-0.743715089	0.326668	-1.936785882	0.0260028	-1.732751381	0.046431
P3-423B22.	1.731130568	0.0635994	1.816132068	0.047366	2.162614282	0.016819
RP4-665N4.	1.033456479	0.5963496	3.735601936	0.0304771	0.428400206	0.827071
P5-875O13	-1.282048228	0.3510378	-3.582036886	0.0170535	-1.44780919	0.294031
RPL23AP2	2.236005494	0.2557197	5.144355871	0.0040164	3.93765434	0.030782
RPL24P7	-0.001214862	0.9981613	-1.581515174	0.0116067	-1.037909235	0.076135
RPL5P25	0.336189971	0.8492022	3.017499831	0.0472486	0.929885607	0.59768
RPL6P27	1.495896221	0.2996827	2.821269685	0.0466909	1.202311789	0.405794
RPL7AP4	1.045436512	0.5366216	3.027466882	0.0364643	2.909474197	0.046267
RPL7P49	0.299293445	0.7953867	1.933477134	0.0478128	1.162156452	0.261792
RPL7P50	0.336187235	0.8404472	3.093724243	0.0272549	1.91880846	0.202679
RPS16P5	1.507559478	0.0909677	2.017682126	0.0182726	2.071733867	0.015589
RPS24P12	-1.331315787	0.377259	-2.98891227	0.0496129	0.542710186	0.661768
RPS24P6	0.342360497	0.8218235	2.822680851	0.020399	2.901532137	0.017219
RPS26P21	0.336177841	0.8433837	3.107341323	0.0307236	3.090870424	0.032346
RRAD	1.625814203	0.0852948	2.330411163	0.012938	1.911098854	0.042406
RTBDN	1.623784232	0.1133659	2.242387096	0.0192888	1.428694917	0.154113
SLC6A17	0.514724746	0.5983888	2.035667713	0.0226259	1.098076669	0.233863
SNRPEP10	1.673445458	0.2042965	2.721165825	0.0259586	3.867558181	0.001102
SP140	-0.112279119	0.9183355	1.959538386	0.0493647	0.062017983	0.953129
SP7	1.276836931	0.3021571	2.538114681	0.0245468	-0.584250766	0.689181
SPATA32	-0.932873385	0.2346393	-1.734024059	0.0431176	-0.713911738	0.347141
SSX3	0.171810825	0.9267361	3.309940185	0.0408333	0.034279088	0.985363
TBC1D27	-0.248293255	0.7799835	1.644591655	0.0236434	1.132199314	0.127517
TBX1	-0.986968084	0.3348135	-2.633155749	0.0439817	-0.629610877	0.513293
TLE1P1	1.470406679	0.0542909	2.213513262	0.0025976	0.581983239	0.468298
TMEM121	0.674451311	0.5972977	2.683196763	0.0206496	1.024387746	0.404339
TPM4P1	-0.136466103	0.903135	2.095061807	0.0342848	-0.090417629	0.934673
TSHR	1.012732144	0.3294845	2.399029912	0.0146079	-0.074447217	0.945788
USP41	-1.393356005	0.3635357	-3.143285303	0.0458236	-1.497809046	0.335596
VIP	1.739272809	0.4259831	4.633148274	0.0188254	2.926807495	0.153366
VN1R107P	-0.616082861	0.6433529	-2.874066798	0.0481758	1.128579496	0.315508
WDR64	-0.483611235	0.8000477	3.554240836	0.0372232	-0.156685957	0.934337
WDR87	-0.257685663	0.8820895	3.463379154	0.0260062	0.779829652	0.629309
WFIKKN1	-0.859850354	0.1553944	-1.531066109	0.0168992	-0.667730621	0.250009
ZDHHC22	1.612796472	0.1977884	3.738356139	0.0013965	2.350959469	0.053006
C16orf89	-0.244490956	0.5532209	0.41443343	0.2906965	1.997127113	7.91E-08
DUSP7	0.013096134	0.9359737	-0.300915314	0.0660248	-0.885614077	2.29E-07
LCN15	-1.41354401	0.0928108	-1.207022354	0.1510016	-4.375518998	2.84E-07
RHOA	-0.084364877	0.1333703	0.084126751	0.1326874	0.286695049	2.87E-07
SWAP70	0.117080846	0.3458339	-0.01354905	0.9125197	0.622841085	3.19E-07
EGLN3	0.306124832	0.2096765	0.275052402	0.2594498	1.2415321	3.49E-07
FAM175A	-0.044528087	0.6536703	0.142522275	0.1437082	-0.496797836	7.57E-07
ANXA7	0.204473343	0.0637439	0.211764145	0.0540132	0.53807366	9.06E-07
ADAM9	0.268732038	0.0552113	0.238626162	0.0885532	0.673417905	1.52E-06

ABI1	0.084808186	0.3042083	0.12757409	0.1189359	0.38898089	1.82E-06
STK38L	0.166216988	0.2245442	0.165466384	0.2263669	0.648354136	2.03E-06
FABP6	-0.093083897	0.9390754	-0.38287488	0.7532187	-5.948403951	3.00E-06
GABRA2	-0.217081402	0.5455413	0.652964829	0.067224	-1.698709674	3.36E-06
SFRP4	1.784727685	0.2308197	1.987310254	0.1764568	6.636565783	3.37E-06
NDNF	-0.137745118	0.7356327	-0.017697302	0.9653447	-1.983661429	3.62E-06
EIF2S2	0.019773077	0.8366212	0.1858043	0.0518675	0.435419577	5.09E-06
LRRC8B	0.239796509	0.0679332	0.230384562	0.0783877	0.594836919	5.13E-06
PHYHIPL	-0.576845367	0.3153441	-0.587994072	0.3066879	-2.866981128	6.29E-06
ROS1	0.989516027	0.0607773	0.356177621	0.5038821	2.28598854	6.66E-06
UBE2J1	0.122684781	0.1698656	0.141244819	0.1127151	0.399923595	6.80E-06
MPHOSPH8	-0.256139606	0.071945	-0.245905263	0.0834704	-0.637329834	8.01E-06
INPP5D	-0.505425996	0.1162677	-0.470022823	0.1425695	-1.446540209	9.28E-06
SLC10A2	-0.215646347	0.8298934	0.25672671	0.7977772	-4.823431233	1.09E-05
LYPD6	0.38255062	0.051908	0.260622265	0.1828669	0.842077714	1.35E-05
PTCHD4	-0.346357351	0.3193183	-0.462839215	0.182282	-1.531790726	1.73E-05
NIPA2	0.090392253	0.3036654	0.134109509	0.124382	0.373134769	1.80E-05
SYTL2	0.334790609	0.1325021	0.072361921	0.7451131	0.951559092	1.86E-05
POLRMT	-0.296924341	0.0590171	-0.266265497	0.0879432	-0.668994497	2.18E-05
STS	0.218957387	0.200556	-0.022388115	0.895877	0.716866445	2.33E-05
ZBTB3	-0.156115844	0.3991617	-0.220358509	0.2338616	-0.799373632	2.41E-05
RBM3	-0.007784071	0.9055673	0.120514944	0.064729	0.268732351	3.77E-05
HAND2	-0.211334495	0.4520024	0.18960801	0.4965335	-1.167926097	3.98E-05
DPP4	-0.058670166	0.9039466	0.420351292	0.3870956	-1.998111675	4.11E-05
CASP4	0.289018893	0.1002718	0.11969847	0.4957583	0.710865807	4.89E-05
UBXN10	-0.62553122	0.0643518	-0.423988338	0.1962483	-1.463779401	5.03E-05
SUSD2	0.17998885	0.7123642	0.264491389	0.5865863	-2.120479852	5.07E-05
DET1	-0.183072805	0.2187017	0.094030908	0.5141354	-0.609153448	5.78E-05
ARPC5	0.108624491	0.2806246	0.188589824	0.0601253	0.395937711	7.88E-05
KCTD21	-0.288896616	0.0991027	-0.215935262	0.2159248	-0.689498089	8.97E-05
TTR	0.204393932	0.8003131	1.308628345	0.10508	-3.189966444	9.40E-05
STAG3L3	-0.207241596	0.1045958	0.005703427	0.9636381	-0.502652942	9.50E-05
PELI2	0.315736664	0.1120538	0.369293529	0.0628212	0.768269727	0.000105
RAB27A	0.422132861	0.0543229	0.152323149	0.4877294	0.844177968	0.000115
PNPLA8	0.261744861	0.1005573	0.27684415	0.0817032	0.611616021	0.000117
NTPCR	-0.104553889	0.3312154	-0.18127778	0.0886504	-0.417538344	0.000117
CD47	0.16931897	0.3075534	0.266627896	0.1076008	0.637356022	0.000118
RBM5	-0.158490842	0.0869717	-0.088469348	0.3390073	-0.356691669	0.00012
SLC19A2	0.11804432	0.2987923	0.174733243	0.1214591	0.431539243	0.000125
MUC17	-0.546546093	0.4715761	-0.224663367	0.7667952	-2.925961714	0.00013
PPIA	0.14060704	0.1339894	0.142923076	0.1275167	0.356930617	0.000141
TNRC6A	-0.268919471	0.1038385	-0.254228276	0.1235949	-0.629060308	0.000143
ARNTL2	0.317491608	0.1005359	0.17148222	0.3745835	0.730853528	0.000144
BRD8	-0.297414172	0.0537304	-0.15148644	0.3239016	-0.58641929	0.000144
C4orf19	0.349081005	0.0590875	0.167458682	0.3650201	0.698083313	0.000148
RBM4B	-0.174545934	0.2350964	-0.280197	0.0561206	-0.558793702	0.000162
F10	0.027052076	0.9418272	0.195895122	0.594549	-1.460240786	0.000163

LPIN3	-0.254059515	0.2323563	-0.397881481	0.06116	-0.801955778	0.000167
APOA2	-0.370827747	0.6676906	0.404145912	0.6394407	-3.288444518	0.000168
AMIGO2	0.266261925	0.3564345	0.091249563	0.7518841	1.07649622	0.000177
ZNF503	-0.359899858	0.1712253	-0.255332574	0.3284238	-0.997373706	0.000181
PRRG4	0.112327054	0.456707	0.156304314	0.2940841	0.550308117	0.000187
ATG4A	0.076240954	0.6878104	0.364413065	0.0512791	0.695328461	0.000191
CTB-63M22.	5.523524305	0.2636327	0.740623488	0.8810218	-18.81000863	0.000193
TACSTD2	0.129374577	0.5410676	-0.143633966	0.4975249	0.784240745	0.000197
DNAJB12	0.10956072	0.1659698	0.130900058	0.0938252	0.289470678	0.000198
SEMA3C	0.377635152	0.076941	0.216725256	0.30999	0.793529396	0.000198
DCTN2	0.10230901	0.2436675	0.168880878	0.0529864	0.323575527	0.000203
SH3TC2	-0.447592195	0.2500581	-0.618617756	0.1108387	-1.466660659	0.000207
C17orf58	0.101532111	0.5565673	0.250213933	0.1381347	0.613656441	0.00021
FSD1L	-0.306083238	0.0707168	-0.25145873	0.1282208	-0.620707818	0.000215
PRKAA1	0.108388039	0.243158	0.126707142	0.1714504	0.342145008	0.000218
C11orf84	-0.067121935	0.5700438	-0.192284747	0.1017934	-0.438489504	0.00022
GNAI1	-0.217762555	0.1040521	-0.113045937	0.3963033	-0.494307051	0.000231
ZNF70	0.013080356	0.931674	-0.059945707	0.6925821	-0.57283202	0.000233
HIATL1	0.105422534	0.2407367	0.099113303	0.2674955	0.3277513	0.000234
TET1	-0.166079919	0.2098602	-0.051085046	0.6981186	-0.485980832	0.000255
SLC9A3	-0.089359458	0.772102	-0.087370404	0.7739929	-1.156078565	0.000265
TMBIM1	0.224920743	0.1218918	0.220749889	0.1287718	0.527244188	0.000284
GPRC5B	0.202920793	0.3612854	0.107868293	0.6270777	0.797699753	0.00031
QPRT	-0.153020079	0.1282529	-0.035385924	0.7238073	-0.363037379	0.000311
MALRD1	-0.499322941	0.2534348	0.301428756	0.4858546	-1.623152026	0.000321
PRSS3P1	0.765559395	0.1019695	-0.008170008	0.9860796	1.682443175	0.000324
AC027612.6	-0.356813938	0.2202543	-0.391548057	0.176383	-1.059993854	0.000329
RPS6KA3	0.124151145	0.2030293	-0.032413116	0.7394988	0.349485616	0.000329
UBE4B	-0.0897304	0.4782726	-0.112137484	0.3744358	-0.455119955	0.000337
HOXB8	-0.615331194	0.2277794	-0.646098211	0.2050104	-2.018353635	0.000343
SLC26A11	-0.075912561	0.6912133	0.147750461	0.4323658	-0.706974803	0.000344
DDX5	0.108322526	0.2456765	0.180486248	0.0529697	0.333349501	0.000351
EFCAB3	0.356308136	0.4941634	0.865719056	0.0720899	1.632993181	0.000388
CA12	-0.360944682	0.5329452	-0.064280082	0.9115012	-2.062035124	0.000395
SLC16A7	0.056981742	0.8309374	0.481031483	0.0685076	0.926110936	0.000455
IL10RB	0.3239889	0.0723523	0.146219694	0.4172726	0.627381578	0.00047
PGM2L1	0.232229599	0.2745785	0.140510633	0.508135	0.740117424	0.000473
GUSBP2	-0.447978951	0.0827923	-0.379357097	0.135763	-0.900921538	0.000554
CAPZA1	0.073308754	0.2160915	0.074584896	0.2065991	0.203564206	0.00056
MYO1D	0.158044883	0.2511798	0.185216473	0.1781046	0.473010138	0.000578
USP16	0.103681765	0.2636294	0.164734444	0.0742045	0.317315432	0.00058
MUC5B	0.936402939	0.1046015	0.889819463	0.1229679	1.983244946	0.000582
GTDC1	0.16732578	0.2298915	0.22201187	0.1074336	0.472897817	0.000593
PRSS1	0.726616367	0.1700187	-0.080216799	0.879616	1.817805214	0.000596
TMPRSS15	0.232429579	0.8430003	0.543657187	0.6357163	3.753154233	0.000599
PPAP2A	0.281534264	0.2187723	0.326631086	0.1506046	0.77415449	0.000613
EHMT2	-0.213010696	0.1440207	-0.201465679	0.1650958	-0.499650473	0.00062

TIMM23B	0.064585573	0.412673	0.142234673	0.0684691	0.266267513	0.000644
TC2N	0.332043225	0.1774801	0.472927817	0.054686	0.836740239	0.000672
ORM1	0.135053072	0.7299208	0.556063433	0.1540646	1.322952537	0.000681
RAC3	-0.245800345	0.2036734	-0.167088767	0.381975	-0.665683428	0.000698
STEAP1	0.273091364	0.1731954	0.019972667	0.9206645	0.671765122	0.000716
C21orf33	-0.88518413	0.1705832	-1.121316671	0.0833054	-2.321493425	0.000723
ZMAT3	-0.065960235	0.6682243	-0.182474152	0.2351271	-0.520806783	0.000727
RAB11A	0.103696309	0.2857893	-0.081000134	0.404213	0.327547089	0.00073
P11-155G14	0.066944637	0.8650316	0.35937541	0.3440216	1.235523915	0.000737
ASIC4	-0.725940873	0.1803039	-0.5150455	0.2957251	-2.397816333	0.000742
PI4K2B	0.025828081	0.845656	0.179531154	0.1718765	0.440875127	0.000745
GNG3	0.419744311	0.4590019	-0.124259095	0.8297881	-2.992726928	0.000758
LAMA1	-0.578426272	0.0638811	-0.157329976	0.613455	-1.054204668	0.000761
SBF2	-0.182343036	0.4058816	-0.129415811	0.5539007	-0.737420212	0.000785
MGST3	0.024398076	0.8159817	0.024431267	0.8149969	0.349084941	0.000798
UGT2B11	-0.787118273	0.1029629	0.149274762	0.7528225	-1.647985074	0.000803
C2CD2L	-0.267413414	0.2421355	-0.143348291	0.5263506	-0.77279846	0.000806
COL6A6	-0.566253792	0.2815601	0.360329755	0.4901744	-1.781228813	0.00081
ARG1	-0.20928721	0.7008264	-0.479568821	0.3817216	-2.106991438	0.000816
EFCAB14	0.131156802	0.3485073	0.031944706	0.8194161	0.46754298	0.000822
TSPAN3	0.026776935	0.7940197	0.04108708	0.6885261	0.342703131	0.000824
HNRNPH2	-0.023056097	0.7686634	0.141370948	0.0687933	0.259607976	0.000828
HEATR5A	0.246237557	0.1547976	0.328992341	0.0564925	0.575557482	0.000835
PIP5K1B	0.277277546	0.4187284	0.572169433	0.0933204	1.132566546	0.00086
MFGE8	0.061546138	0.6360779	-0.133232377	0.3053507	-0.435464355	0.000865
SEPW1	-0.131764285	0.1744343	-0.172542839	0.0742407	-0.323396788	0.000866
PPIC	0.196433445	0.1918931	0.039961665	0.7903099	0.497281982	0.000871
SRP54	0.092130047	0.3039278	0.09009802	0.3121317	0.29594135	0.000877
VTI1A	0.166558868	0.246469	0.274529449	0.0547952	0.47528517	0.000886
DNM3	0.599022491	0.1328601	0.435253929	0.2738205	1.311764005	0.000912
AMFR	-0.02904637	0.7323199	0.022706232	0.7876698	0.27875703	0.000927
GABRA4	-0.737562223	0.3959969	-0.940743981	0.2631365	-2.996054291	0.000934
COX6A1	0.091025873	0.3273804	0.037051304	0.6895401	0.30617357	0.000944
EDA2R	-0.106488696	0.6413978	-0.323613425	0.1567882	-0.766011826	0.000945
TMPRSS3	0.319412071	0.2004492	0.023254244	0.9258764	0.816885498	0.000947
SYNE1	0.018538191	0.9061992	0.009821649	0.9501542	-0.520999968	0.00096
GALNT6	0.491217998	0.1121661	0.278946344	0.3667944	1.018511174	0.000965
FTSJ1	0.141161261	0.2127359	0.085997794	0.4444029	0.369576901	0.000974
TM9SF3	0.021743409	0.8050633	0.043855654	0.6182875	0.289669751	0.000994
ZNF445	-0.0574386	0.6359907	-0.140255024	0.2445065	-0.401839548	0.001018
SH3RF1	0.220818277	0.1321344	-0.015967379	0.9132592	0.479696229	0.001034
CMPK1	-0.005437357	0.9678888	0.071753424	0.5949014	0.442113755	0.001042
ZNF780B	-0.427027092	0.0512802	-0.38337056	0.0779027	-0.718226988	0.001047
SPAG1	0.309884934	0.3138927	0.474499794	0.1213703	1.001477048	0.001059
WT1	-0.592863027	0.3905936	-0.025456702	0.9699355	-2.381238633	0.00106
PPP1R1B	-0.467670775	0.4148795	-0.402792446	0.4809498	-1.932548304	0.001062
CHMP3	-0.018060026	0.8646711	0.174107558	0.0989982	0.344868995	0.001071

CYSTM1	0.344260238	0.1219781	0.115904226	0.6026288	0.727750263	0.001071
NAPG	-0.00678791	0.945927	0.119323526	0.2307051	0.324812556	0.001077
TMEM133	0.143590882	0.3199619	0.084034064	0.5579203	0.461700779	0.00116
SLC51A	-0.362487318	0.4308329	-0.080915821	0.86022	-1.502407111	0.001162
P11-400G3	0.317001773	0.496063	0.641499457	0.145246	1.362995432	0.001196
SGMS1	0.30071908	0.069512	0.303491395	0.0662724	0.533655509	0.001197
GFM1	-0.052855749	0.5405567	0.119085516	0.1626646	0.275962092	0.001198
CXXC4	-0.350240644	0.2729308	0.006772168	0.982969	-1.04455813	0.001203
PPM1B	0.109201945	0.298881	0.099561736	0.3416264	0.337988932	0.001214
CD58	0.05420649	0.7504615	0.161990665	0.3354724	0.540648077	0.001222
CCND2	0.007992431	0.9478941	-0.144664789	0.2367815	-0.395020075	0.001246
TMEM54	-0.03884943	0.8298321	-0.299622106	0.0980355	0.572740539	0.001252
AIF1	-0.577166001	0.1444294	0.032740459	0.9290765	-1.360590382	0.001254
TOMM40L	-0.173731802	0.2962214	-0.05169558	0.7536027	-0.539976891	0.001276
COX7A2	0.067271396	0.4233474	0.049787712	0.5514854	0.268673173	0.001279
PKP4	0.11928146	0.2825957	0.128498329	0.245544	0.355271218	0.001303
MPDU1	0.129830338	0.1922946	0.139392596	0.1591172	0.317090225	0.001336
CSTA	1.111640148	0.3901479	0.185308667	0.8910447	3.870591135	0.001339
MICAL2	0.388466318	0.0743837	0.387447622	0.0750371	0.697517486	0.001341
RNY4P13	-0.236658033	0.4491386	0.504222127	0.0813893	0.91202262	0.001344
SMAGP	-0.161453292	0.3400778	0.042343681	0.8007732	0.533983896	0.00139
MFSD6	0.006633771	0.9683977	0.047346963	0.7767594	0.532142193	0.001392
CBL	0.010189801	0.9338491	-0.174395951	0.155009	-0.394015033	0.001413
ASGR2	0.505696947	0.5738869	0.773877943	0.3869185	-3.648180875	0.001431
MORF4L2	0.050550196	0.5219979	0.020124408	0.7986346	0.250681162	0.001478
LRRTM2	0.615716582	0.0836036	0.447559716	0.2071667	1.124709741	0.001497
GNE	0.15986585	0.411265	-0.04080137	0.8337878	0.615059938	0.001501
OMD	0.979170869	0.2817994	0.60263083	0.5088661	2.826221622	0.001513
TSPAN1	0.29816023	0.1409215	-0.049663448	0.8063263	0.641807862	0.001513
GLRX3	0.079585918	0.4312501	0.09677367	0.335714	0.318348498	0.001524
ANPEP	0.430288966	0.6353882	1.185428922	0.1913821	-2.88375808	0.001533
SURF6	-0.17860321	0.2302746	-0.21630104	0.143186	-0.470332383	0.001581
ZFP82	-0.292968283	0.1134478	-0.235263931	0.1987437	-0.587245305	0.001587
ITIH2	0.443238663	0.0884751	0.120738474	0.6426404	0.821430502	0.00159
CLTA	0.102341939	0.139919	0.10808771	0.1163603	0.217019909	0.001618
PPP6R3	0.12385292	0.1108647	0.112342327	0.1472492	0.244177454	0.001629
ATP6V1G1	0.050435347	0.560413	0.08738275	0.3110586	0.27139357	0.001634
P11-347C12	-0.218744669	0.5265297	-0.303443239	0.3720626	-1.163452779	0.001634
SLC35A2	0.220834646	0.1252277	0.068152535	0.6358039	0.450661993	0.001644
GLCE	0.149298786	0.1924931	0.141110829	0.2162015	0.358221967	0.001668
ZNF562	-0.039542883	0.8099506	0.036652293	0.8217972	-0.516345841	0.001677
ANXA13	-0.053833206	0.925363	0.504729593	0.3790834	-1.817107522	0.001691
DPEP1	0.076079836	0.9202243	0.457622445	0.5393587	-3.185154293	0.00169
C5orf15	0.048499134	0.6975003	0.160997775	0.1953858	0.390056964	0.001705
BLVRB	0.0668546	0.666134	-0.158804275	0.3054704	0.48321981	0.001709
SLC44A4	0.253896174	0.0922537	-0.016579124	0.9124875	0.471525591	0.001756
PLEKHG2	0.045421512	0.8018726	-0.102172321	0.5721355	-0.566397376	0.001794

PPP1R16A	-0.043942358	0.7659815	-0.072992738	0.6210278	-0.461833947	0.001818
GBP3	0.330983544	0.0670376	0.260076447	0.1495914	0.562386335	0.00182
CTTN	0.081531835	0.1943019	-0.088872861	0.1567411	0.194797172	0.001854
KCNJ16	0.589465886	0.0697134	0.240365069	0.4601124	1.009938703	0.001859
SREBF1	0.113709171	0.380904	0.183352392	0.1562704	0.401723619	0.001867
TAGLN2	0.216152364	0.1426422	-0.199668092	0.1758506	0.458150048	0.001872
S100P	0.460900369	0.1585135	-0.240350358	0.4623545	1.010403838	0.001977
FOXN2	0.12457352	0.3718005	0.08116724	0.5597476	0.429124241	0.001997
LRRC19	0.119617487	0.803504	0.313984196	0.5127463	-1.497859215	0.001998
CYBB	0.35916429	0.315735	0.22977469	0.5186047	1.074649198	0.002006
RNF103	0.193388546	0.2318658	0.200088158	0.2153988	0.498009362	0.002012
HOXB6	-0.423300184	0.2217104	-0.176173005	0.6079648	-1.086009818	0.002024
SLC26A2	-0.148718823	0.5588597	0.076956289	0.7618038	-0.788050437	0.002026
GLRX	-0.014097974	0.9343037	-0.047765128	0.7783314	-0.53558968	0.002032
ATF7IP	-0.144382773	0.1982263	0.019938402	0.8586443	-0.346238043	0.00204
MAP7D2	0.552238283	0.1328045	0.22408886	0.5429753	1.109175588	0.002039
RNF144B	-0.057414108	0.8908037	0.074940116	0.8571263	1.274199826	0.002037
ATP11B	-0.082333373	0.4013905	-0.114821506	0.2417292	0.301442153	0.002075
POMK	-0.297885044	0.0821274	-0.30293065	0.075053	-0.52860238	0.002098
PCDHA9	-0.283161447	0.2300568	-0.20138994	0.3843194	-0.742521897	0.002099
C1orf115	-0.414001931	0.3201817	0.057731448	0.8893439	-1.288709814	0.002103
GPR111	0.346999022	0.4221473	0.317117578	0.4590852	1.297913042	0.002103
CFH	0.386945597	0.1062234	0.125744693	0.599615	0.735005803	0.002138
C14orf105	-0.336635249	0.2227903	-0.33859527	0.219233	-0.849096333	0.002144
ANXA3	0.133431653	0.2701185	0.035562427	0.7685486	0.368692276	0.002237
KLHL31	-0.560460627	0.0993816	-0.535334688	0.1095278	-1.068975422	0.002237
CLCN2	0.050735048	0.8464003	-0.063814206	0.8055566	-0.804414351	0.002286
CSNK2A1	-0.017648716	0.8710001	-0.183778626	0.0906372	-0.331683972	0.002288
NAGS	-0.761993091	0.0652536	-0.027590202	0.9436689	-1.302579083	0.002318
IL37	0.261273086	0.7726016	0.042395646	0.9626312	-3.460635901	0.002325
NLGN1	0.317881545	0.2577708	0.405733105	0.148393	0.851018973	0.002347
KLHL20	-0.153933904	0.1995853	-0.220691857	0.0646867	-0.365587521	0.002367
TMBIM4	0.092404623	0.3965441	0.071597445	0.5096324	0.329339472	0.002371
PLOD1	0.111893838	0.401698	-0.186786863	0.1616254	-0.405696834	0.002388
DNAH5	0.242498321	0.3584151	0.282749089	0.2850027	0.793795426	0.002399
MSL2	-0.004347141	0.9692674	-0.151056463	0.1795174	-0.34225114	0.002396
ZFHX2	-0.305627105	0.5909367	-0.720204565	0.2030564	-1.740213617	0.002399
ALDOB	-0.121923463	0.892075	1.054557956	0.2403316	-2.736918841	0.002429
SLMO2	-0.004773363	0.9639462	0.055624083	0.5970213	0.318213645	0.002458
MMP10	0.40511013	0.5374177	0.588029508	0.3698921	1.979551554	0.002467
CDX2	-0.586958213	0.1207636	-0.711812002	0.0595277	-1.147209663	0.002473
ITSN2	0.26214168	0.1733106	0.200660304	0.2963347	0.580327077	0.002474
MAU2	-0.134456113	0.2288564	-0.181128074	0.1034298	-0.337249003	0.002472
SRI	0.03469051	0.7066979	0.085005517	0.3541257	0.27721592	0.002475
ZNF462	-0.180564995	0.3177395	-0.069222386	0.7011922	-0.547154187	0.002507
ZNF891	-0.258881106	0.0966764	-0.226620584	0.1424039	-0.472204475	0.002509
SYDE2	-0.02420025	0.8750994	0.076308149	0.6181421	-0.467454012	0.002548

DAD1	0.100881426	0.2061282	0.145188349	0.0670354	0.239200077	0.002562
MASTL	0.111021362	0.4006537	0.15715561	0.2318083	0.395798731	0.002562
ZNF148	-0.07816002	0.3007259	-0.112469945	0.1346775	-0.227111987	0.002602
PARM1	-0.344008346	0.2262979	0.049530009	0.8616261	-0.855203766	0.002642
LRRC39	-0.304464844	0.7054441	0.187459543	0.8135826	-2.701577087	0.002664
CHRNA5	-0.430886506	0.1315371	0.333135122	0.2333681	-0.859873092	0.002699
MPZL2	0.120742181	0.4175249	-0.057025947	0.7016084	0.445252199	0.002726
P11-474D14	-0.716619764	0.1280815	0.054585973	0.8967257	-1.5898124	0.002735
ZNF219	-0.261600956	0.2536001	-0.180927429	0.4268718	-0.690196153	0.002736
AP000347.2	0.047376125	0.7928878	-0.065785605	0.7135704	-0.547376715	0.00274
GABRE	-0.393553595	0.3160568	-0.375912958	0.3371046	-1.184261103	0.002784
SCGB1A1	-1.707282547	0.3018517	1.110457843	0.4167965	3.968568638	0.002801
GSDMB	0.159892587	0.4417041	-0.205098252	0.3238029	0.616501937	0.002816
PIK3R2	-0.219768972	0.1639027	-0.087191221	0.5782613	-0.472466838	0.002827
PTPRD	0.349280105	0.1442603	0.280886332	0.2400747	0.71196142	0.002869
MXD4	-0.035416442	0.7793054	-0.22802876	0.0715459	-0.378546983	0.002885
SPATA17	-0.485256598	0.0922411	-0.306438099	0.278707	-0.878832932	0.002918
ZNF334	-0.232620386	0.3379461	-0.318986529	0.187335	-0.728400993	0.002933
ATHL1	-0.078004826	0.6750752	-0.238706395	0.1998934	-0.556482158	0.002938
DIABLO	0.023076851	0.7794104	0.141589008	0.0812335	0.241270755	0.002943
MCFD2	-0.135591572	0.1222154	-0.087001802	0.3200393	-0.26080144	0.002949
SYT14	1.359015575	0.0704376	1.190012372	0.1102484	2.171775168	0.00298
KIF27	-0.217789403	0.1856072	-0.137858015	0.390605	-0.48828488	0.002983
ZKSCAN3	-0.17245489	0.2781942	0.124395937	0.4207473	-0.475802953	0.002995
RAB8B	0.085662483	0.6003698	0.26708138	0.1021778	0.4842687	0.00302
GLRX5	-0.088476308	0.2903785	0.08790512	0.2853293	0.243285244	0.003046
ZNF408	0.020671931	0.9176184	-0.288430689	0.1517215	-0.606433296	0.003062
SAT2	-0.267464497	0.0655294	-0.26192105	0.0690032	-0.430010243	0.00307
APOM	0.012282884	0.97906	0.164416568	0.7247765	-1.399408322	0.003129
NUDT14	-0.058543217	0.7002604	-0.08276504	0.5786211	-0.459702982	0.003145
ADH1B	1.581385769	0.1483786	1.992402981	0.0570399	3.03937745	0.00316
AVPI1	0.052353262	0.8538502	-0.359781367	0.207544	-0.859205935	0.00316
REPS1	-0.162895176	0.157673	-0.123745409	0.281412	-0.339792801	0.003204
ZNF784	0.107351957	0.5359903	-0.032490712	0.8501719	-0.535616473	0.003209
CHMP2B	0.125678293	0.3081964	0.077754483	0.5276434	0.361890571	0.003241
SQRDL	0.056210791	0.7224117	0.063447697	0.6874541	0.462296813	0.003277
ZSCAN12P1	-0.374095048	0.2758211	0.050631678	0.8782912	-1.050985225	0.003314
TM9SF2	0.100717446	0.3153877	0.132481355	0.1862135	0.293589067	0.003379
GNL2	0.082395912	0.5562558	0.226893878	0.1047528	0.409592939	0.003386
RELL1	0.223388267	0.1320726	0.004896029	0.9736561	0.430634476	0.003438
C19orf69	0.119200054	0.894848	0.524237569	0.5555844	-3.521557973	0.003446
WNK4	0.527506545	0.2591342	-0.011192453	0.9809385	-1.422787102	0.003499
EPT1	0.187907277	0.0799199	0.097910922	0.3602799	0.312110548	0.003519
ORAI3	-0.039894016	0.7881548	-0.26785933	0.0723398	-0.438163215	0.003519
SIRT4	-0.494799457	0.2208072	-0.269700031	0.4728848	-1.233915017	0.003518
CD82	0.397242372	0.1422552	0.355148938	0.1869189	0.78245362	0.003527
GJA3	1.579533422	0.0692455	0.152782281	0.86923	2.462724597	0.003561

NDST4	0.615144299	0.724436	1.755254535	0.2949789	4.708266854	0.003559
UBE2N	0.04655448	0.5897247	0.137649521	0.1090664	0.250629629	0.003563
PITPNB	0.313691791	0.063423	0.198373483	0.2398251	0.490842414	0.00357
KIF28P	-0.269495678	0.613696	-0.282883936	0.5869259	-1.601759367	0.003577
3HD14A-AC	-0.380583808	0.5750953	-0.463538489	0.4903785	-2.458147161	0.003587
SENP7	-0.123203814	0.3614337	0.012560447	0.9255836	-0.393589929	0.003597
EDRF1	-0.267305551	0.05244	-0.165069661	0.2265617	-0.399745639	0.003611
C16orf70	-0.059554377	0.5902492	-0.180819488	0.0980536	-0.325165226	0.003649
NPRL3	-0.554669186	0.0911895	0.158108438	0.6195408	-1.010625486	0.003676
SPINK1	0.418769312	0.0669615	-0.274892135	0.2291849	0.662975081	0.003726
PPTC7	0.203696747	0.1579421	0.122076046	0.3967087	0.415829698	0.003743
CDR1	0.647620736	0.1236497	-0.562258359	0.1937808	-1.306703924	0.003801
C19orf43	0.189315226	0.0657873	0.125811904	0.2201198	0.296545006	0.003813
AFF1	0.131845393	0.4100326	-0.179581685	0.2618687	-0.462910428	0.003826
LRRC59	0.222579358	0.0506075	0.128488712	0.2587359	0.328233324	0.003894
KRBA2	-0.258258273	0.2183449	-0.115990694	0.5693094	-0.615350616	0.003914
ABHD16B	0.462365662	0.068912	0.371026152	0.1398367	0.712354316	0.003927
TENM2	0.769573806	0.2855231	1.025496306	0.1544025	2.069557524	0.003947
ANKRD22	0.259602003	0.2309452	0.203584266	0.3472084	0.62155399	0.003969
TTC5	-0.159958734	0.3272257	-0.264726942	0.1034679	-0.469371841	0.00398
BCL2L1	-0.032561374	0.8411432	-0.317475054	0.050705	-0.46833578	0.003983
P11-254B13	1.005991632	0.1387541	0.756422593	0.2662439	1.831827821	0.004
CADPS2	0.238145223	0.2907572	0.358950843	0.1099067	0.64429355	0.004038
DCAF15	-0.09938946	0.5212107	-0.173053935	0.2612818	-0.445522909	0.004135
TMEM150B	-0.575184731	0.337425	-0.044051674	0.9400604	-1.79753245	0.004141
YES1	-0.008602893	0.9242564	-0.021568673	0.8112309	0.258342021	0.004176
FMO1	0.637203087	0.611721	1.525296537	0.2115841	3.412261551	0.004185
CKMT1B	0.292897536	0.1704608	0.191313347	0.3699055	0.608476675	0.004207
DLD	-0.0061815	0.9387428	0.086733311	0.2774501	0.228621922	0.004203
TAF3	-0.082042798	0.5990596	-0.245011345	0.115138	-0.451764052	0.004209
RBFOX3	-0.329180147	0.4225941	-0.494877294	0.2296875	-1.235570104	0.004213
KCTD9	0.036755569	0.7082728	0.168593679	0.0839417	0.277382909	0.004254
UBXN7	0.005747617	0.9679663	-0.033169963	0.8164922	-0.40892382	0.004304
CENPJ	-0.262786606	0.0564282	-0.132575773	0.3325981	-0.392630396	0.004326
FAM118A	-0.133833707	0.304761	-0.163108069	0.2087922	-0.372091891	0.004393
PADI2	-0.679770749	0.1754021	-0.669013623	0.1812272	-1.437527169	0.004409
LGI4	-0.419053494	0.4554246	-0.353504786	0.5277652	-1.615416135	0.004416
PLS1	0.220657263	0.1301852	-0.050059559	0.7313476	0.414187302	0.004451
MTMR2	0.172946273	0.0719234	0.009085704	0.924519	0.272119314	0.004468
MLH1	-0.098106946	0.508979	0.243298106	0.099587	0.419920535	0.00451
URAD	-0.216643394	0.8384207	0.338906983	0.7475451	-3.46715304	0.004512
P11-39K24.1	1.250084799	0.1028003	1.03612719	0.1756867	2.073869026	0.00453
ARID1A	-0.136654471	0.4734612	-0.336048679	0.078129	-0.541666048	0.004536
SMIM3	-0.15780844	0.4716747	-0.212091714	0.3324101	-0.62393485	0.004596
TSC22D2	0.19316114	0.1294393	0.178912458	0.1591488	0.360109145	0.004602
KLF4	0.201302445	0.5066811	0.139589552	0.6449577	0.856032835	0.004605
RHOC	0.16104525	0.2186108	0.050863586	0.6974646	0.370081838	0.004641

VAV3	-0.815438537	0.0569761	0.287490981	0.4940354	-1.262822533	0.004655
NGF	1.554852795	0.1006067	0.844985021	0.3822312	2.594017712	0.004681
SLC7A11	0.332282078	0.1766394	0.441870945	0.0721156	0.694363354	0.004709
DHRS7	-0.017581615	0.8940798	-0.053706162	0.683216	0.37033659	0.004745
STX19	0.635218449	0.1646164	-0.309135368	0.5207076	1.242248569	0.004838
HIPK3	0.074169961	0.3639676	0.10671595	0.1903813	0.229352681	0.004872
P11-453E17	-0.433035046	0.0570767	-0.222333298	0.3059034	-0.645998465	0.004913
C8G	-0.4689138	0.5935421	0.974264989	0.2525729	-2.85323441	0.00493
XKR8	-0.201853021	0.3486836	-0.400049968	0.0615151	-0.61275373	0.004949
DDX60	0.151335987	0.4759729	0.098155748	0.6421521	0.591764901	0.004961
CCDC47	0.131437087	0.2875805	0.134911559	0.2740668	0.346058903	0.004968
EID2	-0.216834686	0.1814373	-0.238242516	0.1365868	-0.458761679	0.004998
GIPC1	0.033909145	0.7896913	0.038217404	0.7631674	0.354893768	0.005028
MOGAT3	-0.474358908	0.3061506	-0.166168345	0.7177241	-1.316723125	0.005051
MYH8	-1.062391976	0.3338987	-1.747420879	0.1155171	-3.352010784	0.005145
AMD1	0.072697372	0.5554692	0.09737397	0.429292	0.344258088	0.005179
ST14	0.227339251	0.2040147	0.273257698	0.1267259	0.499917335	0.005185
MMP14	0.330302608	0.0537385	0.322157325	0.0597334	0.478208495	0.00519
DISP1	-0.162141675	0.3682124	-0.011508922	0.9488134	-0.506972486	0.005211
ZCCHC11	-0.18655161	0.0713729	-0.050807797	0.6220053	-0.288838521	0.005212
OR13K1P	1.280098018	0.1139005	1.141942371	0.1565667	2.181320358	0.005218
DDX39A	0.28345349	0.0615423	0.146491783	0.332959	0.421498088	0.005229
NAPB	-0.234585894	0.1811184	-0.341710024	0.0503698	-0.492100983	0.00523
ZNF506	-0.024271355	0.8420869	-0.09556706	0.4294349	-0.340309185	0.005239
TINAG	-0.305905323	0.5498866	0.668204167	0.1842963	-1.492025986	0.005251
VSIG2	0.277495432	0.3661526	-0.093034534	0.7619488	0.856025613	0.005271
CLIC1	0.183928812	0.1181185	-0.027893448	0.8126529	0.328051085	0.005293
HSPD1P6	0.762737296	0.1126409	0.76739207	0.0953242	1.280634365	0.005292
PPP1R3F	0.192486855	0.5644532	-0.329641819	0.3240643	-0.9463801	0.005335
FKBP10	-0.016808203	0.9192484	0.024164801	0.8840556	-0.46207154	0.005356
ZNF528	-0.025492757	0.9363035	0.002808331	0.9930019	-0.915705631	0.005371
ACIN1	0.090478343	0.1979057	-0.099339227	0.157263	-0.195613433	0.005428
C1orf145	-0.003397763	0.9925939	-0.346183357	0.3468314	-1.045257163	0.005437
SLC23A3	-0.356990159	0.2679381	-0.205031625	0.5198099	-0.903476801	0.005465
EFR3A	-0.045227649	0.5393804	0.040797346	0.5774787	0.202880122	0.005504
MASP2	-0.214675542	0.2786067	0.00737864	0.9692447	-0.560049838	0.005518
P11-113D6.	-0.312794569	0.116411	-0.258880465	0.1820487	-0.558875571	0.005523
ZFP90	-0.275967329	0.0744202	0.016020705	0.9161032	-0.42541986	0.005572
ASPHD2	-0.057042605	0.7796151	0.231625756	0.2407279	0.541381836	0.005593
GATS	-0.302155652	0.1678372	-0.234081353	0.2816975	-0.610866584	0.005589
GUK1	0.165720079	0.1061003	0.036380218	0.7221177	0.282545585	0.005627
ZRANB2	-0.190972792	0.0645183	-0.183974831	0.073927	-0.285272857	0.005733
SLC1A1	-0.23491267	0.5507867	0.202664695	0.6053395	-1.091101135	0.005761
ZSWIM5	-0.238883831	0.2472726	-0.243846522	0.232615	-0.575042255	0.005832
LNP1	-0.427028893	0.0725144	-0.296901486	0.1989989	-0.661329407	0.005853
NRN1	-0.865757622	0.5303168	-1.22518108	0.3795976	-4.638136705	0.005881
SLCO2B1	-0.460658573	0.2526366	-0.352499608	0.3808497	-1.111722345	0.00588

APAF1	-0.007827636	0.9595283	-0.054238245	0.7246669	0.42352737	0.005889
DTWD2	0.004890134	0.9755185	0.01466755	0.925253	0.427710769	0.005887
KCTD1	-0.336658985	0.0830855	0.096082436	0.6130057	-0.536766887	0.005989
ZNF862	-0.311817416	0.2577588	-0.242227108	0.3792894	-0.763775089	0.006013
CNOT4	-0.000561624	0.9964054	-0.039053808	0.753502	-0.343586606	0.006036
MAP3K5	0.047136765	0.8806047	-0.408626426	0.1934141	0.855946958	0.006044
SPG7	-0.181532838	0.092162	-0.178681195	0.0965358	-0.295519058	0.006043
MAP3K2	0.205107702	0.0519596	-0.012177439	0.9079481	0.288745802	0.006065
MGAM	0.971860794	0.0515862	-0.732805582	0.1448321	1.367557207	0.006071
AAGAB	0.004517956	0.9690232	0.044796608	0.6989009	0.316628306	0.006088
POF1B	0.32132315	0.0557132	0.20441464	0.2234242	0.460300329	0.006102
EFCAB4B	0.359136975	0.2974947	0.38722098	0.2557997	0.924606856	0.006191
CA3	-0.216987259	0.5088857	0.014174407	0.9640533	-0.9482263	0.006212
XPNPEP2	0.594462053	0.3651639	0.311077548	0.6346346	-1.859414312	0.006217
DHRS9	-0.554566573	0.4319794	-0.20105192	0.774644	-1.948646366	0.00626
P11-139J15	0.339160697	0.6224869	-0.190876884	0.7842931	-2.323189488	0.006257
AQP11	-0.026380263	0.9338501	-0.264751567	0.401194	-0.899009171	0.0063
FASTKD2	0.064990609	0.5524928	0.165283401	0.1282644	0.296759726	0.006325
FAM69B	0.012727306	0.9344409	-0.14082397	0.361052	-0.427142831	0.006329
RGS17	-0.127162102	0.6480848	-0.393182304	0.1570297	-0.767211615	0.006363
PAPSS2	-0.252614442	0.3159785	-0.106742887	0.6708367	-0.689333625	0.006386
TYMP	0.492113409	0.0582321	0.502686917	0.050438	0.699149758	0.00641
RPN1	0.048560608	0.5926808	0.128044264	0.1579234	0.247049404	0.00642
CCNL2	-0.004139459	0.9760154	-0.257599901	0.0612543	-0.375352201	0.006433
TERF1	-0.090817106	0.4752607	-0.045396036	0.72003	-0.3476312	0.006438
RGCC	0.474218965	0.6363851	0.405524403	0.6867805	2.634830153	0.006462
FCF1P2	-0.436538692	0.0648517	-0.238908516	0.2932378	-0.648797024	0.006472
CDC73	-0.160295672	0.1434523	-0.118352867	0.2777131	-0.297939661	0.006476
WDR20	-0.105222762	0.3889483	-0.194624826	0.1109547	-0.333054843	0.006498
CSNK1A1	0.049602796	0.4629285	0.120215334	0.0745144	0.183364797	0.00651
TBC1D8	0.072476067	0.5196339	0.087572706	0.4344143	0.302242544	0.006511
ANXA5	-0.042406615	0.7262344	0.181234241	0.1340991	0.328886429	0.006549
LMO4	-0.12162629	0.2559111	-0.196035055	0.0664783	-0.29107602	0.006549
WWP2	0.098793538	0.373309	0.185180208	0.0919476	0.298697599	0.00655
SHOX2	2.476292798	0.0897801	1.495600705	0.3073521	3.948068871	0.00656
ANGPTL2	0.001165711	0.995813	-0.289214717	0.1941285	-0.607429632	0.006593
AP3S2	-0.053797824	0.7990625	-0.038311867	0.8558473	-0.575331834	0.006595
CD151	0.030912655	0.7570085	-0.103215623	0.3008893	0.270131086	0.006585
LRRFIP2	0.090999846	0.3903121	0.016898227	0.8721215	0.284331586	0.006588
TM2D2	0.17553057	0.1725713	-0.051337007	0.6891339	0.346962116	0.006626
RUFY3	0.167518663	0.1059426	0.020209827	0.8445013	0.278501262	0.006649
ZNF79	-0.322933095	0.1974313	-0.398797986	0.1097542	-0.68375837	0.006694
C12orf79	0.772018332	0.194905	-0.308025275	0.6289059	1.571938332	0.006709
FAM3B	0.051058566	0.8590906	-0.180579091	0.5299832	0.769862021	0.006744
LGALS14	0.052169712	0.9393556	0.669676367	0.3169974	-2.145322786	0.006779
TTC4P1	-1.377055696	0.0772801	-0.340513955	0.6280749	-2.364989014	0.006784
ACKR4	0.223135785	0.6549606	-0.541784764	0.2846199	1.321011047	0.006818

SEH1L	0.046119004	0.7055567	0.168668864	0.1653965	0.328661806	0.006867
TAB1	-0.137626999	0.3414439	-0.267346751	0.0629419	-0.391334428	0.006954
DBNL	0.059420458	0.5893006	0.045741752	0.6769581	0.295625634	0.00699
POLD2	-0.496720158	0.0860988	-0.503325327	0.0787553	-0.781500395	0.006981
UBE2L3	-0.052009523	0.5762251	0.131350822	0.1557651	0.249527987	0.006989
PLEKHG5	0.015220241	0.9212912	-0.021877623	0.8864938	-0.419710281	0.007
HOMER2	0.32638911	0.4540314	0.537999731	0.2147749	1.167579273	0.007009
ZNF660	-0.258846381	0.197831	0.087175168	0.6588953	-0.543184364	0.007048
P11-296I10	-0.128054344	0.4568274	-0.146111638	0.3924373	-0.467754753	0.007061
CP	0.417718696	0.0683949	0.309904137	0.1763629	0.617053053	0.007097
APOE	-0.113119658	0.6345915	0.203705305	0.3903504	-0.643865123	0.007168
CCPG1	0.008332487	0.9568606	0.056465133	0.7129601	0.411970797	0.007165
ZFP91	0.068532405	0.3086205	-0.017181236	0.7978873	0.179952086	0.007201
DPYSL2	0.136686122	0.1561941	-0.036052053	0.7082823	-0.259354762	0.00721
DNAJC10	0.013639476	0.8991076	-0.029342174	0.7845645	0.288109892	0.007268
LSAMP	0.012147079	0.9524904	0.324091075	0.1113606	-0.549472153	0.007261
ZNF155	-0.318009669	0.1138547	-0.241193219	0.2252797	-0.541050351	0.007245
ASGR1	-0.213405562	0.5711043	0.205763007	0.5787294	-1.033889833	0.007297
HSD17B12	0.233763095	0.0537329	0.149581955	0.2164968	0.324633304	0.007309
TRIM59	-0.176162291	0.1854246	-0.063059061	0.6321378	-0.356016368	0.007394
SNRPB	0.230100804	0.0807866	0.090268039	0.492959	0.351801367	0.00746
SEMA5A	-0.062655279	0.8459475	-0.202298877	0.5301404	-0.86425254	0.007509
NGFR	0.678755385	0.324695	0.051663102	0.9404065	1.824325214	0.007515
KIAA1755	0.700369998	0.2861898	0.427759599	0.5145342	1.722598605	0.007539
P11-182I10	1.399532747	0.0523084	1.16006699	0.107818	1.855589954	0.007605
SLX4	-0.153328388	0.2894506	-0.167082618	0.2452092	-0.388075006	0.007713
TFEC	0.059015773	0.9452566	1.137369099	0.1799317	-2.400362761	0.007744
FARSB	0.038109376	0.7350625	0.204955824	0.0663272	0.297095537	0.00778
GBF1	0.020731174	0.8451545	-0.130254916	0.2194596	-0.282922187	0.007813
ARRDC4	0.079919701	0.7269293	-0.324092392	0.1568033	-0.608841092	0.007845
CD63	0.174568367	0.0890106	-0.020719852	0.8400234	0.272684341	0.00786
SLC6A11	-0.151295939	0.8024523	0.651267761	0.2754813	-1.705609746	0.007897
SH3GLB2	0.039043437	0.7501692	-0.071129051	0.561795	0.324326969	0.007913
TRMT12	0.100571913	0.517952	-0.205580556	0.1836833	-0.415157767	0.007927
AC007040.1	0.40045992	0.4846432	0.905933568	0.1116759	1.499488897	0.008015
PHF23	-0.170759584	0.1880503	-0.251211598	0.052296	-0.343658247	0.008017
SLC25A13	0.119579101	0.3447755	0.153779546	0.2227975	0.334002597	0.00801
LARGE	0.113157421	0.3649358	-0.11078461	0.376216	0.329355331	0.008055
ZC3H10	-0.299006621	0.0789792	-0.185263447	0.2690471	-0.449881735	0.008076
SESN3	-0.281429989	0.2413719	0.376334535	0.1168395	-0.636259949	0.008106
AATK	-0.248276275	0.4826629	-0.45234029	0.2017603	-0.94368093	0.008178
SBDS	-0.060414398	0.5367056	0.157888171	0.1032018	0.256279698	0.008182
WDR48	0.077304173	0.4808184	0.194880383	0.0740667	0.288817727	0.008164
BCAR3	0.272312794	0.2002146	0.33580682	0.1138182	0.558481002	0.008186
FREM1	-0.177832555	0.5932632	0.164252429	0.6215755	-0.88092607	0.008218
ZNF569	-0.309550637	0.1914506	-0.092564091	0.6929561	-0.624849898	0.008287
ANKRD13C	-0.190953121	0.0917137	-0.105485236	0.3498337	-0.299091024	0.008301

ARHGEF35	-0.075959189	0.5113256	-0.162914188	0.1566469	0.300090182	0.008317
LUZP1	0.070254742	0.6060636	-0.200960848	0.1408009	-0.360116604	0.008392
GRM8	0.530883333	0.5011054	0.411072075	0.5947073	1.968518577	0.008404
NAPEPLD	-0.495301275	0.0603758	-0.45016036	0.087175	-0.697984822	0.008455
ADCY1	0.389809883	0.3592703	0.229570943	0.588728	1.108938106	0.008465
ASXL3	-0.274718692	0.350814	0.159050404	0.5844672	-0.775537009	0.008476
AGBL5	0.089743746	0.3824583	-0.089864985	0.380245	-0.271937079	0.0085
PRRX1	2.095426482	0.0501151	1.495668219	0.1624327	2.812117112	0.008512
VIT	-0.947182949	0.4299731	0.795338474	0.4746276	2.831499516	0.00852
ZNF615	-0.227273465	0.280135	-0.107447453	0.6058563	-0.553470415	0.008542
HLA-DRB1	-0.249308038	0.640661	-0.112914387	0.8324979	-1.405884413	0.00861
GIN1	-0.451977614	0.0558725	-0.244105094	0.2845974	-0.609022162	0.008685
GOLGA8J	-0.062483654	0.9128717	0.466110346	0.3909295	-1.822016212	0.008693
LRRC3	0.072465006	0.6276996	-0.186923676	0.2116778	-0.402471157	0.008694
PCED1B	0.281628914	0.4149034	0.090761642	0.7940086	0.872698609	0.008791
TSSK5P1	-0.740032285	0.1363937	-0.821267577	0.0917778	-1.410677647	0.008789
NAALADL2	0.209765235	0.3717978	0.392046627	0.0948902	0.613859703	0.008853
SLC36A1	-0.132273237	0.7401262	0.206876928	0.603554	-1.052460942	0.008856
TMEM9B	0.013221158	0.897339	-0.039861903	0.6951705	0.264236303	0.00887
KIAA0226L	0.333349791	0.6782463	-0.017886648	0.9821801	-2.362740429	0.008897
ZNF16	-0.14730004	0.2445523	-0.142718063	0.2530071	-0.331894259	0.008914
AHCYL1	0.108873178	0.3472133	0.183804177	0.112168	0.3024593	0.008925
MORN4	-0.319065982	0.1329629	-0.412755414	0.0503658	-0.565916184	0.008959
SPOPL	0.236720746	0.0890016	0.048092399	0.7295763	0.36240854	0.009031
DIAPH2	0.016015697	0.9081589	0.068550261	0.6209896	0.361360803	0.009087
TMEM246	0.119201515	0.348827	-0.016195242	0.8984775	0.329523083	0.009084
IKBIP	-0.269726183	0.070044	-0.17008319	0.2499554	-0.387674778	0.009094
AK8	0.656100907	0.1613246	0.381010036	0.4214581	1.201452221	0.009251
C11orf70	-0.791603012	0.0963117	0.042168801	0.9266838	-1.281588341	0.009247
MPP5	-0.074270586	0.3496098	-0.146501879	0.0628585	-0.205424933	0.009296
IDNK	0.034785251	0.8679818	0.027030679	0.894298	0.51383031	0.009318
SOX4	-0.167876744	0.250275	0.093059613	0.5230885	-0.379784251	0.009321
PLEKHA8P1	-0.512198838	0.0683663	-0.476483462	0.0852548	-0.736624739	0.00933
AIFM1	0.067717858	0.6189494	0.129281432	0.339185	0.351208877	0.009355
CYBA	0.229844317	0.1260229	0.074406729	0.6206514	0.389784371	0.009356
ERVMER34-	-0.029816264	0.9210731	0.313589663	0.2855844	0.765383287	0.009367
SUSD4	0.395921393	0.2173062	-0.329907781	0.3131503	0.823410095	0.009414
CHMP7	0.061507686	0.565404	-0.012759401	0.9045888	0.275661063	0.009423
GGT1	-0.065360989	0.8840013	0.503088138	0.2569156	-1.188696328	0.009423
ZNF223	-0.323177	0.0785781	-0.249664746	0.1666398	-0.480041696	0.009519
COX5B	0.143083367	0.1964564	0.022595938	0.8380102	0.285454544	0.009555
RAI14	0.203386765	0.3138546	0.117881244	0.5593425	0.522521998	0.009579
TNFRSF10C	-0.158516752	0.6081625	-0.2812778	0.362816	-0.804997806	0.009605
PDPK2	-0.260809889	0.3156997	-0.18976023	0.4588344	-0.683006952	0.009703
SYNM	0.337470862	0.1703282	0.344473793	0.1604924	-0.645122826	0.009716
RCOR2	-0.397469511	0.1168136	-0.391320373	0.118224	-0.656014464	0.009728
KDM7A	-0.03464568	0.8619028	0.091711348	0.6448454	0.512542762	0.009756

AC096921.2	-0.588429016	0.3002867	-0.897399768	0.1158391	-1.569026284	0.009799
BNIP3	-0.129113609	0.4358703	-0.309314738	0.0618432	-0.428358039	0.009805
DSTYK	-0.135757219	0.270458	-0.178656131	0.143741	-0.318551658	0.009837
HOXB3	-0.338580336	0.275005	0.124912103	0.6853972	-0.802375509	0.009873
ARL6	-0.415770611	0.0529134	-0.169675617	0.4145885	-0.547847701	0.009891
PCGF6	-0.354496493	0.0550059	-0.055670913	0.7577757	-0.470746785	0.009888
SDPR	0.032342813	0.9321873	0.222254066	0.5581112	-0.984924962	0.009922
SLC6A4	-0.522797762	0.2312196	-0.285790403	0.4969947	-1.124090222	0.009959
GSG2	-0.250960435	0.2179987	-0.220891201	0.2737699	-0.527494031	0.010007
CUEDC2	0.040511936	0.7620401	-0.100812222	0.4500548	-0.347947262	0.010024
?11-1136G1	0.244846394	0.4132174	-0.140878178	0.6428986	0.730090734	0.010152
TBL1XR1	0.095648108	0.3274408	0.163712171	0.0928447	0.250298759	0.010196
PRG4	-0.397288739	0.5840803	0.697477991	0.2801947	1.643426534	0.010212
ISX	-0.511120628	0.3131504	-0.567028754	0.2629162	-1.304331287	0.010256
UCK1	0.056123907	0.6978629	-0.114252076	0.4278067	-0.373772272	0.010356
FAN1	-0.011214503	0.9706705	0.175599282	0.5629629	-0.812765205	0.010407
DCDC2	-0.392391591	0.3536163	0.433380433	0.3017733	-1.092496052	0.010433
XPNPEP1	0.15952727	0.136756	0.104950612	0.3265437	0.273627317	0.010457
EDEM3	0.095615248	0.5131386	-0.120099656	0.4113112	0.373808134	0.010489
TMPRSS11A	-0.569628269	0.6578089	-1.518182956	0.2907329	2.847492257	0.010518
CCT5	0.09594831	0.393803	0.157009894	0.1621785	0.287260741	0.010533
TMEM198	-0.116035014	0.6217828	-0.092589536	0.6855709	-0.622924603	0.010546
KAT6B	-0.017582437	0.8620134	-0.178907866	0.0762085	-0.259478209	0.010566
USP15	0.034202525	0.804708	0.151582501	0.2721121	0.352698406	0.010574
UMPS	0.113171958	0.4838933	-0.000383218	0.9981062	0.412614052	0.010584
PKM	0.02421059	0.8058526	0.046421989	0.637381	0.251691147	0.01059
CYP24A1	-1.451898976	0.387183	1.361654668	0.3242644	3.444454346	0.010601
PSMD10	-0.078179137	0.6106236	0.245011185	0.1078715	0.389252666	0.010604
UBFD1	-0.075818116	0.4512284	-0.110413473	0.2704023	-0.257112038	0.010645
TRIM39	0.135747741	0.1863132	-0.158914401	0.1223536	0.259586985	0.010709
PHOSPHO2	-0.568823036	0.3320802	-0.341048421	0.5583085	-1.576000863	0.010761
MYL12B	-0.167734562	0.0766894	0.02528369	0.789144	0.240958257	0.010782
PXDN	-0.021739007	0.8905453	0.074536368	0.6368215	-0.403203093	0.010779
TBC1D5	-0.105189861	0.2954836	-0.118025185	0.2386768	-0.256746755	0.010808
RAB2A	-0.13651244	0.0972787	-0.05969518	0.4656808	0.208057129	0.010891
PROSER2	0.306016259	0.202582	0.175478516	0.4627826	0.604798476	0.01091
UCK2	0.011464356	0.9367933	0.161960886	0.2600973	0.365672686	0.010918
XPC	-0.185485853	0.2344654	-0.250264975	0.1070817	-0.396917681	0.010948
FADS1	0.194253013	0.1833843	0.023513933	0.8720271	0.370994871	0.010999
TVP23B	0.271101345	0.054535	0.194693692	0.166392	0.35769118	0.010988
GMFB	0.122211801	0.1460423	0.113480545	0.1755134	0.213008138	0.011029
NDUFV2P1	0.34444568	0.3083125	0.372414999	0.2636991	0.832616075	0.011065
MRPL14	0.165719705	0.0812634	-0.083433016	0.3802974	0.239434382	0.011094
NHS	0.37183228	0.238796	-0.057773033	0.8548033	0.799760526	0.011115
NPIPA1	-0.023039178	0.8743783	0.032981128	0.8207464	-0.371128508	0.011131
SP4	-0.231021775	0.1009308	-0.241896705	0.0843846	-0.357173638	0.011142
BMP3	0.450778418	0.1417091	-0.327555526	0.2865475	0.777620939	0.011152

IFNLR1	0.312392492	0.0876306	0.263222813	0.1492419	0.460579865	0.011175
MOCS1	0.077404677	0.7301517	-0.117141669	0.5987235	-0.581669148	0.011173
TRIM25	0.133558399	0.336752	0.093115506	0.5030382	0.351986416	0.011174
SART3	-0.010902392	0.8808368	-0.119835955	0.0973941	-0.18427877	0.011279
WDR75	-0.040111453	0.6459154	0.098601482	0.254426	0.218851664	0.01132
SLC13A4	-0.910656351	0.1008493	-0.634872256	0.2423999	-1.403015208	0.01138
SCP2	-0.101743298	0.3337056	-0.144307567	0.1693444	0.264413606	0.011588
NAALADL1	-0.511961991	0.4319278	0.001682171	0.9978843	-2.187958707	0.011611
NMB	0.376112676	0.1121248	-0.049042646	0.8371754	0.589819652	0.011608
SLC51B	-0.589869574	0.3495264	0.268634789	0.6639719	-1.639940567	0.011644
FAM9C	0.192200593	0.5697983	-0.474382711	0.1676131	-0.880118818	0.011738
OLFM3	1.74327739	0.2040422	1.853238795	0.1745078	3.423984797	0.011734
CLDN11	0.650215757	0.3191116	0.282606639	0.665132	1.63895865	0.011779
EFNB3	0.140159905	0.6433892	-0.040993542	0.8921841	-0.775488209	0.011816
ARL6IP1	0.080069636	0.4756003	0.094707919	0.3982181	0.282082311	0.011843
RAB38	-0.377696618	0.1792949	0.172894126	0.5318937	-0.71119027	0.011845
RP1-95L4.4	-0.491339914	0.2725247	0.005739093	0.9891766	-1.198176221	0.011843
BLZF1	0.027973256	0.7327849	0.004455779	0.956124	0.202889636	0.011861
IFIT3	0.42290265	0.1327956	0.463294823	0.0969561	0.699781867	0.011886
KRAS	-0.12270903	0.1525046	-0.108551454	0.203331	0.213912805	0.011939
LRRFIP1	-0.015317389	0.8795302	0.011888972	0.9060978	0.252888242	0.011949
EPS15	-0.033954426	0.7500233	0.082702742	0.4362829	0.266090341	0.01205
RAD9A	-0.150239052	0.4001591	-0.180608471	0.307222	-0.451032181	0.01208
PSMG4	0.24957754	0.1381266	0.130299077	0.4366652	0.419003975	0.012106
BCL9	-0.494532342	0.3585576	-0.687391059	0.2005412	-1.362437337	0.012137
CYP2C19	0.175916804	0.467928	0.46252405	0.055397	0.604968341	0.012155
NDEL1	0.001143439	0.990815	-0.042894611	0.6660584	0.247957802	0.012204
MGP	0.795645114	0.2656684	0.819681977	0.2514107	1.788083255	0.012266
PSME1	-0.00865107	0.9237953	-0.063500914	0.4801906	0.224201277	0.012268
SEMA4A	-0.044992015	0.8823616	0.032977318	0.9131781	0.741806893	0.012263
RTCB	0.010232868	0.9050564	0.043511277	0.6095351	0.2130543	0.012289
ADH1C	-0.266554265	0.6100599	0.035127973	0.9461351	1.293941009	0.012356
HN1	0.032997967	0.7545937	0.10400347	0.3228084	0.262874799	0.012353
GUCD1	0.171645849	0.1402638	0.178079137	0.1249362	0.290088069	0.012457
FHOD1	-0.198207919	0.2467024	-0.225358291	0.1850765	-0.425971586	0.012606
3-Mar	0.123638409	0.5870026	0.374123457	0.0955539	0.558151215	0.012672
S100A11	0.240830421	0.1383486	-0.008283689	0.9593473	0.404834732	0.012678
NFRKB	-0.007721209	0.9359279	-0.155319642	0.1046418	-0.23968217	0.012706
FAM91A1	0.095447391	0.3098832	-0.021369476	0.8197359	0.233274901	0.012746
RABGGTB	-0.043819811	0.6139366	0.039411603	0.6485271	0.215203167	0.012745
SIX1	1.011294752	0.3441492	1.838141419	0.0848459	2.652658112	0.01277
G3BP1	0.154065631	0.0998955	0.142275223	0.1283845	0.232938103	0.012788
BCAS1	0.002489943	0.9915614	-0.276292056	0.2406644	0.585159385	0.012828
PPARA	-0.054789053	0.7536869	-0.21525081	0.2175246	-0.435519631	0.012846
MAP2K1	0.24211092	0.1005551	-0.040241931	0.7853253	0.365404147	0.012893
NKPD1	-0.527753197	0.222523	-0.487987351	0.2483113	-1.136903711	0.012949
DENND1A	-0.205781869	0.1789275	-0.235162635	0.1225816	-0.381860079	0.012974

NMI	0.137612494	0.547471	0.077033881	0.7341248	0.552892146	0.013
PLP2	0.10740765	0.5169321	-0.121005992	0.4652741	0.410231223	0.013061
CORO7	0.263771412	0.107671	0.238316158	0.1432703	0.402744557	0.013076
EZR	0.20580599	0.1051988	-0.120028646	0.344773	0.315046503	0.013099
RABL2A	-0.121214803	0.5265088	-0.292084014	0.1236856	-0.476069193	0.013107
IGSF9	-0.259817202	0.3088573	-0.443303516	0.082348	-0.63560966	0.013137
CFL1	0.129070436	0.1407729	0.120053542	0.170368	0.21705741	0.013179
CCDC14	-0.176332595	0.1440672	-0.029757948	0.8047068	-0.299262503	0.01322
BNC1	0.092198529	0.8787427	0.542359605	0.3664067	-1.532576234	0.013256
GREM2	0.117474244	0.6177072	0.420756462	0.0722164	-0.587309838	0.01335
CLOCK	0.028878763	0.7810459	0.134690696	0.1932941	0.255605856	0.013377
UBE2O	-0.212103992	0.2377389	-0.336580687	0.0599122	-0.444031587	0.013407
AGPAT4	-0.350652202	0.15057	-0.049141173	0.8398512	-0.605628676	0.013413
HERC6	-0.149563621	0.5368005	0.190217271	0.4173636	0.5759107	0.013507
ZMYND10	0.534955268	0.3340057	0.275848253	0.6128993	1.305325243	0.013524
CLASP1	-0.011853623	0.9191193	-0.194589622	0.0950167	-0.289105038	0.013535
NADK	0.142577378	0.1650851	-0.189339253	0.0648755	0.252110805	0.013563
BPIFB1	0.311552153	0.6329332	-0.009839774	0.9879664	1.607101766	0.013608
S100A2	-1.375163997	0.1271758	-0.283511024	0.7492974	2.153400964	0.013671
KRBOX4	-0.195465966	0.2142296	-0.304108028	0.0528687	-0.389823955	0.013891
RPIA	0.042041619	0.7411252	-0.054660436	0.6648988	0.306105599	0.013891
RRM2B	-0.350554388	0.1148078	-0.191351091	0.3881413	-0.546454638	0.013921
ZNF561	-0.155477216	0.1742474	-0.102393342	0.3664863	-0.281176077	0.01393
TOP3A	0.015395767	0.90836	-0.091944985	0.490156	-0.33036493	0.013943
LCT	-0.090711012	0.9109639	-0.283227653	0.7268067	-2.069288901	0.013984
CUL4A	0.160289841	0.0639333	0.058410757	0.496192	0.210766503	0.013997
SLC17A4	-0.412724912	0.5670231	0.107430408	0.8814165	-1.778476164	0.014009
MMP19	-0.019514844	0.9493635	-0.263423837	0.3919553	-0.767736478	0.014076
CDK11A	0.189070109	0.252182	-0.14162851	0.3924508	-0.407518221	0.014127
MTDH	-0.197527748	0.1323801	0.235375689	0.0722961	0.321442162	0.01413
USH2A	-0.664699107	0.0618728	-0.266705757	0.4304109	-0.880290864	0.014115
ATP6V1E1	0.084141945	0.3845217	0.128639318	0.1798614	0.23514839	0.014161
NAA15	0.175067995	0.0910175	0.125784117	0.2236871	0.253449633	0.014189
PRR5L	0.065291431	0.9110108	0.731180148	0.1973094	1.381717553	0.014184
FAM214A	-0.196528212	0.158593	-0.087022101	0.5277838	-0.341016984	0.014212
MFN1	0.135237958	0.1745879	0.043525813	0.6610174	0.242745847	0.014296
BET1	-0.107071262	0.4476194	-0.133203154	0.3410132	-0.345187888	0.014322
MED31	-0.114168947	0.4702811	-0.174911756	0.2657484	-0.397234578	0.014526
AC016773.1	-0.686705542	0.0918683	-0.056908963	0.8811436	-1.014418155	0.014604
ADAMTS20	-0.039089988	0.9090024	-0.457401257	0.1856284	-0.865922422	0.014614
NRG4	-0.023707719	0.9316407	0.000324838	0.9990605	0.664816851	0.014652
CA9	0.219287163	0.5560367	-0.128104615	0.7309583	-0.910705875	0.014685
ZNF773	-0.218399596	0.2050866	-0.043893995	0.7958259	-0.420842901	0.014702
TMEM63C	0.110130162	0.9077339	0.554424464	0.5615657	-2.684542264	0.014709
HOXB5	-0.127658094	0.7721252	0.121507456	0.7767862	-1.159628448	0.014748
IGF1	0.432641109	0.366188	0.362651831	0.4484994	1.165023988	0.014794
ING4	0.032781141	0.8487407	-0.130829591	0.4440534	-0.421417548	0.014798

SNX4	-0.178716761	0.0728063	-0.060961706	0.5375991	0.239973291	0.014806
SH3BGR	-0.651640688	0.2715623	-0.294411486	0.6085585	-1.523520508	0.014836
SYK	0.026973772	0.836179	-0.225301305	0.0839956	0.315775932	0.014843
COL4A4	0.509131861	0.3624008	0.17772034	0.7511942	1.333441657	0.014888
P11-173M11	1.266491614	0.1122128	0.79368217	0.3272963	1.869064947	0.014886
RAD23B	0.090965375	0.4917259	0.189678625	0.1513658	0.321811976	0.014924
ABCB11	-0.471694018	0.5724184	0.420524151	0.5989769	-2.172922179	0.015022
FBXO17	0.02154465	0.8776609	0.063936829	0.6438282	-0.343784237	0.015029
NEK7	0.11335367	0.3474495	0.17536548	0.1445878	0.292210073	0.01503
BAIAP3	0.549679457	0.0880402	-0.112454158	0.7331966	-0.805273966	0.015063
LINC00674	-0.457803948	0.1574357	-0.01262975	0.968613	-0.792364174	0.015082
ACVR2B	-0.053185157	0.6788343	-0.026886419	0.8335766	-0.312432005	0.015168
ATP6V0A1	0.08432081	0.5322025	-0.066708816	0.6217681	-0.330550507	0.015187
TMEM33	0.126617873	0.2252674	0.196931311	0.0584114	0.252781426	0.015185
TXLNG	0.214556536	0.099693	0.233773582	0.0722401	0.315745526	0.015195
HAT1	-0.052110834	0.6392929	0.113548444	0.3030085	0.267274143	0.01523
MAPRE2	0.187143283	0.2820253	-0.102543493	0.5560817	0.421805664	0.015223
PITRM1	0.100481328	0.2861014	0.05749289	0.5397876	0.22735499	0.015239
CLIC6	0.378636085	0.2748049	0.174074045	0.6155747	0.839910216	0.015251
SYT4	-0.537817475	0.5030593	-0.572500173	0.4785996	-2.098670823	0.015278
TMEM30B	0.073588857	0.6499287	-0.210268264	0.1949357	0.391127047	0.015339
BST2	0.101600803	0.7449572	-0.432647936	0.1678207	-0.763465982	0.015356
STOM	-0.01840512	0.921354	0.23811351	0.2008178	-0.452818836	0.015361
COL10A1	0.153733375	0.7537188	0.133686903	0.7785135	1.151731025	0.015421
MAPRE3	0.057750271	0.7991068	-0.223198517	0.3269971	-0.564365246	0.015418
TMEM145	-0.946904267	0.0722215	0.502397266	0.3373289	-1.315129928	0.015419
TAGAP	0.935667424	0.0724087	0.413010699	0.440286	1.253371563	0.015456
NBL1	0.236740653	0.1884788	0.259659339	0.148392	0.434071127	0.015494
WASH2P	-0.172837044	0.2865601	-0.197852602	0.2212989	-0.39344321	0.015613
C18orf8	0.083807799	0.5565199	0.023929546	0.8656579	0.339682321	0.015679
ZNF134	0.06374066	0.6278313	-0.07449813	0.5697955	-0.319161511	0.015702
NUP153	0.202250968	0.0695922	0.182548424	0.1010486	0.268872281	0.015728
NEUROD1	-0.12374654	0.8555664	0.06637818	0.9199317	-2.032246284	0.015735
FDXACB1	-0.379649187	0.0967621	-0.185836095	0.4054577	-0.556368653	0.015777
PANK3	0.070876617	0.461428	0.038463958	0.6890981	0.232015858	0.015787
PSME2	0.184029753	0.0699653	0.054852609	0.5881085	0.243883014	0.015847
HLA-DRB5	-0.182340499	0.7911874	0.052570391	0.9390745	-1.672838251	0.015859
ACMSD	-0.679416875	0.2270328	0.154889345	0.7766283	-1.424537834	0.015875
UQCRFS1	0.005939527	0.9513365	0.051219363	0.5968201	0.233197556	0.01588
PDE4C	0.162235376	0.4249196	-0.251020239	0.2196206	0.488808323	0.015892
FAM193B	-0.233706961	0.1288875	-0.293108873	0.0563668	-0.372606328	0.015926
EBP	-0.11608019	0.3811919	-0.031798121	0.8099804	0.318104033	0.01597
PTCD1	-0.092238759	0.5971476	-0.167743718	0.3325315	-0.420720109	0.016011
ECHDC3	0.235307882	0.7481122	0.219566113	0.7594969	-2.072525432	0.016024
GLTPD2	0.228704082	0.5958822	-0.00329976	0.9938937	-1.087744747	0.016035
SGIP1	-0.329812332	0.16889	0.208061424	0.3761316	-0.581594107	0.016053
HEPACAM	-0.192088961	0.6532856	-0.105821964	0.8021259	-1.070722366	0.016088

IGFN1	1.050453931	0.3640441	0.236252284	0.8391269	2.754251037	0.016165
RALA	-0.128200287	0.2675779	-0.058043471	0.613791	0.275613671	0.016316
DISC1	-0.178476226	0.4961074	0.009720358	0.9702102	-0.634608164	0.016341
WFDC3	0.429786326	0.383121	0.035785015	0.9422776	1.130251075	0.016368
GRB10	0.367854161	0.1033421	0.29965281	0.1841191	0.540842041	0.016395
ASL	0.026640415	0.8692323	-0.001451774	0.9928268	0.386900787	0.016437
PRSS36	-1.069685775	0.0573815	-0.36308299	0.4964665	-1.347495595	0.016497
AFG3L2	-0.079059609	0.446468	0.034361523	0.7392652	0.246973996	0.016602
MYH14	0.265494237	0.3735924	0.444386368	0.1361021	0.71389273	0.016625
PIP5KL1	0.243533937	0.410466	-0.161365302	0.5882344	-0.752806047	0.016643
TGFB2	0.802658423	0.3031055	1.091994241	0.1607999	1.86255511	0.016639
RANGAP1	0.244724887	0.1010659	0.056762102	0.7033674	0.356075402	0.016733
LRRFIP1P1	-0.164113376	0.2910266	0.102092036	0.4943881	0.353801962	0.016748
EXOSC10	0.116703586	0.0919937	0.079105686	0.2504453	0.164571793	0.01679
IL1RAP	0.249516873	0.215959	0.158230643	0.4322987	0.480969101	0.016863
CETN3	-0.236269288	0.1735525	-0.220438856	0.198623	-0.414267215	0.017086
GPC6	0.040965554	0.7974307	0.10545826	0.508327	-0.381008972	0.017103
MAGEL2	0.140930466	0.6547833	-0.414756042	0.1942702	-0.781272233	0.017117
FAM198B	-0.209643234	0.3742092	-0.17862775	0.4481386	-0.562398061	0.017157
SAP18	0.028871163	0.7311641	0.024359003	0.7708778	0.19887256	0.017336
CCDC88A	-0.206458773	0.218245	0.288543618	0.0840088	-0.399394119	0.017358
SLCO2A1	0.462366511	0.1237561	0.014757635	0.9608731	0.712879315	0.017461
FHAD1	0.348079821	0.3511403	0.530607626	0.1475815	0.862184138	0.017514
KEAP1	-0.088234241	0.5901157	-0.125598172	0.4421263	-0.388610727	0.017522
FDFT1	0.27784713	0.0590452	0.107457117	0.4652306	0.349353351	0.017561
OSTCP1	-0.571158734	0.110216	-0.336056325	0.3233633	-0.865737724	0.017564
AXL	0.052510783	0.7978991	-0.147643184	0.4714352	-0.489172695	0.01761
DLST	0.118166174	0.167357	0.112429965	0.1869789	0.202145518	0.0176
ARPC1B	0.031103396	0.8174754	0.226154867	0.0918241	0.318344115	0.017645
TMEM178A	-0.436958049	0.15988	-0.304652509	0.3091865	-0.735053697	0.01765
ZSCAN21	-0.041428011	0.8204648	-0.310652215	0.0885418	-0.437815359	0.017684
THAP9	-0.266930346	0.2268937	-0.15638689	0.4741744	-0.522159278	0.017742
AC093642.5	0.087268262	0.8045559	0.252136266	0.4682658	0.811813526	0.017787
MYL12A	-0.137989027	0.1029783	0.012865406	0.8787657	0.199740835	0.0178
GRHL1	0.310275426	0.0761923	-0.064165197	0.7134269	0.411051164	0.017852
IDI1	0.014523028	0.91717	0.13857222	0.3205779	0.330382616	0.017855
PMCHL1	-1.162695138	0.0865106	-0.704463813	0.2702332	-1.63581692	0.017856
TIMM17A	0.21337388	0.0538371	0.207645418	0.0590308	0.260572578	0.01793
ZNF614	-0.054598099	0.6814396	-0.245620665	0.0639567	-0.315665324	0.017965
DBR1	-0.178480641	0.3022569	-0.02089998	0.9034823	-0.409516756	0.017978
SUMO3	0.10166402	0.272475	0.142355603	0.1224253	0.218092884	0.018001
FABP3	0.197107463	0.5033138	0.309015672	0.2922224	0.691355275	0.018025
AFG3L1P	-0.150569917	0.3075055	-0.184632216	0.2089796	-0.34999855	0.018054
RPL36AL	-0.083345908	0.4799396	0.078264059	0.5060565	0.278091628	0.018071
RARRES2	0.184647889	0.4492352	0.255862612	0.2890723	0.566701039	0.018085
C4orf33	0.054483265	0.7117802	0.02663183	0.8547682	0.341100848	0.018093
FAM124A	0.792334994	0.0564507	0.797012705	0.0503684	0.968279052	0.018124

CHRNG	-0.531756837	0.1775674	-0.158912155	0.6663115	-0.944952467	0.018164
RPS2P7	1.276416459	0.0576534	1.256365605	0.0573482	1.540485878	0.018201
UNC13D	0.223195944	0.4882879	0.270132304	0.4010656	0.755764926	0.018194
PTPN22	0.269403525	0.6106452	0.048515878	0.9279335	1.207545314	0.01827
TBC1D30	-0.358625952	0.0887421	-0.07339486	0.7227221	-0.491941123	0.018272
P11-162P23	0.811636926	0.1355591	0.918280368	0.0904347	1.277479313	0.018313
DND1P1	0.259305768	0.8605162	-0.391132359	0.7923139	-4.172409064	0.018364
CGRRF1	-0.341603479	0.0717762	-0.214880345	0.2500048	-0.447329051	0.018414
GPR20	-12.13727267	0.0923922	-12.22239294	0.0901297	16.83192908	0.0185
HSP90B2P	-0.265009328	0.5490019	-0.06229028	0.883901	-1.128002321	0.018535
NTN1	0.364613667	0.3006208	0.501011851	0.1536968	0.825169028	0.018546
SYTL4	0.077919627	0.5927403	-0.099585544	0.4935	0.340229452	0.018547
CLDN19	-0.174931442	0.6340522	-0.309452497	0.3924179	-0.914975752	0.018558
C16orf87	-0.100325233	0.4942048	-0.031336291	0.8295251	0.340755041	0.018632
CCNC	-0.012618449	0.9018144	-0.022920527	0.8221391	0.239232883	0.018728
ITGB3BP	-0.370458478	0.0667207	-0.186475526	0.3540371	-0.474375111	0.018747
UPP1	0.466027834	0.1060729	0.055635078	0.8469583	0.672437283	0.018743
PPP1R11	0.207050363	0.0725345	0.070338563	0.5411429	0.269729654	0.018797
ZNF781	-0.451878779	0.2343488	-0.555938438	0.1410551	-0.897528084	0.018822
CTC-471F3.5	-0.308360301	0.3531365	-0.257877996	0.4229777	-0.817724885	0.018897
RASL12	0.720295777	0.1873897	0.035421182	0.9485737	1.271703384	0.018959
P11-239L20	-0.935061817	0.247357	0.074430337	0.9159425	-2.4298091	0.01919
PSMD8	-0.024234603	0.8243716	0.185894505	0.0874292	0.254704527	0.019215
ZNF652	-0.100805082	0.4208873	-0.229716238	0.0661497	-0.293879217	0.019209
MTMR1	-0.086246282	0.5448631	0.231124951	0.1038206	0.332114679	0.019229
CLHC1	-0.391436184	0.0775067	-0.053421047	0.8061021	-0.51420372	0.019237
HLA-DMB	-0.332632713	0.5396405	-0.841578816	0.1195061	-1.318712952	0.019345
EI24	0.172596067	0.1935118	0.047620582	0.7195263	0.309856675	0.019362
ORMDL2	0.132908939	0.357591	-0.16402909	0.2560621	0.336372269	0.019398
RBMS1	0.094149572	0.2892815	0.070470608	0.4270025	0.207269053	0.019393
RNASE4	0.132257002	0.4996761	-0.17062478	0.3841712	0.456965577	0.019433
MFSD7	0.224430607	0.2919474	-0.383689635	0.0821556	0.488087569	0.019467
SEC23B	0.076613824	0.557027	-0.025174949	0.8467344	0.303933778	0.019457
ARHGAP8	-0.232864543	0.2688881	-0.015783861	0.9399691	-0.492073086	0.01965
APOL6	0.232263894	0.1526503	0.080890733	0.6181519	0.377618966	0.019782
IGDCC3	0.150162644	0.5947174	-0.173704327	0.5384005	-0.660311941	0.019777
INPP5A	0.059724334	0.6367105	0.010145943	0.9357926	0.293442185	0.019779
MST1L	-0.513946386	0.2882876	-0.481201676	0.3174794	-1.176586138	0.019765
TRPV3	-0.334694736	0.2553583	-0.206905502	0.4710637	-0.680247653	0.019755
TICRR	-0.216555338	0.2234013	-0.277391393	0.1174317	-0.414850009	0.019793
MTA1	0.11203796	0.3775209	0.049847287	0.6932576	-0.296194054	0.01985
PSME3	0.26071601	0.0628497	0.181600742	0.1943641	0.325861283	0.01989
TMCO6	0.096464403	0.6119506	-0.204647815	0.28114	-0.447065324	0.019993
KIAA0895L	0.126956184	0.5254179	-0.143766366	0.4734287	-0.469078548	0.020028
ZNF34	-0.250684639	0.1538917	-0.192386163	0.2566585	-0.408342438	0.020025
FOXC1	1.17228888	0.1079704	1.057582577	0.1467805	1.691214276	0.020059
BCO2	-0.369737402	0.2617208	0.169563181	0.5972736	-0.78019472	0.020089

CCT6P3	-0.160994132	0.2172	-0.239908847	0.063956	-0.304357966	0.020093
HOXC11	-1.442285095	0.0802665	-0.01528437	0.9844228	-1.949675781	0.020098
KANK4	0.763813179	0.0574386	-0.005189555	0.9897896	0.93248499	0.020101
AP1M1	0.078330985	0.5419521	-0.060979534	0.6334879	-0.299238968	0.02012
GNG4	-0.170753722	0.5840712	0.05510782	0.858906	-0.729022946	0.020167
LDHA	-0.179674065	0.1524619	0.076224669	0.5436944	0.291577528	0.02018
UBE2Q2P6	0.095774294	0.4096904	0.162593362	0.1613117	0.269654875	0.020178
EMB	-0.314832339	0.2437118	-1.44E-05	0.9999571	-0.628129709	0.02022
MED7-TICAI	-0.466420988	0.1939318	-0.23724416	0.5048991	-0.836016862	0.020251
P11-480I12	-1.152747941	0.0690674	-0.816657038	0.1697766	-1.515638254	0.020397
ARL8A	0.36481642	0.1008323	0.411335293	0.0627041	0.516178514	0.020427
NLK	0.033513413	0.7575206	-0.086652503	0.4196686	-0.252233921	0.020495
BEND6	-0.342228624	0.5327883	0.802167108	0.1211025	1.203203401	0.020518
GPD1L	-0.127549583	0.3991134	-0.153697744	0.3064795	0.345662305	0.020522
E2F7	0.158919224	0.5513852	0.344363694	0.1960426	0.616008824	0.020579
SHROOM3	0.211056107	0.1842656	0.120494761	0.4482302	0.367598738	0.020604
MAFG	0.194746284	0.0838244	-0.036630739	0.7447182	0.258254959	0.020683
RDH11	0.027212972	0.8035497	0.03740162	0.7319677	0.252501911	0.020695
EOGT	0.016371593	0.9099267	0.230768778	0.0971436	0.324199258	0.020738
15-Sep	-0.00628278	0.9369951	0.007809321	0.9213157	0.182427369	0.020806
ABHD1	-0.493907502	0.3702173	-0.860184317	0.1142308	-1.352819758	0.02089
KIDINS220	-0.1139297	0.2386555	-0.040941646	0.6712605	-0.223211377	0.020922
TAF8	-0.096554998	0.3911293	-0.019019834	0.8651203	-0.261143759	0.020925
SEC14L4	0.037649014	0.9143087	0.131260597	0.6985163	-0.897025955	0.021072
WDR1	0.122918656	0.216568	0.044270737	0.655971	0.229090354	0.021107
KPNA1	0.178199239	0.0794583	0.138716774	0.1704006	0.233139036	0.021299
MURC	0.131179985	0.7150323	-0.138930029	0.6998084	-0.909649951	0.021291
RAB15	0.277018139	0.0751763	0.036788624	0.8134303	0.35762155	0.02134
STK31	-0.138285067	0.6962588	0.37340201	0.2785457	0.784745812	0.021356
GNG5	-0.052504212	0.6051427	0.190466215	0.0593003	0.232519223	0.021373
PPP1CB	0.03813058	0.6524918	0.094895419	0.2615981	0.194490479	0.021391
BAD	0.341893023	0.0772365	0.041075604	0.8320664	0.442651945	0.02142
RP11-3L10.3	0.285549986	0.4926307	-0.285181131	0.5009926	-1.040791862	0.021413
HSD17B11	-0.350716572	0.0987171	0.026271347	0.901125	-0.488172004	0.021452
SLC38A1	-0.003251414	0.9794739	0.138116727	0.2738389	0.290215499	0.02146
PRAP1	0.015839617	0.9867824	0.726059147	0.4470827	-2.212518669	0.021551
VAT1	-0.027119228	0.8510734	-0.211388879	0.1430449	-0.332814467	0.021569
P11-192H23	0.053405801	0.7918946	0.156165672	0.4337644	-0.479386956	0.021579
BATF2	-0.020460154	0.9648127	-0.631348977	0.1802316	-1.188449002	0.021644
CHST2	0.59106343	0.0524384	0.475876935	0.1175502	0.696363698	0.021725
KLHL26	-0.304529651	0.2663643	-0.109561613	0.6831309	-0.632645081	0.021737
PLEKHA3	0.164803988	0.236214	0.076381293	0.5823587	0.318147621	0.021778
BOC	0.889693949	0.0581017	0.566442597	0.2280044	1.076047914	0.021838
C19orf25	-0.231894434	0.1074637	-0.055957042	0.6939414	-0.332669251	0.0219
PIGCP1	0.04536932	0.8331785	0.119987676	0.5675146	-0.5147796	0.02202
GMPS	-0.070435515	0.4777881	0.070823746	0.4733054	0.226229415	0.022035
KLHDC4	0.12506285	0.4646318	0.145315753	0.3902912	0.387025877	0.022065

ANKRD16	-0.169930629	0.4343359	-0.368274903	0.0884318	-0.500987441	0.02213
IL1R2	0.602723862	0.1691463	0.553278555	0.2065641	0.993933316	0.022126
DCLK2	0.180595031	0.5311265	-0.370068266	0.2008856	-0.667833558	0.022162
GAP43	1.611703379	0.0952926	0.818630834	0.3987046	2.202294851	0.022186
ARHGEF38	0.36525056	0.2455684	0.366241586	0.2435407	0.715799183	0.022217
ENPP5	-0.423272528	0.1088259	-0.253616262	0.3269429	-0.607524936	0.022241
GTF2A2	-0.078422378	0.4817741	0.213992964	0.0521098	0.252347975	0.022278
KIAA1199	-0.587108604	0.2682167	-0.446766705	0.3992666	-1.212705799	0.022316
SUFU	0.248208309	0.1948453	-0.072225714	0.7083465	-0.454307173	0.022334
CALCR	-0.501215324	0.4734289	-0.762697871	0.2731104	-1.62941922	0.022372
EIF3B	0.070046938	0.4664227	0.121617877	0.2052971	0.219309698	0.02237
DCAF6	0.120641802	0.3296848	0.125436972	0.3097221	0.281598105	0.022467
TRAF3IP1	-0.370055354	0.05775	0.026815347	0.8895393	-0.44301237	0.022487
SLPI	-0.156172207	0.7889982	0.471306518	0.4158863	1.316870749	0.022517
NFASC	0.493371549	0.3348425	0.1280221	0.8013439	1.152897275	0.02253
PRKCG	0.951644568	0.1763907	-0.041775871	0.9544499	1.602939526	0.022642
RANBP10	0.102529481	0.5201078	-0.209899371	0.1891253	-0.376397804	0.022629
BSN	0.376714648	0.4406254	-0.193631841	0.6923137	-1.151877889	0.02268
KCNK6	-0.325918334	0.0769929	-0.265566626	0.1483658	-0.419697916	0.02269
NT5C2	0.035273938	0.7340823	-0.05514006	0.5951827	0.236055336	0.022697
TCEB1	0.19250516	0.0581786	0.129782579	0.1984455	0.230160515	0.022688
AMIGO1	-0.3922489	0.058285	-0.366481682	0.0671306	-0.46484182	0.02274
COLEC11	-0.772511622	0.1731848	-0.887791887	0.117305	-1.29576791	0.022748
PDE1B	-0.502755493	0.2682197	-0.52417747	0.2506772	-1.04959409	0.022748
WNK3	0.100383371	0.6354934	0.177986617	0.398549	-0.485858615	0.022798
CACNA1E	-0.850627201	0.2780632	-1.381669714	0.0770809	-1.818513648	0.022813
DLK2	-0.655172502	0.0574924	-0.13762876	0.6558322	-0.779866406	0.022833
SARS	-0.020565922	0.8469635	-0.00777054	0.9417219	0.241663312	0.022837
SETD1B	-0.09061105	0.6862867	-0.216314113	0.3331437	-0.517601883	0.022862
SMLR1	-0.129941982	0.8074694	0.459691816	0.3730675	-1.301324997	0.022929
ZNF566	-0.140062961	0.4939803	-0.256763899	0.2055507	-0.463994326	0.022927
TFF1	0.099338449	0.6587375	-0.222217105	0.3231726	0.511506129	0.022947
NPM1P26	0.288590256	0.2754111	0.396991066	0.1237886	0.582925991	0.023028
PACSIN3	-0.209932953	0.2537616	-0.273212032	0.1367088	-0.417693232	0.02303
CCNE1	-0.396586735	0.0583573	-0.199872211	0.3370029	-0.474952485	0.023102
POLI	-0.204297047	0.187265	-0.090587182	0.5562466	-0.351635546	0.023135
CHMP2A	-0.020111837	0.8009036	-0.050360496	0.5240064	0.178325011	0.023148
AQP1	-0.49705264	0.3554578	-0.428058442	0.4239777	-1.245989313	0.023159
C10orf11	0.100397319	0.8421244	-0.719748716	0.1480158	-1.159216886	0.023168
ZNF865	0.138464689	0.592601	-0.372403559	0.1592841	-0.620744341	0.023209
LUC7L3	-0.221363982	0.1323401	-0.12948536	0.3783226	-0.334048283	0.023226
EIF1B	-0.180993972	0.1654457	-0.112922317	0.3809211	-0.295559545	0.02328
GAB2	-0.12280957	0.4574746	0.013382906	0.9349535	0.370717581	0.023299
HID1	0.139156435	0.5066616	-0.125752214	0.5485472	0.474407636	0.02326
KPNA6	0.224155043	0.0583064	0.038338181	0.7461563	0.268500286	0.023297
P11-430C7	-0.325564751	0.6144812	-0.569508131	0.3786677	-1.747383832	0.023274
MYO1H	0.473489328	0.4522835	0.716689231	0.2494566	-1.483363997	0.023322

RORB	1.194343595	0.1374596	0.279464551	0.7301775	1.815123779	0.023331
SCNN1B	0.523844032	0.4101428	-0.166869854	0.7957544	1.431245546	0.023331
KNG1	-1.221322474	0.2199005	0.817628027	0.3764042	-2.519460892	0.023365
MAP4K1	0.036192881	0.9084039	-0.494803922	0.1200893	-0.737880996	0.023358
SLC25A51	0.012646658	0.9020087	-0.125070026	0.2198771	-0.235098107	0.023397
SPIN4	-0.133922737	0.5484904	0.22130798	0.3183475	-0.507591525	0.023397
HEPH	-0.165334258	0.5455474	0.027091051	0.9210927	-0.620033583	0.023428
CHRM2	-0.008680217	0.9736547	-0.252568213	0.336435	-0.596466244	0.023477
MYO7B	-0.590557655	0.3549439	0.201044062	0.7523152	-1.447428792	0.023578
RAPGEF6	-0.056186054	0.7015504	-0.006380526	0.9651897	0.330671881	0.023627
GRID1	0.368947141	0.5011049	1.033654675	0.050947	1.183833436	0.023643
ARFGEF2	0.1405881	0.0862382	-0.003111533	0.9696509	0.184632476	0.023725
CHIC1	-0.115438752	0.4620994	-0.065817272	0.6721767	-0.355703073	0.023726
WDR65	-1.220031441	0.0505755	0.223343603	0.7045204	-1.335005943	0.02377
TMSB4X	0.215347307	0.1488286	0.091640904	0.5389622	0.337105377	0.023812
NDUFA9	0.398727793	0.0759786	0.21428674	0.3396228	0.507247135	0.023855
RAP1GAP2	0.285781215	0.4708669	0.535284952	0.1745798	0.888426513	0.023894
ALG1L13P	-0.662890296	0.1879798	-0.628894385	0.197873	-1.222527558	0.023924
TCEANC2	-0.199985982	0.2456923	-0.290997865	0.0852243	-0.387795556	0.023915
CDRT1	1.01259092	0.0983094	0.649002534	0.2788056	1.352746708	0.024044
RPS24P8	-0.227335282	0.7224686	0.595083664	0.2975746	1.239821279	0.024068
DTX1	1.391033458	0.194701	0.578716508	0.59882	2.382139446	0.024099
PITX1	-0.009649006	0.9836301	-0.032388654	0.9450514	1.03226295	0.024107
SACS	-0.182195276	0.279153	0.055074943	0.7430962	-0.379634631	0.024132
PROM1	0.063536671	0.746585	0.178238692	0.3639697	0.442243469	0.024193
CBX1	-0.216980715	0.1041404	0.031081146	0.8152142	-0.300701313	0.024285
CTTNBP2	0.158649942	0.7493671	0.295959545	0.5479427	-1.134923824	0.024307
CYP3A43	-0.349139705	0.4022462	0.329978857	0.3873957	-0.999394691	0.024306
CXADR	-0.033116072	0.8176258	-0.276301845	0.0542733	0.322699321	0.024409
PIGC	-0.00287138	0.9785715	0.082431666	0.4335465	0.236638834	0.024404
SLC7A9	0.966261928	0.1645966	1.079195821	0.1201679	-1.652880694	0.0244
YPEL2	-0.045067121	0.8089566	-0.149545795	0.4215833	0.416211023	0.024429
CDA	-0.509971475	0.3634433	-0.197718123	0.7224512	-1.28360246	0.024477
NKAP	-0.177171143	0.1805734	-0.195333839	0.1367409	-0.298023901	0.024496
2-Sep	0.081997221	0.1322922	0.097079777	0.0740112	0.122255155	0.024514
PDZD3	-0.303491844	0.6420478	0.094283325	0.8842544	-1.484432378	0.024656
TP53RK	0.183874849	0.1563082	0.052670955	0.6837363	0.289936089	0.024801
PIDD	-0.450276947	0.0749355	-0.301691608	0.2310526	-0.569393817	0.02483
ENKUR	-0.316116168	0.5902072	0.224753376	0.6922773	1.241011402	0.024853
FUK	-0.102295772	0.4997248	-0.139116528	0.3562411	-0.340063046	0.025054
NDUFS1	0.036508915	0.6264445	0.098325718	0.1879877	0.167483413	0.025054
RAB3C	0.816731329	0.2333752	0.39705282	0.5632687	1.523486726	0.025056
ALPI	0.475482404	0.5894134	0.047790776	0.9570101	-2.470257052	0.025141
POC1B	-0.052918206	0.7508565	0.094315541	0.5697634	0.370604193	0.025258
P1-179N16.	-0.037477023	0.9339384	-0.933104769	0.0544886	-1.127996931	0.025377
RPS3AP5	0.064254127	0.926835	0.450865596	0.5019054	-1.997963356	0.025373
ING1	-0.139214213	0.3790486	0.194924325	0.2090409	0.347768649	0.02541

CLTB	0.080031633	0.6426305	0.194362945	0.2574893	0.383004756	0.025448
AKT1	0.176651555	0.0927716	-0.09573444	0.3622175	0.234487955	0.025459
SEC22A	0.225567358	0.2381503	-0.112590917	0.5554519	0.425092738	0.025505
SLC8A1	-0.053171212	0.874892	0.572968327	0.0891704	-0.756210541	0.025495
VAPB	-0.018702675	0.854567	0.006685852	0.9473993	0.226156526	0.025502
CAP1	-0.025177949	0.7924067	0.078887224	0.408978	0.213277874	0.025541
SIRT5	0.034005248	0.8317226	0.066209424	0.6778771	0.355056528	0.025558
GFRA2	1.054365247	0.4218911	1.148500493	0.3667145	2.7737118	0.025642
ZNF668	-0.066334507	0.8218859	-0.346416442	0.2375085	-0.660293935	0.025647
SH2B1	-0.148730913	0.4496711	-0.28495206	0.1467961	-0.43973506	0.025719
FAM171A2	0.061921478	0.8269125	-0.227263729	0.4221803	-0.636466118	0.025788
ZNF527	-0.015722891	0.9319078	0.025850742	0.8865434	-0.41036837	0.025863
PPP1CA	0.020350952	0.8034234	0.078653452	0.3330286	0.180745504	0.026062
RASA4	-0.078669464	0.7541366	0.143439286	0.5666267	-0.562289224	0.026058
R5-ARHGA	-0.014527967	0.9853255	0.015741638	0.9840362	1.73369008	0.026164
TAS1R3	-0.234963319	0.3898079	-0.460262304	0.0917226	-0.625591897	0.026215
SWSAP1	-0.368669403	0.0843602	-0.141909518	0.4890911	-0.473081389	0.026293
HMGN2P46	0.281247652	0.293685	-0.351184534	0.1970052	0.571439829	0.026329
CDK7	0.647394809	0.149071	0.3657025	0.415034	0.992382728	0.02645
BCL2L2	0.213226508	0.0911909	-0.075890428	0.547344	0.2781364	0.026509
ELMO2	0.060923937	0.6434229	-0.002942454	0.9820788	-0.292346767	0.026501
RHBDL1	-0.744324062	0.0587335	-0.671039744	0.080497	-0.887642786	0.026496
CPA3	0.827265545	0.2049006	-1.176790024	0.0899643	1.41474058	0.026523
TLDC2	-0.138049359	0.8579736	0.500597816	0.5114239	-1.813746492	0.026531
AMOTL1	0.06573882	0.6656931	0.02970828	0.8449537	-0.33732637	0.026661
SPATA25	-1.144120524	0.0629621	-0.816620879	0.1530581	-1.385796826	0.026664
TM6SF2	-0.558899255	0.3348946	0.238951223	0.676767	-1.300357621	0.026659
KLHL32	0.486776041	0.5088444	-0.458448335	0.5349948	-1.857544075	0.026676
P4-631H13.	-1.405481931	0.0626122	-1.312749484	0.0726868	-1.701548377	0.026699
GALNT10	0.253931273	0.1754514	0.359083423	0.0548218	0.414043686	0.026724
PKD1L1	0.27919103	0.5418219	-0.484996582	0.2958602	1.011068494	0.026796
BCL11A	-0.459528578	0.152777	0.046432365	0.8838147	-0.710424249	0.026851
CACNG6	0.303245931	0.5014486	-0.026581713	0.9531255	0.982169115	0.026817
FBXO36	0.10260445	0.4835401	0.174404531	0.2224964	0.313279347	0.026846
P11-797H7.	-0.26359073	0.6530924	-0.21720775	0.7032243	-1.514009183	0.026846
EIF3A	0.147059377	0.080348	0.068461869	0.4153064	0.185936922	0.026924
PLA2G12B	-0.67752241	0.4938263	0.444508843	0.6501566	-2.251488371	0.026946
INHA	0.022740261	0.93635	0.032926977	0.9076518	0.623491376	0.027035
ITGA2	0.469547545	0.0938819	0.491206444	0.0795494	0.619432946	0.027035
USP32	0.080240103	0.4387321	0.120469272	0.2425234	0.227735214	0.027031
IGFBP3	-0.317472312	0.3912931	-0.237281879	0.5216351	-0.818876575	0.02705
NFYA	0.059888061	0.6275332	-0.202566615	0.1009429	-0.273333959	0.027121
RNF13	-0.034041933	0.7742484	0.054754533	0.6426982	0.260485832	0.027172
XK	-0.060000285	0.7851008	-0.183035433	0.4051781	0.484394654	0.02718
P11-347C18	-1.020651612	0.1263133	-0.553695846	0.3850271	-1.519646488	0.027405
TNFRSF25	-0.29337762	0.219819	-0.059130888	0.8023758	-0.540229948	0.027414
SPTLC2	-0.113264945	0.3600977	-0.196946969	0.1112436	0.272171449	0.027434

CHCHD7	0.004480994	0.9696267	0.100945165	0.388	0.257468433	0.027462
OTUD4	0.189444592	0.1006194	0.216273426	0.0602827	0.253572023	0.027596
CYP51A1	0.162666946	0.2324889	0.065508918	0.6305368	0.299964029	0.027618
DAG1	0.154804023	0.2838979	0.052320016	0.7170861	0.317771878	0.027642
GLCCI1	-0.391976445	0.122793	-0.312291554	0.2176774	-0.561977795	0.027678
LIX1	-0.404083515	0.4376976	0.737896969	0.1549516	-1.1497489	0.027691
EEF1A1P25	0.641102593	0.1661703	0.654616892	0.1487325	0.980695924	0.027769
ESM1	1.043018149	0.3275938	1.123029043	0.2828028	2.265770443	0.027795
P11-848G14	0.115367837	0.6493552	0.129851213	0.6052729	-0.57385666	0.027784
ZNF396	-0.420349117	0.1824935	-0.311453223	0.3118557	-0.691283915	0.027793
GBP6	-0.377368902	0.3958464	-0.378990472	0.3850784	0.900628984	0.027881
LGALS4	-0.298391949	0.5944684	0.043568699	0.9380292	-1.232592754	0.027915
DRG1	-0.186731213	0.051059	-0.027024475	0.7762059	-0.209749534	0.027938
CTNS	0.104097431	0.5044397	-0.070099947	0.6527588	0.340577051	0.027952
CDK18	0.110674485	0.6524753	0.082199103	0.7372265	-0.548553101	0.027966
EML2	0.191227416	0.3621384	0.005290943	0.9798467	0.459413123	0.027985
GPR133	-0.029211685	0.920877	-0.038177306	0.8966488	-0.64850977	0.028122
APBB2	2.10E-05	0.9999097	0.235255072	0.2045339	0.405950909	0.028174
CYB5B	-0.137255074	0.1077825	0.116897649	0.1684525	0.186379483	0.028204
PALM3	-0.48684147	0.1190342	-0.562206808	0.0670309	-0.692647375	0.028219
ENOX2	0.066821951	0.760678	0.302704718	0.1639349	0.472504475	0.028239
RAB25	0.216269285	0.185269	-0.052756735	0.7466835	0.357385918	0.028345
HTR7	-0.678100418	0.3821574	-0.68362788	0.3757699	-1.750975608	0.028462
OR52K3P	-0.129076812	0.8191465	-0.614965012	0.2770418	-1.250467362	0.028488
RPAIN	-0.078598111	0.3914928	-0.062845006	0.4876392	-0.200834461	0.028458
RRP12	0.211251247	0.1930507	0.213049518	0.1887202	0.3549333	0.028484
SGCZ	0.045460611	0.9220718	0.018858806	0.9667916	-1.175323728	0.028461
GUCY2C	0.004066296	0.9960318	0.446673892	0.5846395	-1.79710165	0.028527
VSTM5	0.172617729	0.6684218	0.109057499	0.7836236	0.838748496	0.028527
ACTBL2	1.249106034	0.1795465	1.413868479	0.1203637	1.95492222	0.028597
DOK5	1.003144218	0.3839396	0.208844938	0.8561514	2.423999373	0.028697
ARHGAP21	-0.009531108	0.9416438	-0.010633195	0.9348234	0.28414278	0.028803
CDC42P6	-1.015501496	0.383694	-0.585578903	0.6054383	-2.991171223	0.028806
UQCC1	-0.068688815	0.5470536	-0.145919661	0.1978548	-0.249452564	0.028818
IL1RAPL2	0.98205875	0.1281672	0.72901826	0.256965	1.410653404	0.028853
ORM2	0.352391241	0.6554358	0.718213773	0.3493372	1.634777229	0.028914
ADSSL1	0.184606813	0.4996455	-0.452108032	0.0970601	-0.615545416	0.029037
RXRB	0.034210239	0.807579	-0.170180523	0.2270806	-0.308389686	0.029021
SCN3A	-0.452659238	0.3655092	-0.62928417	0.2084889	-1.10465317	0.029038
BCHE	-0.231234953	0.5225035	0.70151895	0.0519055	-0.790437523	0.02906
VSIG10L	-0.199180829	0.5021843	-0.200205578	0.495215	-0.662727604	0.029053
DHRS1	-0.228556075	0.1067465	-0.204702374	0.1474345	-0.308841505	0.029179
NDUFB4	-0.035650802	0.7111918	0.024306022	0.7991189	0.207856503	0.029244
LANCL2	0.013054868	0.9178621	0.13875608	0.270156	0.273961464	0.02936
FKBP2	-0.056562543	0.6605579	-0.028075258	0.8265961	0.277757082	0.029565
KLF7	0.314629556	0.2529365	0.097331285	0.72349	0.596744394	0.029533
RMND1	-0.267215662	0.0551311	-0.091072951	0.5048188	-0.302268385	0.029513

PAWR	-0.000249199	0.9985655	0.060617579	0.6605396	0.299592344	0.029593
PLGRKT	0.020692103	0.9074958	0.100804582	0.5641527	0.377042807	0.029769
LYSMD1	-0.293722859	0.1758101	-0.166602046	0.4302787	-0.468486103	0.029912
PTPRB	-0.43362075	0.0838981	-0.093619353	0.7057832	-0.544950126	0.029901
ZNF622	-0.135390886	0.2050145	-0.201420647	0.057485	-0.231451943	0.029931
MRPL1	-0.295994875	0.0566193	-0.255404556	0.0969956	-0.335355262	0.029968
CHMP4BP1	0.729458132	0.128488	0.690471204	0.1449038	1.014273828	0.030009
A4GALT	-0.948911723	0.3069631	0.516712399	0.5493723	1.837029724	0.030046
P11-405L18	-0.382195851	0.5000858	0.036189508	0.9457905	-1.383579082	0.030051
CYP2C58P	0.702315511	0.3054796	0.717835336	0.2859548	1.409352243	0.030086
DUSP8P5	-0.461820923	0.1825493	-0.187752805	0.5634418	-0.770743538	0.030084
SPATA6L	0.1977861	0.3230643	-0.009084318	0.9635879	0.429220883	0.030096
SLC50A1	0.094561896	0.4897369	-0.153686607	0.2614112	0.294926053	0.030153
PTPN2P1	-0.837357762	0.376177	-0.655222639	0.4691611	-2.753971362	0.0302
ABR	0.036784161	0.9039015	0.017479339	0.954898	-0.680849118	0.030289
BTBD10	0.08358458	0.3523715	-0.002696148	0.9760057	0.193689165	0.030286
UBQLN1	0.018060471	0.8772492	0.144435716	0.2158393	0.252799726	0.030297
NDUFAF2	-0.093382683	0.5838485	0.313780492	0.0603971	0.362179198	0.03035
HSPBP1	0.146432252	0.4026423	0.296853639	0.0864414	0.372537667	0.030374
CROCC	-0.232010224	0.235433	-0.221304617	0.2559011	-0.424047701	0.030444
ZNF3	-0.143938494	0.1674338	-0.103518356	0.3175767	-0.225382941	0.030475
KCNJ2	0.211153136	0.6875422	-0.143423766	0.785557	1.117282112	0.030506
MYO1F	-0.148123333	0.6413862	0.319472426	0.2884926	-0.714653545	0.030527
SETDB1	-0.071824836	0.4629887	-0.122287599	0.2098011	-0.211973287	0.030541
TTC22	0.500095781	0.1492322	0.353141573	0.3081638	0.747743808	0.030557
KLHL14	-0.885883349	0.0880827	-0.356428827	0.4889253	-1.122953632	0.030645
AL139099.1	-0.285975501	0.4947807	0.150371704	0.7025923	-0.967965818	0.030674
CCDC88B	-0.258132193	0.3706458	-0.55728806	0.0514408	-0.624836777	0.030673
CCL7	1.334817753	0.1223592	0.133255557	0.8818449	1.837275267	0.030681
RAB17	-0.249807688	0.2759222	-0.413332897	0.0714544	-0.501056635	0.030708
RRP15	0.175255164	0.2656586	0.135000321	0.3899624	0.339480253	0.030701
IDH2	-0.12101997	0.3288735	0.049058039	0.6909475	-0.267759001	0.030773
GSAP	0.150453122	0.3617471	0.127137404	0.4376697	0.353267123	0.030949
B4GALNT2	-0.363417427	0.0614843	0.003899793	0.9837333	-0.418134989	0.030976
MRPL50	0.068159234	0.5255248	0.175366093	0.0998315	0.230068945	0.030987
ASPN	-0.105549096	0.6950262	-0.266659834	0.3218628	-0.581037105	0.03105
ZNF281	-0.111576071	0.2620723	-0.06829858	0.4906973	-0.214823574	0.031063
DMC1	-0.172186928	0.6484901	-0.057319595	0.874638	-0.816372231	0.031127
BACE2	0.257095711	0.2164859	0.07614033	0.7142538	0.447703637	0.031167
DIRC2	0.034604005	0.8190879	0.281438381	0.0605239	0.323296109	0.03121
C2orf42	-0.089779415	0.5513543	-0.076276638	0.6085456	-0.32409723	0.031222
LRRC37A4F	-0.128818917	0.508161	0.048211102	0.801372	-0.421910128	0.031297
P5-1180C10	-0.117355693	0.6592402	-0.221374812	0.4006544	-0.590050956	0.031311
LRP12	0.191457959	0.3038732	0.140174507	0.4504387	0.399651527	0.031331
CLRN3	-0.480791978	0.3775632	-0.107145283	0.8436779	-1.175528439	0.031598
DENND5B	-0.312649534	0.1378285	-0.20621238	0.3216238	-0.449623312	0.031609
FAM35A	-0.161875794	0.1111584	0.165245199	0.0985685	0.215270061	0.031587

OIT3	-1.409229093	0.1565634	0.359273847	0.7018765	-2.230893864	0.031547
PIH1D1	0.009709895	0.9428278	0.136562697	0.3097902	0.288805307	0.031506
P11-486G15	-0.841079172	0.1104532	-0.689316496	0.1656485	-1.171054104	0.031519
P11-823P9.	-1.166471471	0.0752756	-0.280765594	0.6536034	-1.417448078	0.031766
P11-680G24	0.191523102	0.6308827	-0.273038948	0.5042687	-0.982838853	0.031794
RBCK1	0.068810165	0.6050911	-0.193685309	0.1461033	-0.287054135	0.031828
ELFN2	-0.227746481	0.7219919	-0.578494511	0.3636053	-1.390549973	0.03185
BICD2	-0.158788996	0.1392315	-0.188850098	0.0777725	-0.230085129	0.031876
FAM20B	-0.042007216	0.5080905	0.026403033	0.6747026	0.134938292	0.031903
CDH3	-0.322022727	0.0566752	0.126119682	0.4547327	-0.362588725	0.031924
PNP	0.075123135	0.5350381	-0.089372417	0.4601558	0.258945096	0.031957
ARHGAP24	0.288548825	0.1441622	-0.093486829	0.6396871	0.419903699	0.032108
PEX10	0.115659679	0.3591274	0.189493365	0.1275024	0.26546464	0.032113
MTSS1	0.306629132	0.2045421	0.220675882	0.3609538	0.514947884	0.032151
ETV4	-0.030114887	0.870603	0.051662836	0.7796038	-0.396937911	0.032212
RSRC1	0.049227664	0.659371	0.065635625	0.5532282	0.236732576	0.032205
TRAM2	0.08133013	0.4687659	-0.100961964	0.3677093	-0.241590659	0.032304
PIP4K2C	0.034450055	0.8329801	-0.20110338	0.219525	0.347764669	0.032322
IFT46	-0.181627754	0.1809699	-0.088823332	0.5086602	-0.291077993	0.032346
SIRT1	0.242665952	0.0627363	0.122951114	0.3442001	0.277789889	0.032454
BUD31	0.009906287	0.9333363	0.085654892	0.4668941	0.251216873	0.032473
BFSP1	-0.222474769	0.3553831	-0.216718408	0.3619233	-0.517449192	0.032541
TBX20	-1.555501564	0.1266632	-1.707017153	0.0909683	-2.284218915	0.032539
RASSF7	-0.012929428	0.9781704	-0.134969189	0.7742732	-1.018055457	0.032573
FABP4	0.434803406	0.4711742	0.963797513	0.0997026	1.239737878	0.032603
SUPT20H	-0.194492874	0.069909	-0.023640532	0.8246319	-0.229173377	0.03263
HAUS8	-0.289464047	0.1288132	-0.304969662	0.1087897	-0.407740096	0.032651
P1-296G17	-0.62195767	0.3334514	-0.397468217	0.5203975	-1.493783789	0.032675
ARHGAP26	0.349229751	0.079595	0.327291983	0.0997129	0.424484092	0.032717
AFAP1	0.252262924	0.0935205	0.132691627	0.3766701	0.320616383	0.032761
CD2AP	0.090470694	0.4606416	-0.118832041	0.332428	0.261504614	0.032752
ZNRF2	-0.066851923	0.7252529	0.024974802	0.89532	0.403595163	0.032749
NRBP1	-0.085923539	0.3846504	-0.132309435	0.1799458	-0.210857323	0.032785
ISCA2	-0.068693551	0.6484727	-0.193831137	0.1931006	-0.322319366	0.032795
MTFMT	0.260619007	0.0577861	0.196197066	0.1473369	0.290441375	0.032825
ZNF829	-0.184393681	0.3896397	-0.256397723	0.2285315	-0.45906182	0.032834
CFI	-0.002988465	0.9913086	0.057702447	0.8332903	0.584201435	0.032949
SCN9A	0.027641637	0.8990934	-0.274470169	0.208751	0.462701802	0.033085
ZNF318	-0.222118642	0.1383656	-0.159904396	0.28391	-0.319282685	0.033136
TMPRSS4	0.313878274	0.153083	-0.004798731	0.9825764	0.467756438	0.033165
RPL4P6	0.532973173	0.2346391	0.403020073	0.3654452	0.925683883	0.033229
SYVN1	0.099068477	0.6093571	-0.331185399	0.0889302	-0.415301714	0.033257
P11-210K20	0.588098933	0.2594854	0.595559836	0.2480738	-1.282251801	0.033343
TMX3	-0.168397233	0.1622317	0.131036564	0.2741267	-0.255703197	0.033419
DLK1	-0.275778808	0.631539	-0.800421962	0.1640705	-1.224094348	0.033452
ORC5	-0.219542759	0.1072516	-0.178541444	0.1814185	-0.287462492	0.033449
PPP1R37	-0.166096455	0.2599171	-0.136141879	0.3547176	-0.313453489	0.033511

CA4	-0.251984273	0.7868391	1.05905885	0.2520025	-2.024646197	0.033573
EIF3D	0.130984363	0.1913597	0.13173919	0.1879362	0.212633217	0.033574
YPEL5	0.193084517	0.1060493	0.059727161	0.6163541	0.253077391	0.033601
ZNF623	-0.171716007	0.3063618	-0.303592394	0.0697643	-0.357078336	0.033695
SLC46A3	-0.577505055	0.1075785	0.355193918	0.3068303	-0.768808504	0.033722
UBE2B	0.171255739	0.1874458	0.176813461	0.1699334	0.27378581	0.033754
SYMPK	0.046477194	0.7473988	0.260497749	0.0697446	0.30565082	0.033801
SS18L1	-0.208550551	0.1977699	-0.306239331	0.0579089	-0.343314638	0.033843
DDX10	-0.098667207	0.5932577	0.310997049	0.0896736	0.389541889	0.03391
PGAP3	-0.08781414	0.592862	-0.181492272	0.2648621	0.340503434	0.033962
CLTC	0.104733864	0.1224298	0.042606091	0.5294651	0.143559431	0.034055
ACYP1	-0.238799528	0.3171968	-0.459122753	0.0538867	-0.509931428	0.034084
C21orf62	-0.29457068	0.4862038	-0.442575744	0.2899005	-0.926358757	0.034084
PRR5	-0.149242465	0.3802075	-0.278803928	0.0976016	-0.360240951	0.034112
QRSL1P3	0.237532789	0.5665368	-0.274768716	0.5176387	-0.98828588	0.034175
NLRP2	-0.417043698	0.2081366	-0.014919591	0.9639219	-0.708959021	0.034243
FAM171B	0.484622414	0.2436324	0.38076761	0.3593003	0.877758474	0.034291
VTN	0.02655693	0.9710271	0.847812832	0.2458507	-1.551931095	0.034294
MLLT3	-0.112981098	0.692152	0.027576647	0.9227583	-0.605479621	0.034363
CD302	0.565588113	0.1210958	0.214069623	0.5575872	0.770463376	0.034439
DNM1P51	0.177931187	0.6799855	-0.201742682	0.6400419	-0.921349249	0.034422
TMEM234	0.140304138	0.4644553	0.14542789	0.4434214	0.401665162	0.034427
UBA6	0.056125272	0.5242472	0.072427694	0.4099657	0.185866794	0.034436
CBY1	-0.25691739	0.4135296	-0.422293673	0.1778512	-0.668741161	0.034522
TECR	0.103823811	0.4351093	-0.091094723	0.4933426	0.28044122	0.034547
KCTD12	0.23031345	0.2722759	-0.141698715	0.4994967	-0.443812032	0.034577
MEF2C	0.027986629	0.9322831	0.004652376	0.9887052	-0.69888559	0.034624
PLIN4	0.152398835	0.5725437	-0.515596965	0.0611074	-0.586988098	0.034616
PNRC2	0.110656729	0.1098668	0.072470714	0.2938824	0.145910005	0.034617
PRKAR2A	-0.09923314	0.4227324	0.169795668	0.1690493	0.260726911	0.034634
RBM12	0.053813493	0.7440017	0.321197213	0.0510565	0.347789698	0.034669
P4-539M6.1	0.350792256	0.4540718	0.832491687	0.0736626	0.982366876	0.034709
TMEM177	-0.271801844	0.133839	-0.100930557	0.5693711	-0.381693145	0.034719
CRYAB	-1.038195019	0.1083336	-0.706376478	0.2590718	-1.377836684	0.034789
NTHL1	-0.078414437	0.6745409	-0.29756814	0.1089855	-0.398674763	0.034785
WFDC2	0.269008645	0.3407759	-0.009948402	0.9719124	0.594516361	0.034849
RAB37	-0.36882774	0.3146721	-0.234168076	0.5203721	-0.777640618	0.034882
ZNF512	-0.026589607	0.834575	-0.149948188	0.2368597	-0.269110122	0.035005
BTN3A1	-0.124851479	0.4509983	-0.143737192	0.3829008	-0.349814358	0.035033
DDHD1	0.216627021	0.3022639	0.243762898	0.2463162	0.440991246	0.035036
SYPL1	-0.094755513	0.3312687	-0.136555556	0.1596493	0.203663052	0.035174
MDP1	-0.620608325	0.1740164	-0.391376325	0.3877653	-0.964693958	0.035238
RPS29	0.152632617	0.2915988	0.222614543	0.1236107	0.304498528	0.035226
AGAP4	0.158426938	0.6794592	-0.671254857	0.0829056	-0.809351793	0.035287
SLC22A3	-0.220598123	0.6187185	-0.111776694	0.799834	-0.94475624	0.035304
MTPAP	0.154927575	0.199911	0.118449847	0.3246719	0.253313381	0.035349
DPF2	0.030258921	0.8005652	-0.030791448	0.7960961	-0.252879007	0.035455

MEF2A	0.103046154	0.3566655	0.081914004	0.4632923	0.234620044	0.035428
P11-380M21	1.650667619	0.1292902	1.504164639	0.1657927	2.250106853	0.035452
RPL23AP7	-0.094904222	0.6225238	-0.256016681	0.1810701	-0.403625685	0.03542
STARD4	0.276804507	0.0669384	0.054604676	0.7177184	0.31749336	0.035453
ST3GAL6	-0.649566273	0.0530085	0.053811841	0.870569	-0.709288977	0.035474
EMG1	-0.164914635	0.2193885	0.165108486	0.2149619	0.27999413	0.03554
ANKRD11	0.172091425	0.1196896	0.092235156	0.4029189	0.232048313	0.035554
MIEF2	-0.350981455	0.0939584	-0.212180989	0.3066746	-0.445022867	0.035597
PPFIA2	0.386750375	0.3275412	0.088785127	0.8264143	0.832215346	0.035606
PYCARD	0.07562077	0.7601973	-0.043549654	0.8589229	0.501885721	0.035638
ROPN1B	-1.584698523	0.1011804	-0.164253132	0.8451303	-2.11110532	0.035655
SLITRK3	0.28837116	0.5225304	0.314063096	0.4753309	-1.010590073	0.035619
NIPSNAP1	-0.169135581	0.1353815	-0.071608653	0.5256667	-0.237655975	0.035694
PLEKHG4B	-0.483956939	0.3039112	0.273921279	0.5540311	-0.989541354	0.035707
ZBTB45P1	-0.691357043	0.3220183	-1.096111949	0.117841	-1.493719488	0.035717
RPL13AP3	0.282973572	0.6945725	0.810450415	0.2221132	1.36518999	0.035729
DEPDC5	0.170829214	0.4188603	0.169459357	0.4203417	-0.444804486	0.035811
LIN28B	-0.363027279	0.2830653	0.003078556	0.992725	-0.710277804	0.035837
NOC3L	0.089215997	0.3776595	0.126829787	0.2064337	0.210403191	0.035854
CASR	0.64756544	0.2745204	0.601033794	0.3036501	1.21968755	0.035936
COL21A1	0.601730773	0.4819616	-0.604793998	0.4807914	1.791772802	0.035924
GRIA4	-0.261080267	0.4728363	0.49309227	0.1729073	-0.765754281	0.035967
CD9	0.035496104	0.895699	0.30963707	0.2517947	0.565911228	0.036118
CRIP1	0.033541589	0.9139747	-0.269465215	0.3874664	0.638013835	0.03613
SPTBN4	0.390609348	0.6373834	0.887618553	0.2669789	-1.890665497	0.036244
AC079753.4	0.596266187	0.3872015	0.986897538	0.1410828	1.389923382	0.03626
TOMM6	0.129796618	0.2473779	0.170099985	0.1276749	0.233844321	0.036342
GPA33	-0.301896686	0.6465618	-0.28199843	0.6683089	-1.379191552	0.036446
SLC6A20	0.013443983	0.9804046	0.398323012	0.4633655	-1.164306278	0.036437
MYL1	-0.802235803	0.7476386	-3.913717249	0.1380275	-5.585347266	0.03648
IFI44L	0.547115951	0.1723557	0.121327261	0.7635298	0.836167461	0.03652
SBK1	0.058183768	0.8040992	-0.28704315	0.2240488	-0.504222985	0.036554
CTNND1	0.111216634	0.2421632	-0.075847615	0.4253573	0.198687653	0.036584
LAMP5	1.125444854	0.2240259	-0.102449501	0.9128897	1.925175529	0.036602
SSBP3	0.195179044	0.234468	0.251208303	0.1250674	0.341528375	0.036597
CPT1B	-0.155057154	0.4602326	-0.398907501	0.0576352	-0.438210904	0.036667
GKAP1	-0.157995349	0.4673365	-0.317205514	0.1322417	-0.458131566	0.036679
DPYD	0.399216054	0.0820749	0.364893894	0.1109098	0.478137339	0.036735
VIPR1	-0.545755869	0.313601	0.24369844	0.6507464	-1.139649108	0.036728
HES4	0.430378407	0.2664451	0.532341795	0.1589972	0.784624985	0.03677
SH3GL2	-0.161476817	0.7617878	-0.574876013	0.2853831	-1.184333195	0.036783
PRDX5	0.076132657	0.5046215	-0.00230382	0.9838578	0.237339883	0.036805
ZNF142	0.117010409	0.560455	-0.119349397	0.5527174	-0.420574226	0.036904
CAMK2N1	-0.329053213	0.2560954	-0.108265981	0.7079991	-0.605240424	0.03693
PCP4L1	-0.698979147	0.2191106	0.306996716	0.567308	-1.219841019	0.037015
SLC39A11	-0.0730609	0.4950457	-0.084584405	0.4275922	0.21907807	0.037033
HSPA9	0.135107523	0.3834572	0.240353238	0.12084	0.323042522	0.037082

POLA2	0.223723236	0.1897174	0.023780906	0.888702	0.353230134	0.037108
TTC18	-0.39661255	0.1741141	-0.279534233	0.3251902	-0.60559235	0.037112
FBXO11	0.046011092	0.6598441	0.13021717	0.210584	0.216700254	0.037162
KIAA1467	0.249283583	0.2272145	-0.006757387	0.9740039	-0.447139481	0.037236
ADCK1	-0.281951502	0.1892723	0.195247294	0.3425469	-0.44045902	0.037268
ANO10	0.065965182	0.6705847	0.053993264	0.7258776	0.320050883	0.037356
CIR1	-0.188525307	0.118772	-0.147702431	0.2179694	-0.250612937	0.037366
LAT	0.046295776	0.8789331	-0.327457372	0.2812631	-0.648799436	0.037364
SRPX2	0.164912128	0.5586321	0.341913732	0.2229574	0.583197143	0.03736
NR3C1	0.265882289	0.1944537	-0.045305487	0.8249675	0.42555954	0.037419
SLC3A1	-0.227176805	0.5519925	0.421453908	0.2660889	-0.799397005	0.037418
TGFBR1	0.194033172	0.142324	0.220675247	0.0941768	0.274641325	0.037424
HNF4A	-0.148434606	0.6307018	-0.244498304	0.4277335	-0.644983622	0.037442
SPOCK2	-0.4347813	0.5092854	-0.332393328	0.6137959	-1.371607648	0.037468
B4GALNT3	0.180000482	0.4301747	-0.099520776	0.6633264	0.471921355	0.037569
BEGAIN	0.860391735	0.1750797	0.848753221	0.1770744	1.310866454	0.037555
GPATCH3	-0.302605791	0.2046665	-0.29834971	0.2079307	-0.498490706	0.03757
PLAC9	0.041143462	0.9276955	-0.112298531	0.803973	0.905417766	0.037593
RPSAP9	-0.219461516	0.8198722	-1.008584032	0.3061983	-2.257201133	0.037657
P11-122G18	0.946524628	0.53922	2.348942052	0.1173522	3.10216479	0.037686
RP11-454L1.	-0.458412976	0.3341831	-0.389308463	0.4005796	-1.028971672	0.037696
SHBG	0.297663018	0.6283078	1.081536928	0.0695497	-1.544642055	0.037785
TRIM23	-0.386846414	0.0699202	-0.084070623	0.6908998	-0.441979232	0.037872
ZNF691	-0.203327458	0.3833347	-0.269547478	0.2448033	-0.488246446	0.037865
POLR2B	-0.081183602	0.3247358	-0.002805612	0.9727621	0.170514229	0.037906
CSRP1	0.253557133	0.1541652	0.207460546	0.2434493	0.368868427	0.038054
SLC25A1P5	-0.302630148	0.4745434	-0.584787363	0.1698024	-0.947197456	0.03808
GNL3	0.101128592	0.3993054	0.04070957	0.7341437	0.248284057	0.038181
WDHD1	-0.274511231	0.0815019	-0.232100408	0.1389574	-0.326257631	0.038254
SLC35B3	0.150856298	0.1787828	0.037383267	0.7373733	0.229866484	0.038314
P11-356M20	-0.190002612	0.7534537	-0.436230507	0.4711685	-1.400556391	0.038345
HERC2P9	-0.336125955	0.1144744	-0.155446789	0.463416	-0.440244099	0.038418
EHBP1L1	0.291049934	0.0767772	0.118773125	0.468613	0.339038209	0.038465
CLIP3	0.408489463	0.2008006	0.020085692	0.9499982	-0.676730009	0.038596
EIF2B5	-0.187849758	0.1258676	-0.132450801	0.2790404	-0.253211503	0.038598
NUTM2D	0.097041902	0.6782891	-0.248068801	0.2904609	-0.491307463	0.038636
ELOVL4	-0.019733662	0.9393036	-0.027842963	0.9138225	-0.54319967	0.038852
ST6GAL1	-0.123814307	0.7045905	0.367639642	0.2570931	-0.679262176	0.03885
BAZ2A	0.059329106	0.7195439	0.027270427	0.8689642	-0.341957898	0.038866
ALG5	-0.177671874	0.0924094	-0.003291367	0.9747471	0.21383174	0.03896
MRPS18A	0.181757613	0.1226247	0.030416947	0.7957909	0.241889355	0.039054
NUDT4	-0.203195168	0.0757685	-0.202496659	0.0763293	-0.235965846	0.039041
TFPI	0.156246878	0.2869969	0.132100121	0.3677678	0.302704597	0.03904
NEURL1	-0.315747392	0.3300616	0.094607748	0.7688503	-0.682524528	0.039212
SULT1A1	-0.256012781	0.5261999	-0.026938664	0.9465232	-0.835035578	0.039216
CCDC144NI	-0.049859867	0.9167937	-0.186611637	0.689594	-1.022023714	0.039253
CTPS1	0.058695884	0.7358684	0.162650025	0.3487204	0.357283299	0.03929

SERPINB2	-0.193262873	0.8053326	0.78507761	0.3134371	1.602693761	0.039279
SEL1L3	0.107495817	0.5641875	0.07473289	0.688411	0.38375867	0.039338
GNAI3	0.06714697	0.2700013	0.084890144	0.1610207	0.124836896	0.039489
CDH17	-0.595750022	0.2851053	-0.17927015	0.7477008	-1.147394961	0.039537
CPA6	0.168904739	0.779276	1.018618942	0.0702918	1.151455979	0.039609
RLIM	0.030219922	0.6829277	0.060014813	0.4154186	0.151727201	0.039603
CEP63	0.019826361	0.9015512	0.022307931	0.8891112	0.327988271	0.039647
IFT27	-0.360918889	0.0800983	-0.249758298	0.2225701	-0.424407653	0.039692
UEVLD	-0.034699489	0.7391768	0.152711253	0.1379298	0.211762825	0.039699
P11-480I12	-0.303386372	0.5466408	-0.334851925	0.4983548	-1.111956176	0.039733
GPR125	0.094283619	0.4295704	0.215136936	0.0704553	0.244618733	0.039874
EMBP1	-0.19472703	0.7580134	0.809943884	0.1690892	1.197772696	0.039923
LAD1	0.146998142	0.3714698	0.144980914	0.3780307	0.337720921	0.03993
C12orf5	-0.358041154	0.0843701	-0.39162844	0.0585641	-0.426894441	0.039953
P11-175I17	-0.290533398	0.0647296	0.066940489	0.6600967	-0.32027568	0.040054
ERP27	-0.380970346	0.2881101	0.144993287	0.6835541	-0.739220585	0.040092
TAS2R64P	-0.226137905	0.54902	-0.207888602	0.5730929	-0.798875558	0.040117
MICA	-0.028018185	0.8477769	0.173951326	0.2276695	0.296082731	0.040132
ACTN2	-0.401506139	0.6057871	0.234239789	0.7570612	-1.662161485	0.040178
CLEC3B	1.1317232	0.2707821	-0.005344487	0.9959416	2.071928415	0.04025
TTC38	-0.263573788	0.1034255	-0.018254103	0.9096673	-0.332567396	0.04025
FKBP7	-0.041986592	0.8241791	0.014486449	0.9385049	-0.388858181	0.040278
ECM2	0.1074358	0.8260557	0.721446754	0.1274148	0.968451758	0.040379
SLC35G2	0.571648167	0.1289032	0.18801728	0.6229966	0.751276706	0.040361
SMYD5	-0.044527385	0.8219069	-0.26485851	0.1792758	-0.405522387	0.040389
YAP1	0.079017236	0.3418499	0.145613975	0.07851	-0.170263932	0.040493
MGAT4A	-0.306468813	0.286257	-0.032339605	0.9102667	-0.589410675	0.040514
NEURL4	-0.184059961	0.297985	-0.336820684	0.055521	-0.361948619	0.040532
FAM109B	0.193590341	0.6548351	-0.281901735	0.5226831	0.846796061	0.040584
P11-345J13	-0.950367719	0.08548	-0.312135552	0.5422518	-1.137855347	0.040583
HOXB4	0.112221456	0.6862492	0.148563442	0.5894024	-0.582510852	0.040636
SIK3	0.11427406	0.4674124	-0.175948851	0.2627027	-0.322592839	0.040667
PBXIP1	-0.121848552	0.4694133	-0.209632015	0.2126439	-0.344946391	0.040686
SYNCRIP	-0.012523624	0.91312	0.113721267	0.3212003	0.234494246	0.040758
EPB41L5	0.235355624	0.0948833	0.051176377	0.7163333	0.287883919	0.040783
ATP5L	0.181774253	0.0667155	0.031299112	0.7517412	0.202213998	0.040905
LRRC3B	1.016046046	0.1943117	1.072415875	0.1679789	1.562138826	0.040943
SRBD1	-0.146011822	0.2052425	0.123287333	0.2749701	0.231439952	0.040963
CDHR2	0.00768565	0.9923263	0.651175685	0.4146587	-1.646047372	0.041053
FANCC	-0.168597737	0.4011693	-0.193918289	0.3308598	-0.40909734	0.041051
GALNT12	-0.078725818	0.6560925	-0.130974268	0.4577699	0.359061727	0.041002
PUM1	0.114978728	0.0633553	-0.009469645	0.877939	0.126052039	0.041043
TRMT1L	0.10269117	0.5799881	0.018092019	0.921674	-0.380121126	0.041016
FRMPD1	0.093308131	0.9055092	0.419225198	0.5865536	-1.651798182	0.041228
UBE2E1	0.036578827	0.7233427	0.02104225	0.8381335	0.210137981	0.041234
COX7B	-0.060903954	0.5552035	0.150924602	0.1415596	0.209598621	0.041337
C20orf144	0.287540561	0.4340025	0.230018418	0.5249135	-0.842381371	0.041532

PCDH10	0.056919849	0.87925	0.309939325	0.4067873	-0.770709299	0.041524
SHH	0.243912553	0.1370042	0.024057327	0.8833004	0.333500261	0.041552
C7orf50	0.104015257	0.5636893	0.142998658	0.4227329	0.363592218	0.041667
GTF2F1	-0.143882452	0.1972445	-0.069768475	0.5308392	-0.227075523	0.041666
IMPAD1	0.145525793	0.389692	0.078162486	0.6440364	0.344446085	0.041702
BDNF	0.156787515	0.6173567	-0.380176647	0.2277207	-0.646354933	0.041808
FAM149A	0.042001099	0.8244799	-0.223105756	0.2376824	0.379626009	0.041869
MUS81	-0.232244761	0.0729894	-0.124218612	0.3349656	-0.263625817	0.041876
SOS2	0.077131315	0.4345211	0.074690857	0.4467235	0.199715167	0.041896
GNRH2	-0.946066802	0.2525185	-1.375352274	0.1039044	-1.766391788	0.041925
TFIP11	-0.061865193	0.6682907	-0.154479259	0.2842285	-0.293486599	0.04192
DGKI	0.277053383	0.510043	-0.059206482	0.8880193	-0.863804432	0.041951
TACC1	-0.318726844	0.0833261	-0.284395839	0.1219953	-0.374378959	0.041953
ARID3A	-0.121463324	0.5627113	-0.318620056	0.1288636	-0.426875369	0.041983
PPT2	-0.314672064	0.0829542	-0.287483311	0.1136803	-0.372839656	0.042038
ALOX15B	0.029137872	0.9491727	-0.365775133	0.4263336	-0.9607663	0.042066
MTCH2	0.028280641	0.8463814	0.170748536	0.2401249	0.295438628	0.04224
USP40	-0.260313194	0.0697551	-0.154777814	0.279725	-0.291125099	0.042269
MTHFS	-0.114690077	0.5438157	-0.338321925	0.0731476	0.377624874	0.042355
SEPP1	-0.310484175	0.4994097	0.82545639	0.0723761	-0.933617203	0.042351
HDAC6	-0.121091717	0.2758183	-0.145294855	0.1901551	-0.225436114	0.042386
PLN	-0.390973603	0.2694595	-0.416375233	0.2337816	-0.727537957	0.042494
CHMP4A	-0.24253439	0.1721691	-0.196602551	0.2641487	-0.359876373	0.042515
DNAJC2	0.072702943	0.5080529	0.162847646	0.1358651	0.221685444	0.042522
ATP13A1	-0.036224017	0.8147507	-0.163426223	0.2899908	-0.313315126	0.042806
TMEM139	0.385646308	0.0749214	-0.079200537	0.7165328	-0.445566155	0.042804
GJB4	0.771055947	0.3421984	0.992771854	0.2132165	1.591630642	0.042933
ISOC1	0.078125775	0.413799	-0.128817533	0.1773999	0.192133338	0.042921
POMT1	-0.060376921	0.7559284	-0.270918	0.1613923	-0.3939971	0.042909
ENTHD2	0.183201611	0.4030987	-0.295239692	0.1789425	-0.450941897	0.04296
GEN1	-0.137228914	0.4443922	-0.289535979	0.1055299	-0.362417617	0.042983
NR1D1	-0.718906461	0.1685016	-1.001727337	0.0552643	-1.061727595	0.043018
RAB35	0.238155478	0.0519714	0.148519468	0.2264918	0.24755211	0.043027
LEMD3	-0.196039966	0.1402974	-0.099445886	0.4533553	-0.268875521	0.043053
ARSD	0.120248364	0.3213308	0.058187206	0.6306439	0.244219847	0.043086
ACO1	0.113696828	0.2765726	0.01409215	0.8925384	0.21065729	0.043209
HECTD1	-0.133361882	0.1083013	0.141120959	0.0878835	0.167175378	0.043191
SLC2A8	-0.161805867	0.4377126	-0.394333077	0.0576274	-0.420195853	0.04325
AMPD2	0.131270051	0.2906293	-0.110381301	0.3736782	-0.251449433	0.043462
IARS	0.037849944	0.6902418	0.074214644	0.4336953	0.191364161	0.04349
COQ10B	0.26609177	0.0720873	0.097862737	0.5082377	0.297767492	0.04368
ZNF793	-0.226981752	0.1473218	-0.156165092	0.3131989	-0.316192282	0.043694
IMPACT	0.038209466	0.7596034	0.235813871	0.0568283	0.250139835	0.043743
UGT2B17	0.025381678	0.9763374	0.988369831	0.2446476	-1.77242293	0.043731
ACSBG2	-0.328303872	0.6930832	0.335748589	0.659668	-1.839795908	0.043854
TMPRSS11E	-1.003993968	0.466967	1.798496117	0.1319764	2.407085513	0.043886
SENP3	0.136045889	0.2773321	0.212542929	0.0876129	0.251053747	0.043911

PTMA	0.061426533	0.5573088	0.153111643	0.1433956	0.21080641	0.043948
TBCK	-0.0374924	0.8262648	-0.072884724	0.6687393	-0.344598174	0.043952
REPS2	0.297546814	0.2248787	-0.076375396	0.7558525	0.491693534	0.043983
C1D	0.218853992	0.2967924	0.360846309	0.0842122	0.421026987	0.044051
RLTPR	-0.367457395	0.4022524	0.055387871	0.8993115	-0.94107277	0.044065
ACTR3	0.064984523	0.4585657	0.059995011	0.4930182	0.176156468	0.044104
ATP5C1	0.040799923	0.7366051	0.186413389	0.123534	0.243660484	0.044126
ITGB1	0.043664397	0.7204237	0.191272207	0.1167834	0.245383786	0.04421
CYP39A1	-0.414827102	0.1531079	0.479462153	0.0845667	-0.588577976	0.044554
ADAMTS8	0.299882183	0.5156618	0.166241275	0.7182509	0.916573159	0.044663
ANXA9	-0.150769613	0.5970298	-0.151956248	0.5919355	-0.577332068	0.044705
LRRC37A2	-0.053737552	0.6146061	-0.016084926	0.8778591	-0.214727607	0.044766
TMEM116	0.090434655	0.5745515	-0.076382842	0.6320708	-0.32745356	0.044816
ZDHHC14	-0.19253935	0.329563	0.089357648	0.6448618	0.387405125	0.044822
GALE	0.201521119	0.1335029	-0.196214123	0.1447364	0.268708693	0.044872
HINT1	0.087971687	0.2892876	0.103112552	0.2126408	0.166013129	0.04492
C1QTNF2	-0.033335961	0.9294515	0.585117409	0.0945026	0.70046914	0.04506
EIF3M	-0.032842607	0.7433832	0.169226059	0.0907555	0.200610638	0.045026
NRIP2	-0.03545089	0.8941737	0.129030323	0.6218184	-0.546972098	0.045054
RP11-2J18.[illegible]	-1.100899614	0.0930878	-0.767074438	0.2052081	-1.33512876	0.045011
C18orf21	-0.116832929	0.4064854	-0.180937133	0.1968827	-0.283123445	0.045074
POLR3GL	-0.201175497	0.139198	-0.23051707	0.086958	-0.27097488	0.045088
ITIH4	-0.385033563	0.4010646	-0.455223923	0.3212864	-0.949191199	0.045128
DNAH1	-0.257532824	0.3608976	-0.081437534	0.7726168	-0.566478997	0.045273
MOCS3	0.018330791	0.8582045	-0.076931599	0.4483926	-0.206940084	0.045235
SIX4	0.011798652	0.9659892	-0.079456959	0.7736105	0.550824252	0.045261
SPTLC3	-0.372980954	0.1594186	-0.27248834	0.3013143	-0.530030119	0.045386
C20orf24	-0.001444949	0.9911943	0.127511333	0.3261082	0.259544877	0.045424
PCDHGB6	0.14793906	0.4369504	0.088838963	0.6392299	-0.384835042	0.045488
ZBTB12	-0.378531763	0.27644	-0.514595345	0.1374931	-0.700521483	0.045554
PDK3	0.164972258	0.2856292	-0.101816097	0.5102934	-0.312295263	0.045623
PDZK1	-0.546829726	0.2993625	-0.108100515	0.8368489	-1.05365902	0.045752
GAREML	0.548148627	0.1531095	0.718357951	0.0604307	0.767213687	0.045888
LEF1	0.183338149	0.753688	0.460289147	0.4295592	1.15761679	0.045869
PHF11	0.055027957	0.8069829	0.34511142	0.1204637	0.443057749	0.045869
ZNF284	-0.158823742	0.2902231	-0.124023482	0.3970222	-0.299887331	0.045911
ASS1	0.526647984	0.1267976	0.330454099	0.3379057	0.686966625	0.045994
P2RY1	0.090376397	0.6811863	-0.196513598	0.3718074	-0.440017161	0.046025
CTR9	-0.12243049	0.2918955	-0.072292221	0.5336414	-0.232307971	0.04615
ATP6AP2	0.058780908	0.5626756	0.052150422	0.6065276	0.20146879	0.046422
SUSD1	0.20018479	0.0752983	0.112512807	0.3164506	0.223415584	0.046453
TYMS	-0.035462127	0.8372922	-0.192229559	0.2648412	-0.344111703	0.046429
SELT	-0.005923975	0.9670281	0.186615079	0.1916698	0.284533914	0.046547
PPP4R1L	-0.02139331	0.9279967	0.372778416	0.110662	0.464880961	0.046611
WDR43	0.114887026	0.5101666	0.144996371	0.4056191	0.3468991	0.046606
NUDT16P1	-0.360927187	0.1829849	-0.236261729	0.3670722	-0.539916911	0.046696
RARA	-0.269287189	0.0902011	-0.172204595	0.2777884	-0.316182382	0.046685

PPIF	-0.002318697	0.9881265	0.088457743	0.5689898	0.30836011	0.04677
FAM63A	-0.181493745	0.4123855	-0.283091155	0.1997344	-0.440226495	0.046873
RPS3AP47	0.475756437	0.4650865	0.152167053	0.8169014	1.20673403	0.046881
CASP2	-0.012478089	0.9437253	-0.292860216	0.0979878	-0.351927043	0.047048
H2AFJ	0.113021894	0.5297804	0.01286679	0.9428516	0.355475136	0.046934
PRADC1	0.086753171	0.6104787	0.128317607	0.4426747	0.330253192	0.047016
RASSF6	0.224387865	0.2092748	-0.022410566	0.9002159	0.354402394	0.047048
UNC13B	0.199625046	0.1689628	-0.088065467	0.5441075	0.28763545	0.047144
SERPINH1	-0.131277596	0.3386558	-0.143996776	0.2937436	-0.272317285	0.047193
EGLN1	0.012765312	0.9218642	0.214907834	0.096741	0.256927232	0.047211
TRRAP	-0.060941926	0.734789	-0.262425533	0.1444696	-0.356950584	0.047386
DYNLL2	-0.119299352	0.2814889	-0.141841026	0.1986167	-0.219380393	0.047469
PBX2	0.077725701	0.334819	-0.145177963	0.071221	-0.15984814	0.047516
C1orf63	-0.240329482	0.1174737	-0.130423312	0.3938024	-0.304483713	0.047558
ERGIC3	0.063688132	0.6144357	0.075771613	0.5485314	0.250121177	0.047598
MAMDC2	-0.31168797	0.6054594	0.710775938	0.2086221	1.111985682	0.047571
RP11-203L2.	0.668721737	0.3229089	1.009798753	0.1185122	1.26856535	0.047579
SLC4A4	0.065405723	0.7289866	0.192548715	0.3072356	0.373479912	0.047588
CCDC127	-0.262165125	0.1168252	-0.129463342	0.4348269	-0.329978536	0.047712
PIGT	0.291846279	0.0585735	0.205306997	0.1829635	0.305214814	0.047731
ERMARD	-0.286661823	0.1747273	0.066354898	0.7465002	-0.415165199	0.047777
WDR37	-0.125230656	0.3241994	-0.225574873	0.0762739	-0.252734023	0.04778
PRKG2	-0.345516154	0.3006017	-0.192383266	0.5590643	-0.667767403	0.047806
RP9	-0.130890562	0.3004258	-0.127663576	0.3041976	-0.249985912	0.047859
RDH5	-0.446236487	0.1248519	0.003456795	0.9903809	-0.579344951	0.0479
B4GALT2	0.077301073	0.4442635	0.175854376	0.0791041	0.198321336	0.048095
GPN3	-0.210581038	0.2391604	-0.084895441	0.6297247	-0.351130615	0.048239
PCK1	0.335104771	0.6237547	0.953384673	0.1590095	-1.396799995	0.04822
TSTA3	0.099474156	0.4618101	0.052783289	0.6949708	0.265819935	0.048233
CHST12	-0.178470364	0.2950317	0.009625704	0.9542309	-0.336854794	0.048271
DKK3	-0.13715724	0.5090277	0.079639165	0.6970658	0.402696892	0.04838
HSBP1	0.006225025	0.9366831	0.014762919	0.8497886	0.153900407	0.048358
PEX5	-0.140720113	0.3271159	-0.148980911	0.2972634	-0.283713738	0.048397
SLIT3	0.587473076	0.0831145	0.564830173	0.0951688	0.668967754	0.048386
SYT7	-0.138353129	0.6839308	-0.496072805	0.1449499	-0.678875454	0.048366
VPS26A	0.201738772	0.0939427	0.127245168	0.2893395	0.23694303	0.048315
DUOX2	0.764644851	0.1052272	0.778708889	0.0989285	0.930538412	0.048639
KCNG3	0.819039685	0.3516148	1.030294659	0.2299992	1.656663131	0.048646
P11-174O3	-0.59461018	0.1906198	0.203249473	0.6135998	-0.922178149	0.048696
CAPN15	-0.16986763	0.2237752	-0.178768277	0.2008913	-0.275346325	0.048769
EPDR1	0.414123826	0.5265379	1.176910827	0.0588386	1.208199005	0.048793
CCDC41	0.080614945	0.631554	0.25370557	0.1259283	0.327504267	0.048834
8-Sep	0.098931451	0.3204392	0.092106104	0.3524895	0.195118227	0.048919
ME2	0.095172806	0.5510633	0.303963151	0.0560793	0.313472338	0.049015
PLXNB1	-0.218726098	0.0807099	-0.167867832	0.1798456	-0.246483311	0.049047
TUBB2BP1	2.16850546	0.0807732	1.98167448	0.1104948	2.4414528	0.049099
MTND2P12	0.141636254	0.8307397	0.026418729	0.9679297	-1.537797998	0.04913

MED6	0.021514522	0.7992263	-0.140033232	0.0976368	-0.166836787	0.049263
CYP2C9	0.076003553	0.7573461	0.395834478	0.1049402	0.479488943	0.049358
FBXO7	0.095353723	0.1675024	-0.005659563	0.9343524	0.134945709	0.049489
HSPA14	0.115885524	0.2316872	0.053187675	0.5811182	0.189092277	0.049497
RNF216P1	-0.077791503	0.5295401	-0.003324428	0.9784058	-0.243798348	0.049482
ZFYVE20	-0.144676687	0.1816812	-0.0613639	0.5680837	-0.212451027	0.049531
GBP5	0.338785375	0.2669852	0.409482593	0.1769267	0.594149156	0.049599
ZNF425	-0.35758142	0.243685	-0.328430493	0.2743838	-0.599308445	0.049601
HPGD	-0.279332137	0.4441181	-0.153791965	0.6734068	0.715595662	0.049729
CEP78	-0.186046241	0.1870624	-0.053706101	0.7017613	-0.276013819	0.049816
EPHA7	-0.089094745	0.7424852	-0.163620213	0.5457845	-0.532813151	0.049808
VWC2	1.108997092	0.1808731	1.042185215	0.207618	1.614640562	0.049813
COLEC10	-0.491425819	0.2388836	-0.526282951	0.206815	-0.819129917	0.049844
RAB11FIP3	-0.045223208	0.8478283	-0.303380724	0.1968065	-0.46520166	0.049911
FAM83B	0.23534337	0.2239416	0.273863732	0.15617	0.378521721	0.049984
AC006195.2	2.630599148	0.0544227	2.626964424	0.051425	2.720182937	0.043755
AC019181.3	-1.047489868	0.2143014	-1.257532314	0.1346298	-1.990957686	0.039713
AC024937.4	-1.34866216	0.3429969	-1.74783522	0.2257685	-3.996347226	0.012611
AC026882.1	1.798042708	0.2778071	2.564983635	0.0890284	3.462185087	0.015789
ACTBP11	0.498830256	0.5958371	0.807580559	0.3635226	1.75217028	0.035951
ACTBP12	1.292616462	0.2338212	1.746167569	0.092673	2.183738724	0.032583
ACTBP8	1.353410605	0.3478072	2.323452027	0.0819172	2.791995359	0.033911
ARPP21	-1.964910291	0.1630456	-0.884344514	0.5155771	-3.850243547	0.014158
ASB15	-2.08476283	0.175676	-0.401471791	0.7520592	-3.435755948	0.027966
C11orf16	-0.907452794	0.3374286	-0.988787876	0.2843833	-2.665123708	0.031519
C11orf42	-0.46648207	0.4622801	-1.283062398	0.0619862	-1.493019293	0.038961
C11orf53	-0.706375942	0.4536617	-0.960250326	0.3090398	-3.186630496	0.014665
C20orf202	-0.212216921	0.7334995	-0.690484042	0.2799843	-1.447820291	0.043263
CCDC54	1.057519433	0.5572977	2.153559811	0.2050679	3.432783041	0.030163
CER1	-0.55931266	0.4618771	-0.216750731	0.7654185	-2.154411694	0.018945
CHRND	0.094477021	0.9355692	-1.175229681	0.3599567	-3.713856324	0.011241
CRISP3	1.326204647	0.2001418	1.591461401	0.1005763	2.343571132	0.018512
CTC-484M2.	1.563402266	0.182062	2.02553507	0.0706184	2.246061271	0.043681
CYCSP34	0.233994311	0.7729882	-0.448355075	0.5993059	-3.050366588	0.012905
CYLC2	-0.71095457	0.6437654	-0.594588648	0.6855216	-3.243345001	0.04752
CYP4F25P	-2.226425116	0.1136711	0.842517442	0.4247148	-3.08530344	0.028369
DDX50P2	-1.864035802	0.1004086	-0.922748509	0.3559604	-3.72984415	0.005576
ENO1P3	-0.74789452	0.4607914	-0.195168885	0.8370095	-3.378376276	0.010974
EPHA8	0.457475095	0.6330187	0.240996046	0.8009557	-3.032434186	0.014348
FCGR1A	-0.69773346	0.5306215	1.161142439	0.2125327	-2.704547142	0.042159
FCGR2C	-2.58900466	0.1131947	0.596141727	0.6616822	-3.235257495	0.048483
FKBP6	-0.382455114	0.6617622	-0.751665451	0.3881736	-3.315894327	0.00216
FRG2C	0.314122694	0.7239451	-0.075309726	0.9334954	-3.582969525	0.004915
GNAQP1	1.3577603	0.0947872	1.311562365	0.1028426	-2.700268379	0.032115
GS1-304P7.	2.546949569	0.0574192	1.719824948	0.2136347	3.135162754	0.016385
HBBP1	-1.474667137	0.1622211	-1.013478514	0.2991926	-2.917611453	0.025783
HDC	-2.061452189	0.1668889	-0.534959191	0.6735475	-3.001857175	0.044131

HMGB2P1	1.182365644	0.3005371	1.325804185	0.2329092	2.431227151	0.021573
HMGN1P30	0.336103057	0.8943979	1.577818015	0.5320495	4.72767798	0.043678
INSL4	0.790294125	0.5473045	2.134454378	0.0698629	2.61301177	0.024349
IP6K3	2.644152759	0.0589585	2.40526758	0.0858471	3.922819397	0.002966
ITLN1	0.851008098	0.6016193	1.36371452	0.3661808	2.888703503	0.044135
KLK8	1.839804912	0.2243491	0.769549152	0.6220104	2.963942884	0.046038
KRTAP5-1	1.057530092	0.5576083	2.845790779	0.0795062	3.234000196	0.043125
A16c-3G11.	0.890059746	0.4121165	1.935151496	0.0532408	2.349101765	0.017535
LDHAP1	-0.385161385	0.8215528	0.809229271	0.6339284	2.931127214	0.044788
LY6D	1.074325694	0.46828	-0.311087529	0.8506304	3.010430866	0.027865
MBL2	-0.767997189	0.6360215	0.215951195	0.8887365	-3.596420729	0.045546
MRPS18AP1	-0.923554511	0.3650433	-0.510212409	0.5882593	-3.093115888	0.01641
MTND6P5	2.291772614	0.1687893	1.557241413	0.3727696	4.245067448	0.004352
MYH4	-1.140229026	0.2456592	0.05930522	0.9476734	-2.123034292	0.04786
NDUFB1P1	-0.773244148	0.3801134	-1.253550872	0.1690357	-3.45116986	0.005156
OR4F28P	-2.936114538	0.103435	-1.074681374	0.5061527	-3.794041578	0.035357
OR52N2	-2.126333409	0.1437275	-1.629728961	0.2587456	-2.985211685	0.040111
OR5BA1P	0.833152268	0.3099075	1.388256186	0.0693646	1.787607216	0.01704
PCDH8	0.724737234	0.6086836	2.061898445	0.1078218	2.855100832	0.023669
PDE6G	-1.036053684	0.3160203	-1.604266803	0.1472077	-2.629969347	0.043062
PLK5	1.756391174	0.2012654	1.9452286	0.1343671	3.055122645	0.01537
PRAF2	-1.108282563	0.3214095	-1.339768102	0.2304634	-2.964543597	0.025845
QPCT	0.918299605	0.4634212	2.275699196	0.0516736	2.525385073	0.031199
RAP1AP	1.868402404	0.165345	2.436179469	0.0586489	2.788530658	0.028787
RHOXF2B	2.013705437	0.1936463	2.10608468	0.1641043	2.9194203	0.04517
RP11-107F6.	2.400179485	0.1748792	0.100254633	0.957226	3.482696259	0.035507
P11-1149O2	0.905725477	0.3228219	1.44502035	0.0936305	1.845929726	0.029276
RP11-159H3.	-1.912804727	0.1042905	0.116274738	0.8981354	-2.594875238	0.046437
P11-173G21	-1.786696922	0.0565554	-0.751896007	0.3450036	-2.248328899	0.02343
P11-211A18	-1.266519317	0.2664612	-0.713802316	0.4922368	-3.844590113	0.004367
P11-212F11	-1.018709029	0.4197163	-1.260399786	0.3184455	-2.883109316	0.047501
RP11-253E3.	2.063280511	0.1572631	2.627922081	0.0571275	3.098721232	0.022371
P11-264F23	-0.830711828	0.4882306	-0.773777395	0.5070322	-3.736061863	0.009269
P11-323D18	-0.956123304	0.3964065	-0.187906127	0.85173	-2.812388841	0.03562
P11-331G2	-1.149614002	0.2595653	-0.542791429	0.5584968	-3.366016971	0.009298
RP11-343B5.	0.95628516	0.302646	1.637648636	0.0590936	2.027168621	0.017702
P11-350G24	0.336182623	0.845797	0.834698713	0.6272757	2.914019695	0.049905
P11-378J18	-0.532244054	0.5269393	-1.682327267	0.077532	-2.288653549	0.034966
P11-561B11	0.336191507	0.8397955	2.417515346	0.092846	2.920187372	0.038212
P11-662B19	1.03612125	0.5001047	2.398886409	0.0668545	2.729580234	0.034222
RP11-77K12.	-0.835243273	0.4507023	-0.388761887	0.7004284	-3.455141326	0.009138
P3-391O22	-1.107184697	0.2304005	-1.555016346	0.1010864	-2.04992708	0.048031
P5-1120P11	0.336174059	0.8474182	0.821609414	0.6381699	3.249963531	0.034122
RPEP4	-1.857139511	0.1261342	0.06656245	0.9373574	-2.708552026	0.028233
RPL21P4	2.741074464	0.0631503	2.14740035	0.1591894	3.234019217	0.021804
RPL3P3	-0.474341455	0.6683349	-0.441790115	0.6811022	-3.380762534	0.012979
RPL6P7	-1.529733736	0.1776695	-1.07494694	0.2887247	-2.978409543	0.020003

RPL9P25	1.95642221	0.1624415	1.097941316	0.4533533	2.882789367	0.030947
RPL9P28	1.777175708	0.299498	2.487966621	0.1144998	3.988550083	0.006513
RPS4XP11	2.362413777	0.0833741	2.396269263	0.0733208	2.722475856	0.039003
RWDD4P1	-1.398054366	0.2071139	-0.791894435	0.4437209	-2.463936279	0.048035
SERPINB4	1.290985228	0.3183476	0.840048268	0.5196185	2.510151007	0.040333
SLC35D3	1.48119454	0.1105683	-2.163990321	0.1009798	1.811538906	0.045044
SPRR2E	3.371648321	0.0554647	0.100271176	0.9596593	3.605324126	0.039014
SUMO2P14	-1.36849858	0.1447497	-0.050608715	0.9483596	-2.849746278	0.019735
TMEM207	-0.142501234	0.8879661	-0.423577146	0.6759401	-2.378888406	0.046026
TMEM229A	-1.353567723	0.1122204	-0.396399228	0.6114386	-3.812576812	0.002134

Table S1. DEGs of STM, T3SS-1^{mut} and T3SS-2^{mut}-infected HIOs at 2.5h relative to PBS-injected HIOs. Significant DEGs ($p < 0.05$) in at least one infection condition are listed.
