# Supplementary material for: Salmonella enterica Serovar Typhimurium SPI-1 and SPI-2 Shape the Global Transcriptional Landscape in a Human Intestinal Organoid Model System
Source: mBio. 2021 May 18;12(3):e00399-21. doi: 10.1128/mBio.00399-21 (PMC8262845; doi:10.1128/mBio.00399-21)
Supplement: TABLE S2 [file mbio.00399-21-st002.pdf]

Table S2: Significant genes 8h pi

Symbol	STM log$_2$(fold change)	STM p-value	T3SS-1^{mut} log$_2$(fold change)	T3SS-1^{mut} p-value	T3SS-2^{mut} log$_2$(fold change)	T3SS-2^{mut} p-value
CXCL6	5.597614945	1.91E-43	6.509501976	3.53E-58	5.741659725	1.23E-45
SOD2	2.81705125	1.23E-43	2.887465979	9.17E-46	2.900980272	3.54E-46
CCL20	5.300510006	4.49E-35	5.68262129	4.61E-40	5.787032343	1.74E-41
DEFB4B	7.243911346	1.55E-32	7.562899546	2.48E-35	6.777292196	1.12E-28
IKBKE	1.772301168	5.87E-32	1.761030059	1.13E-31	1.848226461	7.45E-35
CXCL2	3.549344875	1.91E-29	3.585719389	4.61E-30	3.872555325	7.66E-35
IL32	3.488754107	7.37E-27	3.208438023	6.21E-23	3.24484966	1.90E-23
CXCL5	4.548879155	7.53E-25	4.678411666	3.43E-26	4.944753146	4.57E-29
TNFAIP2	3.528253199	7.49E-25	3.554410485	3.36E-25	3.688265891	5.20E-27
ZC3H12A	2.524102503	1.70E-24	2.61729811	2.96E-26	2.507612265	3.15E-24
CXCL1	3.381493656	7.73E-23	3.642327409	2.99E-26	3.71203949	3.33E-27
NFKBIA	2.140364484	3.10E-22	1.686581117	2.15E-14	2.154868819	1.53E-22
LAMC2	1.760129217	2.23E-20	1.936218087	2.49E-24	1.673663127	1.41E-18
SLC6A14	3.254708399	8.97E-20	3.135119739	1.84E-18	3.619726693	4.39E-24
IL6ST	1.240230314	3.66E-19	0.912677163	4.56E-11	1.109059508	1.23E-15
C6orf222	2.81489137	4.21E-19	2.682175885	1.71E-17	3.297339889	1.01E-25
TNFSF14	3.113410599	7.88E-19	2.86115791	3.89E-16	3.111467391	5.97E-19
CXCL10	5.644047208	8.80E-19	4.643498418	3.49E-13	5.670983023	5.93E-19
NFKBIZ	1.098090646	3.10E-18	1.020753522	5.54E-16	1.42227805	1.29E-29
VNN3	3.025897821	3.58E-18	2.535691843	4.98E-13	2.946707147	2.05E-17
CXCL3	2.72446395	6.00E-18	2.968790533	4.86E-21	2.974344351	4.08E-21
OPTN	1.384529312	1.54E-17	1.112710255	7.20E-12	1.233040915	2.96E-14
GGCT	-0.96413316	5.42E-16	-0.519949982	6.83E-06	-0.562284855	1.23E-06
UBD	3.513513093	2.93E-15	4.889304684	2.94E-28	3.232234774	3.84E-13
TMEM14A	-1.109393403	3.28E-15	-0.885223488	6.48E-11	-0.89228916	3.68E-11
RARRES1	2.498000834	8.90E-15	2.887854218	2.41E-19	2.106430486	6.34E-11
ADAR	0.427531921	1.38E-14	0.238485553	1.72E-05	0.356327219	1.23E-10
KLHL5	0.77103178	3.95E-14	0.865405647	1.52E-17	1.013609706	1.34E-23
ICAM1	3.796347735	4.52E-14	3.4380986	8.37E-12	3.750145211	9.06E-14
ZC3H12C	1.041126776	5.48E-14	1.036742073	6.12E-14	0.913948456	3.76E-11
TNFAIP3	2.43530681	7.90E-14	1.790749504	4.11E-08	2.457862458	4.54E-14
ATP5H	-0.575098915	1.54E-13	-0.265514025	0.00055	-0.353269813	4.45E-06
GCNT3	1.881369483	2.26E-13	1.601323428	4.37E-10	2.290811034	4.12E-19
SPRR2A	7.554988969	3.06E-13	7.519575376	3.91E-13	6.92393794	2.41E-11
P11-761N21	41.59064444	5.54E-13	-0.296849595	0.95958	-0.48560085	0.933921
CBX3	-0.500354773	5.82E-13	-0.247151293	0.00033	-0.180912735	0.008446
DUT	-1.13899228	6.40E-13	-0.587408132	0.00015	-0.829709676	9.66E-08
LRP11	-0.775958413	6.51E-13	-0.546366315	2.98E-07	-0.541789385	3.72E-07
FANCL	-0.901078799	1.03E-12	-0.642984515	2.67E-07	-0.550609781	9.21E-06
GRINA	0.928222913	1.87E-12	0.226091312	0.08775	0.204630421	0.122651
LIPG	1.302639796	2.45E-12	1.532775795	1.41E-16	1.504914798	4.96E-16
ARF3	0.739554728	3.20E-12	0.354766343	0.00083	0.515803633	1.17E-06
SGK1	1.793845931	4.34E-12	1.506011266	6.04E-09	1.72402544	2.72E-11
SDCBP2	1.210653999	4.79E-12	1.201867816	6.48E-12	1.106909692	2.55E-10

RHOG	0.8240319	6.26E-12	0.449121935	0.00021	0.516825472	1.78E-05
DUOXA2	3.408401335	7.75E-12	3.489417724	2.44E-12	2.866612894	8.68E-09
MYO1B	0.780714645	8.83E-12	0.620782467	5.62E-08	0.931320443	3.28E-16
UNG	-1.124012539	9.25E-12	-0.759591931	3.23E-06	-1.15477972	1.98E-12
SNPH	3.512013436	1.02E-11	3.877783443	5.52E-14	3.094058934	2.08E-09
TUBB2A	1.046674747	1.43E-11	0.999516965	1.06E-10	0.777468403	5.28E-07
BCL3	1.747587301	1.70E-11	0.719916958	0.00593	1.045370087	6.19E-05
LITAF	1.246092133	1.90E-11	0.34401987	0.06432	1.178132216	2.11E-10
OAS3	1.043870529	2.42E-11	0.882061759	1.66E-08	0.969798058	5.33E-10
HRSP12	-0.920525472	2.53E-11	-0.52918143	5.88E-05	-0.481236723	0.000241
IL8	3.833444329	3.34E-11	3.907650753	1.38E-11	4.040425034	2.76E-12
MAD2L1	-0.916182482	4.68E-11	-0.485781998	0.00042	-0.314594574	0.022138
DEFB4A	5.643649266	5.57E-11	6.545360238	2.70E-14	5.497031499	1.71E-10
JAK1	0.770275454	5.84E-11	0.90801077	1.11E-14	0.825329531	2.19E-12
SRD5A3	1.220683742	6.87E-11	1.302562207	3.16E-12	1.11477368	2.48E-09
TOMM7	-0.74908048	6.90E-11	-0.36082279	0.00136	-0.48890904	1.48E-05
VRK1	-1.022921749	7.25E-11	-0.72287658	3.14E-06	-0.518777287	0.000804
CX3CL1	2.250984176	8.76E-11	2.373116585	6.92E-12	1.715050187	8.17E-07
IL19	5.591551302	9.81E-11	6.394229779	1.14E-13	5.408928801	3.85E-10
LYN	1.292225838	1.06E-10	1.418941682	1.19E-12	1.171901197	4.54E-09
TMEM261	-0.673174017	1.11E-10	-0.236725101	0.02065	-0.359025737	0.00045
SBNO2	1.883607379	1.13E-10	1.167081017	6.95E-05	1.666162769	1.20E-08
IFNGR1	1.129848143	1.42E-10	1.414369385	8.79E-16	1.205472814	7.30E-12
SRP9	-0.387462927	1.48E-10	-0.10644328	0.07639	-0.043284965	0.470641
TNIP1	1.123604486	1.48E-10	0.845501299	1.44E-06	1.077540019	7.94E-10
TAPBP	0.793432194	1.66E-10	0.533591129	1.73E-05	0.253336934	0.041621
GDI1	0.686535517	1.73E-10	0.335708236	0.00185	0.546072767	3.65E-07
C3	2.128478	1.90E-10	2.072005609	5.62E-10	2.255752315	1.47E-11
MAGI1	-0.912158151	2.32E-10	-0.920808721	1.37E-10	-0.827531016	7.66E-09
EGR1	1.657152958	2.61E-10	0.599470615	0.02274	1.043900363	7.01E-05
ST5	0.989822237	2.93E-10	0.526849596	0.00081	0.634150616	5.46E-05
SRF	0.635346464	3.40E-10	0.192211469	0.05935	0.487567602	1.36E-06
WARS	0.873175691	3.50E-10	0.626076753	6.79E-06	0.692028472	6.49E-07
LTB	3.420563091	3.61E-10	3.612286212	2.71E-11	3.608214334	2.85E-11
NDUFA1	-0.571958584	3.66E-10	-0.128806171	0.14341	-0.25953538	0.003307
AP006216.11	-2.397634892	4.31E-10	-1.257563179	0.00079	-1.388393018	0.000214
KYNU	2.153901238	4.64E-10	2.611680195	4.00E-14	2.246838878	7.89E-11
CTPS2	-0.532610633	4.79E-10	-0.439366461	2.34E-07	-0.141527286	0.094172
NFKB2	1.601585115	4.87E-10	0.987012427	0.00013	1.423392504	3.18E-08
AHCYL2	1.36495043	4.98E-10	1.106620406	4.63E-07	1.260965898	8.97E-09
THBS1	1.803482362	5.33E-10	0.909682374	0.00174	1.682355858	6.94E-09
CYB5A	-0.832269799	5.47E-10	-0.5621977	2.47E-05	-0.546903187	4.01E-05
MCMDC2	-1.011402836	5.85E-10	-0.50333863	0.00096	-0.523837719	0.000594
RPL36A	-1.122643901	6.83E-10	-0.580480521	0.00136	-0.508350616	0.005021
DAPK3	0.862217556	7.35E-10	0.498107223	0.00039	0.796835547	1.12E-08
SLC2A6	1.898462139	7.42E-10	1.930526359	3.48E-10	1.742565707	1.53E-08
CEBPD	2.034039059	7.73E-10	1.436825367	1.43E-05	1.333215784	5.67E-05

TNFRSF9	7.659960442	7.90E-10	7.027694961	1.82E-08	7.924369166	1.92E-10
PCNXL3	0.681966835	9.01E-10	0.098206197	0.38066	0.006234181	0.955698
OSBPL1A	-0.65202699	9.71E-10	-0.542735711	2.09E-07	-0.536738856	2.96E-07
BIRC3	2.031788226	9.94E-10	1.875688178	1.68E-08	2.219654682	2.36E-11
PI3	4.514295293	1.04E-09	3.778124193	3.52E-07	4.678278827	2.38E-10
SPRR1A	5.042591801	1.05E-09	5.917954299	6.35E-13	4.968244709	1.77E-09
S100A3	4.154951994	1.15E-09	4.638630274	7.75E-12	4.176635805	8.38E-10
MAP3K11	0.693739125	1.27E-09	0.410353961	0.00034	0.410036703	0.000347
DHRS7	-0.803439786	1.30E-09	-0.326922026	0.01273	-0.488112112	0.000205
PCNA	-1.005738298	1.30E-09	-0.620143094	0.00017	-0.644977754	9.28E-05
TCEA1	-0.527456712	1.56E-09	-0.321889699	0.00021	-0.222491239	0.010145
PKHD1	-0.982112426	1.64E-09	-0.395990184	0.01201	-0.822799963	2.67E-07
ARHGEF19	-0.906836507	1.76E-09	-0.808328204	5.76E-08	-1.031712986	7.68E-12
TRIM31	2.283307349	2.01E-09	1.445754058	0.00015	1.979752761	1.95E-07
OXTR	1.872062874	2.31E-09	2.09403756	1.68E-11	1.602138367	3.27E-07
ADAMTS1	0.818513386	2.43E-09	0.253395319	0.06533	0.827061609	1.52E-09
AKT1S1	0.712082873	2.41E-09	0.319459117	0.00769	0.197010949	0.100573
TD-2287O16	-1.060655442	2.95E-09	-0.480477826	0.00686	-0.725438538	4.56E-05
CFLAR	0.893497947	3.09E-09	0.819235284	5.46E-08	0.823406496	4.64E-08
P11-274B21	1.14397746	3.28E-09	0.152091661	0.44299	0.596413844	0.002214
SPRY4	0.857090204	3.44E-09	0.312411794	0.03175	0.318719907	0.028335
CRIM1	0.808057485	3.49E-09	0.408088094	0.00287	0.467752247	0.000629
XK	-1.307413552	3.52E-09	-0.706861575	0.00131	-1.559582824	1.88E-12
ZFP36L1	0.80995857	3.58E-09	0.192860959	0.16084	0.226087594	0.100047
RBMS2	0.831072823	4.02E-09	0.091317757	0.52063	0.515517083	0.000265
SMIM14	-0.920356608	4.72E-09	-0.702452987	7.44E-06	-0.953137908	1.25E-09
TFPI	0.857527696	5.05E-09	0.80311841	4.35E-08	1.061677869	4.43E-13
MANEA	-1.043933113	5.16E-09	-0.586391259	0.00088	-0.644443125	0.000262
HMGCS2	-2.145084551	5.81E-09	-1.944823769	1.26E-07	-2.776882568	5.06E-14
PTAFR	2.08269672	5.89E-09	2.078119779	6.04E-09	1.729308025	1.36E-06
SLC37A1	0.778740834	6.32E-09	0.843538448	2.86E-10	0.7534075	1.77E-08
EBPL	-0.666519522	6.68E-09	-0.3913369	0.00045	-0.327597778	0.003121
PIEZO1	1.161804005	7.16E-09	0.960746671	1.70E-06	0.881797978	1.14E-05
ZSWIM8	0.848846927	7.16E-09	0.646082326	1.07E-05	0.808842898	3.44E-08
TXNDC16	-0.924801854	7.67E-09	-0.382148956	0.01395	-0.477349904	0.002123
PRPS2	-1.128194735	7.89E-09	-0.63835787	0.00105	-1.099318714	1.79E-08
AKR7A2	-0.537260994	8.57E-09	-0.330410921	0.00029	-0.621259567	1.69E-11
RAC1	0.668410322	9.92E-09	0.634843234	5.14E-08	0.66059181	1.44E-08
PAPSS1	-0.505658916	1.03E-08	-0.258262326	0.00302	-0.272495755	0.001786
SMAD3	1.12838624	1.01E-08	0.666306684	0.00072	0.861489262	1.22E-05
ZDHHC20	-0.511427155	1.02E-08	-0.201649913	0.02344	-0.323881646	0.000273
CTDSPL	-0.558971646	1.08E-08	-0.469070427	1.46E-06	-0.38035579	9.09E-05
ARHGAP18	-0.73930156	1.12E-08	-0.486476297	0.00015	-0.810989946	3.12E-10
ABCD3	-0.513848332	1.22E-08	-0.117508019	0.18923	-0.232584304	0.009432
NUAK2	1.931246631	1.23E-08	0.986457277	0.0043	1.427838279	2.82E-05
ACKR3	1.209052734	1.29E-08	0.477257535	0.02528	0.692673342	0.001146
GPD1L	-0.86539446	1.46E-08	-0.535898486	0.00039	-0.753721505	6.61E-07

OLR1	2.021675274	1.51E-08	1.61692195	6.06E-06	1.54659457	1.53E-05
PEA15	0.740141492	1.67E-08	0.424887519	0.0012	0.579580669	9.62E-06
ACADM	-0.523580814	1.72E-08	-0.214300881	0.0185	-0.25101614	0.005846
CXCL16	1.08631005	1.92E-08	0.939327202	1.19E-06	0.815941737	2.50E-05
MAP7D2	2.044438195	1.96E-08	1.589739549	1.21E-05	1.583887369	1.34E-05
CSNK1G2	0.800056244	2.02E-08	0.492088604	0.00056	0.587523813	3.73E-05
DSE	1.190835587	2.01E-08	1.181225253	2.59E-08	1.355070046	1.67E-10
TNFAIP1	0.758281013	2.01E-08	0.52836411	9.32E-05	0.793641182	3.95E-09
CDC23	-0.482935901	2.18E-08	-0.113129533	0.18072	-0.064043375	0.446817
SGPP2	1.44730145	2.17E-08	1.299230758	5.02E-07	1.443567	2.30E-08
DNA2	-0.987236533	2.20E-08	-0.448523422	0.01027	-0.435444241	0.012366
LRBA	-0.495041609	2.28E-08	-0.347470332	8.34E-05	-0.298655442	0.000719
TBCA	-0.564954188	2.32E-08	-0.444996967	8.98E-06	-0.189720482	0.056882
RND1	2.639282658	2.57E-08	2.808872572	3.02E-09	2.757753781	5.78E-09
MCM3	-0.676708143	2.75E-08	-0.555436134	4.79E-06	-0.384852223	0.001511
VNN1	2.340496975	3.18E-08	2.003150053	2.43E-06	1.994892825	2.59E-06
SCCPDH	-0.628845452	3.22E-08	-0.220526362	0.0479	-0.329642707	0.003153
PTPN1	0.434070669	3.35E-08	0.069614103	0.37952	0.316589896	5.19E-05
SDC4	1.32910536	3.35E-08	1.596113912	3.30E-11	1.493099798	5.49E-10
ASAH1	-0.585421283	3.49E-08	-0.135440623	0.19802	-0.531691622	4.90E-07
TOMM70A	-0.492287517	3.87E-08	-0.242859441	0.00625	-0.288258628	0.001177
LRG1	1.271465963	3.98E-08	1.143773861	7.54E-07	1.230054245	1.03E-07
SH3BGRL2	-0.798155847	4.28E-08	-0.460647749	0.00153	-0.921289671	2.49E-10
TMEM181	-0.586624764	4.47E-08	-0.165977482	0.11996	-0.560523673	1.61E-07
RHBDF1	0.925268543	4.75E-08	0.523681633	0.00206	0.643379292	0.000148
CCL2	2.782813523	4.84E-08	1.788613424	0.00046	2.361675494	3.64E-06
MAP3K8	1.356221998	5.00E-08	1.477614882	2.66E-09	1.651823666	2.74E-11
AASDHPPT	-0.539255337	5.08E-08	-0.154057787	0.11533	-0.040746916	0.676203
TP53INP2	1.496945909	5.10E-08	0.70078026	0.01185	0.959313124	0.000513
CXCL11	5.853814455	5.15E-08	5.505876634	3.08E-07	6.015783673	2.05E-08
KLF6	0.914796385	5.50E-08	0.535504699	0.00147	0.818654897	1.12E-06
TSPAN18	1.42294795	6.09E-08	0.814144143	0.00214	1.130304389	1.73E-05
EIF4BP7	-1.347281078	6.85E-08	-0.055693393	0.81347	-0.457830838	0.054606
GPR153	0.957383812	7.21E-08	0.556517155	0.00187	0.635452372	0.000363
TNFRSF1A	0.633545417	7.50E-08	0.413336501	0.00044	0.463193892	8.26E-05
HDAC9	1.619039692	7.60E-08	1.37779327	4.69E-06	1.896846263	2.83E-10
ID1	1.168442558	8.01E-08	1.152992972	1.17E-07	1.436550137	3.97E-11
MYO1C	0.578392991	8.36E-08	0.205578095	0.05698	0.359865516	0.000844
CAPS	-1.251932187	9.13E-08	-0.602798223	0.00942	-1.06278374	5.08E-06
BRCA1	-0.872049436	1.01E-07	-0.379037314	0.01922	-0.520171832	0.001318
PTPRE	1.17733911	1.02E-07	1.272104144	6.62E-09	0.694233304	0.001705
SCOC	-0.714552048	1.09E-07	-0.176712492	0.18591	-0.242737265	0.069353
GYS1	0.58303835	1.12E-07	0.28041413	0.0108	0.229827932	0.037057
B4GALT1	1.048442096	1.16E-07	0.903195657	4.96E-06	0.550878417	0.005389
RUFY1	-0.535723893	1.21E-07	-0.332154894	0.00091	-0.242827637	0.014353
RPL21	-0.633187951	1.22E-07	-0.29915677	0.01228	-0.289391507	0.015409
AKAP2	1.18526146	1.25E-07	0.3024774	0.17797	0.936999042	2.93E-05

GEMIN6	-0.709914204	1.26E-07	-0.248352303	0.05878	-0.143649461	0.270403
USP1	-0.49615332	1.35E-07	-0.108313441	0.24499	0.008686205	0.925547
SPRR2F	4.053799865	1.43E-07	4.161115625	6.34E-08	3.666883045	1.99E-06
HMGN3	-0.687799968	1.45E-07	-0.351993436	0.00661	-0.312198368	0.015862
IVNS1ABP	-0.423326084	1.48E-07	-0.194444366	0.01522	-0.180974291	0.023752
IL6	3.511151041	1.51E-07	1.803501129	0.00781	3.220604125	1.48E-06
NID1	0.881937449	1.50E-07	0.115535963	0.49203	0.460463117	0.00612
POLA1	-0.67444416	1.50E-07	-0.483247682	0.00016	-0.476801157	0.000189
NFKBIB	1.038855762	1.52E-07	0.497045649	0.01238	0.443509741	0.025589
MCM4	-0.69462919	1.60E-07	-0.390188668	0.00317	-0.394943409	0.002798
GALNT12	-0.934885043	1.63E-07	-0.618640243	0.00047	-0.978568964	3.76E-08
STOM	0.976999974	1.69E-07	0.522223899	0.00522	0.630606656	0.00074
SPCS1	-0.41677615	1.79E-07	-0.101218766	0.19403	-0.325281531	3.44E-05
PLAGL1	0.854843779	2.01E-07	0.488453544	0.00306	0.30131933	0.067779
BLOC1S5	-0.714605911	2.24E-07	-0.384856067	0.00474	-0.446883393	0.00105
ALG6	-0.533303869	2.29E-07	-0.11827926	0.22489	-0.191459513	0.049498
CREG1	-0.655728071	2.36E-07	-0.340338749	0.00669	-0.639316103	4.00E-07
CSNK1E	0.448218471	2.36E-07	0.156662358	0.07128	0.27264769	0.001641
ADAMTS9	1.190503262	2.54E-07	1.190051287	2.54E-07	1.26435898	4.33E-08
TMEM64	-0.618296935	2.53E-07	-0.248016464	0.03679	-0.24429387	0.039597
FAM46C	-1.750895847	2.60E-07	-1.137421203	0.00076	-2.393229881	2.45E-12
VPS35	-0.468363447	2.76E-07	-0.132460858	0.14436	-0.212287062	0.019375
UBQLN4	0.640123181	2.97E-07	0.328849643	0.00855	0.393706597	0.001597
PRMT3	-0.695099075	3.04E-07	-0.225136303	0.08906	-0.494962158	0.0002
SCO1	-0.562437887	3.04E-07	-0.295027558	0.00683	-0.350793623	0.001279
WDHD1	-0.8036096	3.09E-07	-0.421253913	0.00682	-0.344607867	0.026676
MUC13	6.398486039	3.23E-07	5.223150778	3.15E-05	6.500521255	2.06E-07
COX6C	-0.482592484	3.36E-07	-0.155747855	0.09398	-0.171690572	0.064646
BID	1.121048	3.59E-07	1.14705602	1.79E-07	0.680152591	0.001991
UBL3	-0.582110744	3.72E-07	-0.340441791	0.00271	-0.289277581	0.010699
CCDC25	-0.628812016	3.91E-07	-0.476463116	0.00011	-0.409452024	0.000864
CUL4B	-0.522396839	4.01E-07	-0.402466143	8.86E-05	-0.29157581	0.004429
꞉P11-384K6.	1.179112045	4.02E-07	0.150610693	0.53034	0.131189353	0.584013
CLUHP3	0.785496306	4.05E-07	0.642881679	3.23E-05	0.931836344	1.31E-09
ITGA5	0.889990115	4.08E-07	0.452865485	0.00997	0.661291266	0.000166
LIG1	-0.714199386	4.07E-07	-0.585284823	2.91E-05	-0.535901155	0.000132
MRPL33	-0.503374954	4.25E-07	-0.295935395	0.00218	-0.311919414	0.001206
SC5D	0.574205717	4.50E-07	0.275984312	0.01524	0.494842867	1.33E-05
NIPSNAP3A	-0.536128249	4.59E-07	-0.178872485	0.07702	-0.256722526	0.011219
CABLES1	-1.234910499	4.71E-07	-0.970221567	6.37E-05	-1.519159466	5.81E-10
FBXL4	-0.545370296	4.70E-07	-0.417615997	0.0001	-0.515402128	1.59E-06
PPIL1	-0.548659241	4.96E-07	-0.138676047	0.19721	-0.067577675	0.529034
SNX7	-0.507076038	5.01E-07	-0.07044481	0.47332	-0.242092013	0.014118
EEF1A1P9	-0.916693706	5.08E-07	-0.598065216	0.00063	-0.463008958	0.00731
ENPP1	1.107080551	5.45E-07	1.579197605	6.40E-13	0.665736585	0.002645
ANKRD33B	3.341831341	5.51E-07	2.706823209	6.22E-05	3.217699846	1.34E-06
SUMF1	-0.529263909	5.59E-07	-0.232042694	0.02585	-0.495508745	2.30E-06

C1GALT1C1	-0.669930627	5.63E-07	-0.265572978	0.04398	-0.358014517	0.006653
EEF1A1	-0.591335907	5.67E-07	-0.306680014	0.00948	-0.231386695	0.050299
APIP	-0.551225877	5.71E-07	-0.320496384	0.00289	-0.229542662	0.031405
NSMF	0.878847173	5.84E-07	0.48875204	0.00575	0.679170347	0.000122
PCGF3	0.407124359	5.85E-07	0.298660694	0.00024	0.394099267	1.19E-06
NKIRAS2	0.378858071	6.01E-07	0.07109319	0.35145	0.183801535	0.01495
MAT2B	-0.421227378	6.29E-07	-0.23092161	0.0056	-0.159780593	0.054257
IARS2	-0.473342785	6.46E-07	-0.21568046	0.02265	-0.412084176	1.37E-05
SLCO3A1	1.504914907	6.50E-07	1.030285786	0.00065	1.340602739	8.67E-06
C1S	0.97412808	6.53E-07	0.604846493	0.00203	0.874598449	7.79E-06
DSCC1	-1.091980736	6.93E-07	-0.651870353	0.00235	-0.674038564	0.001616
NADK2	-0.499677664	7.30E-07	-0.183903823	0.06255	-0.251200616	0.011453
RNF207	0.8932138	7.28E-07	0.449958103	0.01292	0.865970775	1.45E-06
CCDC130	0.513670429	7.37E-07	0.325372182	0.00177	0.437874627	2.09E-05
ZYX	0.946462435	7.50E-07	0.082486205	0.6674	0.373478635	0.051304
CSF1	1.24039724	7.59E-07	0.634074176	0.01178	0.80322634	0.001388
TSFM	-0.576696808	8.04E-07	-0.171552878	0.12701	-0.139958655	0.211529
JUND	0.701981327	8.13E-07	0.078149952	0.58514	0.313783568	0.027755
SLC25A44	0.625078986	8.11E-07	0.376607704	0.00301	0.44843392	0.000401
ALDH9A1	-0.546783513	8.53E-07	-0.377888852	0.0006	-0.398941713	0.000296
TSPAN13	-0.561188496	8.54E-07	-0.181679767	0.10875	-0.420184254	0.000213
MRPS18B	-0.466576021	8.81E-07	-0.253066775	0.00666	-0.335650306	0.000317
DUOX2	2.318599347	8.90E-07	2.421184509	2.86E-07	1.755546664	0.000199
SUCLG2	-0.577784514	9.75E-07	-0.413483192	0.00044	-0.482829845	4.04E-05
IGFBP3	1.811827051	1.01E-06	1.471063742	7.18E-05	0.93344633	0.011776
GABPB2	-0.676590344	1.06E-06	-0.374733139	0.00644	-0.376009426	0.00632
ZNF330	-0.516474086	1.07E-06	-0.184075531	0.07557	-0.191185834	0.064037
SCIN	-1.438482694	1.08E-06	-1.097544357	0.00019	-2.105093908	1.04E-12
RPIA	-0.653891471	1.10E-06	-0.249072486	0.05286	-0.443965193	0.000623
OXR1	-0.594548976	1.13E-06	-0.299137296	0.0137	-0.512656636	2.48E-05
RFC3	-0.767587385	1.13E-06	-0.573664606	0.00024	-0.439161675	0.004721
RPL39	-0.601378097	1.13E-06	-0.069503439	0.57314	-0.314285721	0.010871
AP2A1	0.516328055	1.16E-06	0.277846819	0.00882	0.116028708	0.275319
FAM83A	-0.773166667	1.17E-06	0.751087986	2.18E-06	0.289025563	0.068489
MFSD6L	-1.228724965	1.21E-06	-0.612896433	0.01144	-1.382288466	4.00E-08
FAM213A	-0.511037054	1.25E-06	-0.396739425	0.00014	-0.539370444	2.44E-07
ATP10B	1.224474043	1.26E-06	1.357655119	7.54E-08	0.788395055	0.001814
NUDT21	-0.321790268	1.30E-06	-0.152470187	0.02112	-0.034926482	0.596236
MAGOHB	-0.621730678	1.31E-06	-0.112269295	0.37261	-0.090348173	0.470415
ETV3	0.567853672	1.33E-06	0.614860549	1.46E-07	0.650248093	2.62E-08
PIK3C2G	-1.333581847	1.35E-06	-0.326102693	0.23542	-0.736050014	0.007458
GLP1R	-2.575182931	1.37E-06	-2.176966584	1.91E-05	-2.908264899	6.52E-08
RAP1B	0.408723317	1.40E-06	0.39136618	3.63E-06	0.544231391	1.13E-10
CFB	1.101375117	1.41E-06	1.328100559	5.83E-09	1.355682102	2.80E-09
EIF4A2	-0.435320321	1.44E-06	-0.052776901	0.55839	-0.194920642	0.030645
SCP2	-0.510171822	1.47E-06	-0.220750405	0.03597	-0.421226909	6.46E-05
TMEM168	-0.639837172	1.48E-06	-0.197607862	0.13351	-0.336393411	0.010688

TMEM167A	-0.647614567	1.48E-06	-0.322855331	0.0162	-0.173921363	0.194837
ESD	-0.428521913	1.51E-06	-0.23221582	0.00856	-0.090744587	0.30237
H3F3A	-0.475975491	1.52E-06	-0.167722059	0.08944	-0.155549081	0.115138
IFITM3	0.66870278	1.52E-06	0.272961731	0.04985	0.407374402	0.0034
DPY30	-0.545066693	1.55E-06	-0.296995748	0.00746	-0.293054856	0.008095
ATP6V0A1	0.642880587	1.60E-06	0.36527874	0.0066	0.483522066	0.000312
TAP1	1.024852498	1.69E-06	1.028262878	1.46E-06	0.767063276	0.000341
ITGA1	0.942835069	1.76E-06	0.562261601	0.00439	0.730606601	0.000212
NACC1	0.941533591	1.80E-06	0.037560907	0.84906	0.105433474	0.593148
OSMR	1.160395242	1.80E-06	1.055639696	1.40E-05	0.996987783	4.09E-05
SLC39A11	-0.520249949	1.79E-06	-0.085711465	0.41908	-0.489438927	5.48E-06
CDR1	2.071113333	1.81E-06	0.100018146	0.82536	-0.055960915	0.902152
PHIP	-0.371507937	1.85E-06	-0.177629341	0.02196	-0.187728505	0.01542
PYURF	-0.501004238	1.86E-06	-0.074737744	0.464	-0.333439455	0.001199
TMEM14C	-0.453898719	1.89E-06	-0.110587846	0.23514	-0.407073693	1.52E-05
POLR2G	-0.578711815	2.05E-06	-0.24416922	0.04136	-0.30679037	0.0104
SMIM7	-0.408783085	2.05E-06	-0.258500008	0.00238	-0.237303765	0.005119
AFM	-3.08488267	2.13E-06	-1.817216323	0.00366	-1.737351416	0.004852
KBTBD7	-0.50166227	2.17E-06	-0.281333423	0.00658	-0.373997291	0.000311
STAT6	0.433542481	2.17E-06	0.256096879	0.00512	0.291937236	0.001401
C8orf4	1.847978081	2.22E-06	1.819726372	3.12E-06	2.005529721	2.71E-07
CAPG	-0.57596398	2.30E-06	-0.212804359	0.07919	-0.327701799	0.006908
MED15	0.766315889	2.31E-06	0.319319684	0.04939	0.445275411	0.006084
C1R	0.936266324	2.32E-06	0.928227702	2.79E-06	0.767870842	0.000106
PRCP	-0.406951245	2.35E-06	-0.095987353	0.25569	-0.14322551	0.089704
CSF3	4.026403194	2.38E-06	4.002120264	2.71E-06	3.930299808	3.98E-06
MTAP	-0.510253785	2.42E-06	-0.403423203	0.00018	-0.264350282	0.014004
TRIOBP	0.422513589	2.45E-06	0.278336635	0.00191	0.018983216	0.832672
CFL2	0.745229846	2.47E-06	0.561591568	0.0004	0.934634926	2.75E-09
PARP14	0.646243065	2.47E-06	0.745540214	5.08E-08	0.688530673	4.85E-07
PPARD	0.75801454	2.54E-06	0.314148658	0.05344	0.337144799	0.037355
TMEM107	-0.638754916	2.54E-06	0.081791619	0.52458	-0.297103319	0.022374
ZMIZ2	0.674559555	2.66E-06	-0.053417612	0.71228	0.082357989	0.568714
SERPINB8	1.177067863	2.70E-06	1.103043163	1.10E-05	1.454188715	5.92E-09
ZNF367	-1.104576565	2.79E-06	-0.337440039	0.14364	-0.631514394	0.006428
POGZ	0.580964174	2.81E-06	0.228534561	0.06596	0.251895532	0.042196
CREB1	0.642529691	2.84E-06	0.006447985	0.96252	0.55171825	5.58E-05
SLC30A1	-0.577339325	2.90E-06	-0.185759267	0.12855	-0.485401506	7.60E-05
RIMBP3C	-0.723429782	2.91E-06	-0.501913299	0.00117	-0.336678531	0.029446
NACAD	1.588655262	2.92E-06	1.06170111	0.00188	0.839336725	0.014656
FEZ1	1.343141834	2.98E-06	1.560531212	5.04E-08	1.302683519	5.44E-06
AB019441.29	-1.518951105	3.01E-06	-0.858779497	0.00359	-0.917414634	0.001852
RNF19A	1.124616064	3.04E-06	0.893091099	0.00021	1.075721262	7.91E-06
STAP2	0.752695027	3.13E-06	0.894084438	2.74E-08	0.556077756	0.000563
RFC5	-0.66527524	3.17E-06	-0.660354422	2.95E-06	-0.325145383	0.020473
LIMD2	1.058881159	3.21E-06	0.631855574	0.00558	0.789990192	0.000507
IL3RA	1.538833057	3.22E-06	0.758831147	0.0264	1.230452561	0.000207

EEF1E1	-0.692621497	3.30E-06	-0.35247492	0.01648	-0.28996546	0.048082
SS18	0.45833907	3.36E-06	0.332896335	0.00072	0.394004247	6.05E-05
FAM219A	0.893062176	3.38E-06	0.404589785	0.03601	0.692507577	0.00031
COMT	-0.741010438	3.48E-06	-0.743148603	3.07E-06	-0.903000376	1.52E-08
GSTA4	-0.555520919	3.52E-06	-0.624828284	1.58E-07	-0.48872778	3.59E-05
HAUS4	-0.607047145	3.55E-06	-0.414158756	0.00124	-0.443597883	0.000566
WBP2	0.847702033	3.58E-06	0.480545704	0.00868	0.287638337	0.116868
SAT1	1.193562608	3.61E-06	1.202784539	3.03E-06	1.19208166	3.70E-06
MESDC1	0.600541126	3.80E-06	0.344142019	0.00827	0.355607883	0.006219
RBPMS	0.593155037	3.81E-06	0.062134594	0.63031	0.49278443	0.00012
C14orf159	-0.642535192	3.87E-06	-0.376626531	0.00606	-0.410860778	0.002801
CHEK2	-0.627964942	3.92E-06	-0.361099244	0.00669	-0.314177591	0.018263
TYMP	1.14852637	3.92E-06	1.323395959	8.20E-08	0.863826367	0.000534
HSDL2	-0.59458585	3.96E-06	-0.247713773	0.05322	-0.338276348	0.008355
INSR	0.697254202	4.05E-06	0.609672022	5.53E-05	0.585054917	0.000108
MACC1	-0.624377025	4.04E-06	-0.45269931	0.00082	-0.561411703	3.33E-05
MGRN1	0.788461206	4.12E-06	0.41746163	0.01511	0.427618931	0.012817
RPS21	-0.677511455	4.18E-06	-0.139885525	0.33948	-0.419073673	0.004267
SH3BGRL3	0.807600037	4.26E-06	0.338120508	0.05454	0.240500916	0.171588
FAM136A	-0.579788571	4.46E-06	-0.235291893	0.05994	-0.274724703	0.028004
PDCD4	-0.875971652	4.49E-06	-0.461410992	0.0155	-1.127182057	3.52E-09
TFAM	-0.601365478	4.52E-06	-0.345170497	0.00816	-0.287332674	0.027531
ALDH3A2	-0.801645623	4.56E-06	-0.723153859	3.44E-05	-0.64036551	0.00024
OSGEP	-0.458990413	4.83E-06	-0.207886533	0.0342	-0.134701329	0.167658
TMEM127	0.427735882	5.01E-06	0.176573774	0.06016	0.050236291	0.593
DLGAP4	0.778878219	5.11E-06	0.3520536	0.04037	0.40956365	0.016745
ORC1	-0.81943997	5.14E-06	-0.607385904	0.00064	-0.546134749	0.002082
RNF24	0.801926715	5.25E-06	0.547034602	0.00191	0.461348214	0.00883
ENDOD1	-0.582611444	5.34E-06	-0.538444349	2.35E-05	-0.791375108	6.16E-10
PSMG2	-0.393065606	5.35E-06	-0.028962421	0.72837	-0.047752928	0.566251
KCTD10	0.516045788	5.57E-06	0.268727944	0.01803	0.502603192	8.94E-06
PUS7L	-0.596682157	5.58E-06	-0.541337013	3.67E-05	-0.361259359	0.005769
GLTP	0.472884376	5.66E-06	0.413362526	6.56E-05	0.300684688	0.003759
SLC35A1	-0.466040654	5.67E-06	-0.196893258	0.05048	-0.311410078	0.002046
IL4R	0.708990067	5.73E-06	0.514501466	0.00098	0.429825442	0.00588
CSNK2B	0.497334108	5.79E-06	0.159867463	0.14563	0.390445632	0.000365
HIF1A	0.623237901	5.84E-06	0.570661687	3.31E-05	0.789145746	9.31E-09
DRAM1	0.855125061	5.94E-06	0.96099551	3.41E-07	0.667543571	0.000403
MCM6	-0.781239796	5.99E-06	-0.644435189	0.00018	-0.603336673	0.000451
CREB3L4	-0.956096274	6.12E-06	-0.551654038	0.00713	-0.694945778	0.000733
PTPMT1	-0.511955301	6.38E-06	-0.296513504	0.00811	-0.274909822	0.013801
ZZZ3	-0.417207916	6.36E-06	-0.251051568	0.00621	-0.252922771	0.005731
DECR1	-0.44974227	6.43E-06	-0.162726946	0.09541	-0.11570908	0.234545
DCLRE1A	-0.681369191	6.56E-06	-0.205666905	0.16571	-0.268045847	0.07123
SNX2	-0.532206863	6.56E-06	-0.28172945	0.01633	-0.337806551	0.004022
EEF1A1P1	-1.335905287	6.61E-06	-0.021754016	0.93297	-0.319039598	0.224488
HDGF	0.535215328	6.65E-06	0.60535195	3.42E-07	0.670842497	1.59E-08

GLRX	0.792560974	6.70E-06	0.582566339	0.00092	0.444525482	0.01185
WWC3	0.725905219	6.80E-06	0.57768541	0.00034	0.445788363	0.005716
DPY19L4	-0.639521215	6.95E-06	-0.224406925	0.1131	-0.180726309	0.201512
AGR2	-0.802562411	7.10E-06	-0.553432991	0.00195	-0.622113355	0.000499
DEPTOR	-1.056353226	7.10E-06	-0.786092563	0.00081	-1.189979303	4.16E-07
FABP6	5.712007943	7.24E-06	2.404518787	0.06041	2.579143615	0.043974
TMEM230	-0.303291935	7.24E-06	-0.081239202	0.22004	-0.25367928	0.000137
WIZ	0.57003278	7.20E-06	0.021003258	0.87011	0.081366122	0.524974
PFKM	-0.642922254	7.52E-06	-0.479118696	0.0008	-0.327963325	0.021319
SYNPR	-2.187842129	7.55E-06	-1.70106598	0.00047	-2.701156957	3.77E-08
TFE3	0.644136971	7.72E-06	0.279819742	0.05262	0.396245508	0.005903
TBC1D10B	0.40914729	7.74E-06	0.166204635	0.07022	0.09706943	0.289062
CIC	0.690142859	7.77E-06	0.072206963	0.64084	0.009625773	0.950432
SAMD4A	1.089802622	7.98E-06	0.671561053	0.00599	0.948473355	9.91E-05
RAD51AP1	-0.815631332	8.08E-06	-0.467219628	0.00951	-0.31912612	0.075856
CTTNBP2NL	0.495991709	8.16E-06	0.384062529	0.00055	0.465174724	2.75E-05
RCL1	-0.867430004	8.20E-06	-0.094664578	0.61609	-0.374301387	0.049065
CDCA7L	-0.826756645	8.23E-06	-0.691003841	0.00019	-0.584823851	0.001535
SESN1	-0.890617201	8.34E-06	-0.481791714	0.01525	-0.684354971	0.000579
TBC1D5	-0.46012424	8.33E-06	-0.40380182	8.69E-05	-0.470056544	4.86E-06
EBI3	1.986774319	8.44E-06	2.179406195	8.73E-07	1.850798438	3.22E-05
CCDC85A	1.947054685	8.54E-06	1.26519829	0.00427	1.737292217	7.07E-05
FNDC3B	0.789140483	8.57E-06	0.579946335	0.00107	0.715209763	5.47E-05
IRAK2	1.609667509	8.68E-06	1.533986636	2.21E-05	1.382448584	0.000133
ORC3	-0.384414766	8.83E-06	-0.329088777	0.00012	-0.217048862	0.01028
SF3A2	0.528615819	8.91E-06	0.168407433	0.16449	-0.022016392	0.855738
DHX40	-0.472555001	9.11E-06	-0.105692627	0.31696	-0.204449676	0.052861
P11-407G23	1.12221621	9.12E-06	0.56044871	0.03055	0.711297337	0.005375
FAM213B	0.556245936	9.37E-06	0.191443168	0.13112	-0.056690852	0.658557
SCML4	-1.787517969	9.43E-06	-1.287540647	0.00106	-1.722479363	1.50E-05
NUP37	-0.502960889	9.53E-06	-0.166641641	0.12988	-0.1580262	0.149789
PEX2	-0.393047867	9.64E-06	-0.039882246	0.64769	-0.056099402	0.519497
HMGN2P46	1.074211746	9.95E-06	1.314012106	3.94E-08	1.064722264	9.86E-06
ATXN2L	0.67124505	1.01E-05	0.112284657	0.46271	-0.033557316	0.826239
DUOXA1	2.015210504	1.03E-05	2.148833654	2.26E-06	1.994899808	1.18E-05
ERI1	-0.518837503	1.02E-05	-0.377310034	0.00121	-0.374923198	0.001255
SF3B4	0.452292628	1.02E-05	0.068124432	0.51057	0.254994547	0.012846
TMEM87A	-0.477370911	1.02E-05	-0.206770175	0.05349	-0.32576673	0.002394
ASUN	-0.634810094	1.03E-05	-0.343250953	0.01631	-0.284742159	0.046076
GRAMD1B	-1.349987836	1.03E-05	-1.211656824	6.43E-05	-1.142188005	0.000162
EFNA2	1.101850961	1.04E-05	0.542482963	0.03326	0.392939514	0.124992
ATP5I	-0.457604045	1.06E-05	0.056101337	0.58189	-0.181383953	0.076264
ADAT2	-0.826156412	1.14E-05	-0.830083269	9.20E-06	-0.562466835	0.00244
CRISPLD2	1.276438209	1.13E-05	0.721514708	0.0132	0.923644489	0.001496
RDX	0.564529062	1.17E-05	0.30971768	0.01607	0.501883859	9.44E-05
TEX9	-0.758003691	1.17E-05	-0.351350572	0.03957	-0.453566961	0.008034
BCAT2	-0.720079014	1.18E-05	-0.711943288	1.30E-05	-0.795713504	1.14E-06

MYADM	0.659512254	1.19E-05	-0.096140029	0.52631	0.150598888	0.319366
BCL2L1	0.717145679	1.21E-05	0.166266517	0.31126	0.735483514	7.01E-06
ANO5	-0.874728283	1.22E-05	-0.471877879	0.01589	-0.502077863	0.010873
GK	1.320617427	1.22E-05	1.527620946	4.07E-07	1.598155996	1.14E-07
HLTF	-0.648660966	1.22E-05	-0.266966359	0.07033	-0.302622584	0.040291
WDR74	-0.815607153	1.22E-05	0.166530307	0.37162	-0.324937754	0.081301
IL18R1	1.058961584	1.25E-05	1.376196275	1.16E-08	0.789314138	0.001121
KRTCAP3	-0.842478399	1.26E-05	-0.34030538	0.06975	-0.516923544	0.006099
VCAM1	2.716676685	1.28E-05	2.003125266	0.0013	2.994082292	1.51E-06
NEURL3	3.08237699	1.29E-05	3.058076371	1.42E-05	3.095600016	1.09E-05
SH3GL1	0.470639724	1.30E-05	0.066063792	0.54201	0.057146267	0.59773
BDKRB2	1.966028203	1.31E-05	1.326933755	0.00337	1.565881212	0.000522
ELK1	0.559827022	1.31E-05	0.180835395	0.16285	-0.023786398	0.854787
MANBA	-0.748573885	1.31E-05	-0.556628526	0.00114	-0.627805848	0.000247
PCDHGB6	0.841864418	1.34E-05	0.224832585	0.24975	0.429524153	0.026899
SLC25A46	-0.370897711	1.35E-05	-0.060648554	0.47292	-0.110783366	0.18991
ADRBK2	0.940355525	1.37E-05	0.575250065	0.00787	0.980556025	5.59E-06
SP3	-0.263245013	1.40E-05	-0.079698358	0.18522	-0.035266079	0.557183
LRRC40	-0.35918451	1.41E-05	-0.187284352	0.02042	-0.017090211	0.830221
IARS	-0.411740295	1.43E-05	-0.192278688	0.04233	-0.166704416	0.0783
SPATA2	0.711469178	1.45E-05	0.084476044	0.6177	0.173927469	0.298745
CENPU	-0.81379286	1.48E-05	-0.454785282	0.01426	-0.361598568	0.050897
PITHD1	-0.433491482	1.49E-05	-0.21591384	0.02806	-0.240826223	0.014213
THUMPD2	-0.635125596	1.50E-05	-0.505391093	0.0005	-0.418126019	0.003643
CCSAP	-0.366574525	1.51E-05	-0.03850967	0.63667	-0.093502455	0.250894
WSB2	0.469730425	1.51E-05	0.235135143	0.03025	0.271795205	0.01221
NUP133	-0.402242655	1.53E-05	-0.169040076	0.06628	-0.058879464	0.521342
AEBP1	0.982687828	1.54E-05	0.478218401	0.03548	0.526944129	0.020573
AGL	-0.362374538	1.54E-05	-0.096513698	0.24472	-0.178787266	0.031451
CENPH	-0.78888077	1.58E-05	-0.570357205	0.00149	-0.302548258	0.088382
GALNT7	-0.576408669	1.58E-05	-0.469874936	0.00042	-0.548031713	3.90E-05
SOCS3	1.668618432	1.58E-05	1.125392973	0.00361	1.697953445	1.11E-05
RGP1	0.408426289	1.59E-05	0.1558647	0.09975	0.292309183	0.001952
RP4-669L17.	1.363190427	1.59E-05	0.463018594	0.15039	0.369410547	0.251831
GXYLT2	1.128849132	1.62E-05	1.262225541	1.33E-06	0.730009114	0.005401
TGDS	-0.559829838	1.63E-05	-0.253472286	0.04459	-0.252091116	0.044413
DMXL1	0.498706906	1.65E-05	0.599869569	2.04E-07	0.667037437	7.47E-09
NR0B2	-1.315513801	1.65E-05	-1.346574459	9.96E-06	-1.269789617	3.02E-05
RNASEH2A	-0.570721234	1.66E-05	-0.271857731	0.03567	-0.275026564	0.033162
WIPI1	0.553326948	1.66E-05	0.54143434	2.36E-05	0.3471324	0.00679
PXK	0.529359317	1.67E-05	0.140913694	0.25497	0.272781997	0.025987
PCCA	-0.662618344	1.68E-05	-0.164535931	0.28196	-0.310163562	0.042609
UBE2E2	0.607610216	1.68E-05	0.351832942	0.01293	0.444299651	0.001562
RPL9	-0.681423846	1.69E-05	-0.440458753	0.0054	-0.514255715	0.001161
WISP1	1.237188332	1.72E-05	0.482960225	0.094	0.89069803	0.001969
CADPS2	-0.962185794	1.75E-05	-0.648796346	0.00357	-1.042589099	3.10E-06
FAHD2CP	-1.244879343	1.76E-05	-0.681582643	0.01402	-1.255965244	1.06E-05

PDE8A	0.599771442	1.77E-05	0.691292244	6.97E-07	0.635107197	5.17E-06
ITGAV	0.751600463	1.79E-05	1.096336533	3.85E-10	0.906661032	2.26E-07
RRM1	-0.52779715	1.79E-05	-0.254114252	0.03783	-0.115521454	0.344499
KDELC2	-0.530550771	1.82E-05	-0.203655832	0.0979	-0.510949463	3.39E-05
GABARAPL1	0.84629437	1.83E-05	0.371240374	0.06049	0.825670694	2.81E-05
VCL	0.467314153	1.83E-05	0.020572594	0.85051	0.347729582	0.001425
DBT	-0.53654859	1.90E-05	-0.215328863	0.08464	-0.246417706	0.048958
DSN1	-0.684842837	1.90E-05	-0.279354872	0.07494	-0.280276988	0.074419
NMD3	-0.392355201	1.93E-05	-0.188169374	0.03849	-0.183443537	0.043395
GNL3	-0.518122025	1.99E-05	-0.17806259	0.14075	-0.215922676	0.073985
MBNL1	0.422572507	2.01E-05	0.229840251	0.02016	0.261488118	0.008201
NR2C2AP	-0.854341097	2.01E-05	-0.448418565	0.02086	-0.472679133	0.015042
UTRN	-0.624283427	2.02E-05	-0.330034154	0.02392	-0.077299409	0.596492
LCN15	3.622785171	2.06E-05	1.237110048	0.14841	-0.040174718	0.962795
TMEM128	-0.662838919	2.07E-05	-0.051408105	0.73031	-0.183506063	0.21985
ATP13A3	-0.54681781	2.11E-05	-0.449874891	0.00046	-0.480687459	0.000182
SEC11C	-0.554226464	2.15E-05	-0.151036947	0.23981	-0.347094926	0.007058
DOC2B	4.54943835	2.15E-05	4.187125342	9.75E-05	4.354377988	4.88E-05
AC131180.1	-8.006798869	2.19E-05	-1.582578546	0.32134	-1.402525196	0.379064
AGR3	-0.826007669	2.23E-05	-0.509352852	0.00867	-0.594667675	0.002179
RAD1	-0.469774803	2.26E-05	0.036316738	0.73899	-0.043344273	0.690162
RPS18	-0.553249246	2.36E-05	-0.243837261	0.06221	-0.381266749	0.003551
ETFB	-0.569142264	2.39E-05	-0.402396158	0.00272	-0.4922352	0.000248
KRT6B	4.78955966	2.37E-05	5.232378929	3.84E-06	4.569262414	5.53E-05
MCEE	-0.895944307	2.38E-05	-0.351586782	0.0844	-0.172945171	0.388247
MGAT4B	0.436818737	2.37E-05	0.276258856	0.00744	0.105500337	0.308051
SKIV2L2	-0.339485574	2.38E-05	-0.097749301	0.21949	-0.035451299	0.655158
ASS1	1.453945168	2.40E-05	1.970443722	9.47E-09	1.011059497	0.003337
MUT	-0.344798671	2.41E-05	-0.054189546	0.49628	-0.023405947	0.767985
CCNI	0.460334823	2.45E-05	0.167622819	0.1246	0.186105319	0.088029
UNC119	0.535349568	2.45E-05	0.413770351	0.00108	0.38800899	0.002184
CDC42BPB	0.362820417	2.48E-05	0.150984818	0.07955	0.171599438	0.045957
ALG5	-0.451130231	2.49E-05	-0.110243189	0.29266	-0.293120532	0.005395
STK40	0.50781574	2.50E-05	0.213665828	0.07724	0.34746004	0.003974
SVIP	-0.664161853	2.50E-05	-0.368917458	0.01811	-0.565648814	0.000293
POU2F2	2.610235987	2.51E-05	2.213215031	0.00037	2.099454748	0.000731
PTGES	1.691356467	2.51E-05	1.017388277	0.01143	0.966620261	0.016262
ARHGAP26	0.836290869	2.53E-05	0.583475756	0.00325	0.867280427	1.20E-05
CD69	2.014266205	2.56E-05	1.224282615	0.01065	2.188582531	4.66E-06
BCL9L	1.092898723	2.58E-05	-0.124493236	0.63386	0.527041168	0.043499
LRRC45	-0.778013455	2.58E-05	-0.343484349	0.05611	-0.762749634	3.41E-05
EIF2S1	-0.448383751	2.62E-05	-0.096134421	0.36476	-0.09830901	0.3532
RPS3A	-0.522090509	2.63E-05	-0.29051694	0.0193	-0.264479805	0.033166
ATAD2	-0.57112469	2.66E-05	-0.281214799	0.03783	-0.138937802	0.304232
ECHS1	-0.427529334	2.67E-05	-0.118657731	0.2373	-0.321431405	0.001426
AKR1A1	-0.389090322	2.72E-05	-0.086028258	0.34877	-0.127987823	0.163907
C10orf32	-0.626010226	2.71E-05	-0.302232881	0.03958	-0.331749598	0.024203

GRB14	-1.157058967	2.72E-05	-0.871754572	0.00151	-0.917711927	0.000721
NDST2	0.774442339	2.72E-05	0.440187941	0.01729	0.40699331	0.027183
ASF1B	-0.730879989	2.78E-05	-0.393649293	0.02179	-0.622135053	0.000306
TMEM101	-0.512043202	2.80E-05	-0.447637677	0.0002	-0.381943024	0.001419
ARHGAP29	-0.67879364	2.81E-05	-0.556584285	0.00057	-0.642589059	6.96E-05
FNBP1	0.505808102	2.81E-05	0.519934728	1.74E-05	0.547587635	5.69E-06
MAPK1IP1L	0.736581728	2.85E-05	-0.074776687	0.67105	0.313935866	0.074246
TMX1	-0.403018767	2.86E-05	-0.090392656	0.34326	-0.106802068	0.262235
ACAA1	-0.578132395	2.94E-05	-0.387430905	0.00479	-0.266209389	0.052013
NRP2	0.999232888	2.92E-05	0.393724604	0.10023	0.527600516	0.027466
OAT	-0.600057743	2.94E-05	-0.504974993	0.00041	-0.583187161	4.65E-05
RPL36	-0.440771015	2.94E-05	-0.113360821	0.2811	-0.331628916	0.00163
RPS12	-0.545064536	2.93E-05	-0.065411851	0.61555	-0.279317144	0.032059
ZNF146	-0.336818018	3.02E-05	-0.208040359	0.00959	-0.035359864	0.658809
ACTN1	0.450678794	3.04E-05	0.16892686	0.11811	0.329701419	0.002271
HIVEP1	0.602153405	3.10E-05	0.024760091	0.86454	0.441276117	0.002234
PLA2G12A	-0.781499774	3.11E-05	-0.414107116	0.02645	-0.323958174	0.082786
MOCOS	-1.308460154	3.14E-05	-0.448310506	0.15118	-0.844818178	0.006984
RNF141	-0.423256354	3.19E-05	-0.197678628	0.04927	-0.276712993	0.005909
ZDHHC4	-0.538528481	3.21E-05	-0.572594769	8.58E-06	-0.346168069	0.006989
CHKB-CPT1I	0.770572929	3.29E-05	0.346642482	0.06182	0.658683205	0.000344
SYDE1	1.030614041	3.30E-05	0.19024825	0.44588	0.470707654	0.058434
GPN2	1.193870965	3.30E-05	0.398135156	0.1662	0.886140055	0.002034
SULT1C4	1.150557605	3.34E-05	-0.047185175	0.86855	0.930817538	0.00079
RFC4	-0.786938398	3.40E-05	-0.365135422	0.05203	-0.262362957	0.161233
TXNRD3	-0.590097036	3.40E-05	-0.108229091	0.42078	-0.59712264	1.69E-05
ZBTB7A	0.614514163	3.41E-05	0.225590367	0.12975	0.139408403	0.349506
SLC39A13	0.588043149	3.45E-05	0.148892119	0.29744	0.050368744	0.724227
AGPAT3	0.414075501	3.47E-05	0.430997606	1.56E-05	0.183925356	0.066125
CYB561	-0.779468378	3.50E-05	-0.49887482	0.00741	-0.876829116	2.89E-06
ATP6V0C	1.232779578	3.51E-05	0.667971578	0.02557	0.748479298	0.012228
ELOVL5	0.648687175	3.53E-05	0.48064384	0.00218	0.835760115	9.36E-08
TPRKB	-0.493112319	3.61E-05	-0.126912166	0.27	-0.105403086	0.357511
EHBP1L1	0.680990553	3.64E-05	0.285154377	0.08374	0.367060457	0.025849
RHOT2	0.525627705	3.68E-05	0.107593556	0.40028	0.05969536	0.64097
SLC44A1	-0.575556779	3.71E-05	-0.403066242	0.00381	-0.530741987	0.000139
CEP44	-0.563751928	3.87E-05	-0.183147574	0.1741	-0.234700884	0.080767
CPSF1	0.784504928	3.86E-05	-0.074459201	0.70198	0.256761862	0.180673
LRRC8A	0.484827945	3.87E-05	0.340234135	0.00385	0.288824118	0.014192
IL17C	3.632521577	3.96E-05	2.990042213	0.00074	2.927593212	0.00095
IL23A	2.52230968	3.96E-05	2.055687795	0.00089	3.073282027	3.57E-07
MRPL13	-0.544709057	3.99E-05	-0.093997977	0.46965	-0.199052	0.125798
PQLC3	-0.784084467	4.05E-05	-0.199988033	0.28176	-0.660913319	0.000424
F2R	0.700179671	4.10E-05	0.483482521	0.00462	0.635923934	0.000193
KIAA1919	-0.454657018	4.10E-05	-0.300576954	0.00593	-0.45029859	4.02E-05
UBALD2	1.165636567	4.11E-05	0.377713846	0.18504	0.43528347	0.127191
NDUFB3	-0.444297723	4.13E-05	-0.097996793	0.35542	-0.151078738	0.154336

BCAR1	0.703551829	4.18E-05	0.187788327	0.27495	0.497421822	0.003809
ENKUR	2.437664284	4.20E-05	2.757409169	3.23E-06	1.893197062	0.001547
ERMP1	-0.457085833	4.20E-05	-0.493710219	9.14E-06	-0.609803149	4.43E-08
SMC2	-0.470228564	4.21E-05	-0.082802779	0.46772	-0.109563791	0.336411
TOM1	0.871051221	4.24E-05	0.592166665	0.00543	0.817374054	0.000119
ATP13A2	0.652293038	4.35E-05	0.345625231	0.03036	0.301009046	0.059751
IRAK3	1.777228976	4.36E-05	1.770113874	4.62E-05	1.363335988	0.001726
SETD5	0.443699123	4.37E-05	0.144795681	0.1827	0.325202867	0.002728
CAPZB	0.306691102	4.42E-05	0.155961332	0.03767	0.249170603	0.000859
LRRC58	-0.322358929	4.47E-05	-0.231324152	0.00315	-0.231348454	0.003108
VPS18	0.399559855	4.48E-05	0.32676666	0.00082	0.358121569	0.000221
RPS27L	-0.55401488	4.50E-05	-0.196605822	0.14612	-0.326718016	0.015762
MRPS14	-0.462603815	4.53E-05	-0.190432242	0.08682	-0.11589653	0.292864
HIST1H4C	-0.72553868	4.56E-05	-0.182216578	0.30468	-0.072381616	0.683358
BCKDHB	-0.541817935	4.59E-05	-0.437268317	0.0009	-0.422114608	0.001406
NLGN3	1.909281911	4.60E-05	1.56284818	0.001	1.054746252	0.027788
NCOA7	0.949727855	4.61E-05	1.040241512	7.87E-06	0.92405427	7.25E-05
MBOAT2	-0.680599458	4.69E-05	-0.386774103	0.0203	-0.461462295	0.005638
TCF19	-0.723896497	4.71E-05	-0.30797694	0.07888	-0.466253356	0.00791
CHAF1B	-0.6279341	4.74E-05	-0.536042994	0.00044	-0.42136547	0.005515
MICU2	-0.342845404	4.73E-05	-0.072859344	0.37784	-0.126832757	0.124204
KIAA1324	-2.19358513	4.76E-05	-1.502181111	0.00497	-2.63174098	1.21E-06
SLC38A4	-1.135369636	4.78E-05	-0.672630687	0.01514	-1.014447811	0.000263
OSGEPL1	-0.664500968	4.85E-05	-0.521870775	0.00109	-0.29113893	0.064075
PRICKLE2	0.86444935	4.86E-05	0.430560819	0.04333	0.490603552	0.021154
CRLS1	-0.438922656	4.90E-05	-0.120331516	0.2572	-0.153630577	0.148634
SESTD1	0.569826406	4.90E-05	0.493943451	0.00043	0.645969696	3.98E-06
SH2D1B	4.620154233	4.92E-05	3.260941718	0.00497	3.853440047	0.000796
EEF1A1P6	-0.861823018	4.94E-05	-0.630409941	0.00286	-0.841590017	7.04E-05
STYK1	-1.197504962	4.95E-05	-0.680175351	0.02048	-1.318464927	7.77E-06
NUDT15	-0.398918657	4.96E-05	-0.227601003	0.01843	-0.062151247	0.515842
ACSL4	0.794687758	4.97E-05	0.941537626	1.53E-06	1.182045273	1.57E-09
CCDC138	-0.602393569	4.98E-05	-0.467248153	0.0014	-0.442348542	0.002445
RPL22	-0.438257418	5.00E-05	-0.20352157	0.05912	-0.269998777	0.01229
UBE2M	0.439649284	5.00E-05	0.178020467	0.09907	0.149366952	0.167646
CA8	1.32422422	5.03E-05	1.540620205	2.26E-06	1.306128601	6.25E-05
PPARG	-0.816691478	5.02E-05	-0.566707707	0.00485	-0.852075828	2.31E-05
STT3B	-0.325743805	5.04E-05	0.098193048	0.21973	-0.18786184	0.018991
MIPEP	-0.692647188	5.06E-05	-0.446647219	0.0083	-0.55671001	0.000998
NKX3-1	1.765761903	5.06E-05	0.098899542	0.82465	0.899282194	0.039796
C4orf21	-0.666542158	5.08E-05	-0.341942347	0.0361	-0.270187628	0.096856
TOR1AIP2	0.254564009	5.17E-05	0.203778014	0.00116	0.351320858	2.02E-08
CIAO1	-0.312392545	5.21E-05	-0.154316833	0.04267	-0.181948685	0.016808
MLXIP	0.639118566	5.23E-05	-0.032532136	0.83784	0.098754463	0.534015
BMPR1A	0.471831403	5.26E-05	0.409258575	0.00045	0.524989389	6.36E-06
GCNT1	-1.077023259	5.30E-05	-0.646001305	0.01526	-1.207275384	5.86E-06
GEM	1.061202334	5.31E-05	0.4694093	0.07424	1.263613813	1.44E-06

NOX1	2.172403175	5.30E-05	2.593614375	1.05E-06	2.127118137	6.67E-05
GPX3	1.342196531	5.34E-05	0.924908118	0.00559	1.460337593	1.03E-05
SLC4A4	-0.761749098	5.35E-05	-0.735312497	9.47E-05	-0.483445433	0.010228
CA5B	0.902148671	5.38E-05	0.791089082	0.00039	1.008934509	5.85E-06
TMCO1	-0.32386302	5.42E-05	-0.173649769	0.02852	-0.171273309	0.030572
USP32P2	1.013359366	5.44E-05	0.676091018	0.00702	0.96860812	0.000104
PGRMC1	-0.384046454	5.51E-05	-0.212020207	0.02553	-0.31264025	0.000996
PRCC	0.59669524	5.52E-05	-0.064492607	0.66553	0.190979054	0.197758
SSX2IP	-0.477945221	5.51E-05	-0.288696551	0.01397	-0.133825293	0.252485
NXF1	0.612478846	5.53E-05	0.089491234	0.55679	0.30421727	0.044853
DIS3L	-0.471547937	5.55E-05	-0.177031515	0.1266	-0.225357547	0.051036
HIVEP2	0.781103034	5.60E-05	0.422087063	0.02962	0.760890647	8.57E-05
BMPR2	0.607422994	5.63E-05	0.464923581	0.00205	0.474673849	0.001646
CDC14B	-0.556003544	5.65E-05	-0.293351976	0.03282	-0.299530753	0.029022
CNOT3	1.033375642	5.73E-05	0.272915962	0.2915	0.188503735	0.468604
SRFBP1	-0.515064715	5.76E-05	-0.278793068	0.02743	-0.19351823	0.124682
GIT2	0.474765368	5.82E-05	0.352029744	0.00287	0.349829059	0.002959
PMS1	-0.585224717	5.82E-05	-0.247740507	0.08549	-0.125267249	0.384001
GOT1	0.446071366	5.87E-05	0.230812167	0.03653	0.502204349	4.65E-06
HSPB11	-0.453507644	5.98E-05	-0.117079981	0.28329	-0.21505867	0.049222
THYN1	-0.467661121	6.12E-05	-0.084729863	0.45717	-0.122829587	0.280404
MRPL40	-0.506823253	6.14E-05	-0.214991635	0.08094	-0.321175204	0.009381
TMEM65	-0.325891891	6.14E-05	-0.099867794	0.20865	-0.119462172	0.131828
MYEF2	-0.632773496	6.21E-05	-0.336359665	0.03236	-0.045218341	0.772134
H2AFZ	-0.617545975	6.26E-05	-0.199243919	0.19517	-0.13572393	0.377339
STXBP6	-0.79574914	6.26E-05	-0.577596481	0.00342	-1.052635533	1.16E-07
RASGRP1	1.198289083	6.31E-05	1.581273795	1.10E-07	0.649429917	0.030477
CMC1	-0.751435629	6.32E-05	-0.493882148	0.00822	-0.734098836	7.44E-05
CHI3L1	2.943779914	6.41E-05	2.774033452	0.00017	2.34544466	0.001514
SF3B14	-0.362965301	6.50E-05	0.101136746	0.25587	0.002776704	0.975144
FBP1	-1.193819798	6.54E-05	-0.585734987	0.046	-1.420106813	2.10E-06
MAMLD1	1.74975069	6.54E-05	0.435779832	0.34509	1.023401972	0.022399
NDUFA10	1.204436497	6.53E-05	0.358524911	0.23725	0.657583759	0.029431
UBE2V1	0.391041256	6.73E-05	0.007546505	0.93889	0.245620944	0.012163
CDCA7	-0.807345294	6.75E-05	-0.840170902	3.19E-05	-0.686634329	0.000658
ANKRD13D	0.577928552	6.82E-05	0.250050028	0.08696	0.367742126	0.011248
PQLC1	0.563874124	6.82E-05	0.446835642	0.00162	0.080440984	0.572222
LDB2	-0.844361216	6.93E-05	-0.499556336	0.01749	-0.78668826	0.00019
PIK3AP1	1.394076318	6.98E-05	1.78043157	3.69E-07	1.900264866	5.73E-08
TRNP1	-0.837547218	7.03E-05	-1.204326114	1.11E-08	-0.832161158	7.80E-05
PEX7	-0.666253952	7.04E-05	-0.282520283	0.0732	-0.449247801	0.00472
HMGCL	-0.485718954	7.09E-05	-0.204435066	0.08958	-0.369719753	0.002256
TNFSF15	1.062355114	7.18E-05	0.568296946	0.03421	0.648669515	0.015467
STAT3	0.509262629	7.21E-05	0.40340431	0.00166	0.368502439	0.004065
PHF5A	-0.446125511	7.26E-05	-0.121642249	0.26755	-0.170753284	0.118494
KSR1	0.844941558	7.30E-05	0.100756396	0.63742	0.33974593	0.111434
MOB3A	0.59946299	7.32E-05	0.006099704	0.96818	0.149688798	0.324697

TLN1	0.540761799	7.36E-05	0.057201372	0.67501	0.363519696	0.007681
FANCI	-0.54591367	7.50E-05	-0.263055317	0.05516	-0.299433056	0.02878
IFI16	1.090389154	7.56E-05	1.083178288	8.38E-05	0.878157103	0.001432
PATL1	0.49841503	7.61E-05	-0.035805984	0.77843	0.066174362	0.601114
EFR3A	-0.294340242	7.68E-05	-0.017905216	0.8076	-0.126377638	0.086503
RC3H1	0.561592978	7.71E-05	0.181595626	0.20103	0.60089838	2.26E-05
MAPK7	0.791884036	7.78E-05	0.489142818	0.01507	0.693555845	0.000548
KIAA1147	-0.33337689	7.80E-05	-0.06189698	0.45742	-0.258007412	0.002026
HNRNPA3	0.527197217	7.86E-05	-0.199669039	0.13519	0.129472979	0.332149
MZT1	-0.539416565	7.86E-05	-0.069539787	0.59845	-0.12976126	0.325849
ANAPC16	-0.434726165	7.89E-05	-0.307035743	0.00489	-0.27855565	0.010639
CHD1L	-0.432653739	7.96E-05	-0.230119402	0.03454	-0.136766988	0.207275
RHBDD2	0.491524325	7.98E-05	0.095397991	0.44555	0.190281722	0.128261
FMO5	-0.714956778	8.11E-05	-0.575007535	0.00139	-0.679413454	0.000164
LRRCC1	-0.71200343	8.11E-05	-0.689544913	0.00012	-0.281729996	0.113897
COX11	-0.368194271	8.13E-05	-0.346646792	0.00017	-0.326155354	0.000399
PKD1	0.69559477	8.19E-05	0.040652563	0.81847	0.077590497	0.661503
)C1S5-TXNI	-1.035946307	8.22E-05	-0.351574461	0.17991	-0.655303499	0.012505
MTATP6P1	1.261052453	8.23E-05	0.51476323	0.1102	0.309000135	0.338715
GATM	-0.74590676	8.27E-05	-0.555110306	0.00332	-0.726049493	0.000124
ZKSCAN1	-0.432432693	8.28E-05	-0.270159996	0.01328	-0.30416102	0.0054
B3GNT2	0.532258636	8.36E-05	0.950149706	1.37E-12	0.765911876	1.18E-08
P11-958N24	1.722030103	8.44E-05	1.07864416	0.01488	0.910624268	0.038545
ELL	0.674624596	8.54E-05	0.141382297	0.41072	0.241665306	0.159846
TMEM139	0.873750433	8.61E-05	0.993544106	7.07E-06	0.553609764	0.013087
CHMP4B	0.319438031	8.71E-05	0.282104957	0.0005	0.300092801	0.000204
IGLON5	1.025271115	8.70E-05	-0.132594109	0.62311	0.409918454	0.120406
CDC5L	-0.267457725	8.74E-05	-0.173320621	0.01034	0.018006494	0.788613
YEATS4	-0.462182617	8.73E-05	-0.274420387	0.01694	-0.113896588	0.317032
DHRS7B	-0.470900842	8.84E-05	-0.061192153	0.60165	-0.21362379	0.068887
RPS27A	-0.493600357	8.89E-05	-0.174400292	0.16572	-0.241411525	0.055034
LETM2	1.007998298	9.05E-05	0.696216687	0.00665	0.728596921	0.004564
PSEN1	0.349509391	9.06E-05	0.28508523	0.00138	0.301959288	0.00069
DCP1A	0.724784169	9.08E-05	0.369800949	0.04836	0.368480893	0.047271
ACN9	-0.697188799	9.14E-05	-0.395782181	0.02275	-0.552945375	0.001555
C1orf112	-0.558098438	9.13E-05	-0.284506223	0.04378	-0.125632333	0.371128
NPTN	0.356576522	9.16E-05	0.120418479	0.18546	0.21862896	0.01619
AAK1	0.69996484	9.23E-05	0.432744391	0.01641	0.301647336	0.093889
ABCE1	-0.477057804	9.37E-05	-0.185324806	0.1277	-0.247736283	0.041599
CENPM	-0.861795492	9.36E-05	-0.558647447	0.00913	-0.67609967	0.001675
NHLRC3	-0.44204925	9.47E-05	-0.20379602	0.06966	-0.292104726	0.009149
EMP3	0.617440695	9.48E-05	0.180539287	0.25463	0.531439763	0.000762
FASTKD2	-0.435605262	9.54E-05	-0.228586005	0.03854	-0.224119499	0.042127
RPS15A	-0.420170694	9.59E-05	-0.202090901	0.06042	-0.195517724	0.06924
PPP1CC	-0.29731672	9.62E-05	-0.163734351	0.03104	-0.076918983	0.310428
MCM2	-0.623737957	9.74E-05	-0.476570715	0.00283	-0.542557508	0.000672
MEN1	0.395510047	9.87E-05	0.177734381	0.08165	0.182756754	0.071664

NFKB1	0.792042761	9.99E-05	0.430932721	0.03444	0.92331496	5.61E-06
SERPING1	0.858146314	0.0001	0.559845483	0.01126	0.557078901	0.011552
DHFR	-0.667348598	0.000101	-0.400401268	0.0193	-0.357233855	0.036767
FBRS	0.640860622	0.000101	0.307601973	0.06314	0.264527096	0.110372
LGR5	-1.723171916	0.000102	-0.919726775	0.03693	-1.89368787	2.03E-05
KIF20B	-0.573966426	0.000103	-0.224600416	0.1265	-0.092281822	0.529524
CDC42EP2	0.632788347	0.000104	0.503013814	0.00201	0.413660905	0.011103
PDCD6IP	0.365819157	0.000104	0.246164242	0.00892	0.397715151	2.37E-05
MTMR8	-0.987652552	0.000105	-0.301445184	0.2233	-0.933679903	0.000197
RPS23	-0.526872373	0.000106	-0.228205723	0.09303	-0.265118705	0.051015
TNF	4.563542268	0.000107	4.108541449	0.00063	4.796779311	3.93E-05
SUPT6H	0.308037662	0.000108	0.168777446	0.03376	0.25109948	0.001557
TBC1D31	-0.520027289	0.000109	-0.262429428	0.04575	-0.189220212	0.147115
CMBL	-0.883717486	0.00011	-0.606386023	0.00746	-0.885112689	0.0001
ANGPTL2	0.863120195	0.00011	0.63752788	0.00439	0.792523841	0.000377
GINS2	-0.795575443	0.00011	-0.36542317	0.07335	-0.473887609	0.020173
FERMT2	0.712126409	0.000112	0.490436307	0.00778	0.880640596	1.72E-06
CNBP	-0.30621875	0.000112	-0.222072441	0.00494	-0.202761799	0.010222
COL9A2	1.055109792	0.000112	1.353625613	6.23E-07	0.969372245	0.000374
TSPAN6	-0.368055976	0.000112	-0.184555595	0.05091	-0.239429703	0.011333
EFCAB3	1.515319674	0.000113	0.940583169	0.01883	1.131849227	0.004175
RPS14	-0.489480106	0.000113	-0.319732142	0.01161	-0.323590268	0.010632
FAF1	-0.476600849	0.000113	-0.328521977	0.00736	-0.281772571	0.021314
C14orf2	-0.396778195	0.000114	-0.198869862	0.05144	-0.283871704	0.005373
RPS27	-0.447488322	0.000114	-0.246079072	0.03372	-0.264041466	0.022699
ROBO1	0.493956503	0.000115	0.325659257	0.01098	0.526639612	3.84E-05
MRPL16	-0.427604178	0.000116	-0.191486853	0.07818	-0.185081719	0.088066
PHKA1	-0.635734849	0.000117	-0.39362594	0.01529	-0.343503225	0.033654
PHLPP2	0.624504489	0.000117	0.365339443	0.02413	0.389148077	0.015977
RNY4P13	-1.212954904	0.000117	0.323369353	0.23797	-0.167706443	0.550211
ASF1A	-0.434428377	0.000117	-0.06900349	0.53145	-0.138496281	0.208984
CH25H	1.770810819	0.000118	1.013056066	0.02903	0.831882801	0.07373
ANTXR1	0.770173786	0.000119	0.48977245	0.01445	0.429151561	0.032058
IFITM2	0.676774275	0.000119	0.345104642	0.0499	0.363479258	0.038841
AC040977.1	0.648697377	0.00012	0.24659349	0.14513	0.208410177	0.218091
GNA15	1.742006319	0.00012	1.989092197	1.07E-05	1.606546588	0.000387
PTPRK	0.344339919	0.00012	0.335347909	0.00017	0.258942137	0.003715
TRAPPC2L	-0.470927216	0.00012	-0.145947985	0.22296	-0.420626473	0.000489
BEND4	3.071026386	0.000121	2.76178368	0.00056	2.87170842	0.000321
REXO1	0.940475062	0.000122	0.202406227	0.40963	0.412549804	0.0932
STK35	0.276886791	0.000123	-0.050975035	0.48324	0.033114518	0.646057
C9orf114	-0.583732301	0.000125	-0.335163783	0.02643	-0.586575384	0.000102
CRNKL1	-0.464749473	0.000125	-0.151316854	0.20777	-0.11904256	0.320416
LRCH4	0.762175826	0.000125	0.104502199	0.60098	0.135307996	0.499838
DDX39B	0.328936526	0.000126	0.121540682	0.1565	0.355409832	3.27E-05
IER3IP1	-0.4604589	0.000126	-0.063339094	0.59433	-0.121112101	0.309195
CEP85L	-0.555216012	0.000128	-0.548508385	0.00015	-0.297547905	0.039229

FAM189B	0.72053746	0.000128	0.15438091	0.4167	0.013251283	0.944669
MSH6	-0.489221291	0.000128	-0.356724332	0.00511	-0.130879717	0.303781
TFEB	0.756198207	0.000128	0.803383068	3.60E-05	0.919688695	2.86E-06
ADTRP	1.575744781	0.000129	2.27394537	1.34E-08	0.943143897	0.023274
ARHGAP5	-0.4097401	0.000129	-0.146170845	0.1713	-0.291744385	0.006341
TK1	-0.664777248	0.00013	-0.531307332	0.00209	-0.564380516	0.001083
FBXL19	0.712817747	0.000131	0.214904141	0.24916	0.102943218	0.581228
PFN2	-0.312717746	0.000131	0.033880435	0.67619	-0.007114728	0.930112
TUBB2B	1.583278812	0.000131	1.304209835	0.00164	1.107058908	0.007546
PML	0.676986572	0.000131	0.061101011	0.73181	0.073860865	0.679052
NUP155	-0.409467657	0.000133	-0.39766106	0.0002	-0.287566742	0.007023
GRTP1	-0.817082805	0.000133	-0.529388029	0.01154	-0.827620487	9.66E-05
ADRM1	0.422925598	0.000134	0.236870471	0.03247	0.110805513	0.318051
ALG10B	-0.395884954	0.000134	-0.256276844	0.01238	-0.181716337	0.075853
RAB1A	0.338055484	0.000135	0.245829312	0.0054	0.226480712	0.010344
NRBP2	0.549329218	0.000136	0.121468016	0.4038	0.19086937	0.181845
TMEM184B	0.565550162	0.000136	0.091438104	0.53746	0.285379423	0.054155
FLII	0.472390002	0.000137	0.235473758	0.05711	0.270290006	0.028987
OSTC	-0.436612503	0.000137	-0.058460857	0.60738	-0.258875869	0.022997
EIF4G2	0.577140685	0.000138	0.244176758	0.10676	0.465568505	0.0021
PRRC2B	0.469012967	0.000138	0.099017439	0.42146	0.062640839	0.610853
ARHGEF28	-0.954990541	0.000138	-0.430807467	0.08452	-0.883205828	0.00041
ALDH1A1	-0.685190796	0.00014	-0.465517051	0.00963	-0.455963041	0.011213
AP3D1	0.439092291	0.00014	0.169411533	0.14164	0.237313233	0.039488
DUSP7	0.653117336	0.00014	0.137854761	0.42965	-0.079000222	0.653749
MYEOV2	-0.563234416	0.00014	-0.045664393	0.7477	-0.468379251	0.001202
PSMC4	0.461329431	0.000141	0.459956837	0.00014	0.438647306	0.000281
CENPQ	-0.654805254	0.000144	-0.248286511	0.1344	-0.182172339	0.268888
MTRF1	-0.533896695	0.000146	-0.26467346	0.05503	-0.210182322	0.125409
STYXL1	-0.436967437	0.000146	-0.204230125	0.06706	-0.41790202	0.000196
PPAT	-0.564977338	0.000148	-0.320927243	0.03008	-0.226959291	0.124545
GPX2	0.789934092	0.000148	0.635929107	0.00225	0.770184334	0.000215
TJP1	0.501058057	0.000148	0.113730685	0.38935	0.335156496	0.011048
KNTC1	-0.511098115	0.00015	-0.299091566	0.02585	-0.162002855	0.2267
SSBP1	-0.318462741	0.00015	0.066036104	0.42233	-0.010022783	0.903028
RNF38	0.603239899	0.000151	0.146187774	0.35897	0.383301764	0.015801
RPE	-0.380272396	0.00015	-0.09911197	0.31764	-0.247357111	0.012795
CIZ1	0.480729878	0.000151	0.046588695	0.7145	0.205524113	0.105289
MTHFS	-0.717252971	0.000152	-0.293199733	0.11495	-0.640905746	0.000621
PLK4	-0.593638191	0.000152	-0.34150845	0.02783	0.023039693	0.881599
PRTFDC1	-0.714140414	0.000154	-0.250348355	0.17678	-0.252779169	0.170185
ETS1	0.582635316	0.000155	0.344358825	0.02541	0.389696422	0.011317
HLA-DQB1	1.521710333	0.000155	0.907188378	0.02451	0.43144161	0.286875
SPRR1B	3.559524678	0.000157	4.31917021	3.59E-06	2.819782917	0.003
NINJ1	0.81099563	0.000157	0.372774285	0.08331	0.498156335	0.020381
NUDCD2	-0.546957874	0.000158	-0.214902005	0.13536	-0.2394982	0.095507
PHGDH	-0.432385335	0.000158	-0.310702074	0.00633	-0.358406357	0.001634

MT-CYB	0.907656998	0.00016	0.133845949	0.57799	-0.001297218	0.995698
MTND4P12	0.970833217	0.00016	0.085479192	0.74105	0.155190925	0.548053
PTBP1	0.485991881	0.00016	0.056932253	0.65834	0.118674285	0.356523
STX8	-0.416111357	0.00016	-0.283433596	0.00866	-0.112743926	0.291038
SARNP	-0.35829512	0.000161	-0.141019285	0.13181	0.111961729	0.22735
JUP	0.617846066	0.000161	0.486553899	0.00296	0.276065473	0.091856
METTL12	-0.888831962	0.000162	0.284326438	0.22139	-0.097256094	0.6766
WASF1	0.714689977	0.000165	0.175197581	0.35976	0.527451506	0.005268
IRAK1BP1	-0.831158769	0.000166	-0.116678196	0.59004	-0.203022329	0.349589
AREL1	0.437589831	0.000166	0.196979446	0.08927	0.318364043	0.005933
RPS4XP22	-0.866294083	0.000166	-0.142252999	0.50425	-0.219049678	0.304299
COX15	-0.392163429	0.000168	-0.122510244	0.23403	-0.279500997	0.006814
ALPK3	0.688783507	0.000168	0.489467029	0.00752	0.478110743	0.009033
EEF1A1P4	-0.753520843	0.000169	-0.119958893	0.51944	-0.357398485	0.058251
HDAC7	0.657740043	0.000169	0.376131297	0.03155	0.421536147	0.015856
SENP6	-0.254066373	0.00017	-0.07750865	0.24798	-0.016938982	0.800251
ZEB2	1.014788304	0.000171	0.482130096	0.07456	0.836671031	0.001935
PAIP2B	-0.46593349	0.000174	-0.320535345	0.00861	-0.296251903	0.014792
CENPE	-0.843263619	0.000174	-0.377858785	0.09203	-0.10166749	0.650133
PI4KB	0.437977633	0.000175	0.196550866	0.0914	0.243911688	0.036139
C7orf43	0.601983242	0.000175	0.298529904	0.0636	0.538131728	0.000837
DEK	-0.504464174	0.000175	-0.243777211	0.06913	-0.064996935	0.627809
AC004057.1	1.480206383	0.000176	0.905389494	0.02184	1.208425847	0.002199
ZNF703	0.687048039	0.000176	-0.272394996	0.1522	-0.083192038	0.657447
RP11-762I7.5	1.357839369	0.000177	0.875902115	0.01598	0.607559633	0.095495
NDRG4	0.879471857	0.000177	0.346613329	0.14083	0.525916376	0.023884
FUT2	0.603213527	0.000177	0.721501727	7.01E-06	0.368703322	0.021883
TUFM	-0.575193848	0.000178	-0.208335174	0.17373	-0.43234694	0.004781
IGDCC4	0.903894495	0.000179	0.343194245	0.15626	0.422530836	0.080917
C6orf211	-0.412660027	0.000182	-0.133990657	0.21894	-0.138971947	0.201331
TD-2005D20	-2.475820074	0.00018	-1.012753087	0.06774	-1.878815628	0.001576
HIPK1	0.362057333	0.000181	0.127052294	0.18835	0.197959985	0.040292
LSM3	-0.445050414	0.000182	-0.186194521	0.11312	0.013156528	0.9104
MAPKAPK2	0.285553194	0.000181	0.198863966	0.00883	0.160505716	0.034614
PSPC1	-0.555659533	0.000181	-0.18576037	0.20505	0.015239976	0.916832
RPL31	-0.470007451	0.000182	-0.208799482	0.09611	-0.341435319	0.006511
SH2B3	0.828173414	0.000182	0.662039667	0.00278	0.62734896	0.004543
SUSD4	-1.227370509	0.000181	0.420222147	0.18106	-1.489203083	6.65E-06
ZFAND1	-0.479845236	0.000182	-0.109160877	0.38885	-0.163628111	0.195754
CLCN6	0.606380192	0.000183	-0.065872674	0.68679	0.396002183	0.014609
ELMO2	0.497037262	0.000184	0.380969526	0.004	0.426968379	0.001232
FABP5	-0.668798467	0.000184	-0.529262639	0.00301	-0.415649802	0.019674
RPA3	-0.639352377	0.000185	-0.364333922	0.02899	-0.415568318	0.012934
STEAP3	-0.895211679	0.000185	-0.563329502	0.01805	-0.955651255	6.34E-05
TMEM259	0.412313557	0.000185	0.102021264	0.35553	0.198831306	0.071739
EPRS	-0.454523014	0.000189	-0.02386538	0.84413	-0.266514575	0.028194
MFHAS1	0.555820091	0.00019	0.264313507	0.07644	0.384995904	0.009628

SP110	0.68960074	0.00019	0.086818196	0.64228	0.427257938	0.020452
RNF223	-1.031189752	0.000191	-0.548034367	0.04025	-1.090902965	6.67E-05
JARID2	0.418692301	0.000192	0.39317667	0.00044	0.424324631	0.000145
RARS2	-0.363969716	0.000194	-0.101673478	0.29125	-0.106172967	0.269808
PRRC2A	0.456357563	0.000195	0.163407819	0.18252	0.207115471	0.091048
P11-459D22	-1.725836162	0.000195	-0.838296042	0.04109	-0.627189666	0.118019
GJB6	-2.51581248	0.000197	-1.081438503	0.08084	-3.688600929	9.78E-07
NDUFAB1	-0.471428337	0.000197	-0.10621871	0.39432	-0.103723171	0.405057
DKK3	-0.791796717	0.000199	-0.248321216	0.23247	-0.357331597	0.087677
ATRAID	-0.364412858	0.000199	-0.242278226	0.01268	-0.307210106	0.001587
CASP5	2.111397195	0.000199	2.052654441	0.00029	1.937485033	0.000642
ATAD1	-0.356647763	0.000201	-0.247073643	0.00958	-0.221830546	0.019825
NHSL2	1.277923856	0.000202	0.148764587	0.67476	0.546528946	0.116116
EXOSC9	-0.46802296	0.000203	-0.226589839	0.06937	-0.006372715	0.959026
MATR3	-0.315552027	0.000203	-0.122940898	0.1471	0.015289892	0.856821
NAT10	-0.506160821	0.000203	-0.398630149	0.00328	-0.164551997	0.224416
XPO1	-0.329070409	0.000204	-0.083192722	0.34677	0.014351098	0.871002
SVIL	0.56580148	0.000205	0.338569914	0.02633	0.48854483	0.001336
FANCF	-0.451947992	0.000205	-0.167629463	0.15797	-0.244546056	0.039604
TMED3	-0.54476604	0.000206	-0.242117473	0.09815	-0.557184555	0.000144
SULT1C2	-1.189537067	0.000209	-1.170893916	0.00026	-1.277080555	6.70E-05
FAT1	0.500451098	0.000211	0.341325399	0.01148	0.529637673	8.75E-05
JTB	-0.386160338	0.00021	-0.081390127	0.42689	-0.295012287	0.00414
PLK3	0.794335596	0.00021	0.259133591	0.22912	0.425581272	0.047226
SLC28A2	-1.728652509	0.000211	-0.279230512	0.54649	-1.647711952	0.0004
SMIM6	-0.927706099	0.000211	-0.458585936	0.06098	-0.301864861	0.214575
IMP3	-0.438240221	0.000213	-0.003010351	0.9792	-0.155882459	0.178449
MAP4K4	0.470161806	0.000213	0.338230663	0.00771	0.518804302	4.30E-05
PIEZO2	0.591935904	0.000212	0.713790656	7.70E-06	0.530855222	0.000879
TTC9	0.69266673	0.000213	0.702952385	0.00016	0.468008902	0.012323
C1orf27	-0.357114906	0.000213	-0.05704206	0.54812	-0.021143931	0.823127
AL353671.3	-1.415809973	0.000214	-0.572523407	0.12179	-0.394368772	0.284163
PRDX3	-0.454169014	0.000214	-0.22264015	0.06841	-0.286913235	0.018858
C17orf62	0.434391514	0.000215	0.349705999	0.00286	0.275897586	0.018652
P11-1220K2	1.547510961	0.000215	1.194901807	0.00428	0.841921475	0.044221
IER5L	0.98221434	0.000215	0.404538763	0.12884	0.36589217	0.169521
RASA1	-0.321098145	0.000215	0.009389609	0.91307	-0.166975337	0.052845
PDLIM7	0.843803118	0.000216	0.312386866	0.17169	0.316166252	0.166541
AGPS	-0.461726228	0.000217	-0.153610429	0.2165	-0.302953153	0.014898
ARHGAP17	0.539215198	0.000217	0.199847248	0.17111	0.558263976	0.000127
GSK3A	0.549881557	0.000218	0.020730338	0.88954	0.292219838	0.049123
UBR4	0.493011221	0.000219	0.244187111	0.06718	0.455783445	0.000633
PLAT	0.962077717	0.000221	0.456806559	0.07954	0.672855049	0.009793
COPG2	-0.490615207	0.000222	-0.158600014	0.22482	-0.280964186	0.03186
NOP58	-0.402218833	0.000222	-0.276553205	0.01066	-0.070793843	0.511703
RSBN1L	-0.531532366	0.000224	-0.197627005	0.16721	-0.211361184	0.138872
TESK1	0.571265595	0.000225	0.128490619	0.41085	0.354043466	0.022704

WDR76	-0.857290961	0.000227	-0.498348226	0.03086	-0.548317911	0.017565
SNX29P2	1.02031957	0.00023	0.278491729	0.33647	0.673645797	0.015606
P11-155G14	1.325099223	0.000232	1.20409495	0.0008	1.380972601	0.000109
TPM1	0.499834421	0.000232	0.306061822	0.02417	0.620008827	4.90E-06
NBN	-0.368020631	0.000233	-0.131888864	0.1837	0.03987523	0.686948
CDC34	0.4481459	0.000234	0.193192976	0.11312	0.130066213	0.28671
ANXA10	-0.872010815	0.000234	-0.897449984	0.00015	-0.987193028	3.10E-05
NR1D1	-1.92998094	0.000234	-2.153587432	4.12E-05	-2.129816814	4.81E-05
SREBF2	0.454669504	0.000235	-0.034496241	0.78069	0.117838532	0.340985
ERI2	-0.397718357	0.000236	-0.224716942	0.03364	-0.267532988	0.011271
NIT2	-0.547305181	0.000237	-0.127130651	0.38952	-0.199916517	0.175817
DNHD1	0.812827864	0.000238	0.715546906	0.00124	0.427623611	0.053399
KIRREL	0.794497176	0.000238	0.326035842	0.13178	0.544326702	0.011794
RP11-257I8.:	-0.782570591	0.000238	0.1466004	0.43534	-0.320783471	0.10046
TSG101	0.460340593	0.000241	0.216360958	0.08392	0.232636306	0.063075
NACC2	0.441019253	0.000242	0.297371801	0.01317	0.271237475	0.02377
PIGF	-0.555393083	0.000243	-0.282109243	0.05809	-0.250703579	0.092162
SPR	-0.610537249	0.000243	-0.15547989	0.3394	-0.531652411	0.001221
GTPBP8	-0.515651276	0.000245	-0.220726199	0.10405	-0.22473035	0.096695
MFSD1	-0.460841648	0.000245	-0.04260786	0.72977	-0.3107537	0.012115
MSH2	-0.743585319	0.000244	-0.373718843	0.06371	-0.303856498	0.131279
WBP1L	0.284913351	0.000245	0.356811768	3.60E-06	0.169159674	0.028746
WWC2	0.495283099	0.000244	0.334799586	0.01323	0.440215408	0.001077
NRG4	-1.043052721	0.000246	-0.366297412	0.18119	-1.247539234	1.22E-05
ICMT	-0.39993773	0.000247	-0.145992366	0.17861	-0.274355606	0.011547
EML5	-1.229742308	0.000248	-0.320673838	0.32263	-0.117783194	0.714854
PI4K2A	0.423787215	0.00025	0.131361514	0.25845	0.305458885	0.008053
VPS36	-0.358226566	0.00025	-0.189768304	0.05094	-0.0988961	0.307142
PROX1	1.127016123	0.00025	1.291563341	2.62E-05	1.634121761	1.01E-07
CHCHD1	-0.530871311	0.000252	-0.155713711	0.27118	-0.24168134	0.087693
KAZN	0.728430585	0.000253	-0.090104616	0.6561	0.323156893	0.105612
SH3PXD2A	0.485461332	0.000255	0.229802619	0.08342	0.158813919	0.231727
CYP26B1	1.148643958	0.000256	0.534613424	0.09077	1.04912257	0.000823
GALNT13	-1.325425516	0.000256	-0.803775746	0.02591	-1.542531452	2.13E-05
RPL37A	-0.427265787	0.000256	-0.179983818	0.12325	-0.226274881	0.052652
ZNF277	-0.496824298	0.000258	-0.065102972	0.62666	-0.046890513	0.725774
NDUFB10	-0.372647033	0.000261	-0.215387538	0.03235	-0.195515635	0.051255
SLC44A5	-0.834934053	0.000261	-0.102191587	0.63883	-0.244474824	0.266906
CDC25A	-0.625953268	0.000263	-0.355211304	0.03676	-0.286904068	0.091411
GPR37L1	1.02498485	0.000264	1.009545074	0.00029	0.814469137	0.003692
PDHB	-0.273843408	0.000265	-0.035818826	0.62838	-0.072778806	0.324869
DCTN1	0.533688781	0.000266	0.138923007	0.34254	0.202259762	0.166766
PTCD3	-0.278532304	0.000268	-0.14324837	0.05857	-0.004679014	0.950623
UGT8	-0.674575735	0.000269	-0.545336188	0.00317	-0.516598576	0.005169
MT-ATP8	1.235663135	0.00027	0.799958004	0.01853	0.4027334	0.236998
ANO9	0.641972679	0.000271	0.57856086	0.00096	0.519595184	0.003085
PPP1R9A	-0.392218851	0.000271	-0.251220668	0.01906	-0.236655026	0.027099

TCEB2	-0.321513137	0.000272	-0.019583166	0.82268	-0.197009313	0.024456
FRAT2	-0.673866128	0.000272	-0.521717039	0.00435	-0.242449953	0.181057
TROVE2	-0.259580274	0.000274	-0.067313497	0.34333	-0.038733192	0.584741
LINC00888	-0.497133736	0.000275	-0.075175379	0.56698	-0.01750939	0.89253
PTPLAD1	-0.366937372	0.000282	-0.280484084	0.00543	-0.095988336	0.340783
IHH	-1.082669074	0.000284	-0.562387375	0.05858	-1.516068968	3.86E-07
FHL3	0.68610946	0.000287	0.09680193	0.61106	0.198361163	0.296175
SRSF10	-0.304568397	0.000287	-0.095803393	0.25214	0.043473731	0.602713
TCEA3	-1.045170736	0.000287	-1.029495646	0.00032	-1.379557219	1.99E-06
ZCCHC11	-0.37533842	0.000288	-0.076636156	0.45739	0.005743032	0.955461
CBL	0.446528819	0.00029	0.099599393	0.42008	0.230759725	0.060965
FAM193A	0.488841601	0.000289	0.060463058	0.65518	0.073055229	0.588369
SPINK1	-0.828634329	0.00029	-0.253789474	0.26687	-0.5436121	0.017401
RAD18	-0.45499244	0.00029	-0.344180129	0.00552	-0.199026351	0.106264
ODF3B	1.729695444	0.000292	1.731374014	0.00028	0.787373736	0.108267
MOB1B	-0.39230136	0.000296	-0.182863519	0.08888	-0.13111768	0.221144
MTRNR2L4	-1.015741274	0.000296	-0.017641511	0.94908	-0.544934363	0.049641
RRM2	-0.712334189	0.000297	-0.468846812	0.017	-0.459706031	0.019288
ITGB8	0.721133524	0.0003	0.533562317	0.00746	0.445584556	0.025456
MANEAL	-1.11805415	0.000302	-0.692340089	0.02112	-1.046880858	0.000547
MCAM	0.832154718	0.000302	0.394502189	0.08806	0.315886031	0.171145
SF1	0.420102539	0.000302	0.078560314	0.50043	0.21940238	0.059193
SNRPF	-0.529743625	0.000304	-0.214564802	0.13883	-0.064946971	0.653227
OAS1	0.654197309	0.000305	0.75916289	2.71E-05	0.497039555	0.006045
SLC9A3	1.119281385	0.000306	1.015562048	0.00107	0.975403335	0.00163
ARPC2	0.334625972	0.000307	0.254709586	0.00596	0.34508812	0.000193
TRMT2B	-0.429085534	0.000308	-0.432911073	0.00023	-0.554493076	2.53E-06
SPCS3	-0.27786027	0.000312	-0.043348793	0.57208	-0.075413394	0.325668
GOPC	-0.285278049	0.000313	0.064029208	0.41093	0.064796612	0.404756
CASP3	-0.376010928	0.000315	0.086249384	0.40157	-0.040553269	0.693695
HEG1	0.96105764	0.000317	0.797226046	0.00286	0.789179761	0.003139
SPATA5	-0.462283659	0.000318	-0.422622885	0.00092	-0.273733148	0.031251
CENPT	0.692862801	0.000319	0.525596422	0.00623	0.900432904	2.46E-06
TSEN15	-0.415028288	0.00032	-0.095462003	0.39734	-0.149812077	0.183346
ILK	0.423326594	0.000321	0.140810162	0.23121	0.34389506	0.003389
SDHD	-0.34597067	0.000321	-0.186986237	0.04976	-0.098050013	0.302012
EIF2AK1	-0.337007424	0.000322	-0.266666423	0.00429	-0.362778782	0.000103
PBK	-0.93783237	0.000322	-0.27886717	0.27912	-0.152220395	0.55393
BEND3	0.794993772	0.000324	1.113395586	4.45E-07	0.53203165	0.01613
ATG4C	-0.918193401	0.000325	-0.6227729	0.01327	-0.510709497	0.041955
SDC3	0.683299972	0.000326	0.307774059	0.10573	0.288634472	0.128925
TADA1	-0.385208868	0.000329	-0.193029258	0.0653	-0.116304344	0.262503
PITPNA	0.395696802	0.000329	0.203960664	0.06424	0.307085104	0.005269
TGM2	0.832848931	0.000331	0.377203396	0.10439	0.424293406	0.067636
TEAD3	0.642428138	0.000332	-0.076959056	0.66832	0.276266152	0.122573
FAM20B	-0.233888844	0.000332	-0.097143211	0.13167	-0.024149789	0.706733
NQO1	-0.632971649	0.000332	-0.622131935	0.00042	-0.369410813	0.035988

CNTNAP1	0.808008745	0.000334	0.572457209	0.01117	0.521187903	0.020806
SLC38A11	-1.716045262	0.000334	-0.686038038	0.13458	-1.253687293	0.007182
RMI2	-0.763019866	0.000336	-0.742171656	0.00044	-0.533371263	0.010984
NAV1	0.824317885	0.000337	0.263769565	0.25247	0.082749516	0.719825
PPCS	-0.427434137	0.000339	-0.213340444	0.07136	-0.397890323	0.000796
RCAN1	0.937421385	0.00034	0.726278612	0.0055	1.243888701	1.90E-06
GATAD2A	0.341845802	0.000341	0.076802439	0.42185	0.151869469	0.11188
HSPB1	0.591446701	0.000341	-0.009975615	0.95194	0.330073861	0.04573
GTF2H3	-0.466899956	0.000342	-0.344797298	0.00736	-0.286210976	0.026357
FOCAD	-0.481014452	0.000344	-0.084179925	0.52906	-0.15713322	0.239765
MRPL1	-0.57034944	0.000344	-0.210135846	0.18006	-0.322054728	0.040306
POFUT1	-0.461155911	0.000344	-0.252615005	0.0489	-0.409456604	0.001439
PFDN5	-0.308798381	0.000346	-0.121923328	0.15521	-0.064999078	0.447864
SMIM19	-0.611271517	0.000347	-0.255192189	0.12894	-0.223893095	0.182593
DPYSL2	0.345257649	0.000349	-0.132959351	0.169	0.153378829	0.111996
PPA2	-0.495407843	0.000349	-0.284685624	0.03847	-0.40898157	0.00298
FAM46A	-0.653527786	0.00035	-0.353519266	0.05291	-0.551575994	0.002533
SERP1	-0.412872209	0.000352	-0.15851662	0.16906	-0.217690406	0.058966
SGIP1	0.866736096	0.000352	0.42303435	0.0824	0.53213782	0.028511
NHP2	-0.467643556	0.000355	-0.248328771	0.05332	-0.395933452	0.002129
KIF24	-0.559361315	0.000356	-0.346184103	0.02453	-0.159498813	0.296158
POLR3B	-0.492354406	0.00036	0.150831023	0.26758	0.026190914	0.847161
NARG2	-0.386459156	0.000361	-0.089355526	0.40585	-0.03593936	0.737689
MCTS1	-0.37733155	0.000365	-0.164172636	0.11425	-0.236210105	0.023227
FN3KRP	-0.393762477	0.000367	-0.262455417	0.01513	-0.128839241	0.229239
SLC2A10	-0.542949565	0.000367	-0.395599684	0.00907	-0.79054248	2.24E-07
C14orf105	-0.988287498	0.000368	-0.669134225	0.01553	-0.480896837	0.081661
MGME1	-0.535257713	0.000369	-0.316160714	0.03226	-0.386861709	0.008798
MIDN	0.596773426	0.00037	0.161201814	0.33801	0.144972743	0.388564
CYP2C18	0.995533749	0.000371	1.024370994	0.00025	0.9065892	0.001177
BGLAP	2.136971622	0.000372	0.529010295	0.41244	1.104685023	0.074415
PIAS3	0.433327898	0.000373	-0.204283922	0.09656	0.142518197	0.243569
LSM6	-0.459878949	0.000376	0.065361017	0.60066	-0.033465915	0.788097
PLEKHA4	0.756729724	0.000375	0.319644009	0.13394	0.428332395	0.044332
PLIN3	0.584900403	0.000376	0.302596906	0.06558	0.398107766	0.015357
SIN3A	0.341315739	0.000375	0.087594998	0.36123	0.102093837	0.286123
ATG9A	0.496390863	0.000378	0.291298033	0.03707	0.347672874	0.012753
DDR1	0.425089217	0.000378	0.372007295	0.00185	0.275229094	0.021364
DNAJC19	-0.444910767	0.000378	-0.204829857	0.09579	-0.242108517	0.048711
SLC44A3	-0.672633766	0.000378	-0.354247559	0.06029	-0.765403391	5.17E-05
P11-404P21	2.417771539	0.000379	1.50288328	0.02951	2.206471692	0.001174
PLCE1	-0.460978942	0.00038	-0.308942471	0.01678	-0.377292041	0.003519
CAND1	-0.327536738	0.000383	-0.104743769	0.25514	-0.026246363	0.775417
SPATA6	-0.653384779	0.000387	-0.419827153	0.02142	-0.491507139	0.007071
LAMTOR4	-0.446118786	0.000389	-0.260388182	0.03389	-0.360166284	0.003447
PEX11A	-0.705624193	0.000391	-0.065006618	0.73743	-0.151629889	0.4361
TRIM16L	-0.677100524	0.000391	-0.536755917	0.00413	-0.386548166	0.039162

VPS53	0.572838825	0.000392	0.368045182	0.02269	0.127842045	0.428368
HEMK1	-0.55745917	0.000395	-0.410260863	0.00879	-0.544911594	0.000512
SLC25A40	-0.417569524	0.000396	6.35E-05	0.99956	-0.135260734	0.242852
LDHB	-0.40875861	0.000399	-0.345443058	0.00275	-0.166825726	0.147978
PIP5K1C	0.546378185	0.0004	0.145977606	0.34753	0.008846527	0.954683
METTL15	-0.405606523	0.000404	-0.166131977	0.14219	-0.222061344	0.048875
SLC8B1	0.83407038	0.000406	0.447569581	0.05744	0.642200048	0.006253
RAB35	0.449298471	0.000408	0.119627479	0.34672	0.245710584	0.052574
E2F1	-0.626938804	0.00041	-0.640979665	0.00028	-0.588155612	0.000826
TUBB6	0.74079572	0.00041	0.370979709	0.07691	0.561074108	0.007415
SAMM50	-0.367456621	0.000411	-0.080418854	0.43215	-0.182192022	0.074484
CNN2	0.47326361	0.000413	0.069910793	0.602	0.137357414	0.305312
SP4	-0.507365083	0.000413	-0.404755511	0.00454	-0.416670257	0.00339
ACTR1A	0.323611204	0.000413	0.233780262	0.0106	0.32295954	0.0004
COMMD8	-0.590214975	0.000414	-0.203188257	0.20972	-0.331632992	0.041354
P11-139J15	-2.971671753	0.000415	-0.209736485	0.75375	0.153145066	0.816813
RPS8	-0.404505023	0.000415	-0.34044251	0.00296	-0.236762892	0.038713
HS3ST3B1	0.865566387	0.000417	0.241103042	0.33263	0.704885499	0.003944
TNFAIP6	1.603998711	0.000418	1.866636922	3.64E-05	1.512960434	0.000852
WDR33	-0.336819871	0.00042	-0.10702676	0.26043	-0.027487862	0.772372
GGH	-0.604032756	0.000421	-0.066282116	0.69554	-0.047637439	0.778585
BMP10	3.08945586	0.000423	2.394618498	0.00661	2.095803599	0.017892
ZNF532	0.521662903	0.000424	0.174514986	0.23901	0.387036704	0.008887
UQCRH	-0.331673889	0.000425	-0.156812556	0.09358	-0.084734649	0.363946
LTB4R	0.748003298	0.000426	0.528023335	0.01228	0.268005969	0.207329
ALDH5A1	-0.653291407	0.000428	-0.392648906	0.03208	-0.509210544	0.005558
RIF1	-0.252739818	0.000429	-0.236971069	0.00092	-0.084373737	0.236981
ZRANB3	-0.651451591	0.000432	-0.489818091	0.00738	-0.382690294	0.034953
RPS6KA2	0.988373599	0.000433	0.676548272	0.01634	0.685080245	0.014862
TTC13	-0.599301212	0.000435	-0.182168198	0.2806	-0.012844049	0.939126
METTL9	-0.570598198	0.000436	-0.05062822	0.75359	-0.116841536	0.469314
UHRF2	-0.434182884	0.000437	-0.223995474	0.06756	-0.211249688	0.083839
NIPSNAP1	-0.405400662	0.000439	-0.495833355	1.65E-05	-0.344936043	0.002605
PPP1R12C	0.513321197	0.000438	0.245787444	0.09297	0.341445697	0.019401
SCNN1G	-2.256484839	0.000438	-1.816566379	0.0037	-2.577232909	6.37E-05
PIGK	-0.406436417	0.00044	-0.221521335	0.05109	-0.232609165	0.040387
ATM	-0.431794367	0.000442	-0.192154835	0.11688	-0.019428438	0.873986
ARL6	-0.761906259	0.000444	-0.436157436	0.03619	-0.742000984	0.000433
ZNF124	-0.361575916	0.000445	-0.273460297	0.00749	-0.125693798	0.217478
RPL7A	-0.405927537	0.000446	-0.242081679	0.03611	-0.267275709	0.020674
GPR107	0.385657798	0.000448	0.160914598	0.14314	0.238772549	0.02949
P11-570P14	-2.366793641	0.000451	0.15525967	0.77667	-0.949987233	0.098218
FANCM	-0.607547131	0.000452	-0.500534678	0.00345	-0.420996096	0.013843
NACA	-0.367146049	0.000454	-0.160246763	0.12541	-0.146427549	0.161326
TTC3	-0.2688192	0.000455	-0.103618094	0.17588	-0.245363753	0.001353
PMAIP1	0.98670473	0.000456	0.756575205	0.00715	0.913940027	0.001141
HNRNPF	-0.306114511	0.000456	-0.137769746	0.11346	-0.028854968	0.739943

THOC7	-0.35284874	0.000457	-0.11103255	0.2615	-0.0622282	0.528061
ZNF24	-0.263359283	0.000462	-0.071765931	0.33649	0.104396021	0.160506
NDUFB5	-0.510982191	0.000462	-0.21137703	0.14376	-0.306478873	0.034305
MYD88	0.700062696	0.000464	0.308464217	0.12443	0.308083725	0.124248
NMNAT2	1.377697223	0.000464	1.13316406	0.00397	1.052021467	0.0075
RALGPS1	-0.660664881	0.000464	-0.440765376	0.01879	-0.515110925	0.00598
RAB3D	-0.574670297	0.000466	-0.524141169	0.00137	-0.566769608	0.000538
TRIAP1	-0.528579463	0.000467	-0.329450958	0.02715	-0.269840431	0.069433
FAM173B	-0.547408687	0.00047	-0.268904853	0.07756	-0.195387993	0.197457
RNF145	0.374416824	0.000474	0.383647726	0.00033	0.5240899	9.13E-07
PLCD1	-0.66561032	0.000476	-0.388534766	0.0394	-0.470282716	0.012981
BMF	0.655199561	0.000477	0.526643582	0.00481	0.299762597	0.109057
HOXB4	0.94060957	0.000478	0.084191637	0.75802	0.530166089	0.049707
PDP1	0.539543287	0.000478	0.584406975	0.00014	0.722682248	2.49E-06
GEMIN5	-0.395362776	0.000487	-0.290364437	0.00973	-0.129149952	0.247337
GNPDA1	-0.379685809	0.000491	-0.212249736	0.04706	-0.227606078	0.032895
GPR108	0.478660258	0.000493	0.276327616	0.04396	0.370089825	0.006876
ATP6V0D1	0.346148653	0.000493	0.151921493	0.12509	0.132168248	0.182753
SPIRE1	0.893027358	0.000494	0.095224685	0.71488	0.863064924	0.000779
CCL28	1.160352431	0.000495	1.205047455	0.00028	0.745458978	0.025767
FUT9	-1.845292927	0.000495	-1.42933551	0.0069	-2.336308731	1.06E-05
EEF1A1P22	-1.08411427	0.000496	-0.620244391	0.0305	-0.084308272	0.754945
MRRF	-0.448300007	0.000497	-0.10711365	0.40272	-0.135424625	0.289403
SLC25A24	-0.316824486	0.000497	-0.21071958	0.02005	-0.033250032	0.712953
FARSB	-0.392346876	0.000498	-0.300792137	0.0072	-0.154203285	0.166581
RAB5B	0.220471441	0.000498	0.049655105	0.43346	0.164548214	0.009048
NDFIP1	-0.28919216	0.0005	-0.092698563	0.25948	-0.100828906	0.21951
ERP29	-0.461521477	0.000501	-0.273927061	0.03833	-0.36886244	0.005305
LOXL2	0.778392259	0.000501	0.756126825	0.00072	0.3952155	0.077372
RNF217	0.784811501	0.000502	0.150463259	0.50538	0.133444834	0.55437
RP11-583M2.	-2.219264838	0.000503	-0.151464073	0.78728	-1.154463932	0.047046
BTBD19	0.566708855	0.000503	0.200967045	0.22135	0.125724244	0.443191
GGT5	1.050728527	0.000504	0.509488081	0.09046	0.349888008	0.251193
PET100	-0.468559603	0.000505	-0.062511157	0.63497	-0.21867404	0.096904
COA1	-0.299767923	0.000507	-0.191649567	0.02451	-0.015470523	0.854773
MLLT11	0.559830983	0.000507	0.121694619	0.45126	0.685248588	1.93E-05
WNT9A	1.499092683	0.000507	1.360584881	0.00157	1.045747713	0.016671
LYZ	-1.155264706	0.00051	-0.597741207	0.07214	-0.98822708	0.00295
SLAIN1	-0.705406347	0.000511	-0.435213331	0.03055	-0.171547616	0.392745
PSMD1	1.019698325	0.000513	0.832833616	0.0045	0.314378927	0.284241
SCYL1	0.434089392	0.000514	0.176029735	0.15915	0.280060636	0.02488
TMPRSS5	-1.065525648	0.000514	-0.93734512	0.00213	-1.329348106	1.48E-05
ANKRD18CF	-1.178742638	0.000515	-0.605386094	0.05262	-0.942220228	0.003327
C6orf48	-0.41831838	0.000517	-0.229173889	0.05559	-0.309900544	0.009688
STOX1	-0.719928756	0.000522	-0.585526964	0.00348	-0.925481514	7.72E-06
DCLK3	2.25948189	0.000524	1.42402067	0.02943	1.392562478	0.033349
MGAT5	-0.390371454	0.000524	-0.198878487	0.07642	-0.404353883	0.000323

TRIP10	0.430388836	0.000523	0.195543462	0.11587	0.263265815	0.033694
CASP8AP2	-0.340467393	0.000525	-0.206555717	0.03344	-0.113625428	0.240325
EEF2K	-0.517037575	0.000528	-0.526396077	0.0004	-0.673068935	6.12E-06
THRA	-0.406730131	0.000528	-0.449115429	0.00012	-0.745690924	2.30E-10
NAMPT	1.099984798	0.000529	0.851575861	0.0073	1.133625621	0.000355
FUNDC1	-0.461671822	0.00053	-0.036417618	0.77893	-0.0493885	0.703109
MED25	0.52045506	0.000533	0.004727211	0.97521	0.118404239	0.435066
SAPCD2	-0.815314753	0.000537	-0.353691709	0.12926	-0.799944968	0.000646
GLI2	0.863790474	0.000538	0.040631198	0.87163	0.197716918	0.429561
RABGGTB	-0.304975583	0.000541	-0.167041873	0.05629	0.011982148	0.890667
CHEK1	-0.651797848	0.000544	-0.41543179	0.02681	-0.359154075	0.055367
CD248	0.717228365	0.000545	-0.180785963	0.38672	-0.071981016	0.729895
GCSH	-0.526898376	0.000548	-0.459058317	0.00237	-0.22346681	0.137293
TGFBR1	0.457969825	0.000548	0.413958042	0.00174	0.449498943	0.000672
FKBP3	-0.357671551	0.00055	-0.153876099	0.1328	-0.082236671	0.420553
RBBP7	-0.251379008	0.000551	-0.109489773	0.13019	-0.021339274	0.76798
DBF4	-0.413619173	0.000554	0.027498097	0.81508	0.057231792	0.625506
ELP4	-0.377618552	0.000556	-0.220599947	0.03847	-0.130312961	0.216586
HADHB	-0.252526312	0.000555	-0.153429814	0.03366	-0.160681246	0.026187
HM13	0.322751539	0.000555	0.047260271	0.61378	0.328622621	0.000427
NXN	0.786835169	0.000555	0.057617969	0.80116	0.54089642	0.017601
OGFR	0.49959119	0.000555	0.351565625	0.01507	0.243738575	0.091649
OMA1	-0.666067953	0.000557	-0.148928822	0.4334	-0.262825487	0.169027
TP53	0.82363525	0.000557	0.330268612	0.1665	0.638404853	0.007401
RUSC2	0.567734244	0.000557	-0.024484297	0.88246	0.142165147	0.38961
MCPH1	-0.36569832	0.00056	-0.12732644	0.22518	0.012884701	0.901724
C8orf33	-0.512820086	0.000561	-0.234186553	0.11212	-0.267356782	0.069492
DAB2	0.908383361	0.000566	0.200477919	0.4487	0.545140586	0.038609
EHHADH	-0.831080796	0.000567	-0.446495782	0.06168	-0.680109408	0.004743
CCT6A	-0.339015978	0.000568	-0.151919571	0.12144	-0.103223767	0.292659
PAQR6	0.865569155	0.000568	0.3462159	0.17067	0.51744714	0.039947
SMIM20	-0.479070406	0.000572	-0.350888612	0.0106	-0.569710843	3.65E-05
TANK	0.407180855	0.000575	0.486994889	3.50E-05	0.623758742	1.07E-07
SNCAIP	0.848846734	0.000576	0.343491517	0.16473	0.585091757	0.017655
GPR114	-1.260470349	0.000583	-0.999922411	0.00581	-1.401837945	0.000115
TMEM109	-0.474628381	0.000584	-0.198639279	0.14698	-0.440911061	0.001316
TMEM59	-0.405739524	0.000587	-0.124875413	0.28868	-0.332190596	0.004793
WDFY3	0.449629016	0.000588	0.270529588	0.03851	0.465787028	0.000363
SLC30A5	-0.309033307	0.000591	-0.053561451	0.54393	-0.114169806	0.195742
OPA3	0.397272786	0.000593	0.026186123	0.82115	0.338519752	0.003199
ARHGEF17	0.716736611	0.000595	0.073142786	0.7268	0.55163011	0.008204
MYO5C	-0.570424148	0.000597	-0.309831841	0.06121	-0.557657733	0.000765
PKNOX2	1.1578937	0.000599	0.999250309	0.00309	0.389803871	0.250885
MRE11A	-0.381019446	0.0006	-0.299569859	0.00646	-0.179261726	0.101577
TBC1D4	-0.660172005	0.0006	-0.466730541	0.01468	-0.710076024	0.000217
ACSS1	-0.738097915	0.000605	-0.691748744	0.00124	-1.241276154	9.95E-09
RP11-85G20.	1.332465699	0.000606	0.321188157	0.44285	0.677475079	0.09154

GFRA1	1.268315159	0.000607	1.371695198	0.00021	0.960601073	0.009445
SFMBT2	0.763937064	0.000609	-0.078867864	0.72537	0.540045028	0.015395
MT-ATP6	0.908107386	0.00061	0.429925101	0.10478	0.068717603	0.795491
FAM222A	0.917181727	0.000614	-0.26835311	0.33536	0.347231444	0.200388
GBP1	1.062246766	0.000614	0.279401926	0.36847	1.341474351	1.50E-05
TRIM69	0.638905858	0.000616	0.600649764	0.00127	0.623607823	0.000812
CDK1	-0.540568339	0.000619	-0.15171995	0.33415	-0.055124837	0.725431
IL1A	2.437287445	0.00062	3.016493448	2.24E-05	2.58059395	0.000288
SP2	0.872953213	0.00062	0.193290055	0.46176	0.125056156	0.634112
EXT1	0.400311447	0.000621	0.360112234	0.00206	0.48247216	3.55E-05
ATP5A1	-0.31975035	0.000624	-0.146812366	0.1157	-0.118889943	0.202585
BTN3A2	-0.541581648	0.000624	-0.381913435	0.01469	-0.450465356	0.004258
RHOQ	0.577987824	0.000625	0.42211685	0.01242	0.379469317	0.024504
WDR1	0.340284655	0.000631	0.264457528	0.00785	0.209631397	0.03504
SLIT3	1.155594315	0.000633	1.029955127	0.00232	-0.082697173	0.807606
NCKAP5L	0.499533289	0.000635	0.192255088	0.1904	0.199204148	0.174177
LRRC32	0.902430657	0.000636	0.808982778	0.00218	0.273472263	0.303041
EMD	0.455842541	0.000637	0.19898	0.13513	0.318353942	0.016439
ZNF485	-0.724193598	0.000637	-0.101964236	0.61211	-0.452672856	0.026012
ANKRD36B	0.62695245	0.000639	0.606284234	0.00093	0.366669471	0.045667
CLDN12	0.445941737	0.00064	0.431967495	0.00091	0.453255297	0.000499
PTGIS	0.643958147	0.000643	0.392388769	0.03752	0.026462549	0.888581
TMEM74B	1.261945491	0.000643	1.221751621	0.00092	1.18336966	0.001283
RNF168	-0.34171048	0.000645	-0.239739972	0.01567	-0.144948616	0.143883
CTNNAL1	-0.358911519	0.000648	-0.216618822	0.03773	0.002765207	0.978713
POGK	-0.281813409	0.000648	-0.139876519	0.08789	-0.095300583	0.243775
C2CD5	-0.531617834	0.000651	-0.253717948	0.10172	-0.134255957	0.386116
RFWD2	-0.325280152	0.000651	-0.054115406	0.56732	-0.039516943	0.675516
MLLT4	0.305746734	0.000653	0.037971733	0.67197	0.360643302	5.61E-05
ARHGAP21	0.443716151	0.000663	0.182944063	0.16025	0.384990165	0.003112
NUDT9	-0.467692446	0.000664	-0.072568901	0.59201	-0.297145717	0.028219
MCM8	-0.585135077	0.000668	-0.359465415	0.03384	-0.132149295	0.431758
FAM133B	-0.357674306	0.000669	-0.203319932	0.05054	-0.131415911	0.203997
GSTA1	-1.420174599	0.000671	-1.182026963	0.00458	-1.414621476	0.000696
COX16	-0.417300653	0.000674	-0.257050321	0.03431	-0.145339539	0.228709
PLK2	0.778699722	0.000674	0.88052398	0.00012	0.727895251	0.001514
ZNF92	-0.386111756	0.000674	-0.136509264	0.22275	-0.045082435	0.685849
NOC3L	-0.3545088	0.000678	-0.196434611	0.05536	-0.101396937	0.321512
PTBP3	0.459888631	0.000678	0.317473091	0.01892	0.282474634	0.036729
RND3	0.543544982	0.000679	0.596038056	0.00019	0.838917601	1.46E-07
TMEM163	0.752017746	0.000682	0.934024978	2.35E-05	0.419556151	0.05823
PLEKHB2	0.307287558	0.000684	0.360528624	6.37E-05	0.382328471	2.20E-05
C9orf116	-0.803636704	0.000685	-0.64970072	0.00447	-0.389034331	0.083725
ZMIZ1	0.747985441	0.000689	0.359263236	0.10355	0.14225483	0.519072
FAM35A	-0.3544152	0.000697	-0.084396448	0.41175	-0.02615842	0.798607
FOXRED1	-0.447806673	0.000698	-0.30924888	0.01709	-0.292676877	0.023765
SH3RF3	1.009466424	0.0007	0.51581107	0.08594	0.402905	0.180608

E2F2	-0.975871893	0.000708	-0.630463831	0.0273	-0.622760837	0.0299
ANAPC4	-0.524298982	0.00071	-0.194554298	0.20234	-0.298122481	0.050752
CDK9	0.493584193	0.000711	0.305010474	0.03603	0.328499704	0.023637
RTKN2	-0.581585272	0.00071	-0.339580406	0.0455	-0.222636784	0.186634
WIPF2	0.525531821	0.000714	0.089817878	0.56294	0.247005726	0.111356
ZNF121	-0.341013713	0.000715	-0.16055478	0.11	-0.074019199	0.460583
LPO	2.186889479	0.00072	1.474352939	0.02443	1.262222941	0.05633
VPS29	-0.299973061	0.000725	-0.016990233	0.8459	0.086415897	0.321646
RAD54L2	0.320240513	0.000727	-0.055273329	0.56154	0.057831556	0.541854
CCT8	-1.382698698	0.000728	-0.14147019	0.72843	-0.513863495	0.207531
SMC6	-0.340359797	0.000729	-0.031733993	0.74915	-0.002661733	0.978545
BRIP1	-0.568132682	0.00073	-0.500338469	0.00281	-0.317554245	0.057193
THOC3	-0.470617358	0.000732	-0.259487285	0.05805	-0.405868521	0.003307
RBMX2	-0.549827404	0.000736	-0.004450561	0.97766	0.08907202	0.573973
MRPL24	-0.4452011	0.000738	-0.27340719	0.03514	-0.424516887	0.001119
ZNF813	-0.622437391	0.000738	-0.157127688	0.38941	-0.277909968	0.128133
SEMA5A	1.087923275	0.000739	0.182989828	0.57111	0.351385856	0.276145
RAB43	1.643394423	0.000746	1.134056743	0.02006	0.656139232	0.179148
GCA	-0.62400814	0.000748	-0.280728468	0.12432	-0.436631714	0.017247
CCSER1	-0.615063779	0.00075	-0.434297118	0.01684	-0.421878826	0.020055
MAOA	-0.753600746	0.000751	-0.50840219	0.02277	-0.417482655	0.061374
PSMD3	0.250004453	0.000754	0.170990071	0.02075	0.190646152	0.009845
CREBZF	-0.330265859	0.000755	-0.334013828	0.00063	-0.034778721	0.720586
CLIC4	0.446613926	0.000757	0.331139697	0.01247	0.498804994	0.000166
CDC42SE1	0.347940706	0.000758	0.18124241	0.07939	0.435714934	2.34E-05
RDH10	0.593267604	0.000759	0.33562488	0.05674	0.709126525	5.60E-05
ANKMY2	-0.365162726	0.000765	-0.129498122	0.22647	-0.214994933	0.044726
RAB13	0.424515778	0.000764	0.225994176	0.07322	0.091249638	0.470129
RTN4	0.282333644	0.000767	0.270343423	0.00126	0.33480595	6.42E-05
FAM160B2	0.442314328	0.000767	0.333162049	0.01128	0.285035917	0.03021
BRD4	0.589918712	0.000769	0.154928375	0.37766	0.28704242	0.101827
PSMB9	1.2778389	0.000769	1.559725181	3.14E-05	1.480148542	7.97E-05
GNPDA2	-0.385001031	0.000772	-0.103031286	0.36074	-0.059263912	0.597746
FDX1	-0.444759129	0.000775	-0.194128875	0.13644	-0.39263821	0.002715
SKP2	-0.459473683	0.000776	-0.411527469	0.00237	-0.25201331	0.062586
IMPDH2	-0.386075482	0.000779	-0.15950566	0.16386	-0.259416184	0.023666
ZSWIM4	0.762616406	0.000779	0.534160872	0.01882	0.513036425	0.024221
KLF7	0.919475988	0.000781	0.618597386	0.02395	0.244852278	0.371729
UGCG	0.64121699	0.000781	0.279881255	0.1428	0.837921584	1.11E-05
IMPDH1	-0.655217699	0.000783	-0.636097403	0.00109	-0.859630793	1.04E-05
SYPL1	-0.32878057	0.000784	-0.03864531	0.69	-0.123025677	0.204674
PDIA3P1	-0.751737311	0.000786	-0.253150822	0.23886	-0.434516613	0.044549
OXCT1	-0.569174677	0.000788	-0.288435982	0.08354	-0.135872196	0.41623
RALGDS	0.455271814	0.000788	0.067584547	0.61932	0.187963885	0.165896
POLR3GL	-0.482570829	0.000789	-0.539789459	0.00015	-0.455769255	0.001231
CLIP1	0.317808798	0.00079	0.168094383	0.07558	0.352533698	0.000179
ADAMTSL5	1.064670885	0.000799	0.676837904	0.03462	0.302856293	0.349893

TMA7	-0.257518817	0.000799	-0.050515298	0.50602	-0.048661109	0.52121
PLIN5	1.178432364	0.0008	0.920715081	0.00911	0.706076595	0.045374
CHST15	1.018607822	0.000803	-0.117639654	0.70005	0.673140978	0.026868
SNRNP48	-0.336926426	0.000804	-0.282547009	0.0045	0.084210279	0.389073
CWF19L2	-0.361792013	0.000807	-0.064001092	0.54414	0.020168717	0.847141
DOK3	1.019008382	0.000811	0.913934887	0.00275	0.81597102	0.007117
SETD1B	0.758558805	0.000811	0.311489016	0.17335	0.152300921	0.506365
GINS1	-0.529907393	0.000812	-0.342424024	0.0292	-0.20028363	0.200655
IDH1	-0.416919154	0.000812	-0.308039958	0.01327	-0.252093979	0.042641
HAT1	-0.372519825	0.000813	-0.127514708	0.24728	-0.121230998	0.270961
CD1D	1.080062951	0.000816	1.226551102	0.00013	0.606667164	0.061272
IMPACT	-0.42364684	0.000819	-0.216478636	0.08393	-0.308662923	0.013735
ASB9	-1.194929443	0.000824	-0.637295547	0.06928	-0.009786828	0.977354
HIBCH	-0.63982317	0.000823	-0.608167292	0.00138	-0.456531008	0.016168
CINP	-0.319638183	0.000826	-0.205723881	0.02818	-0.268666028	0.004189
PRRT2	1.063709089	0.000829	0.186281654	0.56472	0.783230858	0.014095
AFF4	0.444033456	0.000831	0.212834325	0.10903	0.287380139	0.030427
CALM2	-0.32528925	0.000832	0.024233868	0.80309	-0.095864301	0.324008
DENND5A	0.556576623	0.000832	0.279705118	0.09307	0.450658415	0.006737
RPL6	-0.382359168	0.000833	-0.280283286	0.01428	-0.142172094	0.213807
SPAG7	-0.244785751	0.000833	-0.099839007	0.16587	-0.020846732	0.770744
3-Mar	0.786311385	0.000842	0.945224543	4.87E-05	0.749540578	0.001329
HSPE1P3	-2.279927015	0.000845	0.175955183	0.78767	0.529674878	0.416486
ULK1	0.714615174	0.000845	0.228584757	0.28788	0.192973843	0.370163
TEAD2	0.567671958	0.000848	-0.082210055	0.62952	0.471193288	0.005533
PPM1D	-0.32461485	0.000849	-0.170204942	0.07581	-0.123006145	0.196522
C9orf152	-1.112048204	0.000852	-0.916019847	0.0057	-1.511866143	6.18E-06
RUVBL1	-0.415137411	0.000858	-0.220567706	0.07339	-0.138934988	0.258906
HEBP2	-0.350208756	0.000862	0.12045389	0.24892	-0.149716813	0.152129
AVPI1	-0.970079055	0.000872	-0.843359317	0.00343	-1.014679366	0.000453
MEX3C	0.691528612	0.000871	0.449131205	0.03057	0.416565285	0.044885
PTDSS1	-0.34890697	0.000875	-0.208840907	0.04515	-0.37309624	0.000357
BMP5	1.100744806	0.000876	1.034086523	0.00175	0.632981925	0.056265
HSF1	0.48922354	0.000877	0.185002421	0.20891	0.242143907	0.100701
MRPL19	-0.380445974	0.000878	-0.004049827	0.97147	-0.186819358	0.098593
CCDC71L	0.690156861	0.00088	0.326187163	0.11683	0.550639901	0.007878
SIPA1L2	0.612088249	0.00088	0.362454842	0.0489	0.607446249	0.000956
ADH5	-0.378106717	0.000881	-0.271168916	0.01658	-0.066800238	0.554289
FAM208A	-0.31627976	0.000883	0.006187167	0.94788	-0.032757904	0.729355
HEXB	-0.495129737	0.000885	-0.259028618	0.08136	-0.264485986	0.075128
ANAPC5	-0.343172633	0.000888	-0.199765064	0.05199	-0.328434316	0.001407
GJC2	-0.982266476	0.000888	-0.95085752	0.0011	-1.040597556	0.000363
EXOSC8	-0.405904771	0.000889	-0.024056382	0.84208	-0.027452343	0.819453
NUP54	-0.356629923	0.00089	-0.025665686	0.8093	0.016140125	0.879144
ARHGAP24	-0.658506774	0.000893	0.026032553	0.8935	-0.45683453	0.020211
BRCC3	-0.351459334	0.0009	-0.034508905	0.74023	-0.128204727	0.218882
ARSD	-0.405284702	0.000902	-0.239722864	0.04828	-0.517947128	2.13E-05

SHC1	0.386944337	0.000902	0.280607895	0.016	0.34224689	0.003281
IQGAP2	-0.67069868	0.000903	-0.515764262	0.01064	-0.687234731	0.000666
PRKACA	0.664622622	0.000904	0.367922712	0.06759	0.232152576	0.249763
AZI2	0.384701197	0.000906	0.423269229	0.00025	0.396198258	0.000597
DBP	-1.204316317	0.000905	-1.542811432	2.06E-05	-0.937557552	0.00935
DTWD1	-0.376099397	0.000908	-0.310416256	0.00579	-0.262479137	0.019051
U2SURP	-0.329233536	0.000908	-0.147732751	0.13528	-0.000863099	0.993029
ARHGEF34F	0.550287183	0.00091	0.147211289	0.37593	0.322355658	0.051976
VGLL4	0.294746792	0.000912	0.030887183	0.72723	-0.089747445	0.311008
CPNE3	-0.266090829	0.000918	-0.047289591	0.55327	-0.196421639	0.013781
EIF4G3	0.273696785	0.000919	0.161249883	0.05047	0.111744723	0.1746
UBL5	-0.380387424	0.000922	-0.177467555	0.1189	-0.208885678	0.06636
EHD4	0.380773799	0.000927	0.486536976	1.94E-05	0.392918662	0.000568
PRIMPOL	-0.706106975	0.00093	-0.271852626	0.19022	-0.499524099	0.016217
TMEM173	1.023012147	0.00093	0.728761092	0.01839	0.282175217	0.362998
CLIP3	1.075291126	0.000936	0.058141411	0.86033	0.39797527	0.224655
COQ3	-0.765617763	0.000942	-0.170475292	0.44211	-0.653950116	0.004127
H2AFV	-0.427522863	0.000943	-0.090397428	0.48317	-0.057828819	0.653452
ZNF22	-0.422884153	0.000949	-0.180566111	0.15399	0.000282541	0.998212
TAF4B	-0.739352576	0.000955	-0.555551067	0.0115	-0.686063904	0.001867
PSMA1	-0.31207614	0.000962	0.09722001	0.29937	0.077181607	0.410008
CDK6	0.510806551	0.000963	0.190217694	0.21887	0.207804929	0.179152
PSD	1.041266014	0.000963	-0.338951512	0.30538	0.760984241	0.016088
PPAP2C	-0.869562961	0.000971	-0.579022136	0.02628	-0.912406342	0.000485
VMA21	-0.283010754	0.000972	-0.176737425	0.03802	-0.166939767	0.049704
NR1D2	-0.830186134	0.000975	-0.84285857	0.00078	-0.735699026	0.003289
EXOC7	0.312093081	0.000977	0.071236483	0.45199	0.403033863	1.99E-05
CENPK	-0.536095529	0.000986	-0.201686612	0.20799	-0.185653182	0.24495
ARL8B	0.934897311	0.000987	0.745522286	0.0085	0.466536251	0.09948
CCDC34	-0.512825432	0.00099	-0.478706203	0.00191	-0.381875026	0.013111
ATP11B	-0.323105293	0.000991	-0.018930064	0.84666	-0.081122767	0.407347
MCM7	-0.509644106	0.000994	-0.367770844	0.01729	-0.288885994	0.061178
RAC2	0.957452439	0.000993	0.494247395	0.09002	0.530163744	0.06882
SORBS2	-0.774229814	0.000994	-0.517467618	0.02635	-1.239587003	1.52E-07
SAE1	-0.34101557	0.000999	-0.345706371	0.00083	-0.052779148	0.608356
ANAPC1	-0.362531027	0.001006	-0.272829337	0.01305	-0.274628628	0.012354
SLC35F6	0.303484501	0.001014	0.095714128	0.29988	0.041084243	0.656479
CTGF	1.116922777	0.001023	1.105987948	0.00114	0.939851455	0.00571
IMMP2L	-0.677226126	0.001028	-0.404579091	0.04428	-0.650698442	0.001473
C19orf66	0.582020317	0.001031	0.450934722	0.01093	0.604515037	0.000608
DVL3	0.337137206	0.001033	0.056974159	0.58169	0.060021793	0.56058
LIN7C	-0.304695554	0.001035	-0.28018844	0.00246	-0.143773795	0.119335
FHOD3	1.721556498	0.001039	1.830058108	0.00047	1.324583313	0.013716
PLXNB2	0.540589263	0.001039	0.137981014	0.40258	0.235567496	0.153072
TOLLIP	0.442514268	0.001042	0.135955183	0.31372	-0.033315811	0.80618
CDC6	-0.656287352	0.001043	-0.561260361	0.00486	-0.41645155	0.036452
JAG1	0.616841254	0.001047	0.983313928	1.68E-07	0.652955576	0.000517

RAB7A	0.604801305	0.001055	0.469856135	0.01095	0.534398989	0.003795
ZNF443	-0.643726887	0.001055	-0.326401957	0.08871	-0.082327078	0.663534
SYNPO	1.300573348	0.001056	1.060577921	0.00757	0.938970555	0.018205
PGGT1B	-0.379036043	0.00106	0.024956777	0.82805	-0.057193252	0.618365
SLC19A2	-0.378132765	0.001062	-0.183825609	0.1076	0.011306583	0.920714
POT1	-0.528185328	0.001065	0.051760608	0.74518	-0.363182983	0.022823
KDM4B	0.609027449	0.001068	0.066276896	0.72319	0.235681101	0.206782
CYLD	0.450686865	0.001069	0.45643233	0.00089	0.473284549	0.000553
TMEM19	-0.675683368	0.00107	0.014587202	0.94328	-0.388092314	0.059084
ALDH18A1	-0.463513263	0.001071	-0.312218104	0.0274	-0.592090499	2.91E-05
SIRT6	0.605962302	0.001075	0.235286234	0.20715	0.021564309	0.909387
ANKRD11	0.363096082	0.00108	0.054923458	0.62125	0.119566961	0.281575
SLC27A5	-0.545628406	0.00108	-0.46160013	0.0052	-0.42812622	0.009576
FAU	-0.332634861	0.001084	-0.025701605	0.79984	-0.127326085	0.209382
ALOX5	1.607460186	0.001089	1.772720516	0.00031	1.423368693	0.003795
DSP	-0.423768735	0.001088	-0.398012575	0.00215	-0.594363748	4.60E-06
SASS6	-0.56884907	0.001093	-0.261836588	0.12864	-0.128873173	0.453088
AIMP1	-0.373729917	0.001097	-0.159931344	0.15749	-0.219294103	0.052176
FAM3B	-0.948820464	0.001102	-0.134836138	0.63741	-1.273089718	1.36E-05
GPD2	-0.453798436	0.001104	-0.312217615	0.0244	-0.220110592	0.112631
ALG13	-0.482622528	0.001107	-0.199531049	0.17082	-0.377896989	0.009838
AUH	-0.593457187	0.001111	-0.256836629	0.14967	-0.367924853	0.039689
RTFDC1	-0.356603482	0.001114	-0.146145198	0.17796	-0.149447717	0.168045
ADAMTSL2	1.379997114	0.001117	0.966237582	0.02417	0.130085907	0.772293
CSPP1	-0.446080704	0.001117	-0.169568644	0.20949	-0.230584429	0.08781
GPR124	0.810922243	0.001117	-0.052233108	0.83397	0.208529972	0.402328
MGAT1	0.309214744	0.001121	0.181600752	0.05523	0.084424763	0.373652
HNRNPDL	-0.253104246	0.001124	-0.1196225	0.12291	-0.032826676	0.671795
TARSL2	-0.498092599	0.001124	-0.542003816	0.00037	-0.352259969	0.020066
RASA4B	1.151732549	0.001131	0.337723795	0.34743	0.01712779	0.962387
CHML	-0.34501751	0.00114	-0.047792503	0.64818	-0.1957079	0.062259
TTC39B	0.408463175	0.001141	0.601164981	1.53E-06	0.432146002	0.000562
DNAJB5	0.68298442	0.001144	0.287092979	0.17396	0.594390888	0.004472
CAMLG	-0.447623323	0.001148	-0.138745184	0.30478	-0.175650206	0.194713
PLCH1	-0.737885446	0.001147	-0.281269388	0.21141	-0.772453981	0.000645
PRSS12	0.965928017	0.001147	1.162541065	8.88E-05	1.029210726	0.000526
CARM1	0.373220534	0.001153	-0.0398255	0.72864	0.009655614	0.93297
CTSV	-0.58986167	0.001152	-0.386045363	0.0303	-0.540659449	0.002622
MAP3K9	0.506554556	0.001154	-0.127873305	0.41447	0.152200035	0.329217
SEC24B	0.235330853	0.001154	0.010614123	0.88361	0.224025856	0.001834
COX7C	-0.352858292	0.001162	-0.114208593	0.29164	-0.194409157	0.072675
UBXN10	-1.176946768	0.001165	-0.730358546	0.03332	-0.884598587	0.010483
CASP4	0.571802913	0.00117	0.763822719	1.38E-05	0.643685292	0.000249
CYB5B	0.278985431	0.001172	0.287607404	0.00079	0.4615741	6.69E-08
DARS	-0.313187908	0.001172	-0.214307642	0.02564	-0.164567352	0.086394
FAM168A	0.285798927	0.001172	0.203596616	0.02023	0.163798361	0.06158
HINT3	-0.3014493	0.00117	0.041967669	0.64438	-0.071053419	0.435457

PTX3	2.06697005	0.001171	1.642330706	0.00993	2.063404498	0.001191
TASP1	-0.419882166	0.001171	-0.253452552	0.04567	-0.319772564	0.011519
P1-130H16.1	4.469109534	0.001176	3.292676144	0.01741	3.313532808	0.01671
ATN1	0.876623339	0.001178	0.147839897	0.58709	0.310442765	0.252514
CRTC2	0.711911348	0.001184	0.189183847	0.39303	0.484451545	0.027897
DHODH	-0.51369588	0.001187	-0.315294653	0.04515	-0.353332817	0.024159
CHST11	1.080212335	0.001192	0.423537503	0.20398	0.137619431	0.680105
MLLT1	0.369632007	0.001211	0.244153389	0.03286	-0.044209024	0.70015
CMTM6	-0.301069767	0.001215	0.027194828	0.76905	-0.088063296	0.341933
IL1R1	1.035486314	0.001216	0.710953412	0.02636	1.041306672	0.001138
PDLIM4	0.720628208	0.001213	0.611565336	0.00594	0.389832208	0.080554
PIK3CG	1.472870608	0.001214	0.893003207	0.05612	0.864687609	0.05988
VAMP2	0.653027645	0.001217	-0.112875707	0.58842	0.175812824	0.389489
COL4A3BP	-0.358745574	0.001219	-0.153993746	0.16143	-0.122569632	0.263816
PXMP2	-0.60289063	0.001219	-0.364004415	0.04625	-0.504995016	0.005604
TMEM245	-0.28982047	0.001222	-0.25118031	0.00492	-0.148915077	0.094611
RPS17L	-0.525350737	0.001223	-0.24299205	0.13451	-0.108465611	0.504048
MIS18BP1	-0.498982872	0.00123	-0.202361948	0.18791	0.147338125	0.336663
ANKLE2	0.403323858	0.001232	0.263872394	0.03423	0.474579927	0.000138
PRDX6	-0.321378094	0.001232	-0.219983576	0.02663	-0.204887421	0.038835
C4orf27	-0.419148802	0.001236	-0.489358809	0.00014	-0.242725973	0.055349
FPGT	-0.498454058	0.001241	-0.481599589	0.00137	-0.40772605	0.006707
PDPN	1.594290987	0.001242	1.319505728	0.00766	1.321247103	0.007596
RCN2	-0.302254067	0.001245	-0.07145586	0.44203	0.026323672	0.776601
PPP1R3B	-0.518312429	0.001248	-0.384508168	0.01646	-0.587426666	0.000251
SPRR3	2.570134907	0.001249	3.16578251	6.55E-05	1.902151342	0.017328
KFZP761J14	0.715822911	0.001254	0.244345229	0.27244	0.131368603	0.557222
FANCB	-0.767359488	0.001254	-0.520271391	0.02575	-0.385837983	0.095699
PGRMC2	-0.565177253	0.001254	-0.046666976	0.78849	-0.416583521	0.016972
FBXL7	0.799823969	0.001256	-0.029466108	0.90661	0.57666338	0.020065
NAA15	-0.33853918	0.001259	-0.118843647	0.25593	-0.153552927	0.14115
CENPC	-0.321526794	0.00127	-0.060369069	0.5387	-0.060553106	0.535946
AKT2	0.379264699	0.001275	0.01427121	0.90347	0.279806881	0.017186
F2RL2	0.715699412	0.001275	0.616593704	0.00551	0.615637884	0.005568
FAM222B	0.673764617	0.001279	0.147644231	0.48297	0.338189379	0.106839
HAPLN3	1.182867927	0.001279	1.272384066	0.0005	0.521579643	0.158581
TLDC1	0.335599897	0.001278	0.14078481	0.17402	0.174554458	0.09183
NUMB	0.367673372	0.001285	0.191954957	0.09231	0.26006478	0.022494
ERCC6L	-0.684014506	0.001289	-0.14995647	0.47369	-0.212500514	0.310084
PCCB	-0.526242529	0.001288	-0.325297049	0.04518	-0.271853158	0.094306
ZSWIM6	0.343404662	0.001289	0.110482934	0.30125	0.271742241	0.010466
GEN1	-0.583572823	0.001291	-0.338243948	0.06125	-0.130588424	0.469314
FBRSL1	0.819096465	0.001293	-0.220016714	0.38969	-0.259065386	0.312805
SH3KBP1	0.616917339	0.001306	0.493744195	0.0099	0.165154527	0.390429
SNRPE	-0.388139825	0.001307	-0.149534663	0.21099	-0.000629307	0.995788
TNFSF18	1.352924108	0.00131	1.123310964	0.00763	0.932091275	0.026978
CEBPZ-AS1	-0.893292015	0.001312	0.130676678	0.63006	-1.114859597	6.08E-05

NDUFS3	-0.327840872	0.001315	-0.13412583	0.18184	-0.182683116	0.068579
PLBD1	-0.726952706	0.001321	0.141708726	0.52724	-0.576829867	0.010465
CASP10	0.758925808	0.001324	1.076036734	4.84E-06	1.084028456	4.11E-06
PIK3CD	1.860380234	0.001324	1.612066205	0.00563	0.982516131	0.091327
PTGR1	-0.484294756	0.001324	-0.191502022	0.20204	-0.216127235	0.149868
NPLOC4	0.295138162	0.001334	0.285921329	0.00185	0.411870202	6.96E-06
PFDN4	-0.471490522	0.001333	-0.137696273	0.33078	-0.001124804	0.993566
SLC16A1	-0.368866211	0.001334	-0.212429225	0.06337	-0.297237692	0.009421
SOD1	-0.273699777	0.001341	-0.050746591	0.54813	-0.054075718	0.521764
NSMCE4A	-0.386801092	0.001342	-0.121418676	0.30812	-0.052512643	0.658078
AGO3	0.441470779	0.001345	0.239269945	0.08219	0.196215632	0.153419
TMEM141	-0.515869034	0.001353	-0.352989626	0.02774	-0.518811934	0.001233
OTUD5	0.409415293	0.001357	0.38467502	0.00253	0.40052427	0.00167
SLBP	-0.388684974	0.001357	-0.184244441	0.1253	-0.138990396	0.247174
EEF1G	-0.384389747	0.001359	-0.212476532	0.07655	-0.307946446	0.010265
EEF1B2	-0.388702034	0.001365	-0.181853429	0.13368	-0.234610105	0.053017
HIP1	0.899433518	0.001365	0.617964129	0.0279	0.675023484	0.016275
FBLN1	0.464913568	0.00137	0.013894848	0.92395	0.342754137	0.018192
HAUS3	-0.338146996	0.001372	-0.009630039	0.92576	-0.05991603	0.560579
PPP1R16A	0.4771855	0.001375	0.275157115	0.06484	-0.054885568	0.71333
HELZ2	0.823867473	0.001378	0.653027713	0.01112	0.92247529	0.000353
PSMD4	-0.305429167	0.001378	-0.137542296	0.14698	-0.060169949	0.525163
QRSL1	-0.364634314	0.001382	-0.007005075	0.95037	0.014946	0.894194
DIO3	2.092139499	0.001384	2.1565082	0.00097	0.931977653	0.156019
PAN-P2RY1	3.204805515	0.001389	1.766948827	0.08118	1.731547752	0.087407
TXN	-0.404436254	0.001391	0.093405527	0.45824	-0.015787717	0.900254
PKD1P6	0.58110745	0.001395	0.014219055	0.93842	0.094453228	0.605507
RPS26	-0.422661141	0.001395	-0.138942882	0.29178	-0.12259076	0.352063
RPL34	-0.365761045	0.001398	-0.168631541	0.14026	-0.106966683	0.349393
ZNF204P	-0.59906128	0.001408	-0.410794619	0.02675	-0.117324175	0.523403
MMS22L	-0.710108547	0.001416	-0.349509646	0.11549	-0.347995004	0.116946
TAGLN2	-0.470648271	0.001431	-0.072064314	0.6249	-0.579058342	8.70E-05
TUSC3	-0.380794265	0.00143	0.03186136	0.78851	-0.203364451	0.087274
ZNF573	-0.562568323	0.001431	-0.180919208	0.29194	-0.293079143	0.088439
OTUB1	0.252408461	0.001434	0.049200006	0.53596	0.186738077	0.018017
TTYH3	0.600201585	0.001434	-0.01425265	0.93976	-0.172660992	0.360689
SZRD1	0.330503149	0.00144	0.089789681	0.38597	0.096858817	0.349263
C15orf48	1.177909596	0.001442	1.665148122	6.16E-06	1.104111987	0.002793
PDIA3	-0.374678848	0.00145	-0.119295229	0.31035	-0.074374387	0.527054
WDR45	0.540831774	0.001449	0.253704749	0.135	0.408297051	0.016043
MNT	0.470278205	0.001454	0.202853294	0.17191	0.232290909	0.117382
RECQL	-0.358563932	0.001457	-0.008917209	0.93614	-0.033657113	0.762416
UBE2R2	0.191650038	0.001456	-0.045423683	0.45187	0.173630245	0.00358
CCRL2	1.565105988	0.001464	1.364932602	0.00552	0.564159212	0.263797
MIS18A	-0.516652219	0.001476	-0.287844139	0.07085	-0.222516968	0.16094
BAIAP2L2	1.200776946	0.00148	0.465462175	0.22189	1.134914049	0.002651
EGR2	1.137172169	0.001482	0.977259708	0.00629	0.697479704	0.051705

LDLRAD4	0.962863222	0.001481	0.662245251	0.02869	0.716873576	0.017779
ZNF75A	-0.450702475	0.001487	-0.012218349	0.93001	-0.028407804	0.838117
ABCF2	0.367204558	0.001493	0.357277591	0.00194	0.42389429	0.000231
SUMF2	-0.368837863	0.001494	-0.41399579	0.00035	-0.46385737	6.33E-05
RELA	0.760450715	0.0015	0.098521181	0.68154	0.328679083	0.170443
PPM1L	-0.430482486	0.001502	-0.496948989	0.00024	-0.491946545	0.000274
C19orf47	0.575481383	0.001507	0.289714003	0.11261	0.469215863	0.009538
ARGLU1	-0.360772126	0.001509	-0.263692743	0.02003	0.017403371	0.877673
AGFG2	0.538347366	0.001515	0.241626343	0.15598	0.301444541	0.075297
GNB1	0.36242728	0.001516	0.196403299	0.08545	0.390809601	0.000616
CNTNAP3	1.01816229	0.001518	0.252734951	0.43483	0.724711434	0.023464
TMED1	-0.433296816	0.001523	-0.186618572	0.16152	-0.324011431	0.015121
MT-ND4	0.877301606	0.001525	0.226885311	0.41245	0.219256471	0.428334
PLAUR	0.719670661	0.001526	0.551970785	0.01507	0.649701084	0.004183
CHAC2	-0.914707434	0.001527	-0.048843434	0.85593	-0.49558604	0.071417
POU2F1	0.623877888	0.001528	0.348333915	0.07674	0.536712839	0.006353
MUC1	-1.880673082	0.001531	-0.628730871	0.28762	-1.535132428	0.009548
SBF1	0.526835348	0.001531	0.327750364	0.04861	0.196326908	0.237905
ALDH7A1	-0.483131419	0.001534	-0.181392187	0.23052	-0.329621711	0.029492
KIAA0368	0.485297809	0.001535	0.502403358	0.00103	0.782316409	3.19E-07
DDX11	-0.445217112	0.001542	-0.26745649	0.05632	-0.108288555	0.437842
FBXO45	-0.304449619	0.001541	-0.010933541	0.90724	-0.06098858	0.51509
MMP25	1.646197505	0.001541	1.417196994	0.00665	0.55341597	0.308857
NABP1	0.544477879	0.001546	0.271949029	0.11399	0.244686905	0.155595
C6orf57	-0.506616654	0.001548	-0.363195069	0.02124	-0.248781268	0.111558
TARBP1	-0.597855384	0.001549	-0.264011659	0.15959	-0.29504935	0.116184
AC010970.2	-1.614948473	0.001552	0.329366627	0.51635	-0.298433642	0.55683
LMO2	1.526541554	0.001554	1.364951571	0.00457	0.546609696	0.264616
HIPK2	0.552172318	0.001558	0.284692	0.10281	0.394660072	0.023705
RP11-641D5.	-0.408638278	0.001558	-0.275465104	0.03272	-0.251227581	0.051407
HNRNPA1P4	0.803974662	0.001564	0.409534841	0.11301	0.678646134	0.007162
CSE1L	-0.389242758	0.001571	-0.174542751	0.15548	0.022884744	0.852108
ESCO2	-0.784876188	0.001572	-0.421182277	0.08824	-0.568036039	0.021505
HDDC3	-0.734758419	0.001574	-0.08544208	0.70456	-0.299875306	0.186894
VDAC3	-0.251939816	0.001575	0.090450709	0.24934	0.013375837	0.864836
EPM2A	-2.013010162	0.00158	-0.195399936	0.7037	-0.112448972	0.828088
RNF170	-0.359542167	0.00158	0.107036093	0.33681	-0.253851751	0.024052
SNRNP40	-0.345197407	0.001578	-0.065225075	0.54669	-0.142299829	0.18826
SPPL2A	0.357114207	0.00158	0.434297793	0.00012	0.282033262	0.012299
NIPAL3	-0.366941678	0.001582	-0.245276113	0.0322	-0.420956062	0.000269
AP3S1	-0.302497127	0.001583	0.065216122	0.4902	-0.099676757	0.292361
GOLPH3	0.4020979	0.001587	0.453385963	0.00036	0.555080006	1.25E-05
ZFYVE1	0.543222558	0.001587	0.525575646	0.00206	0.336175074	0.049403
CHORDC1	-0.364475044	0.001589	-0.168362252	0.14182	0.090512534	0.427686
LIF	0.962804322	0.001589	0.433481816	0.15541	1.128290597	0.000209
RBMS1	0.281784709	0.0016	0.295899943	0.0009	0.424394111	1.83E-06
TXNDC17	-0.541996102	0.001599	-0.148900648	0.38175	-0.23493458	0.167018

ZSWIM1	0.356082352	0.001603	0.131687444	0.24269	0.197660915	0.077986
BRIX1	-0.440912611	0.001609	-0.151148924	0.274	-0.087195545	0.527393
ADCK3	-0.603301421	0.001615	-0.331470963	0.08157	-0.619655605	0.001173
CSMD1	2.619392155	0.001617	1.607136536	0.05498	1.186827466	0.178043
ITFG3	-0.382254762	0.001614	-0.406866682	0.00078	-0.396813443	0.001051
SCAF4	0.36794973	0.001617	-0.060189023	0.60885	0.083398745	0.476325
ZNF582	0.833630895	0.001615	0.467459539	0.08197	0.119102506	0.656954
P11-745A24	1.208391722	0.001621	0.592320493	0.13474	1.20009258	0.001507
GKAP1	-0.684945101	0.001627	-0.344261635	0.09306	-0.392959815	0.056727
GLRX5	-0.264027643	0.001635	0.05251207	0.52336	-0.191420986	0.020905
RFTN1	0.688187082	0.001636	0.341551804	0.11827	0.521790331	0.016898
SLITRK3	1.534877618	0.001636	0.654457644	0.19096	0.567154696	0.249753
NUP35	-0.450945318	0.001638	-0.154060474	0.27386	-0.167943516	0.232523
ELF1	-0.398932991	0.001646	-0.103518353	0.41006	-0.2749686	0.029017
MESDC2	-0.273078805	0.001646	-0.123808536	0.14998	-0.114837073	0.180807
PI4K2B	-0.419361336	0.001649	-0.025961397	0.84349	-0.331018259	0.012261
PHLDA1	0.66168047	0.001653	0.502942779	0.01677	0.327423967	0.119538
?P11-355I22.	-1.009723953	0.001653	-0.757717427	0.01733	-1.525920371	2.30E-06
ZNF846	-0.513155194	0.001651	-0.176539035	0.27142	-0.215459805	0.178777
RGS7	-1.104317896	0.001654	-0.2325983	0.49092	-1.038600072	0.002849
FOXA2	-0.910046886	0.001658	-0.655763968	0.02292	-0.648740883	0.024369
FAM204A	-0.298235337	0.001665	-0.13393521	0.15312	-0.153293131	0.101284
MRFAP1L1	-0.287482747	0.001664	-0.148077152	0.10174	0.014450405	0.872427
INPP5E	0.434291412	0.001673	0.188730552	0.1743	0.304891437	0.026236
TPMT	-0.373356352	0.001675	-0.11496435	0.32465	-0.238784287	0.04128
AC243547.1	0.515801344	0.001678	0.075221307	0.64805	0.211558173	0.19792
ESM1	3.273093689	0.001678	2.054180985	0.05114	0.284140192	0.797353
IL18BP	0.652708757	0.001679	0.209253046	0.32437	0.537441923	0.0096
MID1	0.630075917	0.001679	0.088427485	0.65979	-0.084299563	0.674876
NLGN2	0.735343731	0.001682	-0.113706669	0.63123	0.069851152	0.766841
ZNF702P	-0.570157674	0.001682	-0.489567205	0.00626	-0.305817379	0.08551
DENND2D	-0.779146159	0.001696	-0.823529	0.0009	-0.656466505	0.008106
ZNF718	-0.791508765	0.001697	-0.324496842	0.18403	-0.375942374	0.12373
C1QTNF6	0.440252431	0.001704	0.26707707	0.05678	0.113701	0.418499
MID1IP1	0.714529404	0.001704	0.294591796	0.19804	0.223006267	0.329951
NPIPA7	0.433472033	0.001714	0.139754637	0.31322	0.329232544	0.016997
RPP30	-0.307089616	0.001714	-0.115017516	0.22996	-0.14255797	0.135849
NOTCH1	0.748981146	0.001719	0.355102617	0.13776	0.224854792	0.347584
STEAP4	1.024905578	0.001727	1.593823831	9.70E-07	1.524960472	2.83E-06
EXOC4	-0.435942977	0.001734	-0.553679535	6.75E-05	-0.415579774	0.002751
WDR11	0.314787195	0.001739	0.421750578	2.57E-05	0.349583641	0.000488
PHF14	-0.300230799	0.001747	-0.359766568	0.00016	-0.324931481	0.000638
ZWINT	-0.542171243	0.001759	-0.256780805	0.1359	-0.115108377	0.503452
ADAMTS7	0.807881333	0.001766	-0.019224806	0.94101	0.20238175	0.43505
SYTL5	-0.548945098	0.001766	-0.137194256	0.43196	-0.481004485	0.005987
KLHL12	-0.316190298	0.001772	-0.013778544	0.88948	-0.261263092	0.008936
NFATC1	1.319316756	0.001778	1.069493951	0.01199	0.832218167	0.052329

SMCO4	-0.665262627	0.001783	-0.096301646	0.63158	-0.643686543	0.002047
EHF	0.711667689	0.001792	0.928891462	4.52E-05	0.967526729	2.15E-05
PPT1	-0.433970954	0.001791	-0.222299745	0.10798	-0.085641337	0.534991
PCDHA14	-0.561907668	0.001795	-0.278735971	0.11611	-0.553055101	0.001947
SNRNP25	-0.48177827	0.001814	-0.222563947	0.14354	-0.242981215	0.109539
CTTN	0.196618847	0.001817	0.106937165	0.08902	0.142547201	0.023395
ACTB	0.26306437	0.001822	-0.043685076	0.60464	0.127241195	0.131522
GABBR1	0.743522972	0.001829	0.249699471	0.29928	0.363777688	0.126682
MSN	0.567607049	0.001837	0.398381577	0.02875	0.395044666	0.030095
PABPC4	-0.429559099	0.001838	-0.254795789	0.06414	-0.181228278	0.188125
AC002398.9	1.42056493	0.001841	1.491017676	0.00103	1.419351145	0.001782
PLA2G7	2.168555183	0.001842	1.939320729	0.00536	0.397341062	0.579475
DTYMK	-0.472918843	0.001847	-0.253728395	0.08867	-0.220509599	0.13707
PRPF6	-0.235129296	0.001847	-0.159378398	0.03278	-0.146996136	0.048352
TCTN3	-0.320659744	0.001846	-0.280137233	0.00624	-0.372992024	0.000275
TRAF6	0.474637341	0.001847	0.480462402	0.00157	0.557431741	0.000234
GDF10	1.736242542	0.001856	0.304385669	0.59616	0.592366001	0.297352
ZDHHC8	0.534706263	0.001857	0.267280292	0.12257	-0.095276369	0.586256
NCOA6	0.491254965	0.001866	0.036253102	0.81926	0.265141431	0.093776
VPS54	-0.22848466	0.001875	-0.005949297	0.9345	-0.046359148	0.521699
CT83	2.963229282	0.0019	2.715967014	0.00454	2.939668933	0.001918
AEN	-0.538540804	0.001911	-0.46927516	0.00657	-0.364156749	0.034436
CLNS1A	-0.295732175	0.00191	-0.223550319	0.01838	-0.279610085	0.003177
CYTH1	0.430478524	0.001907	0.422928288	0.00228	0.627207768	5.64E-06
GNRH2	2.615144079	0.001909	0.012064608	0.98934	-1.761628978	0.077924
TNFRSF10D	0.583410021	0.001918	0.307908244	0.10164	0.563165952	0.002723
SIN3B	0.419980369	0.00192	0.054529031	0.68778	0.1437287	0.287985
ETV1	0.662567292	0.001935	0.481903007	0.02414	0.806189661	0.000158
NDC1	-0.446313803	0.001937	-0.21822971	0.12855	-0.214520659	0.13504
GLIPR1	0.96110918	0.001943	0.654609499	0.0348	0.745597175	0.016178
CLEC2B	0.983176744	0.001953	1.014119624	0.00133	0.768947557	0.015372
MLF2	0.259388555	0.001956	-0.104029744	0.21639	0.015362616	0.854479
ARMC1	-0.279283917	0.001961	-0.163685311	0.06683	-0.023258517	0.793337
MECOM	-0.661695906	0.001962	-0.259861239	0.22317	-0.362722755	0.089126
MRPL27	-0.383646351	0.001974	-0.176643269	0.1475	-0.10847121	0.371857
LATS2	0.580053944	0.00198	0.229066941	0.22213	0.534051384	0.004236
POP4	-0.384250346	0.001982	-0.085495449	0.48245	-0.110630636	0.362618
RTN3	-0.307222873	0.001997	-0.272493495	0.0059	-0.286473458	0.003784
ARHGEF40	0.620969718	0.002016	0.476637408	0.0177	0.407167655	0.042939
FAM84B	-0.576924795	0.002013	-0.184431974	0.32237	-0.151093039	0.417435
RPL9P7	-1.789124768	0.002016	-1.573669386	0.00577	-1.291865028	0.021909
SAP30	-0.411292135	0.002017	0.027517623	0.82865	0.050149911	0.692054
TRAF3	0.453203951	0.002013	0.172825508	0.23969	0.485871431	0.000897
TRIM47	0.991233966	0.002018	0.808616261	0.01226	0.497934704	0.124757
RMDN1	-0.363220432	0.002019	-0.177201261	0.12567	-0.201457887	0.082543
DICER1	0.408734502	0.002027	0.144579184	0.27486	0.482197375	0.000268
TGFBR3	0.759606609	0.00203	0.327969012	0.18293	0.273574367	0.26677

PTP4A3	0.990619314	0.002038	0.086764272	0.78844	0.48153466	0.134488
RPS25	-0.32960493	0.002041	-0.039852281	0.70797	-0.009831286	0.926337
ELF5	-0.90926535	0.00205	-1.130554364	0.00012	-0.853494681	0.00332
PGC	-2.200119145	0.002051	-1.265823086	0.07501	-1.93690988	0.006536
RPL22L1	-0.61755165	0.00205	0.002873305	0.98846	-0.24450695	0.219028
CKS2	-0.449054109	0.002055	-0.120960828	0.40117	-0.055475302	0.699633
TOR1B	0.431045935	0.002063	0.499396861	0.00031	0.358031926	0.010184
LSM11	-0.268334513	0.002075	-0.064256486	0.44409	-0.009040243	0.913336
ZSCAN2	0.562703507	0.002075	0.174095342	0.34282	0.474057161	0.0088
IMMP1L	-0.408775489	0.002083	-0.106794961	0.40627	-0.275010963	0.031327
FAM118B	0.392456582	0.002091	0.591233875	2.73E-06	0.643482669	3.01E-07
NDUFB2	-0.415768201	0.002103	-0.11733477	0.37894	-0.1742892	0.191238
CSNK2A1	-0.336062875	0.002104	-0.201874658	0.06415	-0.265222638	0.014936
ORC5	-0.422024197	0.00211	-0.115925869	0.38317	-0.114545172	0.388656
ARNT	0.353258506	0.002117	0.176903075	0.12334	0.223375297	0.051138
GPN3	-0.553651697	0.00212	-0.0385308	0.82589	-0.23481405	0.180747
TSC1	0.282029204	0.00212	0.258501115	0.00474	0.300806166	0.000974
SCRN1	0.248663583	0.002123	0.130479516	0.10655	0.361571568	7.33E-06
ATP5SL	0.421391992	0.002127	-0.011464891	0.93373	0.156640911	0.253645
SPICE1	-0.470704221	0.002127	-0.228930631	0.13208	-0.476544557	0.001762
PTGS2	1.798036753	0.002133	1.388576112	0.01772	1.24038866	0.034166
SNRNP200	0.278228914	0.002151	0.031204311	0.73056	0.139325336	0.123837
ABCA12	1.890430674	0.002153	1.437142533	0.01995	1.085967948	0.079114
BACH1	0.421544028	0.002154	0.173884331	0.20543	0.228071589	0.096725
NUCB2	-0.693622905	0.002157	-0.084768551	0.70525	-0.339655437	0.129637
RP11-3J10.4	0.55925306	0.002166	0.564542613	0.00181	0.704117069	8.89E-05
DMBT1	1.90687317	0.002168	2.618786337	2.53E-05	1.895851955	0.002298
THBS3	0.547502348	0.002169	0.603910354	0.00063	0.516293349	0.003591
CLK3	0.456455409	0.002174	0.103073035	0.48927	0.481628477	0.001203
RP11-20I23.1	6.807466425	0.002177	5.351233517	0.01638	5.131625966	0.021453
ATP5S	-0.50565596	0.002186	-0.251867203	0.12478	-0.533870134	0.001102
CLUAP1	-0.487996562	0.002185	-0.355146025	0.02441	-0.41068328	0.009255
RPS19	-0.428424524	0.002183	-0.275655638	0.04856	-0.390156755	0.005245
STIM1	-0.489565893	0.002192	-0.205747575	0.19739	-0.232668769	0.144874
HIST4H4	-0.513945685	0.002201	-0.195196608	0.24134	-0.18486859	0.266765
CTSS	0.726397895	0.002211	1.301750482	3.74E-08	0.328556267	0.166279
MAP3K2	0.325477269	0.002211	0.24881559	0.01894	0.193885019	0.067457
SHC2	0.569635378	0.002209	0.069162025	0.71092	0.352774904	0.057728
SLC25A37	0.3895637	0.002211	-0.01593748	0.90066	0.167891916	0.186403
CHMP4BP1	1.554283312	0.002214	0.536028046	0.31629	0.822897751	0.11489
TAF1C	0.544772313	0.002215	0.280319917	0.11598	0.242846768	0.173946
TMEM106B	-0.302571656	0.002223	-0.123362385	0.21044	-0.258075327	0.008875
RORA	0.770314748	0.002231	0.599650824	0.01726	0.667168727	0.007998
PPIE	-0.290646069	0.002236	-0.172360384	0.06402	-0.219506849	0.017829
11-Sep	0.357098861	0.00224	0.385016369	0.00098	0.627112185	7.74E-08
ACSM3	-0.657246522	0.002243	-0.359887383	0.09241	-0.028593794	0.893291
EPB41L3	0.891951871	0.002244	0.099009775	0.73578	0.689641822	0.018021

HDGFRP2	1.125641169	0.002246	0.499594435	0.17706	0.442283227	0.232221
AKAP5	0.60806241	0.002248	0.185071476	0.35508	0.783626418	7.35E-05
GUCY1A2	-0.740946975	0.002251	-0.76641126	0.00157	-0.500200588	0.038882
PPIH	-0.325849361	0.00225	-0.314438636	0.0028	-0.172372209	0.096434
APOPT1	-0.37591953	0.002256	-0.027235426	0.81968	-0.0671429	0.576195
RCN1	0.395176282	0.002262	0.327565442	0.01131	0.104727299	0.418415
C12orf23	-0.332435654	0.002268	-0.082087144	0.44855	-0.094350165	0.383777
PEBP1	-0.300597224	0.002268	-0.291502991	0.003	-0.220529577	0.024507
ACAN	2.397501369	0.002274	0.673346889	0.40283	0.07971065	0.922058
PLA2G6	0.661528743	0.002272	0.573195009	0.00843	0.45269508	0.036722
PPP1R18	0.808428475	0.002274	0.403471327	0.12837	0.371951825	0.160418
ZNF566	-0.639272342	0.002272	-0.423600295	0.04101	-0.383117718	0.063675
CABIN1	-0.464503081	0.002277	-0.480113617	0.0016	-0.690878579	5.66E-06
RAB31	0.846029644	0.002276	0.990609365	0.00034	0.797447167	0.003981
SLFN5	0.816196746	0.002283	1.047619086	8.90E-05	0.756043214	0.004704
TATDN1	-0.389139784	0.002285	-0.067550152	0.58991	-0.133289616	0.287176
EXOSC2	-0.452241423	0.002288	-0.231323419	0.1144	-0.294546631	0.044768
DNAJC24	-0.453579696	0.002292	-0.190074607	0.18756	-0.187933749	0.19187
HLA-E	0.426679013	0.002293	0.547569076	8.82E-05	0.306861082	0.028175
RASA3	0.67880598	0.002292	0.311307209	0.16524	0.536654238	0.015527
MRPL50P2	2.205051183	0.002295	1.273723107	0.08187	2.559725507	0.000368
CALM1	-0.325170895	0.002298	-0.253058356	0.01754	-0.196736987	0.06473
IRS1	0.445563658	0.002299	-0.295153928	0.04402	0.211444928	0.148012
TNFRSF1B	0.950600003	0.002299	0.708505786	0.02254	0.201494123	0.525037
POLE2	-0.71900256	0.002302	-0.225062217	0.33234	-0.160712229	0.487221
EM256-PLS(	0.655116892	0.002308	0.179608293	0.40796	0.039892219	0.854305
DDA1	0.467706643	0.00232	0.161656377	0.2938	0.193661905	0.207509
MRPS31	-0.3458995	0.002322	-0.006951028	0.94926	0.091746256	0.397697
AC006978.6	-0.621438737	0.002329	-0.226347097	0.24135	-0.188136005	0.32607
ING2	-0.437448842	0.002329	-0.194404649	0.16659	-0.284477442	0.043178
LTA4H	-0.374460925	0.00233	-0.251644671	0.04015	-0.313940529	0.010479
SH3D21	0.699121174	0.002328	0.440579026	0.05544	0.136387012	0.555051
AC004980.1	1.573424163	0.002336	0.85824673	0.1086	0.439088178	0.423347
MCM5	-0.501169672	0.002343	-0.434404667	0.00812	-0.447486588	0.006383
PRKAR1B	-0.372604886	0.002345	-0.401249464	0.00098	-0.454473442	0.000232
ZNF706	-0.320820672	0.002348	-0.034164607	0.74204	-0.03226367	0.755251
TNIP2	0.444905803	0.002351	0.505902035	0.00048	0.42249971	0.003589
TMPO	-0.394586063	0.002354	-0.163177688	0.20767	-0.097119427	0.453147
UHMK1	-0.200305356	0.002358	-0.043280553	0.50663	-0.185724197	0.004484
CACNA1C	0.845501891	0.00236	0.311192914	0.2651	0.397040095	0.154226
FYB	-2.15866535	0.002364	-0.945679447	0.13285	-1.49815089	0.030803
FIS1	-0.378797933	0.002366	-0.194445962	0.11398	-0.18547841	0.131134
P1-182O16.	1.8604643	0.002373	1.53440062	0.01286	1.4230687	0.021369
MAML2	0.449504315	0.002377	-0.083558146	0.5738	0.136823253	0.355417
SLC30A9	-0.357581062	0.00238	-0.015547043	0.89448	-0.008606559	0.941467
FBLIM1	0.422286815	0.002384	-0.101832435	0.46415	0.29125026	0.035987
P11-261C10	1.004919491	0.002386	0.285270607	0.39876	0.029791127	0.930465

LCAT	0.653350989	0.002391	0.365345602	0.09124	0.53918146	0.01143
MAEA	0.282471971	0.002394	0.193020856	0.03656	0.284559865	0.002016
UBE2V2	-0.26314661	0.002401	-0.077545063	0.36521	-0.024257968	0.776295
CYTIP	-1.523212986	0.002419	-0.254436671	0.59952	-1.08155067	0.027766
ITGAE	-0.650843794	0.002446	-0.226725777	0.2811	-0.163133023	0.43511
SCARF2	1.166954871	0.002446	0.388777362	0.32483	0.547095961	0.161649
CDC37	0.324033051	0.002448	0.287198159	0.00713	0.264641867	0.013253
HNRNPR	-0.262895813	0.002465	-0.220102287	0.0111	0.048753117	0.57304
KIF9	-0.661437002	0.002465	-0.470259186	0.0292	-0.392134522	0.067274
CLDN2	-1.237012606	0.00247	-0.735125163	0.07106	-1.405897909	0.000578
CHMP1B2P	-1.030863898	0.002481	-0.235792009	0.46886	-1.363765218	6.94E-05
RPSAP53	-0.734660186	0.00249	-0.009926986	0.96663	-0.258144599	0.27849
HERPUD2	0.46453689	0.002495	0.384781706	0.01208	0.433182915	0.004682
.A16c-17H1.:	-0.816747395	0.002494	-0.075471712	0.76449	-0.21359525	0.398719
LYSMD4	-0.542218902	0.002511	-0.363748937	0.04037	-0.268234697	0.128995
TMEM242	-0.427654624	0.002529	-0.34204859	0.01365	-0.295709885	0.032959
TBC1D10A	0.512143274	0.00253	0.4135856	0.01451	0.089399507	0.600114
MRPS26	-0.466688164	0.002533	-0.397750025	0.00934	-0.778677601	5.13E-07
ANPEP	2.745586502	0.002541	0.811020037	0.37296	0.007829819	0.993143
OR2I1P	2.94805712	0.002542	3.902964532	4.46E-05	1.853527113	0.06555
DTL	-0.664000817	0.002545	-0.668627557	0.0023	-0.649762058	0.003037
DEF6	-0.614462762	0.002557	-0.459457438	0.02121	-0.380450464	0.056226
NCOA2	0.294902134	0.002557	0.250131936	0.01028	0.184420897	0.058327
?P11-204M4.	-1.619758119	0.002556	-0.194086878	0.67935	-0.280349622	0.551062
TNFRSF11B	1.088791658	0.002559	1.389978034	0.00012	0.72036259	0.046108
MAVS	0.239160281	0.002566	-0.028454294	0.72008	0.028725179	0.717106
MRPL11	-0.383334038	0.002566	-0.234549386	0.06127	-0.254060621	0.042819
AMOTL2	0.659229975	0.002573	0.083097546	0.70466	0.535165862	0.014333
PATZ1	-0.335922032	0.002576	-0.374086035	0.00072	-0.366319747	0.000936
FBXO21	-0.338505145	0.002579	-0.278901983	0.01245	-0.231133503	0.037854
KLC3	1.042095225	0.00258	0.72756926	0.03764	0.750642203	0.030411
JPH3	1.091416036	0.002585	0.900554911	0.01286	0.511899815	0.168695
CD40	1.733838051	0.002593	2.502558265	1.03E-05	1.254651604	0.02949
KIF21A	-0.471930193	0.002595	-0.178284082	0.25366	-0.178227956	0.253664
MCL1	0.45462361	0.002594	0.493488047	0.00107	0.467398013	0.001942
MAML1	0.600062582	0.002603	0.040134732	0.84052	0.361631198	0.069457
NAMPTL	1.078093051	0.002603	0.990189695	0.00567	0.981337345	0.006117
STRN4	0.298264228	0.002601	0.079131576	0.42563	0.143111408	0.148658
PUS7	-0.543388644	0.002618	-0.418286434	0.02011	-0.258964758	0.148941
TRIT1	-0.439198779	0.002637	-0.329346765	0.02269	-0.143324761	0.320048
RFT1	-0.403138409	0.002641	-0.17777929	0.18102	-0.210971344	0.111899
TMEM189	0.425416492	0.002643	0.243060594	0.0856	0.459500974	0.001135
ARHGEF37	0.984156488	0.002659	0.955521482	0.00328	0.5950967	0.07015
CEACAM1	1.288680075	0.002656	0.477931735	0.26537	0.2680894	0.53216
RNF138	-0.388477868	0.002657	-0.335566133	0.00884	-0.04096765	0.748051
LRP2	1.718232841	0.00266	1.429796903	0.01234	1.651434969	0.003808
ARHGAP10	-0.362666169	0.002668	-0.290572658	0.01469	-0.258844315	0.030058

CPD	-0.397842716	0.002678	-0.315596448	0.01718	-0.329013005	0.012986
STARD7	-0.301373138	0.002679	-0.206116125	0.03949	-0.053383892	0.593577
ABHD17B	-0.364893582	0.002684	-0.126487566	0.28864	-0.160349788	0.178232
CCDC120	0.667146634	0.002682	-0.009515491	0.96635	0.094476319	0.674354
LARS2	-0.359204402	0.002683	-0.062594371	0.59805	-0.231742485	0.050842
HIST1H4G	-1.101657861	0.002697	-0.379197136	0.29136	0.01146778	0.974374
FYCO1	-0.345935817	0.002701	-0.176366499	0.1223	-0.301686743	0.008349
NSRP1	0.47405798	0.002711	0.106374994	0.5025	0.148894513	0.344549
OVCA2	0.800940316	0.002712	0.714639991	0.00735	0.600300488	0.024511
TBC1D2B	0.477505018	0.002709	0.402343605	0.01138	0.331216915	0.03732
WDFY1	-0.210058332	0.00272	-0.06134507	0.37765	-0.069999837	0.313814
MOB1A	-0.205833213	0.002724	0.004457472	0.94798	0.018607386	0.785239
SLC6A20	1.653620773	0.002723	1.175184646	0.03462	0.61651308	0.267177
JAK3	0.889954379	0.00273	0.649638662	0.02874	0.572786014	0.053892
DNAL4	-0.454373664	0.00275	-0.297924683	0.04295	-0.296155114	0.044465
PRPF40A	-0.272741018	0.002754	-0.183703782	0.04323	0.060938678	0.501801
MYCBP	-0.469176019	0.002769	-0.121511125	0.43458	-0.266111398	0.087243
TBC1D17	0.446499281	0.002785	0.16237467	0.27988	-0.040365558	0.789219
AP3M2	-0.454922664	0.00279	-0.28280754	0.06086	-0.045682807	0.760994
IL2RG	0.869702535	0.0028	0.335347274	0.25095	0.527217233	0.070308
MMADHC	-0.273734268	0.0028	0.057378329	0.52607	-0.094040777	0.299805
ABHD10	-0.361869356	0.002807	-0.201151193	0.08983	-0.164861018	0.164453
C19orf43	0.310424335	0.002806	0.060241396	0.56243	0.239737236	0.020693
GPR160	-0.609913524	0.002808	-0.148164026	0.46577	-0.196441675	0.333624
PMPCB	-0.293539494	0.002806	-0.065340066	0.50128	-0.0498347	0.607374
MAP1LC3B	0.525051657	0.002811	0.486012547	0.0056	0.622664857	0.000376
TMEM138	0.448270692	0.002817	0.050149678	0.73813	0.110901108	0.459548
ARNTL	0.757254726	0.002827	0.883387501	0.00043	0.784932059	0.001807
NEDD8	-0.231648141	0.002832	-0.1671492	0.0295	-0.09652336	0.206892
RPL13A	-0.367430263	0.002831	-0.196223908	0.11081	-0.331714701	0.007023
SYNJ2	0.412987261	0.002836	0.127756944	0.35604	0.383928727	0.005414
RPSAP58	-0.843795067	0.002844	0.307097455	0.27245	0.332642978	0.234411
DDAH1	-0.327691693	0.002859	-0.391927615	0.00036	-0.345906137	0.001617
GLYCTK	-0.614009423	0.002857	-0.564017735	0.00592	-0.559856899	0.006194
RPLP0P6	-1.739896546	0.002858	-0.095821257	0.85263	-0.724088445	0.171474
TRIM62	0.705890266	0.002858	0.4193954	0.07682	0.157047635	0.508937
CASP6	-0.396619842	0.002866	0.033022266	0.7981	-0.243416205	0.061227
RAD54L	-0.753719419	0.00287	-0.754719969	0.00275	-0.529147327	0.0348
PHF23	0.393421412	0.002877	0.127591605	0.33257	0.356541926	0.006637
IRS4	1.823326007	0.00288	2.903368282	1.65E-06	2.258683441	0.000203
ZNF165	-0.60190523	0.002882	-0.319542954	0.10298	-0.15187831	0.432718
CDC37L1	-0.511660171	0.002885	-0.174856206	0.3007	-0.175065672	0.299008
MPP6	-0.645112739	0.002889	-0.228530822	0.28818	-0.061120603	0.776192
COL15A1	1.088331598	0.002899	0.285418951	0.43854	0.477713722	0.212989
E2F8	-0.601569317	0.0029	-0.179410241	0.36886	-0.304862954	0.12717
FEN1	-0.535600135	0.0029	-0.382473695	0.03257	-0.182972357	0.305771
GPR50	1.928282452	0.002905	0.424300826	0.51728	1.882738605	0.003612

EIF4ENIF1	0.429790669	0.002923	0.145296331	0.31454	0.507055865	0.000419
MPC2	-0.413443493	0.002924	-0.178250179	0.19607	-0.300205716	0.02953
ZNF107	-0.509198343	0.002922	-0.321443154	0.05689	-0.131947017	0.430739
ISG20	0.793167901	0.002928	0.506735787	0.05768	0.873147291	0.001029
TRAPPC6A	-0.536473775	0.002929	-0.455384644	0.01067	-0.646291495	0.000323
RBBP8NL	-0.848547857	0.002935	-0.700219917	0.01277	-0.761934079	0.006752
DBN1	0.663950589	0.002942	0.186587779	0.40342	0.481989837	0.030794
PLSCR1	0.377278884	0.002942	0.624055005	7.77E-07	0.56914861	6.73E-06
MTIF2	-0.431637472	0.002955	0.018323917	0.89861	-0.206713741	0.151045
TRIM16	0.534392637	0.00296	0.539365913	0.00265	0.504447191	0.004954
SEC61A1	0.235420549	0.002976	0.063226024	0.42449	0.093341747	0.238527
FAM167A	0.897924229	0.002997	0.842575602	0.00513	0.963215841	0.001289
PARP8	0.377751584	0.002997	0.475590369	0.00018	0.393234727	0.001911
TRAPPC1	0.31228011	0.002997	-0.016969348	0.87272	0.044491096	0.672849
VWA8	-0.545890625	0.002996	-0.676633158	0.00022	-0.577275832	0.001625
NDUFC2	-0.308029378	0.003007	-0.181274001	0.07864	-0.183895432	0.074067
TPT1	-0.317974148	0.003009	0.023613244	0.82557	-0.103289218	0.335061
HTATSF1	-0.267856806	0.003015	-0.184467285	0.0394	-0.093187525	0.296429
POLH	-0.45094711	0.003026	-0.410345449	0.00681	-0.375667881	0.012869
RP11-84C13.	-0.479362837	0.003025	-0.185200895	0.24488	-0.059969634	0.705246
FGFR1	0.731134885	0.003032	0.427925555	0.0827	0.407892256	0.098215
GNPAT	-0.298126908	0.003039	-0.178935567	0.07281	-0.072495224	0.465497
IMPA2	-0.489223539	0.003038	-0.444411822	0.00689	-0.599736371	0.000273
RNASET2	-0.447940345	0.003044	-0.243357812	0.10513	-0.585154949	0.000107
GNRHR2P1	-1.213328575	0.003053	-0.347721375	0.38258	-0.245870769	0.535864
MRPL21	-0.370372578	0.003066	0.023976073	0.84319	-0.100991989	0.405876
SYNGAP1	1.034960496	0.003067	-0.201065994	0.57891	0.607098095	0.082334
HHLA2	1.275899526	0.003082	1.406021386	0.0011	1.078020021	0.012389
TMPRSS2	0.747072469	0.003086	0.826476409	0.00106	0.543911998	0.031183
UBASH3A	2.192494521	0.003096	2.135551044	0.00369	1.965594946	0.008027
SUZ12	-0.350659558	0.003099	-0.106549849	0.36586	-0.057660952	0.624
EPB41L1	-0.668783369	0.003104	-0.63725379	0.00484	-0.408795466	0.070334
ADCK2	-0.574853786	0.003113	-0.13219103	0.48999	-0.400227069	0.038004
LRRC3	0.493862546	0.003113	-0.019646358	0.90892	0.200779531	0.231389
RAPGEF2	0.331347652	0.003116	0.269078351	0.01624	0.392494869	0.000444
ATF7IP2	-0.481080503	0.00312	-0.269115406	0.0968	-0.464964308	0.004157
HARS2	-0.281007938	0.003121	-0.024326273	0.79311	-0.207467347	0.026578
MAP2K2	0.439309201	0.003121	0.207340013	0.16292	0.011640832	0.937642
TLE3	0.73907846	0.003122	0.270440135	0.28048	0.422765548	0.091185
KCNA1	1.923253431	0.003125	1.484454057	0.02443	1.470358996	0.025349
DCTN2	0.261342936	0.003127	0.183983641	0.03664	0.338798895	0.000113
PIGV	-0.373736051	0.003135	-0.02480411	0.84057	-0.414645208	0.000874
RPS7P11	-1.184859777	0.003135	-0.140468255	0.70866	-0.712015398	0.063867
PHKA2	-0.484034018	0.003164	-0.358955713	0.02833	-0.355098075	0.030077
TOMM5	-0.338918371	0.003173	0.01674769	0.88321	-0.064761785	0.570102
C2CD4C	1.24931483	0.00319	0.76547865	0.07326	0.877422137	0.039069
NLN	-0.349231692	0.003189	-0.302181707	0.01036	-0.22906682	0.05117

FBXO5	-0.41749373	0.0032	-0.244520554	0.08136	-0.035934268	0.796758
SLC10A2	3.060995838	0.003205	0.85557724	0.4163	0.504113679	0.633354
KLHL29	0.453639818	0.003208	-0.039711813	0.79804	0.349761581	0.022565
FAM214B	0.657068747	0.003211	0.511401478	0.02177	0.511601821	0.021717
PLEKHM1	0.73472875	0.003217	0.054480557	0.82628	0.308627593	0.213696
CAMK2A	1.535456376	0.00322	0.087462008	0.87393	0.555552909	0.300978
SEMA3C	0.62842943	0.003222	1.134538976	1.02E-07	0.530201499	0.01292
RPL30	-0.317235041	0.003231	-0.149942045	0.16361	-0.063615606	0.554406
GRSF1	-0.241170701	0.003232	-0.131824069	0.10554	-0.141384589	0.082752
PRIM1	-0.771366804	0.003242	-0.585041175	0.0238	-0.304519416	0.237492
SNRPD2	-0.36363517	0.003253	-0.2146649	0.08121	-0.191005149	0.120404
GTF3A	-0.386023395	0.003256	-0.193149272	0.13924	-0.319510489	0.014427
MMD	0.432606519	0.003259	0.275397919	0.06084	0.433244843	0.003114
TES	-0.25672909	0.00326	-0.090958993	0.29536	-0.140068823	0.106628
UNC93A	-1.152797949	0.003261	-0.975915234	0.0124	-1.281239029	0.001039
PIFO	-1.242055594	0.003277	-0.134611573	0.74167	-0.627973307	0.125725
MYO1H	1.868766252	0.003285	1.173257773	0.07463	1.579157707	0.012809
MICALL1	0.379947332	0.003287	0.203563576	0.11584	0.252982877	0.050668
APMAP	-0.33237394	0.003304	-0.106911693	0.34164	-0.216173836	0.054711
VPS37B	0.401366051	0.003303	0.210304953	0.12358	0.292196946	0.0322
PODN	0.982921561	0.00332	0.365097156	0.28693	0.790374638	0.017989
DENND1A	0.456515872	0.003334	0.007360585	0.96239	0.150475704	0.333988
FAIM3	1.248281715	0.003348	1.505033999	0.00038	1.002434771	0.018962
FUCA2	-0.466278484	0.003346	-0.406101391	0.01036	-0.417589805	0.008366
NOL11	-0.316873516	0.003353	-0.126104548	0.23866	-0.095751555	0.369974
PSPH	-0.414862585	0.003354	-0.444025711	0.00157	-0.844228943	2.97E-09
TUBA1A	0.790858321	0.003357	0.425765063	0.11437	0.73486123	0.006417
PDE4DIP	-0.45142046	0.003367	-0.1086598	0.47679	-0.370409768	0.015647
PDLIM1	0.361128029	0.003366	0.334668333	0.00647	0.367662314	0.002757
CLN5	-0.660826523	0.003373	-0.200167686	0.36643	-0.39298707	0.076841
KXD1	0.404209474	0.003381	0.277563685	0.04385	0.40137852	0.003503
RUNX1T1	0.99494258	0.003383	0.575465518	0.0905	0.673685182	0.047202
ACBD6	-0.347502751	0.003398	-0.265117311	0.02411	-0.289067936	0.013855
ANKRD49	-0.393422947	0.003394	0.045784896	0.72915	-0.186818907	0.157143
DDX50	-0.270801868	0.003393	-0.081700084	0.37124	-0.050625601	0.578638
ODAM	-1.794419621	0.003397	-0.734041845	0.22494	-0.926600292	0.125809
WDR3	-0.46027847	0.003397	-0.255816651	0.10126	-0.234911357	0.132211
TCF7L2	0.720470591	0.003401	0.091331237	0.71094	0.584417961	0.01751
RBBP9	-0.471761796	0.003406	0.064428847	0.68715	-0.18193628	0.25557
GLB1	-0.458343381	0.00341	-0.412808484	0.00829	-0.650724323	3.20E-05
NQO2	-0.461463006	0.00341	-0.674776595	1.84E-05	-0.558156739	0.000363
CALD1	0.556500174	0.003416	0.16893558	0.37424	0.479700631	0.011601
ZNF33A	-0.326739916	0.003421	-0.247477761	0.0258	-0.092874768	0.402091
MRPL23	-0.633550246	0.003429	-0.349513231	0.09972	-0.477025511	0.025145
DLK1	1.684208857	0.003436	1.202407285	0.03676	0.486174394	0.3987
PTGR2	-0.566037716	0.003449	-0.2060652	0.28263	-0.362476045	0.058244
SQRDL	0.463777942	0.003452	0.70115519	8.84E-06	0.281200093	0.075838

NCR3LG1	0.515959518	0.003457	0.055921079	0.75235	0.455851974	0.009629
RPS24	-0.392595768	0.003495	-0.164234683	0.22165	-0.244781418	0.06853
NDN	0.376928611	0.003499	0.374675562	0.00358	0.372252786	0.003769
RCAN3	-0.678232394	0.003498	-0.607132365	0.0088	-0.2908997	0.209356
RP11-38O23.	-1.397262065	0.003517	0.122846387	0.76772	-0.69387912	0.111789
SLC9A1	0.791288802	0.003521	-0.067420892	0.80533	0.145057903	0.596222
TENM3	0.948236074	0.00353	0.336588208	0.30066	0.731352915	0.024432
COPS4	-0.369310193	0.003539	-0.128764126	0.30427	0.129533185	0.300052
GABPA	-0.233054783	0.003538	0.004269217	0.95674	0.009453937	0.90416
STAG3L1	1.006259449	0.003536	0.415717588	0.23901	-0.233032672	0.528804
RP11-742N3.	-1.021126788	0.003543	0.534020962	0.11699	-0.569177414	0.09904
ENTPD3	-0.813048709	0.003556	-0.369442627	0.17456	-0.680653977	0.013634
NAA60	0.413928569	0.003556	0.155418485	0.27347	0.329185918	0.020133
VILL	-0.520073343	0.003581	-0.416739582	0.01929	-0.641394489	0.000323
ZNFX1	0.326019961	0.003594	0.317005092	0.00452	0.255887911	0.022002
ZNF77	-0.495462256	0.003611	-0.261674843	0.10836	-0.314470491	0.052007
OXLD1	-0.575426569	0.003625	-0.512123552	0.00785	-0.683257903	0.000427
CCHCR1	-0.579251522	0.003632	-0.434612985	0.02741	-0.505699417	0.010291
IGF2	0.530868756	0.003631	-0.089199027	0.62509	0.242975748	0.183123
RAB11FIP5	0.482783588	0.003633	0.009835066	0.95298	0.072847441	0.662642
TNNI3	-0.982533578	0.003632	-0.954809904	0.00399	-0.353337852	0.249803
ADD3	-0.298397723	0.003639	-0.138394146	0.17664	-0.249024815	0.015073
GBAS	-0.318923348	0.003641	-0.183753411	0.09193	-0.319299293	0.003459
GBP2	0.560623491	0.003645	0.549055492	0.00439	0.687140292	0.000362
LSM4	-0.419958578	0.003656	-0.267463298	0.06261	-0.289778125	0.043584
CYP3A5	0.631815597	0.00367	0.547596067	0.01179	0.620660324	0.0043
KIF7	0.758435661	0.003668	0.230292472	0.38052	0.12675769	0.628284
TSEN2	-0.709728838	0.00367	-0.61584889	0.01139	-0.34357786	0.157194
GNA12	0.384802705	0.003675	0.076186576	0.56595	0.156486387	0.236249
TMEM130	0.952259162	0.003679	1.111689632	0.00057	0.416010471	0.205971
RP11-432B6.	-5.143241001	0.00369	-0.458039067	0.7572	-0.140671977	0.923975
SPTLC1	-0.235703649	0.0037	-0.06565135	0.41201	-0.177971971	0.026416
MADD	0.375509039	0.003703	0.244436542	0.05833	0.301611611	0.01946
HSD17B1	0.442595054	0.003707	0.065352281	0.6685	-0.028716331	0.850052
RRNAD1	-0.505341222	0.003715	0.022728252	0.89418	-0.280026104	0.102307
SMYD3	-0.587705325	0.003721	-0.463173747	0.02061	-0.518595772	0.010259
TTC5	-0.478362782	0.003719	-0.18746061	0.25091	-0.372867807	0.022409
USP16	-0.27668191	0.003721	-0.005023222	0.95755	0.029004259	0.758078
ZZEF1	0.475759096	0.003726	0.165229079	0.31391	0.166307793	0.311177
CYB5R1	-0.431364313	0.003739	-0.370030822	0.01217	-0.613961661	3.77E-05
LYPD6B	0.649613188	0.003752	1.233667622	2.73E-08	0.883392932	7.26E-05
DST	0.445629829	0.003761	0.396809219	0.00986	0.395915849	0.010033
ECHDC2	-0.453371451	0.003767	-0.253190321	0.10243	-0.249140444	0.108201
LRRC8C	0.498730272	0.003772	0.135648809	0.4327	0.159733641	0.353807
LLGL1	0.493983988	0.003782	0.207053905	0.22537	0.133663457	0.4339
MT-CO2	0.705986186	0.003787	0.186262793	0.44495	0.145332062	0.55117
COL5A1	0.739468012	0.00379	0.524016996	0.04019	0.074990423	0.769116

TOR4A	0.473830115	0.003794	0.165317506	0.31371	0.101544902	0.536136
RYK	-0.270164385	0.003809	-0.108652598	0.24214	-0.105350997	0.256032
TMEM158	1.303649089	0.003807	1.190525572	0.00817	0.601027598	0.185191
HNRNPU	0.216130997	0.003814	0.009787582	0.89572	0.157431237	0.0349
NOS1	0.920082069	0.003817	0.601457103	0.05993	0.209201368	0.519034
PCDH11X	1.665826444	0.003815	1.649855896	0.00414	0.068940103	0.906152
WARS2	-0.359344238	0.003812	-0.320637829	0.00877	-0.192722118	0.115948
ABHD4	0.555925856	0.003824	0.167120217	0.38579	0.032659268	0.865475
ARSE	-0.578037994	0.003824	-0.359411406	0.07054	-0.592075891	0.002935
STK36	0.49402262	0.003824	0.230987619	0.17614	0.301844444	0.076272
BAG1	-0.34484645	0.003838	-0.187020642	0.11306	-0.195789555	0.096607
GAL	1.700582273	0.003837	2.319184197	6.57E-05	0.691162217	0.249128
HNRNPA2B1	0.312390284	0.003838	-0.090880444	0.40047	0.078192847	0.469203
SMTN	0.634367452	0.003843	-0.303950239	0.16725	0.204685427	0.351193
COMMD9	-0.318344	0.003845	-0.061760053	0.56496	-0.174919186	0.103934
KIAA1324L	-0.519906492	0.003848	-0.311530051	0.08264	-0.152013023	0.396123
MRPL4	0.402143066	0.00385	0.019632637	0.88875	0.084429561	0.545305
FAM193B	0.443993051	0.003857	0.056406102	0.71401	0.237757482	0.121396
SMC3	-0.307122506	0.003856	-0.279463725	0.00838	0.153372858	0.146137
IL7	1.276856335	0.003873	0.836117266	0.06056	0.795791274	0.074766
MT-ND1	0.847708772	0.003872	0.374455761	0.20216	0.334177199	0.25502
PRR14L	0.336077834	0.003872	0.158934562	0.17135	0.303386845	0.008968
PCYOX1	-0.290321407	0.003885	-0.315698179	0.00166	-0.296005601	0.003173
CD55	0.877382927	0.003892	0.569755862	0.06094	0.852932975	0.004998
WRNIP1	-0.271667887	0.003904	-0.18539774	0.04671	-0.058619463	0.526995
PKD1L1	-1.34883529	0.003913	-0.265931254	0.5677	-1.091582998	0.0198
LSM7	-0.361443973	0.003927	-0.170057171	0.16799	-0.297711286	0.01602
OTUD1	0.599888559	0.003927	0.913100972	9.66E-06	0.947221123	4.33E-06
LTBR	0.437019395	0.00394	0.164555006	0.27688	0.27627118	0.067943
MAP2K7	0.777405159	0.003946	0.311871039	0.24879	-0.012670752	0.962659
MAPK11	1.128934752	0.003951	1.076857884	0.00551	0.660291402	0.09159
ZNRF1	0.440649681	0.003957	0.052813463	0.73161	0.261765418	0.086731
HMGB3P9	-1.787029927	0.003971	-0.424494628	0.41587	-0.735959768	0.166866
ODC1	-0.463731819	0.003983	-0.408138693	0.01112	-0.144522571	0.367738
RPS6	-0.377762181	0.003986	-0.309347977	0.01837	-0.297752277	0.023218
BTF3L4	-0.259284374	0.004003	-0.132524378	0.13929	-0.034139731	0.701668
HOMEZ	0.453344588	0.004	0.185859557	0.23726	0.152484942	0.332129
HSD17B11	-0.614571447	0.004004	-0.296630211	0.16318	-0.458325737	0.031279
KRT23	1.965706885	0.004008	1.42281766	0.03757	2.283181152	0.000821
SHMT1	-0.54874468	0.004016	-0.429519757	0.02309	-0.45237479	0.01656
ABCC5	-0.447152534	0.004037	-0.367248386	0.01749	-0.332555333	0.031317
RAB34	0.364735942	0.004035	0.147249983	0.24525	0.319673897	0.011524
TSN	-0.250098043	0.004037	-0.021604641	0.80157	-0.03113681	0.717182
ATL3	-0.222768909	0.004048	0.076158418	0.32014	-0.180371128	0.019153
HMGN1	-0.220543519	0.004048	-0.020580147	0.78772	0.06051504	0.428115
BLVRB	-0.450048512	0.004066	-0.343249151	0.02758	-0.369931115	0.017666
ANO1	0.861762021	0.004079	0.57666751	0.05463	0.297986232	0.320935

GLI3	0.874450593	0.004078	0.466013685	0.12646	0.377836859	0.215495
PPIL3	-0.341359794	0.004086	-0.229967724	0.05057	-0.114122783	0.328687
NDUFAF1	-0.436446183	0.004094	-0.029434444	0.83817	0.062190281	0.665965
TIMELESS	-0.482573267	0.004096	-0.352344657	0.0353	-0.287451215	0.085651
TANC2	0.640866106	0.004099	0.536913768	0.01619	0.225048113	0.313844
PDCD2L	-0.655829613	0.004111	-0.185304113	0.39383	-0.182296161	0.400674
GSS	-0.289995932	0.004125	-0.102448921	0.30406	-0.301606536	0.002616
HOXA3	1.082898345	0.004136	-0.046558952	0.90438	0.039834483	0.91673
SF3A1	0.398295536	0.004156	-0.046893611	0.73593	0.168153925	0.225845
ORC2	-0.354618966	0.004164	0.096869406	0.42899	0.034065037	0.780915
PTS	0.407401856	0.004164	0.534700178	0.00015	0.607287296	1.50E-05
AC141586.5	0.559425467	0.004178	-0.054223585	0.78443	-0.0683747	0.729535
ZC3H11A	0.193572957	0.004181	0.082427529	0.2222	0.07693523	0.254036
MOB4	-0.334879809	0.004187	-0.130645868	0.26026	-0.060615558	0.600806
HDHD2	-0.329623261	0.004199	-0.219390919	0.0528	-0.228609757	0.04318
GMPS	-0.283970188	0.004202	-0.080838481	0.41352	-0.064936599	0.510811
TMEM5	-0.360851353	0.004221	-0.20482478	0.0967	-0.145774503	0.23768
MBTD1	-0.297524129	0.004225	-0.213289709	0.03807	-0.153048999	0.134914
C5	-0.876860774	0.004234	-0.252263515	0.40712	-0.690277577	0.023583
FAM131A	0.506437175	0.004237	0.289331736	0.10181	0.277746478	0.114848
NFYB	-0.354656337	0.004237	-0.053514491	0.66353	0.212067462	0.083123
SEC24D	0.457317343	0.004244	0.091605082	0.56661	0.171971652	0.282072
C11orf31	-0.390878122	0.004254	-0.263385178	0.05143	-0.513593989	0.00016
NPEPL1	-0.544652996	0.004256	-0.520283418	0.00613	-0.246061641	0.191286
UGGT1	-0.28462411	0.004262	-0.044217071	0.6561	-0.016526211	0.867866
ZNF644	-0.21048608	0.004261	-0.065555412	0.37053	0.085734873	0.23987
CHD8	0.293272036	0.004272	0.225776094	0.02756	0.372111822	0.000278
MAP3K12	0.698488102	0.004279	0.201788802	0.4097	0.140953054	0.563875
NFU1	-0.338205093	0.00428	-0.010894237	0.92527	0.003372021	0.976833
TMEM144	-0.50752785	0.004285	-0.323166406	0.06723	-0.611277353	0.000565
LSM12	0.308085302	0.004289	0.065599196	0.54336	0.25099648	0.019504
FXYD6	0.677986434	0.004301	-0.382481574	0.11079	0.416491241	0.079646
GTF2F2	-0.313128619	0.0043	-0.140789854	0.19137	-0.082012233	0.443739
LRRC55	1.887926132	0.004304	1.392314926	0.03604	2.318898424	0.000414
RPL4	-0.332708374	0.004303	-0.191106286	0.10095	-0.169867993	0.144829
NEIL3	-0.421758376	0.004327	-0.250571578	0.08265	0.051146532	0.718517
TMEM99	-0.60267076	0.004327	-0.233225962	0.24966	-0.473240267	0.021073
CABP4	1.511382095	0.004339	0.81505508	0.12583	1.119092561	0.03274
MED29	1.004865632	0.004338	0.19190174	0.59357	0.345561184	0.329885
OR7E38P	0.61234886	0.004338	0.45942065	0.03211	0.509555565	0.01673
TENC1	0.65952312	0.004337	0.131851247	0.56879	0.233832295	0.312199
AC012501.3	-0.823417976	0.004351	-0.653269515	0.0204	-0.8638715	0.002402
GNG10	-0.428836239	0.004363	-0.024775901	0.86808	-0.040701952	0.784882
RAB11B	0.548798554	0.004366	0.101615734	0.60063	0.169381284	0.383331
COIL	-0.253503946	0.004375	-0.059999208	0.4917	-0.070244693	0.41889
RPL14P1	-1.405369623	0.00438	-0.546095241	0.22759	-0.840168921	0.067599
CLK1	-0.46655642	0.004388	-0.586727277	0.00034	-0.355060773	0.029886

EEF1A1P5	0.406523299	0.004384	0.165826875	0.24516	-0.049690972	0.727661
TMEM251	-0.606671828	0.004388	-0.197904855	0.31595	-0.355004951	0.075973
JAZF1	0.561226028	0.004396	0.249840584	0.20444	0.631327704	0.00123
RNF13	-0.339658672	0.004406	-0.025150008	0.83144	-0.301413328	0.010954
SLC43A1	-0.644564612	0.004405	-0.776123613	0.00059	-0.676145254	0.002743
TLE4	0.400756391	0.004415	0.039684029	0.77826	0.552586733	8.05E-05
TM9SF3	-0.250936318	0.004415	0.097552671	0.26769	-0.079760926	0.364938
IRF2	0.581059531	0.004422	0.091848737	0.65303	0.07519404	0.712721
TAPT1	0.409761383	0.004423	0.311783825	0.02928	0.42049527	0.003196
ZNF697	0.500352229	0.004432	0.515484238	0.00326	0.327517341	0.062181
NEK4	-0.287228053	0.004438	0.016793208	0.86667	-0.217212761	0.029871
SAP130	0.32718968	0.004455	-0.07282223	0.52994	0.134308242	0.242668
EMG1	-0.383500508	0.0045	-0.014504358	0.91367	0.024363133	0.855274
UBR7	-0.427147031	0.004508	-0.270651205	0.06778	-0.197230523	0.182211
NME1-NME2	-0.341635702	0.004512	-0.068233943	0.56933	-0.206642853	0.085012
ZER1	0.318921256	0.004513	0.230551435	0.04032	-0.072649487	0.519111
FAM83H	17.59449634	0.004531	-0.178510771	0.97721	15.96596831	0.010564
POP7	-0.348540436	0.004545	-0.137358413	0.25145	-0.246011352	0.040557
TCF21	0.752919781	0.004549	-0.246847804	0.35564	0.357509767	0.178342
ZNF252P	-0.343867663	0.00456	-0.144632335	0.22843	-0.253415009	0.034954
KANK1	-0.403053818	0.004562	-0.172318458	0.22313	-0.372501619	0.008559
LOX	0.934089914	0.004568	0.944331234	0.00411	0.363774162	0.2698
UBQLN2	0.31200718	0.004582	-0.047724936	0.66668	0.058243198	0.596868
KIAA0556	0.546132533	0.004589	0.346388114	0.07185	0.328853541	0.08725
SNAI1	0.626202232	0.004589	-0.176944601	0.4356	0.312727277	0.158052
BBS2	-0.434893548	0.004594	-0.027844726	0.85448	-0.479661939	0.001655
GPAA1	0.488155394	0.004604	0.273572331	0.11108	-0.108142845	0.530387
PXN	0.572147169	0.004611	0.066482186	0.74243	0.073267158	0.71702
TAF1D	-0.313519933	0.00461	0.067978225	0.53749	-0.024701417	0.82264
ZNF287	-0.759271112	0.004609	-0.235046761	0.37898	-0.436540187	0.100883
ARID5A	0.678433981	0.004616	0.393853457	0.10142	0.194483883	0.420636
POLR1E	-0.377169776	0.004618	-0.400657828	0.00246	-0.356900799	0.006725
CCDC115	-0.405315981	0.004628	-0.439428884	0.00197	-0.488939048	0.000564
SLC5A5	1.010820847	0.004648	0.8495137	0.01728	0.613176613	0.088867
UBE2K	0.329825505	0.00465	0.318929724	0.00606	0.279785484	0.015935
PFN1	0.424571606	0.004666	0.236444317	0.11511	0.481971248	0.001314
PHC1	0.679880761	0.004665	0.060564636	0.80141	0.593897346	0.013353
SNF8	-0.276977079	0.004664	0.047875098	0.61848	-0.06781412	0.480404
SH3YL1	-0.365863256	0.004694	-0.275189345	0.03309	-0.378897032	0.00337
DYRK4	-0.37557897	0.004698	-0.089490304	0.48987	-0.081920914	0.524608
ALOX15B	1.505008991	0.004721	0.343301397	0.53882	-0.105968196	0.853448
FXR2	0.330137703	0.004735	0.072009842	0.53648	0.347654138	0.002773
MCMBP	-0.234495112	0.004755	-0.068509336	0.40581	-0.027613876	0.736925
MRPS16	-0.352296689	0.004754	-0.178012248	0.15176	-0.268154599	0.030989
NR1H2	0.433328572	0.004754	0.241531072	0.11349	-0.145479044	0.345906
HSPA4	-0.275329722	0.004764	-0.010669591	0.9126	0.040871546	0.673607
UBE2Z	0.231247818	0.004764	0.111756658	0.17087	0.076490781	0.348548

TD-3148I10.	1.735252728	0.004771	1.421507449	0.02146	1.39424941	0.023914
AIFM1	-0.383520754	0.004782	-0.23447934	0.08207	0.039406455	0.769144
SNX5	-0.296429592	0.004787	-0.186674602	0.07456	-0.22718661	0.029875
CA5A	0.717149799	0.00479	0.502232124	0.04769	0.29562773	0.245145
REL	0.480962651	0.004796	0.218586197	0.20006	0.424101347	0.012814
LGALS9C	1.614037936	0.004799	1.188724785	0.03851	0.534822403	0.357511
GAS7	0.610314923	0.004826	0.258910707	0.23327	0.579184968	0.007466
PARK7	-0.308971345	0.004825	0.013506661	0.90108	-0.035174377	0.746164
PVRL2	0.333718527	0.004827	0.111943186	0.3446	0.231667731	0.050138
ZNF263	-0.283293796	0.004825	-0.183225973	0.06497	-0.225911677	0.022642
SLC38A2	0.362337271	0.004836	0.124008315	0.33481	0.406851337	0.001545
THUMPD1	-0.259795298	0.004836	-0.092959678	0.30949	-0.021742274	0.81169
SEMA6B	1.109949058	0.004844	0.983093186	0.01233	-0.227223069	0.569858
LINC00493	-0.304050889	0.004864	-0.29693765	0.00536	-0.232439143	0.02825
ZNF71	0.585698875	0.004865	0.202024413	0.33029	0.540883234	0.009039
RANBP6	-0.302467414	0.004875	-0.146447902	0.16882	-0.044585538	0.674154
CCDC97	0.498378358	0.00488	0.249415119	0.16166	-0.062888807	0.726329
KIAA1009	-0.469833653	0.004883	-0.161784902	0.327	-0.0500933	0.760222
NDOR1	0.41211753	0.004884	0.29603966	0.04215	0.179232863	0.221419
UBE2T	-0.432158836	0.00489	-0.253747974	0.09091	-0.177592704	0.233118
ARMC10	-0.267777774	0.004904	-0.238892274	0.01177	-0.240482642	0.010858
LUZP1	0.38774277	0.004903	0.194956393	0.15728	0.251043926	0.067965
TTC30B	-0.463633928	0.004931	-0.179946985	0.26522	-0.353450231	0.029405
HS3ST5	-1.252779672	0.00494	0.216688288	0.60026	0.30787028	0.462045
FOXJ2	0.486456143	0.004951	-0.133803247	0.44418	-0.059115851	0.734923
DTNB	-0.585267774	0.004955	-0.366152616	0.07452	-0.688992998	0.000882
SMPD1	0.536491144	0.004958	0.436285057	0.02245	0.231166137	0.22646
DEDD	0.305712888	0.004972	0.143821109	0.18474	0.392565478	0.00027
TPST2	0.510428854	0.004972	0.413808105	0.02243	0.580974115	0.001291
STRBP	-0.349369831	0.004978	-0.185237566	0.13363	-0.111597706	0.364995
GTF2H5	-0.30228758	0.004985	-0.18334725	0.08285	-0.178148895	0.090796
RBM12B	-0.535097918	0.004988	-0.245854101	0.19597	-0.208223301	0.272538
SDHA	-0.24736735	0.004991	-0.177312666	0.04349	-0.158454368	0.070591
SELE	3.49860886	0.004982	2.264139165	0.0722	3.127814665	0.012063
TYSND1	-0.529207217	0.004989	-0.186708979	0.31285	-0.508950096	0.006391
ZBTB43	0.372962919	0.005002	0.120850255	0.36227	0.128000655	0.332579
SIM1	-1.219611019	0.005027	-0.068626443	0.87287	-1.648776669	0.000145
DIAPH2	0.388742449	0.00503	0.554095655	6.24E-05	0.67134298	1.22E-06
FKBP9L	-1.260823611	0.005039	-1.23672266	0.0059	-2.539646255	2.00E-08
MEA1	-0.270753508	0.005048	-0.077108115	0.41854	-0.039207313	0.68001
RP1-152L7.5	-0.671857657	0.005057	-0.530263146	0.02577	-0.555705778	0.019343
BATF3	1.884203058	0.005063	2.001092777	0.00271	2.272671347	0.000522
YKT6	0.189818361	0.00511	0.271836471	5.23E-05	0.233491036	0.000513
ST3GAL1	0.605917684	0.005115	0.301887211	0.16373	0.299007518	0.167184
GTPBP1	0.426501004	0.005122	-0.07862069	0.60653	0.205509551	0.177367
HDAC1	-0.213262106	0.005134	-0.251228742	0.00094	-0.186872713	0.013688
AC004797.1	-0.552250106	0.005148	0.189943716	0.32737	0.148922727	0.442484

PPP2R1A	0.239498952	0.005146	0.105760953	0.21661	0.000713764	0.993344
TXNDC12	-0.340891035	0.005145	-0.210018805	0.08386	-0.239878553	0.048011
YES1	-0.253965516	0.005145	0.102969853	0.25439	-0.098291913	0.27714
PIWIL1	-2.024337666	0.005187	-1.651116488	0.01843	-0.670769441	0.323237
C2orf69	-0.275044847	0.005194	-0.022278903	0.81601	-0.179595204	0.062176
ARL8A	0.621773911	0.005211	0.158045443	0.47875	0.484091486	0.029346
FRMD8	0.601931374	0.005212	0.516551686	0.01627	0.171843455	0.425915
ATAD5	-0.439310399	0.00525	-0.313532874	0.04488	-0.234178821	0.133166
ZDHHC23	-0.525906153	0.005249	-0.33548618	0.07039	-0.308934622	0.097293
SMUG1	-0.294951715	0.005253	-0.190595006	0.06754	-0.223602815	0.031792
LPIN1	0.40912426	0.005271	0.18145846	0.21539	0.401746045	0.006049
AHCY	-0.358918651	0.005274	-0.37487757	0.00354	-0.191832726	0.135084
F13A1	2.137788135	0.005289	0.66092855	0.39861	0.260869063	0.739863
DGKA	0.676466604	0.00532	0.180550871	0.45771	0.277555592	0.253874
LIAS	-0.460639769	0.00533	-0.244749394	0.13165	-0.552911053	0.000678
ATP7A	-0.318684482	0.005344	-0.104545965	0.35777	-0.304121814	0.007575
VWA5A	-0.436570897	0.005352	-0.280574524	0.07115	-0.677843773	1.57E-05
CCDC15	-0.436367714	0.005369	-0.167153209	0.26338	-0.256837339	0.085186
MORN2	-0.483792902	0.005368	-0.013518836	0.93503	-0.168757085	0.31013
TBC1D9	-0.411555847	0.005368	-0.054631275	0.71012	-0.332380516	0.024036
CSPG4	0.764668255	0.005378	0.353592813	0.19893	-0.060805018	0.825844
FAM169A	-0.471661612	0.005403	-0.331024133	0.04999	-0.030299714	0.857052
HECTD3	0.401324157	0.0054	0.202960264	0.15934	0.16966484	0.240007
NCAPG2	-0.48055375	0.005401	-0.103376246	0.54624	-0.180356556	0.292434
SLC16A2	0.7930792	0.005394	0.424721752	0.13674	0.424615305	0.136803
SPTSSA	0.327287685	0.005398	0.40834979	0.00048	0.607074468	1.80E-07
PPFIA4	0.608356089	0.005412	0.198252632	0.36537	0.156821302	0.473815
PTGER4	1.104729277	0.005411	0.421141307	0.28886	1.25241342	0.001507
CAPN2	0.411346551	0.005422	0.306975734	0.03785	0.099577641	0.50083
RINL	-0.593154164	0.00542	-0.169620449	0.41527	-0.545282896	0.010104
CCNE2	-0.702366312	0.00544	-0.053778714	0.82826	0.032916633	0.894164
CCNT1	0.212334199	0.005439	0.074294991	0.32789	0.261683015	0.000519
COL6A6	1.461785699	0.005439	0.586797336	0.26572	0.87063864	0.098324
BOLA3	-0.459086939	0.005454	-0.136903061	0.39378	-0.246475967	0.126097
MYOCD	1.05025852	0.005454	-0.682471312	0.07305	0.659277335	0.081045
C10orf10	0.745524095	0.005465	0.454347123	0.09088	0.741437752	0.005601
EPHA1	-0.460628065	0.005474	-0.171306324	0.29697	-0.461004441	0.005316
HNMT	-0.267743812	0.005473	-0.065387838	0.49285	-0.123495642	0.194919
RPA2	-0.411401907	0.005492	-0.149158972	0.30823	-0.182701497	0.21197
ATP6V0E2	-0.588110888	0.005503	-0.356282979	0.08373	-0.476546934	0.020923
FAM160A2	0.455326565	0.005518	0.160886288	0.32688	0.16118838	0.325184
LGALS8	0.432743865	0.00552	0.526191721	0.00073	0.487091815	0.001753
ART3	-1.137498376	0.005527	-0.956591019	0.01641	-0.568890195	0.144421
FAM168B	0.335014985	0.005531	0.040310696	0.73819	0.282148289	0.019182
PM20D2	-0.356045988	0.00553	-0.339094456	0.00799	-0.177728969	0.163022
TMEM256	-0.35574886	0.005534	-0.078826211	0.5316	-0.239227153	0.058698
AFMID	-0.370937329	0.005541	-0.28391167	0.03261	-0.376678913	0.004605

DUSP19	-0.551052816	0.005546	-0.378849335	0.0524	-0.496786706	0.011986
PRDX1	-0.363861063	0.005543	-0.06482951	0.62053	0.0094005	0.942753
SMC4	-0.409322427	0.005546	-0.21002622	0.15418	-0.11300528	0.442987
TMEM159	-0.446767713	0.005547	-0.118180534	0.45604	-0.52721026	0.001003
NCOA1	0.304266401	0.005563	0.223333213	0.04179	0.274831199	0.012115
P11-598P20	0.52193575	0.005569	-0.067908174	0.72639	0.309279281	0.099946
ELOVL2	1.25332587	0.005571	0.958952572	0.0344	1.658388221	0.000205
AP1G1	0.200383118	0.005578	0.048575362	0.49991	0.219284459	0.00228
ZC3H8	-1.000964317	0.005583	-0.328597423	0.3606	-0.707472996	0.049518
SLC16A13	0.525905336	0.005606	0.222080148	0.24733	0.345437041	0.067296
RNF183	0.662840455	0.005609	0.764198272	0.00138	0.434164874	0.069817
ARID3A	0.581052113	0.00563	0.239870479	0.25297	0.268970327	0.199911
SAMD13	-0.623864705	0.005638	0.141286506	0.51744	-0.382064611	0.083265
MYO15A	0.728645568	0.005653	0.789467745	0.0028	0.597226775	0.02338
DES	0.905954021	0.005659	0.290963158	0.37585	0.94001282	0.004027
NAA20	-0.283194499	0.005659	0.048492771	0.62595	-0.091225387	0.361788
ADSS	-0.339618869	0.005669	-0.107690925	0.37787	-0.329352501	0.007039
DCLK2	0.816250087	0.005667	0.166918561	0.5764	0.852474538	0.003706
RPL8	-0.363941388	0.00567	-0.263280951	0.04528	-0.402300226	0.002223
XPNPEP2	1.874601085	0.005666	0.821984891	0.23047	-0.244246704	0.726913
FTL	-0.313142111	0.005683	-0.26159584	0.02081	-0.410535229	0.000287
FGF23	0.781360673	0.0057	0.599504816	0.0339	-0.468300335	0.110592
ITIH2	-0.719663379	0.005702	0.007737707	0.97627	-0.442811128	0.088817
PTPRJ	0.612820001	0.005732	0.784923308	0.00038	0.704619113	0.0014
TBC1D20	0.204314843	0.005737	-0.015201991	0.83725	0.067425488	0.358715
CSRP1	0.491728415	0.005745	0.156838979	0.37852	0.340254222	0.055914
FGF2	0.981086077	0.00575	0.690271236	0.05204	0.622896519	0.079612
SRSF1	-0.322665512	0.00575	-0.123475464	0.29023	0.035001308	0.764212
HS3ST1	0.707874297	0.005753	0.7078137	0.00551	0.47867727	0.060988
IRAK1	-0.522203085	0.005773	-0.299727125	0.11156	-0.629855609	0.000845
LAMA1	-0.860416145	0.005774	-0.471830942	0.12949	-0.223623201	0.471973
NEK3	-2.507106349	0.005775	-0.934472288	0.2757	-0.943036983	0.269296
YBX3	0.392345713	0.00578	0.505299585	0.00037	0.425772229	0.002697
LAPTM4B	1.003852317	0.005784	0.574817181	0.1147	0.853264872	0.018967
TYK2	0.310655396	0.005798	0.220176994	0.04991	0.14009965	0.212517
IDS	0.410583066	0.005805	0.381872098	0.01029	0.100452307	0.500513
DUSP5	0.902952457	0.005822	0.046224712	0.88789	1.598227011	1.01E-06
ZNF90	-0.50774206	0.005831	-0.379255747	0.0361	-0.223240592	0.213526
ZMYM4	0.32729417	0.005841	0.298552125	0.0118	0.43810183	0.000217
HS3ST3A1	1.822374954	0.005851	0.301515356	0.65376	0.052350468	0.938104
CCDC104	-0.369918616	0.005864	-0.100610829	0.4478	-0.023510095	0.858915
PTCH2	-0.847720314	0.005863	0.908447661	0.00293	-0.012075622	0.968534
YIPF4	-0.381949773	0.005865	-0.010601919	0.93879	-0.173464937	0.209201
CCT4	-0.284039408	0.005872	0.008645365	0.93297	0.002787756	0.978355
SLC25A15	-0.391751775	0.00587	-0.292547674	0.03602	-0.367088414	0.008338
NOP56	-0.349995742	0.005878	-0.168798626	0.18247	-0.053702426	0.671172
SMARCAD1	-0.214828033	0.00588	-0.119453686	0.12292	0.07854225	0.307582

MT-ND2	0.825895919	0.005884	0.568723229	0.05791	0.462255761	0.123257
CABLES2	0.386171201	0.005901	0.071017589	0.61465	0.119685287	0.392487
TOMM20	-0.281328432	0.005925	-0.209126618	0.04048	-0.125130681	0.219993
MASTL	-0.367664215	0.005928	-0.196899141	0.13753	-0.191579897	0.14802
B4GALNT4	0.571909107	0.00594	0.211508491	0.30951	0.547133094	0.008421
POLE4	-0.506403462	0.005945	-0.336562449	0.06075	-0.338756854	0.058131
PPP1R13L	0.651637792	0.005941	0.221747417	0.35068	0.304896297	0.202013
TFCP2L1	-0.911210207	0.005943	-0.614618518	0.06271	-1.507668123	5.64E-06
TACR3	-1.040475281	0.005954	-0.645843687	0.08691	0.009779223	0.979275
LZTS3	0.444103253	0.005964	0.173711637	0.28353	0.4314459	0.007249
HEYL	1.062744656	0.005986	0.827895886	0.0324	0.175814622	0.653766
HOXB9	2.118206863	0.00599	0.25578474	0.74395	0.916155004	0.237828
MARK4	0.540373125	0.005989	0.177405246	0.36962	0.142889001	0.468789
TMEM258	-0.35648995	0.005988	-0.125327128	0.3301	-0.254753321	0.047805
CBWD1	-0.507349497	0.006008	-0.060863996	0.74056	0.161865436	0.377681
GUCY1B3	0.497034165	0.00603	0.233369139	0.19752	0.721917099	6.13E-05
KIAA0355	0.39735458	0.006048	0.134320571	0.35257	0.328284765	0.022671
TMEM219	-0.312068974	0.006052	-0.16166033	0.15015	-0.236852686	0.035324
TMEM52	-1.201040978	0.00606	-1.251187364	0.00468	-1.177552484	0.005859
CCL11	1.739128006	0.006072	1.165922486	0.06888	0.818469565	0.206239
GPR176	0.895580004	0.006089	0.83275212	0.01048	0.894831461	0.005974
RANBP1	-0.327248901	0.006106	-0.119705445	0.31351	0.006065372	0.9592
RPAIN	-0.25293634	0.006113	-0.306515294	0.00085	-0.090236327	0.320191
TMEM11	0.373323874	0.006115	0.068090216	0.61814	0.234350652	0.082562
CTSZ	-0.471549182	0.006126	-0.313488309	0.06741	-0.491060098	0.004215
BCL2L14	-0.554340061	0.006148	-0.227746557	0.25275	-0.240386476	0.227615
COTL1	0.553633145	0.006141	0.429103402	0.03364	0.366525741	0.069598
PRKX	-0.432755464	0.00615	-0.52563707	0.00086	-0.354735124	0.024424
SIRPA	0.62767761	0.006147	0.17247871	0.45439	0.563825673	0.013508
SPAG16	-0.357774614	0.006142	-0.100049012	0.4365	-0.086268058	0.500152
PHF3	-0.247364969	0.006164	-0.165634981	0.06618	-0.122163204	0.174809
EEF1A1P13	-0.681541882	0.006171	-0.02333975	0.92275	-0.308196399	0.203767
LENG8	1.100199543	0.006174	1.000851639	0.01236	0.680347714	0.092511
VNN2	1.175507904	0.006185	1.435352581	0.00069	1.274668457	0.002741
NUP43	-0.292247363	0.006197	-0.100369437	0.34285	-0.119429127	0.258697
AGFG1	0.330999117	0.006202	0.176411854	0.14426	0.343843961	0.004388
ZNRF3	-0.463185702	0.006205	-0.386856319	0.02113	-0.492266603	0.003364
CCDC141	-1.34095871	0.006241	-1.102504968	0.02365	0.017929151	0.970258
GPM6B	0.634466754	0.00624	0.267490768	0.24919	0.426755797	0.064182
SH2D3C	1.947522956	0.006242	1.69600755	0.01447	1.344793044	0.057353
RSL1D1	-0.356486521	0.006248	-0.228172341	0.07967	-0.183829616	0.15774
SLC29A1	-0.538556051	0.006246	-0.452229939	0.02131	-0.706624213	0.00033
DACT3	0.569948112	0.006253	-0.148669119	0.47889	0.325737468	0.118234
SLC9C2	-1.631359034	0.006262	-1.252010225	0.03445	-2.650497311	1.40E-05
C1orf74	-0.479602833	0.006272	-0.113315364	0.50482	-0.51775741	0.0028
ZNF730	-0.411475762	0.006273	-0.153745788	0.29559	0.036605606	0.800683
CYR61	0.851089069	0.006376	0.753039765	0.01572	0.944591424	0.002353

LRRC37A15P	-1.144276502	0.006384	-0.63823057	0.10412	-0.446869907	0.245926
PRL	3.353057574	0.006417	2.45945356	0.04594	3.416745331	0.005455
GLMN	-0.506726102	0.006435	-0.04205985	0.81747	-0.233050845	0.202016
COL23A1	0.936822097	0.006443	0.221457214	0.53323	0.131777784	0.70919
LMNB1	-0.448356702	0.006438	-0.304116318	0.06409	-0.227934975	0.164844
TMEM132A	0.605941741	0.006443	0.168305888	0.4498	0.218505998	0.326156
CHRNA7	1.571359047	0.00645	0.362140878	0.53462	0.85378733	0.138799
PNPLA2	0.710571549	0.006451	0.372344429	0.15737	0.376604098	0.150457
SOWAHA	-0.755096553	0.00647	-0.419186542	0.12255	-0.732697029	0.007524
DCUN1D3	0.38888402	0.006486	0.426749597	0.00271	0.541828542	0.000133
ECE1	0.655377787	0.006491	0.716942941	0.00289	0.086519808	0.71955
STAG3L4	0.457135621	0.00649	-0.078767736	0.63711	0.256054055	0.117028
ZNF700	-0.446368998	0.006501	-0.245775386	0.12962	-0.243889703	0.132105
ENTPD5	-0.412733577	0.006523	-0.329259248	0.02979	-0.624512962	3.85E-05
FANCC	-0.549977399	0.006524	-0.510936458	0.01095	-0.466051615	0.020011
RNF186	1.817753425	0.00653	1.339777871	0.04519	0.218028157	0.745833
HEATR1	-0.347207486	0.006537	-0.331745061	0.0092	-0.050031646	0.694339
ITGB6	0.772361151	0.00654	1.610232298	1.36E-08	0.781436304	0.005907
FKBP15	0.303297362	0.006547	0.025381309	0.81955	0.0362865	0.74393
KIAA0100	0.287858262	0.006563	0.22977884	0.02984	0.175515611	0.097085
MRPL52	-0.369266004	0.006576	-0.080796805	0.54664	-0.385908872	0.004163
NT5DC1	-0.538154662	0.006575	-0.076049047	0.69945	-0.215572642	0.27398
ARID2	0.261557058	0.006583	0.101558039	0.29147	0.287373427	0.002728
PET117	-0.563558762	0.006594	-0.345766955	0.08805	-0.526722154	0.009843
PRPS1	-0.380754312	0.006604	-0.344565768	0.01363	-0.358187452	0.010296
PLEKHM2	0.36916583	0.006607	0.152998528	0.26032	0.257980813	0.057047
GUSBP1	-0.484274469	0.006626	-0.308102077	0.08022	-0.261654999	0.136034
LIMK2	-0.312828959	0.006638	-0.111679555	0.32909	-0.28784907	0.012105
NDUFB9	-0.276275787	0.006642	0.000954376	0.99242	-0.156843802	0.119123
ZNF609	0.278218312	0.006644	0.026079849	0.7993	0.085098327	0.405717
FGF5	0.668646689	0.006652	0.603396116	0.01409	0.5292624	0.031378
SERPINI1	-1.016892745	0.006654	-0.377536484	0.29669	-0.469022946	0.204706
SUN2	0.403897784	0.006654	0.125677613	0.39737	0.174551279	0.239829
FAM127B	0.421246288	0.006694	-0.224737404	0.15916	-0.077048176	0.624499
HIST1H2AI	-0.570983089	0.0067	-0.305284868	0.1464	-0.332349773	0.113823
ELK3	0.521280304	0.00671	0.428439035	0.0256	0.189876327	0.324062
ZDHHC2	-0.633041881	0.006721	-0.027637787	0.90552	-0.153046085	0.51142
LSM5	-0.359307817	0.006731	-0.129305167	0.32619	-0.179515239	0.172044
IRF1	0.5400727	0.006742	0.300356601	0.13216	0.46629561	0.018967
MAP9	-0.344601179	0.006746	-0.28382044	0.02455	-0.173986862	0.167969
MNAT1	-0.312124143	0.006746	-0.103158362	0.36207	-0.09081032	0.421031
SYVN1	0.52718516	0.006747	0.288524346	0.1394	0.152722238	0.433845
UACA	-0.40015586	0.006748	-0.094090807	0.52264	-0.389686215	0.008225
LRRC31	-0.91937486	0.006769	-0.526544814	0.11142	-1.328025835	9.37E-05
F816-ZNF32	-0.582405337	0.006775	-0.236769213	0.2624	0.038654013	0.854228
TD-3088G3.	1.114316036	0.006782	0.697295265	0.09228	0.570147786	0.168431
GIPC2	-0.809885594	0.006782	-0.478075215	0.10618	-0.766329588	0.010167

C19orf57	-0.91631917	0.006801	-0.468801938	0.15536	-0.61147469	0.064099
THSD7B	1.881429721	0.006808	0.870658238	0.21318	1.98620511	0.004016
AC024592.1[illegible]	3.442894069	0.006822	-0.70385989	0.58426	3.536463484	0.005468
ANXA2	0.294905352	0.00682	0.43756184	5.93E-05	0.324949554	0.002864
SRSF5	-0.304285335	0.006824	-0.070574731	0.5297	0.092577296	0.408874
CA13	-0.431675616	0.006836	-0.585189378	0.00025	-0.463846703	0.003497
ELFN1	1.025118958	0.006841	0.002101984	0.99574	0.65772848	0.084105
NDFIP2	0.45603034	0.006842	0.250255099	0.13721	0.590106727	0.000436
EME1	-0.508163583	0.006862	-0.243584953	0.17969	0.061958534	0.731654
TJP3	0.644330589	0.006873	0.591447253	0.01307	0.563314621	0.018115
DGKB	1.423064015	0.006896	0.09833016	0.85371	0.72030036	0.173351
MGST2	-0.556000782	0.006924	-0.326334482	0.11158	-0.24214133	0.237548
APEH	-0.344095308	0.006928	-0.230400447	0.06972	-0.191926496	0.130558
DAAM2	0.80113555	0.006937	0.869295675	0.00335	0.704312961	0.017569
AMZ2P1	-0.58894733	0.006942	-0.323740291	0.13095	-0.391741259	0.06852
UBALD1	0.604742894	0.006951	0.12741668	0.58055	0.229321272	0.31329
SRRM1	0.232466711	0.006991	-0.215302762	0.01318	0.094011651	0.274615
FAM177B	-0.958092416	0.007009	-0.651977383	0.06496	-1.228180042	0.000548
RPL41	-0.255681413	0.007009	-0.21021528	0.02657	-0.300112779	0.001545
GPR183	0.842887908	0.007017	-0.080082045	0.80307	1.289095972	2.81E-05
MAST2	0.467156903	0.00702	0.235808672	0.17482	0.507987404	0.003376
C2CD4A	-1.039606296	0.007034	-0.24170328	0.52582	-0.45859479	0.229445
MTF2	-0.302226	0.007048	-0.129454861	0.24606	0.132531607	0.233309
LRRC41	0.480660305	0.007058	0.237082405	0.18402	0.279148123	0.116381
C19orf48	-0.370069999	0.007112	-0.315221031	0.02123	-0.412784473	0.002594
RPL27A	-0.30752201	0.007115	-0.081727997	0.47414	-0.154150329	0.177023
ECHDC1	-0.269997319	0.007123	-0.098567096	0.3205	-0.156506235	0.115652
BAG3	0.33645947	0.007129	0.073402336	0.5576	0.127267183	0.307446
CCND1	-0.494570179	0.00713	-0.534320757	0.00362	-0.574215061	0.001775
CORO7	0.44205756	0.007135	0.050047246	0.76105	-0.069880594	0.671377
SLC25A26	-0.37702818	0.007158	-0.109186322	0.4322	-0.139950807	0.311757
ITPKA	-0.716989713	0.007166	-0.763669083	0.00384	-1.204904855	1.03E-05
ENY2	-0.31244876	0.007179	-0.003038391	0.97903	-0.098934224	0.390497
CCDC93	0.44724803	0.007187	0.130275942	0.43242	0.295565948	0.075114
SEC61G	-0.312018957	0.007186	0.049376319	0.66725	0.132074312	0.249097
EPS8L3	0.489262997	0.007204	0.42186051	0.02049	0.607427516	0.000836
RPS29	-0.389406475	0.00721	0.088670614	0.53975	-0.084401398	0.55953
PILRB	0.432530168	0.007222	0.330345646	0.03877	0.569686345	0.000334
KIAA1191	0.352618154	0.007231	-0.031924532	0.80822	-0.054925443	0.676058
LIPA	-0.343441703	0.007228	-0.130658118	0.30295	-0.319339827	0.012144
MT-CO1	0.526281858	0.00726	0.079738699	0.68418	0.096464504	0.622661
DHDDS	0.289577648	0.007267	0.206241144	0.05498	0.173773552	0.1049
[illegible]HD1-EIF4E	0.721748092	0.007273	0.408434094	0.129	0.446319515	0.097071
CMTM1	0.954432924	0.007287	0.109316533	0.76668	0.29895902	0.410261
ESRRA	0.355662541	0.007294	0.432227854	0.00098	0.236221576	0.07411
RPL39L	-0.515312373	0.007289	0.00406566	0.98234	-0.303089008	0.102939
THY1	0.614514223	0.007292	-0.066454274	0.77211	0.274597087	0.230665

TMEM106A	1.375811867	0.007288	0.39515484	0.45821	1.185974839	0.020883
SSR4	-0.359403833	0.0073	-0.126572088	0.34317	-0.470896529	0.000433
QDPR	-0.545180846	0.007308	-0.27862701	0.15917	-0.405329682	0.040659
SLC30A7	0.273456329	0.007306	0.361188031	0.00038	0.200985566	0.048121
CCDC90B	-0.229841425	0.007317	0.025381605	0.76418	-0.073288571	0.38678
RPL10A	-0.300169241	0.007316	-0.146643379	0.18973	-0.170670555	0.126932
DPH6	-0.605231754	0.007322	-0.333669107	0.13523	-0.591560671	0.008075
SEPHS2	-0.376868947	0.007325	0.274604707	0.0468	-0.093668559	0.500032
CSRNP2	0.367370848	0.00735	0.219059688	0.10954	0.365059183	0.007508
ARID5B	0.355888455	0.007355	-0.126571171	0.34121	0.258177108	0.051454
MMP3	1.735383793	0.007362	0.784967903	0.22901	-0.120145402	0.85534
RBM44	-1.141168417	0.007362	-1.146385809	0.00601	-1.088632959	0.008698
SLC41A1	0.521303455	0.007366	-0.051735547	0.7909	0.245525596	0.20771
CRTAP	-0.333106631	0.007378	-0.394030354	0.00149	-0.461930601	0.000198
MAPK8IP3	0.563880621	0.007378	-0.017037151	0.93565	0.432679795	0.039583
PPP1R11	0.310455343	0.007378	0.227659911	0.04896	0.19724797	0.087845
SLC27A2	-0.864019868	0.00738	-0.558158555	0.08117	-1.241983459	0.00012
DGKD	-0.611193918	0.007406	-0.270944905	0.23269	-0.676115746	0.003209
MPHOSPH6	-0.341368045	0.007402	-0.372111815	0.00317	-0.111539294	0.371972
SYNM	0.663266769	0.007407	-0.448970264	0.07442	0.680626128	0.005877
NUF2	-0.493224943	0.00742	-0.030394188	0.86632	0.071270987	0.692319
RP11-889L3.	-0.574779025	0.007423	-0.554671355	0.00879	-0.319013806	0.124739
GBP3	0.485629746	0.007428	0.667861576	0.00022	0.608763052	0.000773
TGIF2	0.75144646	0.00743	0.383693193	0.17029	0.675226656	0.015535
ALDH6A1	-0.4123273	0.007435	-0.290097056	0.05853	-0.241421947	0.115221
MICB	1.117090356	0.00745	0.852032348	0.04322	0.56426788	0.185963
RP11-777B9.	-2.516168117	0.007468	-0.58682045	0.53159	-1.127875048	0.229371
LRRC6	-0.932885665	0.007476	-0.681796974	0.04687	-0.932677384	0.007611
PPP1R14B	-0.323003425	0.00748	0.027173021	0.81994	-0.203521122	0.089292
CCDC92	0.679680634	0.007485	0.448789966	0.07812	0.241321126	0.344581
ATPAF1	-0.406143049	0.007498	-0.34882007	0.02101	-0.134440755	0.371842
TRIM9	1.070880221	0.007506	0.764231651	0.05565	0.573777649	0.152713
TICAM1	0.924773169	0.007522	0.134363897	0.70219	0.144325139	0.680705
CRTC3	0.519645595	0.007568	-0.156717524	0.42464	0.389325448	0.045842
DOPEY2	-0.55332118	0.007566	-0.625658076	0.00248	-0.544209917	0.008535
EVI5	-0.361798652	0.007598	-0.068061977	0.61453	-0.044129347	0.74412
ABHD17A	0.546062758	0.007636	0.077138476	0.70701	-0.0715017	0.727595
MTHFD1	-0.362912617	0.007639	-0.251958947	0.06276	-0.279822602	0.038682
PTGER2	-0.701203416	0.007635	-0.162145848	0.53137	-0.655757764	0.012078
DENND4C	-0.325576131	0.007643	-0.294748208	0.0156	-0.06508105	0.593064
PEX6	-0.328451228	0.007648	-0.203851628	0.09442	-0.414576561	0.000713
PRDM6	1.407225725	0.007664	0.994099863	0.06097	1.013796243	0.054912
TTPA	-0.897197264	0.007668	-0.679831153	0.03679	-1.033951221	0.001749
BBS10	-0.353421142	0.007677	-0.156455478	0.2302	-0.084304288	0.515948
LMBRD2	-0.609628953	0.007684	-0.178724114	0.43324	-0.015430715	0.946091
CYTH2	0.367964922	0.007692	0.155032122	0.26174	0.267400795	0.052408
TGFBR2	-0.320607314	0.007705	-0.457809026	0.00014	-0.409102369	0.000663

TPCN1	0.43583339	0.007712	-0.26456107	0.10734	-0.027809347	0.865218
LTBP4	0.561499536	0.007716	0.023793899	0.9105	-0.017283375	0.934912
HOXC8	1.086120889	0.007764	0.191625547	0.64395	0.0936047	0.821607
DCK	-0.521228084	0.007801	-0.381209605	0.04954	-0.146643001	0.449001
HS6ST1	0.338658382	0.007803	-0.092838638	0.46738	0.443098182	0.00047
ZNF449	-0.375720749	0.007834	-0.226341682	0.10344	0.017251383	0.900084
GNG4	0.833323937	0.007866	0.203050444	0.51944	0.292727598	0.352279
PSMA6	0.278637505	0.007864	0.300480865	0.00404	0.347982257	0.000867
FAM13A	-0.654023082	0.007876	-0.79446647	0.00124	-0.730825674	0.002979
LDLR	0.300949288	0.007874	0.00044625	0.99686	0.200310152	0.07682
SMAD1	0.574993677	0.007871	0.403213356	0.06247	0.652609446	0.002501
EHD1	0.649012734	0.007957	0.338521262	0.16605	0.569637652	0.019214
IFNAR2	0.543689253	0.007953	0.652266681	0.00141	0.416253726	0.041786
INCA1	0.896620391	0.007962	0.186371585	0.59583	0.861719781	0.009128
TAB3	0.33765963	0.007959	-0.144209608	0.25854	0.096727177	0.446371
DNAH5	0.684751434	0.007968	0.585682359	0.02282	0.624728378	0.015197
FAM208B	-0.266059558	0.00797	-0.060792092	0.54323	-0.196525253	0.049028
TBC1D25	0.547795625	0.007976	0.015604618	0.93994	0.244793769	0.23471
CECR2	1.388909252	0.007993	0.55223099	0.29968	0.802142446	0.127982
LYAR	-0.515562613	0.007995	-0.221726681	0.24953	-0.161666449	0.400286
UQCC2	-0.414107977	0.008046	-0.173095517	0.26341	-0.303724199	0.04978
NDUFS4	-0.304862394	0.00805	-0.093334858	0.41094	-0.175211042	0.122876
IFT122	-0.438117936	0.008056	-0.166121912	0.31091	-0.538439427	0.001087
LRRN2	1.222671189	0.008068	-0.136455167	0.77185	0.191031619	0.684599
TMEM92	0.602806089	0.008069	0.183963605	0.41929	0.200411186	0.379402
CARD9	1.116529168	0.008073	0.742069844	0.07774	1.044535427	0.012615
C9orf142	-0.524340493	0.008079	-0.181898951	0.34997	-0.363986368	0.063026
TTC40	1.147338207	0.008143	1.379185427	0.00126	0.990542774	0.020885
GLB1L2	-0.512801803	0.008151	-0.56164551	0.00371	-0.590571653	0.002274
TXNIP	-0.541735694	0.008149	-0.524163567	0.01045	-0.751792201	0.000241
POLE3	-0.369329158	0.008172	-0.23735822	0.08729	-0.091155578	0.509958
PTP4A2	0.309897807	0.008169	0.374411054	0.00139	0.430419878	0.000236
CDC42EP1	0.518731495	0.008183	0.284537584	0.147	0.138934811	0.479121
CAB39L	-0.462669872	0.008187	-0.176968841	0.30667	-0.443620365	0.01076
RQCD1	0.243418349	0.008193	0.132873116	0.14852	0.325317707	0.000373
TCAIM	-0.303313673	0.008222	-0.06470264	0.5681	-0.135306107	0.233025
EMB	-0.714013893	0.008232	-0.289492138	0.28074	-0.322812907	0.228796
LIN9	-0.418347056	0.008232	-0.323191074	0.03843	-0.151685394	0.327718
ZNF33B	-0.654647658	0.008227	-0.158773306	0.51592	-0.449382625	0.066126
KIAA0020	-0.274829658	0.008248	-0.225105175	0.02946	0.019935024	0.84634
GARS	0.385610906	0.008259	0.208184833	0.1536	0.396871314	0.006476
C2orf61	-0.699726412	0.008271	-0.059698377	0.80771	-0.320221328	0.197884
WWTR1	0.557540365	0.008331	0.600215041	0.00445	0.173233838	0.412533
THNSL1	-0.403188673	0.008338	-0.15857116	0.28625	-0.37896289	0.011587
FAM115A	-0.19479382	0.008356	-0.021390043	0.77077	-0.133516714	0.069034
C8orf37	-0.41889394	0.008377	-0.041857216	0.78231	-0.337900599	0.028562
NUTF2	0.2765645	0.008374	0.278374953	0.00785	0.401276316	0.000124

WBP5	-0.249801414	0.008373	0.071103383	0.44733	0.044903451	0.631226
TRIM40	2.56866474	0.008385	0.873729109	0.38477	3.276808835	0.000695
PDHA1	-0.231871975	0.008402	-0.142878968	0.10211	-0.100238863	0.249876
UBE3D	-0.700147816	0.008406	-0.46305189	0.07853	-0.557924184	0.033722
HTR1B	-1.190183831	0.00847	-0.413520409	0.35597	-1.784919744	8.56E-05
CPSF3L	0.333222655	0.008516	0.071153825	0.57438	0.203807708	0.107962
RPL5	-0.350382682	0.008518	-0.15473397	0.24518	-0.080824454	0.543802
TEX10	-0.324523513	0.008523	-0.306198889	0.01268	-0.00956448	0.937624
BCCIP	-0.294124139	0.008537	-0.15382868	0.16626	-0.055981834	0.613092
SCN8A	-1.133602475	0.00859	-0.081586978	0.84778	-0.632408426	0.137371
CDRT1	1.641315171	0.008604	1.059880261	0.08818	1.217884012	0.049145
MLPH	-0.701290672	0.00862	-0.461964156	0.08247	-0.976643424	0.000255
PTCD2	-0.459138115	0.008638	-0.487177446	0.00509	-0.504022052	0.003633
CSTF2T	-0.258266029	0.008688	-0.143093597	0.14211	-0.101820911	0.294919
YAE1D1	-0.347124566	0.008717	-0.173152538	0.18245	0.034068876	0.790857
NFKBIE	0.776686628	0.008739	0.340287911	0.25681	0.495555387	0.096128
FAM169B	2.118830301	0.008803	1.882853506	0.02039	2.42397586	0.002135
PDZD8	0.531694685	0.008805	0.431226534	0.03342	0.449224979	0.026701
RAB5C	0.247594422	0.008828	0.264787109	0.00498	0.126910859	0.178815
CD274	1.87534467	0.008832	1.302387127	0.07224	1.947260531	0.005953
MRPL15	-0.319664027	0.008852	-0.098180802	0.41197	-0.117626926	0.325515
ENOSF1	-0.373290555	0.008867	-0.246868574	0.08193	-0.285521107	0.044071
STK10	0.553232174	0.008869	0.279246416	0.18571	0.476195561	0.02408
TNIP3	1.094525975	0.00891	1.464947791	0.00041	0.896558416	0.031954
RBMX	-0.204108025	0.008916	-0.01589151	0.83831	0.057550025	0.459477
STX10	-0.34180429	0.008931	-0.278416397	0.03128	-0.133919104	0.299058
RALY	-0.228989945	0.008939	0.013696025	0.87518	0.000963997	0.991159
SCPEP1	-0.353047106	0.00894	-0.205846444	0.12061	-0.051021888	0.700982
PPID	-0.386552272	0.008964	-0.054594143	0.71004	-0.004777435	0.974005
NDNF	1.094215541	0.008972	0.375286018	0.37399	-0.743067486	0.088107
NTN1	0.908017372	0.008975	0.711266099	0.04073	0.327277784	0.3472
BMS1	-0.223188025	0.00901	-0.037178883	0.66114	-0.035706709	0.67349
MARS	0.331454297	0.009013	0.349369204	0.00588	0.331364492	0.008988
AC010620.1	-0.347777354	0.009046	-0.402526428	0.00245	-0.230010774	0.082072
CNPY2	-0.344330044	0.009032	-0.087930461	0.50248	-0.204445326	0.11937
DPH5	-0.338829769	0.00904	-0.189572198	0.13667	-0.017057162	0.892819
PRKACB	-0.548822328	0.009049	0.105385611	0.61419	-0.515843037	0.014027
SH2D3A	0.414773029	0.009048	0.05229002	0.74292	0.504138364	0.001438
SPIN2B	-0.475743852	0.009044	-0.353430615	0.04133	-0.067678511	0.691897
B3GALT5	1.003257688	0.009056	0.396405828	0.30362	-0.299686629	0.437984
CD3G	-1.040925092	0.009076	-0.341369227	0.38879	-1.028205401	0.00974
ERLEC1	-0.277811429	0.009081	-0.068168995	0.51906	-0.1300678	0.218835
IGF1R	0.542543044	0.009078	-0.077413529	0.70992	0.169025675	0.416509
FKBP9	-0.254481806	0.009102	-0.400619036	4.02E-05	-0.395655756	4.92E-05
PDK4	-1.182297192	0.009099	-0.40411121	0.36278	-0.898352561	0.045551
ELFN2	1.710335941	0.009114	0.2016086	0.7644	0.222971873	0.741895
NCOR2	0.595992839	0.009113	0.315902609	0.16752	-0.07137816	0.755814

DKKL1	1.093179008	0.009118	-0.211725941	0.63694	0.424718981	0.322138
PARP12	0.533328172	0.009133	0.65303645	0.00131	0.47474479	0.020082
IGSF3	0.408028791	0.009138	0.292190875	0.06132	0.040481816	0.79583
TCF20	0.211989787	0.009142	0.101399581	0.21024	0.152802875	0.058035
CMPK2	-0.66022944	0.009149	-0.238140659	0.322	-0.472332175	0.053347
ZDHHC21	-0.406979558	0.009214	-0.275301986	0.07712	-0.429749045	0.005832
EIF1AXP1	-0.83362232	0.009236	0.029829228	0.92389	-0.136852946	0.661808
CWC22	-0.22544728	0.009255	-0.118472022	0.1656	0.054435369	0.520077
FABP2	1.86357825	0.009251	0.696272189	0.33344	0.905378686	0.2077
MYO7B	1.667478047	0.009253	0.848102899	0.18641	0.65466124	0.308487
TDO2	1.553114921	0.009249	1.238779675	0.03792	1.231580097	0.039044
EFCC1	0.772856352	0.009271	-0.337182003	0.27942	0.429707818	0.150523
PARPBP	-0.428623187	0.009279	-0.266218574	0.10136	-0.017762599	0.912365
KLHL17	0.712634123	0.009293	0.193635704	0.48467	0.174247153	0.532224
ZNF677	0.370422447	0.009291	0.082835428	0.56221	0.31221584	0.027683
CCDC64B	0.505229069	0.009301	0.116111774	0.55221	-0.016900658	0.931072
METTL5	-0.300190361	0.009314	-0.00686486	0.95158	-0.13791171	0.223726
GSDMC	1.669829581	0.009328	0.762293132	0.25324	0.766272308	0.246015
ARIH2	0.218526782	0.009343	0.075389116	0.36804	0.183952788	0.027568
EDEM3	-0.38014938	0.009338	-0.158119708	0.27898	-0.33146374	0.023311
IKBKB	0.408979332	0.009334	0.011031658	0.94409	0.254470367	0.104397
MTR	-0.246972197	0.009344	-0.10616264	0.26257	-0.16169754	0.087689
MARK2	0.322143571	0.009361	0.03252086	0.79393	-0.01637387	0.895207
GRHL2	-0.409326001	0.009398	-0.195417463	0.2139	-0.301984242	0.054559
POLR3K	-0.521906432	0.009399	-0.244534202	0.21087	-0.357670358	0.06966
?P13-128O4.	1.051134532	0.009406	0.477390163	0.25163	0.563486593	0.171089
ZNF180	-0.348513622	0.009403	-0.048868251	0.7108	0.015422981	0.906077
SEPW1	-0.253900862	0.009418	-0.184566672	0.05806	-0.278834917	0.004208
PTHLH	1.84070771	0.009435	2.63459971	0.00019	0.847679861	0.234675
TAF7	-0.233123024	0.009433	-0.049305201	0.57953	0.047322177	0.593739
S1PR2	0.70609737	0.009465	0.294296212	0.28032	0.129731909	0.634481
CCNYL2	-1.636072794	0.009471	-0.270025257	0.62908	-0.961333966	0.097778
ABCA1	0.591904932	0.009479	0.439420162	0.05406	0.354554921	0.120118
CKAP4	0.211788432	0.009492	0.263101155	0.00124	0.153549009	0.05958
XPO4	-0.277249461	0.009501	-0.122021415	0.25025	-0.114872657	0.278518
FKTN	-0.475305162	0.009514	0.020721478	0.90957	-0.337948636	0.064359
HMGB1P14	0.995629852	0.009514	0.480268751	0.22449	0.665792603	0.085215
RAD21	-0.246573898	0.00953	-0.024319797	0.79746	0.034301975	0.717077
GBGT1	0.714021371	0.00956	0.524516504	0.05594	0.063942843	0.820894
SWAP70	0.322572592	0.009574	0.487812875	7.21E-05	0.439613459	0.000347
TAS2R4	-0.891788442	0.009573	-0.796924869	0.01826	-0.504235389	0.12719
ARPC3	-0.227617898	0.009587	0.033284791	0.70334	-0.042654316	0.625648
C14orf166	-0.213003957	0.009584	-0.081328588	0.32001	0.087547136	0.283507
TYRO3	-0.354295197	0.009614	-0.730100824	9.75E-08	-0.594725818	1.37E-05
CDC45	-0.672077972	0.009621	-0.39022434	0.12963	-0.224926503	0.381221
RAB3GAP2	0.297391549	0.00964	0.359039441	0.00172	0.200718636	0.079646
FCGRT	-0.373610119	0.009663	-0.415091296	0.00402	-0.662704605	4.49E-06

RELT	0.64658968	0.00968	0.690091036	0.00553	0.669301621	0.006935
WDR93	1.265823404	0.009678	0.726390442	0.14174	0.647058608	0.189916
SIX5	0.795142844	0.009698	-0.262036996	0.40263	-0.198262957	0.524545
ATP1B2	0.994084725	0.009756	0.181836812	0.64024	-0.561945085	0.157636
BAI3	0.761925016	0.009745	0.262716471	0.37523	0.992207774	0.000695
PAX5	1.529772406	0.009753	1.617167132	0.00594	0.895858111	0.136603
PIP5K1A	0.303500003	0.009758	-0.019169967	0.87025	0.350805095	0.0027
RUSC1	0.284265925	0.00975	-0.066962584	0.54722	-0.042362436	0.701224
P11-697N18	1.201163288	0.00977	0.59990403	0.20791	0.637327433	0.178211
BHMT	-1.184871878	0.009784	-0.719020659	0.09795	-1.515876094	0.000856
CLDN9	-0.884231562	0.009782	-0.512678703	0.11252	-0.54941169	0.088151
MCCC1	-0.460152151	0.009777	-0.31655399	0.07378	-0.257452637	0.145178
SQSTM1	0.497721564	0.009788	0.370378774	0.05452	0.497448595	0.009782
STX3	-0.512795642	0.009812	-0.509195841	0.01024	-0.323193661	0.1028
ADAMTS2	1.251412722	0.009824	1.586841832	0.00101	0.828790167	0.089093
UQCR11	0.300676891	0.009835	-0.07577092	0.51772	-0.348374792	0.003074
RPL35A	-0.296207258	0.00984	-0.078124347	0.4954	-0.168029926	0.142613
ZNF592	0.227026092	0.009856	-0.069354994	0.43225	-0.086346673	0.327131
BRE	-0.341564642	0.009867	-0.223096777	0.08806	-0.185782156	0.15501
MCM10	-0.605509805	0.009864	-0.494763462	0.03432	-0.268420322	0.250095
REEP4	0.375405441	0.009874	0.191591787	0.18493	0.100655628	0.489498
CCDC17	0.86084217	0.009897	0.535416622	0.10865	0.962570769	0.003402
CPA6	-1.52838109	0.009899	-0.66119903	0.22184	-1.940263483	0.001216
OSBPL3	-0.33944308	0.009894	-0.253457365	0.05231	-0.33415277	0.010609
ST6GALNAC	0.552157998	0.009902	0.336792853	0.11508	0.249287661	0.243512
PDE7A	-0.215591366	0.009925	-0.021142084	0.79773	0.070042629	0.394067
C19orf10	-0.338938194	0.009938	-0.090970566	0.48532	-0.309910288	0.017748
STK38L	-0.353791268	0.00994	0.207620546	0.12874	-0.003382595	0.980272
TARS2	-0.307730364	0.009932	-0.091706279	0.43304	-0.168929499	0.149906
ZNF281	0.260677774	0.009954	0.093353664	0.35574	0.479289599	1.77E-06
RANBP2	-0.182752699	0.009967	-0.087788619	0.21464	0.004745984	0.94645
BDH2	-0.369056447	0.009987	-0.314639033	0.02699	-0.07680486	0.586493
COBLL1	-0.493604846	0.009983	-0.359303687	0.06028	-0.637980294	0.000859
FRS3	0.63502935	0.009994	0.147611114	0.5589	-0.117574079	0.640911
IFT74	-0.357226563	0.009993	-0.284063782	0.03834	-0.101732281	0.456056
LPL	1.891399519	0.009985	2.081197977	0.00444	2.210183983	0.002498
CD96	-1.377791089	0.01001	-0.714321894	0.17566	-2.704422398	7.95E-07
P11-1396O1	-1.808482418	0.010035	-1.184545851	0.07427	-1.35208833	0.042919
MRPL42	-0.333407257	0.010056	-0.150174999	0.24536	-0.152695814	0.23749
CACNG8	0.479860744	0.01008	0.357201633	0.05478	0.152929691	0.414331
C16orf62	-0.27166152	0.010117	-0.081586059	0.4353	-0.245644148	0.019345
DMC1	-1.002835087	0.010121	0.286653598	0.43833	-0.389439561	0.299407
LY6H	6.336879712	0.010129	4.993318124	0.04351	6.262927862	0.010998
1-Mar	-0.876436309	0.010126	-0.645892504	0.0576	-0.863237262	0.011224
MGAT3	0.361772591	0.010124	0.139050804	0.32271	0.387532689	0.005757
NTAN1	-0.42400354	0.010132	-0.010426224	0.94912	-0.035182402	0.829221
TMEM18	-0.347536039	0.010147	-0.222481874	0.0948	-0.157420273	0.236222

ALS2CL	0.643580923	0.010154	0.590158236	0.01818	0.800898242	0.001318
NPM1	-0.288930147	0.010173	-0.154654248	0.16881	-0.016758502	0.881445
LGR4	-0.309356172	0.010178	-0.168735814	0.1606	-0.030980164	0.796613
GPR137	0.55208374	0.010184	0.427661741	0.04486	0.213845031	0.318167
ST3GAL3	0.574864183	0.010208	0.360601833	0.10775	0.343827386	0.122221
AC243772.2	0.490199879	0.01024	0.137359385	0.47559	0.218160851	0.25394
PDIA6	-0.25497213	0.010244	-0.149494227	0.13191	-0.126815997	0.201145
SCAMP2	0.408806548	0.01025	0.182386321	0.25172	-0.026477234	0.868027
PPP2R5A	-0.275393653	0.010255	-0.019413255	0.85533	-0.105881005	0.320035
FBXO10	0.664635961	0.010277	0.281656166	0.27909	0.335645324	0.19523
PCBD1	-0.403241756	0.010286	-0.251831753	0.10752	-0.379388246	0.015434
CHRNA5	-0.724927597	0.010292	-1.216916364	1.98E-05	-0.489084951	0.081351
CYP7B1	1.318879042	0.010305	1.12015905	0.0292	0.786507448	0.127651
DENR	-0.180829663	0.010316	-0.041544098	0.55131	-0.064841137	0.352804
CPA3	-1.715883092	0.01036	-0.30433701	0.63451	-0.576038773	0.375426
DMTF1	0.317878865	0.010365	0.061787169	0.61797	0.269467321	0.029157
NAA16	-0.409353607	0.010378	-0.321344726	0.04295	-0.15602807	0.324951
RSRC1	-0.285432016	0.010388	-0.134734988	0.22205	-0.040598721	0.711503
ACTR10	0.293691977	0.010397	0.222972888	0.04985	0.240528405	0.034399
GSPT2	-0.304183007	0.010433	-0.057674275	0.62201	0.010187024	0.93036
INTS7	-0.4735086	0.010428	0.047583024	0.79614	-0.057525252	0.754445
KLF9	0.882510836	0.010429	0.08551963	0.80589	0.899684823	0.008811
UNGP1	1.252991582	0.010423	0.696054568	0.16668	0.617243786	0.220466
ARHGAP11A	-0.454018025	0.010438	-0.020835423	0.90607	0.025516469	0.885
MRPL20	-0.249037335	0.01045	-0.054360942	0.57071	-0.119325032	0.213439
KDM5A	0.198705028	0.010455	0.038419807	0.61915	0.148912235	0.053591
MAP4	0.414183807	0.010458	0.266324654	0.09976	0.221160008	0.171637
EBAG9	-0.374080712	0.010502	-0.193888438	0.17812	-0.243324797	0.091201
TMEM136	0.46986172	0.010553	0.517408498	0.00451	0.432532994	0.017908
DPH3	0.357017022	0.010582	0.265677787	0.05628	0.407767035	0.003282
TBC1D30	-0.538816553	0.010586	-0.518942559	0.01289	-0.588906413	0.004677
RNF128	-0.511182456	0.010611	-0.217954938	0.27546	-0.430216323	0.031393
ELMSAN1	0.381597017	0.010639	-0.028782476	0.84761	0.085674949	0.566665
GBP5	0.7826053	0.010641	0.311206188	0.31151	0.491550834	0.10833
PABPN1	0.359833247	0.010639	0.004267725	0.97585	0.312193355	0.026442
XRCC2	-0.428923599	0.010653	-0.191576746	0.25059	-0.247343643	0.138106
LACE1	-0.576290308	0.010661	-0.182152356	0.41148	-0.213271867	0.334862
MMP19	0.818977229	0.010668	0.369096527	0.25401	0.500283733	0.119361
HIST1H1D	-0.586784848	0.01068	-0.042368261	0.85341	-0.040176317	0.860897
GLO1	-0.197528484	0.010696	-0.077047254	0.31729	0.106027628	0.167577
PDX1	0.924776165	0.010704	0.618239672	0.08875	1.306802659	0.000269
LDLRAD3	0.64506753	0.010718	0.457426151	0.06998	0.344309837	0.17459
NCCRP1	1.072289194	0.010724	0.819157496	0.05194	0.386909346	0.366809
CUEDC2	-0.350076693	0.010734	-0.382884771	0.00482	-0.173604498	0.197133
CCDC8	-0.352997357	0.010745	0.009511292	0.94484	-0.425077511	0.002079
TERF1	-0.326582159	0.010745	-0.340597548	0.00763	0.086599383	0.49472
NTPCR	-0.272773504	0.010762	-0.213987419	0.04417	-0.152444591	0.14922

USP35	0.596041642	0.010766	0.388878277	0.09661	0.342313991	0.143024
ECI2	-0.341029409	0.010777	-0.176584031	0.18293	-0.123324543	0.352027
C12orf75	-0.477581663	0.010796	-0.212302954	0.25543	-0.051416528	0.78281
POLI	-0.393025576	0.010799	-0.35231863	0.02208	-0.2288372	0.135524
SLC22A3	1.15149182	0.010803	0.701979054	0.1216	0.534079747	0.239776
SPRED3	0.669136095	0.010843	0.429621726	0.1045	0.279682473	0.289377
NR2C2	0.410252374	0.010848	0.240161082	0.13635	0.184867611	0.250896
KIAA0430	0.568440466	0.010861	0.010471982	0.96263	0.304591832	0.171925
MED24	0.331030529	0.010861	0.199326562	0.12495	-0.000356938	0.99781
NSDHL	0.32563919	0.010887	0.323629972	0.01115	0.491491572	0.000109
ALKBH8	-0.353468301	0.010918	-0.040500838	0.76363	-0.251100156	0.063093
CCRN4L	0.610596043	0.010926	0.360654382	0.13293	0.620913037	0.009287
C5orf34	-0.654793987	0.010931	-0.443055245	0.07922	-0.200664994	0.423181
COPRS	-0.359522421	0.010942	0.106029878	0.43754	-0.104388856	0.447494
RAD51	-0.576978717	0.010939	-0.272735422	0.22161	-0.501559206	0.02561
FGF7	0.674942966	0.010978	0.320913316	0.22697	0.831914949	0.001695
LRCH3	0.334610947	0.010988	0.001668479	0.98987	0.224249448	0.087416
TTC22	0.892014323	0.011006	-0.042040997	0.90548	0.267026653	0.448495
SP6	1.08549532	0.011039	0.540867694	0.20946	-0.061000693	0.889452
AAR2	0.277539477	0.011058	0.028637144	0.7939	0.083459266	0.444151
PRMT1	0.280954018	0.011058	0.186747135	0.09115	0.333515944	0.002521
JADE1	-0.964024891	0.011079	-0.769269555	0.04212	-1.038970068	0.006102
HDDC2	-0.285644805	0.011087	-0.023786353	0.82983	-0.235935765	0.03383
A2M	0.633039284	0.011117	0.496030463	0.04663	0.292495458	0.240738
DAPK1	-0.48851919	0.011126	-0.483731858	0.0119	-0.152222706	0.428371
CEP290	-0.321322731	0.011135	-0.124937524	0.32163	-0.123617607	0.325714
C4BPA	1.629929854	0.011177	1.424692834	0.02643	0.565359155	0.383103
RP4-765C7.2	-0.533296239	0.011184	-0.554008078	0.00825	-0.555297677	0.00806
NPM1P40	-0.636762675	0.011197	-0.506752484	0.03677	-0.672534396	0.006104
YPEL4	0.643927541	0.011206	0.120393263	0.63815	0.276557983	0.276389
UBE2L6	0.567463112	0.011213	0.79685993	0.00033	0.540312357	0.015298
GGA1	0.273924749	0.011233	0.044149672	0.68252	0.117516414	0.275199
TSC22D4	0.412179044	0.011281	0.069250503	0.67149	-0.011848791	0.94228
MMP9	2.316200837	0.011297	2.499985702	0.00617	-0.225188269	0.810504
ARL6IP6	-0.358893831	0.011322	-0.286746313	0.04067	-0.343562813	0.014188
RPLP0	-0.284730463	0.011318	-0.194043181	0.08427	-0.191842787	0.08785
EP300	0.36836984	0.011341	-0.01828883	0.90009	0.035025654	0.809858
C11orf54	-0.316994631	0.011378	-0.150004832	0.22855	-0.157557144	0.206087
CLSPN	-0.527148951	0.011372	-0.568350283	0.00628	-0.423970244	0.040916
HOXB2	0.717532094	0.011375	0.017141386	0.95233	0.574552983	0.04237
MON1B	0.243469192	0.011402	0.165931573	0.08401	0.09886545	0.302597
BRINP2	1.782366318	0.011413	1.116696317	0.11479	0.831952134	0.241108
EARS2	-0.284282656	0.011417	-0.237288579	0.03371	-0.053251118	0.632131
AC004166.6	0.903463963	0.011447	0.073520044	0.84397	0.466450288	0.197265
CMTM3	0.584956897	0.011441	0.58053575	0.01185	0.528286871	0.022076
FAM210B	-0.312144234	0.011443	-0.075865481	0.53501	-0.235627341	0.054437
GFPT2	1.05803239	0.011436	1.210964706	0.00371	0.900166332	0.031341

AP1S1	-0.340063427	0.011454	-0.339200978	0.01139	-0.35549557	0.007957
ECI1	-0.429680999	0.011464	-0.239843405	0.15137	-0.373495991	0.025746
FZD3	-0.430659289	0.011485	-0.230232044	0.17602	-0.278266884	0.101692
UGGT2	-0.265070081	0.011517	-0.022640115	0.82816	0.026462938	0.799596
C4orf46	-0.380758719	0.011545	-0.292913393	0.05073	-0.011064668	0.940775
C19orf70	-0.323229766	0.011551	-0.20394418	0.10332	-0.498171153	9.50E-05
MRPL43	-0.216543405	0.011562	-0.222588611	0.00859	-0.145649003	0.082278
ZNF561	-0.290461755	0.011558	-0.09551528	0.40246	0.024998783	0.825754
SGTA	0.366100556	0.011569	0.070503619	0.62727	0.143380154	0.322639
GCHFR	-0.405314558	0.01158	-0.120979911	0.44101	-0.424009337	0.007623
NUBPL	-0.434209923	0.01158	-0.228571988	0.17722	-0.193166984	0.253618
PTPRH	0.570376658	0.011584	0.407687929	0.07123	0.494530429	0.028569
YPEL2	0.461180465	0.011591	0.286539143	0.11625	0.531526966	0.003532
CENPV	-0.280910228	0.011607	-0.355884172	0.0013	-0.093547026	0.393522
CACYBP	-0.368359144	0.011622	-0.121793007	0.40157	-0.07299243	0.614715
DLD	-0.204858443	0.011623	0.021088031	0.7932	0.060221723	0.453159
PPM1H	-0.522926182	0.011655	-0.203036739	0.32548	-0.709496378	0.000609
DUSP10	0.457185259	0.011712	0.215333369	0.23786	0.620529857	0.000544
CC1L-ADOF	0.797460362	0.011709	0.04159086	0.89652	0.438960953	0.166092
RARS	-0.191725086	0.011746	0.031514601	0.67601	-0.021767938	0.772775
FAM199X	-0.271933824	0.011763	0.008531514	0.93664	-0.023749088	0.824818
GLUD2	-0.706711113	0.011768	-0.14470713	0.59801	-0.674494064	0.015254
LIMS2	1.414624603	0.011775	0.197951824	0.73174	0.514077338	0.366749
NCKAP1L	1.426999441	0.011789	1.121903596	0.05069	0.260330106	0.6516
BLMH	-0.389278179	0.011798	-0.198646032	0.19528	0.053296736	0.727307
P11-1143G9	-1.27550852	0.0118	-0.154394197	0.72611	-0.732775799	0.112123
SPATA20	-0.249800611	0.011809	-0.16496503	0.09425	-0.301213882	0.002279
ARL5B-AS1	-1.03260651	0.011835	-0.549944493	0.14579	-0.315053504	0.383928
PLEKHG2	0.455170365	0.011839	0.156876916	0.3868	0.28024606	0.121157
SUPT16H	-0.209066683	0.011832	-0.176380084	0.03331	-0.04333352	0.600389
BAZ2A	0.415619276	0.011865	0.033884793	0.83763	0.300824988	0.06864
B3GAT1	0.894516379	0.011873	-0.032859642	0.92808	0.300294542	0.400855
PHF1	0.451693019	0.011874	-0.014082765	0.93773	0.434319428	0.015101
TRNAU1AP	0.269281102	0.011879	0.194174902	0.06502	0.222664849	0.033478
SLC45A4	-0.395530378	0.011884	-0.215927239	0.16847	-0.356002957	0.023427
MIB2	0.437342351	0.011898	0.182497631	0.2958	-0.104038717	0.552036
RPAP3	-0.305393511	0.011908	0.050284688	0.67646	0.06071005	0.613196
NEDD1	-0.265618353	0.011922	-0.096527256	0.35616	0.085768225	0.410045
RPL11	-0.320679979	0.01193	-0.174400452	0.17136	-0.132470199	0.298768
RPL28	0.370082101	0.011931	0.058441517	0.69141	0.091490542	0.534257
GPR133	0.74482939	0.011938	0.14717492	0.62173	0.557300194	0.060127
CAMTA2	0.557385275	0.011945	0.021587462	0.92285	0.114076983	0.608136
DCAF7	0.493973244	0.011949	-0.026999384	0.89235	0.18123375	0.358451
IDH3A	0.292944917	0.011964	0.035460179	0.76116	0.098251834	0.397091
HYPK	-1.263695128	0.011981	-0.077862658	0.87604	-0.260521705	0.602129
FABP5P7	-0.566106676	0.012009	-0.522812271	0.02005	-0.17839854	0.425937
FAM127A	0.387738519	0.012005	0.083981118	0.58728	0.128390426	0.406109

PPP1R10	0.471305711	0.012006	0.259003816	0.16742	0.601498217	0.001341
CYCS	-0.252646926	0.012014	0.030849052	0.75819	0.081627006	0.415124
FAM129B	0.427134885	0.012027	0.389342098	0.02202	-0.043140884	0.799851
GABRE	1.001293796	0.012024	1.074761238	0.00694	0.619787418	0.120272
FBXW9	-0.447892503	0.012041	-0.355434695	0.04095	-0.501709096	0.004275
SEMA6D	0.858979543	0.012044	0.396786901	0.24658	0.417038563	0.223161
SLC35D2	-0.33934287	0.012044	-0.203039419	0.13136	-0.162656464	0.226313
MYL9	0.563233839	0.012064	-0.373587005	0.09683	0.210872996	0.347458
BDNF	0.799131401	0.01209	0.618971272	0.05188	0.921406212	0.003669
CAPN9	-0.98906881	0.012115	-0.982686077	0.01252	-1.248633657	0.001555
GNB2	0.285953698	0.012126	0.240567247	0.0345	0.112675949	0.322838
IER3	0.837904351	0.012127	0.565827966	0.09033	0.575732138	0.0848
LSS	0.481758725	0.012125	0.112257324	0.55911	0.169348358	0.378063
SH3BP5L	0.244828526	0.012112	0.089846183	0.35393	0.196161513	0.042813
C9orf40	-0.454050092	0.012138	-0.326040017	0.06467	-0.576221659	0.001303
EPHA2	0.49796406	0.012135	0.283331647	0.15344	0.316874849	0.110236
PEG10	0.548349729	0.01215	0.301860391	0.16812	0.281667724	0.198865
MLEC	-0.275778783	0.012165	-0.237078184	0.03102	-0.400544837	0.000269
TPM4	0.276414374	0.012179	0.13841595	0.20934	0.338484914	0.002132
CARS2	-0.233163535	0.012192	-0.163351842	0.07565	-0.007831391	0.931952
GFM2	-0.364011784	0.0122	-0.047399053	0.74223	-0.058560794	0.684407
TRAK2	-0.244525212	0.012202	-0.093932318	0.33077	-0.157031691	0.103574
MEX3A	0.39538977	0.01224	-0.10964454	0.4887	0.323299436	0.040121
MST4	-0.342875496	0.012239	-0.122685485	0.36768	-0.088922335	0.513818
MTCH1	0.225068029	0.012235	0.252535347	0.00482	0.160409243	0.073538
GET4	-0.436624301	0.012252	-0.616917941	0.00042	-0.212743361	0.218329
FBXO17	-0.35021752	0.012322	-0.432561385	0.00196	-0.267332781	0.053524
ORMDL1	-0.214373836	0.012384	-0.017589916	0.8345	0.000635055	0.993958
BRSK1	0.691274374	0.012416	0.603322835	0.02941	0.834411991	0.00247
MKL1	0.609062003	0.01242	0.468469935	0.05464	0.443516859	0.069004
HIC1	1.125965445	0.012437	-0.010733825	0.98127	-0.377149841	0.413443
KRIT1	-0.432777428	0.012444	0.021681694	0.89975	0.046891952	0.785164
MAP3K3	0.439572473	0.012442	0.172002239	0.32753	0.293837061	0.09337
RYR2	1.105975618	0.012453	0.766325185	0.08327	0.821506643	0.062342
FXYD3	-0.431562305	0.012463	-0.305889248	0.07638	-0.559275828	0.001199
CDC42BPA	-0.403702787	0.012483	-0.292647016	0.06981	-0.275855146	0.087417
FAM50A	0.279275936	0.012492	0.237836527	0.03253	0.2336512	0.035542
P11-195F19	-0.859053512	0.012487	-0.044006105	0.89415	-0.521513066	0.120782
SERPINB1	-0.425458905	0.012485	-0.374526037	0.02778	-0.285485781	0.093253
WDR37	0.328490739	0.012484	0.256013771	0.05036	0.279601458	0.032121
BMS1P10	-0.622497712	0.012573	-0.320356888	0.18642	-0.18206403	0.448426
LGALS9	0.735851725	0.012576	0.42892524	0.14673	0.366905807	0.214389
PRDX4	-0.349109939	0.012565	-0.14951635	0.28433	-0.169846064	0.223896
RARB	0.378248004	0.012572	0.086153198	0.5703	0.604501951	5.96E-05
RP11-583F2.	0.736399086	0.012577	0.567324755	0.05253	0.480844027	0.103741
RPL3	-0.30965944	0.01257	-0.204297344	0.09956	-0.24330546	0.049825
TTC37	-0.231164975	0.012587	-0.020571288	0.82343	-0.089016407	0.334028

HAND1	0.991999488	0.012627	-0.539097768	0.18631	0.712905347	0.073202
HCN3	0.735495998	0.012639	0.377860262	0.20062	0.684259532	0.020577
GJB1	-0.577983093	0.012649	-0.535214404	0.02069	-0.624239674	0.006997
ID3	0.536671661	0.012664	0.313817917	0.14514	0.296288437	0.168952
SVEP1	0.48133066	0.012686	-0.075615657	0.69565	0.323761516	0.093566
SLC31A2	0.683469724	0.012704	1.028306763	0.00011	1.1251261	1.99E-05
LY6G5B	0.559186116	0.012716	0.075121477	0.73997	0.478044366	0.032628
ADH1C	-1.287247895	0.012728	0.25136226	0.62462	-0.43654862	0.396054
FRG1	-0.26042965	0.012731	0.011461694	0.90843	0.053604607	0.583946
SRPRB	-0.358006541	0.012732	-0.037473633	0.79304	-0.248437665	0.082361
LRPAP1	-0.328831151	0.012739	-0.274761631	0.03706	-0.358719193	0.006509
MIS12	-0.356689138	0.012741	-0.004378927	0.97511	0.089006516	0.523394
HDX	0.562767915	0.012753	0.29440928	0.19405	0.668143038	0.002772
SAR1A	0.239823011	0.012772	0.296820236	0.00197	0.273815103	0.004279
MYLIP	-0.36746418	0.012786	-0.211516771	0.14602	-0.637150505	1.56E-05
DAPP1	1.410878823	0.012803	1.260803095	0.02623	0.740876077	0.19531
KNOP1	-0.356042787	0.012821	-0.404773711	0.0045	-0.272047712	0.056101
ALAS1	0.405086808	0.012842	0.164917975	0.31096	0.440723441	0.006682
MRPL48	-0.329832179	0.012852	0.065045385	0.6153	-0.084046495	0.51652
NLGN1	-0.707832006	0.012853	0.111735038	0.69031	-0.896927829	0.001566
ROCK1	-0.28554987	0.012852	0.011993596	0.91663	0.041277574	0.718348
XPA	-0.422490408	0.012855	0.002877527	0.98618	0.151044065	0.358486
SRBD1	-0.282508536	0.01288	0.261063525	0.01928	-0.094006353	0.401976
CD4	0.834705922	0.012887	0.134478632	0.70245	0.446363619	0.185516
PSMC1P1	0.276918613	0.012891	0.401751751	0.00028	0.488532164	9.77E-06
ELOF1	0.289615898	0.012897	0.156310152	0.1785	0.164849703	0.154325
TMEM8A	0.319288269	0.012901	0.182540804	0.15505	-0.087672154	0.49555
C5orf30	-0.3650505	0.012925	-0.077773655	0.5929	-0.237859838	0.10273
DENND4B	0.406028642	0.012945	0.083807475	0.60938	0.209086061	0.201196
FAM155A	0.693669145	0.012948	0.557790156	0.04534	0.35758073	0.201691
FUCA1	-0.273029726	0.012944	-0.078093338	0.47281	-0.421529886	0.00012
MAFF	0.671420227	0.012947	0.337401475	0.21231	0.732837877	0.006555
RP6-24A23.6	2.220092787	0.012928	2.594095702	0.00332	2.087740544	0.019123
SORBS3	0.477834111	0.012941	-0.358877034	0.06407	-0.077577458	0.688483
ZNF131	-0.29741119	0.012922	-0.179618891	0.13035	-0.052493347	0.657668
PCMTD2	-0.331967144	0.012968	-0.266207457	0.04456	-0.326164574	0.013687
SLC22A23	0.515097904	0.012984	0.424807968	0.04009	0.239114714	0.248526
RPL7	-0.282909244	0.012995	-0.168029866	0.14002	-0.129927031	0.253797
ZCCHC4	-0.382198923	0.013011	-0.201469464	0.18096	-0.308297818	0.040897
DAB1	1.282020373	0.013021	0.224282726	0.66734	0.606315985	0.241947
GPRC5A	0.634283232	0.013073	0.026141773	0.91858	0.225488121	0.377773
SCAMP4	0.358062943	0.01308	-0.047716103	0.7439	0.021453623	0.882523
CAP1	0.237557208	0.013099	0.324672305	0.00069	0.302197975	0.001582
CRCP	-0.249749459	0.013097	-0.117246551	0.24028	-0.221255721	0.027062
EID1	-0.332363698	0.013089	-0.114436812	0.39231	-0.140328551	0.29391
FIG4	-0.481019303	0.013102	-0.378703164	0.04885	-0.008719044	0.963789
CHST9	-0.743612849	0.013131	-0.173029695	0.55409	-0.221428245	0.449341

FAM84A	0.740574259	0.01313	0.365651401	0.22138	0.661878624	0.02655
NOSTRIN	-0.655597814	0.013131	-0.494664847	0.06157	-0.529370503	0.044657
POLQ	-0.520841947	0.013134	-0.102068962	0.62448	-0.084432404	0.685091
DUSP8P5	0.841761117	0.013141	0.310597226	0.37534	0.372507009	0.281861
GPR37	0.956101182	0.013154	0.207358132	0.59748	0.970942272	0.01128
NMNAT3	-0.956083602	0.013189	-0.556317014	0.12824	-1.465915352	0.000136
MRPL54	-0.303197354	0.013204	0.005692002	0.96127	-0.217860965	0.066565
C6orf203	0.402764789	0.013222	0.425802189	0.00823	0.493866671	0.002115
FAM177A1	0.279851951	0.013241	0.337890397	0.00259	0.363417459	0.001184
LRP6	0.316793478	0.013239	0.054079337	0.67184	0.039332969	0.757622
TBX19	0.920212852	0.013233	-0.150094763	0.70044	-0.00703564	0.985418
ZNF30	-0.717323697	0.013231	-0.199510659	0.47741	-0.791037215	0.005388
KCTD9	0.247575794	0.013252	0.295555925	0.00286	0.311830703	0.001611
SF3B1	-0.186388329	0.013253	-0.035587916	0.63583	0.048065289	0.522137
ADAM2	-0.84485309	0.013267	-0.13878432	0.67078	-1.025617741	0.002787
BATF2	1.179556967	0.013301	0.633109997	0.19037	-0.023496684	0.962995
CC2D1A	0.288885716	0.013308	0.033777483	0.77232	-0.016060065	0.890943
SRRM2	0.498007617	0.013323	-0.105974924	0.59872	0.196349152	0.329303
SNX4	-0.249997586	0.013354	-0.01269993	0.8989	0.108017998	0.278621
DERL2	-0.269712288	0.013401	0.113401005	0.29398	-0.003728625	0.97242
HSPA1L	1.739161771	0.013398	0.460294453	0.51507	0.038913581	0.956267
MN1	1.215777873	0.013416	0.555559241	0.25888	0.560330627	0.257422
CLPTM1L	-0.294619275	0.013428	-0.119280689	0.3129	-0.294515771	0.012998
RPLP2	-0.353726651	0.013455	-0.271306417	0.05783	-0.319655452	0.025434
P11-255H23	-0.51574608	0.013488	-0.266656743	0.19868	-0.541169974	0.009295
VPS28	0.232606974	0.013514	-0.096771464	0.30749	-0.00066704	0.994344
CYB5R3	0.37673188	0.013524	-0.092729603	0.54433	-0.231410355	0.13085
ZMYM5	-0.291947369	0.013538	-0.110172201	0.34131	-0.089441815	0.439119
ARHGAP12	-0.250559046	0.013547	-0.133978676	0.18457	-0.151589448	0.133317
PAM16	-0.601213034	0.013569	-0.605932005	0.01232	-0.6589238	0.00656
EYA3	0.27807695	0.013588	0.019634298	0.86176	0.280634083	0.012456
RPL13	-0.295831188	0.013592	-0.218508525	0.06814	-0.351420511	0.003355
TMEM255B	1.268793216	0.013604	1.687583608	0.00098	0.884739025	0.087181
ERGIC2	-0.294016197	0.013623	0.060713368	0.6059	-0.038274384	0.747283
LPAL2	1.166428176	0.013626	0.291683961	0.54336	0.93540417	0.047935
SNTB1	-0.446300695	0.013653	-0.567968786	0.00167	-0.536846666	0.002953
UST	0.762048762	0.013666	1.03133621	0.00083	1.446710506	2.55E-06
TRAF4	0.288957609	0.013722	0.041607738	0.72252	0.296993638	0.011036
ZNF136	-0.459644067	0.013728	-0.357839335	0.0526	-0.155030943	0.400027
CYP4F11	1.204244911	0.013752	0.522561842	0.29829	0.686425448	0.166315
WEE1	-0.263114704	0.013818	-0.146079817	0.16962	-0.019885988	0.851167
NDUFB11	-0.29734745	0.013823	-0.117741909	0.32339	-0.233091848	0.051239
RPS2P5	-0.662459808	0.013941	-0.124303387	0.63719	-0.368283751	0.164373
WLS	-0.317883694	0.01394	-0.352540354	0.00615	-0.508789492	7.97E-05
COL7A1	0.740428652	0.013963	0.202314871	0.50311	0.533603162	0.076779
BTNL8	1.033475156	0.014007	0.355162459	0.40531	0.000389546	0.999281
GPATCH2	-0.252271794	0.014014	-0.111941013	0.26985	0.002359242	0.981317

FAR1	-0.326212437	0.01402	-0.016719126	0.89952	-0.210600468	0.111954
NARS2	-0.444787129	0.014025	-0.425232861	0.0177	-0.461951962	0.009941
SNX14	-0.317943084	0.014033	0.046449163	0.71852	-0.014561732	0.910007
LURAP1L	-0.467732638	0.014076	-0.580759313	0.00222	-0.292535213	0.119602
CPSF3	-0.28165788	0.014082	-0.102856908	0.36589	-0.000369691	0.997402
PXDN	0.387715692	0.014105	0.056039774	0.72289	0.081571068	0.605656
ZBTB6	-0.247725645	0.014106	-0.084107043	0.39484	-0.032908881	0.737705
TM9SF4	0.215506859	0.014114	-0.117640252	0.18096	0.004868784	0.955751
PPP4R1L	0.579612229	0.014122	-0.27876229	0.2412	0.130525286	0.579425
INHBA	0.980986879	0.014201	1.097940165	0.00601	0.595361554	0.136759
ZFAND5	0.200307309	0.0142	0.23431534	0.004	0.336801633	3.45E-05
AVEN	-0.641096067	0.014213	-0.768947927	0.00321	-0.33045843	0.202803
GRIA2	1.883998514	0.014214	2.166214961	0.00483	1.7951076	0.019184
DYRK3	0.73252346	0.014229	1.017216507	0.00057	0.910060211	0.002084
MUC17	1.87709491	0.01425	0.271562186	0.72405	-0.138990864	0.859204
ALG1	-0.328745533	0.014265	-0.284935588	0.03156	-0.311481	0.019153
TESC	-0.963693629	0.014286	-0.522718624	0.1826	-1.012600685	0.009966
PPME1	0.310698637	0.014312	0.125390809	0.32257	0.174054287	0.169147
TMEM171	-1.312786933	0.014312	-0.767492793	0.13778	-1.629369896	0.002335
PSTPIP2	0.754965173	0.014343	0.918801117	0.00272	0.824914921	0.0072
CHMP5	-0.293605245	0.014365	-0.006274091	0.95786	-0.107902235	0.363912
UTP18	-0.434123329	0.014366	-0.189239319	0.28287	-0.267298348	0.129946
UBE2D3	0.235893071	0.014377	0.242161104	0.01186	0.486474131	4.19E-07
WBSCR22	-0.36265136	0.014382	-0.084470853	0.56543	-0.398763077	0.006827
GALNT5	-0.474253615	0.014396	-0.307619617	0.11144	-0.266990569	0.166749
USP18	-0.678600286	0.014416	0.061210037	0.81376	-0.771382012	0.004864
AMOTL1	0.371737064	0.014425	-0.030010396	0.84343	0.121914369	0.422136
GAREM	0.385490479	0.014436	0.284501913	0.07066	0.602970848	0.000126
NDST1	0.391003345	0.014439	-0.105315999	0.51053	0.021600609	0.892477
TBCE	-0.262042138	0.01445	-0.271928111	0.01022	-0.134914748	0.198108
RPS13	-0.270449979	0.014492	-0.060349669	0.58489	-0.1420602	0.19852
MXRA7	0.293075876	0.014498	0.035020135	0.77033	0.387907833	0.001165
ERGIC1	-0.276896182	0.014513	-0.523069292	3.82E-06	-0.431732269	0.000135
TMED10	-0.268505954	0.014513	-0.146439084	0.18218	-0.255601224	0.019862
C11orf49	-0.332104258	0.014551	-0.06974241	0.60426	-0.332801704	0.013932
LTB4R2	0.687887924	0.014549	0.616884126	0.02821	0.832625647	0.002572
SV2A	0.555889369	0.014565	0.186883656	0.41294	0.264267153	0.245718
KLHL14	1.259682668	0.014571	0.292427845	0.57229	0.521461571	0.313069
FBXO48	0.519966303	0.014587	0.298795755	0.16182	0.181172724	0.398101
FTSJ2	-0.317915606	0.014621	-0.030223284	0.81322	-0.068333855	0.592858
RILPL1	0.438209228	0.014638	0.463526727	0.00951	0.549636649	0.002063
CD99P1	0.495877583	0.014652	0.450900248	0.02371	0.23342215	0.247839
PARD3B	-0.850812126	0.014652	-0.733031729	0.03449	-0.728159486	0.036881
CMC2	-0.310927428	0.014672	-0.221808222	0.07953	-0.071285901	0.570168
RPL3P2	-0.427636973	0.014671	-0.140562016	0.41672	-0.356617442	0.040125
NREP	0.499487257	0.014682	-0.028855857	0.88794	0.498531491	0.014837
?P11-15H20.	-0.476942864	0.01469	-0.314303956	0.10681	-0.420542604	0.030615

PLEKHF2	-0.334237809	0.014699	-0.166855407	0.21677	-0.308332169	0.023054
TRMT112	-0.234298242	0.014705	-0.020743455	0.82687	-0.078873293	0.405312
RAP1GDS1	-0.266286947	0.014717	0.018945255	0.86089	-0.002110686	0.984393
TTK	-0.459881268	0.014737	-0.023947538	0.89832	0.139794667	0.455226
PLCB3	0.256965589	0.014779	0.145965974	0.16525	-0.109198241	0.300508
ZNF484	-0.281250246	0.014782	-0.14403866	0.20422	-0.081899385	0.468661
PTPN9	0.291997762	0.014794	0.284262169	0.01734	0.384652697	0.001239
SUCLA2	-0.329325669	0.014849	-0.001177966	0.99299	0.1624303	0.224638
OCLN	-0.517063483	0.014857	-0.314446927	0.13791	-0.179104394	0.397896
CD93	1.242300226	0.014866	0.314804888	0.54428	0.754056299	0.141307
BCAS1	-0.575706883	0.014874	-0.608449817	0.00997	-0.538715364	0.022534
CRBN	-0.251633842	0.014894	0.027921776	0.78405	-0.069189139	0.495846
DOCK9	0.538876346	0.014895	0.080232941	0.71716	0.196082338	0.37537
TIPIN	-0.539559914	0.014893	-0.305268411	0.16144	-0.512451449	0.019475
CETN3	-0.415773326	0.014932	-0.075301603	0.65448	-0.173519628	0.30285
CHRM2	0.642787063	0.014955	0.041362505	0.87565	0.603527191	0.022075
MOSPD1	-0.334535008	0.015013	-0.079175164	0.55982	-0.07102696	0.596956
STAG1	-0.293720793	0.015032	0.00215351	0.98572	-0.086629807	0.471131
ZNF469	1.276429407	0.015109	1.042980907	0.04718	0.305717288	0.563627
POLR1D	-0.311533473	0.015141	-0.20113529	0.11579	-0.197129769	0.122752
GIT1	0.444865905	0.015154	0.113058703	0.53835	0.157762512	0.389886
GAK	0.381744839	0.015162	0.073919664	0.63807	0.051482439	0.743293
DUSP23	-0.414044074	0.01517	-0.125463397	0.43983	-0.465697572	0.005292
SLC7A9	1.750883899	0.015182	0.340174046	0.64156	0.231296239	0.752845
GGT1	1.105391342	0.015187	0.390527476	0.39424	0.291162156	0.525627
KIF11	-0.396262138	0.015203	-0.095896914	0.55556	-0.030828553	0.849611
VAT1L	1.436584294	0.015208	2.431566396	3.46E-05	0.523648506	0.38067
MAP3K1	0.263046133	0.015214	0.443826532	3.93E-05	0.404515594	0.000179
LETMD1	-0.315734499	0.015221	-0.328831836	0.01117	-0.245665448	0.057396
ANKRD27	0.404276483	0.01523	0.241715742	0.14553	0.252387803	0.12856
KIAA0101	-0.496125293	0.015233	-0.34691376	0.08772	-0.298299793	0.141854
AJUBA	0.373030775	0.015248	0.121723003	0.42928	0.238505726	0.1202
ASRGL1	0.565204119	0.015266	0.648800324	0.00513	0.893930918	0.000106
FBXO16	-0.751974116	0.015292	-0.497670797	0.10234	-0.447359207	0.143831
FGD5	1.102212213	0.015298	1.147958652	0.01104	-0.109871302	0.813925
ORC6	-0.381404206	0.015298	-0.21166982	0.17527	-0.182808584	0.241142
COL4A5	-0.459558406	0.015315	-0.414392854	0.02868	-0.166130191	0.380144
PPP1R12B	0.330448414	0.015333	-0.068088221	0.61887	0.262637153	0.053628
METTL17	-0.228705311	0.015374	-0.199033443	0.03329	-0.094315861	0.30883
BAMBI	0.6458795	0.015399	0.823389719	0.00194	0.288371804	0.279957
GLRX2	-0.427799412	0.015394	0.166427699	0.31403	-0.150823745	0.370753
SMIM12	-0.272400263	0.015404	-0.075716751	0.49703	-0.084317222	0.447031
ZNF83	-0.236376773	0.015394	-0.166391979	0.08628	-0.022588618	0.814814
WDR34	-0.398996392	0.015423	-0.19526945	0.23158	-0.384808262	0.018831
NIF3L1	-0.273267283	0.015456	-0.255067648	0.02191	-0.19303519	0.081781
PLRG1	-0.220954282	0.015471	-0.001243671	0.98903	0.107498877	0.232498
MAF	0.659728181	0.015497	0.569864678	0.03647	0.269837494	0.322591

SLC9A2	-0.827902476	0.015504	-0.465731362	0.17215	-1.139134212	0.000866
SGK2	-0.427132644	0.015524	-0.424831655	0.01553	-0.46243248	0.008509
RBM3	-0.160142348	0.015534	0.236049255	0.00032	0.111003496	0.090638
DNPH1	-0.458442475	0.015545	-0.2266046	0.22328	-0.282201702	0.129169
ANKRD50	0.355496863	0.015581	0.242553846	0.09865	0.417093775	0.004463
ZDHHC5	0.27981902	0.015641	0.053755111	0.64198	-0.015103726	0.896082
SLC26A2	0.616751943	0.015662	0.526175591	0.03924	-0.051912653	0.83908
PPIP5K1	0.366349708	0.015678	0.302291455	0.04531	0.20332678	0.178246
SPTBN1	-0.190698717	0.015671	-0.24855168	0.00163	-0.166089265	0.035238
TMSB4XP6	0.440932645	0.015678	0.749016821	3.94E-05	0.655557644	0.000321
KDELR1	0.278324526	0.015697	-0.145550724	0.20707	-0.17947052	0.119599
MAP7	-0.433560387	0.015702	-0.183592004	0.30469	-0.116854411	0.513403
SCN5A	1.387416961	0.015705	0.507882614	0.3824	1.107567766	0.052405
ST3GAL2	0.491327659	0.015694	0.317034563	0.11888	0.040172804	0.843505
PHC2	0.257593668	0.015742	0.065423858	0.53926	0.078858743	0.459083
NDUFS1	-0.181307945	0.015769	0.051209717	0.49339	-0.044984058	0.547282
PINLYP	-0.708657092	0.015778	-0.453181628	0.1201	-0.589725501	0.042417
CRTC1	0.489027458	0.015792	-0.183269413	0.37531	-0.070732607	0.730339
SFXN2	-0.533466409	0.015805	-0.262595332	0.23341	-0.243041127	0.269545
USP32	0.249639354	0.015803	0.030736551	0.76613	0.192538999	0.061619
SFMBT1	-0.353853737	0.015815	-0.016475526	0.90876	-0.213231289	0.139766
DCUN1D4	-0.297527599	0.015834	-0.176637882	0.15019	-0.130465424	0.287032
ZNF584	0.378411218	0.015847	0.184943044	0.23834	0.065419281	0.675979
TMEM237	-0.552900431	0.015913	0.050880578	0.82312	0.088780015	0.696223
CLEC2D	0.618034393	0.015941	0.571687028	0.02544	0.779104421	0.002232
INTS3	0.222847853	0.015942	0.13609779	0.14027	0.160032366	0.082608
RELB	1.516312541	0.015949	0.743029272	0.24154	0.716683533	0.257571
NPIPB3	0.567689904	0.015963	-0.2109136	0.37674	0.195981714	0.406744
RIPK2	0.500306999	0.015975	0.113466144	0.58569	0.636056495	0.002085
SLC25A38	-0.269037418	0.016005	-0.187985309	0.08833	-0.197719187	0.072202
SPINK5	-0.447140282	0.016008	-0.068964421	0.70781	-0.50899557	0.00585
SUSD1	-0.273959043	0.016012	-0.159074717	0.15912	-0.129375892	0.251439
GSAP	-0.407550396	0.016028	-0.163329921	0.32662	-0.217984792	0.19009
TSR2	-0.249821602	0.016055	-0.111091709	0.27457	-0.259567067	0.011111
SMPDL3B	-0.750318557	0.016066	-0.165812932	0.58794	-1.043368087	0.000846
TMEM229B	-0.616150767	0.016069	-0.256804063	0.30789	-0.627776505	0.013299
FASTKD1	-0.345021417	0.016085	-0.042107521	0.76592	-0.017525669	0.901112
HCFC1	0.455057083	0.016083	-0.253864936	0.18008	-0.002716801	0.988557
ATP5EP2	-3.361576141	0.016109	-1.347319994	0.32375	-2.251189029	0.100964
SDSL	-0.703178656	0.016106	-0.067677391	0.80162	-0.425222355	0.124571
NAT14	-0.381743945	0.016167	-0.172518967	0.26487	-0.37259532	0.017259
PCNX	0.342522741	0.016194	0.206895943	0.14596	0.266908109	0.060637
TPK1	0.526506417	0.016195	0.310296759	0.15484	0.470166123	0.031021
CREB3L3	0.991086157	0.016253	0.418000822	0.31142	1.06948172	0.009444
EFNB2	0.275779421	0.016248	0.448013567	8.89E-05	0.222724829	0.051777
IDH3G	0.255399154	0.016249	0.12916544	0.2234	0.31404922	0.002942
ITGB3	0.792748272	0.016243	0.647301903	0.04945	0.204592851	0.538642

SELM	2.462263926	0.0163	1.878376914	0.06692	1.980684288	0.053445
KANSL3	0.275566735	0.016323	0.012780368	0.91125	0.160976618	0.159274
FBXL12	0.211013951	0.016332	-0.058654761	0.50669	0.107399996	0.217046
SEC24C	0.225255079	0.016372	0.167451388	0.07386	0.21954381	0.018976
KLC1	0.317692564	0.016397	0.021533543	0.87071	0.322765363	0.01447
RP1-20N2.6	-0.64032738	0.016399	-0.262934836	0.31023	-0.453867234	0.080971
TIMM8A	-0.4248065	0.016405	-0.052481692	0.75691	-0.226282572	0.185197
CDK16	0.215710772	0.016495	0.028959612	0.74753	0.126009068	0.160371
EIF1AD	0.256775663	0.016499	0.08803517	0.41249	0.085494988	0.42252
TMEM9B	-0.247509217	0.016493	0.03677477	0.7166	-0.120954875	0.234191
MPP5	-0.197815812	0.016514	-0.189690658	0.02005	-0.06157682	0.446444
TIMM21	-0.315054968	0.016533	0.044768191	0.72887	-0.126997278	0.327928
FGD4	-0.404738084	0.016561	-0.199583964	0.23625	-0.331987909	0.048902
MFSD2B	-1.06314509	0.016556	0.138928193	0.74205	-0.478855364	0.263323
CAB39	0.247232884	0.016569	0.182479446	0.07648	0.228070274	0.026614
SERBP1	-0.269376807	0.01662	-0.201244207	0.07338	-0.111635792	0.320538
EFCAB2	-0.408630465	0.016628	-0.200672068	0.23293	-0.04985385	0.764871
MELK	-0.510242946	0.016669	-0.185541477	0.38128	-0.090803812	0.668047
MANBAL	-0.249088662	0.016675	-0.142254513	0.15954	-0.03497412	0.726542
ABRACL	-0.303406782	0.016684	-0.096271886	0.44009	-0.152448384	0.221471
ASAP1	0.463448085	0.016722	0.089592737	0.64436	0.219962471	0.255944
FAM107B	-0.412019879	0.016714	-0.113692453	0.50801	-0.224469559	0.191492
KIF1C	0.239633498	0.016722	0.256993522	0.01021	0.178523312	0.074317
SNX6	-0.164389148	0.016732	-0.038309459	0.57385	0.01055241	0.876547
ULBP3	-0.593873067	0.016735	-0.532334951	0.02987	-0.894763061	0.00033
CDH11	0.518017625	0.016756	0.38687451	0.074	0.405594763	0.061047
MBD1	0.232208465	0.016759	0.092476605	0.33947	-0.001507975	0.987571
SERPINA3	1.324695367	0.016757	2.335445081	2.47E-05	1.559935595	0.004849
EMP2	0.536783457	0.016788	0.368345123	0.1005	0.254086973	0.257302
RBM43	-0.488493014	0.016795	-0.117800334	0.55328	-0.15693323	0.431302
TMEM198B	0.401258913	0.016833	0.090496742	0.59046	0.025030867	0.881776
RAP1A	-0.360350993	0.016855	0.034081601	0.81979	-0.163791953	0.274256
BAG2	-0.496752977	0.016879	-0.160323231	0.43634	-0.431846014	0.036688
TAB2	0.261131307	0.016875	0.125609781	0.25003	0.157071429	0.150302
ABI3	1.217758773	0.016884	0.055854506	0.9177	1.210058375	0.016476
FOXC1	1.739331515	0.016907	1.677388417	0.02119	0.611108644	0.403126
AFAP1L1	0.956799845	0.016965	0.765604373	0.05567	0.798948262	0.043818
IP6K1	0.412366305	0.016974	0.32403943	0.05914	0.43836269	0.010693
TMEM106C	-0.422600196	0.016982	-0.310962689	0.07765	-0.277020164	0.116216
MAP1B	0.803471488	0.016999	0.698009121	0.03812	0.382863598	0.255504
GOLM1	-0.443057083	0.017016	-0.24855671	0.18055	-0.065623223	0.723659
RIC3	-1.987858705	0.017014	0.001368485	0.99867	-2.337868615	0.004845
BRWD1	-0.203430668	0.017048	0.046126896	0.58706	-0.056108397	0.508482
TMEM66	-0.185563195	0.017049	0.058733817	0.44652	-0.085254064	0.270163
RP11-213G2.	-0.599921848	0.017055	-0.311618506	0.19892	-0.153340431	0.52141
PIGX	-0.264872739	0.017062	-0.075485328	0.49076	-0.178059915	0.103867
PIGB	-0.298939256	0.017069	-0.413254301	0.00083	-0.353924217	0.00392

ABCB8	0.34107741	0.017117	0.053917326	0.70597	-0.241491929	0.09264
BCL2A1	2.155187858	0.017107	2.13826995	0.01782	2.078382581	0.021436
DDR2	0.604565283	0.017115	0.287909247	0.25625	0.376795848	0.137202
RASGEF1A	1.404692228	0.017116	1.016113578	0.07602	1.222152069	0.03338
ZSWIM7	-0.354507843	0.01713	-0.105429256	0.47136	-0.206381091	0.157043
CEP170B	0.406279666	0.017147	0.011210486	0.94764	-0.160854015	0.346719
TMEM117	-0.390671618	0.017147	-0.243652234	0.13329	-0.18660514	0.248441
RPL10	-0.248657433	0.017158	-0.369797754	0.00039	-0.226958532	0.029534
P11-231C14	0.859527475	0.01717	0.338231731	0.35094	1.082118657	0.002546
TGM1	1.548584274	0.017166	-0.459486892	0.4856	0.229199277	0.727611
PITX2	1.012607386	0.017214	0.245387417	0.56538	-1.063626707	0.014048
ATP1A1	0.304492688	0.017257	0.307883918	0.01603	0.191751728	0.133682
HSBP1	-0.187112959	0.017313	-0.008145798	0.91692	-0.022975515	0.768184
IFT43	-0.403338623	0.017309	0.015391097	0.92457	0.002621569	0.987101
SEMA4C	0.36340476	0.017329	0.19230763	0.20796	0.24807148	0.103992
UBE2Q2	-0.311637969	0.017328	-0.101817642	0.43202	-0.071529038	0.58149
ABHD6	-0.43873384	0.017349	-0.363372478	0.04647	-0.41624705	0.022813
C4orf3	-0.233858123	0.017357	-0.225317142	0.02169	-0.208588334	0.033386
ATP6V1A	-0.336238131	0.017373	-0.176684994	0.21026	-0.033838684	0.810335
DESI1	0.442458941	0.017398	0.285715752	0.12449	0.552842985	0.002913
DZIP3	-0.240523213	0.017432	0.219674743	0.02687	-0.044333348	0.656404
FH	-0.331625188	0.017436	-0.08004664	0.5642	-0.095728248	0.490385
HSD17B8	-0.433723893	0.017454	-0.385158489	0.03148	-0.350524285	0.048611
AC091654.7	-1.344087168	0.017468	-0.330636362	0.50848	-0.48007143	0.340292
WDR12	-0.447737674	0.017465	-0.252852909	0.17843	-0.267800074	0.153822
ANKFN1	-2.015961632	0.017479	0.047416004	0.95094	-1.146398256	0.155861
BEX5	-0.902220844	0.017479	-0.189202185	0.59046	-0.532737488	0.137802
ABCC1	0.518054929	0.017519	0.25858567	0.23567	0.025893484	0.90549
ANKRD32	-0.501875689	0.017526	-0.125903171	0.54628	-0.306914018	0.140994
ETV6	0.364392214	0.017588	0.153148124	0.31859	0.160150706	0.296592
TAF10	0.328805275	0.017601	0.139121269	0.31507	0.305094943	0.026935
PITPNM1	0.421235702	0.01761	-0.179946163	0.31172	0.049213028	0.782354
MFAP4	0.504420597	0.017632	-0.121064313	0.56961	0.065149217	0.759385
CYP20A1	-0.526036061	0.017643	-0.117186441	0.58772	-0.121209701	0.57595
SAMD4B	0.388180825	0.017649	-0.232595717	0.16391	-0.010469155	0.949442
ZMAT2	-0.265173828	0.01765	-0.146107999	0.18796	0.021707475	0.84434
CTDNEP1	0.494614457	0.017659	0.003949023	0.98494	0.218217649	0.295298
CA2	-0.613666001	0.017675	-0.53907004	0.03705	-0.688121691	0.007786
SIPA1	0.566288293	0.017697	0.330775924	0.17123	0.132645171	0.580032
TNFRSF13C	0.807607767	0.017697	0.262917988	0.45087	0.636206041	0.060744
CLIP2	0.412199723	0.017705	-0.045207355	0.79491	0.150204266	0.387511
ANKRD36	-0.370798223	0.017728	-0.350977245	0.02435	-0.217070005	0.163675
CCNA2	-0.412011989	0.017766	-0.073499245	0.67009	0.086706606	0.614441
VIM	0.472089367	0.017797	0.210218434	0.29133	0.260467896	0.191034
RNF44	0.355278985	0.017808	-0.139731972	0.35818	-0.310122435	0.041177
AC004951.5	1.308629242	0.017822	0.579562508	0.30252	0.313304224	0.580504
FOXD2	1.467922667	0.017845	1.518354119	0.01413	-0.989895683	0.122624

6-Mar	-0.232856495	0.017861	-0.141543226	0.14895	-0.093304539	0.34094
COLEC11	1.354212583	0.017871	0.973870008	0.08881	0.804802824	0.160304
ASB2	1.023784772	0.01791	-0.569530284	0.20684	0.603701939	0.166308
TNFRSF14	1.057596906	0.017954	1.178669797	0.00742	0.874754743	0.054496
BRD3	0.361124503	0.017978	-0.029712589	0.84585	-0.067528193	0.65863
PIGM	-0.339016188	0.017972	-0.164797743	0.24439	-0.263249573	0.063311
SAMD1	0.300953846	0.017975	0.094230585	0.45906	0.086854495	0.494546
SYT2	0.871230133	0.017967	0.01791721	0.96214	0.887694292	0.015187
PLXNA1	0.355578269	0.018006	-0.059758069	0.69146	0.057367822	0.703104
IFI27L2	-0.397169125	0.018071	-0.135710202	0.40056	-0.543006889	0.001094
LAMP1	-0.27652101	0.018072	0.066525279	0.5679	-0.285939541	0.014357
SEC11A	-0.268290668	0.018068	0.040698874	0.71879	0.009922808	0.930046
YJEFN3	0.559345712	0.018075	0.141831053	0.55653	0.304238191	0.198866
EPHA4	0.745335481	0.018088	0.393548054	0.21184	0.244078869	0.438808
LONP2	-0.196393087	0.01809	-0.095703627	0.24828	-0.160771145	0.052234
AP000347.2	0.426418692	0.0181	0.266364452	0.13604	0.357855507	0.044929
SIRT7	0.387402191	0.01816	0.36088062	0.0269	0.399859866	0.014146
CROT	-0.541393414	0.018172	-0.21782927	0.33737	-0.243704664	0.2844
CHSY1	0.398491005	0.018196	0.508744986	0.00253	0.280243171	0.096622
DCLK1	0.999933061	0.018191	1.725212755	3.73E-05	0.779765078	0.064286
PGS1	0.290148859	0.018207	0.031628374	0.79776	0.220081812	0.071425
SIPA1L3	0.56619239	0.018211	0.594327278	0.0132	0.33859236	0.158286
SLC26A10	1.333582802	0.018213	0.912704288	0.11174	0.987641482	0.083396
STAC3	1.267947066	0.018204	1.353538369	0.01007	0.887362041	0.095552
FOXI3	1.471723371	0.018295	1.097948759	0.08013	0.467903378	0.465976
NAE1	-0.236236647	0.018317	-0.101145986	0.30749	-0.001412598	0.988592
FDPS	0.407584719	0.018348	0.229144635	0.18484	0.433626733	0.012069
IRS2	0.596179256	0.018345	0.195625488	0.44252	0.392832863	0.119743
GCFC2	-0.418362977	0.018382	-0.22123774	0.21022	-0.193945184	0.271968
MRPL35	-0.267298815	0.018407	0.024514986	0.8266	-0.012216771	0.913126
FAM72B	-1.056247391	0.018469	0.278270408	0.51373	0.295977352	0.48218
PRRC1	0.232714145	0.018472	0.089999397	0.36078	0.028454975	0.772534
CXCR4	-0.854778072	0.018484	-0.861599497	0.0175	-0.987722021	0.006467
CA5BP1	0.462560842	0.018526	0.288049189	0.14338	0.380765029	0.051138
GCLC	-0.532500186	0.018515	-0.516144715	0.02201	-0.455774194	0.042965
TOPBP1	-0.361857495	0.018523	-0.153244308	0.31759	-0.002686118	0.986014
DUSP9	-0.895839017	0.018568	-1.101849304	0.00383	-0.829824574	0.028038
ZUFSP	-0.318380801	0.018577	-0.106417382	0.42476	0.117818265	0.372933
COG5	-0.268596889	0.018593	-0.085917468	0.44934	-0.117061096	0.302655
FUT10	-0.341253686	0.018601	-0.1613771	0.26128	-0.195132486	0.174325
LARP6	0.560324709	0.018599	0.704283358	0.00299	0.38990053	0.100635
ROR2	0.469185204	0.018628	0.371676831	0.06228	0.073278226	0.713478
TIMMDC1	-0.256915559	0.018644	0.042572089	0.69048	0.095192275	0.372664
TMEM30A	0.292091221	0.01864	0.590315699	1.94E-06	0.242541997	0.050592
WDR75	-0.20974702	0.018638	-0.005136871	0.95344	0.033323004	0.704362
WDR46	-0.347872801	0.018686	0.005726914	0.96883	-0.005482742	0.970179
MMP7	1.635223234	0.018734	2.789988955	6.00E-05	1.347584135	0.052718

TM7SF3	-0.481463866	0.018757	-0.194448492	0.34105	-0.509633374	0.012661
RNF126	0.368906741	0.018766	0.097793632	0.53452	-0.286640931	0.071397
OR7E12P	-1.369217164	0.018803	-0.512746298	0.33882	-1.962938006	0.00122
ZC3H3	0.423958353	0.018817	-0.080717066	0.66178	0.051426889	0.777876
ZNF134	0.317215796	0.018812	0.283236391	0.03496	0.313987282	0.019429
ASNSD1	-0.380040701	0.018851	0.11270748	0.4834	0.125421509	0.434855
NAGA	-0.215246238	0.01886	-0.098627681	0.27241	-0.320523251	0.000404
OXGR1	2.907784207	0.01885	2.356058806	0.05806	-0.452416756	0.722427
SYMPK	0.337938262	0.018856	0.300651866	0.03662	0.466394141	0.001147
CD101	0.993952841	0.018872	0.604908195	0.15215	0.369913002	0.382279
UQCRB	-0.313440113	0.018898	-0.02720894	0.83811	-0.097015784	0.466315
SLC9A4	-0.811699148	0.018985	-0.262086454	0.4477	-0.894511058	0.00971
ASIC3	1.229182134	0.019028	0.652632423	0.22316	0.127237295	0.820431
RPS20P22	1.034880045	0.019037	0.522553693	0.24246	0.549689827	0.220905
TCEA1P2	-0.427398036	0.019086	-0.224344273	0.21313	-0.166370608	0.354474
BGN	0.761112906	0.019112	0.473355012	0.14523	-0.369593855	0.258453
CCT2	-0.233265541	0.019109	-0.066402248	0.50353	0.115636482	0.243326
PDE6B	-0.653711296	0.019101	-0.28687391	0.29772	-0.509519157	0.065752
PSMC1	-0.425612123	0.019099	-0.056686314	0.75385	-0.093461585	0.604925
ZNF652	0.294518654	0.019138	0.012743941	0.91932	0.100037243	0.425924
PHGR1	1.596933281	0.01916	0.756254742	0.2699	-0.10813594	0.876047
SNRPGP15	1.334530899	0.019149	0.330465132	0.56516	0.930870158	0.102718
SPAG9	0.324337317	0.019156	0.234461534	0.08972	0.370355216	0.007365
AARS2	-0.227519764	0.019205	-0.036217868	0.70263	-0.18588857	0.051032
CLCN4	-0.630928615	0.0192	-0.260145042	0.32913	-0.116860114	0.658294
EFCAB11	-0.406940339	0.019206	0.007831634	0.96339	-0.263946715	0.122332
ZNF596	0.638458946	0.019206	0.484466192	0.07309	0.547661315	0.041277
FHOD1	0.39987823	0.019221	0.078563583	0.6458	0.254805484	0.135982
FHL2	0.482567756	0.019242	0.079586023	0.69974	0.426557795	0.038344
IGF2BP1	0.318594907	0.019238	-0.050474908	0.71092	0.065119734	0.632049
RNF19B	0.367928391	0.019277	0.481714178	0.002	0.19117179	0.222768
ASGR1	0.910312374	0.019322	0.356217895	0.36519	0.567915889	0.147102
JPH2	0.881329053	0.019331	-0.5032367	0.21404	0.48279281	0.203602
PCTP	-0.325679218	0.019352	-0.20856483	0.13123	-0.260031023	0.060028
SPG7	-0.252922715	0.019366	-0.093631963	0.38419	-0.133087044	0.216063
CRHR1-IT1	0.280935578	0.019399	-0.098629425	0.41176	0.149090199	0.212521
RMND1	-0.331220281	0.019437	-0.043685534	0.75275	-0.16539757	0.233776
BLCAP	0.342888759	0.019451	0.041201817	0.77938	-0.042920437	0.770653
GDA	1.03697183	0.019477	0.426209057	0.33765	0.888078365	0.04535
H3F3AP4	1.225456357	0.019501	0.606784883	0.24768	0.541954913	0.301852
PPP1R3C	-0.846338063	0.019517	-0.797595793	0.02753	-1.274997717	0.000441
ARSK	-0.413081105	0.019563	-0.427774114	0.01481	-0.45471127	0.009532
KCTD11	0.502311929	0.01956	0.457591961	0.03318	0.119533062	0.579078
FAM127C	0.380924392	0.019596	-0.136986566	0.41001	0.02291307	0.889036
MSH3	-0.266555092	0.019593	-0.101414999	0.36808	-0.144143365	0.200209
PRDM5	-0.438710058	0.019599	-0.07435434	0.68786	-0.219134866	0.237641
PPIL4	-0.192801359	0.019622	-0.108819119	0.1822	0.035410691	0.662149

SLC35E1	0.292637579	0.019617	0.193850788	0.12169	0.125988089	0.314973
TNC	1.069763326	0.019628	1.395884819	0.00233	0.478910208	0.296231
SLC26A9	-0.630448405	0.019667	-0.581483671	0.0314	-0.779931807	0.003923
TTI2	-0.504409209	0.019674	0.483588057	0.02374	-0.103828724	0.628661
SH3GLB1	0.213145773	0.019691	0.334672918	0.00024	0.200244856	0.027937
TP53BP2	0.345083207	0.019719	0.102290127	0.48941	0.375447989	0.010994
C15orf62	0.672487482	0.019746	-0.041262439	0.88986	-0.205820322	0.492894
C16orf89	-0.896894728	0.019747	0.169102542	0.64556	-1.001648446	0.01278
MTSS1	0.570439621	0.019756	0.615895855	0.01132	-0.145854485	0.554542
RAB11FIP3	0.548105806	0.01976	-0.045681817	0.84682	0.183897144	0.435152
NARFL	0.376273345	0.019767	0.172437068	0.28308	0.221271224	0.168318
HADHA	-0.185459444	0.019809	-0.190437852	0.01649	-0.105184916	0.18469
FAM129A	-0.667112763	0.019824	-0.086153852	0.76161	-0.958800203	0.000855
SMG1	0.337346614	0.019829	0.14167096	0.32783	0.249214236	0.085091
GALE	-0.315052814	0.019838	-0.19866346	0.14009	-0.491262436	0.00028
EGR3	1.24318274	0.019865	0.925699069	0.08394	0.487710382	0.366552
GPR125	-0.277516955	0.019866	-0.19872191	0.09466	-0.020002631	0.86629
MAP3K13	-0.223795718	0.019849	0.074937982	0.43179	-0.022025896	0.81715
NGDN	-0.255106473	0.019856	-0.024278032	0.82084	0.029808284	0.779441
CDKAL1	-0.208361375	0.019905	-0.254117048	0.00417	-0.164263541	0.063207
CLIC5	1.384897897	0.019925	0.370383473	0.53433	1.392945919	0.019197
CDH12	-0.901652794	0.01995	0.158662742	0.67923	-0.7728851	0.044936
CROCC	0.4585893	0.019946	0.024933519	0.89965	-0.100344269	0.61198
KIAA0391	0.291397307	0.019951	0.349119419	0.00511	0.277665751	0.02578
SDC1	-0.504392729	0.019956	-0.589209932	0.00652	-0.93980309	1.47E-05
VWA9	-0.186052279	0.01997	-0.002738841	0.97229	0.022306108	0.776719
STAG3L3	0.293595682	0.019989	0.049292954	0.69706	0.173251939	0.169471
SLC5A3	0.315598131	0.020015	0.384419211	0.00457	0.013987635	0.917909
ATXN1L	0.194546342	0.020023	0.011269681	0.89325	0.178394131	0.032631
FLAD1	-0.341876137	0.020038	-0.078928169	0.58722	-0.207464475	0.153459
TMEM60	-0.375210778	0.02004	-0.183737257	0.24164	-0.003282026	0.983052
SAYSD1	-0.300150402	0.020076	-0.136830877	0.27658	-0.271665136	0.031661
CD9	0.632162122	0.020082	0.493579694	0.06941	0.132325066	0.626968
CLDN23	-0.620600538	0.020112	-0.3948026	0.13123	-0.683727648	0.009745
SOX21	-0.839555348	0.020149	-0.418358737	0.24394	-1.104168792	0.002251
SELENBP1	-0.340423791	0.020185	-0.451903498	0.00201	-0.245979535	0.090023
ATG4D	0.423403185	0.020229	0.220411386	0.22372	0.25397103	0.159781
EXD2	-0.235442288	0.020227	-0.18399058	0.06555	-0.272742541	0.006313
SMOC2	0.577680227	0.020295	-0.051708072	0.83577	0.70928342	0.004318
TTC17	-0.225351392	0.020305	-0.132153815	0.17207	-0.126208473	0.191804
MAST3	0.6262001	0.020322	0.209929968	0.43767	0.133948745	0.621494
MANF	-0.392339774	0.020342	-0.136402253	0.41739	-0.125701794	0.454552
UBE2G2	0.212980083	0.020375	0.083608733	0.36014	-0.024577563	0.787731
NSUN3	-0.369776835	0.020398	-0.162011744	0.30357	-0.234822154	0.134797
NOMO3	0.825273764	0.020481	0.333229631	0.35011	0.933266533	0.008609
RTF1	0.209353049	0.02049	-0.054967228	0.54313	0.089799732	0.318399
NUDT12	-0.281709095	0.020532	-0.152945623	0.20258	-0.218873488	0.068161

EPAS1	0.456804054	0.020554	0.230917211	0.24163	0.499282536	0.011302
HIST1H1C	-0.415465375	0.020543	-0.201157193	0.26172	0.036879924	0.836904
QRICH1	0.168646195	0.020555	0.087883434	0.22662	0.164948738	0.022552
C14orf178	1.22187642	0.02057	0.006312099	0.99115	0.145079514	0.793393
FNIP2	-0.340441003	0.020598	-0.24190242	0.09949	-0.225394486	0.124708
RBL2	-0.182365527	0.02062	-0.117048773	0.13472	-0.140491983	0.072331
RRAS	0.478821911	0.020641	0.153033518	0.46079	0.171581649	0.407529
AKT1	0.243326457	0.020672	-0.118299285	0.26055	0.039518217	0.706858
TNKS1BP1	0.376328156	0.020689	0.479433969	0.00318	0.312217275	0.054898
CCDC147	-1.026198842	0.020718	-1.034219086	0.01746	-0.694777079	0.096492
RIOK1	-0.391720676	0.020743	-0.069843625	0.6771	0.100302048	0.548681
ACER1	1.296383872	0.020802	1.449467419	0.00866	0.865925571	0.126632
EMC3	-0.225184764	0.020792	-0.162075525	0.09281	-0.057255183	0.551941
ESF1	-0.350303598	0.0208	-0.280849178	0.06217	-0.07012697	0.639585
MRPS36	-0.417568903	0.0208	-0.189371788	0.28723	-0.074955259	0.673148
ERF	0.379001108	0.020814	0.222458092	0.17597	-0.004441411	0.978472
TONSL	-0.410018516	0.020822	-0.470047893	0.00787	-0.708719566	6.55E-05
CCND3	0.410232618	0.020878	0.10472468	0.5556	0.023837108	0.89325
AMICA1	-0.726860606	0.020946	-0.683502371	0.02972	-0.484094243	0.122734
LMBR1L	0.417782512	0.021003	0.41371277	0.02201	0.454150269	0.011834
CHFR	0.305313103	0.021054	0.12910453	0.32864	0.133838608	0.31062
HIST1H2BN	-0.438482471	0.021077	-0.143623379	0.44657	-0.064878827	0.730371
NFIB	0.370357972	0.021094	0.324991196	0.04266	0.094864182	0.554547
DDIT3	-1.226801189	0.021128	-1.469376696	0.00577	-1.452240707	0.00634
PHYKPL	0.288215787	0.021133	0.017608145	0.8878	0.150510928	0.22626
ACE2	1.332098492	0.021169	1.205395362	0.037	1.539824269	0.007702
PISD	0.314785852	0.021207	0.297105272	0.02885	0.387528674	0.004204
AKAP12	0.586157979	0.021214	0.108601766	0.66951	0.419070061	0.099472
C8orf47	-0.755259007	0.021222	-0.167476225	0.60074	-0.160859388	0.614885
PICALM	0.243316337	0.021234	0.227241324	0.03121	0.360201896	0.000633
SPATA33	-0.476254596	0.021266	-0.188443223	0.3555	-0.202395047	0.319299
GIMAP1	1.238100249	0.021302	0.921682544	0.08899	0.805769446	0.138299
RAB20	-0.498879049	0.021324	-0.307905944	0.15098	-0.751414759	0.000528
RAB6A	0.246299208	0.021341	0.195597981	0.06714	0.267783149	0.012112
TMX3	-0.277791011	0.021366	0.022165587	0.85315	-0.130779111	0.275641
FREM2	-0.432654772	0.021396	-0.133329387	0.47743	-0.259449153	0.166827
C6orf223	1.205690809	0.021413	0.686355032	0.1952	0.518824563	0.326186
MSL2	-0.263842145	0.021421	-0.190110523	0.0956	-0.102929822	0.365995
METTL21A	-0.261581692	0.021434	-0.300374908	0.00844	-0.199766588	0.07891
SIRT5	-0.37156802	0.021475	-0.121222671	0.44801	-0.042076151	0.792241
NEXN	0.52019818	0.021487	-0.150381208	0.50972	0.419306628	0.062313
SH3BP1	0.363588662	0.02149	0.171594521	0.27755	-0.119950388	0.449098
ZBTB47	0.424548095	0.021504	0.358030711	0.05112	-0.090031434	0.632994
CNRIP1	0.778598303	0.02154	0.40549625	0.2332	0.518461747	0.126122
DSEL	0.59269456	0.021545	0.625812306	0.0151	0.329076991	0.202007
PRSS3P1	-1.075754622	0.02154	0.172911348	0.71164	-1.79292144	0.000129
AFF1	0.366639191	0.021584	0.013279743	0.9337	-0.088573414	0.579121

EXOC5	-0.275641208	0.021608	-0.085227625	0.47553	0.06328771	0.595665
SOX12	0.366517147	0.021602	-0.249886791	0.1217	-0.18308496	0.255076
SLC12A4	0.469414209	0.021626	0.03146927	0.87773	0.395696787	0.05255
GPN1	-0.203887944	0.021661	0.009241704	0.91547	0.074196509	0.392696
KCTD21	0.408083487	0.021673	0.331379462	0.06203	0.226373986	0.201708
RETSAT	-0.374602279	0.021669	-0.350898718	0.03103	-0.429482568	0.008358
VTI1B	-0.194105893	0.021678	0.02692643	0.74805	0.02429488	0.771385
ASTN1	1.643384777	0.021693	1.210010152	0.09182	0.946855523	0.191332
PPWD1	-0.25839005	0.021688	-0.011343714	0.91866	0.081853973	0.459685
ATP5O	-0.270074263	0.021706	-0.083096074	0.47805	-0.172564048	0.140914
ZBTB8B	-0.434396259	0.021707	-0.292211358	0.11781	-0.368000391	0.049048
MFSD5	0.346717969	0.021733	0.000170617	0.9991	0.150554173	0.315974
MINPP1	-0.299926466	0.021732	-0.082737053	0.52236	-0.121429738	0.348693
RAMP2	0.651467135	0.021779	-0.001099008	0.99698	0.567799878	0.043603
RHOBTB3	0.340581224	0.021775	0.193421642	0.19249	0.263934184	0.075135
TARBP2	-0.314775483	0.021775	-0.032282115	0.81079	-0.081547481	0.545669
PCDHGB7	0.467140546	0.021787	-0.394402026	0.05524	-0.136455731	0.504921
BRI3BP	-0.3189054	0.021811	-0.165816728	0.23215	-0.373668264	0.007137
AXL	0.473941729	0.021823	0.01702557	0.9345	0.078004777	0.706246
NTRK2	1.290762012	0.021844	0.855369067	0.12931	0.680281101	0.227827
AS3MT	-0.344342371	0.021919	-0.251715697	0.08801	-0.250674393	0.088127
STK39	-0.246287316	0.021936	-0.221208305	0.03886	-0.328162974	0.002192
GRAMD4P7	0.908791413	0.021974	0.979675181	0.01224	0.378027192	0.350304
GNS	-0.257841904	0.021987	-0.150205925	0.1811	-0.301778733	0.007299
C19orf26	0.976060234	0.021998	0.44578368	0.3019	0.192547903	0.657503
DERA	-0.291883834	0.022007	0.053286176	0.67078	-0.156717833	0.213469
PAPD7	0.29667521	0.022017	0.111571931	0.38783	0.164680062	0.202191
CRYL1	-0.416309384	0.022037	-0.391239378	0.03083	-0.705650983	0.000106
GTF2H2	-0.38384148	0.022035	0.234157699	0.15631	-0.052794089	0.750307
P11-206L10	-0.947173032	0.022029	-0.299454164	0.46374	-0.424563733	0.296858
ELF2	-0.20594071	0.022058	-0.000467134	0.99581	0.041437881	0.640781
RCCD1	-0.396656339	0.022073	-0.357260976	0.03737	-0.175511531	0.30344
VOPP1	-0.241652089	0.022141	-0.147052683	0.16028	-0.446951766	2.17E-05
C3orf33	-0.467641435	0.022149	-0.282170793	0.1547	-0.430551505	0.030425
ETF1P2	0.813662025	0.022177	0.663744118	0.0617	0.246485538	0.495933
GLUL	-0.319890206	0.022182	-0.1681359	0.22873	-0.192891735	0.167335
SPATA18	-0.556704278	0.022229	-0.623132558	0.01021	-0.637142075	0.008635
ARF1	0.235123562	0.022278	0.070339956	0.49382	0.029863636	0.771522
CD276	0.279739478	0.022277	0.161472328	0.18684	0.15925662	0.192681
HIST1H4J	-0.626639673	0.022279	-0.241373725	0.37818	-0.178532209	0.514449
MATN1	1.319411627	0.022278	0.932275743	0.10609	0.378133491	0.521353
MED16	0.348800536	0.022267	0.011485629	0.94012	-0.028276845	0.853446
POM121	0.86832741	0.022283	-0.210391828	0.58111	0.023139996	0.951548
ZFYVE27	0.32391569	0.022259	0.160458197	0.2572	0.238491799	0.090162
OSBPL5	0.463073586	0.022297	0.05798567	0.77574	0.248440305	0.220923
TOX4	0.279216897	0.022306	0.167547607	0.1695	0.269031692	0.027202
NUP210	-0.640798236	0.022393	-0.629767321	0.02455	-0.859885451	0.00216

ACADS	-0.408923843	0.022401	-0.193416857	0.27005	-0.659369413	0.00023
CD37	-1.776533193	0.022426	0.49406838	0.52253	0.085044029	0.912488
TVP23B	-0.325558822	0.022421	-0.062382915	0.66025	0.009748403	0.945158
RFWD3	-0.313276619	0.022433	-0.049569187	0.71665	-0.01664236	0.902743
ZNF740	-0.326510572	0.022466	-0.057118139	0.68853	-0.07852218	0.581148
ZNF480	-0.306375153	0.022481	-0.350908092	0.00848	-0.06124594	0.644809
DNALI1	-0.36004865	0.022507	-0.445137651	0.00476	-0.128515814	0.408688
MRPS17	-0.286007899	0.022517	-0.123196441	0.31871	-0.296014788	0.017109
KIAA0141	-0.275712683	0.022548	-0.434447716	0.00032	-0.278902866	0.020269
CTAGE5	-0.261682271	0.022557	-0.141534194	0.21378	-0.10648799	0.348992
ADPRM	-0.423217023	0.022571	-0.239265185	0.18277	-0.199686525	0.263285
TMEM38A	-0.568368491	0.022588	-0.525242862	0.03432	-0.533079634	0.031563
LSM2	-0.300876066	0.022597	-0.223491312	0.08681	-0.146812519	0.259058
CAMK2D	-0.252477657	0.022609	-0.005183616	0.96251	-0.225582827	0.041097
MORC3	-0.215174552	0.022626	-0.066004268	0.48112	-0.01865076	0.841894
ZNF747	0.385700122	0.022686	0.381211421	0.02316	-0.016565673	0.922586
ABHD12	-0.260413927	0.022785	-0.016868215	0.88167	-0.228209848	0.045144
COG4	-0.2521015	0.022792	-0.194258771	0.07633	-0.192461133	0.078806
HOOK1	-0.508530352	0.022792	-0.059818417	0.78808	-0.140392214	0.528518
LPHN2	0.303590712	0.022787	0.244078663	0.06701	0.386931649	0.003671
HIST1H2BD	-0.388822008	0.02282	-0.121581809	0.47584	-0.049302633	0.772391
PAFAH1B2	-0.182610414	0.022819	0.13197009	0.09845	0.101421063	0.203689
TMEM14B	-0.230031365	0.022853	-0.082540831	0.40779	-0.165831986	0.09696
KIAA0930	0.32800823	0.022867	0.232680669	0.10626	0.09152397	0.524844
TRAF3IP2	0.340639356	0.022863	0.286822878	0.05484	0.168961629	0.25855
XRCC6BP1	-0.471283067	0.022874	-0.135478507	0.49031	-0.245211192	0.2179
WTIP	0.551332068	0.022914	0.524079058	0.0306	0.549630801	0.023212
BDKRB1	1.212653054	0.022974	0.97252341	0.0694	1.136211267	0.032834
FSTL3	0.557765278	0.022956	0.540037054	0.02734	0.175727715	0.47469
TMEM37	0.617180775	0.022974	0.693799184	0.01046	0.506084105	0.061996
TMSB4XP4	-1.059082203	0.02297	0.059741342	0.88634	0.152011456	0.713798
RAD50	-0.250018987	0.022988	-0.219979223	0.04458	-0.267444685	0.014545
CYB5R2	-0.571010124	0.023	-0.4055029	0.1043	-0.485795649	0.052207
EFR3B	0.668274011	0.023067	0.070696232	0.81117	0.644829808	0.027979
PEAR1	0.968357797	0.023067	0.592886397	0.16413	-0.275652894	0.51964
TTF2	-0.450954596	0.023094	-0.42104474	0.03338	-0.249424684	0.20694
NFE2L1	0.414587822	0.023129	0.082631058	0.65081	0.246761862	0.176359
IGIP	-0.496789683	0.023143	-0.02756097	0.89468	-0.676594707	0.001881
ATAT1	0.348455968	0.023162	0.023693113	0.88027	0.213652027	0.164502
SPG20	0.36255758	0.023158	0.186182855	0.24292	0.647967621	4.59E-05
ITGA7	0.947274242	0.023174	0.63946327	0.12583	0.052880878	0.900768
TRMT13	-0.340109918	0.023183	-0.350314827	0.01855	0.02453954	0.867875
ITSN1	0.255389344	0.023201	0.0798456	0.4782	0.493743149	1.07E-05
NRIP3	0.629393028	0.023253	0.131976827	0.63718	0.541431544	0.050298
PNPLA6	0.499458319	0.023249	0.27400071	0.21265	0.168918392	0.444565
TIMP3	0.555158862	0.023241	0.308112918	0.20791	0.185709081	0.447938
TMEM126A	-0.364080832	0.023237	0.035978562	0.81717	-0.088842258	0.568792

ACYP1	-0.529845045	0.023273	-0.323106959	0.15697	-0.277424705	0.222333
SPDYE3	0.399690703	0.023354	0.126588353	0.47499	0.279477201	0.110405
ZNF433	-0.409442631	0.023371	-0.2116206	0.23078	-0.104931835	0.544791
RP11-986E7.	1.12977241	0.023385	1.574973208	0.00114	1.380618882	0.004554
UBAP2L	0.334334869	0.023384	0.097016263	0.51095	0.258497583	0.079538
C4orf19	0.418315978	0.023405	0.745469442	4.95E-05	0.550557447	0.002756
CD46	-0.232122546	0.023424	-0.254902378	0.01274	-0.145179545	0.155799
GATA3	1.238676395	0.02347	0.039827905	0.94288	0.208286452	0.70625
FAM92A1	-0.293573286	0.023492	0.0086776	0.94617	0.168490164	0.18804
GPA33	1.500162294	0.023492	0.278496495	0.67481	0.307969235	0.642925
DNAJC10	-0.24324816	0.023523	0.079110924	0.46031	-0.042437461	0.692154
POGLUT1	-0.280635328	0.023532	-0.224715589	0.0681	-0.257317986	0.036242
ZNF362	0.503219253	0.02357	0.085444747	0.70134	0.061886412	0.780782
KLHDC2	-0.323005173	0.023611	-0.095368425	0.50113	-0.146787482	0.300975
MPP7	-0.354678374	0.023604	-0.133784228	0.38724	-0.299408537	0.053694
CGN	-0.352236922	0.023684	-0.250726933	0.10684	0.076512417	0.622319
POU4F1	1.482869805	0.023698	0.866300706	0.19048	0.970366838	0.141056
AGBL4	-0.879381325	0.023732	-0.418204017	0.26389	-0.735989787	0.052988
GDF15	-0.845970174	0.023735	-1.442315635	0.00012	-0.876903961	0.019042
RAB4A	-0.285753762	0.023728	-0.171416011	0.17229	-0.110145543	0.379552
RGMA	0.667044273	0.023722	-0.049385246	0.86731	-0.168586264	0.56923
HOXB3	0.699074965	0.023763	-0.509238844	0.10255	0.400954107	0.195169
RHOV	1.033624449	0.023763	1.235564827	0.00667	0.645383829	0.158748
FNTB	0.364331052	0.023774	-0.161626476	0.31491	-0.131096551	0.414697
LAMP3	0.880505055	0.023791	1.26116004	0.00082	0.48766699	0.210775
CYB561A3	-0.270899762	0.023827	-0.171785828	0.14741	-0.277265798	0.019697
SLC39A5	-0.399855211	0.023832	-0.549900213	0.00187	-0.418102424	0.017957
PHB	-0.299033857	0.023843	-0.143810618	0.27642	-0.168933896	0.20107
AUNIP	-0.465588669	0.023865	0.018605578	0.92585	-0.100987594	0.614538
PTGFR	0.858015596	0.023872	-0.16833497	0.66819	1.229820133	0.00104
PRELID1	0.409986195	0.023887	0.273646003	0.13145	0.517381897	0.004312
HMGB1P10	-1.689129547	0.023901	-1.089750258	0.13231	-0.933199334	0.194624
CPNE5	1.316518943	0.02392	1.22652334	0.03669	0.780288717	0.197486
AC093642.5	-0.765980904	0.023953	-0.572240102	0.08793	-0.83014696	0.014037
C1QBP	-0.330905677	0.023961	-0.146636509	0.31573	-0.133365283	0.36131
CDA	1.33519001	0.023956	0.636842744	0.28604	0.122165859	0.840463
NAPA	0.24522552	0.023965	-0.104024968	0.33967	0.114831683	0.289462
SMAD6	0.464491761	0.023969	0.696098114	0.00063	0.037188058	0.856278
TMCO3	-0.369846812	0.023935	-0.143013283	0.38129	-0.105182389	0.519276
ZNF684	-0.592031458	0.023943	-0.192661906	0.44531	-0.20327703	0.420571
HMMR	-0.465521206	0.023998	-0.024926042	0.9032	-0.000209324	0.999184
HEATR3	-0.352809945	0.024019	-0.053243869	0.73083	-0.127540799	0.410308
PTPRF	-0.310418977	0.024025	-0.241011511	0.07955	-0.369284161	0.00726
AMPD2	0.281697619	0.024057	-0.144262286	0.24828	0.063801202	0.609621
PRDM1	0.601814326	0.024083	0.170587009	0.52461	-0.371777531	0.166049
ASAH2B	-0.429183877	0.024123	-0.159903174	0.39461	0.068709409	0.711778
HLA-A	0.347948524	0.024119	0.148400251	0.33611	-0.073241773	0.635145

HLCS	-0.359657467	0.024121	-0.453045127	0.00435	-0.554262018	0.00048
PHB2	-0.200075106	0.024113	-0.14301566	0.10617	-0.093529881	0.290192
CSNK1D	0.259587898	0.024149	0.150225116	0.19205	0.123049361	0.28474
TFB2M	-0.336539809	0.024175	-0.07541423	0.60328	-0.000552573	0.996939
MGLL	0.79263522	0.024224	0.436055708	0.21529	0.762167951	0.030183
PLCB2	-0.981033033	0.024225	-0.83772411	0.05411	-0.917511469	0.031385
SYNPO2	0.522348743	0.024228	-0.05474859	0.81344	0.580520768	0.012209
ZNF761	-0.307585118	0.024233	-0.210635188	0.1201	-0.18165615	0.179347
MGST1	-0.397118843	0.024246	-0.20249596	0.24958	-0.304140145	0.083805
TSHZ1	0.426808863	0.024261	0.086192942	0.64988	0.229515837	0.225201
TTC19	-0.243717449	0.024284	-0.021254497	0.84245	-0.164108502	0.125662
BEAN1	1.118516833	0.024311	0.899685801	0.06846	0.441802608	0.37725
SLC25A14	-0.40924076	0.024332	-0.199399967	0.25346	0.026776874	0.875849
HDHD1	-0.268057267	0.024359	-0.14600523	0.2129	-0.122506686	0.293995
OVOL1	-0.41308798	0.024354	-0.507084871	0.00563	-0.599478454	0.001082
EML2	-0.476572887	0.024383	-0.138718453	0.50644	-0.58161734	0.005694
GUCA2A	2.256322494	0.02438	-0.329943236	0.75945	0.803214177	0.433751
ECT2	-0.360199013	0.024392	-0.124466708	0.43564	0.091360308	0.566894
ZNF865	0.575898237	0.024407	-0.098565761	0.70915	-0.092388359	0.725317
FILIP1L	0.38502953	0.024422	0.384846118	0.02435	0.400232487	0.019158
PPP4R2	0.237287591	0.024421	0.558139097	1.11E-07	0.432843466	3.79E-05
NPRL2	-0.47109221	0.024441	-0.360444276	0.08403	-0.332370916	0.111152
HIST1H4A	-0.481272886	0.024451	-0.25203817	0.23816	-0.234394538	0.272558
HOMER3	0.545226015	0.024471	0.42860207	0.07713	0.360347452	0.13809
TCTEX1D2	-0.584738079	0.024483	-0.379038807	0.13657	0.000488996	0.998436
ST8SIA6	0.759101863	0.024497	0.299169015	0.37728	0.507779393	0.132759
PAK1IP1	-0.286686308	0.024509	-0.121104006	0.33481	-0.008921602	0.943005
DLX1	3.074244046	0.024539	3.889469463	0.0041	1.702611081	0.213678
PANK1	-0.293134681	0.024552	-0.167507076	0.19583	-0.008954479	0.94465
TRIM34	-0.504060694	0.024623	0.104686434	0.63118	-0.238172159	0.278254
HELB	0.320309394	0.024631	0.207931081	0.14394	0.043358265	0.760519
UBXN8	-0.421816421	0.024654	-0.237103325	0.19754	-0.318236299	0.082353
CLDN16	1.560008291	0.024673	1.141500419	0.10355	0.380237531	0.599127
DDB1	0.159637842	0.024666	0.107686471	0.12901	0.216247309	0.002282
PAPSS2	0.568894568	0.024742	0.713459515	0.00478	-0.326575774	0.200543
RASSF4	0.506125371	0.024745	0.202725583	0.37027	0.311086682	0.16725
RP11-137H2.	-0.279435453	0.024782	-0.121714231	0.32135	-0.277511327	0.024305
CTSA	0.453767283	0.024837	0.114084982	0.5727	-0.23692516	0.242158
C1orf159	0.397792491	0.024845	0.24445371	0.16714	0.361952316	0.039651
SPEF2	-0.634783214	0.024885	-0.470671027	0.09332	-0.383581108	0.171011
VCP	0.217723829	0.024908	0.38467857	7.26E-05	0.315420995	0.00114
ITSN2	0.437312877	0.024936	0.555133272	0.00423	0.353108461	0.069604
LNX2	-0.350400687	0.024937	-0.079527989	0.60912	-0.033929327	0.827177
SDHAF1	-0.418341647	0.024934	-0.210911831	0.24313	-0.30724837	0.08989
SHISA3	1.043443429	0.024936	1.368154607	0.0032	1.095307651	0.018386
NFIA	0.563386944	0.024985	0.406867894	0.10541	-0.039761089	0.87444
PDGFC	0.478754926	0.024983	0.462118423	0.0304	0.660527975	0.001963

CA4	2.115235519	0.025055	1.112768051	0.24031	1.788098872	0.058402
UQCRC1	-0.305169408	0.025069	-0.14193211	0.29681	-0.239824167	0.077979
SOD3	1.04535929	0.025085	-0.046990723	0.92075	-0.214091949	0.651069
ANKS6	-0.314259417	0.025113	-0.359764366	0.01008	-0.265004607	0.05813
C15orf39	0.383629719	0.025123	-0.059508445	0.73044	-0.165314568	0.336998
SIVA1	-0.389608412	0.025124	-0.164003702	0.3423	-0.515451697	0.002917
GRB2	0.245302332	0.02522	0.102778771	0.34797	0.233925609	0.032375
STAG2	-0.192540262	0.025217	-0.083218646	0.33181	0.069825254	0.414958
HSPA4L	-0.541908859	0.025304	-0.432997431	0.07316	-0.160944652	0.504935
ANKEF1	-0.422597387	0.025316	-0.039314592	0.83322	-0.247539628	0.186318
ERCC6L2	-0.22788408	0.025319	-0.056362117	0.57821	0.062830428	0.534437
AGO2	0.419763346	0.025343	0.053295017	0.77654	0.263905461	0.159578
DFNA5	-0.591061064	0.025346	-0.086954579	0.73851	-0.366299753	0.162618
LCP1	1.528401973	0.025391	2.308697381	0.00072	1.599147306	0.01928
WRN	-0.324636341	0.02539	-0.097091308	0.49941	-0.260997751	0.06989
ZBTB7C	-0.622358451	0.025397	0.051989329	0.85054	-1.266094252	6.22E-06
COA5	-0.315722034	0.025442	-0.144780466	0.29998	-0.26438454	0.057895
POR	0.339190657	0.025494	0.061646849	0.68492	0.224514391	0.13931
BAALC	1.388164484	0.025528	1.18607845	0.05575	0.477418039	0.446567
DPYSL4	0.681002916	0.025514	0.213448879	0.48436	0.310371117	0.309027
GPR110	0.986169724	0.025525	0.753249833	0.08819	0.727608265	0.099301
OLFM2	0.511212032	0.025531	0.079320556	0.73091	0.144799967	0.526994
RGN	-0.947221462	0.02555	-0.302168826	0.44726	0.026938541	0.94564
SBSPON	-0.697059424	0.02554	-0.138291416	0.65357	-0.429104478	0.165571
ZBTB24	-0.308758235	0.025545	-0.027442393	0.83999	-0.076662829	0.572672
FGFBP1	1.023036124	0.025562	0.131452768	0.77574	-0.025996601	0.955135
INTS10	-0.250606211	0.025575	-0.131792388	0.23587	-0.146670163	0.186999
SMU1	-0.124494117	0.025608	-0.045541937	0.40835	0.098629115	0.070963
PSMG1	-0.344488563	0.025617	-0.052348951	0.73141	-0.098332322	0.518852
LYRM1	0.35678013	0.02565	0.354943875	0.0257	0.497652396	0.00167
CBLN3	0.895722941	0.025697	0.2921682	0.47094	-0.085331503	0.835164
TM2D1	-0.239928216	0.025691	-0.061600093	0.55841	-0.091519319	0.38407
FUT6	0.817802966	0.025721	0.342644153	0.36659	0.312457449	0.405645
GTPBP6	0.324865494	0.025721	0.162716937	0.26363	-0.154824438	0.291646
EDNRA	0.581219888	0.025774	0.457436107	0.07923	0.655926982	0.011812
GUCD1	0.261773537	0.025789	0.075203574	0.52183	0.103655448	0.37699
SASH1	-0.309248414	0.025811	0.020954484	0.87835	-0.166294778	0.224995
DPP9	0.419860103	0.025828	0.185100083	0.32552	0.173580038	0.357473
KATNB1	0.455900976	0.025899	0.29058751	0.15305	0.236219198	0.248707
PAAF1	-0.407648537	0.025964	-0.197864779	0.27465	-0.485383851	0.007628
PARP1P1	0.707838423	0.026016	0.636214053	0.04391	0.708081967	0.023637
ACSL5	0.439680078	0.026045	0.495824955	0.01197	0.201117425	0.308654
LRRC20	-0.543353675	0.026062	-0.607260922	0.01271	-0.575829305	0.01758
UGT2B11	-1.083176383	0.026075	-0.612762439	0.20456	-0.257579555	0.589128
EIF3E	-0.355402401	0.026105	-0.361600779	0.02356	-0.042634721	0.789445
FILIP1	0.721588521	0.026112	0.204438174	0.5307	0.462259197	0.154753
IRF3	0.271806364	0.026124	0.077118712	0.52636	0.108332988	0.37425

GTF2H2B	-0.359963075	0.02614	-0.177788836	0.26816	-0.146561182	0.360765
ARHGAP35	0.396522268	0.026149	0.238033806	0.18124	0.545761992	0.002148
CBR4	-0.261753368	0.026175	-0.220018539	0.05947	-0.083973617	0.470141
SPDEF	-1.051341031	0.026238	-0.796906189	0.09098	-1.272106167	0.007149
IKZF2	-0.421753148	0.026261	-0.2780952	0.14176	-0.292682719	0.122102
RPS19BP1	-0.286889662	0.026262	0.017321488	0.89152	-0.399980537	0.001807
ENPP4	-0.434919586	0.026282	-0.086571701	0.65589	-0.200830807	0.301647
TNFAIP8	0.503851682	0.026286	0.778980647	0.00058	0.79163104	0.000467
MAX	0.266784113	0.026413	0.28816224	0.01616	0.392341307	0.001025
TTF1	-0.352539081	0.026409	-0.194683718	0.21424	-0.05962946	0.700643
ZDHHC3	0.23361713	0.026422	0.150780043	0.14987	0.160529832	0.124945
CXADR	-0.319792598	0.026433	-0.195964027	0.17301	-0.260398026	0.070157
ATP2C1	-0.219734677	0.026446	0.197521799	0.04449	0.035565511	0.717682
HIST1H4F	-0.529806989	0.02645	-0.186714698	0.43314	-0.081420005	0.732381
CYP2C8	-1.235048399	0.026469	-0.345077658	0.51731	0.284730665	0.588101
FAM229B	-0.61045746	0.026466	-0.046702982	0.86123	-0.217429037	0.418763
FAM149A	-0.415286839	0.026497	-0.177902823	0.33608	-0.244889164	0.187059
GAP43	2.161846398	0.026529	3.005651784	0.00197	1.199016564	0.220658
POLR2F	-0.265536166	0.026632	-0.195370741	0.09815	-0.167810426	0.153326
ZFPL1	0.390856921	0.026625	0.273102518	0.11995	0.493660368	0.004849
AIM1	0.628594435	0.026658	0.471652756	0.09618	0.254579894	0.369786
ATP5F1	-0.210540499	0.026659	1.95E-05	0.99984	0.010450342	0.911995
ABCD1	0.473231175	0.026668	0.41954151	0.04865	0.052654815	0.807372
NDUFA8	-0.241141904	0.026681	-0.196312275	0.06726	-0.335756236	0.001843
PRRG1	0.516995605	0.026689	0.386239819	0.09774	0.384686957	0.099105
CCDC3	0.656013244	0.026709	0.375596006	0.20482	0.594699759	0.044412
TTC4	-0.245650853	0.026721	0.156022008	0.14667	0.121200234	0.257869
AKIRIN1	0.255375457	0.026743	0.173225629	0.13217	0.3350868	0.003528
RP11-427H3.	0.371360679	0.026748	0.150453014	0.36972	0.118397021	0.480133
TMED2	-0.203989293	0.026773	-0.018283807	0.84234	-0.127914203	0.164255
OXNAD1	-0.262950711	0.026781	-0.072055487	0.53418	-0.033654029	0.76984
G3BP1	-0.207999823	0.026789	-0.066671217	0.47719	-0.046763505	0.617959
MMP2	0.398506344	0.02684	-0.032105022	0.85847	0.02302441	0.898207
RN7SL467P	-0.596719804	0.026836	0.002077618	0.99348	-0.390605255	0.132974
RORB	1.818576842	0.026838	3.427222916	2.51E-05	0.727181985	0.38127
STARD9	0.599606707	0.026844	0.170661274	0.5302	0.111389452	0.680917
ATPIF1	-0.279635205	0.026853	-0.229762673	0.06813	-0.240106542	0.056344
TMEM254	-0.33553297	0.026868	-0.205628382	0.16987	-0.3233838	0.031813
JUNB	0.749512724	0.026889	0.597254907	0.07778	0.038190487	0.910316
MT-ND5	0.660900194	0.026985	0.412009474	0.16797	0.333121286	0.264958
MTX3	-0.329004078	0.026971	-0.395349756	0.007	-0.436519331	0.002969
SYS1	0.279020771	0.026981	0.253132917	0.04346	0.206437138	0.099481
AJAP1	1.270295321	0.027023	0.71671688	0.21656	0.402150045	0.492306
DNAAF2	-0.316012161	0.027073	-0.042928468	0.7578	0.002654369	0.984709
PIN1	0.259557435	0.027069	0.150860565	0.19808	0.079661626	0.495974
P11-574K11.	0.761969058	0.027089	-0.951269094	0.00615	-0.144693288	0.672904
APOA2	1.917612886	0.027098	0.054929403	0.94971	0.9990972	0.250104

DPF2	0.263112408	0.027105	-0.017267615	0.88499	0.159860838	0.178601
C1orf226	0.603327214	0.027146	0.31875498	0.24358	0.010621928	0.969155
ZBTB14	0.918879598	0.027149	1.075598391	0.00922	0.822243042	0.049574
DHX36	-0.301866043	0.027177	0.110785931	0.4145	-0.132833335	0.327713
ST6GAL2	0.711004316	0.027174	-0.25216236	0.43439	0.516917134	0.108054
DYX1C1	-0.416793281	0.02721	-0.334260724	0.06956	-0.435599794	0.018446
SERPINE2	0.71769471	0.027207	1.032163311	0.00147	0.117676979	0.717479
PDE9A	0.347718113	0.027255	0.176444968	0.26205	0.253273849	0.106902
ERH	-0.264203793	0.027276	0.107933144	0.36518	0.012697933	0.915154
FSTL1	0.585317092	0.027285	0.684923646	0.00978	0.306703664	0.247388
ANXA3	0.269361117	0.027395	0.375819891	0.00203	0.505489016	3.20E-05
PRIM2	-1.089452517	0.027392	0.283057321	0.55545	-0.031527676	0.948021
RPSA	-0.262132483	0.027423	-0.15643405	0.18793	-0.123436899	0.298771
XNC01-221I	-0.709026209	0.027443	-0.013464241	0.96429	-0.157942139	0.601379
DTD1	-0.33589927	0.027479	-0.219205561	0.14517	-0.107561035	0.472068
S1PR1	0.855869135	0.027573	0.63783309	0.10072	1.03659927	0.007358
CD44	0.778149116	0.027611	1.029847821	0.00354	1.302123294	0.000226
GMPR2	-0.25227008	0.027621	-0.176418038	0.12175	0.034036218	0.76442
MDC1	0.479744411	0.027622	-0.013095354	0.95215	0.292038124	0.179379
C7orf73	-0.200177489	0.027651	-0.112574347	0.20915	-0.06009811	0.500649
CDK4	-0.283430302	0.027659	-0.307996843	0.01656	-0.228771849	0.074778
STX11	1.856210084	0.027682	1.34197472	0.11947	1.810288671	0.030048
CELF2	0.543285449	0.027692	-0.482030614	0.05212	0.311136716	0.207303
TTI1	-0.224773576	0.027704	-0.172781026	0.08665	-0.069867146	0.485651
PSMD8	0.240360057	0.02774	0.124773427	0.25253	0.241272397	0.026758
NCAPD3	-0.367776063	0.027844	-0.13758942	0.40801	-0.21418667	0.197525
ABTB2	0.752596737	0.027861	0.412124006	0.2296	0.524495162	0.125127
EPCAM	-0.434255966	0.027873	-0.236575504	0.23073	-0.217000892	0.271599
RP11-343C2.	1.379456017	0.027888	1.221603188	0.0514	1.226476296	0.050286
GALK2	-0.323328922	0.02791	-0.246895524	0.09007	-0.338732069	0.019955
DLG1	0.21307437	0.027927	0.161708985	0.09434	0.282856327	0.003408
PBX2	0.177811553	0.027923	-0.0793011	0.32701	0.14641291	0.069215
DYNLL1	-0.192795164	0.027971	-0.004141514	0.96216	0.04292118	0.622543
SERPINB6	-0.307832723	0.028013	-0.348436628	0.01279	-0.229079893	0.101209
XPNPEP1	0.236367173	0.028006	-0.021075517	0.84437	0.193559251	0.071027
SCAND1	0.706981655	0.028038	0.498869698	0.12064	-0.060757477	0.851284
ZC3H7B	0.194791999	0.028047	-0.042411859	0.63272	0.105667915	0.232435
RHOBTB2	0.385010613	0.028061	-0.084121419	0.63194	0.205026829	0.242176
NDUFB4	-0.213475149	0.028084	0.074095676	0.43972	0.057115671	0.550985
TTC30A	-0.260140051	0.028158	0.046865795	0.68784	-0.176983346	0.131104
TBCD	-0.320804994	0.028223	-0.282155614	0.05293	-0.180429897	0.214927
TNS3	0.326626737	0.028223	0.033112078	0.82407	0.26131673	0.079113
EXO1	-0.563144647	0.028235	-0.377242885	0.13844	-0.25642369	0.313321
NECAP2	0.24816134	0.028263	0.230014895	0.04134	0.261450899	0.020186
SSBP3	0.358256605	0.028262	0.021423233	0.89617	0.148573577	0.363614
TBC1D15	-0.308193205	0.028251	-0.114033062	0.41258	-0.015148195	0.913119
R3HDM1	0.351935144	0.028286	-0.054118223	0.7361	0.131873135	0.411056

OCIAD1	-0.20110147	0.028351	-0.114068198	0.21054	-0.076401583	0.401447
IFNGR2	0.396390237	0.028368	0.478173505	0.00813	0.474226932	0.008671
MMP12	2.293197362	0.028382	4.146997578	4.93E-05	2.858108928	0.005635
TYMS	-0.381446305	0.028439	-0.296721585	0.08637	-0.380335776	0.027806
BIVM	-0.30388731	0.028462	-0.066644539	0.6268	-0.111565864	0.416548
CAMKK2	-0.255378028	0.02848	-0.173398515	0.13248	-0.156818492	0.173138
LETM1	0.249847351	0.028477	-0.019618909	0.86344	-0.008007503	0.943958
RLIM	-0.164274923	0.028468	0.028761839	0.69945	0.065453246	0.379211
E2F7	-0.583772499	0.02853	-0.721882474	0.00671	-0.375571115	0.157206
NUCKS1	-0.282237354	0.028577	-0.178717608	0.16548	-0.084403816	0.512363
C14orf142	-0.376224522	0.028592	-0.033397495	0.83676	-0.237559951	0.148014
GRIPAP1	0.2744577	0.028653	0.134537215	0.28137	0.165741853	0.184205
E2F4	0.184276012	0.028678	0.022256932	0.79117	0.177087754	0.034441
RASL11A	0.515740107	0.028817	-0.002803986	0.99055	0.35308362	0.132577
YY1	-0.200374425	0.028824	-0.051829548	0.57087	-0.1104278	0.227081
ZCCHC7	-0.330437216	0.028844	-0.21961399	0.14317	-0.338726026	0.024146
PCDHA4	-0.380339581	0.028882	-0.298757534	0.08402	-0.481583189	0.005554
MTO1	-0.297749667	0.028893	-0.185026494	0.17174	-0.18876841	0.163115
SUGP1	0.397885294	0.028956	0.26517873	0.14491	0.49659525	0.00614
COL1A1	0.710606799	0.028975	0.727487476	0.02537	-0.11959143	0.713255
MEGF9	-0.212539076	0.029032	-0.074594083	0.43656	0.017746388	0.852247
HCAR2	2.007801788	0.029061	0.931295057	0.31404	0.544793742	0.557148
PARG	-0.417838158	0.029056	-0.263114104	0.16565	-0.212853116	0.260193
RPL29	-0.267442939	0.02909	-0.219767461	0.07279	-0.300697247	0.0141
GREM2	0.513030386	0.029122	-0.090701593	0.70098	0.519317254	0.026877
TEAD4	0.4355615	0.029118	0.162827249	0.41564	0.072711521	0.716383
VAV2	-0.353267709	0.029135	-0.476328475	0.00323	-0.314656833	0.051084
SMOX	0.718617472	0.029273	0.793317644	0.01567	0.197344024	0.549969
.X1A-SULT1.	0.41139154	0.029315	-0.024220498	0.89866	0.13210986	0.484511
PRLR	-1.524313631	0.029361	-1.349605118	0.04651	-0.851901286	0.214495
TAOK1	0.198698613	0.029384	0.102975239	0.25845	0.115886854	0.203075
TMA16	-0.276182273	0.029397	-0.227768225	0.06857	-0.064191453	0.604036
DPEP1	1.787607877	0.029436	0.800998845	0.34773	-0.089176406	0.918922
RAB11FIP1P	0.426728842	0.029459	0.044757872	0.82075	0.21136637	0.280446
KLHL2	-0.404813418	0.029482	0.04208445	0.81905	0.14333996	0.434759
PSMD10	-0.332382817	0.029481	-0.050912809	0.73724	-0.215790016	0.155606
ZHX1	-0.214285394	0.029505	-0.169710938	0.083	0.095827186	0.325362
GNB4	0.386864142	0.029521	0.019842628	0.9112	0.333389957	0.060469
GPS2	0.273103184	0.029514	0.251461605	0.04473	0.319076276	0.010674
ARHGEF2	0.272055754	0.029569	0.11404503	0.36145	0.135516732	0.277978
LRRC37A6P	-0.772436844	0.02961	-0.328732778	0.31821	-0.372357286	0.261801
CAPZA2	-0.219514256	0.029637	-0.028766667	0.77481	-0.046940761	0.640502
CNOT6L	-0.218164989	0.029648	-0.142089376	0.15381	-0.268184828	0.007166
ING3	-0.322642408	0.029658	-0.086666343	0.55303	0.094005677	0.517403
EWSR1	0.226162474	0.029689	0.067581814	0.51579	0.353768091	0.000659
FAM126A	0.472749027	0.029813	0.468350215	0.03127	0.873757155	5.72E-05
SNRPB2	-0.211341704	0.029817	0.026175766	0.78581	0.141871279	0.138619

UMPS	-0.356875541	0.029836	-0.031302999	0.84805	-0.04976167	0.760413
SRPR	0.23757578	0.029847	0.127816782	0.24166	0.170339044	0.118783
MMAB	-0.267932092	0.029866	-0.180127951	0.1428	-0.14377873	0.240526
HMGB1	-0.23099579	0.029948	-0.025714441	0.80887	0.050897644	0.632015
RASGRF2	0.987222181	0.029951	0.371884918	0.42113	0.236827312	0.605713
AC112229.4	0.811481919	0.029992	-0.047412445	0.9024	-0.067432827	0.860352
CTCF	-0.159811132	0.029973	-0.031553227	0.66506	-0.007126583	0.921921
PPFIA1	0.248295707	0.029988	0.129015707	0.2586	0.271531192	0.017113
RAP2B	-0.228571289	0.029983	-0.121944782	0.24437	-0.14307756	0.171765
ANAPC13	-0.227321769	0.030012	-0.138700508	0.181	0.071740763	0.485701
CLIC2	-0.681040368	0.030028	0.159540812	0.58681	-0.265686473	0.378921
MSL1	0.18678307	0.030066	-0.167989813	0.05083	0.168351563	0.049486
PIAS1	0.332444102	0.030071	-0.044669524	0.77076	0.273320373	0.07406
DYNLRB1	0.26516294	0.030083	-0.027304141	0.82358	0.18075364	0.13835
PNMT	-1.227739118	0.030125	-1.007519532	0.06642	-1.042746546	0.056686
CEBPZ	-0.909356123	0.030145	-0.38548857	0.35718	-0.147058059	0.725032
KIAA1244	-0.40170721	0.030188	-0.104807154	0.571	-0.339421418	0.066696
IFRD2	-0.313067069	0.030197	-0.090325231	0.52875	-0.194838635	0.175252
MEIS2	0.330098588	0.030248	0.040307307	0.79139	0.48522311	0.001425
ANG	-0.444387419	0.03031	-0.128381363	0.52886	-0.478653455	0.019302
DCHS1	0.583264544	0.03032	0.020558779	0.93919	0.104261418	0.698755
MTMR12	0.289481736	0.030349	0.430337517	0.00125	0.593539434	7.97E-06
NELL1	-1.366419864	0.030346	-0.464078426	0.46175	-2.155176929	0.000656
STX2	0.485817672	0.030351	0.265594856	0.23546	0.686965425	0.002065
STX12	0.244586276	0.030369	0.186750734	0.09726	0.203216266	0.070713
PARM1	0.616092913	0.030403	-0.127853537	0.65347	-0.073820156	0.795442
PTK2B	0.497718865	0.030396	0.626526555	0.00635	0.415260142	0.07105
TAOK2	0.214428125	0.03039	0.13735364	0.16444	-0.00066396	0.994652
DENND2A	0.59561931	0.030446	0.267204352	0.33313	0.440167617	0.109665
PEX26	0.369999937	0.030445	0.170334171	0.31891	-0.080278569	0.638518
SEC22B	-0.192866313	0.03043	-0.004045412	0.96356	-0.08484833	0.338223
FGA	1.490227778	0.030479	0.294370234	0.66974	2.764950732	5.82E-05
KMO	0.840022275	0.030469	1.376631725	0.00035	0.85708182	0.02642
RAI2	0.89923263	0.030474	-0.40010279	0.36568	0.456656738	0.273785
FLYWCH1	0.532554206	0.030506	0.399524534	0.1051	0.145024503	0.557452
SERTAD1	0.443617504	0.030515	0.523951988	0.00991	0.372219966	0.06782
TRIM56	0.242519216	0.030531	0.094498899	0.39895	0.198505761	0.076447
PRPSAP2	-0.297162107	0.030557	-0.157249166	0.2475	-0.080623084	0.552072
AGA	-0.437430481	0.030591	-0.028035765	0.88829	0.023651821	0.905537
KIAA0907	0.310680071	0.030588	0.189359201	0.18695	0.348917509	0.014701
STXBP1	0.885458205	0.030577	0.327469902	0.42645	0.354658622	0.388579
EFCAB13	-0.531553541	0.030608	0.092927067	0.70245	-0.069769439	0.77441
NEGR1	0.658863559	0.030679	0.004765362	0.98756	-0.036888759	0.903845
PRR16	1.117580001	0.030661	1.146093029	0.02631	0.678039684	0.191915
SURF4	0.220813409	0.03065	0.15458561	0.12934	0.103668066	0.309053
TRIO	0.548682421	0.030671	0.7147602	0.00486	0.431570403	0.089083
ZW10	-0.200133124	0.030676	0.089623633	0.32303	0.032516777	0.718976

CYP2R1	-0.420427344	0.030807	-0.296752034	0.12454	-0.271413206	0.160321
FAM178B	1.407063942	0.030897	1.121469367	0.08674	0.149842048	0.826918
ATG2A	0.314843955	0.030943	0.262227577	0.07231	0.166584319	0.25415
SAMD14	0.514636311	0.030942	-0.173588137	0.47431	0.330082587	0.164181
PTPRG	0.4178707	0.030965	0.189702836	0.32718	0.474752336	0.014102
SNX27	0.252880442	0.03097	0.107004335	0.36154	0.082242813	0.482364
AK4	-0.37144916	0.03101	-0.158422997	0.35741	-0.378299827	0.028012
BTG2	-0.446823522	0.031007	-0.541373409	0.00894	-0.545610524	0.008405
STOX2	-0.596829971	0.031046	-0.293130483	0.28852	0.024230368	0.93
RNF31	0.381034723	0.031091	0.079641036	0.65213	0.256331404	0.14621
NSUN7	-0.412448688	0.0311	-0.129015357	0.49397	-0.487692016	0.010262
KDM2A	0.217258618	0.031159	0.069985968	0.48734	0.130874182	0.193544
MVD	0.469925986	0.031166	0.18380326	0.39972	0.201697217	0.35545
ZNF665	-0.41844538	0.031202	-0.064315389	0.73661	-0.058170283	0.759548
MAPK14	-0.214740093	0.031226	-0.09072192	0.35999	-0.160760166	0.105003
NR3C1	0.441825484	0.031228	0.396445847	0.05308	0.209490586	0.306985
PPM1N	-0.727565503	0.031221	-0.147821007	0.63895	0.039092237	0.899651
FAAH	-0.309095173	0.031247	-0.30829927	0.03035	-0.283876737	0.046216
DIAPH3	-0.355417246	0.031291	-0.142716687	0.38196	0.092071921	0.571493
SLC41A2	0.371115732	0.031285	0.874000624	3.44E-07	0.377093688	0.02822
SPAG1	0.678785668	0.031295	0.650071219	0.03832	0.270205337	0.388628
HNRNPA1P2	0.721156715	0.031319	0.440678557	0.19159	0.625550892	0.059511
TRIB1	0.384321426	0.031325	0.024809386	0.8896	0.077865678	0.662757
NCKAP1	-0.186195789	0.031374	-0.049674081	0.56502	-0.10403681	0.228131
AFF2	0.995836535	0.031394	1.331256811	0.00385	0.505595776	0.275747
POLR2L	0.303173465	0.031411	0.151710308	0.28138	-0.081291697	0.56523
SLC6A19	-1.083725052	0.031476	-1.44641856	0.00422	0.396094999	0.416574
STXBP5	0.337447106	0.03149	0.520582391	0.00088	0.576400916	0.000227
ATXN7L3B	-0.226643247	0.031544	-0.077844505	0.45893	-0.082000261	0.435158
GPBAR1	0.697732023	0.031549	-0.117170658	0.72172	0.54012175	0.095682
HAS2	1.128769799	0.031517	1.703113743	0.00116	0.628213604	0.231579
RGPD5	0.581968349	0.031557	0.118059609	0.66324	0.227200366	0.401161
SEC31B	0.60718458	0.031555	-0.421364617	0.14087	0.466668262	0.098602
SPON1	0.883743397	0.031545	1.084160326	0.0083	0.738312917	0.072384
UPP1	0.625342916	0.031534	0.619703922	0.03202	-0.033242842	0.909315
UQCR10	-0.284774288	0.031524	-0.197444345	0.13235	-0.332621619	0.011483
SLC25A6	0.266748716	0.031569	0.001565413	0.98993	-0.09590183	0.43953
URAD	2.472037673	0.0316	1.282657445	0.27248	0.047119951	0.969062
ZNF571	-0.324011285	0.031621	-0.71416322	3.21E-06	-0.274856637	0.064393
RANGRF	-0.32119917	0.031664	-0.257800228	0.07836	-0.329216614	0.024227
FAM65A	0.609714381	0.03171	-0.181176063	0.5298	0.071694261	0.802901
N4BP2	-0.227433572	0.031705	-0.143949488	0.17315	-0.076182662	0.469978
POLG2	-0.358638416	0.031697	-0.089030111	0.58183	-0.254856758	0.114965
SORCS2	1.127057985	0.031707	0.985197194	0.06065	0.354308178	0.502442
ANGPT4	-1.074463499	0.031754	-0.99863066	0.04545	-2.0553872	4.67E-05
PIAS2	0.222701567	0.031752	0.201452396	0.05157	0.103675638	0.316601
PRR15	-0.365479572	0.031763	-0.222352171	0.18852	-0.626802949	0.000243

OSBPL9	-0.238261198	0.031789	-0.038159488	0.73028	0.051676602	0.640211
CLPP	0.344579673	0.031813	0.262567904	0.10066	0.058546573	0.71538
BACH2	1.302994475	0.031867	1.106859569	0.06751	0.440716812	0.468935
P11-1212A22	0.528775289	0.031863	0.168638305	0.49821	-0.351577398	0.159854
RAB33A	0.883042716	0.031907	0.167980013	0.6924	0.369333566	0.376594
LARP4B	0.258891321	0.031971	0.060788655	0.61423	0.063501567	0.598098
MB21D2	-0.327819798	0.031974	-0.249735494	0.09814	-0.008927414	0.952291
ATP5D	0.77078905	0.032059	0.165232616	0.64817	0.045480708	0.900041
KMT2B	0.44225366	0.032109	-0.078981882	0.70276	0.184163571	0.373162
EPN3	-0.672966311	0.032172	-0.591463407	0.0588	-0.992304701	0.001689
ORMDL3	-0.295739981	0.03221	0.048785827	0.72195	-0.152556641	0.267257
PELI1	0.297410075	0.032213	0.477171118	0.00057	0.492444943	0.000375
RSPH9	0.512641316	0.032241	0.329516896	0.16908	0.415979566	0.07925
ZDHHC11	-0.742174061	0.032282	-0.755834051	0.02812	-0.149152451	0.659977
MT-ND4L	0.658356848	0.032323	0.398758865	0.1949	0.267480615	0.384682
MDH2	0.281562439	0.032332	-0.081143949	0.53738	-0.074266458	0.572111
EFEMP2	0.401843304	0.032395	0.187581007	0.31819	0.346725482	0.064792
GPRC5C	0.357639556	0.032409	0.228328234	0.17194	0.225777529	0.176609
VPS9D1	0.498622477	0.032425	0.013551967	0.95382	0.106797128	0.652213
BRMS1	-0.239224713	0.032444	-0.053279086	0.62649	0.038861611	0.721433
2-Mar	-0.477915767	0.03244	-0.470069451	0.03474	-0.703548063	0.001824
KIF18A	-0.288967879	0.032466	-0.030800035	0.81778	0.146642391	0.270071
MTMR9	0.256471796	0.032478	0.180252076	0.13117	0.421494666	0.000391
PCDH18	0.464897778	0.032518	0.114243957	0.59935	0.698517548	0.001312
VASN	0.638546056	0.03252	0.280450096	0.34921	-0.161418076	0.593146
ZXDC	0.214998619	0.032525	0.192152299	0.05375	0.124245495	0.212536
BOK	0.575632554	0.032557	0.354788339	0.18788	0.1729571	0.521649
ACTR2	0.156851566	0.032591	0.269001244	0.00024	0.113346725	0.121614
ELL2	0.420198962	0.032586	0.006692727	0.97284	0.485910036	0.013309
NSFL1C	0.203086876	0.032583	0.275605783	0.00364	0.253136074	0.00749
SYNDIG1	1.24284013	0.032594	1.315546302	0.02368	1.158483873	0.04521
GINM1	-0.20455235	0.032612	-0.053974885	0.56593	-0.148158437	0.11591
ACPP	0.518396166	0.032628	0.48034721	0.04678	0.504425322	0.036429
GRB10	0.482366494	0.032665	0.670882422	0.00292	0.386786583	0.086593
GRAMD1C	-0.566072913	0.032694	0.077026182	0.75838	-0.569062505	0.033313
SLC22A18	0.682008162	0.032699	0.007373866	0.98167	-0.096378112	0.763968
PDPK1	0.292743248	0.03272	0.445699552	0.00112	0.40750704	0.002895
DDX47	-0.192048376	0.032762	-0.109155265	0.22147	0.004556971	0.959112
LRRC4B	0.851322057	0.032756	-0.039577631	0.92184	0.156810299	0.695659
TXNL4A	-0.238140129	0.032755	0.003935517	0.97154	-0.045660612	0.679086
SLC25A13	-0.270406846	0.032775	-0.18377263	0.14544	-0.202806483	0.107833
FAHD2B	-0.565060924	0.03283	-0.362327149	0.16194	-0.211193192	0.41292
SERPINE1	1.138515117	0.032833	1.514894999	0.00446	0.51086323	0.338965
TM4SF1	-0.39957549	0.032844	-0.181898832	0.3311	-0.250504171	0.18075
ATG9B	1.049784956	0.032876	-0.761562884	0.14759	0.52796487	0.286682
RECQL5	0.298412567	0.032894	-0.04083862	0.76833	0.266682439	0.054533
TNFRSF12A	0.466846876	0.032911	0.498705956	0.02232	0.240805842	0.27059

ARRDC3	0.672337292	0.032988	0.720917847	0.02222	0.631350719	0.045233
SENP2	-0.334786615	0.032993	-0.036359295	0.81645	0.155460264	0.320125
TCF12	0.296343206	0.033008	-0.09927464	0.47511	0.164538677	0.23592
FAM73A	-0.251755999	0.033054	0.085837038	0.46038	0.043840704	0.706339
STK11IP	0.324785285	0.03305	0.325071324	0.03136	0.132015639	0.383017
DPM1	-0.182101836	0.033119	-0.012170589	0.88451	-0.026060168	0.755312
PCYOX1L	-0.329282517	0.033137	-0.447105488	0.00367	-0.293930285	0.054457
RP11-423H2.	-0.332321456	0.033127	-0.114471244	0.45134	-0.274258581	0.072659
TUBA1C	0.331718225	0.033129	0.369217868	0.01768	0.492454603	0.001551
CAPRIN1	0.14940367	0.033178	0.150257682	0.03193	0.188523395	0.007086
IRF7	0.737200753	0.0332	0.060027698	0.86424	0.095401184	0.788022
NCKIPSD	0.406026829	0.033205	0.212683609	0.26432	0.250668018	0.1889
ADAM28	-0.431919966	0.033257	-0.0213648	0.91605	-0.488744454	0.015949
KIAA0825	-0.395760132	0.03325	0.106222122	0.55491	-0.156304801	0.390712
P11-551L14	1.043491794	0.033289	0.067055342	0.89198	1.008779716	0.039405
ST6GALNAC	-0.38656903	0.033287	-0.476196375	0.00861	-0.658377087	0.000282
COBL	-0.488676094	0.033311	-0.602835335	0.00864	-0.313453674	0.170672
CNKSR2	1.436616351	0.033344	1.890441386	0.00448	1.037680704	0.123671
SSXP10	-0.552693535	0.033393	-0.78887489	0.00241	-0.615809049	0.017472
FDFT1	0.313555762	0.033405	0.116508558	0.42933	0.20107257	0.172415
DNAJC25	-0.331549303	0.033433	0.00049633	0.99741	-0.096117299	0.53043
YS1-DBNDD	2.855572198	0.033445	0.428748943	0.76834	2.650316237	0.049991
INPPL1	0.25760045	0.033463	0.19550775	0.10638	0.308036109	0.01092
DNAJC2	-0.241618738	0.033474	-0.120990713	0.28346	0.13774183	0.218994
CREB3	0.217399935	0.03353	0.392160479	0.0001	0.290694299	0.004068
INSL6	0.987939572	0.033545	1.084361727	0.01834	0.386570161	0.414801
LTBP3	0.498719409	0.033536	0.284362442	0.22568	0.247502801	0.292667
VRK3	0.256050007	0.033548	0.146305682	0.22322	0.22278728	0.06248
ZC3H4	0.374035758	0.033537	0.118032787	0.50231	0.049230663	0.779675
ADRA2C	-0.893056118	0.03364	0.042925524	0.91657	-1.152375666	0.006131
ZMYND8	0.37023926	0.033647	0.190302444	0.27507	0.277602399	0.111212
ELP3	-0.290072725	0.033659	-0.105089544	0.43852	-0.139977528	0.302076
DMWD	0.301931668	0.033679	0.093730909	0.51234	0.117354912	0.411499
OST4	0.274338435	0.033705	0.214244825	0.09683	0.179633361	0.163844
MRPS34	-0.326487441	0.033726	-0.192169563	0.20655	-0.481159321	0.001699
ZNF239	-0.386985114	0.033733	-0.072514296	0.684	0.004174264	0.981183
RIOK2	-0.240145832	0.033745	-0.08770752	0.43173	0.040930118	0.712737
APOBEC3A	1.565386932	0.033779	1.177981145	0.11318	1.541649918	0.03518
PALM3	-0.691211544	0.03379	-0.955778486	0.00347	-1.050695161	0.001566
SYT11	0.598331734	0.033785	0.480417812	0.08795	0.571970226	0.041745
CCDC169	-0.567580326	0.033813	-0.266887669	0.3133	-0.963729574	0.000341
FOXG1	2.302096281	0.033876	1.34069446	0.22084	0.04375602	0.969037
PCMTD1	-0.232155381	0.033903	-0.051047643	0.6389	-0.137995699	0.204877
VIL1	-0.535612708	0.033953	-0.500381435	0.04752	-0.579798807	0.021663
TCEAL8	-0.178967447	0.034067	0.094908409	0.25542	0.097179687	0.243701
ERCC4	-0.369519562	0.034115	-0.436006354	0.01212	-0.064989951	0.70765
CTSH	-0.423799834	0.034142	-0.298223822	0.13545	-0.509165007	0.010849

RBM22P2	0.960321298	0.034182	0.302151955	0.51377	0.118352933	0.799381
TAS2R14	-0.682483921	0.03425	-0.088291896	0.77389	-0.419062511	0.179037
HSD11B2	-0.838291707	0.034264	-0.486131799	0.21708	-1.279264394	0.001418
RP11-113I24.	-0.497177373	0.034292	-0.284293407	0.21067	-0.535772814	0.020209
APRT	-0.344831634	0.034324	-0.117476589	0.46793	-0.394539423	0.015201
POLDIP3	0.173741034	0.034328	0.018952032	0.81667	0.108887404	0.181765
CCDC88C	-0.429141826	0.034414	-0.171864557	0.39618	-0.293196402	0.147619
CDIP1	0.502153963	0.034406	0.295861415	0.2125	0.4689768	0.04711
GALNT6	-0.654819384	0.034413	-0.214756941	0.48663	-1.143054161	0.000223
PTMS	0.356903604	0.03439	-0.203724035	0.22851	0.105376937	0.532326
TMEM9	-0.21322697	0.034467	-0.410269865	4.80E-05	-0.227343198	0.023095
PDCL	-0.242489697	0.034479	-0.198475546	0.07921	0.09811473	0.382601
SLC45A3	-0.500391042	0.034539	-0.265402162	0.2574	-0.92330489	0.000102
SEC23B	0.27579906	0.034593	0.266972675	0.04029	0.224613855	0.084458
MPRIP	0.281524708	0.034622	0.277127687	0.03741	0.213421265	0.108921
PRKAB2	-0.357272386	0.034678	-0.499367764	0.00311	-0.118741851	0.480648
MAPK6	0.179245881	0.034689	0.13260888	0.11771	0.403148671	1.78E-06
GNAS	0.165880727	0.034732	-0.048827065	0.5343	0.126667326	0.106704
IL7R	0.64113924	0.034737	0.462747519	0.12674	0.030427374	0.920599
ITGA2	0.592737368	0.034725	0.801477046	0.00428	0.268499116	0.338881
SYTL2	-0.470965432	0.03474	-0.115194307	0.60494	-0.468877239	0.035406
TACC2	0.419200909	0.034801	-0.028135869	0.88749	0.00159804	0.993582
KLRF2	-1.274043134	0.034832	-1.168817492	0.04882	-0.920169308	0.115005
KIAA1143	-0.337101072	0.034841	-0.162021783	0.30818	-0.143781059	0.36508
KDM1A	-0.157019322	0.034859	-0.059190845	0.42386	0.077062148	0.297008
NDE1	0.303063737	0.034954	0.250515011	0.0806	0.159310972	0.265766
NSMAF	-0.243128242	0.034976	-0.041558116	0.7153	-0.113096407	0.320823
GUCY1A3	0.751751127	0.034988	0.461519714	0.19553	0.70601581	0.047613
CHPT1	-0.440791023	0.035003	-0.416129737	0.04613	-0.427355226	0.04031
ASAH2	0.801589877	0.035026	0.275654815	0.46908	0.092383987	0.808164
PIGR	-1.940608163	0.035114	-0.996858981	0.27878	-1.902630732	0.038826
SCMH1	0.262199151	0.03513	0.003235472	0.97936	0.047608723	0.701164
SLC8A3	0.720805548	0.035182	-0.12964182	0.70699	0.741192205	0.029952
KDM5C	0.17195814	0.03523	0.046875909	0.5658	0.088867114	0.276159
RAB3C	-1.433580848	0.035221	0.669502756	0.31518	-1.179557368	0.082449
RPS3AP44	-0.878834093	0.035208	0.037534595	0.92165	-0.286409133	0.46064
ZNF621	0.246218443	0.035219	-0.009695455	0.9338	0.184370529	0.112409
NISCH	0.293465539	0.035247	-0.034796012	0.80322	0.163714308	0.240208
KIF13A	-0.264633218	0.035268	-0.176998028	0.15767	-0.133719125	0.284825
PBXIP1	-0.358231394	0.035288	-0.161753113	0.33973	-0.43339228	0.010703
PCP4L1	1.254249431	0.035283	0.114549842	0.85452	0.692077514	0.251603
KMT2A	0.358477582	0.035323	-0.008071284	0.96224	0.189086428	0.266797
C12orf68	0.967935817	0.035346	-0.059242193	0.90085	0.323797183	0.487491
SEL1L3	-0.392337101	0.035372	-0.284660357	0.12637	-0.12585799	0.499023
TMUB1	0.376187757	0.035364	-0.032034031	0.85839	-0.183930876	0.306742
ASIC2	-1.187156574	0.035385	-2.3799794	8.50E-05	-0.807118567	0.143055
SYNE2	-0.404550738	0.035393	-0.243564965	0.20495	-0.413316724	0.031505

IDO2	0.743184169	0.035443	0.480450083	0.1769	0.717161517	0.039494
TYW5	-0.196216376	0.035452	-0.053913898	0.55725	0.015489962	0.865128
CTC1	0.412019819	0.035465	0.266620703	0.17132	0.406149208	0.036837
EXOSC7	-0.302658051	0.035506	-0.244909455	0.08556	-0.166304621	0.241849
PAICS	-0.290384975	0.035583	-0.307530335	0.02595	-0.250588381	0.069495
CDC25B	0.476519917	0.035635	0.061737294	0.78563	0.126819313	0.576328
YPEL5	0.256116058	0.035628	0.26711486	0.02762	0.368856806	0.00231
RILPL2	0.334924216	0.035649	0.253954655	0.10997	0.170913061	0.282518
CDKN2B	0.733720187	0.035686	1.049458312	0.00259	0.317857	0.362787
COQ5	-0.205577405	0.035734	-0.090904317	0.34006	-0.113277026	0.236906
TOM1L1	-0.332947768	0.035741	-0.238675241	0.12946	-0.238506911	0.129996
RNASE1	-0.703562932	0.035799	-0.350938049	0.29437	-1.428804145	2.08E-05
CCDC43	-0.238129908	0.035851	-0.145050884	0.19671	-0.046152335	0.679936
SCARF1	0.734730805	0.035941	0.309453861	0.3851	0.398886109	0.273353
ANXA1	0.611817696	0.035981	0.121955903	0.676	0.609299702	0.036712
ZNF451	-0.243481254	0.036004	-0.182521471	0.11485	-0.062920996	0.586496
TSTD1	-0.346195429	0.036014	-0.016481162	0.9181	-0.329645187	0.042502
NR5A2	0.521170017	0.036077	0.446052278	0.07255	0.480933757	0.05282
DDB2	-0.536056894	0.036094	-0.275316425	0.27884	-0.714696162	0.005191
NR4A3	1.768552411	0.036167	1.76604486	0.03599	1.144378905	0.177249
PNRC2	-0.146769246	0.036179	-0.103900981	0.13693	0.015193946	0.827377
CNN2P1	0.901058935	0.036208	-0.010902753	0.98097	-0.283312545	0.543767
UPK1B	-0.530575267	0.036242	-0.437995637	0.08376	-0.141274178	0.576908
BLM	-0.465904302	0.036263	-0.412679642	0.0628	-0.043673875	0.843677
TATDN3	-0.274591652	0.036257	-0.095280995	0.44731	0.023007466	0.85331
BPIFB1	-1.36242821	0.036307	-1.048694649	0.10689	-1.856531977	0.004346
SLC2A3	0.613260816	0.036405	0.078736952	0.78828	-0.066368372	0.820943
LNPEP	-0.178479344	0.036437	-0.267194693	0.0017	-0.168672213	0.047303
SEPSECS	-0.364139786	0.036444	-0.02844164	0.86866	-0.442264467	0.010323
METAP1D	-0.548396989	0.036536	-0.380709543	0.13806	-0.385611982	0.133272
CHCHD7	-0.251822532	0.036569	-0.119037118	0.31857	-0.024506304	0.836621
ABCG2	1.064381895	0.036637	0.852933238	0.09184	1.546546869	0.002149
MTRNR2L1	-0.561685876	0.036649	0.150446362	0.57441	0.039183355	0.883737
SLC17A7	0.894995684	0.036665	0.453827766	0.29746	0.308280404	0.481125
YBX2	0.797974491	0.036662	0.202188906	0.6009	0.623644849	0.10285
LYPLA2	0.206035833	0.036822	0.035446969	0.7186	0.116150733	0.236838
PLEC	0.445008881	0.036844	0.400494724	0.0603	0.049852304	0.815199
TMEM200C	0.781305676	0.036891	0.679757026	0.06889	0.084142767	0.822294
PPP1R13B	0.336846215	0.036917	0.126419748	0.4333	0.019886094	0.901822
PKP3	-0.335018701	0.036978	-0.062769773	0.69492	-0.199982144	0.212705
GTPBP10	-0.283864826	0.037094	-0.127597191	0.34335	-0.104256618	0.438514
CD320	-0.446948153	0.037132	-0.397351044	0.06246	-0.580925659	0.006607
ZDHHC9	0.247791429	0.037172	-0.085922679	0.47105	-0.136089319	0.254096
TBL1X	0.257619635	0.037194	0.050035863	0.68593	0.043916479	0.722329
NUDT8	-0.579918507	0.037237	-0.158495901	0.55701	-0.821186547	0.002909
RHOC	0.273371954	0.037229	0.227697323	0.08247	0.198173	0.130732
HIST1H1E	-0.341994963	0.037249	-0.129197816	0.43077	-0.042491998	0.795472

HDAC8	-0.420545266	0.037261	-0.194490648	0.33155	-0.04818393	0.808957
FOXK1	0.33219026	0.037329	0.04027492	0.80077	-0.065233133	0.682761
DTX2P1	0.560779024	0.037371	0.344186365	0.20201	0.382046703	0.155185
LCT	1.811890433	0.037396	1.131694209	0.19714	0.630319724	0.476891
HEPACAM	-0.924226817	0.037408	0.220052957	0.60023	-0.381998918	0.374063
VDAC1	-0.186441082	0.037423	0.033189734	0.71048	-0.028976222	0.745945
FUBP1	-0.160420857	0.037488	-0.1662754	0.03063	0.14984374	0.050585
GDI2	-0.158361605	0.037487	-0.041356658	0.58597	0.047243652	0.53359
PHLDB1	0.451842214	0.037513	0.462854736	0.03295	0.156669256	0.470777
MTND4P23	1.373261372	0.037633	1.672857673	0.00907	1.867059493	0.003154
NAB2	0.444392799	0.037685	0.206583158	0.33406	0.318498286	0.136049
SEZ6	1.214135458	0.037688	1.117268071	0.05668	0.549005763	0.34327
ABCB7	-0.345298774	0.037705	-0.106098204	0.51884	-0.149682795	0.361733
TMEM200B	0.699308002	0.037773	0.120465809	0.72282	0.580121333	0.084179
RAVER2	-0.227485993	0.037809	-0.061245195	0.57303	-0.218938763	0.044363
EIF3H	-0.395696161	0.037878	-0.26297629	0.16622	-0.270291108	0.154031
C15orf57	0.199370628	0.037921	0.227369615	0.01733	0.109981334	0.25025
CCDC174	-0.287635006	0.037909	-0.289595026	0.03486	0.050771268	0.709276
MFN1	-0.209070825	0.037918	-0.032030215	0.74852	0.024356826	0.807267
SLC15A2	-0.756284405	0.037909	-0.060515172	0.86521	-0.870567749	0.015966
SNAPC4	0.342992759	0.037956	0.168500018	0.30783	0.159285608	0.334023
PSMD12	0.227596348	0.037986	0.192445136	0.07838	0.333679364	0.002226
NCAPG	-0.386070323	0.038002	-0.104827815	0.57164	0.141925196	0.443245
BTF3L4P2	-0.339716613	0.03803	-0.119326856	0.45707	-0.05207214	0.744215
POC1A	-0.395369941	0.038034	-0.116423386	0.53259	-0.188517811	0.312362
CDKN1C	-0.392745717	0.038066	-0.796987626	2.74E-05	-0.740199644	9.59E-05
KCNK5	-0.502927609	0.038058	0.012240755	0.95954	-0.980677708	5.45E-05
PPA1	-0.228329776	0.03805	-0.000874017	0.99364	0.039874635	0.715853
SNRPG	-0.249034747	0.038115	0.061020214	0.60798	0.094777039	0.425233
VMAC	-0.440837201	0.038114	-0.384316343	0.0651	-0.334110494	0.105563
ANKK1	1.513706537	0.038172	0.991618073	0.16947	0.988566669	0.173764
P11-632K20	-0.428239664	0.038175	-0.028536882	0.88898	-0.410643825	0.045887
DOCK5	0.465904919	0.038186	0.223164139	0.32073	0.373356303	0.096428
MOB2	1.019672165	0.038194	0.488593136	0.32256	0.89539233	0.068219
HEATR5A	0.355525686	0.038215	0.738721557	1.54E-05	0.354147751	0.038562
KLHL21	-0.384549235	0.038286	0.105220609	0.56645	-0.266501149	0.147337
AGTR1	-0.686676276	0.038302	-0.57222995	0.08422	-0.55331245	0.094989
CAP2	0.584851729	0.038307	-0.445850834	0.13304	0.575202765	0.038926
ANKRD37	-1.046962644	0.038381	-1.813739991	0.00034	-0.916356004	0.069392
FAM163A	0.980655953	0.03839	0.358460311	0.45918	0.273379845	0.572962
SLC7A7	0.528117517	0.03838	0.594369301	0.01963	0.245146149	0.336974
APITD1	-0.363104988	0.038413	-0.009308617	0.95605	-0.253553871	0.138999
C20orf27	-0.364699713	0.03845	-0.3579589	0.04029	-0.789119076	9.28E-06
GCSHP5	-0.934503932	0.038518	0.011677708	0.97856	-0.813913856	0.067287
LCORL	-0.294845607	0.038512	-0.115975262	0.41224	-0.152784696	0.279072
TSNARE1	0.433919678	0.038522	0.071199456	0.73218	-0.046218761	0.826703
CPHL1P	0.694806976	0.038602	1.200350415	0.00032	0.25720304	0.444158

LPP	0.619130966	0.038676	0.080348536	0.78871	0.330123062	0.26991
C2CD2L	0.478711262	0.038767	0.243280371	0.29423	-0.010513051	0.963745
CXCL17	-1.297611816	0.038774	-0.341621213	0.58143	-1.820095728	0.003924
SUCLG1	-0.278555386	0.038759	-0.076308541	0.56914	-0.205100278	0.126135
ZNF205	-0.7188951	0.038764	-0.079781843	0.81637	-0.043999939	0.898214
ATP11A	0.4014912	0.038792	0.36124443	0.06311	0.45885781	0.018145
RHPN1	-0.551818557	0.038799	-0.286289551	0.27834	-0.805858256	0.002486
NPC1	0.252009442	0.038811	0.110757464	0.36348	0.149846645	0.21765
APOL2	0.567701273	0.038822	0.692277374	0.01159	0.686080344	0.012301
APEX2	0.240976768	0.038858	0.196962494	0.09017	0.289821559	0.012165
MRPS35	-0.274361536	0.03886	-0.123988325	0.34833	-0.12662256	0.338021
EVI5L	0.44096746	0.038927	0.290650509	0.17376	0.278884037	0.191499
PRKAG1	-0.171791315	0.03897	0.006593817	0.93595	-0.063920308	0.43578
NDUFAF4	-0.278784725	0.039002	-0.3141672	0.01927	-0.12327765	0.35017
NRARP	0.710515916	0.038992	0.899536935	0.00816	0.243155524	0.483881
ACSF2	-0.25686606	0.039049	-0.420657254	0.0007	-0.274571468	0.026054
CEP128	-0.45998018	0.039042	-0.36527476	0.09967	-0.146120764	0.506686
TPTE2P6	0.8355159	0.039041	0.4740786	0.24767	0.484449485	0.235589
CEP192	-0.206165744	0.039072	0.051792893	0.60076	-0.010920887	0.912164
VKORC1L1	-0.262311886	0.039076	-0.080800901	0.52325	-0.047464812	0.707655
RRS1	-0.424390632	0.039105	-0.207648526	0.30656	-0.37370244	0.066645
KLRK1	0.671845046	0.03912	0.760349431	0.01839	0.543507408	0.093602
MAPT	0.98496158	0.039206	0.813475937	0.08765	1.001618102	0.033924
SNX15	0.384600831	0.039226	0.287529371	0.1231	0.0018565	0.992051
KITLG	-0.380045129	0.039267	-0.346800066	0.05963	0.209007354	0.255166
FANCG	-0.403990098	0.0393	-0.154953114	0.42501	-0.144976625	0.454646
ATP6V0E1P[illegible]	1.17882517	0.039386	0.975206638	0.08887	0.35448851	0.551469
[illegible]TC-479C5.1	0.566872156	0.039394	0.130904532	0.63698	0.220536456	0.421292
FUZ	-0.47110288	0.039387	-0.067329074	0.7637	-0.141650898	0.532876
GSTM3	-0.288897165	0.039384	0.132334507	0.34239	-0.119112378	0.393515
RNF166	0.54375438	0.039374	0.512582353	0.05222	0.070854656	0.788724
CRAT	0.340748094	0.039409	0.110395574	0.50545	-0.08221276	0.620762
LGALS3BP	-0.331468295	0.03942	-0.347402024	0.03082	-0.632163478	8.56E-05
HOXB8	1.127575876	0.039472	-0.325029819	0.58688	0.388439928	0.485037
LAMC3	1.176313254	0.039484	0.569577687	0.31913	0.254943271	0.656488
CYP11A1	-1.542803279	0.039585	-0.370082257	0.5823	-0.178559748	0.790554
KCTD19	-1.570765556	0.03962	-0.368745693	0.62027	-0.591423988	0.42151
MKKS	-0.279673075	0.039668	0.026067912	0.84658	0.114039944	0.396649
ADAM12	1.422348922	0.039743	2.086880281	0.00252	0.431750085	0.53298
ARHGAP39	0.553872741	0.03973	0.250637035	0.35489	0.558168815	0.040021
DDX55	-0.262271576	0.039735	-0.131420765	0.29594	-0.026464298	0.832984
TWF1	-0.19583488	0.039724	0.145304323	0.12573	-0.096543789	0.309568
TUBGCP6	0.316754662	0.039756	0.221842262	0.14937	0.321906746	0.036459
AGTPBP1	-0.301027822	0.039809	-0.161783653	0.26619	-0.132114762	0.363517
DPP10	0.499273671	0.039804	-0.165198306	0.49831	0.495230834	0.040981
SGOL2	-0.292500948	0.039815	0.0487588	0.72882	0.027401761	0.845309
FAM117A	0.530337586	0.039866	-0.11497219	0.65856	-0.022267406	0.931585

CLTA	0.142768765	0.039885	0.21210231	0.00211	0.096069868	0.164753
POLM	0.324832479	0.039894	0.163762797	0.29674	0.05682336	0.718203
SRM	0.337678393	0.039905	0.01535572	0.92569	-0.078495743	0.633838
ASB6	0.219300322	0.039917	0.18350474	0.08127	0.173849821	0.098987
FLOT2	-0.312612826	0.039982	-0.428886852	0.00478	-0.243800312	0.108605
LDHAL6B	0.813030563	0.039992	0.488567887	0.22214	0.48941656	0.219147
UCK2	-0.299555622	0.040005	-0.143105288	0.32352	-0.28796343	0.04703
VPS41	-0.230722703	0.04	-0.161082974	0.14931	-0.223825019	0.045107
FANCD2	-0.403595804	0.040038	-0.234003467	0.23256	-0.159644405	0.414743
SKI	0.638717638	0.040058	0.545781626	0.07957	0.367008827	0.238691
DACT1	0.746817336	0.040093	0.982536616	0.00679	0.2701882	0.457975
C17orf89	-0.404502843	0.040129	-0.120419358	0.53337	-0.123181485	0.523635
PIGL	0.315096173	0.040132	-0.12330074	0.42312	-0.036019595	0.814151
PLXNB1	0.256973224	0.040119	0.064870751	0.60425	0.176070132	0.159106
CSRP2	0.382196605	0.040187	0.0710866	0.70319	0.444573121	0.016651
MAN1B1	-0.343803775	0.040191	-0.209332346	0.20977	-0.613906068	0.000246
IRGQ	0.185376839	0.040242	0.154347762	0.08676	0.283985191	0.001571
NSMCE1	-0.283073757	0.040251	-0.210842576	0.11766	-0.212403955	0.116226
PLA2G4B	0.962685111	0.040306	-0.221187023	0.63667	0.422374577	0.368233
EVA1A	0.747447253	0.04034	0.70687677	0.05242	0.665967315	0.066785
PRKCA	0.306360323	0.040383	0.215855957	0.14861	0.206756874	0.166249
RP11-61N20.	-0.748987216	0.040433	0.165960792	0.62681	0.224742109	0.508131
CMIP	0.976660255	0.040458	0.616571302	0.19668	0.31243776	0.513387
EHD2	0.537049837	0.040474	0.133174369	0.61426	-0.217601042	0.411353
PLEKHF1	0.681200713	0.040523	0.093938636	0.78548	0.231689133	0.489815
METTL18	-0.296124242	0.040541	0.014504012	0.91648	0.093878678	0.492993
CPOX	-0.309214712	0.040624	-0.115224569	0.44002	-0.015403488	0.917873
PDIA4	-0.254345675	0.040627	-0.189424544	0.12713	-0.279230386	0.024539
MCHR1	1.60937449	0.04065	1.15271695	0.14313	0.624895959	0.43657
TTC7B	0.59702214	0.040702	0.250919609	0.39011	0.29794652	0.306779
LRP5	-0.310449765	0.040762	-0.525027445	0.00054	-0.726502913	1.72E-06
KIAA1755	1.372071369	0.040852	1.790332336	0.00685	0.75288809	0.265294
MFAP2	0.377351852	0.040847	-0.011208367	0.95162	0.225610035	0.221442
CYP27A1	0.953506167	0.0409	1.219841478	0.0083	0.119215216	0.802324
IRX2	-0.83264813	0.040917	-0.856227509	0.03494	-0.991714942	0.014732
NTRK3	0.917584228	0.040888	0.706711526	0.11515	0.158433192	0.72423
PHYH	-0.323495659	0.04088	-0.045668564	0.76716	-0.383358853	0.013894
RPS4XP5	-1.222906482	0.040911	-0.440168139	0.41769	-0.548596495	0.314752
CCDC183	-0.483845309	0.04095	-0.755513988	0.0014	-0.511901324	0.030219
SMIM4	-0.544646706	0.040981	-0.596525532	0.0227	-0.544140426	0.038839
ARHGEF10L	0.361280471	0.041047	0.41117456	0.01973	0.046666849	0.792127
FMNL3	0.54527505	0.041073	0.245789166	0.3578	0.495746785	0.063461
ACIN1	0.145812165	0.041179	-0.018375739	0.79671	0.147674316	0.037756
AIM1L	0.524128708	0.041223	0.190926136	0.45838	0.744625161	0.003657
APOL1	0.810032957	0.041209	1.288724429	0.00112	0.555185052	0.161601
CDX2	0.77424004	0.041241	0.415207313	0.27409	0.458970749	0.226352
FAM115B	0.554715387	0.041197	0.140919594	0.60644	-0.042512537	0.877012

MAB21L2	0.511230707	0.041217	-0.234137125	0.35072	0.608830754	0.014972
MRPL9	0.255881854	0.041141	-0.081726247	0.51409	0.248497662	0.045975
PAN2	-0.372723045	0.041143	-0.140593621	0.43992	-0.172127519	0.344404
RAI1	0.383344215	0.041179	-0.046435306	0.80491	0.037274921	0.842921
RASA4CP	0.655726029	0.041192	0.328784047	0.3074	0.365509714	0.256562
SEL1L	-0.281278004	0.041178	-0.071944971	0.60112	-0.087683087	0.524046
SNX19P3	-1.084614365	0.041124	-1.094157305	0.03643	-1.036950394	0.045068
SNX25	0.561244053	0.041138	0.22421795	0.41481	0.413645585	0.132256
TIRAP	0.431923069	0.041242	0.176078835	0.40527	0.178342609	0.39857
ZNF815P	-0.970830719	0.04127	0.312450049	0.44419	-0.971313752	0.033824
TRMT61B	-0.461865465	0.041309	-0.404450707	0.06894	-0.153528143	0.489725
CYHR1	-0.240266079	0.041448	-0.322831747	0.00609	-0.478564538	5.50E-05
ZDHHC14	0.390549741	0.041443	0.247029926	0.19	0.186998441	0.324278
ARHGDIA	0.373806056	0.041462	0.119955576	0.51381	-0.045435869	0.80485
MST1R	0.491630371	0.041501	0.250445634	0.29926	0.400710761	0.096051
GATAD2B	0.400440814	0.041531	-0.021154348	0.9146	0.133141709	0.498173
PABPC5	0.628450916	0.041559	0.216744841	0.4829	0.329539479	0.284128
TUBGCP3	-0.335607269	0.041644	-0.511350739	0.00186	-0.273096227	0.095059
CDHR5	1.241722792	0.041656	0.384224741	0.52899	0.023685703	0.969075
CTSC	0.329703736	0.041696	0.226037258	0.16262	0.400657212	0.013282
RAB27B	-0.317518822	0.041707	-0.330735804	0.03385	0.187791646	0.227757
SLC39A4	1.052640147	0.041721	0.404121473	0.43546	0.520432729	0.315955
AGPAT5	-0.303240427	0.041758	0.053907941	0.71597	-0.004007528	0.978424
CDNF	-0.981245657	0.041774	-0.801771964	0.0763	-0.330803727	0.447634
GPATCH11	-0.248339364	0.041794	0.219311107	0.06109	0.205574037	0.080589
ZNF845	-0.375717326	0.041786	-0.12069701	0.50988	0.1261419	0.490281
IVD	-0.389103246	0.041811	-0.301911095	0.11212	-0.259129547	0.172747
MED27	0.221300792	0.041868	0.144379936	0.18027	0.307587638	0.003764
NCOA5	0.289478432	0.041972	0.149592197	0.29285	0.377258277	0.007704
RHOQP1	-0.918578097	0.042024	0.333770266	0.39265	0.417184086	0.280578
CRMP1	0.712941475	0.042035	0.18655035	0.59553	0.589024071	0.092829
FCF1P8	1.391504224	0.042108	0.533008843	0.45536	0.915962355	0.186556
EMILIN2	0.554832497	0.042216	0.481570751	0.0767	0.32596007	0.233222
GRIN2D	1.115194763	0.042196	0.418507186	0.45723	0.234947281	0.678616
POLR2D	-0.251721945	0.042211	-0.026739212	0.82781	-0.088728701	0.470021
ADAM17	0.220080978	0.042256	0.308021223	0.00431	0.348385877	0.001232
TMEM132C	-2.031300548	0.042263	0.189018503	0.83896	-2.534821143	0.012677
EXTL3	0.310285603	0.042288	0.041523502	0.78583	0.059665065	0.695763
SLC7A6	0.523854076	0.04232	0.098232599	0.70398	0.030241443	0.906786
COX14	-0.319603686	0.0424	-0.383319464	0.01388	-0.307675396	0.046486
ID4	0.534897195	0.042444	0.558960297	0.03359	0.833573705	0.001486
ALDH1B1	-0.307116496	0.042469	-0.296385167	0.04963	-0.141147637	0.348691
SLC6A6	-1.746975591	0.042481	-0.741239631	0.36966	-3.523606267	0.000229
CERS1	0.740863215	0.042517	0.124041973	0.73795	0.355400828	0.333327
KCNIP4	-0.493717611	0.042514	-0.324715508	0.17105	-0.099693715	0.66481
SLFN12	0.512037618	0.042502	0.200300794	0.42757	0.534464428	0.033132
ZWILCH	-0.359912867	0.042582	-0.181551303	0.30188	-0.180565484	0.304321

LATS1	0.205055686	0.042619	-0.059244131	0.55795	-0.015770449	0.875819
ALKBH6	0.377841334	0.042667	0.317653152	0.08843	0.043623403	0.814036
STRA13	-0.336936555	0.042794	-0.205925439	0.2102	-0.37464616	0.02326
TMEM41B	-0.19131726	0.042796	-0.102636571	0.27406	-0.014905637	0.873592
COLEC12	0.51748957	0.042863	0.681885732	0.00753	0.410535879	0.10794
MLIP	1.125828276	0.042837	0.505237	0.36592	-0.09683458	0.867157
RBM41	-0.340361183	0.042858	-0.183380184	0.27198	-0.073959007	0.657077
SCAPER	-0.316812299	0.042857	-0.197194673	0.20469	-0.143116852	0.355513
CKMT1B	-0.435942152	0.042891	-0.428673894	0.04509	-0.46449072	0.029968
CFL1	0.177621652	0.042924	0.084878774	0.3331	0.134214131	0.125793
CSAD	0.458156107	0.042984	-0.005585658	0.98039	0.033908612	0.88143
GSG2	-0.425131041	0.04298	-0.129302597	0.53034	-0.166980977	0.417486
MPND	-0.410532449	0.042966	-0.175926912	0.38027	-0.120829298	0.547599
ESCO1	0.193619678	0.043018	0.025223898	0.79075	-0.169265553	0.075028
SHROOM4	0.36978358	0.043034	0.175812393	0.33585	0.17329642	0.342177
STX5	0.290679384	0.043037	0.151436265	0.29125	0.153701748	0.283471
ARF6	-0.18767977	0.043122	-0.142432484	0.12357	-0.165980157	0.072626
15-Sep	-0.161128576	0.043149	0.069515231	0.37923	-0.096413487	0.223476
P11-159D12	-0.455277245	0.043163	-0.090604516	0.68041	0.284353228	0.190073
ULBP1	-0.508077066	0.043185	-0.050237461	0.8325	-0.314955413	0.191955
ZNF563	-0.530407088	0.043252	0.115522904	0.65425	-0.238486154	0.355551
ANKRD29	-0.89673124	0.043336	-0.614400668	0.15442	-0.792183833	0.068338
IL1RAPL1	0.598550458	0.043336	-0.166501717	0.58163	0.399470282	0.175723
C1GALT1	-0.312946443	0.04336	-0.015640761	0.91939	-0.149849225	0.332437
CSPG4P11	0.578002738	0.043409	-0.263942675	0.36464	0.18131169	0.527813
ICA1	-0.353171034	0.0434	-0.046756803	0.78791	-0.182897069	0.292535
DYNLL2	-0.223584775	0.043422	-0.190277385	0.08449	-0.161416647	0.142837
TTC33	-0.286961593	0.043533	-0.095329942	0.49803	0.114583511	0.413088
ZNF564	-0.360369381	0.043533	-0.168601157	0.33377	-0.388746587	0.026537
PRDM10	0.299536807	0.043575	0.148845024	0.31425	0.32018838	0.030017
STK25	0.16295337	0.043577	0.029043924	0.71849	0.138767945	0.084253
TRAPPC10	0.250147923	0.043622	0.038382674	0.7566	0.092304714	0.455271
P11-495P10.	0.813764332	0.043673	0.040720858	0.92278	0.143778197	0.728883
PROSER2	-0.488007012	0.043722	-0.666415881	0.00581	-0.464543292	0.053152
EBF4	0.420863306	0.043805	0.008988246	0.96586	0.080508292	0.700503
PON1	-1.234828158	0.043803	-0.088760071	0.88097	-0.713991144	0.239652
TBP	0.458859139	0.043812	0.085402417	0.70887	0.147945904	0.516473
TMEM50B	-0.288192538	0.043784	-0.091527744	0.51723	-0.284120602	0.044994
LTV1	-0.290681082	0.04385	0.015749077	0.9116	0.086070987	0.542653
LAMB3	0.391282905	0.043925	0.48598225	0.01231	0.148551252	0.444319
SAMD11	1.52068049	0.043996	0.886652196	0.24133	0.75713218	0.318227
CCDC124	0.374545539	0.044049	0.374077575	0.04332	-0.028409704	0.87921
COL22A1	1.114130392	0.044058	1.560129816	0.00486	0.546861062	0.323123
YIF1B	-0.301270053	0.044057	-0.054066501	0.71207	-0.5207821	0.000534
CDHR2	1.617199206	0.044107	0.612165999	0.4468	0.908471135	0.259178
ERLIN2	-0.285759058	0.044171	-0.234645317	0.09777	-0.492319391	0.000516
BNC1	1.216464577	0.044224	-0.504489725	0.41174	1.273487029	0.034809

ADD1	0.190774315	0.044259	0.152955015	0.10629	0.293158357	0.001948
CDS1	-0.300355661	0.044273	-0.137768706	0.355	-0.255554398	0.086362
GHDC	-0.448455758	0.044275	-0.335943138	0.12464	-0.16373793	0.450738
RBMS3	0.664484808	0.044264	0.704577331	0.03278	0.538266309	0.103166
BVES	0.753917597	0.044288	0.639951947	0.08755	0.47937205	0.201757
MST1L	-0.965342036	0.044311	-0.127734971	0.7892	-0.13224861	0.779081
ERO1LB	0.793360844	0.044337	0.776295277	0.04692	0.437910038	0.265812
DDIT4	-0.575218442	0.044368	-0.891257741	0.00184	-0.932832699	0.001113
TFF3	-0.562569996	0.044366	-0.028713978	0.91824	-1.118954395	6.38E-05
GLCE	0.230908083	0.044381	0.490359215	1.73E-05	0.073339782	0.522558
JAGN1	-0.217158132	0.044455	-0.040920174	0.69943	0.0554792	0.598696
POP5	-0.330868857	0.044463	-0.144095817	0.37296	-0.331980468	0.041626
BCAP31	-0.186571942	0.04453	-0.096241412	0.29722	-0.18833401	0.041464
TERT	-1.335514417	0.044562	-1.058848727	0.10973	-1.840374144	0.007239
PRSS22	0.469928671	0.044603	0.445691378	0.0565	0.315642822	0.177413
IL17B	1.066386173	0.044663	0.348643148	0.52151	1.137209852	0.030391
[illegible]P11-415I12.	2.665697168	0.044658	1.12176727	0.40761	0.874270968	0.515355
COX7B	-0.207472364	0.044722	0.128334705	0.21144	-0.022309092	0.828247
NFATC2	0.504748526	0.044718	0.258145696	0.30477	0.289288105	0.248714
BMP2K	0.27609137	0.044811	0.023498847	0.86413	0.023912931	0.861766
AFTPH	-0.261874423	0.044862	-0.086555931	0.50554	0.067237809	0.604513
NDUFV2	0.239367988	0.044878	0.47791657	5.80E-05	0.505572267	2.09E-05
PPP1R7	-0.209126062	0.044849	-0.11750732	0.25321	-0.11943778	0.246282
SHANK2	-0.439494498	0.044868	-0.170999286	0.43412	-0.496152881	0.023341
SLC51B	1.347410618	0.04487	0.752767919	0.2673	-0.563196586	0.4315
NR2F1	1.003285965	0.044982	1.383028726	0.00567	0.155217149	0.756815
ATF5	0.403997272	0.045036	-0.066854156	0.74303	0.046448773	0.818734
TMBIM4	-0.220066437	0.045033	0.232956012	0.03171	-0.18276033	0.09359
CCT6P1	-0.388015437	0.045146	-0.331599962	0.08352	-0.031028847	0.86824
SPTSSB	-0.816363834	0.045181	0.011219205	0.97781	-0.677406493	0.094416
PPIEL	0.999951833	0.045278	0.261394513	0.6178	0.664038502	0.18692
PTGES3P1	0.870520396	0.045283	0.828762447	0.05519	1.23886946	0.003558
ZNF860	-0.506161681	0.045295	-0.442630405	0.07389	-0.643309715	0.01041
MLH1	-0.296856432	0.045312	-0.007832894	0.95761	0.061307662	0.677237
CDC42	0.151392706	0.045363	0.216334748	0.00418	0.263510474	0.000482
PCDHB14	0.788150975	0.045357	-0.594484614	0.14009	-0.244279651	0.53972
C8orf48	-0.589819312	0.0454	0.062036517	0.82436	-0.437579278	0.126894
CLDN1	0.534907134	0.045462	0.357037239	0.18181	0.568824484	0.033319
KLF12	0.464509259	0.045468	-0.038527046	0.86829	0.060312825	0.794967
OTUD7A	1.680708594	0.045491	0.523959673	0.53202	0.724578712	0.391464
NUMBL	0.557620309	0.045507	0.389620702	0.1626	-0.030068579	0.914624
CA14	-0.53939479	0.045518	-0.482690273	0.07026	0.006094916	0.981247
COPS2	-0.191165741	0.045543	-0.02986597	0.75381	0.101955846	0.28292
TXLNB	-0.656541828	0.045618	-0.2255375	0.48179	-0.658006456	0.043191
CIRBP	-0.250227165	0.045689	-0.131430704	0.29272	-0.140639119	0.259913
HSD17B14	0.983604853	0.045703	0.21850131	0.67259	0.675324988	0.170223
ILDR1	0.62841596	0.045661	0.397284075	0.20936	-0.386710489	0.244166

NUP160	-0.235721964	0.0457	-0.229119367	0.05098	-0.066045303	0.572987
RAPGEF1	0.293308054	0.045679	0.156752736	0.28556	-0.004693883	0.974526
SLC41A3	-0.402619845	0.0457	-0.231139533	0.24734	-0.106065039	0.59511
USP24	-0.164458558	0.045786	-0.177235151	0.03115	-0.050048413	0.542003
ZNF720	-0.297133398	0.045788	-0.233372248	0.11398	-0.083936054	0.568948
DNAJC16	-0.497873333	0.045831	-0.229178836	0.35577	0.035607023	0.885986
CANX	-0.210654402	0.045843	-0.033968553	0.74736	-0.009337417	0.92944
AZGP1	-1.684548751	0.045892	-1.0532115	0.19183	-2.647349351	0.002624
ETFA	-0.207978243	0.045885	-0.102739773	0.32219	-0.080107844	0.439093
C20orf112	0.330726251	0.045944	0.07381707	0.6564	0.098373611	0.55273
GCAT	-0.509607175	0.046041	-0.133361873	0.59539	-0.402352677	0.110506
RBBP4	-0.185574601	0.046058	-0.176588388	0.05721	-0.034180045	0.712344
CDH16	1.684276265	0.046124	1.145394656	0.18346	-1.552983828	0.099221
CWC27	-0.255262299	0.046135	-0.116767217	0.35824	-0.077075423	0.542928
JAK2	0.451141855	0.04614	0.789518178	0.00047	0.606456883	0.007233
NPY2R	1.60631932	0.046177	1.066087496	0.19051	1.770289995	0.026535
RRP15	-0.316524728	0.046262	-0.013687512	0.9311	-0.139679493	0.376629
NXNL2	0.520829906	0.046286	0.159508894	0.54477	0.098071985	0.710412
INPP5F	-0.259422067	0.046379	-0.072050366	0.57849	-0.08602521	0.506604
RNF165	0.900333896	0.046385	0.763496255	0.09131	0.452467281	0.316641
TRIM15	1.677705016	0.046408	0.816201876	0.33477	0.799961011	0.34407
KATNA1	0.320159913	0.046467	0.209030751	0.19064	0.283731986	0.074388
STXBP3	-0.280587199	0.046516	-0.065370117	0.64046	0.16607514	0.233862
BCKDK	0.310894912	0.046549	0.302275084	0.05248	0.14683997	0.34648
BUB3	-0.197427539	0.04659	0.038548563	0.69567	-0.016878026	0.863925
FAS	-0.53209128	0.046575	-0.418318544	0.11671	-0.923446879	0.00057
URGCP	0.277553238	0.046584	0.040397186	0.77163	0.392221455	0.004632
KIAA0754	0.342278525	0.046653	0.021956601	0.89859	0.204844221	0.233327
AP5Z1	0.368267352	0.046675	0.274711781	0.13816	-0.027912136	0.880581
NPM1P37	-1.022826331	0.046673	-0.453140833	0.33851	0.160395798	0.720842
GPR146	0.811029723	0.046906	0.294770686	0.47825	0.254300355	0.541656
FAM221A	-0.570418171	0.046923	-0.683317017	0.01724	-0.447766719	0.118211
PRKD2	0.315089822	0.046947	0.034577584	0.82725	0.195023648	0.218766
MPV17	-0.307021622	0.047001	-0.236277335	0.12008	-0.255302198	0.092571
GPSM1	0.449213088	0.047036	0.011420475	0.95992	0.094801762	0.676145
C3orf58	-0.199779166	0.04706	-0.10103005	0.31278	-0.138097553	0.167627
SREK1	-0.193840809	0.047102	-0.128331874	0.18735	0.044028641	0.650357
PGF	0.63271092	0.047113	0.542618274	0.08782	0.331244431	0.299951
TMEM165	0.234689296	0.047126	0.557427037	2.15E-06	0.382729867	0.001152
ABHD15	-0.370170559	0.04715	-0.303599652	0.10052	-0.757487457	5.40E-05
FAM180B	1.054739928	0.047166	0.482313789	0.37373	-0.492052042	0.396384
PACSIN3	-0.368104858	0.047156	-0.623900285	0.00077	-0.640497729	0.000541
ZNF286A	0.240145793	0.047216	0.192174332	0.11129	0.492430366	4.11E-05
DENND1B	-0.558758028	0.047255	-0.107802025	0.69921	-0.548393998	0.049611
ADCY5	0.64320945	0.047326	-0.725746637	0.02709	0.152782224	0.637449
RPL19	-0.204090814	0.047438	-0.004700005	0.96356	-0.098253619	0.339608
GSTCD	-0.274534044	0.047474	0.006197149	0.96401	0.031374794	0.818981

HNRNPLP2	1.264764893	0.047489	-1.137443735	0.13122	0.393813324	0.548744
P11-603K19	1.117182885	0.047503	0.944886852	0.09366	0.553003843	0.336221
SLAIN2	0.353150577	0.047508	0.135364136	0.44716	0.198323128	0.265164
TUBG1	0.245900301	0.047597	0.007124564	0.95429	-1.37E-05	0.999912
LARP1B	-0.254368261	0.047609	-0.189606341	0.1382	-0.113131092	0.375147
IDE	-0.228137049	0.047623	0.031332938	0.7847	0.004732246	0.967079
SMEK1	-0.146224823	0.047637	-0.011202465	0.8784	0.019843472	0.785985
LAT	0.606888965	0.047705	-0.291036608	0.35647	-0.069022922	0.823384
NUP107	-0.227714608	0.047709	0.013167397	0.90821	0.11345351	0.319271
SERPINA4	-0.315776443	0.047723	-0.090920814	0.56761	-0.400310414	0.011996
TAS2R64P	-0.70561004	0.047713	-0.431422998	0.21295	-0.541956181	0.121036
COPS7B	0.295635137	0.047772	0.332317589	0.02553	0.251763866	0.090475
DDX10	-0.365048254	0.047965	-0.062098867	0.73569	0.036140202	0.843863
FGF11	0.498475811	0.047978	0.380045619	0.13149	-0.116359953	0.644916
LMOD1	0.636662576	0.048079	-0.385253284	0.23813	0.680566441	0.034027
CSTB	0.447211222	0.04814	0.581189323	0.01019	0.286280401	0.205879
TSKU	0.443035552	0.048139	0.025021117	0.91119	0.309410574	0.166942
ZNF782	-0.373868541	0.048139	-0.180089909	0.33628	-0.223915975	0.231327
ICOSLG	0.613793019	0.048266	0.151499064	0.62827	-0.217858073	0.484965
FFAR4	0.766418547	0.048385	0.865482106	0.02464	0.554463257	0.152953
SUPV3L1	-0.282833361	0.048389	-0.11744783	0.4091	0.006087747	0.965825
SLC44A2	-0.281597866	0.048486	-0.22774395	0.10972	-0.467639546	0.001035
OMMD3-BM	0.578777328	0.048514	0.640117031	0.02836	0.712583275	0.014462
ZNF681	-0.371376097	0.048537	-0.148662545	0.42675	-0.193025105	0.301108
HK1	0.264712514	0.048657	0.200186785	0.13575	0.244031264	0.068871
KCND3	0.722580093	0.048675	-0.003949002	0.99143	0.750154148	0.040501
MPC1	-0.228548675	0.048673	0.035576578	0.75243	-0.088569202	0.433765
ALDH1L1	0.811396481	0.048711	0.538782333	0.18968	-0.14855983	0.719424
SNRPA1	0.236147605	0.048731	0.272394169	0.02183	0.377684426	0.001373
LASP1	0.19990099	0.048813	-0.135018052	0.18353	-0.041530322	0.682296
PDE4D	-0.355325031	0.04881	-0.194392936	0.2805	-0.377539617	0.036243
P11-408P14	-0.673787226	0.048796	0.041997152	0.89461	-0.812180454	0.016442
SULF2	0.301409775	0.048801	-0.096942552	0.52661	0.227769983	0.136382
VARS	-0.282727745	0.048793	-0.138546518	0.33342	0.043964936	0.758965
CEACAM19	0.383750015	0.048848	0.029592121	0.88064	0.211202766	0.280562
GLI1	0.733845593	0.048858	0.099128618	0.79059	0.303804247	0.414964
LPAR5	0.636293459	0.048864	0.375647445	0.24708	-0.490307939	0.145551
PTCH1	0.521552532	0.04885	-0.303699451	0.2519	0.208524839	0.430819
AC007256.5	1.224816256	0.048961	0.419591762	0.51413	0.572716731	0.367101
CHRNB2	0.686668089	0.048951	-0.034357879	0.92406	0.369036772	0.292019
MOB3C	0.330778382	0.048954	0.296601297	0.07589	0.26855112	0.10791
BRICD5	0.417140136	0.049035	0.237830983	0.25665	0.098214882	0.639025
NUBP1	-0.263677817	0.049108	-0.212060576	0.10999	0.153903988	0.238772
LRMP	-1.190181174	0.049141	-0.589107579	0.29984	-0.935944278	0.115062
HLA-DRB1	1.053857302	0.049204	0.568384025	0.28902	0.125794133	0.814626
CCDC132	-0.242564329	0.049232	0.070144333	0.56466	-0.008004333	0.947678
SERPINA1	-0.628018886	0.049235	0.150032313	0.63844	-0.537559329	0.092299

CCP110	-0.258253411	0.049266	-0.01367233	0.91648	-0.09265061	0.476834
ADAT3	1.068674346	0.049294	0.341786259	0.54443	-0.217812334	0.709015
LGALS4	1.103079056	0.049307	0.063267979	0.91027	-0.022827969	0.967565
XRCC6	-0.162509716	0.049308	0.046007781	0.57684	0.121898775	0.139027
SPOCD1	0.729169825	0.049348	0.773586831	0.03685	0.188419711	0.612699
KAT2A	-0.348319725	0.049425	-0.077808981	0.65893	-0.26742698	0.129797
KCNH7	-1.025630944	0.049417	-0.608484518	0.23824	-0.851804979	0.098657
THTPA	-0.481124504	0.049546	-0.118574572	0.62234	-0.053765594	0.823408
TAX1BP1	0.193300363	0.049574	0.34408245	0.00046	0.33222425	0.000713
GNAI1	-0.264322593	0.049589	-0.036027095	0.78765	-0.163778798	0.221173
PRICKLE3	0.427555524	0.049618	0.434815116	0.04664	0.307575965	0.159761
HNRNPH3	0.322174317	0.04963	0.083503534	0.61074	0.262341758	0.109473
CCDC148	0.574512715	0.049745	1.015957209	0.00033	0.671779944	0.018925
NSUN2	-0.159221945	0.049739	-0.161416801	0.04434	-0.113071234	0.158254
PLCZ1	-1.459090181	0.049753	-0.311328912	0.64553	-0.877828115	0.209154
ZNF70	0.297338042	0.049746	0.16850383	0.26536	0.111607846	0.460726
ARL6IP4	-0.196258802	0.049787	-0.027562128	0.78106	-0.035172738	0.722593
ZNF671	-0.415650543	0.049782	0.085454097	0.67676	-0.006929114	0.973103
MAGEB10	0.793007468	0.049829	0.29262909	0.4787	0.14937987	0.719215
SLA2	0.761684443	0.049828	0.609027125	0.11661	0.524050182	0.177651
C1QTNF5	0.537309754	0.049933	-0.207880421	0.46	0.427747199	0.117381
ELP2	-0.263715561	0.049951	-0.127691087	0.34021	-0.077162632	0.564081
GSKIP	0.340926852	0.049915	0.156626443	0.36768	0.401606506	0.02044
HMHA1	0.399344049	0.049925	0.263302459	0.19658	0.128552752	0.528429
N6AMT1	-0.277208218	0.049943	-0.227646769	0.10305	-0.190354049	0.169775
SAAL1	-0.299542431	0.049935	-0.032765384	0.82724	-0.167045048	0.266395
AC004945.1	-1.652787798	0.039216	-1.120848139	0.12175	-0.81389677	0.240549
AC005884.1	-1.753598529	0.032112	-0.020042753	0.976	-0.429470603	0.529384
AC006547.14	3.068367607	0.02935	4.43976644	0.00096	3.525542203	0.00968
AC007229.3	-3.93597252	0.008781	-0.526720983	0.66204	-0.415862936	0.72766
AC015818.3	-3.834618201	0.003578	-0.726865927	0.49378	-2.849388889	0.01581
AC022210.2	3.45419932	0.026856	3.528950575	0.02296	1.915588958	0.241322
AC097721.1	-1.767371337	0.033402	-0.330749128	0.62354	-1.634313656	0.036576
AC104843.3	-3.813324075	0.011651	-0.944089821	0.45385	-0.175253042	0.883841
ACTN2	2.069292557	0.011917	0.961137607	0.24827	1.121455157	0.179637
AL353997.3	-2.784372542	0.012747	-0.248864008	0.76871	-3.764137322	0.001559
ALOX12B	2.021679203	0.018541	1.315717811	0.1462	0.515895122	0.573563
AOC4P	-2.006255677	0.021429	-0.18872009	0.81199	-0.586059835	0.461231
AP000936.1	-2.803849859	0.045263	-1.998688806	0.12141	-1.092721301	0.374851
ARHGAP36	-3.440833986	0.030953	-0.591654828	0.65916	-1.428459576	0.327992
ARPC3P1	-1.889190485	0.008775	-0.52194144	0.37716	-1.208944422	0.055227
ATP4A	2.52279383	0.032442	2.301737859	0.04986	0.970321806	0.42768
BEND3P3	3.667569739	0.042326	2.72152966	0.1377	2.588372237	0.158846
C10orf126	3.472866219	0.013971	1.731169529	0.22883	1.902583519	0.184292
C10orf53	1.979253183	0.019063	1.399364137	0.10292	0.756658354	0.393004
C10orf99	2.770215874	0.044723	0.703900511	0.63614	-1.198870221	0.476529
C12orf74	1.301542288	0.044729	0.631103408	0.34936	0.036470689	0.958618

C19orf38	-2.132653418	0.035042	0.322010619	0.63202	-1.292068826	0.118081
C1orf195	-2.745840178	0.013447	-1.741215683	0.0623	-0.326048649	0.699656
C1QTNF9B	-1.458849488	0.019437	-1.124117345	0.04951	-1.351681279	0.02056
CALHM1	-1.987662464	0.02101	0.142049211	0.8244	-0.868343606	0.214102
CALML3	4.296346404	0.041344	2.401510964	0.26089	-0.889930331	0.702255
CASQ2	3.787022531	0.008699	4.384794704	0.00182	1.000460353	0.504004
CDH20	-1.948134694	0.045898	-1.579383519	0.11242	-0.803507804	0.390282
CITED1	2.017889338	0.000898	1.233564166	0.05191	0.987743389	0.124152
CLCA2	4.406531089	0.002497	3.605659876	0.01322	2.708146806	0.069534
CLCA4	3.12445545	0.004482	1.751306245	0.11647	-0.612777601	0.625736
CLEC18C	-3.492689633	0.019695	0.497766802	0.65805	-0.612755916	0.595297
CNFN	2.037177096	0.023728	0.732166345	0.4494	0.251785818	0.802397
CTA-85E5.6	-3.283422401	0.01908	0.579190348	0.57983	0.228629481	0.829913
TD-2302A16	-3.524161684	0.010185	-0.947206433	0.39903	-1.749633775	0.158313
CTRB1	3.982999806	0.009013	2.326817051	0.15357	2.593358424	0.104556
CXCR2	2.190979044	0.031968	2.38675097	0.01794	1.589315953	0.125678
CYCSP39	3.466870356	0.012148	1.59091193	0.29141	3.5849941	0.00886
DHDH	1.150047688	0.047432	0.43690037	0.4804	0.529140672	0.372396
DMRT2	2.969267351	0.034761	3.373341492	0.01375	2.062874832	0.175261
DMRT3	4.497685853	0.003267	4.526340826	0.0028	0.832684203	0.630792
DOK7	2.111091375	0.016222	0.726185983	0.44098	0.539068629	0.569439
DSG1	-2.912915742	0.006717	-1.589162991	0.08454	-0.783911516	0.378844
DYDC2	-4.537450025	0.010667	0.185859667	0.90056	0.769944872	0.601917
EGLN1P1	4.73189834	0.030183	2.416497991	0.28658	1.364055409	0.563877
ESRRAP2	-3.423908828	0.011046	0.791501151	0.43522	0.280964718	0.786824
FAM196B	1.948344367	0.010992	1.558671917	0.04173	1.557881343	0.0443
FAM83C	1.173636849	0.04959	1.013614811	0.08969	0.542215844	0.377615
FDCSP	5.261557143	7.28E-05	4.1645376	0.00179	3.138090748	0.019842
FFAR3	3.295697374	0.034305	2.441224156	0.12904	2.786951221	0.075274
FOXI1	-4.355063201	0.011966	-1.51674568	0.31874	-3.10388541	0.068984
GABRB1	1.577894841	0.044216	0.832659615	0.3012	0.828223359	0.298073
GFI1B	1.691069851	0.010802	1.735888614	0.00762	1.646835694	0.011415
GJB5	2.751277961	0.04072	1.882795352	0.1662	-0.217558698	0.883221
GOLGA8UP	-2.944080093	0.039997	-1.419963943	0.25315	-0.91100634	0.436465
GPR20	30.60901368	1.83E-05	-0.038839249	0.9957	-0.069212438	0.992344
GPR55	5.026614853	0.000363	4.075435309	0.00461	2.375598944	0.116703
GRIA3	3.058603144	0.040524	3.707354034	0.01098	3.406262812	0.01999
GRIK3	4.075688638	0.006962	0.957490315	0.53726	4.455977711	0.002458
GSTM5	2.260310835	0.031942	1.410471263	0.18206	1.266645181	0.228908
GSTT2B	-5.552927234	0.02682	-1.175079912	0.61246	-1.839696135	0.430303
H2AFZP6	3.399959354	0.040849	0.603514853	0.74786	1.702602773	0.344881
HCAR3	2.286181012	0.012063	0.29673059	0.76597	1.610475293	0.081146
HCN2	1.275998625	0.029936	0.350826896	0.56791	0.336885632	0.582063
HEPACAM2	-1.77423063	0.028565	-0.946203974	0.17565	-2.790737803	0.001367
HES5	3.556180488	0.029122	2.941424231	0.07583	2.562766125	0.127553
HLA-J	1.349744734	0.048748	0.775542881	0.26842	0.036029463	0.960461
HMGN1P10	2.725532989	0.009639	2.336930636	0.02767	1.785413655	0.099869

HNRNPA1P4	-3.920042111	0.004182	-2.060826428	0.09512	-1.737276228	0.136255
HNRNPA1P7	1.545202818	0.040183	1.153876826	0.12956	0.790775467	0.3073
HPR	1.373392494	0.018412	0.428454944	0.49007	0.578331351	0.333024
HSD3B2	3.90613102	0.023218	3.78598504	0.02848	-0.183523634	0.925627
HSPD1P7	2.753120698	0.048674	2.401225826	0.08834	2.13254009	0.134492
IDO1	2.41374708	0.008532	3.937692641	7.23E-06	3.108917374	0.000502
IGFL3	-3.587849792	0.020753	-2.325609311	0.13167	-0.657876236	0.612097
IGJ	2.942407499	0.039847	1.955838035	0.18454	-0.500576339	0.765995
IL20	3.465170464	0.033858	0.697743987	0.70642	-0.183523673	0.921079
IL37	2.242163295	0.034639	0.125210809	0.91435	-0.437053116	0.717675
INHBC	2.936489156	0.037223	2.391654113	0.09524	2.06039908	0.156634
INSM2	-3.49319196	0.037573	-1.175501043	0.43684	-1.911265398	0.234381
KCNJ9	2.346568815	0.048785	1.36659017	0.26883	0.936060022	0.459394
KCNV2	1.462547476	0.049205	0.95616917	0.2087	0.656748103	0.396431
KIAA1239	1.637783854	0.035288	1.740956102	0.02361	0.940465713	0.237708
KL	3.407397176	0.00545	2.872608953	0.02145	2.032162178	0.120549
KLRC1	1.959149285	0.039443	1.560152841	0.10432	1.444320451	0.133832
KNG1	2.873457661	0.021524	3.218269779	0.00877	1.946727643	0.121518
KRT18P34	1.620530338	0.025086	0.911907188	0.22289	1.701661462	0.016909
KRT8P48	2.434817535	0.04963	2.740978232	0.02509	1.483705022	0.245142
LBP	5.101583709	0.000582	4.051580658	0.00717	5.74907706	9.16E-05
LDHAP5	-1.412018223	0.022869	-0.401665146	0.45753	-0.607406243	0.267327
LDHC	-1.492254879	0.014873	-0.144338551	0.79219	-1.135827912	0.048195
LILRB3	2.961286296	0.030945	3.418679586	0.01006	3.017033717	0.024507
LIPF	-4.385903967	0.007099	-2.515155719	0.07727	-2.356234807	0.092986
LINC01-131B	3.613731918	0.025864	1.31415669	0.47617	2.689545802	0.106501
MADCAM1	3.081500569	0.005647	1.198861992	0.30664	2.193759755	0.049836
MARCKSL1P	-1.461622283	0.025448	-0.531999625	0.33545	-0.644431607	0.245718
MORF4L1P1	-2.721378137	0.019301	-0.754940427	0.43732	-0.444452823	0.642683
MS4A8	-1.929474215	0.047109	-0.138374736	0.86942	-2.032383626	0.028539
MT1L	3.813658336	0.021075	2.635869151	0.11847	1.150840004	0.525042
MTCYBP3	1.680558305	0.044519	0.390402609	0.66454	0.206910283	0.82007
NAPSB	1.635031376	0.040275	0.609419236	0.45206	1.17136912	0.1449
NETO1	2.289406932	0.023613	1.548885898	0.12202	1.683464783	0.087813
NKAIN2	2.327785834	0.036938	0.775300453	0.5036	0.642163757	0.580461
NT5C3AP1	-2.033140979	0.006082	-0.254112483	0.66753	-0.394049849	0.507708
NT5CP1	3.114640522	0.034122	1.146409105	0.4828	2.073970306	0.172768
NUTM2F	4.644732756	0.005129	-0.117831387	0.95099	4.22033279	0.011215
NXPE1	2.386386585	0.011875	1.784053383	0.05805	1.840315509	0.051158
OR51E1	-2.700777627	0.027551	-4.526068514	0.00158	-4.591760801	0.001349
OR52N3P	3.039260269	0.006349	2.862343177	0.01016	4.211601233	0.000108
OR52P1P	3.134619575	0.009623	2.627491082	0.03125	3.143639832	0.009083
OR52U1P	2.874839829	0.029611	1.867022105	0.17131	3.56447056	0.005902
OR7E11P	2.72948605	0.003644	2.708993662	0.00376	3.065715504	0.000941
PCOLCE2	1.189394525	0.025531	0.57811482	0.29003	-0.642176701	0.28892
PDIA2	1.723340848	0.02909	1.016190354	0.20726	1.165300431	0.148367
PGLYRP2	5.308302049	6.83E-05	5.897555946	8.92E-06	4.469816315	0.001004

PHBP15	5.490072821	0.0045	4.048863255	0.03847	3.77289629	0.05459
PIWIL3	-2.11276606	0.013734	-0.596481866	0.36978	-0.409866656	0.531761
POMC	3.240347907	0.038047	1.845627288	0.27255	2.478218568	0.121624
PPP1R14D	2.314776929	0.006057	2.192309583	0.00921	1.822347019	0.032269
PRKRIRP9	2.072885416	0.035888	1.339087271	0.1864	0.879673203	0.39669
PROZ	1.652796984	0.017668	1.507157253	0.03132	1.449766355	0.045972
PRPH	4.447388581	0.012359	1.619845612	0.38908	4.146674376	0.01929
RBMX2P1	-2.956172876	0.033718	-1.683196984	0.22284	-1.22655721	0.334694
RP1-130L23.	1.265737417	0.040594	0.76309326	0.22763	0.40142264	0.535518
RP1-168P16.	1.31280473	0.046887	-0.017714303	0.98028	0.367182161	0.594618
RP1-278E11.	-1.531220763	0.049637	-0.869897902	0.21548	-0.366971255	0.582107
RP1-315G1.	1.345784637	0.025456	0.429660463	0.49829	0.308546332	0.628344
RP1-39G22.	1.6177624	0.024305	1.424824748	0.04761	1.384231247	0.053936
P11-1094M1	2.864669309	0.049884	2.643362612	0.07136	3.041095526	0.033019
RP11-10L12.	-1.192623512	0.032341	-0.224725539	0.64765	-0.213760517	0.66184
P11-113A11	-2.930428258	0.030824	0.488370313	0.63137	-0.418251831	0.704939
P11-1149O2	3.03799772	0.013744	2.405792077	0.05739	2.840839954	0.020988
RP11-160A9.	-1.790995257	0.049674	-1.051348739	0.1858	-0.394593202	0.591203
RP11-166O4.	2.784911684	0.036815	1.188830846	0.42431	1.475896416	0.303954
P11-288E14	-2.141838234	0.042942	-2.286692266	0.03021	-1.291775011	0.158309
P11-312J18	-2.677679081	0.044056	1.163370207	0.21497	0.808929036	0.392481
P11-345K20	1.561551105	0.028747	1.89772359	0.00627	1.602100086	0.022582
RP11-350G8.	3.133749067	0.017603	3.275516289	0.01207	3.092203808	0.018292
RP11-359M6.	-3.22518783	0.021805	0.211176043	0.84512	-1.365830883	0.279451
P11-382A20	1.722134016	0.032176	0.448354249	0.60612	1.790896024	0.023837
P11-459D22	-2.540022905	0.006039	-0.838907312	0.28134	-1.5593421	0.055108
P11-466P24	-18.13305527	9.35E-05	0.172977556	0.96968	1.777637498	0.695087
P11-486B10	-2.71189858	0.047346	-0.555125366	0.60795	-0.886230093	0.425947
RP11-589M4.	1.235045508	0.030191	-0.043823846	0.94304	0.762211947	0.186292
RP11-632F7.	-4.350596444	0.018201	1.060430463	0.49478	0.103418078	0.947474
P11-655M14.	4.702513518	0.001007	3.36120735	0.02297	3.571758847	0.014663
P11-673D15	-1.450737099	0.023782	0.284679991	0.56578	-0.02828258	0.955513
RP11-687F6.	1.363787864	0.027924	0.520877257	0.42064	0.978487933	0.117635
RP11-71H17.	-2.565535111	0.034425	-0.261693767	0.75385	-0.13425446	0.870032
P11-730A19	2.739537912	0.033254	3.422593625	0.00578	3.367373695	0.006604
RP11-777B9.	2.196441524	0.033041	1.484209959	0.1636	0.58159638	0.611976
RP11-79D8.	-4.917251506	0.000734	0.376084456	0.72515	-2.659424081	0.034492
RP11-86K22.	2.627874792	0.040232	1.243520253	0.36359	3.262556833	0.009025
P11-927P21	2.931828984	0.039629	0.883580479	0.56891	1.448718958	0.331267
RP13-672B3.	2.033569819	0.042562	0.858525113	0.42063	1.837645746	0.066382
RP5-890O15.	3.172089957	0.030195	1.939559571	0.21947	2.661455758	0.07338
RPL10AP6	2.102299402	0.020026	0.958300845	0.30673	1.232384184	0.182047
RPL22P11	-3.863607469	0.028156	-4.034235609	0.0219	-0.025630617	0.986297
RPL35P5	-3.593918519	0.017528	-0.508020057	0.68271	0.251187818	0.832862
RPL5P5	3.16941118	0.036088	1.224930331	0.46122	2.016222117	0.198792
RPL7P21	-4.560030684	0.004005	-1.782509904	0.18845	-0.583626559	0.64699
RPL7P44	-3.518940601	0.013597	-0.647693884	0.57527	-1.000219417	0.399858

RPS2P41	3.114887393	0.025021	1.141168596	0.46522	-0.183523735	0.912137
RPS3AP39	-2.995878055	0.047393	-0.115148472	0.9238	-2.594770592	0.082026
RPS3AP46	-3.335311786	0.009837	0.240015877	0.79334	-1.121431914	0.294078
RPS7P14	-2.579281729	0.003103	0.031780076	0.95477	-0.882401773	0.149675
RTL1	2.20913825	0.019253	1.75662289	0.06517	1.135128087	0.244957
RXRG	3.519473809	0.032381	3.882013191	0.01687	3.25576103	0.056172
SERBP1P6	2.24313982	0.002005	1.165480802	0.12442	1.626961088	0.027368
SERPINB4	3.957049017	0.004227	6.055013105	5.72E-06	3.189810774	0.022741
SERPINC1	3.024907684	0.018827	2.388916975	0.07088	1.756983483	0.190269
SETP20	2.755796182	0.024745	2.328738484	0.06037	2.556968404	0.036964
SLC25A41	1.15799763	0.04641	0.443911069	0.46263	0.980036172	0.090662
SLC28A1	4.126689128	0.00446	3.548892651	0.01505	2.377964543	0.118421
SNRPGP12	1.768342066	0.042976	0.684370232	0.46038	0.542292871	0.560997
SOWAHD	-1.744555268	0.044326	-2.131190343	0.01813	-0.723398906	0.326557
SOX10	2.066631881	0.00776	0.843238568	0.29308	1.366175689	0.081393
SPINK4	-3.827854218	0.0145	-2.103630481	0.12547	-2.417180196	0.079147
SPRR2D	5.06962306	6.86E-05	5.181006765	4.31E-05	4.730501134	0.000209
SRGN	3.942385187	0.02082	3.298468676	0.0543	1.325756835	0.491859
SSX2	3.724740546	0.003853	1.084682679	0.46726	1.827380973	0.188886
SSXP3	2.494609801	0.020375	2.042215827	0.0606	0.791481452	0.500964
SULT1C3	-1.452246175	0.044839	-1.670677714	0.02149	-0.834669443	0.22272
TBX20	3.513683289	0.008078	2.634410443	0.04816	2.83776798	0.033869
TBX22	-2.587565374	0.049329	-1.709631686	0.14322	-2.151440095	0.071557
TNFRSF18	3.289234977	0.004204	2.608043156	0.02549	2.243073184	0.055276
TNFRSF4	1.855486128	0.029374	1.427419806	0.10323	0.764681829	0.385128
TTC4P1	2.227753515	0.003841	1.584105554	0.04258	1.036117832	0.193381
TUBBP10	2.274252012	0.032463	0.68473347	0.54463	1.959318301	0.065984
UBQLNL	1.765692815	0.008039	1.326525267	0.04976	1.426041728	0.033258
UCN2	1.135928908	0.045251	0.198282922	0.73933	0.606436356	0.292982
WAS	1.786906198	0.049296	1.30361838	0.15808	-0.427061499	0.667154
WNT10B	4.898177422	2.06E-05	4.693518344	4.71E-05	4.721624172	4.24E-05
XKRX	1.586209275	0.017658	0.490279672	0.48579	0.090937829	0.899774
YBX1P10	3.284804146	0.02214	3.502672269	0.01303	3.695248463	0.008189
YBX1P2	3.464714079	0.010199	3.130122608	0.02141	1.774725117	0.239434
RPL3P4	-1.407703265	0.560938	-21.79074502	8.36E-17	-1.344142049	0.57844
ABCA6	0.676589751	0.05773	1.824834913	1.66E-07	0.628294363	0.077466
NXT2	0.156824609	0.182334	0.566477121	8.06E-07	0.553872417	1.31E-06
RP5-850E9.3	0.086375742	0.98612	22.86135021	2.55E-06	23.49577359	1.33E-06
SDCBP	0.244751201	0.214156	0.919306877	2.97E-06	0.598437313	0.002361
ALCAM	0.298980257	0.117572	0.881819469	3.80E-06	0.343989355	0.071566
LPGAT1	0.180388899	0.102878	0.496796201	6.58E-06	0.239729433	0.029828
PGM2L1	0.22692685	0.284598	0.929703819	1.08E-05	0.374232865	0.077028
TBL1XR1	0.126381761	0.195012	0.412454804	2.24E-05	0.217040691	0.025771
ABCA10	0.758395099	0.186305	2.349067677	3.34E-05	0.364302121	0.52574
ZNF189	0.054488221	0.653652	0.497071503	3.40E-05	0.298230776	0.013121
SELL	0.325663121	0.494761	1.876541557	3.47E-05	0.318404343	0.50022
ATP5G2	-0.155658114	0.08119	-0.361137835	5.24E-05	-0.272978904	0.002179

PKP4	0.166201359	0.132301	0.441231058	5.99E-05	0.292010268	0.007962
LIX1	-0.505725483	0.331122	-2.104653782	6.05E-05	0.51083336	0.324817
MMP10	1.052270002	0.107609	2.614177851	6.18E-05	0.563267473	0.389345
CTD-2008A1.	0.134176547	0.618775	1.061057579	7.40E-05	0.262418923	0.329036
SLC12A2	0.282900377	0.152857	0.772650785	9.41E-05	0.32331599	0.102278
GTDC1	0.203764969	0.142979	0.537299755	9.53E-05	0.184900016	0.180624
SORD	0.317669357	0.120375	0.792447655	0.0001	0.159794493	0.434526
STK38	0.139458569	0.096696	0.323011222	0.00011	0.265235738	0.001454
PSMB8	0.181555963	0.298262	0.663555442	0.00011	0.177282312	0.30616
SPG21	0.160581463	0.091847	0.363391873	0.00012	-0.000846184	0.992893
SKIL	0.263128668	0.135838	0.675760257	0.00012	0.380782201	0.030716
PSMB10	0.173946845	0.172089	0.472948459	0.00014	-0.061444128	0.629393
NDUFV2P1	0.600000958	0.073611	1.235206979	0.00015	0.607283386	0.067241
COPZ1	-0.08387566	0.33799	-0.327703703	0.00018	-0.09972176	0.252809
CUTA	-0.177865732	0.050139	-0.339147239	0.00018	-0.214465892	0.017267
ZNF354B	-0.178313137	0.364935	0.722279667	0.00019	0.198899706	0.307176
MARCKSL1	-0.011388274	0.883158	-0.28908204	0.00019	-0.066805101	0.387496
AMMECR1	0.184893866	0.131309	0.452264586	0.0002	0.291473251	0.01648
MYO1D	0.253970863	0.065526	0.509079467	0.00022	0.128379943	0.351774
SAMD9L	0.873332838	0.101457	1.916867068	0.00022	1.053056865	0.049073
SNIP1	0.051628591	0.720107	0.525507794	0.00023	0.351079467	0.013846
TMEM27	0.503516232	0.138412	1.236254803	0.00023	1.317957388	8.56E-05
GAMT	-0.252864785	0.207904	-0.74773812	0.00024	-0.737511845	0.000271
PLCD3	-0.013259996	0.953703	-0.85259247	0.00024	-0.655855595	0.004341
KCNN3	0.016008875	0.967909	-1.472796975	0.00025	-0.430026249	0.280737
STEAP2	0.120731017	0.455879	0.58916823	0.00025	0.065845803	0.684047
LRIG3	0.217826407	0.059147	0.420575951	0.00026	0.296594781	0.01002
ITGA8	-0.191824986	0.375302	-0.791796614	0.00026	-0.162438997	0.451915
THBS4	0.149576313	0.867891	3.183589017	0.0003	-0.561114641	0.533462
PDCD5	0.157468865	0.161447	0.40082729	0.00033	0.335111112	0.00267
CRK	0.081713202	0.318841	0.291779857	0.00033	0.304563751	0.000178
KCTD16	0.844436575	0.098807	1.822572378	0.00034	0.709024901	0.166874
BCL2L15	0.690138942	0.085407	1.430456562	0.00036	0.67848287	0.090821
SRPX	0.215695869	0.582371	1.382551332	0.00037	0.152278943	0.697592
IFNAR1	0.119239183	0.188296	0.320654865	0.00037	0.161802452	0.072871
OBSCN	0.041014636	0.806998	-0.600537174	0.00038	-0.467376502	0.005512
CDK19	0.163270622	0.097335	0.346266797	0.00039	0.154835891	0.113369
PAFAH1B3	-0.18371219	0.202963	-0.51392274	0.00039	-0.261285679	0.069131
CTNNA3	-0.511167552	0.101197	-1.117330868	0.00039	-0.403542478	0.191771
SMAD7	0.474164422	0.052676	0.863757439	0.0004	0.419567328	0.086206
MFGE8	-0.029710218	0.820457	-0.463786522	0.0004	-0.497210758	0.000151
EIF4A1	0.077722984	0.455689	0.365739638	0.00044	0.225568528	0.030259
CCDC102B	0.348285972	0.189082	0.913051191	0.00045	0.28078534	0.289377
P11-104O19	0.411601551	0.211388	1.077063119	0.00048	0.639096791	0.043543
NRXN2	1.137043413	0.239315	3.314351009	0.00049	-0.367521675	0.720563
RASGEF1B	0.204179046	0.174775	0.516026617	0.0005	0.148167846	0.320713
TMC7	0.365766737	0.087903	0.738356014	0.0005	0.189666548	0.374818

ABCA8	0.35458399	0.620871	2.427165757	0.00052	-0.393559968	0.59194
PROM1	0.238489058	0.223376	0.678652507	0.00052	0.250494691	0.200563
CTSK	0.471855711	0.258331	1.421260828	0.00052	0.48187166	0.247313
LPAR3	0.60080898	0.098697	1.205858238	0.00059	-0.28892878	0.446807
ACVR1	0.258123585	0.199677	0.678497643	0.00063	0.142826297	0.475464
ANXA8	1.30521597	0.114439	2.793199755	0.00065	0.572543813	0.4916
GALT	-0.127725703	0.294706	-0.412765267	0.00066	-0.194059917	0.107628
CRABP1	1.093718655	0.202475	2.899731077	0.00068	1.190929331	0.164568
NME7	-0.068029499	0.758399	0.745147692	0.00071	0.05369288	0.807778
AACS	-0.217067401	0.06384	-0.393264925	0.00077	-0.32950328	0.004792
SERTAD2	-0.099651166	0.563451	-0.582645442	0.00077	-0.322502955	0.06159
XDH	0.394362654	0.157578	0.931889479	0.00079	0.009378437	0.973299
FBXO36	-0.25333213	0.095339	-0.508203995	0.00081	-0.437646475	0.003559
TNFSF10	0.162220461	0.536216	0.870314861	0.00082	0.039462282	0.880245
GSTA2	-0.853752185	0.105849	-1.78703037	0.00084	-1.881950587	0.000443
NTNG1	0.671852589	0.221079	1.809849655	0.00087	0.971852682	0.075216
FAM27E4	-0.401413398	0.142398	-0.919113984	0.00098	-0.296019347	0.272047
GRPR	0.453117623	0.485861	-2.51696244	0.00098	-0.27933917	0.67149
KANK4	0.750563654	0.057547	1.296159067	0.001	0.302537646	0.444555
GJB2	0.538468667	0.100879	1.073949011	0.00101	0.065794815	0.841451
RNFT1	0.032007847	0.788276	0.377618035	0.00101	0.236695801	0.040836
CNIH4	0.13821574	0.199191	0.351826269	0.00103	0.26172072	0.014464
ABHD14B	-0.249947634	0.092468	-0.487836776	0.00103	-0.51686496	0.000512
KLHL13	0.556330419	0.184859	1.364178568	0.00107	-0.206181918	0.625093
DNAH12	-0.347480261	0.398898	-1.354096275	0.00109	-0.218071729	0.592942
RASSF10	0.221383412	0.398037	0.838866477	0.00111	0.350446789	0.176797
SCO2	0.171120916	0.222413	0.44195426	0.00112	0.190989449	0.164762
CBFB	-0.043763666	0.731362	0.412444551	0.00114	0.084165674	0.507421
SRI	0.071488921	0.441755	0.299745353	0.00114	0.157493505	0.087616
SDK1	-0.18345309	0.606558	-1.172259884	0.00115	-0.264161533	0.456161
RCAN2	0.465216055	0.352484	1.606592818	0.00118	0.337660971	0.49917
FOXF2	0.008438115	0.976964	-0.955340158	0.00119	0.055716473	0.848455
BMP7	-0.563452767	0.089024	-1.074565632	0.00123	-0.781746724	0.018439
PRRG4	0.182260217	0.229954	0.479309541	0.00129	0.06250761	0.678987
MORF4L2	0.141942741	0.072537	0.252689384	0.00137	0.282678106	0.000342
CD47	0.062625186	0.705152	0.52776358	0.00141	0.230136073	0.163918
CRYBG3	0.310241816	0.094956	0.592844572	0.0014	-0.010570871	0.95464
BCL2L11	0.632092025	0.067926	1.090751079	0.00144	0.300193528	0.384584
ITM2A	-0.176070295	0.799339	2.180326467	0.00147	-1.071961211	0.124699
PI4KAP2	0.078869234	0.531445	-0.401114661	0.00154	-0.102362862	0.414831
LRFN5	0.712267757	0.065353	1.221009333	0.00154	0.879668883	0.022599
CLTC	0.094080635	0.165668	0.213589995	0.00161	0.123931356	0.067418
RNF14	0.120240446	0.206235	0.293692003	0.00168	0.1572338	0.093249
TMED4	-0.097802093	0.221853	-0.251134713	0.0017	-0.231377402	0.003771
EZR	-0.235790111	0.063907	-0.398750623	0.00173	-0.232263676	0.067841
BZW1	0.109789623	0.212815	0.275417282	0.00176	0.218378495	0.013109
RAB9B	-0.13497279	0.561803	0.689081636	0.00176	-0.126295419	0.581094

APOD	1.618384545	0.085294	2.923266243	0.00177	-0.406227732	0.672508
CAPZA1	0.077798108	0.191138	0.185352772	0.00177	0.17913149	0.002493
EDN3	-0.108935683	0.779234	-1.211904474	0.00188	0.134900244	0.728143
ABCA9	0.206712408	0.580236	1.150197472	0.0019	0.110150357	0.768069
CAPN13	-0.405066066	0.118486	-0.799549753	0.0019	-0.514145944	0.045107
FADD	0.017586095	0.867493	0.316558822	0.00195	0.127464269	0.215845
RNF103	0.193955914	0.235909	0.50248645	0.00205	0.374486728	0.021659
KRBA1	0.53476702	0.051768	0.838835251	0.00214	0.44440877	0.104675
NUAK1	0.655521186	0.055995	1.04461672	0.00215	0.271571284	0.428698
RASL12	0.763723199	0.167785	1.679565144	0.00216	-0.067307896	0.904322
PRDX2	-0.027241015	0.743335	-0.25503837	0.00217	-0.163016574	0.049732
FAM26E	0.571601548	0.180471	1.290561194	0.0022	0.667458042	0.115861
IGFN1	1.337529812	0.246348	3.477514175	0.00222	0.956294684	0.412899
KIF12	-0.486499653	0.097325	-0.897232063	0.00225	-0.170616802	0.55548
HOXD10	-0.151634968	0.849192	-2.481668423	0.00226	-0.720335912	0.367413
CTNNA1	0.131821373	0.096803	0.241980753	0.00228	0.236159784	0.002894
ETF1	0.138884956	0.083166	0.243407173	0.00228	0.425918866	8.62E-08
GABRR1	0.4136921	0.637255	2.642499924	0.00227	0.278964963	0.751186
ARHGAP42	-0.168115148	0.114975	-0.324382496	0.00229	-0.03140737	0.766398
MICAL2	0.224368473	0.304844	0.666069306	0.00228	0.287557842	0.188137
SLC29A4	0.088786807	0.569913	-0.480947391	0.0023	-0.361215284	0.021364
LIMS1	0.0610774	0.502017	0.275760102	0.00231	0.185724245	0.040241
QPRT	0.044840622	0.65974	-0.311139498	0.00232	0.024017273	0.812977
CHI3L2	0.755184362	0.203674	1.7445315	0.00232	0.918953565	0.11081
AMIGO2	0.215043821	0.45399	0.87082561	0.00234	-0.121087164	0.673488
2-Sep	0.007001011	0.897978	0.165662532	0.00234	0.153739494	0.004729
P11-497H16	-0.316649126	0.564916	-1.724474194	0.00237	-0.383290354	0.486195
YIPF5	0.115324527	0.352258	0.374780206	0.00238	0.141513915	0.252216
UBE2J2	0.187451594	0.182253	0.423316122	0.00244	0.111932533	0.426823
PCF11	0.115022631	0.193049	0.265889613	0.00247	0.243723652	0.005471
MAL2	1.240429701	0.13129	2.470223117	0.00249	0.51500845	0.531641
ZFP91-CNTF	1.375755076	0.052516	2.121399388	0.00252	0.985857471	0.165703
FZD10	0.338392924	0.47617	-1.466838131	0.00265	-0.782647102	0.10324
SEMA3E	0.475098009	0.250366	1.237101017	0.00267	0.540233202	0.190485
CD180	1.067661611	0.077525	1.757758172	0.00272	0.943164857	0.116423
KIF2A	0.094045535	0.415494	0.343696288	0.00276	0.299620684	0.00897
RAI14	0.277816263	0.168941	0.601543822	0.00285	0.118283813	0.558071
KCNJ2	0.819605695	0.117635	1.541616069	0.00291	-0.262845336	0.620845
VRK2	0.056682321	0.643586	0.357897278	0.00292	0.13999126	0.247096
PCDP1	0.520316486	0.070413	0.843633222	0.00296	1.184579641	2.52E-05
NOS3	-0.146291995	0.556673	-0.743165993	0.00299	-0.725630077	0.003553
FADS3	0.397983911	0.131754	0.776739104	0.003	0.347838426	0.188543
OLFM3	2.138788175	0.115857	4.013117196	0.00304	0.707678386	0.605621
UXS1	0.179189718	0.066808	0.287244339	0.00305	0.276522939	0.004268
FBLN2	0.544350644	0.290764	1.518879883	0.00308	0.058450376	0.910516
SUDS3	-0.131632471	0.105206	-0.238912753	0.00313	-0.076390247	0.342854
SYT4	1.646673705	0.067899	2.635885753	0.00319	1.872542203	0.037417

PYGL	0.202965964	0.18959	0.451637202	0.00321	-0.130262925	0.400715
RBM11	0.598579004	0.149285	1.180332553	0.00326	0.377853527	0.363695
UBA6	0.11611496	0.189117	0.259078208	0.00325	0.342670299	9.83E-05
PTPLA	-0.083789358	0.768328	-0.843074315	0.00327	0.040862616	0.884181
CTHRC1	0.792584132	0.278514	2.138037631	0.0033	0.442296497	0.545279
PSME2	0.004038203	0.968296	0.295239847	0.0033	0.212522112	0.034482
P11-848G14	-0.756556482	0.261448	-2.036828351	0.00331	-0.167305576	0.800763
TLR2	0.057703951	0.800827	0.651778041	0.00333	0.014481073	0.949077
TLR3	-0.224557602	0.466822	0.889129074	0.00334	-0.51106855	0.097463
TTC39C	-0.188389324	0.182398	-0.415691956	0.00334	-0.106240872	0.44753
C11orf84	0.065088871	0.580712	-0.34753915	0.00338	-0.034113953	0.771534
ARPC5	0.097096925	0.335741	0.294344819	0.0034	0.242691439	0.015661
BLZF1	-0.077426431	0.373882	0.247852573	0.00342	0.159963626	0.059322
KAT6B	-0.070861093	0.492139	-0.30186577	0.00345	-0.174587112	0.088349
BMPER	1.156720137	0.067645	1.842934385	0.00348	0.039623297	0.950419
C19orf33	-0.288377036	0.079499	-0.480397945	0.00349	-0.608936263	0.000219
TRAFD1	0.163350442	0.160377	0.336227375	0.00347	0.475036069	3.45E-05
PTPN2	0.176014633	0.09821	0.308211662	0.00353	0.392750031	0.000195
ATG4A	0.171013842	0.35807	0.535864312	0.00356	0.297986464	0.106168
CCDC53	-0.085992562	0.510225	0.369328701	0.00358	-0.02038422	0.874116
CREBL2	-0.063155697	0.545419	0.296718675	0.00359	-0.039102422	0.704553
PPP1R14C	1.104871615	0.132702	2.110219028	0.0036	-0.628905092	0.411579
SLC26A11	0.365082141	0.059773	0.56044914	0.00361	0.480611707	0.012682
PSAPL1	-0.26165663	0.428763	-0.965581365	0.00366	-0.564417309	0.087996
ANKRD13A	0.043636479	0.673935	0.297609393	0.00383	0.241025162	0.018966
HIGD2A	-0.09053088	0.389317	-0.303862939	0.00391	-0.226825003	0.030306
CDC42SE2	0.104696698	0.273922	0.274337596	0.00394	0.309467295	0.001132
VMP1	0.220557553	0.060943	0.339175809	0.00394	0.31435937	0.007513
TRAPPC2	-0.207973803	0.085724	-0.345634164	0.00398	-0.266696787	0.025683
TACC1	-0.23433085	0.203009	-0.529410039	0.00401	0.069294059	0.706068
PROCR	-0.118996176	0.781029	1.193014047	0.00412	-0.176695756	0.678218
KCNH2	0.237873777	0.169907	-0.507705585	0.00415	0.040656015	0.814518
NUDC	-0.10575388	0.314159	-0.300326819	0.00426	-0.21693405	0.038537
POMP	-0.10899134	0.148199	0.211062479	0.00432	0.168396891	0.022729
EDN1	0.282459286	0.301005	0.768581099	0.00433	0.416388749	0.124189
MUSK	-0.43789667	0.30535	-1.26633473	0.00435	-0.199214005	0.623823
RAB3B	-0.165554381	0.542631	-0.775686569	0.00436	-0.051533561	0.849637
HPSE2	-0.01179959	0.96479	-0.76688182	0.00439	-0.060715908	0.819992
PBX4	-0.124086163	0.678478	-0.921991049	0.00437	-0.792178536	0.011478
AKT3	-0.006790108	0.976326	0.645576639	0.00442	0.472652055	0.037212
FOXF1	0.453828413	0.073881	-0.725993123	0.00444	0.248694166	0.327299
B2M	0.244245261	0.07971	0.396031436	0.00448	0.238731728	0.086694
LHFP	0.759089772	0.070732	1.190846054	0.00448	-0.005191956	0.990166
TPBG	0.241249323	0.236186	0.575542281	0.00452	0.083870758	0.680223
GRIA4	0.161323844	0.656734	-1.040067281	0.00454	0.174253394	0.630379
ARHGAP4	-0.459564863	0.152268	-0.905667603	0.00463	-1.310684957	7.32E-05
CD82	0.4325099	0.107291	0.750304192	0.00467	-0.422393148	0.118237

CDK17	0.236352158	0.092225	0.394830365	0.00467	0.632277284	5.44E-06
PYGM	0.480848757	0.202682	1.041899097	0.0047	0.352534849	0.352423
SLC36A4	0.088460986	0.492101	0.36113362	0.00471	0.456781153	0.000331
MMP13	0.824414448	0.314446	2.290463039	0.00473	-0.922306901	0.276692
AHCYL1	0.201730589	0.082319	0.32641935	0.00483	0.27807219	0.016415
GREM1	0.452449027	0.257331	1.121416852	0.00484	1.086857801	0.006327
ZNF678	-0.201746674	0.128268	-0.371817279	0.00486	0.0099224	0.939748
DLC1	0.528661712	0.083642	0.859048282	0.00487	0.142593175	0.640844
RBM18	0.065582384	0.600323	0.347616231	0.00498	0.131215922	0.288781
RPL38	0.26035736	0.054046	0.378371377	0.00509	0.214133672	0.112961
AP5M1	0.108481069	0.380215	0.344111242	0.00516	0.16329746	0.18425
HNRNPA3P9	0.467022347	0.239179	1.047701697	0.00519	1.22084776	0.000962
NMB	0.464483623	0.051286	0.657254656	0.00524	0.233199929	0.327679
EI24	0.142261517	0.283406	0.36924142	0.00525	0.196564801	0.13763
ELTD1	0.433494562	0.344516	1.243281667	0.00525	-0.169939556	0.714764
CYP2S1	-0.24535691	0.289681	-0.648704754	0.00527	-1.104995915	2.22E-06
MTG1	0.074876185	0.523464	0.323323758	0.00532	0.163985577	0.157762
BICC1	0.357686371	0.196507	0.77019802	0.00534	0.326724994	0.23784
DPYSL3	0.221662627	0.691485	1.551521909	0.00539	-0.30545555	0.584539
CCND2	-0.040641828	0.73989	-0.340346109	0.00544	-0.226132813	0.064679
CDH2	0.437539042	0.33701	1.264975604	0.00546	0.537885993	0.237695
DCHS2	0.890459756	0.105176	1.515154528	0.00548	0.229539962	0.680864
MAB21L1	0.622603724	0.454876	2.260332335	0.00552	0.757621527	0.359756
FAM175A	-0.147597253	0.14948	-0.282006655	0.00558	-0.035759198	0.72181
DUSP11	-0.004230028	0.968874	0.292876492	0.00561	0.188513842	0.074439
SETDB1	-0.146038105	0.13796	-0.272464103	0.00561	-0.082067098	0.401548
ANKRD20A5P	-0.43557412	0.393213	-1.44208762	0.00566	-0.724880075	0.156962
EPSTI1	0.085414728	0.792181	0.884228723	0.00572	0.367694322	0.251623
NASP	-0.199502537	0.17917	-0.410343773	0.00572	-0.161990388	0.274549
TMEM183A	-0.017317186	0.829633	0.219436022	0.00579	0.135792379	0.088024
ITGB5	-0.245665188	0.111932	-0.426251686	0.00582	-0.460089396	0.002913
KCTD20	0.03174361	0.729008	0.25060143	0.00585	0.219332787	0.016026
KCTD1	-0.195346291	0.301185	-0.521604393	0.00586	-0.41791028	0.02693
HGF	1.080246093	0.111344	1.866723806	0.00591	-0.076086359	0.910813
MICAL3	0.293761737	0.062556	0.432823792	0.00591	-0.025801123	0.870111
SGCB	-0.006713221	0.96633	0.433232638	0.00597	0.072852525	0.644913
KIAA1958	0.039690075	0.782858	-0.396525109	0.00598	-0.134173673	0.348653
HTR2A	0.268127306	0.479741	-1.075577167	0.00602	0.677949019	0.071261
SLC8A1	0.091365388	0.787176	-0.93181229	0.00612	0.381932705	0.258404
E2F5	-0.267729669	0.125297	-0.477009931	0.00615	-0.166449038	0.337078
NSUN6	-0.291791736	0.067904	-0.437516078	0.00617	-0.152669574	0.332733
PTGES2	0.321553033	0.058675	0.463379005	0.00618	0.350651514	0.03852
FOS	-0.224929535	0.311774	-0.608677235	0.00622	-0.290965898	0.190186
PHF10	0.607093179	0.217678	1.34028776	0.00623	0.981659708	0.045502
METTL7A	-0.352151296	0.053038	-0.49733355	0.00627	-0.633925895	0.000496
RAP2A	-0.02820837	0.706918	0.202368846	0.00627	0.1267126	0.086965
CP	0.197248342	0.389479	0.624751251	0.00641	0.421709537	0.065745

MRPS6	0.11393956	0.21485	0.247663871	0.00645	0.028733188	0.753277
EMP1	0.49979173	0.353823	1.464428482	0.00652	-0.022496581	0.966712
PFKFB2	0.160995511	0.349909	0.465864427	0.00658	0.241310364	0.161016
CMYA5	0.445938584	0.336734	1.24365031	0.0066	0.151075531	0.744562
TGFB2	1.336325584	0.086111	2.113131942	0.00661	0.885141282	0.255793
COL16A1	0.534366699	0.090504	0.855693538	0.00668	0.305254644	0.333643
ABCG1	0.462455461	0.254966	1.093533217	0.00675	-0.018572417	0.963707
SOCS5	0.20313807	0.168293	0.397325392	0.00676	0.294312457	0.04496
BOC	0.733797038	0.118121	1.269254215	0.00678	-0.073488755	0.875784
SORCS3	2.093254353	0.165141	4.061760588	0.00682	-1.741538755	0.292776
IFI44L	0.293844032	0.463716	1.073578565	0.00691	-0.337920405	0.401081
CYP27C1	0.325554339	0.229309	0.719444813	0.00698	0.435385354	0.104686
IL10	0.469408938	0.378218	1.33643219	0.00711	0.771585358	0.129184
TLR5	0.341749318	0.432631	1.150611799	0.00712	0.574870673	0.187415
OXSR1	-0.128332576	0.25458	-0.302149479	0.00727	-0.063544084	0.571252
EEF1A1P17	0.831615648	0.142799	1.442037183	0.00732	1.348977012	0.012256
CAPN5	-0.157759484	0.32701	-0.431767831	0.00735	-0.60965592	0.000158
SPHK1	0.726663312	0.105185	1.191129397	0.00741	0.517340498	0.249972
TIMP1	-0.063288401	0.791798	0.640100335	0.00756	-0.31792282	0.184818
RAB32	0.230593014	0.186824	0.458007091	0.00759	0.507807985	0.00296
SAMHD1	-0.047832814	0.754127	0.396947985	0.00767	-0.046870451	0.755999
SOCS6	0.095311466	0.368269	0.279909342	0.00768	0.196251058	0.061287
KCNK2	0.208097817	0.632438	1.153698838	0.00774	0.7142947	0.09947
EEF1A1P38	0.77193316	0.138693	1.311848222	0.00776	0.490367018	0.349074
RPL32P29	-0.305810542	0.108403	-0.507443356	0.00778	-0.230827675	0.219597
CLN6	-0.109682356	0.411992	-0.353374627	0.00786	-0.42380095	0.001424
CMPK1	0.040575572	0.763883	0.358372811	0.00787	0.220272915	0.102511
CCDC82	0.178159253	0.153147	0.328509919	0.00791	0.475911474	0.000112
IFI35	-0.01283871	0.938863	0.431852211	0.00792	-0.054156124	0.744897
KCNJ15	-0.140845967	0.791741	1.405294707	0.00791	0.049084495	0.926379
POSTN	0.494558686	0.296559	1.257921392	0.00792	0.122154932	0.796544
NOV	0.193393459	0.716053	1.330295629	0.008	0.280580978	0.590202
PPP3CA	-0.032240377	0.779694	0.304457497	0.00799	0.135081083	0.240155
STX7	0.281638644	0.054071	0.386692853	0.008	0.538426531	0.000221
CCDC85C	-0.261413927	0.087864	-0.405518934	0.00801	-0.444369512	0.003629
IGSF9	-0.432738801	0.089207	-0.675003073	0.00806	-1.248971066	1.17E-06
TSC22D1	0.11425778	0.518546	-0.469086113	0.00806	-0.201976083	0.253653
RARRES2	0.271495421	0.262677	0.632228888	0.00807	-0.044381025	0.854967
BNIP3L	0.250936786	0.058403	0.350519912	0.00814	0.327156307	0.013511
C9orf72	-0.194790758	0.26191	-0.459694176	0.00811	0.123999218	0.471628
CARD6	0.129961696	0.325764	0.344711343	0.00813	0.284944187	0.028867
CCBL1	-0.267705951	0.283156	-0.670603601	0.00812	-0.274973092	0.269499
LPAR2	0.055920386	0.714004	-0.407117481	0.00809	-0.165116265	0.278534
ANKH	0.426219703	0.224781	0.925409025	0.00829	-0.004621552	0.989509
RER1	0.067332354	0.552201	0.297342999	0.00828	0.057433403	0.611262
RWDD4P2	1.147409212	0.071901	1.671835818	0.0083	1.753534921	0.005587
C1QL1	0.72898455	0.088971	-1.345629924	0.00832	-0.699588428	0.135285

EMCN	0.52259183	0.374831	1.549753207	0.00834	-0.032915778	0.955459
TNFRSF8	1.083050794	0.258464	2.457877234	0.00841	0.200563965	0.837388
FLRT3	0.23266961	0.148132	0.423024859	0.00845	0.347142044	0.030699
CNTNAP5	0.275821937	0.783949	2.489833596	0.00847	1.231160976	0.196987
SLC1A3	-0.389068785	0.195171	-0.792985544	0.00847	-0.821831631	0.006295
SCYL2	0.11935455	0.287794	0.294841373	0.0085	0.242894057	0.030035
KIAA1462	0.572925497	0.084698	0.872626279	0.00853	0.326764818	0.325518
LST1	0.031601891	0.961732	1.649254298	0.00858	0.489345791	0.4572
MXRA5	0.813350218	0.15019	1.484973984	0.0086	0.107025922	0.849856
TECRL	0.550437171	0.263405	1.271069463	0.00864	0.914735831	0.059928
ANKRD45	-0.221535128	0.421373	-0.726880571	0.00875	-0.331540325	0.227643
DEF8	0.291425554	0.235619	0.641061945	0.00875	0.458543926	0.060923
ALDH3A1	-0.818530018	0.151831	-1.49842055	0.00885	-1.788789399	0.002135
EYA4	1.1637396	0.100192	1.841229713	0.00895	-0.094536407	0.894536
CTBP2P8	-0.372807928	0.497351	-1.579743598	0.00919	-0.561408477	0.305154
GPR39	-0.401405387	0.08913	-0.614731439	0.00918	-0.757170435	0.001396
EIF4E2	-0.081974503	0.416124	0.259810941	0.00926	0.133332806	0.182368
FGFR3	-0.293167565	0.087937	-0.445898602	0.00928	-0.63897425	0.000195
MEOX2	0.00682137	0.992464	1.859801536	0.00929	-0.75339939	0.299604
MTERFD3	-0.241635745	0.108781	-0.390591258	0.00932	-0.117553427	0.425776
SLC4A2	0.243806197	0.110587	0.396069121	0.00937	0.193592272	0.204398
ADPGK	-0.071274422	0.558623	0.314129065	0.00943	0.052504658	0.664286
TRIM27	0.319730205	0.072807	0.462082437	0.00946	0.566741911	0.00144
COMMD2	-0.019277081	0.852758	0.265923539	0.0095	0.177666456	0.082528
ARL2	-0.21232464	0.142035	-0.372914921	0.00958	-0.209441077	0.145299
BCHE	-0.481506106	0.183765	-0.939235535	0.00962	0.250311168	0.488568
IFT140	-0.221606405	0.113004	-0.360038815	0.00963	-0.250565472	0.071275
ATP6V1B2	0.091625582	0.285016	0.219200522	0.00967	0.265165105	0.001694
CHODL	-0.642326531	0.243221	-1.425648553	0.00966	-0.479097823	0.383839
MRAS	0.080951628	0.873837	1.287947647	0.00971	0.795689443	0.11042
XPC	-0.278079958	0.074673	-0.400636898	0.00983	-0.136059191	0.380713
PRPF38A	-0.014836286	0.851384	0.202227491	0.0099	0.255262927	0.001026
SLC27A1	0.106410455	0.461268	-0.376824932	0.00999	-0.177068214	0.221268
LUC7L3	-0.068516865	0.642348	-0.379917399	0.01	0.273930292	0.062727
ANGPTL3	-0.628271677	0.119084	-1.041400504	0.01005	-0.358695101	0.366448
IPCEF1	0.755051056	0.134463	1.286658903	0.01009	1.291275819	0.009752
LSAMP	0.148731047	0.468025	-0.530316422	0.01013	0.119705956	0.558647
PDGFB	0.681707612	0.323985	1.769591465	0.01013	-0.608245641	0.382681
IGFBP4	0.450502403	0.123478	0.749296429	0.01016	-0.027256718	0.925898
GNAI3	0.071324122	0.245907	0.156935798	0.01024	0.187491744	0.002126
TAC1	0.929429781	0.471639	3.309563851	0.01027	0.70060817	0.587439
ETV4	-0.103221887	0.580773	-0.480043579	0.01033	-0.184788557	0.321847
GALNT4	0.015132007	0.927712	0.426612875	0.01036	0.516187352	0.001914
PBX1	0.161165389	0.290651	-0.392733227	0.01035	-0.166411895	0.275813
ADAM23	0.508432676	0.125729	0.849550075	0.01042	0.398425546	0.230699
CYP1B1	1.0163222	0.085229	1.502585653	0.01047	0.095683975	0.872763
HPN	-0.257715209	0.273839	-0.603407929	0.01048	-0.381333627	0.105293

DLG3	0.105343045	0.433984	0.342959182	0.01054	0.232780227	0.082512
VEZT	0.058278016	0.543691	0.244580456	0.01054	0.319372651	0.000825
MINA	0.079156841	0.643699	0.433902161	0.01057	0.066531143	0.696412
NFE2	-0.89945116	0.059266	-1.218958539	0.01057	-1.029180006	0.030228
IGDCC3	0.465707172	0.099859	-0.727998491	0.01061	0.243839635	0.388679
BIN1	-0.187793218	0.095111	-0.286925086	0.01067	-0.465435999	3.65E-05
STRN	0.128400041	0.244973	0.280087615	0.01071	0.270497055	0.01359
YWHAB	0.07448237	0.357115	0.205406528	0.01083	0.121347658	0.132374
PCED1B	0.441849942	0.184981	0.827862799	0.011	-0.105927601	0.753877
ZFP91	-0.119996516	0.080674	-0.174002057	0.01102	-0.022531941	0.7408
STEAP1	-0.194861317	0.329589	0.499379997	0.01127	-0.051821976	0.794023
ITPRIPL2	0.214274727	0.139862	0.366234713	0.01142	-0.047417865	0.743904
SRP54	0.049952436	0.581753	0.227617904	0.01142	0.277474573	0.002005
CASZ1	0.512429049	0.087637	0.759576426	0.01148	0.527549895	0.079688
TRAM1	0.099935024	0.450406	0.334403881	0.01147	0.121748972	0.35749
CLCN7	0.194884719	0.168779	0.355748524	0.0116	-0.068911634	0.627604
PTPRN2	-0.155614985	0.556284	-0.668818485	0.01168	-0.839436795	0.001571
AKR1B10	-1.28847867	0.068896	-1.789499217	0.01181	-2.254661201	0.001543
DNM3	0.170063542	0.667586	0.991611738	0.01181	-0.236289122	0.551813
MBOAT1	-0.04387728	0.78598	0.402603181	0.01183	-0.096631884	0.548459
UPF3B	-0.076574078	0.668059	-0.452134018	0.01186	-0.155442948	0.382525
DHTKD1	-0.092135236	0.570016	-0.407206405	0.01189	-0.235919644	0.144684
FRZB	-0.161708658	0.487274	-0.586970123	0.01188	0.148427036	0.522007
SIDT2	0.221342221	0.443641	-0.730654607	0.01191	-0.052336574	0.856409
:SGALNACT	0.369228872	0.050732	0.472079105	0.01195	0.142941398	0.449305
AQP12A	-0.181868783	0.678211	-1.157367617	0.012	-1.063288365	0.019501
NTHL1	-0.232702667	0.208921	-0.464926846	0.01201	-0.433516963	0.018032
ENTPD2	-0.476575208	0.399102	-1.486500253	0.0121	-0.868578209	0.128116
ENG	0.605495674	0.082573	0.867423907	0.01212	-0.480797703	0.17426
BCL2L13	-0.052881575	0.673265	-0.313065896	0.01218	-0.029341776	0.814085
PLSCR4	0.474319768	0.125081	0.770613916	0.01219	0.42995111	0.163384
MGP	0.083069455	0.907367	1.786201279	0.01221	-0.328319816	0.645713
PER3	-0.057873618	0.889057	-1.095401045	0.01221	-0.084609902	0.838773
ABHD11	-0.140550186	0.330199	-0.360848251	0.01225	-0.394972991	0.006232
NALCN	0.25415283	0.585939	1.130265855	0.01233	-0.146979362	0.754298
NGFR	0.700029252	0.308942	1.709079403	0.01233	-0.062346133	0.928206
NSUN5P2	-0.48541238	0.235051	-1.027456244	0.01239	-1.333101221	0.001322
UBLCP1	0.085313806	0.427293	0.26477071	0.01238	0.202779015	0.055135
TRPV6	1.86075366	0.054724	2.350500754	0.01249	2.357879853	0.011851
CHTF18	-0.261788815	0.221354	-0.534041322	0.01251	-0.472121568	0.027885
NPY1R	1.151060219	0.096875	1.7264462	0.01251	0.51583757	0.457228
C18orf8	0.269902279	0.067144	0.364590516	0.01254	0.299006188	0.040726
D2HGDH	-0.401867972	0.136809	-0.673540168	0.01253	-0.625833087	0.020842
PDE3B	-0.146234636	0.548641	-0.608834696	0.01256	-0.15273224	0.530342
IQSEC2	-0.389749045	0.202553	-0.774375053	0.01261	-0.932478917	0.002622
DCAF16	-0.087980841	0.362734	-0.24046016	0.01265	-0.051605266	0.591041
ACY1	-0.080716855	0.679485	-0.487219088	0.0128	-0.261304997	0.18088

TP53TG5	0.537820956	0.165875	0.962076703	0.01291	0.173158411	0.657572
IDUA	-0.06169419	0.788722	-0.580702626	0.01305	-0.708633058	0.002773
PTCHD4	-0.325429286	0.349644	-0.869111639	0.01304	-0.687884753	0.048454
TMEM87B	-0.039538033	0.740118	0.293931855	0.01321	-0.130602015	0.271647
AKTIP	0.019508775	0.896422	0.363814887	0.01326	0.249454465	0.08991
PSMB2	0.116170829	0.235147	0.239544169	0.01325	0.152788205	0.114541
BNC2	0.558835368	0.09057	0.815967173	0.0134	0.239387071	0.468517
IAJC25-GNG	0.099153294	0.702317	0.636669602	0.01346	0.714870344	0.005479
HDAC4	0.389581444	0.102962	0.589116373	0.01361	0.446601761	0.061286
P11-603J24	0.54488976	0.095661	0.803573968	0.01364	0.695819758	0.032763
CHD6	-0.260275279	0.060455	-0.341454845	0.0137	-0.151129748	0.275303
LARGE	0.157237492	0.204646	0.304123948	0.01369	0.044103175	0.721745
P11-814E24	0.246286316	0.683863	-1.674839004	0.01373	0.197019426	0.742915
PRG4	-0.507611767	0.466882	1.542108502	0.01375	-0.6081229	0.366706
ACVR2B	-0.073579232	0.566674	-0.316433822	0.01381	0.091381843	0.474678
CSNK1A1	-0.047287081	0.486771	0.166737139	0.01382	0.177566863	0.008729
NEIL2	-0.062292196	0.704389	0.39516112	0.0138	0.031205292	0.847561
NOL8	-0.191067388	0.262205	-0.418456038	0.01382	0.023687323	0.888842
VWC2	0.001438307	0.998606	1.990752847	0.0138	-1.01691078	0.224256
SLC25A23	0.152229393	0.153252	-0.26406731	0.01389	-0.014080771	0.89496
ATF3	0.108385622	0.820429	-1.186810158	0.01391	0.154464387	0.746011
SUCNR1	0.813881328	0.410609	2.426522016	0.01396	-0.057609597	0.953629
ATP6V0E1	0.173303788	0.14916	0.294045011	0.01402	0.18131766	0.130098
NUDT22	0.205811595	0.118391	0.32007551	0.014	-0.033552168	0.79977
PDXDC2P	-0.262929822	0.093162	-0.38255258	0.01402	-0.019787124	0.897743
SESN2	-0.076746932	0.660635	-0.430607961	0.01403	-0.248373662	0.154938
IFIT3	0.530275512	0.062165	0.691433384	0.01413	0.771595868	0.006026
CCNB1IP1	-0.210030025	0.086747	-0.300269223	0.01418	-0.075794831	0.533868
EFHD2	0.344144181	0.096936	0.507142703	0.01427	-0.192763655	0.353889
TCEB1	0.102110319	0.321891	0.250341051	0.01431	0.250857067	0.013988
CERS6	0.033768515	0.745119	0.253414694	0.01441	0.094848941	0.360098
CYP2W1	-0.057576949	0.859529	-0.813668445	0.01445	-0.838458575	0.012379
HOXA13	-1.155377272	0.227358	-2.37484748	0.01446	0.071550845	0.940023
ATRX	-0.125205369	0.119641	-0.196270052	0.0146	-0.044017096	0.58291
COL27A1	-0.0542723	0.711341	-0.35822968	0.01462	-0.038939629	0.790341
CASP1	0.312590562	0.465943	1.025100395	0.01467	0.465273572	0.265935
LHX8	0.395252382	0.789549	3.505708275	0.01474	-1.621468651	0.307811
ZNF557	0.087019415	0.423456	0.261825972	0.01473	0.287290772	0.007021
FAM43A	0.867405465	0.066646	1.146221333	0.0148	0.056032105	0.906597
HELLS	0.09241999	0.68352	0.551715718	0.01481	0.129445294	0.567752
CHN2	-0.217175021	0.243497	-0.452316039	0.0149	-0.276304833	0.134629
CHST4	0.44118773	0.205778	0.830535338	0.01496	0.865787494	0.01107
MBTPS1	0.063047385	0.386401	0.176050513	0.01495	-0.002240293	0.975326
PARD6B	-0.234492843	0.114284	-0.360992845	0.01495	-0.120211836	0.415891
P11-1055B8	-0.25338035	0.853405	-3.380169838	0.01508	-1.156105545	0.399772
PALMD	0.63923237	0.130562	1.02384539	0.01514	0.372838145	0.37793
GRPEL2	-0.170609256	0.093295	-0.245552983	0.01516	-0.021169762	0.832305

FAM86EP	-0.404259639	0.151027	-0.697611677	0.01522	-0.992548875	0.000527
TENM4	0.255989882	0.542676	-1.027757813	0.01522	-0.208948696	0.622993
HOXD11	-0.540673228	0.550214	-2.26130164	0.01535	-1.126664718	0.215692
PFAS	0.039995239	0.798977	-0.380672153	0.01536	-0.039868133	0.799183
FOXS1	1.401571841	0.103067	2.059552308	0.01542	-0.349873154	0.696934
RGL2	0.023378623	0.822932	-0.252815253	0.01542	-0.230844586	0.026727
EOGT	0.088479247	0.533137	0.333507485	0.01548	0.12916342	0.353425
MEOX1	1.440352604	0.060614	1.852764205	0.01561	0.67949779	0.376706
SLFN11	0.400330539	0.303666	0.939666997	0.01561	0.097418023	0.802324
CHRM3	-0.477765406	0.167454	-0.834996463	0.01586	-1.305714814	0.000171
TPGS2	-0.008755959	0.925336	-0.225355015	0.01586	-0.081144974	0.383279
AC140481.2	-0.363199961	0.402974	-1.126102139	0.0159	-0.124619144	0.763728
NFIL3	-0.193315648	0.392337	-0.54514243	0.01598	-0.007082654	0.974938
SART3	-0.088294068	0.232902	-0.177074495	0.01596	-0.050493312	0.489383
ZNF518B	0.158462321	0.157535	0.269461233	0.01596	0.316688275	0.004565
SECISBP2	-0.123034443	0.198954	-0.230025718	0.01599	-0.053175567	0.575813
ARAP3	0.128171674	0.516116	0.471522471	0.01617	0.103260277	0.599794
RPS9	-0.094753735	0.699751	-0.591600816	0.01616	-0.339296003	0.167284
GNE	0.305935029	0.116259	0.46713085	0.01623	0.073120247	0.707333
CDH9	1.325701108	0.147724	2.156466172	0.01627	0.861923804	0.350498
SLC16A5	0.21454317	0.134122	-0.349814388	0.01631	0.050073098	0.725742
TRHDE	-0.649042465	0.061016	-0.824440396	0.01646	-0.622035769	0.07103
ACSL3	0.078507131	0.528348	0.29825745	0.01651	0.468465579	0.000164
ELK4	0.062393125	0.658449	0.335868518	0.01664	0.062751934	0.6554
SCFD2	-0.343788348	0.083654	-0.475272855	0.01664	-0.241534722	0.221373
VPS39	0.026141852	0.807676	0.255858557	0.01667	0.20636617	0.05301
BBS9	-0.21023796	0.362596	-0.549462267	0.01671	-0.407473565	0.076663
C7	-0.014986852	0.982649	1.647020228	0.01673	-0.240668621	0.726895
CBLC	-0.220716941	0.239289	-0.449125384	0.01679	-0.485709799	0.009659
CD58	0.082511604	0.638824	0.412450368	0.0169	0.108193507	0.535139
C4B	0.197912662	0.672646	1.086464639	0.01693	0.199478121	0.665135
NNMT	0.939060307	0.108021	1.392843746	0.01692	0.979293581	0.093397
FBXL22	-0.031437082	0.950368	-1.275652219	0.01701	-0.759874293	0.131544
PLCL2	0.312795395	0.244293	0.633921944	0.01701	0.277343108	0.299071
RNF6	0.168592924	0.185572	0.302133466	0.01704	0.446749812	0.000413
PDE1A	-0.08119201	0.780896	-0.697718337	0.01708	0.454956996	0.118122
SIK1	-0.004784816	0.993866	-1.48761993	0.01707	-0.795670252	0.201884
AZI1	-0.075051853	0.703775	-0.469724074	0.01712	0.042624767	0.827929
C5orf15	0.155544213	0.212835	0.296569408	0.01717	0.062369296	0.616374
MUTYH	-0.142851436	0.265047	-0.303441896	0.01725	-0.202952141	0.110312
SEMA7A	0.5913566	0.241238	1.198083262	0.01727	-0.019945097	0.96857
TMEM35	0.009316448	0.973402	-0.682735189	0.01726	-0.053339424	0.847544
IGF2BP2	0.13879246	0.173361	-0.243087384	0.01735	0.16101872	0.113547
SLC25A10	-0.439128896	0.051339	-0.534347706	0.01738	-0.885737852	9.05E-05
IDNK	-0.191596604	0.389706	-0.516903624	0.01753	-0.596108324	0.007384
EIF2D	-0.152784717	0.137605	-0.243641291	0.01756	-0.156543849	0.125208
SUZ12P	-0.157015357	0.158637	-0.262305649	0.01757	-0.038846395	0.721593

TP63	0.996461782	0.10879	1.468818148	0.0176	-0.296013942	0.643133
ATP8A1	-0.435799131	0.089817	-0.608516646	0.01762	-0.572382109	0.026146
ADCY2	0.41880681	0.281208	0.907541881	0.01787	0.018312123	0.96241
CKS1B	-0.078507469	0.513597	-0.283880785	0.01807	0.005646055	0.962284
NDST4	1.857080567	0.226621	3.600921901	0.01807	-2.548387904	0.160572
SERPINB9	-0.415353408	0.087898	-0.575447993	0.01804	-0.468400225	0.054078
CTC-499B15.	-0.685912394	0.192793	-1.30356511	0.01808	-1.055000044	0.047925
RP11-17M15.	0.247555248	0.556162	0.936724166	0.01813	0.078163684	0.851765
CCDC171	0.095182355	0.769546	0.76070347	0.01819	0.058903179	0.855902
RP11-83M16.	-0.250086119	0.468853	0.753212185	0.01819	0.461808558	0.152435
FLVCR2	0.160272128	0.766674	-1.30186809	0.01828	-1.348861403	0.014738
RPS23P8	0.037208943	0.882903	-0.596172271	0.01837	0.005398762	0.982945
TSPAN11	0.603973032	0.167182	1.027170105	0.01838	-0.024103506	0.956177
COPB2	-0.040816531	0.604295	0.184814996	0.01846	0.084600991	0.281018
HVCN1	0.206357731	0.699238	1.165719469	0.01853	1.10125982	0.027895
ADCK1	-0.037595788	0.851896	-0.476523085	0.01859	-0.40024248	0.049378
CSF2	1.249173405	0.260393	2.506605984	0.01863	1.083673221	0.327441
PHF20L1	0.060379151	0.631362	0.295380868	0.01868	0.281258397	0.024911
ANO6	0.127252988	0.25917	0.264202906	0.01884	0.097830524	0.384843
PURB	0.109666047	0.250702	0.222963177	0.01882	0.229970272	0.015293
VTA1	0.088931559	0.389115	0.240440758	0.01881	0.124071601	0.226282
AKNA	-0.081846735	0.648568	-0.422022827	0.01887	-0.603903451	0.000795
MAFB	1.058147602	0.173843	1.815193967	0.01897	-0.339104787	0.667367
DOC2A	0.056466111	0.880769	-0.903276943	0.01925	-0.326591085	0.385085
FN1	0.659013628	0.13168	1.023135845	0.01926	0.171042644	0.695604
AP1S3	-0.226880389	0.178731	-0.392354323	0.01937	-0.277835191	0.097444
CYP4B1	0.57998585	0.132892	0.866425668	0.01935	0.760051246	0.041599
F11	0.981929041	0.104021	1.403218372	0.01937	1.459095078	0.014453
FAM171B	0.161730393	0.697247	0.968420216	0.01937	-0.016076092	0.969143
OTUB2	0.156894896	0.432867	0.455387698	0.0194	0.029082859	0.883441
DYNLT1	0.075777655	0.412104	0.213920126	0.01944	0.216000412	0.018037
DFFB	0.257890087	0.190692	0.451860313	0.01947	0.062684355	0.746925
PSAP	-0.240850046	0.065077	-0.304789076	0.01954	-0.384624102	0.003213
SH3D19	-0.045464234	0.733983	-0.311677898	0.01975	-0.106424478	0.425399
CAMKK1	0.360516907	0.088287	0.484629515	0.01979	-0.056055432	0.793813
KPNA1	0.130705384	0.20211	0.237925701	0.01979	0.314699293	0.002006
C12orf29	0.157262215	0.203534	0.285145115	0.01982	0.351836489	0.003868
STAT5B	-0.22768219	0.067792	-0.289569532	0.01986	-0.150756894	0.2232
TET1	0.235839958	0.071466	-0.305890728	0.0199	0.047110451	0.718662
FADS2	0.017815	0.882991	-0.281506004	0.02006	0.028233265	0.815521
FKBP1A	0.064340859	0.638955	0.317469799	0.02002	0.096491002	0.481137
UBASH3B	0.678893521	0.090429	0.930059134	0.02	0.319016321	0.426579
SLC9A3R2	-0.074792454	0.677929	-0.42110913	0.02012	-0.503933887	0.005537
LBH	-0.006845487	0.979751	-0.628349264	0.02019	-0.200536798	0.456906
CTA-313A17.	-0.054574775	0.91189	-1.243713934	0.0203	-0.480071935	0.336272
DDX3X	0.341456495	0.086753	0.461529605	0.0203	0.859913139	1.48E-05
HADH	-0.477394917	0.125391	-0.724046147	0.02031	-0.649213066	0.037374

EGFLAM	0.421344528	0.442802	1.269283434	0.02038	-0.048233698	0.93005
NKX3-2	0.137991239	0.7624	-1.066581937	0.02049	0.150182674	0.741792
CCDC159	-0.257580997	0.148747	-0.411563196	0.02054	-0.327965291	0.062447
IL17RE	-0.418885935	0.083338	-0.558518087	0.02054	-0.454648151	0.059273
TOM1L2	0.310105674	0.066151	0.392582985	0.02053	0.225142644	0.184629
BSG	-0.155682421	0.194633	-0.277859035	0.0206	-0.352604645	0.003309
FABP3	0.57480959	0.053746	0.686734841	0.02062	0.388274549	0.192317
NMNAT1	0.164457271	0.30331	0.364997932	0.02062	-0.03583806	0.821959
CPT1B	0.231125034	0.269692	0.482969097	0.02073	0.339718407	0.103243
RAD17P1	-0.472768809	0.12115	-0.707270567	0.02081	-0.423306563	0.159045
SAA2	0.248420328	0.631469	1.13867424	0.02083	0.486617735	0.33329
ABHD17C	-0.260964736	0.1316	-0.399211014	0.02102	-0.461052485	0.007678
RPSAP57	0.060596643	0.902447	-1.26623737	0.02101	0.033060866	0.945993
RHOU	0.442789134	0.122868	0.660682651	0.02124	0.388629651	0.175549
HMGA1P1	0.254143338	0.408462	-0.732829114	0.02142	-0.094816087	0.758706
MAP2	0.636299434	0.253243	1.276864335	0.02146	0.531730465	0.340167
KRT19	-0.133419591	0.30676	-0.300104567	0.0215	-0.352495036	0.006929
SKP1	-0.103350291	0.168564	0.171431862	0.02161	0.099564269	0.182204
DHRS1	-0.126995159	0.377739	-0.331141272	0.02176	-0.082297933	0.563929
DHX29	-0.280940451	0.069098	-0.353884325	0.02177	-0.220941734	0.151128
MUC15	-0.500726212	0.239404	-0.991305641	0.02177	-1.452492694	0.001006
C12orf73	-0.253094836	0.090008	-0.338514575	0.02185	-0.124659762	0.394294
PHACTR4	0.154518881	0.194146	-0.273588713	0.02185	0.066691204	0.574329
PRKD3	0.164132035	0.237695	0.317519432	0.02201	0.226585646	0.102197
UNKL	0.479096065	0.155538	0.773406897	0.02205	0.161915725	0.631608
ATXN7L3	0.148537334	0.161914	0.2420858	0.0222	0.101818617	0.335974
RHOB	0.031951335	0.887448	0.514538493	0.02222	-0.150892803	0.503734
FAM71E1	-0.152729731	0.708697	-0.960629114	0.02225	-0.508326917	0.2232
HOXD8	-0.541515906	0.214783	-1.003477373	0.0223	-1.015813033	0.020401
PCDH19	0.721928148	0.174584	1.209978065	0.02233	-1.315782052	0.015842
GPT2	-0.241430946	0.158077	-0.389842739	0.02236	-0.619403342	0.000293
SPRED1	0.041189075	0.768397	0.318661109	0.02239	-0.132358022	0.343534
FAM102B	-0.007848735	0.947683	0.27121763	0.02241	0.11402817	0.33782
TPST1	0.193769515	0.238937	0.373595257	0.0225	0.022361789	0.891817
NELFE	0.149916616	0.079622	0.193226751	0.02257	0.161817994	0.055859
ACAD9	-0.177517662	0.112899	-0.252958939	0.02264	-0.167238	0.131286
RPS28	-0.172630266	0.197324	-0.304898896	0.02273	-0.190810494	0.153415
SLC2A4	-0.678355219	0.063028	-0.830779431	0.02277	-0.858048961	0.018659
COX20	-0.014072435	0.884119	0.218264774	0.02285	0.060013245	0.530908
SIX1	1.546494165	0.145967	2.418255504	0.02283	-0.567081015	0.596166
NAP1L6	0.758510648	0.094149	0.998863842	0.0229	0.942752794	0.031645
TNNT1	-0.171062086	0.561663	-0.67289028	0.0229	-0.123837615	0.673469
PABPC1P11	-0.267594608	0.546601	-1.094364822	0.02302	0.149500284	0.721051
UNC13A	1.080171048	0.097944	1.469908154	0.02316	0.796829424	0.223254
CACNA1D	0.093497551	0.755433	0.672971343	0.02318	-0.262491535	0.383007
USMG5	0.050518914	0.612895	0.22518787	0.0232	0.245623829	0.013195
RHBDD1	-0.270466976	0.058082	-0.321789133	0.02327	-0.252287413	0.076045

MOCS1	0.322213444	0.153945	0.493816334	0.02337	-0.006404734	0.977181
RASSF5	0.791841266	0.057793	0.943794456	0.02337	1.471954012	0.000378
ATP1B1	0.289163501	0.087585	0.383399171	0.02347	0.177578044	0.294074
SLC11A2	0.335662725	0.077646	0.430496614	0.02353	0.26502691	0.163429
LIFR	0.087099687	0.757034	0.636625767	0.02367	-0.257880568	0.359744
KLHL25	0.220676691	0.201126	0.385801905	0.02374	0.008833738	0.959138
ANKFY1	0.234920417	0.054667	0.275491846	0.02376	0.182763952	0.133406
CDH26	-0.546499071	0.0991	-0.749029899	0.02386	-0.569858954	0.08499
AK8	0.817987416	0.081779	1.042737639	0.02397	0.323819499	0.498113
ACSS3	0.288418451	0.488884	0.935954335	0.02404	0.053801416	0.897466
HEXDC	-0.116289771	0.495044	-0.385720507	0.0242	-0.043726746	0.794925
LAMB4	-0.984495282	0.066453	-1.215936314	0.02416	-0.260414914	0.608568
PPP2R4	0.103447301	0.325222	-0.236867459	0.02419	-0.153467744	0.143906
RAD54B	-0.405410189	0.082309	-0.522232192	0.02425	-0.043295049	0.850709
LAMA3	0.415687887	0.06889	0.514655581	0.02427	0.103363887	0.651023
APOBEC3C	-0.313445187	0.096822	-0.425134612	0.0243	-0.465073724	0.013673
CLDN11	0.212504884	0.743911	1.461265636	0.02432	-0.769033171	0.23877
CDYL	-0.191636225	0.1518	-0.300443721	0.02442	-0.125759023	0.344509
RNF169	0.102570095	0.131188	0.151180891	0.02452	0.263906957	7.57E-05
RP11-176H8.	0.41470558	0.139648	0.616444524	0.02449	-0.168584573	0.545351
GAPDHS	0.334682931	0.346243	0.771561015	0.02463	0.751062901	0.026105
TMEM164	-0.044234167	0.696171	-0.253781795	0.02462	-0.21507974	0.056121
SH3GL2	0.118657663	0.825266	-1.286923297	0.02466	-0.194714253	0.713938
SLC43A2	-0.033863696	0.902429	-0.621544463	0.02471	-0.518625074	0.060801
CCDC129	0.705448749	0.414948	1.915246592	0.02481	1.221232065	0.160126
C6orf106	0.137161642	0.151657	0.213565315	0.02483	0.216810896	0.02272
PNKD	-0.009002484	0.932262	-0.237843452	0.0249	-0.163044022	0.123791
LHFPL2	0.202614202	0.378951	0.514761787	0.025	-0.085656632	0.709974
C6orf136	-0.319182857	0.116177	-0.458016248	0.02505	-0.565484208	0.005719
HSF2BP	-0.41542287	0.262669	-0.853906933	0.02503	-0.541185687	0.140537
MTMR6	0.035180741	0.730396	0.226761452	0.02506	0.285589844	0.004728
NOX4	0.563336362	0.175888	0.923794533	0.02503	0.361798312	0.38335
RHOA	-0.097041904	0.085538	0.125731198	0.02508	0.142376527	0.011139
6-Sep	0.342011042	0.128226	0.50218429	0.02524	0.143761652	0.522768
P11-254B13	0.658094269	0.129542	0.947038742	0.02528	1.007460908	0.016722
ENPP6	0.878640452	0.09538	1.162381625	0.02552	0.782376197	0.132916
IRF8	-0.710514625	0.07071	-0.873788394	0.02557	-1.080147913	0.005899
METTL24	-0.055918248	0.86898	-0.7861321	0.02561	-0.227581665	0.500384
METTL25	-0.246336609	0.192671	-0.415769641	0.0256	-0.158452452	0.393698
AFAP1L2	0.215024852	0.513126	-0.738773798	0.02565	-0.017738452	0.956992
ATMIN	0.175499504	0.051154	0.200101888	0.02567	0.361643014	5.18E-05
EEF1A1P32	0.135590126	0.763299	0.922822888	0.02568	0.717005152	0.086119
MRVI1	0.308473416	0.327633	-0.707423151	0.02581	0.163541078	0.603673
SNCG	-0.349930493	0.305437	-0.768847447	0.0258	-0.128726099	0.701807
NMI	-0.196816609	0.391188	0.490625273	0.02587	-0.006325605	0.977524
ERN2	-0.002297767	0.993955	-0.677601517	0.02599	-0.339199761	0.264239
ITPR1	0.023135173	0.917862	0.497337716	0.02603	-0.037007022	0.868807

AMT	-0.288583493	0.138218	-0.427341518	0.02611	-0.484762199	0.01206
ETNK1	-0.223088284	0.135599	-0.332331045	0.02613	-0.193417812	0.195266
LAMP5	0.069645037	0.9396	2.025118416	0.02617	-1.965114331	0.037778
KPNA5	-0.172839091	0.237728	-0.323420612	0.0263	-0.03125413	0.828884
TIMM17B	-0.189901856	0.072289	-0.233032907	0.02637	-0.156307908	0.133858
COL25A1	0.627181929	0.483185	1.983142705	0.02649	0.456521256	0.609878
HAPLN4	0.647493204	0.145627	-1.042913543	0.02649	0.206321636	0.644443
PEX11G	-0.604893545	0.104199	-0.846720112	0.02649	-0.29927529	0.397234
ZNF581	-0.115482416	0.522969	-0.401630365	0.02648	-0.377242468	0.036771
RPP40	-0.217722799	0.73582	-1.451717739	0.02654	-0.569012567	0.377343
UBA3	0.024397774	0.792604	0.203706413	0.02667	0.337503455	0.00023
ERI3	-0.181462604	0.123194	-0.259530195	0.02674	-0.1131082	0.330765
PLCB4	0.16897149	0.46468	0.509266022	0.0269	0.486802646	0.034357
PAG1	-0.119725156	0.474373	-0.36972311	0.02709	-0.447048965	0.007552
AKAP1	-0.074845333	0.726451	-0.472274287	0.02716	-0.284310552	0.183468
DPY19L2	-0.1819849	0.410043	-0.48738468	0.02716	-0.13613999	0.531797
PRKCSH	-0.083797623	0.455579	-0.248072538	0.02716	-0.321151994	0.004245
DIS3	0.072748952	0.449829	0.211744086	0.02718	0.35707068	0.000189
GBP4	0.450703669	0.129439	0.64946463	0.02722	0.004511005	0.987969
KCNMB2	-0.918047121	0.102506	-1.247726673	0.02734	-1.166199262	0.038898
RBM25	0.086580067	0.339357	-0.199936815	0.0274	0.024820079	0.783672
RP11-767L7.	-0.591908471	0.136792	-0.889289756	0.0275	-1.007277065	0.013036
DPF3	-0.162310547	0.781808	1.237950045	0.02771	-0.682388848	0.243559
CDKN2D	0.1893955	0.510368	0.614209408	0.02774	0.173397987	0.54167
EFCAB4A	-0.277568498	0.201597	-0.476081168	0.02779	-0.176451887	0.413504
LRIG1	0.031895811	0.8835	-0.479221336	0.02778	-0.308059556	0.15681
SPATA2L	0.37517636	0.064013	0.436692264	0.02785	0.217934875	0.280082
C11orf87	2.476458598	0.126619	3.543182085	0.028	-0.92295998	0.594593
HIF3A	-0.115119083	0.718298	-0.704520077	0.02806	-0.55761901	0.082262
TRIM39	0.144644383	0.182086	0.234697	0.02811	0.300367165	0.004733
RAC3	-0.149117646	0.458776	-0.444801891	0.02822	-0.210621628	0.292903
THSD1	0.337712612	0.436813	0.911729375	0.02842	0.153504517	0.723975
CITED2	-0.084008173	0.671919	-0.434998079	0.02852	-0.209776322	0.289622
CD63	0.042771283	0.677317	0.224829888	0.02855	-0.036143527	0.724973
C1orf95	1.753017769	0.13072	2.52695392	0.02864	0.863637683	0.466457
ZNF177	-0.211896562	0.469536	-0.633240493	0.02864	0.030656034	0.91335
HIF1AN	0.128101136	0.322074	0.282321327	0.02874	0.289872525	0.024626
SYDE2	0.029865147	0.847354	-0.340619746	0.02883	0.109899879	0.476371
WDR55	0.047929491	0.455317	0.13773963	0.02889	0.045977052	0.466696
LRRC37A11I	-0.52867181	0.198014	-0.90995225	0.02896	-0.045690002	0.907948
AK5	-0.487795548	0.302021	-1.049526058	0.02903	-1.112746547	0.019828
LAMC1	0.068221308	0.624506	0.30416286	0.02903	0.14630416	0.293677
TMEM133	-0.139113471	0.356413	-0.329248353	0.02915	0.166587028	0.258072
CNTN6	0.730183504	0.374341	1.764908907	0.02921	-0.111677858	0.892723
NRG3	0.500332356	0.372334	1.215995206	0.02929	0.73595122	0.188103
ST3GAL4	0.260319968	0.229223	0.46535195	0.02928	0.202174675	0.344351
DUSP12	-0.228495113	0.058923	-0.260638768	0.02933	-0.087967686	0.458971

PSMC2	-0.012731097	0.882508	0.186219574	0.02933	0.232100694	0.006513
ACTR3	0.11212165	0.201488	0.190933379	0.02936	0.202274403	0.020901
EBF2	0.70988426	0.475617	2.162679856	0.02938	-0.707439634	0.479234
TUBB8P1	-0.032381639	0.941031	-1.085318689	0.0294	0.038401275	0.927678
DNAH8	1.390071798	0.102493	1.841485196	0.02952	-1.229035577	0.17495
C1orf210	-0.316040749	0.134446	-0.458675269	0.0296	-0.479230406	0.022874
PER1	-0.194339203	0.539275	-0.691953796	0.02957	-0.687877202	0.030177
RBL1	-0.353420713	0.100414	-0.466930629	0.0296	0.039055165	0.855119
MTMR11	0.402861963	0.088688	0.513271426	0.02971	0.411683753	0.081381
SCRN2	-0.313294288	0.065475	-0.367358375	0.02975	-0.410516577	0.0149
ORMDL2	0.273651032	0.059031	0.313341809	0.02977	0.241353437	0.094396
PLAGL2	0.142621083	0.382684	-0.357338368	0.02986	-0.290151658	0.076959
TMEM98	-0.190171344	0.243468	-0.35398407	0.02988	-0.312306403	0.05493
CRLF1	0.450788218	0.391196	1.129327754	0.02991	0.023403056	0.964711
PAQR4	-0.425558175	0.072365	-0.511950244	0.03001	-0.580277895	0.013794
C17orf53	-0.240286415	0.298801	-0.494325458	0.03005	-0.444538073	0.051602
P11-434D12	-0.068133932	0.949447	-2.485957152	0.03013	-0.039268048	0.970774
ABHD14A	-0.372518719	0.056265	-0.419870399	0.03018	-0.573411571	0.003309
ABCC13	0.463942754	0.453574	1.301940185	0.03023	0.211585875	0.736457
AES	0.165292111	0.057773	-0.189132197	0.03024	-0.035837685	0.680484
GOLT1A	-0.157539034	0.530459	-0.547005872	0.03021	-0.329382141	0.189189
ANKS1A	0.122227415	0.25889	-0.235761636	0.03028	-0.087659918	0.41755
RP11-305M3.	0.251392136	0.223953	0.440935515	0.03029	0.498022459	0.014024
CEBPB	0.471916767	0.054985	0.529161287	0.03052	0.161736898	0.511477
KHK	-0.159840136	0.703512	-0.918451902	0.03064	-1.210627947	0.006259
TLE2	0.05968657	0.711953	-0.351186382	0.03062	-0.001532112	0.992429
RNPUL2-BS	0.219823813	0.199961	-0.37236586	0.03081	-0.683072045	7.99E-05
MLC1	0.696260492	0.407679	-1.85956057	0.03084	-0.55825709	0.507218
NPIPB15	0.062065379	0.750513	0.409789517	0.03079	0.411808702	0.02954
RSPH1	0.563559188	0.05856	0.641617457	0.03076	0.415968979	0.162892
S100A2	0.754925473	0.398648	1.912944252	0.03083	-0.51545209	0.570468
TBC1D19	-0.208213286	0.197736	-0.348313861	0.03073	-0.277635889	0.083251
TCTN2	-0.214889804	0.100682	-0.280687192	0.03082	-0.259163447	0.045633
REEP6	-0.100697903	0.669411	-0.510303524	0.03095	-0.672698408	0.004524
MYBPC2	0.884456052	0.137275	1.2538007	0.03108	1.650505692	0.004772
TAF13	0.090303642	0.54073	0.313937478	0.03109	0.13117681	0.369732
AK9	-0.273798068	0.139024	-0.395952255	0.0311	-0.026215955	0.885767
CORO2B	0.932707106	0.135898	1.333275364	0.03116	0.427734199	0.497295
SCG3	-0.247561532	0.459929	-0.753249601	0.03114	-0.076981249	0.810575
SERINC2	0.003723353	0.978304	-0.295235764	0.03125	-0.573948847	2.98E-05
ACSF3	-0.002060437	0.988237	-0.300932237	0.03139	-0.174394465	0.213196
ATP10D	0.188950931	0.290562	0.382101732	0.0314	0.390528383	0.028147
BAAT	1.163402109	0.193837	1.919324659	0.03134	1.59245909	0.074311
GRIP1	0.315689743	0.391527	0.789371167	0.0313	0.475920935	0.196477
PROCA1	-0.291390551	0.283308	-0.596779202	0.03135	-0.237870408	0.376456
YWHAZ	0.114680942	0.144772	0.169171157	0.03135	0.161815841	0.039473
CHAF1A	-0.23880695	0.197155	-0.396848893	0.03156	-0.333494095	0.070268

CUL2	-0.044901864	0.578997	0.171555706	0.03151	0.120112165	0.131934
TRIP12	0.002855239	0.974895	0.194769801	0.03153	0.307614745	0.000679
GIF2-C20orf2	0.012857001	0.967294	0.663546883	0.0316	0.541883798	0.079501
APOC1	-0.091196523	0.682831	-0.480694827	0.03171	0.205828813	0.351987
RPL18	-0.198954209	0.083788	-0.246276576	0.03225	-0.276263907	0.0163
B3GNTL1	-0.167498279	0.646739	-0.783386438	0.03233	-0.653251965	0.073662
EHMT2	-0.151728827	0.294836	-0.309913094	0.03234	-0.163291874	0.258809
LAMA4	0.269915914	0.347392	0.61396806	0.03239	-0.139717874	0.626711
RCN3	0.216225081	0.514676	0.704833331	0.03241	-0.489293756	0.141789
TP53I13	-0.165893889	0.343566	-0.374736839	0.03239	-0.471715121	0.007249
TBX2	0.524998609	0.1863	0.847043485	0.03247	0.137507273	0.729149
FBXO34	0.098978154	0.398613	0.249385606	0.03258	0.243660554	0.036616
RBPMS2	-0.082873273	0.86342	-1.114508352	0.0327	-0.079873293	0.866778
PDE7B	0.912788926	0.172634	1.427890984	0.03289	0.761706818	0.25655
ADRA2A	-0.692899247	0.062465	-0.791197583	0.03299	-0.833896172	0.024587
SLC7A2	0.139797649	0.483882	0.4251867	0.03299	0.104472295	0.600578
COL1A2	0.242453067	0.486676	0.742942731	0.03303	0.043298884	0.901135
CTSO	-0.328412758	0.065582	-0.378391522	0.03314	-0.12246345	0.486852
TNKS	-0.079556676	0.533044	-0.272068253	0.03313	-0.174535437	0.171281
DPH1	-0.180378204	0.260547	-0.341409492	0.03315	-0.280855434	0.078798
CSDE1	-0.018582677	0.779341	0.141056569	0.03322	0.153103575	0.020775
ZNF225	0.13418416	0.380716	0.320962303	0.03325	0.265564335	0.078334
NBR2	0.502783318	0.349534	1.13795781	0.03334	0.406655536	0.448845
C12orf5	0.171578764	0.424629	0.454853844	0.03341	0.398523597	0.061504
CNN3	0.186668778	0.218043	0.322058355	0.03348	0.369236607	0.014764
CCL22	0.344271352	0.355528	0.766706009	0.03355	0.144122484	0.69827
KCNN4	-0.445795734	0.31154	-0.945834239	0.03356	-1.715309163	0.000198
NR1H3	-0.061328741	0.73262	-0.38039841	0.03371	-0.22417715	0.210413
PRKCG	1.216736844	0.100721	1.57653253	0.03376	1.37520914	0.064092
UBP1	0.001685926	0.988901	-0.257090363	0.03374	-0.08510755	0.481271
OSBPL2	0.216466171	0.070705	0.252850156	0.03394	0.234038525	0.049402
BTD	-0.272111058	0.165863	-0.414453048	0.03404	-0.288477001	0.138413
MANSC1	-0.397402372	0.059088	-0.445778663	0.03405	-0.378995185	0.07113
SH3BP5	0.330292434	0.187688	0.528716813	0.03402	-0.092211054	0.713537
SH3GLB2	-0.044032997	0.722265	-0.262240767	0.0341	0.022928912	0.852559
PPP2R3A	0.039421102	0.779552	-0.298546143	0.03413	0.030141721	0.82976
AAAS	-0.037401959	0.741176	-0.240156475	0.03417	-0.006648168	0.952826
GPR180	0.063486721	0.486616	0.191445025	0.03417	0.158008066	0.080282
TKT	-0.219803961	0.146112	-0.320195896	0.03419	-0.355575673	0.018677
KRT18P31	-0.590530242	0.099319	-0.758200525	0.03427	-0.260753607	0.44802
IFIH1	-0.088677413	0.633927	0.386551158	0.03443	-0.049338257	0.78925
RP11-345J4.!	-0.126192157	0.7002	-0.697049694	0.0345	-0.642747032	0.050817
GADD45A	-0.339409885	0.284724	-0.674019474	0.03455	-0.538159862	0.089646
PRKCZ	-0.227732332	0.184577	-0.361054044	0.03455	-0.171519515	0.313957
IWS1	-0.110961757	0.185876	-0.17632069	0.03462	0.075625431	0.361923
TOP2B	0.013540205	0.894056	0.214429382	0.03467	0.250349117	0.013614
NPR1	-0.194345966	0.757456	1.281897765	0.03469	0.764521152	0.212244

SARS2	-0.11068865	0.546871	-0.388451769	0.03477	-0.100949992	0.580354
TUBB3	0.445108163	0.096506	0.563862384	0.03499	0.526018059	0.049241
CYTL1	-0.35503859	0.31127	-0.741329418	0.03504	0.16910062	0.626498
SCG5	0.4642634	0.411852	1.161568085	0.03504	0.290270771	0.608031
SV2B	0.536126565	0.364688	1.238953603	0.03508	0.817638615	0.164323
ADAMTSL4	-0.099379699	0.701649	-0.550711995	0.03523	-0.265820944	0.305384
C2orf76	0.129249356	0.545486	0.431756199	0.03523	0.332403913	0.10667
VPS16	0.202300831	0.103946	0.258918644	0.03524	0.276828639	0.024224
C20orf196	-0.183538619	0.458468	-0.523327936	0.03527	0.284039736	0.223438
FAM192A	-0.131180999	0.126296	-0.179357929	0.03538	-0.016750696	0.843308
CTNNBIP1	-0.180043787	0.16215	-0.268644766	0.03555	-0.3194807	0.012501
TMEM194B	-0.4535474	0.09467	-0.567613526	0.03552	-0.426817678	0.113752
WDR7	0.211175398	0.351737	0.475261011	0.03553	0.502613146	0.026306
APBA1	0.769679015	0.370944	1.762699253	0.03559	-0.3419747	0.706188
FOXP4	0.225764593	0.233945	-0.401648068	0.03561	-0.491787879	0.010003
SFRP2	-0.12310055	0.890657	1.860585821	0.03567	-2.201932223	0.017523
TP53I11	0.112765266	0.427395	-0.299556316	0.03567	-0.295127236	0.038401
ANXA7	0.078294716	0.480375	0.231284696	0.03585	0.097630696	0.375635
BEGAIN	1.077204937	0.085714	1.313278628	0.03585	0.430230771	0.497299
COL11A1	0.333636327	0.497399	1.031453803	0.03579	-0.22993703	0.640162
EPM2AIP1	0.108926494	0.20831	-0.182269614	0.0358	0.131138832	0.127369
NCK1	0.266407698	0.091113	0.328907007	0.03587	0.482909592	0.002044
TAAR3	0.882488578	0.202172	1.404409971	0.03581	0.885103511	0.195112
TANGO2	0.222832629	0.099268	0.278569595	0.03582	-0.074701765	0.579577
GLTSCR2	-0.064854271	0.680133	-0.330050847	0.03597	-0.272193627	0.08382
PCDHB9	0.399507481	0.2372	0.696844245	0.03603	0.214549977	0.524712
RLTPR	0.906656655	0.064064	1.006332112	0.036	0.284792425	0.556171
AGTR2	0.181350784	0.849255	1.960292352	0.03624	-0.904471673	0.352636
ACTG2	0.532257222	0.086619	-0.651335366	0.03636	0.412030973	0.184617
DIP2B	0.155729014	0.12354	0.211004175	0.03642	0.188822576	0.061099
EIF3L	-0.16924549	0.083676	-0.204606473	0.03644	-0.049467066	0.612812
CACNA2D1	0.295193865	0.347426	0.654891609	0.03653	0.010083467	0.974409
ARSJ	0.375378884	0.282887	0.728322832	0.03662	0.099427533	0.776208
NOD1	0.086009709	0.811692	0.741808392	0.03665	-0.301186746	0.406417
DYRK1A	0.099771618	0.205691	0.164033659	0.03681	0.172344534	0.027853
ELP5	0.152741002	0.114031	0.199147619	0.03675	0.203113497	0.031858
ZFAND3	0.226260159	0.068185	0.258585158	0.03676	0.197866197	0.110005
BAIAP2L1	0.154216314	0.250758	0.279515986	0.03686	0.325817797	0.0149
HERC3	-0.437525433	0.07445	-0.510051789	0.03691	-0.357591045	0.144204
GTF2E2	0.105095349	0.428778	0.275062806	0.03695	0.275606117	0.035991
SYT14	0.798641412	0.255782	1.448859861	0.03712	-0.035762285	0.960022
HOXC11	-0.182264707	0.814465	-1.662423689	0.03714	-0.106465258	0.890795
RGS13	-0.162586837	0.855123	1.800919047	0.03721	-0.258330509	0.771144
PMF1	-0.102571225	0.317356	-0.212971632	0.03728	-0.008913874	0.929872
PRSS23	-0.248389956	0.21261	-0.414319095	0.03734	-0.835006654	2.80E-05
MCTP1	0.670841968	0.069049	0.763020879	0.03742	0.106497146	0.773285
TIPARP	-0.297194239	0.176536	-0.457202072	0.03755	-0.140010958	0.523632

MMRN1	0.899165117	0.268324	1.65679622	0.03762	0.842195815	0.294646
TRIM36	0.416161886	0.359656	0.920415029	0.03766	0.296522335	0.50803
TACR1	0.498644519	0.310456	-1.039381012	0.03773	-0.537772178	0.276514
ARFIP1	0.216600091	0.155515	0.31526826	0.03784	0.081545054	0.591566
DPT	-1.430035752	0.050635	-1.513099797	0.0378	-1.512899386	0.03738
HLA-DPA1	-0.785893715	0.089101	-0.948169671	0.03782	-1.042225175	0.028642
GPRIN3	-0.443886919	0.159508	-0.654912596	0.03788	0.193210466	0.538001
LMO4	-0.148535748	0.166066	0.221678217	0.03793	0.022532063	0.833041
CTNNB1	0.128515464	0.178014	0.197827307	0.03804	0.230011432	0.015858
NRBF2P5	-0.278050853	0.623493	-1.268514302	0.03808	-0.264546564	0.634561
AIF1L	-0.222200727	0.418196	-0.563900929	0.03812	-0.528572549	0.051566
GYG2	-0.148583398	0.376306	-0.347232979	0.03819	-0.067375374	0.686429
IGSF5	-0.094177386	0.768289	-0.696112142	0.03847	-0.205263911	0.519556
LDLRAD2	-0.990005258	0.058552	-1.040630668	0.03827	-1.376121718	0.007505
MTHFD1L	0.334691056	0.473269	0.951781149	0.03837	0.315437011	0.495167
SGCE	-0.028129018	0.856404	0.318482013	0.03845	0.391901016	0.010853
SHPRH	-0.109545253	0.4629	-0.307722845	0.03851	0.015711637	0.91569
STK32A	1.161901571	0.11386	1.501160738	0.0384	1.533673395	0.033847
VASH1	0.209620067	0.282067	0.401943975	0.03843	0.083120145	0.669161
GYLTL1B	-0.379327517	0.252213	-0.683634398	0.03857	-0.744384597	0.025265
NCL	-0.073683783	0.580331	-0.27549919	0.0387	-0.081986509	0.538329
JBE2F-SCLY	-0.588789145	0.544663	-2.066538638	0.03874	-0.535875183	0.582229
ADAMTS15	0.915988395	0.244006	1.620440044	0.03901	-0.432105792	0.584005
NDRG1	-0.384064667	0.1916	-0.607043455	0.03902	-0.780765596	0.00794
FAM101B	0.216502755	0.434028	-0.57339155	0.03913	0.177347314	0.521171
UBE2S	0.289656369	0.157125	0.420478538	0.0392	0.421163971	0.038433
C14orf1	0.107006967	0.28688	-0.20771832	0.0394	-0.027489548	0.783848
EBF3	0.397385133	0.690707	2.049833465	0.03949	-0.987905416	0.325536
EXOC1	-0.000911411	0.994069	0.250142792	0.03953	0.103576136	0.394483
KCNS3	0.037657908	0.84107	0.381230861	0.03946	0.259564601	0.161244
NXPE3	0.334174077	0.052611	0.353368434	0.03952	0.31739942	0.064691
RASSF3	0.097141295	0.475468	-0.281469685	0.03952	-0.013350158	0.921433
ACBD7	0.325642966	0.171239	0.484088408	0.03966	0.334458826	0.156375
MMGT1	0.010700278	0.917092	0.208428796	0.03964	0.153189261	0.13049
SULT1E1	-0.05018362	0.913154	0.945427919	0.03968	-0.16027572	0.727558
TRAPPC12	0.018541275	0.916543	-0.365554175	0.03968	-0.075413296	0.670131
HABP2	-0.502399602	0.181482	-0.772689907	0.03988	0.575125165	0.125799
TMTC1	0.156827441	0.473548	0.44614883	0.03989	-0.387025025	0.079113
TRPV1	-0.010063717	0.989365	-1.57064439	0.03988	-0.193769554	0.797521
KDSR	0.084845756	0.535089	0.279333341	0.04001	-0.069909226	0.608371
NR4A1	0.179542017	0.573531	-0.657694919	0.04008	-0.054111546	0.86532
CDT1	-0.445239362	0.073815	-0.509606285	0.04028	-0.714473222	0.004053
GALNT9	0.496078234	0.370277	1.120896038	0.0402	-0.03260416	0.953659
GCSAML	0.146110161	0.72133	0.789745008	0.04029	0.409823105	0.295044
HLX	0.218103019	0.509248	-0.680726063	0.04023	0.04624902	0.888572
MVB12A	-0.154824518	0.274706	-0.289044994	0.04037	-0.388936894	0.005921
TRMT44	0.286360682	0.051771	0.297019784	0.04036	0.192541902	0.183623

ARL16	0.414840537	0.099123	0.51306221	0.0404	0.351412284	0.161381
TRPC3	-0.507497732	0.297357	-1.035816965	0.04041	-0.385401819	0.414334
LINGO1	0.478271238	0.06273	-0.5471449	0.04058	0.312175547	0.224863
RP1-145M24.	0.935741612	0.154633	1.291103622	0.04058	1.120981185	0.077536
P11-466H18	0.143140491	0.302815	0.284107643	0.04055	0.082295246	0.553256
SCD	0.127391949	0.453809	-0.348285634	0.04058	-0.319677461	0.060159
MRPL46	-0.048885193	0.69555	0.250006643	0.04063	-0.03056395	0.803276
MSTO2P	0.040432338	0.931989	0.934103939	0.04064	0.355890106	0.442386
MRPL14	-0.091114131	0.360753	0.199520662	0.0408	0.075232821	0.441801
FRYL	0.183890706	0.079223	0.213703368	0.04085	0.040209584	0.700478
NCSTN	0.040051945	0.753391	-0.26115254	0.04084	-0.12674673	0.320865
PAK3	0.249459024	0.529413	0.80653307	0.04087	0.351376436	0.373757
PDPK2	-0.023337266	0.927192	-0.529708277	0.04088	-0.588375716	0.023132
C1orf35	-0.198619432	0.221727	-0.331284366	0.04092	-0.577105169	0.000394
NAGK	0.150391771	0.45663	0.411107322	0.04098	-0.025288598	0.899963
PCDHGA11	0.377050404	0.705404	-2.201143441	0.04101	0.268440153	0.78731
15orf38-AP3	-0.243600452	0.082023	-0.284541631	0.04107	-0.09310388	0.500131
OXA1L	0.194873439	0.068316	-0.219337612	0.04106	-0.123251318	0.249627
CHRDL1	-0.197741195	0.659582	-0.923946531	0.04122	-0.485208276	0.280168
NRM	0.223908572	0.164979	-0.334644493	0.04124	-0.06393822	0.69271
LRRC4	0.245453143	0.412068	-0.613343208	0.04134	-0.360310207	0.228945
RAB19	0.202668897	0.650222	0.871878603	0.04133	0.140704628	0.750392
PPP4R4	-0.194006784	0.730856	1.081541736	0.04146	-0.171862976	0.753784
GPC4	-0.092422791	0.702773	-0.494734171	0.04162	-0.218110542	0.367259
BBIP1	0.047144308	0.73854	0.285634938	0.04164	0.252241228	0.0726
SLC18B1	0.039310563	0.812378	0.335669939	0.04168	0.521701561	0.001516
FRRS1L	0.274615786	0.388432	0.638209833	0.04179	-0.038749769	0.903386
CSGALNACT	-0.363696174	0.184701	-0.557153745	0.04185	-0.412510963	0.130496
MYT1L	0.651804769	0.143162	0.883695769	0.04189	0.206930996	0.643226
STAT1	0.090000012	0.4471	0.240131373	0.04193	0.382622984	0.001178
STAM2	0.105544108	0.25025	0.185058903	0.04207	0.148669097	0.102382
IL27RA	0.335709781	0.195804	0.523291265	0.04209	0.439637713	0.087892
DTX3L	0.051127214	0.604973	0.199672831	0.04233	0.241962374	0.013754
ARL1	-0.008476272	0.927641	0.188315112	0.0425	0.110238826	0.235598
KCNC4	0.741111484	0.081045	0.860107723	0.04255	0.001287485	0.997582
C1orf174	-0.000917352	0.992359	0.190207812	0.04257	0.1173931	0.210745
CDRT4	-0.421006721	0.40467	-1.066177507	0.04262	-0.767368044	0.131701
COL21A1	1.03353396	0.226713	1.731365	0.04263	-0.95034536	0.267889
BRAF	-0.037269109	0.699045	-0.194913051	0.04272	0.031161055	0.744647
DDIT4L	1.023205434	0.075893	1.1578944	0.04274	0.046802444	0.936867
EFNA1	-0.564208633	0.098039	-0.690708625	0.04281	-0.92706721	0.006589
GALM	-0.295215739	0.075176	-0.334486146	0.04285	-0.46628361	0.004844
TMEM205	-0.276033157	0.093397	-0.331988789	0.0429	-0.385709167	0.018693
LTBP2	0.404042344	0.390375	0.946002986	0.04301	-0.218106645	0.644117
PRKAR2B	0.320549324	0.187526	-0.494167469	0.0431	0.572328939	0.018309
EXOC8	-0.111144377	0.203795	0.171594066	0.04313	0.073848749	0.385137
FERMT1	-0.14297173	0.530416	0.459553629	0.04318	-0.459557275	0.043805

TRPC6	0.595975482	0.392498	1.404200219	0.04322	-1.190178368	0.098776
MTPN	0.080614601	0.252556	0.141594333	0.0434	0.129936056	0.063832
GSDMB	0.199043163	0.338235	0.418272915	0.04351	0.072061179	0.728458
SOAT1	0.149777809	0.223907	0.247538069	0.04355	0.485645257	7.23E-05
SLC16A10	0.494587551	0.180229	0.741735519	0.04366	0.325819897	0.376848
PPP6C	-0.006990156	0.927662	0.1540334	0.04376	0.04569116	0.549011
ZNF605	-0.196091914	0.138732	-0.265850117	0.04385	-0.148349028	0.259607
ASB13	-0.343973771	0.090711	-0.407132643	0.04398	-0.327330219	0.105436
U2AF1	0.260309448	0.202111	0.408580318	0.04404	0.315516484	0.120195
TOR1AIP1	0.004115243	0.976965	0.28570286	0.04408	0.280307794	0.048304
JRKL	-0.13360023	0.259328	0.23029741	0.0442	0.004344382	0.969987
AIDA	0.099272985	0.297255	0.190381007	0.04424	0.169156937	0.073658
LRP12	0.208889307	0.26765	0.376963005	0.04428	0.24791026	0.18481
KALRN	-0.235913425	0.081981	-0.272118087	0.04439	-0.374474208	0.005672
ADI1	0.24156778	0.185914	0.365162972	0.04449	-0.241434227	0.186962
FLRT2	0.42656267	0.236605	0.723955296	0.04452	0.230570263	0.522339
CDH6	0.466868105	0.49025	1.358136742	0.04465	0.907204265	0.179917
TNFSF11	0.659192618	0.333564	1.336735568	0.04464	1.118458541	0.092383
EPS8L2	-0.16406724	0.361579	-0.360733998	0.0447	-0.537929426	0.002764
EPHA10	0.182438173	0.658244	0.80376468	0.04478	0.208502447	0.608806
GNB2L1	-0.212339507	0.061421	-0.227760483	0.04479	-0.156314539	0.168431
P11-927P21	0.609113334	0.088681	0.710533138	0.04477	0.886258567	0.011689
ARL6IP1	0.152787556	0.173777	0.224913138	0.04491	0.247789617	0.027124
AAMP	-0.103584619	0.413912	-0.252820096	0.0451	-0.22920495	0.069392
ADCY1	0.033830601	0.935743	0.832774519	0.04512	0.342705013	0.412165
DENND1C	0.054821359	0.762857	-0.366276899	0.04505	0.03620757	0.841381
PLEKHS1	0.184707137	0.784693	1.299110219	0.04509	0.042283562	0.950111
PROSC	0.020122473	0.859724	0.226414652	0.0452	-0.00194359	0.986277
SLC12A6	-0.197948908	0.211701	0.314901939	0.04522	-0.124739328	0.42915
SPOPL	-0.2244209	0.107792	-0.278832251	0.04512	-0.125560713	0.366491
ZC2HC1C	0.704079093	0.12767	0.924098596	0.04522	0.93378604	0.041148
IL34	1.054651525	0.062151	1.128974681	0.04533	0.886654806	0.119437
ARSI	0.064322869	0.847612	-0.678073765	0.04544	-0.16400121	0.624492
FLI1	0.8230376	0.162257	1.170372834	0.04541	0.036850726	0.950257
GABRB2	0.89584243	0.236534	1.458981968	0.04543	-0.618340548	0.4277
PLCXD1	-0.228687789	0.261399	-0.407498699	0.04541	-0.232097288	0.253743
CCDC38	0.364508533	0.478221	1.008817499	0.04569	0.677496968	0.182029
KCTD13	0.159450024	0.440276	-0.416753584	0.04574	0.064924995	0.752306
SLC9A7	0.396364884	0.474293	1.077404597	0.04586	0.209547893	0.704265
CDC27	0.175519392	0.233665	0.293358649	0.04611	0.413744999	0.004894
WIF1	0.623593726	0.519705	1.91207529	0.04619	-0.925582734	0.347802
NCEH1	0.427978333	0.052419	0.439262824	0.04624	0.619421581	0.004841
WIBG	-0.143570077	0.133896	-0.188942886	0.04637	-0.115950058	0.218404
COL6A3	0.496669113	0.051497	0.507945044	0.04641	0.113418507	0.65656
CDON	0.200461843	0.513606	-0.612022723	0.0466	0.19579059	0.523187
BMP3	-0.167914861	0.583587	0.607822404	0.0468	-0.378019914	0.217152
FSBP	-0.702367104	0.068442	-0.765365968	0.04677	-0.301842968	0.430977

DCXR	-0.01763615	0.916665	-0.335790068	0.04687	0.026847628	0.872466
MAP7D3	0.246736349	0.381658	0.559839417	0.04688	0.620974398	0.026895
PCDHA7	-0.035734006	0.987836	-5.054268124	0.04687	-3.006183023	0.208127
CASP16	0.105226405	0.819473	0.862139322	0.047	-0.342594117	0.459127
SULT1A3	-0.00842786	0.970643	-0.456368221	0.04709	-0.237963871	0.298647
PLCH2	-1.003707806	0.121943	-1.30414946	0.04711	-1.950751303	0.003501
DHCR7	0.041444327	0.797614	-0.320885172	0.04721	-0.306513379	0.058021
PIK3CA	0.181135988	0.26739	0.323013133	0.04725	0.363965233	0.025364
RP11-480I12.	-0.376611451	0.211313	-0.596474092	0.04733	-0.285639981	0.337404
APOA1BP	-0.237716555	0.058327	-0.247818092	0.04738	-0.31517548	0.011658
KCNA6	1.081937681	0.072645	1.185363943	0.04743	0.311540868	0.611607
LEFTY1	0.265745057	0.65199	1.138176025	0.04748	0.809593666	0.161075
HOXB6	0.336065874	0.329406	-0.695208837	0.04755	-0.231728091	0.502902
ARL4A	0.021021192	0.928079	0.456766714	0.0478	-0.551159215	0.018067
ATP9A	0.055849896	0.570062	0.193971223	0.04792	-0.046760628	0.633988
DACH2	-1.213125412	0.114425	-1.502535522	0.04793	-0.704489073	0.33271
FAM151B	0.109919603	0.744731	0.639413125	0.04786	-0.229997491	0.489009
HNRNPA1P4	0.145036197	0.646282	0.622292817	0.04775	0.142831272	0.650478
INPP4B	-0.082664012	0.76874	0.554600463	0.04784	-0.326589843	0.245705
NIPAL2	0.094200188	0.601402	0.35459751	0.04782	0.063684461	0.723253
ONECUT3	-0.096252163	0.737151	-0.568873655	0.04787	-0.187915658	0.511813
TMPRSS15	0.918380351	0.406196	2.154615591	0.04792	-0.436772157	0.708835
ZCCHC12	0.133800822	0.794546	-1.093826269	0.04776	-0.414247595	0.428102
LRRC3B	0.836058378	0.282196	1.498737707	0.04805	0.209747821	0.791268
RLF	-0.157827097	0.076566	-0.175716413	0.04806	0.090700872	0.30554
PARP9	0.15090449	0.233889	0.249479255	0.04811	0.353199992	0.005026
FOSL2	0.002772677	0.990216	-0.44679565	0.04826	-0.506621914	0.025099
NDST3	-0.241923081	0.448033	-0.630939759	0.04826	-0.172155476	0.588245
PCOLCE	0.580655848	0.063116	0.616750637	0.04828	0.053373702	0.864455
NUPR1	0.179293914	0.640176	0.75328915	0.04836	-0.136091722	0.722504
TRAM1L1	-0.402264693	0.181873	-0.595741494	0.04833	-0.446071328	0.133729
DYNC1H1	0.131934786	0.236553	0.219892131	0.04839	0.084373864	0.448869
RAD52	-0.192113717	0.420792	-0.468682139	0.04844	0.149677494	0.52669
MAMSTR	-0.211145471	0.543883	-0.709939724	0.04847	-0.479690133	0.171188
TPSG1	-0.179561251	0.734074	-1.102121119	0.04849	-0.081366323	0.876934
CEACAM7	0.712598935	0.19523	1.083782696	0.04866	0.572283138	0.298233
TGIF1	0.209769088	0.092949	0.244528315	0.04881	0.136984394	0.269733
TMEM26	0.194851681	0.639347	0.814193124	0.04879	0.021705574	0.958368
NFRKB	0.00366771	0.969785	-0.190728542	0.04886	0.055597813	0.563497
PTN	0.262910949	0.454243	0.690944494	0.04901	0.4225832	0.228747
KDM3A	-0.200887185	0.234635	-0.332305522	0.04929	-0.114746436	0.496855
MMP16	0.17588732	0.679603	0.835645605	0.04935	-0.03707477	0.930612
NTRK1	0.443075749	0.657697	1.909955738	0.04936	-0.091468286	0.932226
FAM109A	0.02297794	0.883847	0.30276878	0.04947	-0.171358053	0.273436
PEMT	0.008876568	0.960548	-0.353469629	0.04947	-0.225224253	0.208785
SLC5A2	0.688156943	0.193345	1.020722947	0.04945	0.457096961	0.380916
CLEC3B	1.41059792	0.16567	1.980224212	0.04959	-0.694885236	0.514401

HOXD3	-0.075344434	0.892287	-1.111870147	0.04957	-0.583752933	0.298775
LRTOMT	-0.559629819	0.065888	-0.589975433	0.04958	-0.37864211	0.207174
ISOC2	-0.157276086	0.233691	-0.257890036	0.0499	-0.564362874	2.28E-05
PUS3	0.031541603	0.873401	0.382586129	0.04991	0.329877504	0.090903
UBXN7	0.186149886	0.193414	0.280332706	0.04993	0.26197057	0.066601
ABCC11	-0.075186305	0.928623	2.026690276	0.00738	0.08604663	0.912867
AC018804.6	-2.076606243	0.174805	-4.822643038	0.00467	-2.366627831	0.123431
AC019100.7	-1.33834493	0.064793	-1.966736631	0.00956	-1.362867839	0.055547
AC073150.6	-0.656515679	0.417839	-1.733896528	0.04885	-2.384568364	0.011972
AC079834.1	0.052796801	0.974493	2.761264061	0.04625	1.558506126	0.293072
AC092933.4	1.004889637	0.157849	1.53247845	0.02536	1.335980825	0.052706
ACTBP8	2.292364838	0.103552	3.188592547	0.01729	2.120699082	0.130561
AGRP	-0.660611406	0.482391	-2.795183581	0.0285	-0.001095974	0.998998
ALOX5AP	1.09071114	0.340287	2.386853923	0.02887	-1.039563058	0.437934
ALOXE3	0.947417152	0.57775	3.943460038	0.00818	-0.218037308	0.8999
APOL3	1.331048688	0.075606	1.540264893	0.03667	0.995185414	0.191812
AQP9	0.633858064	0.664227	2.642111329	0.04516	2.331886262	0.079055
BTF3L4P1	-0.336922539	0.617992	-1.623027119	0.02873	-1.577011312	0.03149
CEACAM18	1.517126139	0.261945	2.648781197	0.04133	1.401680136	0.297444
CER1	0.744897681	0.463257	2.300476635	0.01248	1.619322325	0.086206
CLDN8	-2.362865393	0.159118	-5.262891761	0.00551	-4.653325163	0.013312
CLEC2A	-0.587848252	0.274284	-1.218801781	0.02824	-1.93001042	0.00107
CR2	1.233429614	0.083145	2.039595601	0.00213	1.799550927	0.00775
CTLA4	2.769526667	0.267711	5.644887196	0.01894	1.689999875	0.505682
CYP2D6	-3.255632444	0.170893	-5.213440718	0.03068	-4.527335055	0.060551
DLX2	2.598041279	0.072901	3.638651926	0.011	0.818454235	0.584796
DLX6	-0.71239541	0.676524	3.294176191	0.02049	-0.285528735	0.857615
DRC1	-0.542244926	0.548507	2.102104983	0.00984	0.551329783	0.528335
ECHDC3	-1.082164083	0.163936	-1.732830751	0.04316	-0.827188563	0.283967
EEF1GP3	0.052796793	0.975864	2.924863387	0.04929	1.878980952	0.226725
EPGN	3.275142647	0.063367	3.565676128	0.03945	1.384241529	0.45292
FAM86GP	-1.131027853	0.471184	-3.762108059	0.02954	-2.344344546	0.168565
FBXO39	2.105356195	0.266114	3.451625427	0.04954	2.445639536	0.177958
FCAMR	1.545070619	0.109496	1.993251539	0.03711	1.744022948	0.06985
FGL1	1.130810782	0.345785	2.956260956	0.01182	0.406798924	0.739704
FITM1	0.357084263	0.698827	1.715107904	0.03998	1.104970779	0.190794
FRG1B	0.653144941	0.506167	1.87121569	0.04276	-0.57430588	0.588141
FTH1P23	1.126575032	0.230877	1.887994147	0.03573	1.149237947	0.214773
GAPDHP33	2.539388229	0.067782	3.070437943	0.02268	1.782705892	0.214762
GAPT	0.192812952	0.849791	2.812365548	0.00236	0.046909036	0.963019
GCNT6	0.052796787	0.9767	3.123340469	0.04942	2.850570816	0.075609
GDF5	-1.325953681	0.105588	-1.867472532	0.02998	-2.710913548	0.005301
GJA3	0.16674921	0.843672	1.694278024	0.03367	-0.678159656	0.44084
GJD4	0.253465117	0.708296	1.238286464	0.04645	0.322292385	0.624732
GLRA2	-1.162227034	0.105827	-1.604262349	0.03994	-1.199827491	0.089308
GOLGA6A	0.052796788	0.976633	3.070303185	0.04266	3.018657778	0.047951
GRAMD2	1.158396379	0.134934	1.52820142	0.04564	0.778027723	0.332386

GRM1	0.816246865	0.311031	1.632102525	0.03077	1.494552035	0.050449
HEY2	1.481685124	0.065159	2.450512262	0.00155	0.043953171	0.959741
HMGB3P32	-1.74829642	0.160167	-2.627687505	0.03895	-1.417371637	0.210289
HOXD13	-2.833008733	0.077879	-4.236774521	0.01256	-0.070062614	0.963809
HSPD1P2	0.052796779	0.977722	3.484858039	0.03618	2.71717787	0.11149
IL4I1	0.995254063	0.307307	2.955778438	0.00132	1.782903171	0.056998
KCNG3	1.373997685	0.151213	2.277067172	0.01326	0.310825842	0.757969
KRT18P41	2.083179126	0.234961	3.522175226	0.0293	2.290297131	0.178271
KRT8P51	-0.086936263	0.92803	-3.65672555	0.00503	-1.682745484	0.146225
LDHAL6EP	-0.081988947	0.902586	-1.778871691	0.02167	-0.51658109	0.446567
LINC00537	-0.589825751	0.675405	-4.02228193	0.01487	-4.087974197	0.013316
OC1004211(	1.475222272	0.406359	3.582811468	0.0207	1.223003955	0.491209
LPPR1	1.178510036	0.225591	2.642717689	0.00331	0.589582102	0.537802
LY86	-1.481923005	0.235567	-3.746509884	0.00787	-0.726262556	0.52107
MTND5P25	0.645043196	0.650663	2.903137099	0.01645	2.163999837	0.081396
MTNR1A	1.227589038	0.247299	2.267466906	0.02277	2.61328755	0.007848
NEPNP	0.712077835	0.687362	3.192932141	0.03579	0.579525849	0.74162
NHLH1	2.009693003	0.178335	3.498947309	0.00795	1.264591052	0.420708
NINJ2	-0.915283234	0.19439	-1.511412569	0.04817	-2.114387216	0.006913
NPM1P47	2.173481406	0.147669	3.071679559	0.03182	2.582446107	0.075596
NSG2	1.513835572	0.476496	4.428347108	0.02216	3.351768451	0.082255
OC90	-1.033341871	0.49958	3.773460881	0.00368	-0.613960487	0.671485
OPCML	2.934405817	0.110175	4.668542722	0.01149	3.281121485	0.073155
OR10AE1P	1.726950424	0.245125	3.318853961	0.01418	-0.183523739	0.911287
PAX8	0.897736168	0.260466	1.638937357	0.0323	0.992250556	0.211468
PCP2	0.002614848	0.996574	-1.557619582	0.02999	-0.59135114	0.357075
PES1P2	2.069463651	0.165063	3.30748321	0.01349	2.242665475	0.117472
PGR	-0.475387589	0.752247	3.14989357	0.01828	-1.027942374	0.497684
PKLR	0.781911947	0.676917	3.250307532	0.04996	2.342588265	0.174887
PPEF1	1.238277169	0.14316	2.156394301	0.00825	-0.002896974	0.99737
PSKH2	-2.556157793	0.109625	-3.363815799	0.03526	-0.910859475	0.521278
REG3A	1.690013214	0.146628	2.496520465	0.02819	3.225062686	0.004201
RP1-161N10.	2.527335395	0.077961	2.915470217	0.03714	1.476287308	0.329101
P11-1035H1:	4.032194189	0.06954	4.724474022	0.03198	5.244240552	0.016918
RP11-129B9.	2.335979911	0.06745	2.984406517	0.01612	2.227539406	0.079517
RP11-144N1.	0.170719005	0.803812	1.310611204	0.03059	0.950125475	0.124894
P11-168J18	2.535904246	0.069216	2.939958689	0.03053	1.936707697	0.174584
RP11-208P4.	1.291604371	0.118306	1.982618283	0.01268	1.252112191	0.12594
P11-215A21	1.28801817	0.329218	2.42691327	0.04636	2.634403158	0.028905
P11-227H15	2.381804839	0.081004	3.601452254	0.0051	1.173854451	0.42741
P11-349N19	-1.046570456	0.392985	-2.728335394	0.04416	-0.641956719	0.59204
RP11-34E5.4	1.839189515	0.051989	1.902714522	0.04224	2.205055117	0.017097
P11-365D23	-0.19143845	0.854849	-3.218490306	0.01949	0.475371169	0.631992
RP11-375N9.	2.775009233	0.107648	3.322790485	0.04727	1.880327323	0.294837
RP11-3L10.2	-1.022253997	0.065077	-1.568436632	0.00641	-1.074417685	0.048855
P11-404O13	0.609222589	0.603525	2.251561594	0.03373	0.660314187	0.564459
RP11-454P7.	-0.691298396	0.528396	-3.233778193	0.02244	-0.073543975	0.944429

P11-461G12	0.745608264	0.659035	3.059001912	0.03334	1.083255495	0.500623
RP11-54D18.	-1.050492699	0.321508	-2.575552427	0.04998	-2.634198331	0.041883
P11-578F21	2.52794459	0.060413	2.693478269	0.04384	1.434737086	0.299489
RP11-58O9.:	2.350987155	0.062194	2.562770936	0.03891	1.123577352	0.40186
P11-599J14	0.945238121	0.217922	1.846176612	0.01091	1.602431465	0.02828
P11-744O11	-0.431100086	0.399708	-1.17219624	0.03298	-1.553721823	0.006944
RP11-764I5.:	2.125092333	0.134268	2.766260299	0.0414	2.168411652	0.119796
RP11-812I20.	-0.123287704	0.809283	-1.537998384	0.01061	-0.035138748	0.943543
RP11-93O17.	0.770697926	0.566866	2.94461961	0.0187	0.519939558	0.699483
P5-1109J22	1.467908556	0.123095	2.008723715	0.03019	-1.087853448	0.362878
RPL21P39	1.412315583	0.497034	3.901915995	0.03701	3.437220373	0.06841
RPL35AP32	-0.131549164	0.87471	-3.279707188	0.0078	-0.315827136	0.703495
RPS21P4	0.348138853	0.678526	1.56873048	0.04045	1.207173284	0.120098
RPS3P2	0.585967632	0.431544	1.385062557	0.04957	0.573451275	0.434446
RPS4XP6	0.760568358	0.310473	1.748856755	0.01199	0.788669414	0.282688
RPS7P3	2.887594849	0.056415	3.141803158	0.03532	3.285537508	0.026933
RSL24D1P8	0.104777709	0.951442	2.878516424	0.04928	0.407151063	0.804661
RXFP2	-0.261273941	0.877605	3.579111809	0.02393	0.448327482	0.78512
SCOCP1	0.670775779	0.532488	-2.882988176	0.04302	0.056114505	0.959213
SERTM1	0.745540636	0.451978	2.095777027	0.02631	-1.064648826	0.342789
SETP5	-0.756668377	0.394296	-3.055165621	0.01444	-0.353962666	0.673288
SIGLEC9	1.796264726	0.322627	3.397497807	0.04248	2.744508079	0.113248
SLC10A6	2.402369436	0.163967	3.79097223	0.01893	0.497767054	0.789276
SLCO6A1	-0.464605853	0.540722	-2.705207367	0.00355	-0.41916851	0.589247
SPACA3	-0.371208289	0.479924	-1.730585505	0.00576	-1.448494299	0.013997
SPRR2E	2.000959109	0.293182	4.069107453	0.0197	2.835904518	0.115177
SVILP1	0.702455756	0.727506	3.833073922	0.03179	1.260216889	0.499312
TAF7L	1.265338486	0.137007	1.959952186	0.01485	1.623175767	0.04813
TAS2R63P	-0.957815543	0.089064	-1.294582781	0.02423	-0.563538085	0.287993
TCHH	0.05279676	0.979623	4.053535504	0.02769	1.929069398	0.327983
TMEM178B	2.130356295	0.074135	2.504264026	0.033	0.993855884	0.404611
TOMM20L	-1.103347086	0.2215	-1.927334232	0.04536	-0.134376592	0.866111
TPT1P4	-0.033565758	0.965155	1.817291713	0.0065	0.721589905	0.305331
TPT1P5	0.117205832	0.910657	2.02498162	0.02153	1.293419978	0.156647
TRIM60P14	-2.179043984	0.257253	-3.833622385	0.04904	0.840559365	0.619174
WFIKKN2	1.376294176	0.419334	3.431156714	0.02106	2.988852984	0.04631
XCL1	-1.113085397	0.116874	-1.622391222	0.02731	-1.503600885	0.036959
XKR7	1.837647168	0.091668	2.839758505	0.00762	0.507918465	0.655498
ZFAND6P1	0.112378747	0.915788	-3.227416284	0.02176	-0.958095245	0.396738
ZNF355P	0.11714328	0.907169	-3.245391175	0.01887	-1.644228964	0.137549
ZNF648	1.243134052	0.281397	2.96101872	0.00609	0.61664242	0.602527
TPD52L1	-0.409989695	0.060442	-0.304556935	0.15806	-1.147021202	1.75E-07
RNF114	-6.683869548	0.107729	-0.377714205	0.92534	-20.77830528	5.72E-07
TRABD2A	-0.289677062	0.280438	-0.241442108	0.36529	-1.361316373	6.01E-07
VWA2	-0.116070757	0.793266	-0.319463174	0.47093	-2.436859947	7.77E-07
ST3GAL5	0.337855176	0.053261	0.330646362	0.05694	0.840814674	9.09E-07
STARD3NL	0.026928262	0.731157	0.112944601	0.14224	0.361187412	2.05E-06

TM4SF18	0.202572293	0.503187	-0.264342372	0.38628	-1.477195641	2.54E-06
PVRL1	-0.275838536	0.151626	-0.131400148	0.49361	-0.890373158	4.25E-06
FNBP4	0.218817202	0.081459	0.100565487	0.42296	0.573799776	4.42E-06
HNRNPH1	0.140914181	0.175266	0.124407543	0.23123	0.476377569	4.50E-06
ID2	0.219501616	0.201423	0.012251146	0.94312	0.783773568	4.74E-06
VDR	-0.360998488	0.141711	-0.399220542	0.10141	-1.088583727	1.38E-05
GPR56	-0.36018529	0.131001	-0.342800352	0.14951	-1.034337507	1.54E-05
NPNT	-0.407611836	0.127321	-0.10770723	0.68627	-1.160047673	1.59E-05
GSTK1	-0.158919094	0.05947	-0.116125708	0.16494	-0.362940423	1.60E-05
CCNL1	0.162001996	0.203004	0.032593217	0.79775	0.544840733	1.74E-05
DDX5	0.059315626	0.527014	0.152722369	0.10294	0.397546734	2.16E-05
APOBEC3B	0.432769435	0.09016	0.490103042	0.05374	1.043333318	3.39E-05
CLSTN1	-0.135672967	0.09187	-0.127609284	0.11174	-0.329559908	4.24E-05
PPIC	-0.245507193	0.102669	-0.056557578	0.70499	-0.607555388	5.62E-05
C5orf63	-0.504031456	0.216273	-0.094234541	0.81444	-1.676590688	6.18E-05
SNX10	-1.132936296	0.118518	-1.390548506	0.05712	-3.462356681	6.40E-05
ST6GALNAC	0.212109673	0.150651	0.071377034	0.62789	0.580107328	7.48E-05
PSD3	-0.217138715	0.161806	-0.003916458	0.97983	-0.614479004	7.57E-05
RAMP1	-0.39316607	0.112435	-0.286965304	0.24344	-0.987085487	7.56E-05
EEF1DP3	-0.503332409	0.088805	-0.178560478	0.54189	-1.184552395	8.10E-05
IRX5	-0.552703231	0.116296	-0.019797289	0.95396	-1.439425483	8.22E-05
MPHOSPH1(	-0.058312671	0.713568	-0.092354718	0.55915	0.617808308	8.46E-05
ZDHHC12	-0.196674057	0.236672	-0.074402522	0.65005	-0.658877128	8.59E-05
ZNF579	0.026593663	0.908795	-0.248519776	0.28495	-0.935818865	9.05E-05
MCF2L	-0.447659657	0.060806	-0.448428173	0.05858	-0.921490384	0.000111
RSPRY1	0.113916706	0.301327	0.210042796	0.05576	0.421633645	0.000117
NLRX1	-0.312054182	0.084093	-0.048110925	0.78794	-0.694305937	0.000138
SLC6A8	-0.016129473	0.955879	0.028659188	0.92167	-1.112085955	0.00014
SH3RF2	-0.37178301	0.0608	-0.357162335	0.07129	-0.743299999	0.00018
MUC5B	-1.035376004	0.072419	-0.121902368	0.83241	-2.15032817	0.000193
RNPEPL1	-0.248372982	0.125859	-0.222154586	0.17023	-0.60423175	0.000201
GC	-0.001438374	0.997103	0.391928958	0.31828	1.44396698	0.00021
ADORA2B	-0.236047333	0.329622	-0.054852354	0.81884	-0.903950281	0.000238
PMVK	-0.348409171	0.069115	-0.188725386	0.31382	-0.71065057	0.000241
SLC38A10	-0.159835248	0.112664	-0.175107846	0.08112	-0.369624512	0.000247
ITM2C	-0.024747761	0.854982	-0.231058916	0.08787	-0.496320705	0.000248
ILF2	0.018056871	0.832483	0.098425493	0.24781	0.310329868	0.00026
PSMD6	-0.061906733	0.494309	0.13171997	0.14327	0.326023089	0.000268
ME3	-0.261827959	0.110557	-0.220770595	0.17139	-0.59769335	0.000283
VTCN1	-0.2591445	0.361663	-0.333018928	0.24017	-1.043849591	0.000291
GNG5	0.095542409	0.348188	0.10074318	0.32149	0.366476905	0.000297
C1orf172	-0.304013551	0.415842	-0.598257437	0.10978	-1.367276782	0.000302
GATA5	-0.085689666	0.845758	0.152122799	0.72801	-1.655249996	0.000303
SUMO1	0.066901634	0.581127	0.226925875	0.06054	0.435893944	0.000306
RHEB	0.109308828	0.165788	0.090532827	0.24682	0.280182217	0.000307
TCN2	-0.21826658	0.21809	-0.281015096	0.11108	-0.638561469	0.000329
NRAS	0.052313832	0.453142	0.117441302	0.09045	0.248020751	0.000334

LRRTM3	-1.167147974	0.115428	-0.65980109	0.36831	-2.672370785	0.000355
RPS6KB1	0.179727016	0.064456	0.068796079	0.47772	0.343715208	0.000364
CCNJ	0.110782141	0.429577	0.087844924	0.52928	0.492226047	0.000375
ZBTB33	-0.038795637	0.680751	0.006567216	0.94398	0.328653228	0.00039
CLIC6	-0.421538865	0.222926	-0.049944018	0.88503	-1.222484428	0.000419
IYD	-0.514683058	0.085755	-0.373303827	0.20948	-1.054920606	0.000447
PNCK	-0.026511695	0.940999	0.146547577	0.68234	-1.26444101	0.000454
EPHB4	-0.129174552	0.201622	-0.11358951	0.26004	-0.354311402	0.000461
GALNT18	-0.419390582	0.054921	-0.131318981	0.53686	-0.77078963	0.000462
RP11-310H4.	-1.121053315	0.07952	-0.575224275	0.35798	-2.346227064	0.000476
SDF2L1	-0.368642169	0.076338	-0.32665172	0.11159	-0.732637435	0.000479
SIL1	-0.237399503	0.086729	-0.113992991	0.40623	-0.479250288	0.000521
OSBP2	-0.456671568	0.153744	-0.00064048	0.99836	-1.150532738	0.000532
EIF2B2	0.19055702	0.073715	0.13078885	0.21712	0.362945351	0.000554
ALS2	0.080360264	0.401816	0.166426444	0.08131	0.328757248	0.000556
TPX2	0.209153731	0.230123	0.131913447	0.44895	0.59922965	0.000572
PPP4R1	0.154975203	0.146504	0.137482275	0.1962	0.364881639	0.000592
RNF25	0.207180585	0.098737	0.196857419	0.11339	0.421853393	0.000597
HS1BP3	-0.297440754	0.106381	-0.150713923	0.40388	-0.62792072	0.0006
L1TD1	0.525896825	0.222731	0.40131294	0.352	1.474635563	0.000601
ELMO3	-0.183241186	0.178318	0.052343323	0.69185	-0.466805874	0.000621
GGACT	-0.379684398	0.169406	0.228136274	0.3724	-0.933815004	0.000622
TOP3B	0.108296568	0.359227	0.177556252	0.12877	0.397911755	0.000635
LRRC37A16I	0.243097655	0.12291	0.276403897	0.0779	0.535035877	0.00064
HBS1L	-0.000236621	0.997886	0.153091542	0.08431	0.301732726	0.000647
SALL4	0.3103703	0.181162	0.167116781	0.4718	0.787850428	0.000648
NME3	-0.46251148	0.054806	-0.150704299	0.51855	-0.82247903	0.000694
PRSS1	-0.869719435	0.100479	0.122219603	0.8174	-1.793146522	0.000711
WWC1	0.35107013	0.059365	0.091085215	0.62475	0.627714403	0.00072
RBM5	0.116085052	0.21462	0.112030221	0.23018	0.313975968	0.00075
SNAP23	0.178255277	0.155429	0.244792324	0.05002	0.420210179	0.000752
TSPAN8	-0.386129946	0.146204	-0.373530524	0.15975	-0.895585994	0.000754
MGMT	-0.262972743	0.108645	-0.214796217	0.18428	-0.553217546	0.000767
NDUFV1	-0.100656364	0.216957	-0.03029853	0.70749	-0.272721231	0.000768
ZNF207	0.087054276	0.254826	0.023118645	0.76185	0.255207926	0.000807
DDX6	0.182959129	0.061188	0.009381604	0.92355	0.325674069	0.000827
FAM21A	0.020832064	0.854622	0.175782978	0.11856	0.375652807	0.000832
GALK1	-0.189030757	0.299534	-0.291929961	0.10695	-0.619183862	0.000834
FBXO38	0.141057526	0.306602	0.154926028	0.25996	0.458301784	0.000846
XYLT2	-0.131994017	0.318951	-0.213525612	0.10554	-0.442813932	0.000865
ZFP41	-0.293412416	0.371111	-0.194058848	0.55244	-1.123131545	0.000877
COL18A1	-0.276692425	0.281339	-0.034568512	0.89272	-0.855723219	0.000878
ZBTB42	-0.226993102	0.16913	-0.196920066	0.22383	-0.550608023	0.000891
RPL13P12	0.141486435	0.430901	-0.043358535	0.80926	-0.598596165	0.000909
NDEL1	0.146845795	0.155751	0.193845941	0.06054	0.3401959	0.000951
PAIP1	0.039340873	0.686504	0.12949674	0.1817	0.319980504	0.000956
RBM8A	0.026940395	0.791421	0.119660366	0.239	0.334906199	0.000955

PROM2	-0.531455302	0.1016	-0.598743476	0.06422	-1.069285712	0.000982
EFNA4	-0.174822087	0.284328	-0.282662925	0.08216	-0.541903417	0.000988
GPC1	-0.032926751	0.875588	-0.068267183	0.74514	-0.693954197	0.000998
ACOT11	-0.169106567	0.119565	-0.163374522	0.13149	-0.357484258	0.001011
FGG	2.074520554	0.093653	1.050726927	0.39918	4.033222592	0.001013
CRELD2	-0.212245021	0.061728	-0.004930342	0.96459	-0.369687491	0.001037
TSEN34	-0.801801216	0.066999	-0.738161507	0.0897	-1.447812661	0.001045
SQLE	0.016641922	0.878967	0.048637007	0.65612	0.357695051	0.001048
UBA2	0.022733032	0.809243	0.084155873	0.37009	0.306485668	0.001081
KLHL3	-0.330084782	0.114176	-0.290373086	0.1605	-0.680751729	0.001099
MAN2B2	-0.017009597	0.890619	0.022163389	0.85701	-0.403840289	0.001102
PTAR1	0.057059767	0.55516	0.110589058	0.25107	0.313116548	0.001129
ALKBH7	-0.139566267	0.502601	-0.190865856	0.3569	-0.689468616	0.001147
FBXO7	0.106792238	0.126338	0.134578282	0.05257	0.225012857	0.001144
SUPT20H	0.194984206	0.066818	0.153982515	0.1472	0.343413428	0.001173
THEM6	-0.359395401	0.13304	-0.215842041	0.36331	-0.775287231	0.001202
YWHAG	0.122731015	0.113735	0.081600223	0.29235	0.250170833	0.001221
GNPTG	0.053523231	0.645121	0.026550334	0.81771	-0.37601576	0.001259
EFTUD2	-0.045140493	0.672633	0.208198056	0.05051	0.342028067	0.001301
UNC93B1	-0.381821478	0.172078	-0.136589316	0.62382	-0.904733475	0.001353
FEM1B	-0.038155285	0.65253	0.094596035	0.26202	0.269666252	0.001364
KPNA2	0.09572548	0.50356	0.205799405	0.14991	0.456958227	0.001374
CREM	0.076306424	0.557312	0.145382889	0.25731	0.404581674	0.00143
SLC9B2	0.45758961	0.100194	0.488127085	0.07946	0.882045612	0.001442
BEX4	-0.048182311	0.686687	0.194421945	0.10034	0.37470144	0.001476
USF1	0.183294055	0.074604	0.181689262	0.0747	0.322794285	0.00148
NAPRT1	-0.048759655	0.779153	-0.318219082	0.06648	-0.554318128	0.001502
SORBS1	0.04063856	0.799212	-0.246072341	0.12399	-0.508099437	0.001507
FAM120A	-0.133851502	0.177798	-0.113056033	0.25388	-0.31406579	0.00154
SUPT7L	0.144317443	0.136922	0.069102423	0.47467	0.303018366	0.001576
DHX15	-0.112399517	0.107793	0.003265564	0.9626	0.219491006	0.001582
PHF6	0.359911934	0.052096	0.258265541	0.16262	0.582773022	0.001603
VPS37A	0.115353017	0.197521	0.064337928	0.46985	0.278770986	0.001628
SGTB	0.267958352	0.101493	0.301021865	0.06456	0.506891765	0.001655
SLC35A2	-0.185343391	0.201676	-0.12030489	0.40453	-0.456249349	0.001675
MPG	-0.147269948	0.381001	-0.103929119	0.5333	-0.530338474	0.001688
PHRF1	0.071716984	0.709178	-0.167630076	0.38324	-0.60624683	0.001735
SRRT	0.286374345	0.152257	-0.023133714	0.90834	0.621505018	0.001746
WDR25	-0.35082869	0.056013	-0.180860295	0.31988	-0.574246911	0.001762
SMS	0.086498879	0.434026	0.100684297	0.36157	0.344191199	0.00177
MACF1	0.227493126	0.057095	0.172746839	0.14844	0.373331707	0.001784
SIM2	-0.199212189	0.581321	-0.450339134	0.21318	-1.137940184	0.001851
COX8A	-0.230031467	0.127297	-0.146200323	0.33097	-0.468352943	0.001905
FER1L4	-0.539345848	0.10016	-0.604280436	0.06469	-1.018710015	0.001926
ZNF524	-0.10650114	0.631106	-0.091053529	0.67636	-0.695718922	0.001974
TRIM65	0.003219851	0.984634	-0.195609037	0.24047	-0.517518596	0.002
PLEKHA8	0.128878741	0.360598	-0.006714661	0.96199	0.433124198	0.002004

VN1R2	0.556856187	0.144265	0.634664603	0.08969	1.114602637	0.002026
PKNOX1	0.212617505	0.146299	0.18655484	0.19951	0.446879199	0.002029
SERP2	-0.600734068	0.082265	-0.28395631	0.39051	-1.118671329	0.002048
TRPM4	-0.222072386	0.25184	-0.337390263	0.08094	-0.598967874	0.002046
NDUFB7	-0.010777355	0.923237	-0.108190943	0.33184	-0.346014695	0.00206
CYBB	-0.47280434	0.186502	-0.662023572	0.06269	-1.112713557	0.002077
TWSG1	-0.021132617	0.841402	-0.066069769	0.5311	0.323161593	0.002128
CEP95	0.07902685	0.589656	0.171345146	0.24095	0.4456964	0.002147
PADI2	0.739746131	0.146258	-0.40885569	0.42725	-1.625016325	0.002169
HNRNPC	-0.032419795	0.735608	0.048201849	0.61539	0.29395018	0.002174
LEPREL4	-0.261226435	0.127824	-0.292307706	0.08731	-0.525121723	0.002181
CGGBP1	0.130820443	0.166129	0.029762511	0.75208	0.287786895	0.002202
CHDH	-0.300758128	0.070786	-0.171133623	0.30196	-0.509860263	0.002203
HFE	-0.264823822	0.239915	0.018815073	0.93278	-0.69319069	0.002206
HNRNPH2	-0.048763524	0.534043	0.09889854	0.20404	0.237374788	0.002222
RBM48	-0.22764224	0.152118	-0.186538254	0.23515	-0.487484201	0.00222
SLC6A10P	-0.40205163	0.434442	0.061350097	0.90291	-1.723248791	0.002218
SOS2	0.145809261	0.140326	0.170771014	0.0828	0.300279842	0.002217
ASPHD1	-0.209171312	0.436673	-0.193135541	0.46695	-0.854756544	0.002253
OTUD4	0.159382412	0.172067	0.059303325	0.61052	0.354588681	0.002263
DPM3	-0.384931374	0.057321	-0.349483393	0.08021	-0.61726679	0.002272
UBR5	0.2292806	0.139644	0.204304675	0.18775	0.473014339	0.002285
MASP2	0.296876561	0.166101	0.238465812	0.26179	0.628001907	0.002296
ZNF776	0.088781819	0.375367	0.102394113	0.30172	0.300565693	0.002312
TDG	0.051043812	0.582994	-0.002724846	0.97649	0.279233	0.002347
FKBP11	-0.237908844	0.241648	0.300492583	0.13604	-0.617737749	0.002353
NENF	-0.174418392	0.10941	-0.139660021	0.1949	-0.329535209	0.002394
SUB1	-0.018920624	0.82823	0.076844507	0.37695	0.263692186	0.002392
SEPN1	0.092132794	0.553935	-0.246522559	0.11402	-0.475091137	0.002401
TAF6	0.046580697	0.673965	-0.190870629	0.08653	-0.33796054	0.00243
CHPF	0.247651806	0.179813	-0.224332984	0.22551	-0.563084688	0.002432
CDHR3	-0.237506153	0.474374	-0.57310613	0.08531	-1.022794031	0.00244
MOGAT2	-0.933177365	0.159856	-0.454856334	0.46718	-2.154133665	0.002446
SRSF11	0.165420827	0.083758	0.051581466	0.58926	0.288662859	0.002463
ZNF35	0.238399795	0.134805	0.22327316	0.15788	0.466843082	0.002464
CD7	0.863534524	0.222947	1.144316988	0.10583	2.102285489	0.002473
OSBPL11	-0.0417057	0.601825	0.044543759	0.56979	0.233133747	0.002491
ALDH1A2	0.731482785	0.154702	0.164074791	0.74966	1.551206607	0.002519
PPDPF	0.209226936	0.370042	-0.066340963	0.77657	-0.714235599	0.002517
NET1	-0.243063667	0.064048	-0.075475354	0.56471	-0.396263776	0.002524
S100A1	0.726058681	0.104859	0.567962106	0.19869	1.306990311	0.002547
SLC25A11	-0.202491739	0.090027	0.000580437	0.99609	-0.359422427	0.002563
FRY	-0.405247178	0.138642	-0.157103717	0.56539	-0.825573452	0.002573
MAPRE1	0.125164305	0.235451	0.123648713	0.24001	0.316341899	0.002575
ATF2	0.14706762	0.209995	0.153000969	0.1912	0.351429927	0.002612
FGGY	-0.356174798	0.189788	-0.333811233	0.21176	-0.815425097	0.002653
CPT1A	-0.248566146	0.351003	0.301982444	0.25385	-0.801466764	0.002784

DDX41	0.15438673	0.135871	0.040956829	0.69161	0.307767672	0.002788
EPN1	0.203284709	0.209333	-0.233122258	0.15022	-0.485166279	0.002813
TMEM192	-0.228435515	0.088253	-0.19433436	0.14385	-0.39873539	0.002812
ADAM15	-0.223916528	0.288876	-0.242170352	0.24931	-0.6289192	0.002895
CSF3R	0.583035859	0.068363	0.127632137	0.69064	0.944692906	0.002899
GCNT4	0.160478066	0.463326	0.30637347	0.15998	0.646952171	0.002913
SRP19	-0.035616444	0.780819	0.1621639	0.19964	0.374809973	0.002931
CAMSAP2	0.114218312	0.158105	0.089611059	0.26586	0.238608208	0.002937
CYP2J2	-0.367530934	0.202092	0.318385761	0.25924	-0.87343312	0.002998
MAGED1	-0.09439861	0.421835	-0.194558964	0.09771	-0.348640035	0.003011
PPP1R12A	0.125244261	0.405197	0.012660259	0.93292	0.445271795	0.003045
DNAH10	0.624224778	0.147452	0.721120411	0.09582	1.263995503	0.003111
TD-2583A14.	0.15539717	0.566676	0.389863715	0.14259	0.769610145	0.003127
RAB8B	0.094719708	0.562712	0.212816421	0.19313	0.482504205	0.003131
B3GNT3	-0.303235415	0.089313	-0.314489646	0.07778	-0.526860852	0.003154
KIAA0232	0.083488231	0.425659	0.143467735	0.16943	0.30767617	0.003149
PPAP2B	0.28307086	0.138734	0.236225293	0.21631	0.561643881	0.003216
SAMD8	0.153267911	0.174012	0.150719002	0.17889	0.329379743	0.003223
DAZAP1	0.210726481	0.075764	0.22540984	0.05674	0.347740109	0.003257
C19orf60	-0.234109412	0.25536	-0.24879982	0.22783	-0.608733105	0.003264
ANO3	0.764795048	0.222025	0.680865826	0.27673	1.826635946	0.003286
GPR64	0.060286272	0.877807	-0.534139501	0.17446	-1.165111676	0.003289
COLCA2	-0.243172053	0.523157	-0.089770804	0.81243	-1.140377664	0.003313
PPP6R3	-0.073549687	0.348651	-0.00970139	0.90128	0.229196917	0.003314
RRP36	0.013458065	0.886809	0.132156254	0.15677	0.271920287	0.003332
FAM83E	-0.33961053	0.100202	-0.116257077	0.5713	-0.606649633	0.003342
SMG9	-0.199008774	0.072997	-0.113459942	0.30304	-0.323295195	0.003342
NRCAM	0.29056746	0.516036	0.645770044	0.14741	1.300538962	0.003383
KCND2	0.396003817	0.409351	0.208626174	0.66803	1.376283147	0.003433
SMURF1	0.071765124	0.480448	0.112309514	0.26753	0.295881132	0.003435
SCARA5	-0.937961216	0.143315	-1.035459944	0.10837	-1.977918447	0.003454
FOXJ1	-0.524439585	0.257718	-0.313637547	0.49164	-1.401406624	0.003503
SLTM	0.089570857	0.335605	0.179515766	0.05311	0.270401409	0.003495
SPIN1	0.090697259	0.409681	0.169200699	0.12325	0.319936561	0.003507
TBX15	0.187023889	0.394665	0.102321941	0.64054	-0.654178733	0.003515
HAS3	-0.873862487	0.087279	-0.905290362	0.07618	-1.492833536	0.003601
HDAC11	-0.003505747	0.98604	-0.381368441	0.05561	-0.587363706	0.003603
ZNF414	0.295888173	0.348632	-0.606356112	0.0581	-0.943511661	0.003602
PTDSS2	0.192100734	0.191646	-0.173453875	0.23782	-0.428662777	0.00364
P11-1280N14	0.659664243	0.519456	-0.647258297	0.5304	-3.179523895	0.003661
SEZ6L2	0.154807187	0.725507	0.27588262	0.53114	-1.286253691	0.003683
SPINT2	-0.278771319	0.059371	-0.230897263	0.11817	-0.428264668	0.003762
YAP1	0.158032066	0.05684	0.09222663	0.26542	0.23949514	0.003793
LAYN	0.558501455	0.052786	0.535486886	0.06244	0.82010908	0.00383
PPM1B	-0.006392177	0.951736	0.029965856	0.7754	0.302472209	0.00385
TRMT2A	-0.149472931	0.250533	-0.109426842	0.39324	-0.373645287	0.003857
COL9A1	0.249837118	0.412028	-0.524774104	0.08595	0.876422881	0.003877

OSBPL10	-0.209469076	0.238967	-0.243456122	0.16972	-0.51324114	0.003892
SNCA	0.470904401	0.067094	0.119742894	0.64262	0.737518917	0.003892
RAB40B	-0.061470236	0.794033	-0.108105337	0.64511	-0.679117236	0.003911
SLC4A7	-0.038096245	0.774278	0.072997446	0.58163	0.381493551	0.003914
GRM7	-0.684200169	0.175367	-0.131269714	0.786	-1.469925192	0.003947
ARRB2	-0.236835394	0.215803	-0.363825421	0.05555	-0.54951228	0.004037
MORF4L1	0.037900616	0.62268	0.034946312	0.64947	0.220693239	0.004062
GJA1	0.455354944	0.06783	0.478828694	0.05477	0.715583209	0.004091
LANCL3	-0.584643661	0.116668	0.169545252	0.64335	-1.07590973	0.004159
GTF3C5	0.012425178	0.909489	0.177840147	0.10037	0.30930497	0.004179
UFD1L	0.151380944	0.128432	0.140659304	0.1557	0.282146297	0.00426
CA9	-0.618892824	0.096931	-0.699642115	0.06053	-1.065123115	0.004283
ENPP5	-0.446734696	0.087199	-0.069705401	0.78563	-0.746573853	0.004294
ZFR	0.130725837	0.316446	0.05827636	0.65499	0.371859727	0.004302
CCAR1	0.185059223	0.15157	0.150476103	0.24324	0.367358921	0.004328
CLDND1	0.069809282	0.435161	0.001525052	0.98635	0.252608257	0.004333
ZFHX3	-0.121601371	0.664315	-0.317055048	0.25814	-0.800178642	0.004352
RP11-39K24.	1.067190116	0.072759	0.827880055	0.16607	1.620375391	0.004375
PTPN21	0.273832698	0.111213	0.329679041	0.05474	0.487374908	0.004389
POLR2I	-0.083301943	0.48998	-0.13762602	0.24829	-0.341516149	0.004401
SMAD9	0.332159504	0.072787	-0.104680038	0.57283	0.52455673	0.004468
KIAA1199	-0.671380272	0.205761	-0.594279763	0.26263	-1.509427773	0.004485
ITPR2	-0.274615635	0.217507	-0.078488218	0.72411	-0.631826658	0.004523
SLC1A5	-0.149667575	0.330473	-0.143832903	0.34887	-0.43711671	0.004526
SMN2	0.50378155	0.058155	0.20916128	0.43126	0.751253615	0.004532
DNAJC7	0.033016518	0.74312	0.09431366	0.34649	0.283042225	0.004564
SLC39A6	0.300285336	0.091522	-0.045151932	0.79977	0.503617213	0.004582
DEGS2	-0.10649396	0.777101	-0.353075301	0.34687	-1.069134329	0.004628
CMTM7	-0.275831167	0.104376	-0.233902065	0.16585	-0.478242475	0.004709
TMEM52B	0.531624588	0.08354	0.204289129	0.50688	0.862135566	0.004709
TMEM161B	-0.128655189	0.24387	-0.068446063	0.53216	-0.309276589	0.004721
EIF3J	0.188076203	0.115202	0.108724241	0.36073	0.335115467	0.004742
PLA2G4F	-0.636715621	0.089111	-0.63051426	0.08604	-1.076535846	0.004737
PWWP2B	-0.155399089	0.491129	-0.359243989	0.11175	-0.643788916	0.004751
EIF5	0.059067152	0.716308	0.284346093	0.08005	0.458128833	0.004791
ROMO1	-0.124168804	0.312714	0.024196579	0.83941	-0.344171685	0.004815
AMMECR1L	0.038915618	0.604392	0.08327978	0.26064	0.206404824	0.004829
CD151	-0.195836521	0.051184	-0.019781135	0.84305	-0.282396668	0.004826
SLC2A1	0.079564744	0.776423	-0.092069279	0.74242	-0.790037251	0.00483
TMEM184A	-0.121968601	0.508885	-0.16833399	0.35961	-0.520423505	0.004852
ABHD16A	0.162604066	0.13035	0.034302753	0.74773	0.297999171	0.004895
MICALL2	-0.275127498	0.232336	-0.136979324	0.55085	-0.648701995	0.004959
TBK1	0.141323144	0.186984	0.165548744	0.11908	0.296271555	0.004969
RCC6-PGBD	0.175638449	0.349127	0.177291811	0.33856	0.507889741	0.004982
FSTL5	0.360330602	0.302969	0.152174954	0.66383	0.975462475	0.005008
NUPL1	0.112784521	0.165345	-0.024644981	0.76124	0.226689087	0.00505
ANKRD39	-0.239670738	0.170926	-0.222863354	0.19645	-0.486231409	0.005057

TUBA1B	0.228508243	0.119728	0.163644933	0.26513	0.411436244	0.005078
CACHD1	-0.379470871	0.09686	-0.253340053	0.26652	-0.639879317	0.005124
TECTA	0.476131021	0.363194	0.178444779	0.73523	1.437395151	0.005136
CCDC68	0.117066386	0.478708	-0.2445015	0.13936	0.459763238	0.005143
EXO5	-0.186804954	0.29557	0.193537358	0.26983	0.483738377	0.005158
B9D1	-0.295830042	0.085379	-0.096982263	0.56369	-0.481378864	0.005175
CNPPD1	0.071827354	0.547663	-0.219560985	0.06742	-0.336575112	0.005195
P11-343C2.1	-0.130019677	0.387986	0.144182585	0.32901	0.408420932	0.005193
NCLN	-0.073272765	0.549285	-0.070769792	0.56297	-0.342629218	0.005328
RPLP1	-0.012891129	0.932313	0.041988844	0.78189	-0.422829153	0.00534
EGFL6	-2.036834726	0.113832	-1.394511289	0.2767	-3.692502036	0.005377
PQBP1	0.188158322	0.08502	0.143558392	0.18538	0.299847562	0.005367
YAF2	0.170976133	0.321309	0.12370766	0.47063	0.47392969	0.005408
TLK2	0.16938109	0.097209	0.173076336	0.08849	0.281683622	0.005444
HOXC5	-0.568416092	0.186142	-0.346722345	0.40938	-1.230773583	0.005491
AGPAT1	0.105764114	0.394548	-0.194811243	0.11726	-0.345257314	0.005558
PCDHB5	0.201275613	0.517006	0.239654691	0.43911	0.8527744	0.005604
RABEP1	-0.06558902	0.488671	0.041225565	0.6617	0.25986959	0.00563
SSTR1	-0.320903933	0.316865	-0.014497975	0.96333	-0.899816171	0.005685
TRIM5	0.117825914	0.304702	-0.074697024	0.51496	0.314881217	0.005715
TTC21B	0.033359956	0.813909	0.025304151	0.85766	0.388010354	0.005817
CREG2	-0.016409106	0.949648	-0.0621074	0.8075	-0.732036337	0.005898
FEZ2	0.002788918	0.980021	0.042212325	0.70322	0.303756385	0.005893
PDE3A	0.214899433	0.234678	0.156177959	0.3873	0.495664757	0.005955
SLC2A4RG	-0.131269627	0.491988	-0.149333662	0.43339	-0.528878515	0.005972
PGAP2	-0.11555422	0.485871	-0.277009274	0.09237	-0.456205972	0.006008
XRN2	-0.01885664	0.781079	0.033868985	0.6158	0.184560219	0.006051
TBX3	0.991159193	0.053118	0.625628344	0.22209	1.403561707	0.006085
RASSF2	-0.313664268	0.133597	-0.243231443	0.23883	-0.573659216	0.006141
SEC16B	-0.558730065	0.084451	0.138081795	0.65909	-0.895331506	0.006186
BAHD1	-0.068374796	0.589954	0.073896774	0.55659	-0.347606658	0.006236
SPTLC3	-0.35972856	0.174909	-0.403827793	0.12682	-0.724870089	0.006237
CERS4	0.123367523	0.573167	-0.238166176	0.27575	-0.606243392	0.006257
DNAJB4	0.148984019	0.444312	0.239614886	0.21529	0.525870755	0.00626
CADPS	0.877714196	0.052516	0.237702102	0.60062	1.233286171	0.006311
ATP2A3	-0.10292517	0.695537	-0.140058275	0.59425	-0.723765851	0.006325
MSX2	-0.15828632	0.604146	-0.546131425	0.07434	-0.850983914	0.00634
AMDHD1	0.540467677	0.262439	-0.183098913	0.70695	-1.384589379	0.006391
UBE2L3	0.081554259	0.381861	0.112255242	0.22738	0.252833637	0.006436
HERPUD1	-0.381457172	0.188421	-0.508235958	0.07944	-0.789491043	0.006486
TXNRD1	0.148781156	0.302234	0.07045084	0.62506	0.391293944	0.006537
ECD	0.013775705	0.900336	0.03674336	0.73596	0.295159526	0.006581
EBLN2	0.120906877	0.537325	0.197103157	0.30576	0.510800283	0.0066
FAM76B	-0.056166312	0.698452	0.046311579	0.74664	0.387112815	0.006597
SLC17A5	-0.275781065	0.051822	-0.109665649	0.43684	-0.384139341	0.006652
FSD1L	0.096815383	0.564369	0.275687169	0.09401	0.443255269	0.006677
F12	-0.053759672	0.899987	-0.77689194	0.07224	-1.217054803	0.006702

RP11-15J10.[illegible]	-0.531239729	0.100915	-0.156570809	0.61849	-0.880870593	0.006711
FMOD	-0.572878256	0.190423	-0.239846823	0.58272	-1.188460025	0.006734
RBMXL1	0.114177743	0.28005	0.062079457	0.55519	0.282974499	0.006748
GNAL	0.208431417	0.443773	0.107387467	0.69281	0.73127765	0.006769
TIAM1	0.357060832	0.283894	0.609209471	0.06701	0.898789142	0.006802
ANXA4	0.431541968	0.097022	0.285519839	0.27216	0.703225411	0.006824
PCDH20	-0.2552915	0.275453	0.022368737	0.92329	-0.634912484	0.006837
DGCR6L	-0.226720237	0.212124	-0.183085041	0.30924	-0.490773169	0.006889
NUCB1	-0.031400601	0.803781	-0.063213065	0.61662	-0.341690371	0.006885
PUSL1	0.002670704	0.988984	-0.156983571	0.41791	-0.530446386	0.006887
RGS16	0.484170823	0.171304	0.052970146	0.88153	0.947353212	0.006911
CD81	-0.107932663	0.448786	-0.251291052	0.07773	-0.38487396	0.006955
FGB	0.681046322	0.369944	-0.054214939	0.94312	2.044559563	0.007078
DDOST	-0.186385528	0.098314	-0.123864042	0.27146	-0.302958542	0.007179
LRRFIP2	0.140958241	0.185364	0.116797835	0.2705	0.283498979	0.007172
PDZD2	0.395513267	0.085299	0.167682649	0.4676	0.617730483	0.007174
CUL3	-0.063551681	0.483821	0.106704083	0.23612	0.241266661	0.007203
EIF4EBP1	-0.298318382	0.08511	-0.120498976	0.4829	-0.464748504	0.00725
RIMS4	0.670694339	0.394751	0.491674717	0.53021	-2.650995763	0.007283
HMGCS1	0.19498833	0.299264	-0.047290142	0.80124	0.503801157	0.007304
RITA1	-0.07487618	0.590386	-0.023806046	0.86188	-0.374457794	0.007305
UBTD2	0.092945225	0.299556	0.114014027	0.20052	0.237902575	0.007296
7-Mar	-0.048418233	0.579758	0.076174566	0.3817	0.233004008	0.007318
PLEKHG1	-0.367972709	0.086546	-0.13979389	0.51299	-0.574714686	0.007348
SMC5	-0.083296281	0.355843	-0.036679584	0.68319	0.239879028	0.007392
KLF4	-0.182582403	0.545231	-0.307174888	0.30875	-0.808908646	0.007482
TIMM13	-0.129430663	0.372635	-0.220971477	0.1263	-0.387695921	0.007483
GBA2	0.218954338	0.057454	0.106495753	0.35497	0.306778552	0.00751
KDELR2	-0.064620185	0.485808	-0.037661865	0.68399	-0.247108544	0.007613
SYT1	-0.32086002	0.354602	0.321767093	0.34946	-0.926367702	0.007609
SYTL1	-0.424509429	0.096127	-0.412064605	0.10552	-0.682119861	0.0076
C2ORF15	-0.42487373	0.061593	-0.278975028	0.2144	-0.604780062	0.007635
NUFIP2	0.194664599	0.192426	0.145320413	0.33028	0.397253159	0.007764
VPS13D	0.497819117	0.246621	0.550497169	0.19952	1.141085614	0.007781
ABCC3	-0.15086288	0.485522	-0.233900223	0.27943	-0.576268026	0.007805
KIF1B	0.239535746	0.063989	0.124376011	0.33585	0.343377367	0.007806
POLR2B	-0.079906558	0.336077	0.066371895	0.42253	0.219821236	0.007805
SOGA2	0.277378002	0.170486	-0.210973674	0.29919	0.537384913	0.0078
CLDN6	0.178781425	0.391864	-0.129646981	0.53489	0.553199924	0.007876
IGSF1	-0.326383985	0.286858	0.029409613	0.92336	-0.814646097	0.007917
PUS1	-0.238689288	0.197159	-0.21010974	0.25319	-0.490520361	0.007974
SYNPO2L	0.22981495	0.516114	-0.636684039	0.08497	-1.007847698	0.008057
FOXA3	-0.327004072	0.141798	-0.407716582	0.06663	-0.590157749	0.008081
SLC22A15	-0.521224366	0.187892	-0.3923319	0.3094	-1.048693316	0.008079
CCDC51	-0.271439927	0.052744	-0.147340911	0.28093	-0.364848486	0.008278
H3F3B	0.162166792	0.111589	0.131188554	0.19745	0.268536898	0.0083
EPHX3	-0.269750669	0.2935	-0.24166287	0.33953	-0.689315644	0.008319

MFSD10	0.0268937	0.881843	-0.078542455	0.6635	-0.479716807	0.008319
RPS6KA6	0.036632468	0.860094	0.277927164	0.17397	0.535384651	0.008315
PELI2	0.134676749	0.500382	-0.132199472	0.50833	0.525129343	0.008327
ADAMTS20	0.672081091	0.059289	0.532762181	0.13557	0.927129553	0.008334
HEXA	-0.106523437	0.306351	-0.030792014	0.76651	-0.274133827	0.008353
RBM4	0.086879825	0.297649	0.005499639	0.94728	0.218334944	0.008381
CYB5D2	-0.299392056	0.096206	-0.034455719	0.84491	-0.469444794	0.008433
STAG3	0.461157697	0.051165	0.058688941	0.80518	0.613966182	0.008484
TBC1D12	-0.05345574	0.744778	0.203295662	0.21295	0.427641857	0.008614
C20orf194	0.187714864	0.096318	0.191517425	0.08691	0.291788084	0.008627
FBXO2	-0.285145096	0.24853	-0.336411102	0.17228	-0.650117708	0.008669
SLC25A1	-0.023373704	0.871377	-0.127202827	0.37777	-0.379268031	0.008673
NCOA3	0.141142022	0.121983	0.074833439	0.41129	0.238337289	0.0087
HSD3B7	-0.148224622	0.448852	-0.030549873	0.87283	-0.516975796	0.008709
SDPR	0.712301012	0.061495	-0.242761765	0.52539	0.997548557	0.008721
TARS	0.059875237	0.647656	0.158185861	0.22681	0.342975036	0.008721
RBM7	0.153179762	0.227526	0.223685519	0.07694	0.330524267	0.008739
VLDLR	-0.228395275	0.383678	-0.124070062	0.6354	-0.687745452	0.008736
PLCXD3	0.124076434	0.7281	0.143789072	0.68361	-1.003141226	0.008798
MSLN	-0.423336134	0.331657	-0.238785075	0.58213	-1.181278002	0.008826
TNFRSF10C	-0.454792242	0.143951	-0.53877254	0.08383	-0.816149741	0.008837
KRT19P1	-0.282772016	0.183774	-0.079184298	0.70418	-0.557839836	0.008888
CKLF	-0.298803385	0.1142	0.132023734	0.46627	-0.487203614	0.009015
GPR87	-0.531269665	0.216487	-0.36659548	0.38683	-1.143970901	0.009066
CLIC3	0.221294558	0.626743	-0.143568206	0.75563	-1.331412409	0.009097
QKI	0.258066876	0.053511	0.151509549	0.25671	0.347935842	0.00912
BCR	-0.192271674	0.199204	-0.088412301	0.55419	-0.390571582	0.009142
ZNF488	-0.29753386	0.347395	-0.479717351	0.12968	-0.833786965	0.009162
ENAH	-0.127906893	0.337967	-0.001879442	0.98876	0.347115399	0.009192
TRABD	-0.092162435	0.534736	-0.082285025	0.57725	-0.388445912	0.009249
PSMD7	-0.026741255	0.760065	0.083952454	0.33446	0.225497982	0.009282
AC093724.2	-0.389040288	0.178943	-0.443278423	0.12106	-0.76460987	0.009354
PANX1	0.107571893	0.351187	0.201575627	0.07796	0.296018653	0.009343
JMJD6	0.251347063	0.077543	0.270672508	0.05699	0.367286306	0.009365
DCUN1D1	-0.032861466	0.800633	0.235957743	0.06883	0.335864898	0.009489
DISC1	-0.108498617	0.678097	-0.344212586	0.18848	-0.684085087	0.009501
BDH1	-0.371478279	0.125695	-0.329875825	0.17279	-0.63155374	0.00956
TCP1	-0.063383798	0.487823	0.127179051	0.16274	0.235797808	0.009593
FBXO30	0.10955212	0.324953	0.19812385	0.07195	0.283405817	0.009691
DNAJB6	0.044928087	0.657719	0.159350513	0.1147	0.26087398	0.009713
AC026703.1	0.378999855	0.23387	-0.31230502	0.32697	0.821532998	0.009817
MBD4	0.05896424	0.633886	0.172406831	0.15863	0.315208496	0.009813
HNRNPK	0.098000446	0.152733	0.066969269	0.32805	0.176614474	0.009862
SLMAP	0.049368578	0.697306	0.242237759	0.05588	0.326733607	0.009863
RABGAP1L	-0.15228511	0.243594	-0.090412572	0.48764	-0.336124751	0.009948
RIN1	-0.234948261	0.457794	-0.196372804	0.53348	-0.826087227	0.00995
CHD1	0.021426949	0.847038	0.167191675	0.13144	0.285135093	0.009973

CCT5	-0.003995424	0.971671	0.182545203	0.10413	0.289107365	0.010006
CHKB	0.109891263	0.415845	0.252160864	0.06022	0.343924585	0.009991
IQCK	-0.204865847	0.210681	-0.037943754	0.8131	-0.411777491	0.01
C12orf10	-0.071117432	0.519234	0.006673831	0.95094	-0.282764463	0.010033
PNPO	-0.16293865	0.411323	-0.239226859	0.22661	-0.514825776	0.010028
ADCY7	-0.01176829	0.93898	-0.087599642	0.56346	-0.392800786	0.010049
CD164	0.023631341	0.831855	-0.169417224	0.12784	-0.28616045	0.010125
REM2	-0.017804833	0.967503	0.299922431	0.47519	1.038924334	0.010125
SPNS1	-0.007637508	0.949995	0.050449335	0.67641	-0.314509262	0.010179
EVA1B	0.495504892	0.057696	0.183045944	0.48467	-0.693877098	0.010217
PGLS	0.025039839	0.831798	0.052000335	0.65652	-0.303364172	0.010261
RAG1	-0.011189846	0.971505	-0.141140559	0.651	-0.812930271	0.010279
RP4-740C4.(	-0.416870224	0.169862	-0.066447886	0.81572	-0.795069811	0.010297
DBNDD1	-0.578422655	0.09192	-0.351365641	0.2956	-0.880398357	0.010327
PPP2R3C	0.086924194	0.541215	0.247486062	0.07875	0.358793822	0.010342
MAP4K5	0.177827036	0.071517	0.14091017	0.15237	0.251349932	0.010423
RSU1	0.223309008	0.057891	0.172088704	0.14258	0.300240394	0.010433
IQSEC3	-0.71569336	0.078895	-0.603993321	0.12975	-1.030385184	0.01051
DHRS4	-0.047685397	0.789567	-0.048495454	0.78385	-0.458695478	0.010528
UBFD1	0.134302335	0.179063	0.126858719	0.20337	0.254338378	0.010543
DNASE1L3	0.104061542	0.796946	-0.019828222	0.96146	-1.149246792	0.010576
AC018804.7	-0.438204254	0.19686	-0.643011817	0.05991	-0.90148432	0.010595
HCAR1	-0.347088559	0.096586	-0.205302992	0.32242	-0.532970169	0.010634
CDH19	1.394780428	0.050454	1.008900567	0.1552	1.774193203	0.010707
ERCC3	0.091514109	0.413008	0.129365104	0.24437	0.28232058	0.010712
SNRPFP1	1.407744676	0.14991	1.58336237	0.10451	2.478184669	0.010742
PYGB	-0.009877423	0.925938	-0.142362079	0.17954	-0.270708239	0.010773
NRD1	0.160959167	0.26903	0.053060942	0.71505	0.370152823	0.010787
CARHSP1	-0.085811008	0.536584	-0.235989822	0.08919	-0.354056749	0.010805
ZBTB4	0.113081806	0.36953	-0.135693482	0.28205	-0.321928808	0.010898
XYLT1	0.005862459	0.984675	0.073068544	0.81029	-0.77917682	0.010946
TIGD5	0.042891	0.810912	-0.018335106	0.91796	-0.458596028	0.01096
KHSRP	-0.133350896	0.414966	-0.156463449	0.33854	-0.41592032	0.010984
SAMD12	-0.342208443	0.144581	-0.161650531	0.48955	-0.595604768	0.011009
AC004453.8	-0.627204558	0.140952	-0.116411922	0.77077	-1.115467764	0.011029
IGFBP2	-0.208572236	0.239344	-0.18044453	0.30864	-0.450422657	0.011054
KREMEN1	0.192916609	0.407167	-0.401265453	0.08428	-0.594276766	0.011089
OSGIN2	-0.07234455	0.683785	0.277299995	0.11577	0.44527301	0.011137
RDH11	0.017155009	0.875306	0.212365913	0.05162	0.276649295	0.011185
FAT2	0.88247317	0.280804	-0.223445702	0.78637	-2.196112684	0.011224
HMGA2	0.186075872	0.20546	0.043264692	0.76843	0.372413395	0.011204
TRIM2	-0.133722656	0.192304	-0.107772025	0.29245	-0.259683298	0.011279
NONO	0.288647607	0.102116	0.100722567	0.56838	0.446963538	0.011342
MYL12A	-0.059719206	0.482524	0.011100915	0.89587	0.214319954	0.011358
CST3	0.027479462	0.849439	-0.070803741	0.62463	-0.366260626	0.011446
CEP170	0.426830657	0.06746	0.285388293	0.22132	0.589028948	0.011533
BRD2	0.236036643	0.05813	0.136175496	0.27425	0.313847612	0.011676

SMURF2	0.097483618	0.261706	0.165199706	0.05579	0.217154107	0.011683
ERVMER34-1	-0.444576835	0.133209	-0.158384174	0.58712	-0.744489122	0.011769
EXOSC5	-0.478398484	0.062548	-0.477056976	0.06158	-0.646418051	0.011792
ISL2	0.503125029	0.185691	-0.052220967	0.89137	0.934137337	0.011781
TSPYL2	0.165636372	0.286492	0.096104577	0.5356	0.389047875	0.01179
PRPF3	0.105424216	0.250327	0.002024927	0.98233	0.227946676	0.011839
ENTPD4	0.1819943	0.121213	-0.049843766	0.67082	0.294461041	0.011901
P13-104F24	0.425280637	0.324758	0.382271971	0.37116	1.026132523	0.011948
VEGFB	-0.172261436	0.229969	-0.137031763	0.33701	-0.361723961	0.01195
MSI1	-0.051103631	0.892127	-0.31326389	0.40775	-0.954228677	0.012113
G3BP2	0.023931935	0.834319	-0.039053349	0.7327	0.286525458	0.012143
USP42	0.123057041	0.331337	0.2025802	0.10818	0.313853711	0.012143
CRYM	-0.918403466	0.082474	-0.404773467	0.41388	-1.342533111	0.012167
ERBB2	-0.031223073	0.821639	-0.078186336	0.5719	-0.346858199	0.012207
ECSIT	-0.214610552	0.211265	-0.245019653	0.15117	-0.430061333	0.012255
NYNRIN	0.130650077	0.491659	-0.240782365	0.20532	-0.477011385	0.012248
MRPL17	-0.220532703	0.158052	-0.043658297	0.7787	-0.39044104	0.012292
TMEM54	-0.083536707	0.647717	-0.05635271	0.75563	-0.459695537	0.012284
HES6	-0.381306706	0.203342	-0.459132404	0.12249	-0.756183442	0.012309
FAM3A	-0.145596411	0.481931	-0.309430152	0.1343	-0.520337008	0.012383
HLA-DMA	-0.525607456	0.10479	-0.137960248	0.6682	-0.816319867	0.012368
VSTM4	0.028052116	0.909191	-0.056666052	0.81745	-0.621912798	0.012371
SRSF12	0.437121235	0.059809	0.127349404	0.58409	0.577762708	0.012406
DNASE2	-0.300263697	0.14374	-0.135345381	0.50583	-0.511315503	0.012412
SNX3	0.113575491	0.33013	0.188943026	0.10445	0.290625536	0.01242
CIAPIN1	0.11785168	0.340822	0.135017523	0.27216	0.305797922	0.012461
SOCS2	0.669192502	0.051341	0.313890555	0.36133	0.851270335	0.012458
RALGAPB	0.12722919	0.09757	0.045870663	0.54869	0.190465707	0.012503
ESPNP	-0.24085883	0.657178	-0.29650171	0.58474	-1.423525033	0.012581
GLT1D1	0.75573806	0.242824	-0.196131096	0.76654	1.579022107	0.01258
ERBB2IP	0.074888369	0.552856	0.098015875	0.43684	0.314372743	0.012596
HAGHL	-0.692741199	0.125011	-0.729477709	0.10024	-1.111556762	0.012679
B3GNT1	-0.187087484	0.145033	-0.225213412	0.07713	-0.318013328	0.012726
PTPLB	0.079183741	0.379503	0.137503472	0.12507	0.222571035	0.012811
CCNYL1	0.149445249	0.449099	0.243426949	0.21617	0.488304925	0.012829
FAM114A2	0.044771654	0.848674	0.130612121	0.57588	-0.583084372	0.012938
FBXL8	0.077669944	0.831154	0.233118025	0.51522	-0.947829482	0.012945
TDRD7	0.188436181	0.229219	0.284858774	0.06738	0.385803518	0.012937
TTC7A	-0.1548427	0.392703	-0.114900293	0.5217	-0.449194386	0.012924
TMEM86A	0.632860651	0.061721	0.360794495	0.27915	0.820040455	0.013065
ACOT9	0.048875803	0.683405	0.172455706	0.14762	0.293755785	0.013078
SNX22	0.244175541	0.133413	-0.048181093	0.76845	0.395115533	0.013096
TP53INP1	0.130228353	0.266042	0.100291989	0.38974	0.287569328	0.013137
GOLGA7	0.131372254	0.251376	0.006849191	0.95223	0.282090024	0.013165
ZNF439	0.263109637	0.355467	0.147383723	0.60619	0.694133208	0.013207
CDKN2C	-0.514109774	0.194951	-0.361246954	0.35604	-0.987211173	0.01322
USH1C	-0.150535837	0.513455	-0.355111621	0.12295	-0.57078091	0.013237

ACOT1	-0.297079956	0.445424	-0.179453131	0.63948	-1.000337082	0.013254
ZFP36L2	-0.088503377	0.616953	-0.177100653	0.31656	-0.438592584	0.01326
ARRDC4	-0.419257394	0.068234	-0.390829755	0.08898	-0.569219929	0.013275
CAV2	-0.312968128	0.075406	-0.064460686	0.71238	-0.434686573	0.01334
CBWD2	-0.042137478	0.723281	0.18492914	0.1161	0.290164722	0.013364
OSBPL7	-0.164243956	0.480259	-0.103772823	0.65476	-0.574968036	0.013469
PLA2R1	0.220704307	0.497224	0.076230548	0.81452	-0.8096323	0.013538
BX842568.2	-0.652858791	0.200458	-0.105884311	0.82686	-1.310656627	0.013607
POLR2J	-0.19446302	0.129988	0.051938535	0.67832	-0.314402959	0.01364
PIKFYVE	0.156546062	0.214766	0.050091356	0.69094	0.310176001	0.013652
YWHAQ	-0.067524545	0.406459	0.059152973	0.46593	0.199868664	0.013657
STIP1	-0.04511231	0.692735	-0.063996672	0.5747	0.280653518	0.013671
R3HCC1	0.020152741	0.88283	0.03878126	0.77447	0.331198196	0.013735
FKBP10	-0.109333747	0.509941	-0.202535712	0.22212	-0.408915128	0.013744
ZGLP1	0.429896344	0.308532	0.259348567	0.53049	1.001505006	0.013761
DAK	-0.223236362	0.118939	-0.143502361	0.30951	-0.352459824	0.013774
ORAI3	0.152685099	0.297196	0.098981177	0.49736	-0.364549907	0.01379
TNXB	-0.419759765	0.427897	-0.395125848	0.45593	-1.304533614	0.013867
CD99	0.403265594	0.054625	0.19734878	0.34674	0.515291753	0.0139
NRF1	0.142149754	0.338475	0.119316987	0.41529	0.358017972	0.013937
SLC2A12	0.057832628	0.857446	0.524962664	0.09745	-0.810595336	0.013929
ACER3	0.181083176	0.272895	0.305078127	0.06395	0.404686598	0.013956
TOPORS	0.075144547	0.407555	0.147918351	0.0998	0.219915126	0.014069
STX19	-0.814198284	0.070145	-0.045245688	0.91493	-1.10884537	0.014109
PDCD10	0.010626512	0.919602	0.180515478	0.08238	0.254324027	0.014123
SPRTN	0.059914149	0.699471	0.037494597	0.80715	0.375266895	0.01414
GNA14	-0.520139096	0.135421	0.360646426	0.2717	-0.89108123	0.014364
ZNF704	-0.142857021	0.552085	-0.111468948	0.6421	-0.587937779	0.014368
ALG12	-0.216782668	0.169736	-0.092208819	0.55854	-0.38606581	0.014448
PAQR7	-0.035594418	0.811298	-0.066582772	0.65326	-0.366049643	0.014454
CNPY3	-0.168228597	0.088634	-0.158385856	0.10549	-0.239502197	0.014486
TTLL12	-0.118507035	0.47233	-0.30179176	0.06705	-0.402983302	0.014537
NFATC3	0.039483899	0.737944	0.038452227	0.74422	0.28675173	0.014565
GIF	-0.061610319	0.902257	-0.47119323	0.36019	1.178015079	0.014615
RSAD1	-0.277246341	0.071433	-0.262056869	0.08319	-0.371511687	0.014671
FGFR1OP2	0.032826758	0.720971	0.052969961	0.56003	0.219818989	0.014723
HMG20B	-0.146717261	0.225282	-0.15610823	0.19483	-0.294521208	0.014761
AMY2B	-0.225462043	0.448474	-0.148684243	0.61475	-0.724956552	0.014774
ST6GALNAC	0.873681511	0.13438	0.760573487	0.19645	1.399762313	0.014888
TULP3	0.231578299	0.094747	0.225688387	0.10268	0.335678991	0.014905
ZNF641	0.249717738	0.130176	0.193688511	0.23903	0.399726405	0.014882
RREB1	-0.156227447	0.327397	-0.187326032	0.24014	-0.388591577	0.014927
PLGRKT	-0.316713338	0.082369	-0.120079184	0.50151	-0.440151461	0.014947
RPP25	-0.269107547	0.252963	-0.147161553	0.52748	-0.574237646	0.014977
CDH10	-0.305365081	0.277339	-0.008275568	0.97636	-0.685700864	0.014996
ZFAND2A	0.245729505	0.282812	0.005589049	0.98062	0.55075581	0.014997
CNR1	1.239304239	0.10206	1.447500771	0.0549	1.794368779	0.015022

MRPL53	-0.077611663	0.611738	0.009240532	0.95118	-0.369331591	0.015197
ZNF764	-0.13444115	0.434741	-0.18094455	0.29077	-0.41793189	0.015264
RAD23B	0.125002612	0.345124	-0.019747304	0.88135	0.320567169	0.01534
ARG2	0.249331424	0.243165	0.062408458	0.77013	0.511693205	0.015403
EGFL7	-0.029078185	0.903883	-0.202930963	0.39934	-0.584352542	0.015399
KRT7	0.004809896	0.989907	0.131381674	0.72947	-0.92199267	0.015502
C9orf172	-0.009687615	0.965216	-0.251986354	0.2583	-0.54702997	0.015512
C9orf69	-0.104978772	0.372231	0.008192947	0.94368	-0.283482442	0.015518
XBP1	0.038855373	0.875343	0.363516269	0.14174	0.597672232	0.015655
SIGMAR1	-0.271818013	0.090071	-0.223754992	0.1622	-0.38648253	0.015851
CLCA1	-1.889174224	0.139313	-0.031077584	0.98044	-3.110404861	0.015988
RAPGEF6	-0.03540885	0.808685	-0.010466517	0.94285	0.350843472	0.015992
EPHX1	-0.202779858	0.415233	-0.321344957	0.19549	-0.603243464	0.016114
MAP3K5	-0.612823624	0.050248	0.020046164	0.94873	-0.7543982	0.01611
TNFRSF19	0.101090433	0.662549	-0.085265947	0.71302	-0.559403761	0.016196
GPER1	-0.107522093	0.772154	0.353504787	0.32719	-0.930599055	0.016256
MAL	0.044502092	0.957794	-1.434447451	0.10874	-2.297529566	0.016252
HMGCR	-0.009182806	0.946061	0.046287446	0.73299	0.32574003	0.016311
MS4A10	-0.725927637	0.055719	-0.513993284	0.15899	-0.904462011	0.016453
WDR83OS	-0.200125967	0.135622	-0.172680661	0.19566	-0.319737052	0.016755
ARL5B	-0.060567157	0.557725	0.015067742	0.88385	0.246101034	0.016859
PTER	-0.375994627	0.075894	-0.240474508	0.25398	-0.504555642	0.016907
TMEM105	-0.634600606	0.132764	-0.644870179	0.12626	-1.013505796	0.017068
RNF34	0.036653558	0.747348	-0.016188213	0.88595	0.267545232	0.017142
PRC1	-0.01150113	0.945075	0.180662287	0.27785	0.396125069	0.017175
TRAF5	-0.184584141	0.210452	-0.284426818	0.05265	-0.350308477	0.017241
BCL11B	-0.139097615	0.644008	-0.181488797	0.54526	-0.722857078	0.017299
TFR2	0.606670216	0.074583	0.073020633	0.83298	0.790043733	0.017328
SSR1	-0.104123996	0.211586	-0.070331283	0.39758	-0.197742804	0.017472
COQ4	-0.052210253	0.593192	-0.105958585	0.27422	-0.22982716	0.017494
GMFB	-0.034900965	0.682095	0.082489518	0.32955	0.200420271	0.017582
TSPO	-0.003514205	0.991591	-0.189843385	0.56938	-0.802925408	0.017575
FAM172A	-0.189891076	0.15498	-0.021780153	0.86918	-0.315756225	0.01769
TTLL5	0.208310037	0.135904	-0.041012652	0.76878	0.330049831	0.017693
TUBA4A	-0.209404	0.440428	-0.278923918	0.30379	-0.645489687	0.017684
BMS1P8	-0.367612544	0.302848	-0.2752779	0.43132	-0.861135015	0.017799
SLC48A1	-0.069868978	0.722317	-0.075182423	0.70152	-0.471741201	0.017933
PDGFA	-0.082247634	0.717735	-0.154903256	0.49502	-0.54193382	0.017952
C16orf91	-0.21869559	0.224537	-0.068233958	0.69726	-0.42477329	0.017968
CELF4	-0.339809825	0.365727	-0.209845995	0.57084	-0.889808981	0.018032
PPP2CA	0.060702789	0.548168	0.098776329	0.32707	0.237931525	0.018086
PNPLA8	-0.024732599	0.877548	0.032818136	0.83755	0.377513761	0.018119
MCAT	-0.201704987	0.164758	-0.094955336	0.50597	-0.340282744	0.018133
TSPAN31	-0.215087886	0.09511	-0.030573652	0.81149	-0.302430577	0.018155
CHMP7	-0.013730032	0.899721	-0.012576901	0.90735	0.253707494	0.018203
AMD1	0.137564233	0.268161	0.118248603	0.3404	0.292496948	0.018253
CFC1	0.657962273	0.381645	-0.059911269	0.93745	1.737887527	0.018302

CHCHD3	0.007171594	0.943299	0.094387204	0.34636	0.235758234	0.018333
RAB11A	0.070302293	0.470802	0.170006807	0.0805	0.22920996	0.018431
PLS1	-0.281970322	0.053835	-0.047728041	0.74362	-0.344133839	0.018497
SMARCA2	-0.366462501	0.052539	-0.208412361	0.2686	-0.444350799	0.018513
CLYBL	-0.534106211	0.092656	-0.474795415	0.12231	-0.731591763	0.018642
PGBD5	0.170557487	0.603348	-0.140761293	0.66779	-0.777864916	0.018637
XRCC5	-0.029722585	0.809827	0.117305098	0.34203	0.290280332	0.018674
INHA	-0.31833336	0.262398	0.13546683	0.6297	-0.668307451	0.01881
NAP1L4P1	-0.514572712	0.136226	-0.352441452	0.29396	-0.816366326	0.018803
USP7	0.092429855	0.342777	0.117601299	0.22642	0.22812449	0.018852
MLLT10	-0.011818699	0.927437	-0.120388062	0.35223	0.302162647	0.018892
RANBP9	0.164799688	0.063217	0.130332629	0.13924	0.20649887	0.01889
SFPQ	-0.15346052	0.178477	-0.0890788	0.43448	0.267377201	0.018889
IL17D	-0.256212272	0.14017	-0.165663387	0.32731	-0.403846135	0.018963
PAXBP1	-0.136780501	0.181851	-0.083467356	0.4125	0.23753441	0.01895
STS	0.291918738	0.082888	0.292344281	0.08181	0.393484507	0.018989
FNTA	-0.049892729	0.651423	0.203752527	0.06355	0.257224724	0.019026
SEC31A	0.192392309	0.061425	0.109852587	0.28533	0.240912618	0.019023
TRA2B	0.072080421	0.499776	0.101123551	0.34318	0.249736651	0.019062
CYSTM1	-0.194194635	0.38326	-0.139611802	0.53063	-0.522053711	0.019108
ANLN	0.015061252	0.941218	0.159939513	0.43291	0.477587371	0.01913
CBLL1	0.303339755	0.056758	-0.085070708	0.59374	0.371093896	0.019244
CORO1C	0.007105749	0.930843	0.053795147	0.51088	0.190540873	0.01924
HPS5	-0.125450444	0.406425	-0.257316276	0.08609	-0.350948353	0.019251
RXRA	0.122812526	0.307239	-0.049824399	0.67796	-0.28155243	0.019317
AXIN2	-0.401064357	0.547885	0.531472108	0.41854	-1.592711688	0.019392
RP11-438J1.	0.406971745	0.095911	0.389039074	0.1092	0.562412789	0.019448
ACE	0.562490305	0.16748	0.536766384	0.18338	0.924513511	0.019544
AKIP1	-0.290571356	0.072636	0.008804661	0.95583	-0.374292358	0.019698
XKR6	-0.297591388	0.147454	-0.271828397	0.17998	-0.481939246	0.019695
SYNCRIP	-0.069460268	0.547503	0.102034892	0.37626	0.268658524	0.019723
LTK	-0.540080204	0.275726	-0.95289703	0.0584	-1.191648143	0.019776
DUSP3	0.164197587	0.067827	0.141732822	0.11391	0.208594603	0.019829
ATP6V1D	0.090024969	0.356814	0.137484149	0.15578	0.22457034	0.019893
CELSR1	-0.161342587	0.449425	-0.000185666	0.9993	-0.497440568	0.019916
CHID1	-0.304565414	0.077535	-0.161065862	0.34813	-0.400542916	0.020002
ATP8B2	0.31686127	0.059556	0.19950639	0.23497	0.389357626	0.02009
FAM86JP	-0.650076109	0.082483	-0.564873343	0.12173	-0.863974144	0.020199
KIAA1033	0.019860774	0.849039	0.093243791	0.36919	0.240873122	0.020173
ANTD3-TME	0.212168198	0.457175	0.023917323	0.9332	0.657766957	0.020142
RSPH3	-0.069993767	0.616195	-0.151815813	0.27328	-0.323416375	0.020134
SECTM1	0.384484535	0.318316	0.436909833	0.25276	0.876446507	0.020169
WASF2	0.653024224	0.418714	1.281162185	0.11008	1.85610543	0.0202
AIP	-0.182507821	0.126343	-0.100322732	0.39117	-0.274662607	0.020292
HMGN5	-0.13058679	0.682936	-0.335236252	0.29648	-0.758304164	0.020333
H2AFJ	-0.09994633	0.580925	0.079177433	0.66025	-0.419885626	0.020407
RC3H2	-0.024075553	0.769426	-0.040138343	0.6241	0.189204814	0.020396

SMAGP	-0.258296192	0.132311	-0.155599883	0.36085	-0.396320224	0.02045
TMPRSS4	0.122911136	0.576627	-0.179379077	0.41494	-0.51054286	0.020446
PIGZ	0.006634596	0.972159	0.079911534	0.6724	-0.443857777	0.020548
TIMM10B	0.083321823	0.401873	0.015467975	0.87593	0.227811364	0.020596
HSPB8	0.579991959	0.164819	0.106279424	0.80001	0.95957152	0.020607
SLC24A1	-0.095462938	0.635868	-0.291130144	0.14697	-0.46499845	0.020618
PSMC3	0.052387142	0.618573	0.182531871	0.08137	0.24196934	0.020752
DMD	0.350625139	0.112782	0.078250128	0.72366	0.509558217	0.020907
OCIAD2	-0.304010069	0.1148	-0.117537862	0.53802	-0.443194353	0.020892
UGT2A3	-0.54270915	0.117777	0.281993479	0.41271	-0.800567335	0.021014
SBNO1	0.02460165	0.732296	-0.006763403	0.9248	0.16448291	0.021227
TLE1	0.291114792	0.060215	0.178963868	0.24737	0.355984068	0.021221
PURA	-0.139140831	0.262907	-0.014683489	0.90519	-0.284958704	0.021352
DUSP18	0.061033978	0.821278	-0.059203419	0.82582	-0.634593776	0.021407
ARFGAP2	-0.113628893	0.207272	0.133415663	0.13435	0.204516945	0.021451
LMAN2	-0.11520671	0.364058	-0.05577919	0.65971	-0.291627133	0.021464
SYT17	-0.356615212	0.271957	-0.145087657	0.65331	-0.752894085	0.02151
P4HA3	0.222141151	0.691394	0.103777319	0.85111	-1.31704776	0.021585
LSR	0.025542072	0.880272	-0.009227062	0.95659	-0.389870328	0.021609
TRIM68	-0.228274337	0.111501	-0.140124862	0.32384	-0.327737566	0.021761
TTLL1	-0.283634629	0.157755	-0.121653681	0.53649	-0.461058648	0.021775
WDYHV1	-0.145081593	0.430208	-0.15377033	0.39738	-0.417397585	0.021782
FGL2	-0.021832255	0.950565	-0.375762365	0.29215	-0.845844455	0.021918
YIPF2	-0.12812036	0.409197	-0.084541655	0.58469	-0.355982663	0.021947
ZNF404	-0.295843577	0.310557	-0.069693478	0.80571	-0.665836664	0.02194
CDK18	0.082128231	0.74537	-0.399703602	0.11819	-0.586817245	0.022016
ONECUT2	0.526125659	0.341546	0.333301982	0.54679	1.266053568	0.022042
CPSF6	0.000112857	0.99903	-0.017890909	0.84679	0.211520709	0.02209
NDUFS6	-0.219798811	0.127162	-0.110070273	0.43946	-0.327504309	0.022081
PRKCDBP	-0.402324478	0.193389	-0.017182614	0.95493	-0.710580803	0.022067
PHLDA3	-0.148585633	0.366066	-0.060374809	0.71268	-0.376321632	0.022101
HSD17B4	0.119731858	0.484491	0.300068433	0.07944	0.391402407	0.022124
POLB	-0.085021228	0.557099	0.097598624	0.49521	0.324983408	0.022194
RNF212	0.005986072	0.985484	0.310361031	0.33763	-0.767278405	0.022186
ACAT2	0.101042861	0.563522	0.167872537	0.33671	0.398640625	0.022398
FBXW4P1	-0.4000429	0.310938	-0.127019206	0.73653	-0.934087774	0.022469
MKNK2	0.019176189	0.924669	-0.182827591	0.36739	-0.463260443	0.022464
KLLN	-0.104853092	0.556476	-0.00022377	0.99897	-0.409663983	0.022571
PTPN23	-0.066967049	0.787354	-0.187797428	0.45025	-0.568606659	0.022612
CBR3	-0.393273504	0.390049	-0.401025704	0.37477	-1.08544487	0.02275
PSMA3	0.064694257	0.529804	0.142957177	0.1627	0.232829267	0.022777
OAF	0.254602813	0.065535	0.080886176	0.5577	-0.319066103	0.02289
RBM12	0.187354539	0.25708	-0.063738977	0.69971	0.375518704	0.022901
DYNC1I2	0.135275441	0.081067	0.094540256	0.22095	0.175323674	0.022981
TSPAN7	0.43787911	0.392025	0.561867119	0.26523	1.125038111	0.022984
BNIP3P1	0.944591512	0.292374	0.970774074	0.27872	2.027702732	0.023041
IL10RB	-0.241737479	0.189112	0.094925262	0.60274	-0.417675845	0.023034

KCTD3	0.099877486	0.446743	0.230356511	0.07833	0.297001485	0.023138
EPB41L5	-0.055508513	0.696682	-0.020997968	0.88258	0.321821729	0.023226
IMPAD1	0.046702954	0.782506	0.258494052	0.12646	0.38387369	0.023221
TRPT1	0.020565289	0.904233	0.057903024	0.73235	-0.386930129	0.023238
SHKBP1	0.34470748	0.051368	0.065375746	0.71212	-0.403519521	0.023272
TRAV8-5	0.054229468	0.812254	0.097926188	0.66453	0.502253301	0.023447
C16orf87	0.107752227	0.467121	0.014810347	0.92022	0.331962103	0.023478
TFAP4	-0.081759599	0.699925	-0.290012404	0.17051	-0.480603803	0.02347
MFAP1	-0.028129758	0.790578	0.018931155	0.85735	0.237033814	0.023525
FKBP8	0.125820565	0.233443	-0.102083494	0.33383	-0.239647099	0.023585
GBP6	-0.426513152	0.325119	-0.458201562	0.28569	-1.007835705	0.023642
KCTD2	0.219057215	0.132846	0.107920921	0.45771	0.327485233	0.023643
MITD1	0.020907064	0.860262	0.215046426	0.06686	0.264394273	0.023649
GNL1	0.224289386	0.164391	0.15249034	0.34306	0.363598138	0.023732
C14orf80	-0.35270071	0.187777	0.085321388	0.74463	-0.605377715	0.023856
CHD7	0.44626051	0.067974	0.309329396	0.2058	0.552019994	0.023854
RP11-313J2.	0.267137349	0.408075	-0.213778169	0.50799	-0.729318892	0.023841
AKR1E2	0.017235055	0.963147	-0.592531654	0.14427	-0.924033623	0.023869
PARP6	0.176129326	0.20946	-0.06373765	0.64955	0.315486724	0.023947
ALG9	0.035706242	0.809933	0.127693786	0.38761	0.333070721	0.02398
CBX8	-0.148149209	0.541547	-0.455441912	0.05781	-0.540504337	0.024003
MAPK8	0.096791189	0.420368	0.171962642	0.15057	0.268680628	0.024082
C1D	0.193782992	0.36051	0.128517664	0.54213	0.474015998	0.024147
CCDC7	-0.531984238	0.074559	-0.202138016	0.49178	-0.671463582	0.024227
DMTN	-0.303889698	0.243588	-0.314803806	0.22603	-0.587775571	0.024191
DDX1	0.01133763	0.917078	0.07831196	0.46878	0.242282127	0.024332
PCDH7	-0.037008957	0.864019	0.079155073	0.71369	-0.486607808	0.024356
C11orf58	-0.040362485	0.668728	-0.023181065	0.80523	0.211164684	0.024381
GAS6	-0.418608534	0.074657	-0.269565992	0.24634	-0.525778757	0.024432
ZNF143	0.112848945	0.390622	-0.063283366	0.62976	0.292908952	0.024413
PIP4K2A	0.072095886	0.665679	0.076639609	0.64674	0.373784546	0.024485
TMEM51	-0.026803794	0.882676	-0.105023653	0.56187	-0.410313523	0.024563
KLF3	0.165437271	0.104017	0.156896578	0.12265	0.228226186	0.024655
FBXO44	-0.072630581	0.700044	-0.042616829	0.81963	-0.425052875	0.024673
RAB3IP	-0.035330646	0.82678	0.22010839	0.17081	0.360227455	0.024733
TSPYL1	0.122026526	0.142725	0.093871263	0.2584	0.186033102	0.024833
PGBD4P7	-0.066130351	0.784789	-0.222122768	0.35844	-0.557308764	0.024892
DENND5B	0.19992665	0.361517	0.114694973	0.60086	0.485408082	0.025018
EEFSEC	0.069827884	0.678242	-0.135721239	0.41967	-0.385878144	0.025014
TINAGL1	-0.19453676	0.281179	-0.243076615	0.17785	-0.404275155	0.025113
WHSC1L1	0.110705785	0.267708	0.081903375	0.41119	0.222820828	0.025125
SLC25A20	0.127298749	0.525634	0.077591001	0.69801	-0.451065272	0.02515
DOHH	-0.203813552	0.504212	-0.282920694	0.34923	-0.691168309	0.025256
HNRNPM	0.165425231	0.247749	0.145342536	0.30962	0.31979137	0.02528
NF2	-0.126190145	0.162185	0.014733527	0.86993	-0.20168116	0.025338
FAM69C	-1.126506291	0.145122	-1.42765888	0.06439	-1.793775019	0.025395
SULT1B1	-0.203691532	0.317851	0.042721125	0.83391	-0.455682377	0.025454

LMX1B	-0.36114659	0.489742	-0.861223333	0.1058	-1.211687183	0.025659
PFKL	-0.023781778	0.821877	-0.087980781	0.40446	-0.235682996	0.025649
NPTX1	0.024678155	0.973275	0.048027309	0.94795	-1.662296482	0.025748
UBE2J1	0.010703274	0.906199	0.163038085	0.07096	0.201092594	0.02573
FAM3D	0.397255994	0.469557	0.829268797	0.1274	-1.29594285	0.025806
SATB1	-0.139026169	0.457479	-0.337720275	0.07127	-0.417339303	0.025805
HARS	0.045724008	0.593633	0.110653052	0.19411	0.18949363	0.025821
ARL13B	0.254044479	0.118664	0.036834632	0.81971	0.3578552	0.026029
KNDC1	0.113819343	0.834284	-0.719559241	0.19096	-1.22820299	0.026019
TAF5L	0.031653123	0.741562	0.140049318	0.13975	0.209802427	0.026074
TRMT61A	0.003598519	0.981325	0.159294977	0.28997	-0.344619381	0.026076
ICAM3	-0.447480745	0.064797	-0.26762009	0.2592	-0.535519688	0.0262
POLR3G	-0.29397507	0.367365	-0.550558473	0.08808	-0.719045142	0.026251
GATA6	-0.407449363	0.057074	-0.364357461	0.08806	-0.474852052	0.026282
GNPTAB	-0.145685326	0.149256	-0.089087671	0.376	-0.223828909	0.026263
RELN	-0.030786626	0.971637	0.069817573	0.93605	1.917704344	0.026283
LRAT	0.293903783	0.409969	0.177063754	0.61747	0.783434095	0.026324
REPS2	-0.412312246	0.095626	0.177521395	0.46956	-0.550263962	0.026331
SIRT3	-0.264002407	0.111943	-0.147710037	0.3663	-0.366922025	0.026355
GAA	0.014792165	0.957312	-0.306558824	0.26663	-0.61473968	0.02644
RNF43	-0.3993084	0.151825	-0.173142272	0.53326	-0.617540131	0.026595
SDR16C5	1.205193818	0.057698	0.2763846	0.67172	1.401176509	0.026611
PHLDB2	0.282915401	0.183276	-0.138242474	0.51571	0.470659987	0.026665
CDK13	0.185733571	0.080977	0.149055214	0.16132	0.235030472	0.026829
CTSD	-0.019187663	0.897179	-0.277463181	0.06177	-0.328529644	0.027054
NEB	0.243480597	0.444392	0.473188507	0.13112	0.692777355	0.02705
TMEM161A	0.261501805	0.146256	-0.066072717	0.7133	0.395646595	0.027053
PGM1	-0.089305084	0.388378	0.051773574	0.61565	-0.228408187	0.027229
SMIM13	0.044141146	0.764124	0.155529208	0.28784	0.322110824	0.027285
GLTPD1	0.130394813	0.468896	-0.104878805	0.56236	-0.404258944	0.0273
ALDOC	-0.036223599	0.870775	-0.12566951	0.57237	-0.491835271	0.027324
RABEP2	-0.16093541	0.340289	0.060977645	0.71419	-0.374014021	0.027416
NUP153	-0.013260156	0.906593	-0.088008946	0.43558	0.247938936	0.02754
PUM2	-0.222091374	0.066238	-0.087620025	0.46739	-0.265281354	0.027767
SELT	0.126165065	0.379001	0.14165281	0.32194	0.314531379	0.027758
VANGL1	-0.14431941	0.30617	-0.036701445	0.79316	-0.309828688	0.027785
PP13439	0.219914116	0.460036	0.076238334	0.79748	0.633795539	0.027879
PTPN12	0.265712784	0.12048	0.237240688	0.165	0.374817272	0.028085
KIDINS220	0.020093721	0.836039	-0.043016738	0.65719	0.212379343	0.0281
ACO1	-0.18016119	0.085108	0.060398405	0.56174	-0.229066018	0.028212
DNAJB12	0.06400539	0.438531	0.092190798	0.25819	0.177807832	0.02814
FZD7	0.259677245	0.110692	0.162418798	0.31819	0.356831986	0.028116
GLS2	-0.115836198	0.773251	-0.484233093	0.22517	-0.888790422	0.028206
PLEKHG4	-0.067386743	0.863951	-0.049860621	0.89846	-0.872524165	0.028198
RAB1B	-0.286241563	0.325165	-0.408785411	0.16	-0.638776537	0.028196
TIMM22	0.15880955	0.215054	0.072227806	0.5694	0.270204376	0.028279
MUS81	0.186173206	0.150816	0.138791373	0.28234	0.282714213	0.028308

ALDH1L2	-0.318276403	0.333374	-0.359829016	0.2712	-0.727627982	0.028343
EPB41L2	0.235605513	0.124817	0.024954617	0.87079	0.336082805	0.028339
MAPK13	-0.140056002	0.255395	-0.14056139	0.25272	-0.269535014	0.0284
SLC17A9	-0.221663559	0.280383	-0.30326269	0.13933	-0.449284536	0.028418
TSLP	-0.837191069	0.422113	0.177662653	0.86318	-2.310571497	0.028424
GGA3	0.072257767	0.672583	0.163918246	0.33854	0.37342422	0.02847
SERGEF	-0.199854893	0.25047	-0.103188444	0.54551	-0.377355281	0.02854
BRF2	0.066260513	0.682278	0.132835508	0.40741	0.348287862	0.02859
WTAPP1	0.112522096	0.807617	-0.28597809	0.53625	-1.029757444	0.028596
PAK7	-0.308885979	0.639313	-0.396821001	0.54533	1.363567029	0.028678
LMF1	-0.2764212	0.183027	-0.281682588	0.17205	-0.454425136	0.028737
AC112229.1	-0.434811771	0.479279	-0.194770558	0.74502	-1.434758683	0.028764
FRRS1	-0.516101327	0.068408	-0.094399072	0.73811	-0.617738325	0.028808
YWHAE	0.113501152	0.234201	0.087059764	0.36084	0.207986615	0.028827
FGFRL1	-0.247530499	0.138427	-0.282073218	0.09093	-0.364561115	0.028889
QSOX1	-0.202943716	0.237708	-0.155273625	0.36625	-0.37552881	0.028898
MED21	-0.030508199	0.756185	0.083652756	0.38581	0.209718448	0.028951
SNX18P3	-0.574062194	0.170516	-0.327840306	0.4187	-0.924259525	0.028948
DRD1	-0.601450498	0.196578	-0.620566183	0.1756	-1.041431781	0.029126
C9orf131	0.637126829	0.126833	0.712811791	0.08638	0.907143431	0.029176
PPP1R3G	0.215884588	0.355629	-0.169958576	0.4705	-0.520305767	0.029184
NUDCD1	-0.105661954	0.311896	-0.105287923	0.30938	0.22346988	0.0292
CCNT2	-0.068362449	0.425843	-0.010363655	0.90336	0.185369811	0.029212
LEO1	0.000344283	0.996925	-0.07589468	0.39308	0.191331198	0.02928
LIPT2	0.298516384	0.184163	0.300699109	0.1752	0.476334078	0.029285
GNA13	0.16234617	0.076369	0.007828709	0.93181	0.198697711	0.02947
STARD10	-0.190764063	0.195561	-0.262182155	0.07516	-0.320805326	0.029501
ATP5G1	-0.25233259	0.089161	-0.002292346	0.98759	-0.321872731	0.029598
DPP7	0.05600958	0.835286	-0.174135068	0.518	-0.587314021	0.029596
FAM69B	0.018909494	0.905872	-0.220292163	0.1682	-0.349111567	0.029677
RP4-545K15.	0.474796158	0.208141	-0.023710364	0.95008	0.815581187	0.029707
DOLK	0.205907744	0.137593	0.163144431	0.23654	0.297314306	0.029723
HSD17B2	-0.026115998	0.942342	-0.375013505	0.29894	-0.786141486	0.02973
PTGER3	0.169714461	0.545886	-0.448584626	0.11111	0.60937346	0.029776
H6PD	0.05493393	0.601466	-0.103016706	0.32755	-0.228519135	0.029998
KLK6	-0.137454498	0.814542	-0.31939842	0.58492	-1.291074672	0.030093
IDI1	0.067815081	0.628431	0.018105263	0.89715	0.303395297	0.03012
P11-1415C14	-2.222676313	0.483918	-2.398797303	0.44993	-7.195645793	0.030148
VN1R83P	-0.499775855	0.198855	-0.347942598	0.35524	-0.854405695	0.030251
EPHB6	-0.624275464	0.438447	-1.504894509	0.06631	-1.811681681	0.030332
CPM	0.498019129	0.178407	0.525407226	0.15539	0.799461259	0.030381
UBE2I	0.075389304	0.54542	-0.000398023	0.99745	0.269155008	0.030382
UTP3	0.03220591	0.814324	0.158529865	0.24335	0.292723724	0.030439
ZNF750	0.729299758	0.315294	0.181213228	0.80685	-1.744782489	0.030559
PLLP	-0.254718144	0.136192	-0.27204155	0.10985	-0.368425196	0.030586
PLEKHH3	0.033023532	0.886692	-0.121960035	0.59946	-0.503297646	0.030616
TBCB	0.190387701	0.079906	0.147174481	0.17453	0.233403702	0.03062

CELSR2	-0.039670977	0.887241	-0.464867629	0.09736	-0.607801102	0.030635
TMEM30B	-0.17193484	0.289673	-0.108014893	0.50443	-0.349977524	0.03092
SOX8	-0.214531735	0.455057	0.215099736	0.44655	-0.623265707	0.031002
EEF1A1P11	-0.198981739	0.292565	-0.238567744	0.20512	-0.407360812	0.031037
ZFX	-0.02295539	0.756484	0.044370633	0.54647	0.158138548	0.031078
C16orf13	-0.02142119	0.890098	-0.17930793	0.24712	-0.335257025	0.031117
USP39	0.238991249	0.133767	0.243446669	0.12587	0.341984673	0.031122
DNAJC8	0.162344599	0.077003	0.012514933	0.89142	0.196759472	0.031227
CACNA1A	0.274760075	0.638844	0.617077811	0.28227	1.219348779	0.031346
CDKN1B	-0.165134354	0.397312	-0.091278985	0.63878	-0.420147005	0.031294
INPP1	0.149077753	0.452104	0.011742431	0.95273	0.42455476	0.031313
ZNF395	-0.139259332	0.385477	-0.300457221	0.06118	-0.345538043	0.031301
ZSCAN16	-0.167286154	0.300766	0.098729726	0.5255	0.326921005	0.031724
PPAPDC1B	-0.207920487	0.098935	-0.131240893	0.29153	-0.268019268	0.031766
SEC14L4	-0.621053859	0.079708	-0.538013388	0.11815	-0.75034516	0.031803
SIDT1	0.100961833	0.759348	-0.149925466	0.64718	-0.726323348	0.031786
ZBTB3	-0.056887119	0.761668	-0.159670696	0.39546	-0.403769878	0.031822
WTAP	0.101538392	0.26835	0.135447659	0.13749	0.195279416	0.031909
TBX18	0.787052626	0.154338	0.052938277	0.92383	1.182830613	0.031964
MAATS1	0.523168337	0.292581	0.289774792	0.55879	1.05179147	0.032071
VSTM2L	0.0996663	0.815207	-0.115949689	0.78726	-0.968863712	0.032106
ABHD2	-0.202643796	0.311774	-0.075689485	0.70543	-0.429019394	0.03223
CTC-503J8.6	1.658042447	0.053451	0.9357751	0.27844	-2.057873373	0.032444
FBXO18	0.047420726	0.590627	0.117025031	0.17986	0.1862486	0.032458
OR7E59P	0.313462116	0.598609	0.384130163	0.51382	1.219897163	0.032501
ARL4C	0.04374257	0.888535	-0.117068446	0.7073	-0.671954702	0.032524
C22orf39	0.10199043	0.364508	0.134933369	0.22943	0.237079015	0.032552
HPS6	-0.233156653	0.064707	-0.01336696	0.91368	-0.266130981	0.032749
TBC1D1	0.247061034	0.112624	0.133423528	0.39086	0.331166731	0.032753
C14orf39	0.116998752	0.837134	0.482571969	0.39367	1.206606829	0.03278
MIR3916	-0.082234231	0.388242	-0.011475545	0.90315	0.199265226	0.032901
PSMA4	-0.123336262	0.151315	0.031650669	0.71102	0.181628205	0.032948
DSTYK	0.164559387	0.181806	0.171123378	0.16238	0.259923783	0.033031
FOXP2	0.371424838	0.222847	-0.363333769	0.23399	0.648757111	0.033057
LRRK2	1.254411627	0.066279	1.174959925	0.08531	1.462854532	0.033081
GPR3	0.715302065	0.070222	0.644281292	0.10034	0.820561003	0.03325
C3orf14	-0.395080082	0.079333	0.05803777	0.78948	-0.478870393	0.033281
MYH10	0.221795634	0.163049	0.04864977	0.75963	0.33831541	0.033286
DHX16	-0.037628497	0.774646	0.08796608	0.50088	0.277693196	0.033354
PRPF4B	-0.061928131	0.499862	0.006465194	0.94372	0.194243239	0.033363
ZNF155	0.099879428	0.634097	0.196785262	0.3451	0.43586569	0.033466
B3GALNT1	-0.110502663	0.606728	-0.240921541	0.26045	0.450228172	0.033513
GPR126	0.305722012	0.173022	0.059928502	0.78938	0.476296028	0.033681
MGAT4A	0.360741862	0.211714	-0.021550166	0.94054	-0.614989562	0.033847
TMEM147	-0.115772258	0.433181	-0.145550663	0.32312	-0.313166638	0.033869
APAF1	-0.041426032	0.786463	0.266319385	0.0804	0.32288462	0.033957
HECTD1	-0.027112095	0.743888	0.161271124	0.05122	0.175337504	0.033977

DHX9	0.016665843	0.84787	0.104231764	0.22964	0.183760956	0.034084
CHSY3	0.626134054	0.061028	0.008533211	0.97964	0.705344498	0.034172
FUT11	-0.146645974	0.33041	-0.238542433	0.11308	-0.318702192	0.034298
NLE1	-0.18013372	0.350678	-0.24275435	0.20712	-0.407071757	0.034279
RBM23	0.190166415	0.070159	0.074136844	0.47967	0.221360197	0.034267
RGS6	0.089353154	0.889727	0.657283118	0.30028	-1.432853918	0.034259
ZBTB5	0.217642118	0.053073	0.130277587	0.24519	0.235931375	0.034284
ZNF511	-0.169257103	0.199798	-0.183060918	0.16294	-0.277525306	0.03452
LY9	0.081841959	0.779286	0.054593967	0.85181	-0.633499041	0.034574
C18orf21	0.210039524	0.150125	0.139609521	0.33528	0.30320302	0.034622
HCFC1R1	0.241227083	0.120654	-0.09931213	0.52626	-0.332894156	0.034645
SCHIP1	0.578321537	0.122432	-0.36915323	0.32895	0.790027572	0.034702
RICTOR	-0.102629352	0.392701	-0.147683248	0.21705	-0.252453039	0.034751
ABAT	-0.065732906	0.674499	-0.024627928	0.87444	-0.330336231	0.034929
HDGFP1	0.455537515	0.261212	0.571427893	0.15114	0.825661842	0.034928
ITCH	-0.108877924	0.48159	0.133996214	0.385	0.324191944	0.035001
TMEM170B	0.002117127	0.988416	0.225642313	0.11696	0.30221151	0.035117
PAF1	0.063491759	0.59492	0.024453983	0.83731	0.250076568	0.035154
ZNF821	0.145438879	0.527808	-0.178006951	0.43843	0.476796534	0.035352
ARMCX6	-0.25421047	0.082066	-0.02151866	0.88175	-0.306422775	0.035423
UBXN1	0.13767988	0.344912	0.121033711	0.40501	0.304873498	0.035433
DIMT1	-0.017953403	0.878683	0.144280225	0.21605	0.244024757	0.035454
ENOX1	0.323913361	0.296955	0.106076113	0.73374	0.648941241	0.035517
WASH7P	0.717755397	0.087641	0.395301003	0.34733	0.881285694	0.03554
AP003068.18	0.231317774	0.597708	0.664421243	0.11858	0.896976855	0.035616
FAM160B1	-0.02339202	0.890307	0.054109368	0.74891	0.354983573	0.035644
MZT2A	0.085086508	0.702606	-0.044776681	0.84059	-0.469190786	0.03571
TMTC4	-0.082504977	0.511216	-0.068933894	0.57988	-0.26217084	0.035834
CES4A	-0.062142537	0.802012	0.219520104	0.35973	-0.532266875	0.035999
P11-34P13.1	-0.340947766	0.454556	-0.186125435	0.68089	-0.966160806	0.035986
SMG5	0.101477671	0.22611	0.084854978	0.31001	0.174833716	0.036067
DUSP8	0.966915616	0.068846	0.418292623	0.44224	1.111331761	0.036178
FGD3	0.084530215	0.906344	-0.916355887	0.19498	-1.551162856	0.036255
PLEKHB1	-0.634614236	0.082882	-0.534553681	0.14283	-0.764737399	0.036233
C5orf22	-0.046105823	0.673759	0.087942493	0.41627	0.225479412	0.036376
NUP93	0.199097531	0.186366	0.143861868	0.33779	0.313916036	0.036405
ATP2B1	-0.083624166	0.536749	-0.161133726	0.23362	0.282653189	0.036462
ANKRD1	0.368152301	0.451696	0.071363235	0.88425	1.009788559	0.036633
SLC35F2	-0.16666089	0.334636	-0.167592469	0.33044	-0.360060796	0.036809
AGAP1	0.063684133	0.684136	-0.0556793	0.72175	-0.329041917	0.036838
CSNK1G1	0.154329215	0.122607	0.194698225	0.0505	0.207355982	0.03687
HOXD9	-0.24009936	0.737668	-1.430941601	0.05042	-1.528467077	0.036876
FAM86A	-0.12530669	0.44778	-0.133212441	0.41535	-0.341420288	0.036927
MLLT3	-0.399346846	0.161152	0.003913496	0.98902	-0.593898457	0.036922
HOXA10	0.410269113	0.318957	-0.280736384	0.49721	-0.868993576	0.036957
SGSM1	-1.11029366	0.100615	-0.892547775	0.18511	-1.404493952	0.03717
KCNK3	0.469663635	0.200224	-0.715867961	0.06403	-0.807093577	0.037203

TTC12	-0.443446763	0.05013	-0.355756459	0.11104	-0.465608956	0.037195
RP11-396K3.	-0.098126058	0.510618	0.025840305	0.86218	0.309549002	0.037264
TOR3A	-0.238398886	0.09527	-0.258480329	0.06544	-0.292802296	0.03731
SEPHS1	-0.001835633	0.985824	0.077179316	0.45359	0.213779701	0.03738
OSTM1	0.223912518	0.213986	0.115813569	0.51894	0.371422238	0.037509
C11orf57	0.044423958	0.756461	0.112667032	0.42953	0.29602333	0.037563
PLA2G2A	-1.173988993	0.215337	-0.211420936	0.82256	-1.978328889	0.037628
PRKAG2	0.266200696	0.262022	0.054140166	0.82021	0.490212607	0.037696
METRN	-0.021385071	0.947406	-0.039186724	0.90369	-0.680751275	0.037721
HAND2	0.36475135	0.197042	-0.114765998	0.68566	0.584197041	0.037765
ITIH5	0.276849015	0.468953	0.32567898	0.39136	-0.804346756	0.037778
CSNK2A2	0.054552345	0.549981	0.04619862	0.61183	0.188483294	0.03781
PAGR1	-0.188230913	0.072364	0.002457614	0.98095	-0.215182518	0.037918
MYO9A	-0.322181509	0.112555	-0.155258549	0.44393	-0.420854072	0.037989
ZNF160	0.123616561	0.268555	-0.065297305	0.559	0.230802258	0.037984
PAIP2	0.062039338	0.57869	0.211448049	0.05666	0.22997643	0.038082
DLAT	-0.090809514	0.386152	-0.07027176	0.50081	0.215436787	0.038113
SWSAP1	-0.148208919	0.481864	-0.333275699	0.11415	-0.438862867	0.03816
ABHD13	-0.1448139	0.109588	0.019787488	0.82375	0.18232521	0.038182
POMGNT1	-0.127516705	0.209981	-0.031048779	0.75821	-0.209658151	0.038233
P11-872D17	-1.295896532	0.348418	0.619916725	0.64406	-3.090316745	0.038235
HSPA1B	-0.278907533	0.080881	-0.2096123	0.18883	-0.330587864	0.038308
KRT13	0.043933634	0.96971	-1.267103607	0.27544	-2.446300754	0.038296
GNG12	-0.165154473	0.075211	-0.049199072	0.59518	-0.191898323	0.038359
PRSS3	-0.901590799	0.152222	0.307137851	0.59519	-1.280083008	0.038551
STAM	-0.015843771	0.915424	0.068314634	0.64595	0.306899321	0.038519
SNX13	0.059065964	0.549783	0.079559904	0.41847	0.203105941	0.038754
ANKRD40	0.120053854	0.188266	-0.070195723	0.44069	0.186861798	0.038797
FRMPD3	-0.115863786	0.79989	-0.707228358	0.13823	-0.98601412	0.038859
FTH1	0.373868748	0.079223	0.362442728	0.08879	0.439915577	0.038851
FAM49B	-0.094444231	0.416976	-0.055447694	0.63242	0.237958841	0.039
C2orf81	-0.73831695	0.143692	-0.569805861	0.25752	-1.052129572	0.039018
MOK	-0.160860082	0.378434	-0.274461555	0.1292	-0.372419212	0.039094
KLHL35	-0.325139403	0.487138	-0.127856518	0.77989	-0.974685234	0.039215
ZKSCAN5	0.104891166	0.319166	0.118313698	0.25734	0.214103676	0.039377
SHISA2	0.299893175	0.264267	0.250125831	0.3515	0.552233693	0.039424
TMEM143	-0.220128567	0.371962	-0.410234436	0.10167	-0.511965446	0.039421
GORAB	0.089327479	0.540713	0.26341475	0.06723	0.295128939	0.0396
KDM6A	-0.251807601	0.073349	-0.093676198	0.50431	0.287758382	0.039692
FLVCR1	0.193990322	0.291991	0.265426642	0.14865	0.377559637	0.039871
HPGD	-0.469904474	0.197906	-0.31013237	0.39532	-0.75010445	0.039873
UBQLN1	0.140984138	0.228985	0.14903516	0.20259	0.240213982	0.039906
HIST2H2AC	0.099449979	0.590421	0.081334411	0.65957	0.378851658	0.039934
NECAB1	0.089830729	0.706734	-0.227746714	0.34074	0.481879828	0.039961
ZSWIM5	-0.139299032	0.494438	-0.015497762	0.93872	-0.420347362	0.040035
FAM174A	-0.271859667	0.10687	-0.02416611	0.88374	-0.344633932	0.040112
SYCP2L	0.389357627	0.202707	0.276873356	0.36619	0.620951977	0.040104

MYO3A	-0.48987904	0.157193	-0.132863788	0.693	-0.695378126	0.040193
FBXL2	0.13535166	0.331797	0.140776286	0.30864	0.282044273	0.040304
LRRN3	0.368491748	0.237361	0.582369547	0.06077	0.636254052	0.040303
PAXIP1	-0.032107652	0.785294	0.082493319	0.48154	0.238817876	0.040344
AC016739.2	-0.07089071	0.710692	0.012548236	0.9476	-0.391839616	0.040404
ZNF346	-0.205155483	0.324945	0.140433134	0.49852	0.423940951	0.040407
ZNF436	-0.297858942	0.080949	-0.152430043	0.37048	-0.349059126	0.040508
SFXN4	-0.287264096	0.152663	-0.362308447	0.06783	-0.408067022	0.040566
FASTKD5	0.230076694	0.096133	0.173606758	0.20752	0.280762477	0.040608
PRKRA	-0.094638285	0.341449	-0.007214162	0.94157	0.200001866	0.040684
ZNF140	0.033179615	0.800418	0.045006332	0.72899	0.264424844	0.040688
CERS5	0.240460202	0.097447	0.180857576	0.21254	0.295527258	0.040826
FAM110C	-0.098414394	0.687045	-0.069842929	0.77442	-0.501784421	0.040819
EXOSC10	0.10356684	0.139985	-0.051224518	0.46386	0.141852771	0.040923
ISG20L2	0.259547073	0.135331	0.156561702	0.36714	0.35414695	0.040897
BRAT1	-0.05739656	0.740601	-0.039461917	0.81931	-0.354548067	0.0411
RWDD1	-0.132390542	0.206125	0.031586134	0.76137	0.210967133	0.041248
PPP2R3B	0.400448451	0.441114	-0.3709206	0.48881	-1.088573303	0.041395
AMDHD2	-0.261583302	0.36489	-0.517559564	0.07404	-0.592173613	0.041454
MIF	0.211412649	0.237874	-0.052113165	0.77119	-0.36591802	0.041495
ADNP2	-0.09377904	0.465632	0.052749934	0.68013	0.260244683	0.041533
ENGASE	-0.094143311	0.540458	-0.267280301	0.08125	-0.313132664	0.041523
ACOX2	-0.2017858	0.58304	-0.68409106	0.06286	-0.753812976	0.041605
EIF4A1P2	0.116380748	0.925162	-0.303644093	0.80667	-2.622404023	0.041577
ASMTL	-0.192796258	0.355182	-0.021319232	0.91743	-0.423321311	0.041806
FOXD4L5	-0.143461698	0.756909	-0.121166288	0.79135	-0.98708937	0.041801
MROH6	0.18056506	0.613755	-0.111114854	0.76291	-0.746706209	0.041837
G0S2	0.098937627	0.7944	0.079717815	0.83291	-0.791220583	0.041905
CNOT1	0.269008237	0.085398	0.143338884	0.35927	0.317859648	0.04201
TSPAN1	0.019264238	0.924302	0.217687733	0.28247	-0.41238297	0.042057
SNAP91	1.035891715	0.12333	-0.04530598	0.95005	1.378861993	0.042166
RPS5	-0.191700584	0.119278	-0.2084963	0.09006	-0.249757822	0.042299
C21orf2	-0.023801483	0.850496	-0.036056235	0.77468	-0.257133911	0.042363
RCOR3	-0.237407599	0.089958	-0.238189384	0.08702	-0.281992922	0.042418
QTRT1	-0.021797219	0.898974	-0.179980393	0.29348	-0.348483181	0.042484
P11-206L10	-0.271757714	0.621423	-0.405183162	0.4612	-1.128430385	0.042533
ZNF586	-0.198682342	0.079311	-0.160803528	0.14835	-0.225127175	0.042582
SPIB	0.324079396	0.448536	0.263331131	0.53691	0.859049476	0.042709
HIPK3	0.140072204	0.087196	0.100531504	0.21883	0.165412079	0.042853
KIAA1551	-0.022489124	0.936794	0.046535061	0.86965	0.574075587	0.042794
LEPROTL1	0.124554173	0.480976	0.19398789	0.27112	0.35653957	0.04284
RAB18	0.00312499	0.975019	0.08202291	0.41039	0.200747799	0.042843
ZNF669	0.013366901	0.917868	0.088480157	0.48592	0.253196187	0.042925
SLC35B3	-0.180930397	0.11367	0.21018788	0.05856	-0.228971688	0.043047
FCER1G	0.221265756	0.751464	-0.733930277	0.29659	1.405582511	0.043091
DHX38	0.040937935	0.790192	-0.138266405	0.36836	0.310416219	0.043161
TMEM201	0.087370046	0.683082	-0.248961333	0.2475	-0.436152844	0.043131

TSTD2	0.252910056	0.081858	0.078070969	0.59007	0.292091497	0.043139
SLC12A9	-0.087635043	0.578652	-0.047892756	0.75991	-0.31835114	0.043208
USP21	0.116690412	0.471626	-0.106616088	0.5099	-0.328475286	0.04334
ATIC	-0.360610699	0.165663	-0.322852346	0.21342	-0.524792532	0.043593
C12orf60	-0.45831541	0.085854	-0.36891368	0.16619	-0.533773766	0.043579
CNOT7	0.016825336	0.888056	0.072110014	0.54539	0.240334751	0.043562
MAN2A1	-0.264681615	0.053557	-0.060422878	0.6589	-0.276399122	0.043582
MXI1	-0.322897381	0.296537	-0.411902024	0.18277	-0.624456472	0.043548
WDR36	-0.050121235	0.715594	-0.064453831	0.63791	0.274023794	0.043597
C7orf50	-0.287680624	0.11182	-0.193141207	0.28326	-0.363013418	0.043642
CCDC85B	0.262469202	0.498839	-0.525669936	0.19258	-0.829324937	0.043744
IGSF8	-1.143409792	0.092088	-0.134892799	0.8405	-1.364884034	0.043853
KBTBD2	0.078822577	0.493752	0.144067493	0.20843	0.230596305	0.043896
HHAT	-0.47944864	0.075074	-0.103657253	0.69524	-0.541677116	0.043937
PSD4	-0.187652506	0.242384	-0.050342436	0.75271	-0.323201223	0.044006
CNGA1	-0.136805243	0.740042	0.198287892	0.6267	-0.834652711	0.044179
PSMB4	0.083821449	0.315482	0.088002972	0.28998	0.167057991	0.044194
FFAR2	0.448586356	0.261734	0.414964412	0.29524	0.795432921	0.044237
COX18	-0.181796784	0.173597	-0.034625006	0.79331	-0.26638009	0.044422
SLMO2	0.006445388	0.951873	0.13310424	0.21051	0.213397486	0.044428
HABP4	-0.026400852	0.849159	0.012710631	0.92617	0.271589205	0.044479
TMEM91	-0.441737798	0.174174	0.070939769	0.82311	-0.649861693	0.044464
CARD10	-0.319259796	0.058759	-0.152393698	0.36582	-0.339843907	0.044518
GTF2A2	-0.131142522	0.24553	0.139024976	0.21415	0.223914599	0.044565
MRPS12	-0.151097779	0.313108	-0.072824642	0.62253	-0.298968391	0.044733
MUC12	-0.519234586	0.212442	-0.274142476	0.49766	-0.829287979	0.044852
C22orf29	-0.153839345	0.229174	-0.056907323	0.65347	-0.254591093	0.044911
PABPC4L	0.061933327	0.824057	0.107152221	0.69853	0.548240931	0.044946
RRAGA	0.103908051	0.304005	0.191510848	0.05563	0.200197936	0.044961
MAP4K3	-0.006144407	0.949677	-0.146657985	0.13121	0.193794823	0.045151
VWA1	0.012720478	0.939746	-0.122592531	0.46602	-0.337340742	0.045183
FAM86B3P	0.036513145	0.882475	-0.311391713	0.2087	-0.494970472	0.045263
IL6R	0.462728818	0.246986	-0.408679869	0.30884	0.792331775	0.045342
MRPL41	0.21860009	0.193563	0.022306266	0.89456	-0.343137016	0.045324
SREK1IP1	0.283073263	0.084901	0.066310881	0.68531	0.32683891	0.045345
PDE4C	-0.170098598	0.415337	-0.254440281	0.22398	-0.418384851	0.045596
EYA2	1.002442474	0.229674	0.32127937	0.70095	-1.727898962	0.045769
NDRG2	0.032724535	0.870572	0.020860166	0.91715	-0.402284459	0.045779
MT1X	-0.013213911	0.984855	-0.449464496	0.51906	-1.403950467	0.045808
EFCAB6	0.075367606	0.748525	0.102737625	0.65616	-0.469947789	0.045861
TG	1.169877565	0.066936	1.198928107	0.05766	1.268051455	0.045951
ATP8B5P	-0.734346911	0.150723	0.237800533	0.62039	-1.055099566	0.046135
PYCR1	-0.014053971	0.915923	0.015743841	0.9056	-0.265317676	0.046142
LAMB1	-0.157691121	0.325538	0.008186987	0.95928	-0.319744287	0.046246
RCN1P2	0.275855399	0.065588	0.220904055	0.13826	0.295483777	0.04628
PYCARD	-0.115800846	0.663167	-0.05765397	0.825	-0.539153887	0.046327
DENND4A	0.042105786	0.778051	0.001357906	0.99273	0.295964228	0.046667

OPRK1	-0.127123706	0.678002	0.071905492	0.81406	-0.609403499	0.046883
TPM3	0.142916653	0.247417	0.123339602	0.31797	0.245383779	0.046873
P4HTM	-0.267543224	0.107315	-0.275811262	0.09649	-0.329917591	0.046956
SLC1A2	0.381967861	0.462713	0.858892134	0.09003	1.018982594	0.047013
PPP1CB	0.035153729	0.678509	0.035962107	0.67108	0.168014448	0.04706
GLIS3	0.383996242	0.107577	0.145427551	0.54214	0.473165267	0.047082
CLK2	0.159080832	0.091884	-0.01558306	0.86883	0.185830749	0.047143
PVRL3	-0.140103083	0.270008	0.069972986	0.5775	-0.250240139	0.04717
DRG1	0.06086674	0.534511	0.041792135	0.66641	0.191153829	0.047307
ZDHHC16	-0.003947633	0.964983	0.066726152	0.45277	0.175307308	0.047356
ZNF17	0.109011491	0.430817	0.249238119	0.0647	0.266023616	0.047373
ZNF398	0.097458399	0.416316	-0.124632832	0.29666	0.235106946	0.047372
MZT2B	0.089261583	0.724351	-0.060505886	0.81087	-0.503510151	0.04748
RNASE4	-0.342277037	0.082612	-0.297613022	0.13032	-0.389678518	0.04758
TPP2	-0.292217037	0.081492	0.036001594	0.82965	0.330806652	0.047798
FAM115C	-0.032475287	0.882179	-0.066367283	0.76122	-0.434967772	0.047837
COPS7A	-0.196093588	0.09699	-0.034182992	0.76882	-0.231281452	0.04789
MPST	-0.031883371	0.846027	-0.080124172	0.62495	-0.324574789	0.04801
SMPD3	-0.243147002	0.494546	-0.325801236	0.35899	-0.703495005	0.048053
MON1A	-0.112870924	0.627662	-0.203306471	0.37741	-0.457946468	0.048088
GSTO2	-0.422987939	0.145718	-0.481333432	0.09588	-0.57390277	0.04813
AREG	0.342821721	0.532075	0.429770423	0.4327	1.078143198	0.048317
AQP1	-0.032962497	0.95197	-0.573554254	0.2976	-1.104184939	0.048393
SEC23A	-0.060884884	0.584223	-0.029403745	0.79125	0.218920724	0.048443
PAPOLA	0.065051334	0.42291	0.025317307	0.7547	0.159695571	0.048528
CEP89	0.16452643	0.282284	0.015025635	0.92157	0.299883042	0.048562
ZNF473	0.049766747	0.686271	0.050667755	0.6776	0.237972921	0.048658
WDR89	-0.661011724	0.064407	0.268160887	0.44377	-0.703871184	0.048701
WDR18	-0.012946026	0.952235	-0.045911008	0.8313	-0.427702541	0.048755
RBFOX2	0.034969802	0.821773	0.089301464	0.56482	0.305015858	0.048916
CLINT1	0.184036582	0.130019	0.209338564	0.08481	0.239064705	0.049024
ICK	-0.281671693	0.05028	-0.269454538	0.06064	-0.282499124	0.049113
NUP98	0.167463311	0.119293	0.162743297	0.12964	0.211132844	0.049041
SMIM22	-0.240508181	0.197739	-0.169052276	0.36187	-0.366456346	0.049096
SPATS2	0.086699389	0.616736	0.017391775	0.91979	0.33885322	0.049092
VEZF1	0.06192943	0.47472	-0.073744863	0.39361	0.169143864	0.049157
CCT3	-0.01113909	0.920057	0.121647361	0.27227	0.217775923	0.049244
YY1AP1	0.060914982	0.60744	0.015827376	0.89388	-0.232678311	0.0492
BAK1	0.201021964	0.135128	0.090628324	0.49896	0.260407569	0.049286
RABL6	0.012956388	0.887294	-0.071455068	0.43368	-0.17966766	0.049395
LEPREL2	-0.144112294	0.660934	-0.016863641	0.95902	-0.647145703	0.049463
UBXN4	-0.044650208	0.488068	0.066352069	0.29968	0.125496354	0.049465
DEAF1	0.011942611	0.919318	-0.13524415	0.25105	-0.231355319	0.049653
UAP1L1	-0.037271668	0.881608	-0.036757138	0.88235	-0.496901507	0.049736
YTHDC1	-0.031795583	0.729312	-0.016805295	0.85386	0.178119658	0.049695
VKORC1	-0.15673878	0.298935	-0.104574971	0.4865	-0.295719282	0.049823
HOXA1	0.55234865	0.162576	-0.199511654	0.62295	0.764626194	0.049844

LRP2BP	0.376085273	0.067016	-0.079876822	0.69959	0.399610662	0.049948
OGFOD3	-0.114512506	0.436575	-0.201205471	0.1688	-0.286593801	0.049937
REST	0.347554776	0.177746	0.312659796	0.22507	0.50458199	0.049955
AC007875.2	-0.257977093	0.705753	-0.966012982	0.17851	-3.218842594	0.001832
AC007881.4	-1.001413181	0.544005	-1.716339938	0.30225	-6.07641449	0.001502
AC007969.5	-0.385431295	0.688124	-0.032001774	0.97247	-3.702298575	0.005251
AC073551.1	-0.65702208	0.21714	-0.888273895	0.09775	-1.134612504	0.038515
AC098614.1	-0.945716445	0.266917	-1.290533568	0.13895	-2.192799545	0.027692
AC104451.2	-1.644624845	0.0623	-1.557306906	0.06527	-1.895323413	0.032175
AC124914.3	-0.108710695	0.855644	-0.311823346	0.6027	-1.600808268	0.019576
AC239811.1	-0.907094259	0.160956	0.319300768	0.59473	-1.369253533	0.037134
ACRBP	-0.908083148	0.200801	-0.555379521	0.43953	-1.518783849	0.04077
AKR1B10P1	-1.652294868	0.138413	-1.784359419	0.10823	-4.191764655	0.002592
AQP5	-0.526345291	0.596754	-1.949369447	0.06433	-3.211933455	0.007106
ASB9P1	-0.133037747	0.903016	-0.911927271	0.43607	-2.702297034	0.049513
ASCL1	1.577528137	0.254424	0.653661133	0.64626	3.346215388	0.012391
ASIC4	0.586103805	0.286389	0.332381736	0.54507	1.153825481	0.029825
BMS1P3	-0.451744709	0.772458	-0.540172971	0.72816	-3.429632141	0.049778
BMS1P9	-0.062718055	0.940139	-1.487169257	0.08775	-1.953290982	0.028304
C1orf110	-0.050743162	0.96075	-1.494455212	0.16561	-3.519676204	0.006267
C1orf53	-0.136850362	0.79663	-0.05315947	0.91705	-1.193448755	0.032607
C3orf55	0.32720945	0.694379	0.832005097	0.29632	1.938284156	0.011276
C5orf38	-0.824318891	0.402389	-0.113039349	0.90372	-2.570438643	0.018465
8orf44-SGK	-0.88637593	0.691377	-1.18516772	0.59413	-5.965619264	0.013991
CCDC135	-0.477747148	0.445906	0.101602435	0.8684	-1.257094674	0.0483
CCER2	-1.141201459	0.209535	-1.493534513	0.12215	-2.343057973	0.038898
CCKBR	-1.033538294	0.259831	-1.405674662	0.10389	-1.885776589	0.034681
CCL21	2.882502244	0.058836	1.30351372	0.43429	3.265314375	0.029443
CCNJP2	-0.383086786	0.558273	-0.57165659	0.38288	-1.506872488	0.034302
CCNO	0.265407219	0.767877	0.200159378	0.82282	-2.193881879	0.029971
CD86	-1.906380854	0.240634	-0.853600358	0.58011	-3.567799049	0.039147
CDC42P2	-1.525454692	0.156681	-1.206281763	0.24067	-4.017195488	0.003075
CDHR4	-0.923399893	0.115126	-1.164315686	0.05084	-1.255587454	0.033995
CERS3	0.604906251	0.630018	-0.167866546	0.89794	-3.316784556	0.031649
CHADL	0.34173819	0.688197	-0.022920168	0.97696	-2.432990374	0.016584
CPA5	0.99808104	0.577154	2.177818761	0.17073	3.339804346	0.033786
CPNE4	-1.380127067	0.168653	-0.403861111	0.67518	-2.341134991	0.030325
CSNK1A1P1	-0.310291822	0.633011	-0.472606109	0.43276	-1.676436017	0.011511
CTRL	1.486866497	0.112775	-0.514309316	0.63399	-3.63782748	0.007065
CYP3A4	-0.952760069	0.181554	-1.209953099	0.07968	-1.533887316	0.031931
DDX50P2	-2.429336687	0.070543	-1.277082308	0.25467	-4.114808882	0.002716
DND1	4.09684842	0.133865	1.932487683	0.49389	5.539346952	0.041022
DNER	1.443540301	0.080105	1.189958453	0.15488	2.153681293	0.007763
ELAVL3	1.441271622	0.202601	1.995030702	0.07466	2.663124623	0.015923
ELAVL4	0.554861177	0.777856	-0.715951131	0.72828	3.701348944	0.045593
ENHO	-0.797078535	0.472049	-0.458719402	0.66257	-3.785163161	0.005395
EVX1	-0.086068551	0.922772	-1.492107725	0.12031	-4.114449022	0.00157

FAM155B	-0.324842613	0.576665	-0.220813674	0.69954	-1.234130964	0.0442
FAM204BP	-0.641046581	0.474744	-1.645370115	0.08558	-2.289971242	0.024848
FAM90A2P	-0.935758222	0.366924	-0.706962326	0.48583	-2.513168887	0.031442
FBXL16	-0.974711546	0.255203	-0.844067461	0.3159	-2.323676245	0.018215
FDPSP3	2.067985181	0.121205	2.205086515	0.09099	2.505537465	0.049326
FREM3	-1.443609471	0.268427	-0.469972719	0.71395	-3.314744386	0.01519
GABRB3	0.860330787	0.430402	1.483620371	0.16936	2.264559302	0.032288
GAPDHP52	-0.628387142	0.607516	0.190563782	0.8593	-2.8787296	0.034012
GAPDHP65	-1.062706934	0.299501	-0.470349025	0.62552	-3.105323629	0.017579
GBP1P1	0.470496822	0.469787	1.029452708	0.09951	1.396914296	0.020828
GRIN1	-0.233976858	0.852282	-0.83581755	0.52581	-3.023307866	0.032591
GUCA2B	3.085020036	0.05934	0.827915969	0.63738	3.490190411	0.03141
HCG22	2.065134414	0.174586	-0.117831431	0.94511	3.691525975	0.009163
HMGB2P1	2.145833218	0.120521	-0.839178054	0.59927	2.805963805	0.032704
HPGDS	-2.057124436	0.120581	-0.744759614	0.49336	-3.234665777	0.019391
HSPB9	-0.326220897	0.61042	0.149754981	0.80086	-1.669607329	0.030401
IFNW1	-0.400612967	0.595514	-1.20678677	0.1354	-2.118017482	0.02067
KB-1205A7.1	1.330057032	0.339693	1.452211313	0.2874	2.716467902	0.034627
KIF5A	1.812607523	0.107834	1.727512975	0.12054	3.527825339	0.001139
KRT18P21	0.052796792	0.976016	0.603514986	0.7311	3.076322527	0.044318
LGALS7	1.585045965	0.141111	-1.095505709	0.38418	-2.865681655	0.047937
LIPM	0.639571546	0.241607	0.596799049	0.26959	1.07920612	0.037099
LY6D	0.199619458	0.870989	-0.015415461	0.99018	-3.884505749	0.011434
LY6G6E	-1.166619824	0.391453	-0.728295047	0.57295	-3.089707897	0.044449
LYVE1	-0.61144631	0.490008	-0.229792566	0.79263	-1.847598197	0.049857
MIA-RAB4B	-1.062023591	0.494036	-0.139378264	0.92481	-4.016974584	0.020803
MOGAT1	0.862287294	0.25106	0.432146108	0.57047	1.851615653	0.010048
MRPL53P1	-0.119823166	0.891714	-0.258122217	0.76872	-2.36542013	0.039034
NOTUM	-0.020272392	0.975115	-0.829542036	0.21338	-1.739833952	0.015681
NPM1P34	-0.839237263	0.56524	-1.935859225	0.22914	-3.453956789	0.033045
NPM1P39	0.401898727	0.749851	2.029477717	0.06392	2.496205837	0.019993
NRN1	-0.379037894	0.789917	-0.44593802	0.7513	-4.038454679	0.017323
OR2A42	0.610546784	0.48622	0.285311496	0.74771	1.788563781	0.028776
OR52N1	1.500221452	0.078909	0.618110567	0.48165	2.483778784	0.002791
OR52N4	0.912432774	0.318337	1.469570035	0.09792	2.132050403	0.014484
OR52N5	1.157349271	0.240485	0.795620388	0.42439	2.513334411	0.007245
OR56B1	2.864622931	0.094835	1.854079076	0.28846	3.666034091	0.030401
OR7A19P	-1.027727906	0.32861	-0.864289178	0.39192	-2.551814519	0.04665
OTOF	-1.02592645	0.106047	-0.307383933	0.6132	-1.710806867	0.010419
PCDH8	-0.889663275	0.415889	-1.850420157	0.11281	-3.260363529	0.018351
PHOX2B	3.723866131	0.153424	1.180632598	0.66906	5.622341985	0.029299
PHYHIP	-0.930947297	0.282406	0.07222577	0.92826	-2.382947853	0.020712
PMCHL1	-0.61561706	0.339965	-0.213764233	0.73364	-1.76016054	0.010278
PSMC1P9	-1.430403828	0.094751	-0.904382497	0.26164	-2.067815818	0.020997
RBM24	2.38502737	0.06173	1.647984272	0.20068	2.815318455	0.02361
RET	1.444995719	0.219114	0.862559102	0.47005	3.460368579	0.002054
RNF224	-0.699354362	0.139837	0.05776164	0.89204	-1.272944961	0.010664

P11-162A23	-1.92414012	0.159802	-0.483255359	0.68278	-3.432787797	0.020828
P11-169K16	1.289870539	0.370271	2.266567601	0.0944	2.621954848	0.049872
P11-182I10.	1.348413851	0.367208	1.602993502	0.26439	2.877062184	0.029589
P11-236F9.	-1.324324846	0.098263	-1.275883925	0.11811	-1.642360561	0.039463
P11-302B13	1.137772607	0.281251	1.511753713	0.14038	1.988915599	0.047643
P11-323I15.	0.397160408	0.750164	-1.590255166	0.29123	-3.090077211	0.041522
P11-325O24	-1.444358941	0.109614	0.089123243	0.91527	-1.785648544	0.049613
P11-355O1.	-0.046903758	0.947697	-0.995855215	0.19439	-1.754844093	0.036328
P11-403P13	-0.958969256	0.086936	-0.107252613	0.82985	-1.339186708	0.019245
P11-430L17	0.883910315	0.188123	0.455823956	0.50801	1.362452538	0.03253
P11-436M15	0.504641972	0.500549	-1.554160755	0.08454	-2.232827381	0.024843
P11-445J14	-1.185983027	0.197835	-0.938836025	0.29122	-1.997057123	0.041323
P11-512H23	-0.293814378	0.54141	-0.759314763	0.12521	-1.214673593	0.018968
P11-536L3.	-0.020983876	0.97844	0.780281033	0.27383	1.394589697	0.042241
P11-561C5.	-0.284198138	0.879483	-0.463098438	0.80453	-4.275279053	0.040893
P11-569G9.	1.159628792	0.202604	1.250711304	0.16128	1.87311747	0.02907
P11-676M6.	-0.57158756	0.70521	0.867945101	0.55185	-4.445619536	0.011491
P11-677M14	-1.150706172	0.128139	-1.033685492	0.15737	-1.678752293	0.032894
P11-718B12	2.667074507	0.059017	1.896529169	0.19477	3.088589455	0.024793
P11-82K18.	2.481535862	0.128403	-0.117831404	0.94893	3.475809422	0.027322
P11-87N24.	-1.402448251	0.233688	-1.106468686	0.32654	-2.989700333	0.031987
P11-894J14	-1.515600627	0.145726	-0.067160633	0.94471	-2.121715621	0.049005
P4-583P15.1	-2.169403349	0.202633	-0.35734775	0.81044	-3.922203629	0.023773
RPEP3	0.863424964	0.476845	0.957410525	0.41944	2.235843249	0.040975
RPL29P25	-0.319284618	0.564829	-0.998177533	0.08606	-1.369209496	0.022895
RPL5P24	-1.083919276	0.076844	-0.954915368	0.10778	-1.27953733	0.035919
RPL5P30	0.75528691	0.19789	1.042438732	0.06506	1.410661656	0.010231
RPS24P13	0.052796806	0.973693	2.176773603	0.11162	2.6118185	0.049608
RPS3AP49	-0.84193013	0.366009	0.41139233	0.59534	1.477468814	0.039028
RTP4	0.003481883	0.997585	0.658326632	0.52969	2.122357343	0.025471
SETP17	2.284554408	0.150457	1.200364307	0.47673	3.006116686	0.049751
SH2D2A	-0.406964755	0.479884	-0.219168048	0.70178	-1.711290875	0.007336
SHD	1.899445277	0.104002	-0.004685753	0.99717	2.460986358	0.031223
SLC16A11	0.985495888	0.088956	0.066313032	0.91294	-1.352005032	0.041644
SLC22A31	2.191512876	0.110888	1.705192194	0.22127	2.658237773	0.047193
SLC35F3	-0.306871871	0.727158	-0.638923187	0.4725	-3.357519418	0.007632
SLC35F4	-1.372651817	0.093383	-0.309786295	0.68653	-2.32054747	0.004556
SMIM1	0.096672929	0.835782	-0.373980831	0.43528	-1.194337568	0.026944
TATDN2P3	-0.763874019	0.483776	-0.561775153	0.59605	-3.553061519	0.010699
TEKT3	0.583462745	0.348843	0.798465555	0.19564	1.332583569	0.025059
TFAP2B	-1.609351003	0.316911	-2.304974308	0.15551	-5.830689834	0.0018
TMEM63C	0.22250749	0.82693	0.293623159	0.77719	-2.884632598	0.015902
TMIE	2.545903642	0.061969	0.594688815	0.7106	3.230347512	0.013714
TSPYL5	-2.602924592	0.050106	-1.292175279	0.26702	-4.980204765	0.000715
TTLL2	-0.980655491	0.13034	-0.787989343	0.20766	-1.472649024	0.026235
TUBBP6	-1.03629098	0.171362	-1.058114226	0.15875	-2.252284808	0.005941
UBE2L4	-0.531828841	0.373535	-0.987811285	0.10508	-1.74349064	0.007224

VIP	1.044781663	0.608321	-0.153142759	0.94342	4.32517759	0.024691
VIT	0.070779789	0.947834	0.631766422	0.55441	-3.160695235	0.013528
VWF	-1.050695793	0.253147	-1.055800971	0.2519	-2.661062285	0.005304
WEE2	-0.025410718	0.977267	-1.172393661	0.20374	-2.113152753	0.029986
WISP2	-0.155696295	0.732472	0.188100717	0.66341	-1.0921566	0.025188
XCR1	0.790544525	0.516638	0.140566663	0.91028	-3.059429973	0.017843
YBX1P7	-0.551987509	0.482979	-1.540237189	0.06701	-2.633034604	0.005983

Table S2. DEGs of STM, T3SS-1^{mut} and T3SS-2^{mut}-infected HIOs at 8h relative to PBS-injected HIOs. Significant DEGs (p< 0.05) in at least one infection condition are listed.
