# Supplementary material for: Salmonella enterica Serovar Typhimurium SPI-1 and SPI-2 Shape the Global Transcriptional Landscape in a Human Intestinal Organoid Model System
Source: mBio. 2021 May 18;12(3):e00399-21. doi: 10.1128/mBio.00399-21 (PMC8262845; doi:10.1128/mBio.00399-21)
Supplement: TABLE S3 [file mbio.00399-21-st003.pdf]

Table S3A: Reactome Pathways Upregulated 2.5h pi

Description	STM		T3SS-1^{mut}		T3SS-2^{mut}	
	gene ratio	p-value	gene ratio	p-value	gene ratio	p-value
Cytokine Signaling in Immune system	0.2194767	4.65E-28	0.199128	2.80E-21	0.2514535	7.85E-26
Signaling by Interleukins	0.2359307	7.40E-23	0.201299	6.70E-15	0.2662338	1.24E-20
Toll Like Receptor 3 (TLR3) Cascade	0.4141414	1.68E-18	0.272727	5.73E-08	0.3939394	5.08E-13
MyD88-independent TLR4 cascade	0.41	2.62E-18	0.28	1.74E-08	0.4	1.40E-13
TRIF(TICAM1)-mediated TLR4 signaling	0.41	2.62E-18	0.28	1.74E-08	0.4	1.40E-13
Toll Like Receptor 4 (TLR4) Cascade	0.3384615	7.70E-16	0.253846	1.38E-08	0.3461538	1.85E-12
TRAF6 mediated induction of NFkB and MAP kinases upon TLR7/8 or 9 activation	0.3870968	2.74E-15	0.247312	3.49E-06	0.3655914	1.77E-10
Toll Like Receptor 7/8 (TLR7/8) Cascade	0.3829787	4.08E-15	0.244681	4.24E-06	0.3617021	2.48E-10
MyD88 dependent cascade initiated on endosome	0.3829787	4.08E-15	0.244681	4.24E-06	0.3617021	2.48E-10
Toll Like Receptor 9 (TLR9) Cascade	0.3673469	1.87E-14	0.234694	8.98E-06	0.3469388	8.93E-10
MyD88:MAL(TIRAP) cascade initiated on plasma membrane	0.3684211	3.92E-14	0.242105	5.14E-06	0.3473684	1.52E-09
Toll Like Receptor TLR6:TLR2 Cascade	0.3684211	3.92E-14	0.242105	5.14E-06	0.3473684	1.52E-09
Toll-like Receptor Cascades	0.2967742	4.54E-14	0.232258	3.78E-08	0.3032258	1.19E-10
Toll Like Receptor TLR1:TLR2 Cascade	0.3571429	1.16E-13	0.234694	8.98E-06	0.3367347	3.77E-09
Toll Like Receptor 2 (TLR2) Cascade	0.3571429	1.16E-13	0.234694	8.98E-06	0.3367347	3.77E-09
Toll Like Receptor 10 (TLR10) Cascade	0.3764706	2.44E-13	0.247059	9.37E-06	0.3529412	5.49E-09
Toll Like Receptor 5 (TLR5) Cascade	0.3764706	2.44E-13	0.247059	9.37E-06	0.3529412	5.49E-09
MyD88 cascade initiated on plasma membrane	0.3764706	2.44E-13	0.247059	9.37E-06	0.3529412	5.49E-09
Interleukin-17 signaling	0.3888889	3.04E-12	0.236111	0.00012	0.3611111	3.32E-08
MAP kinase activation	0.40625	5.56E-12	0.234375	0.00032	0.375	4.82E-08
RAF-independent MAPK1/3 activation	0.6086957	5.61E-10	0.608696	5.45E-10	0.6956522	8.01E-11
Interleukin-10 signaling	0.4255319	5.90E-10	0.446809	6.80E-11	0.4680851	1.25E-09
Death Receptor Signalling	0.2624113	7.12E-10	0.198582	2.93E-05	0.2695035	2.30E-07
TICAM1-dependent activation of IRF3/IRF7	0.8333333	1.45E-09	0.416667	0.00239	0.75	4.93E-07
Interleukin-4 and Interleukin-13 signaling	0.287037	1.62E-09	0.305556	7.24E-11	0.2962963	1.98E-07
Interleukin-1 family signaling	0.2446043	2.42E-08	0.165468	0.00211	0.2805755	4.91E-08
DDX58/IFIH1-mediated induction of interferon-alpha/beta	0.3076923	2.55E-08	0.24359	3.06E-05	0.2820513	3.68E-05
TAK1 activates NFkB by phosphorylation and activation of IKKs complex	0.4545455	2.88E-08	0.242424	0.00643	0.3636364	0.00016
Interleukin-1 signaling	0.2718447	3.69E-08	0.165049	0.00778	0.3106796	5.80E-08
Signaling by the B Cell Receptor (BCR)	0.2589286	6.83E-08	0.196429	0.00024	0.2946429	1.49E-07

MAPK family signaling cascades	0.1843003	1.01E-07	0.139932	0.00173	0.1774744	0.00068
Interferon Signaling	0.2081218	1.24E-07	0.208122	1.17E-07	0.248731	6.92E-08
p75NTR signals via NF-kB	0.625	1.27E-07	0.375	0.00165	0.625	1.46E-06
NF-kB is activated and signals survival	0.6923077	1.54E-07	0.384615	0.0036	0.6923077	1.44E-06
Ovarian tumor domain proteases	0.3947368	2.82E-07	0.236842	0.00464	0.3421053	0.00018
ZBP1(DAI) mediated induction of type I IFNs	0.5238095	3.47E-07	0.428571	3.25E-05	0.5238095	4.69E-06
Negative regulators of DDX58/IFIH1 signaling	0.4117647	3.80E-07	0.294118	0.00047	0.4117647	8.63E-06
Activation of IRF3/IRF7 mediated by TBK1/IKK epsilon	0.5555556	5.87E-07	0.333333	0.00328	0.5555556	6.45E-06
Negative regulation of MAPK pathway	0.375	6.21E-07	0.225	0.00664	0.35	7.45E-05
Downstream signaling events of B Cell Receptor (BCR)	0.2650602	1.69E-06	0.180723	0.00517	0.3253012	2.20E-07
TICAM1, RIP1-mediated IKK complex recruitment	0.5	2.10E-06	0.25	0.02598	0.45	0.00016
Nucleotide-binding domain, leucine rich repeat containing receptor (NLR) signaling pathways	0.3090909	2.59E-06	0.218182	0.00242	0.3090909	7.95E-05
Neutrophil degranulation	0.1503132	3.26E-06	0.11691	0.01493	0.1899791	3.85E-07
RIP-mediated NFkB activation via ZBP1	0.5294118	3.77E-06	0.470588	3.97E-05	0.5294118	3.20E-05
TICAM1,TRAF6-dependent induction of TAK1 complex	0.5294118	3.77E-06	0.117647	0.44587	0.4117647	0.00167
IRAK2 mediated activation of TAK1 complex upon TLR7/8 or 9 stimulation	0.5294118	3.77E-06	0.117647	0.44587	0.4117647	0.00167
Adaptive Immune System	0.1349206	4.86E-06	0.125661	0.00016	0.1746032	1.58E-07
NOD1/2 Signaling Pathway	0.3611111	5.73E-06	0.194444	0.03406	0.3333333	0.00041
Regulation of necroptotic cell death	0.5	6.96E-06	0.333333	0.00328	0.3888889	0.00247
TRAF6-mediated induction of TAK1 complex within TLR4 complex	0.5	6.96E-06	0.111111	0.47598	0.3888889	0.00247
p75 NTR receptor-mediated signalling	0.2371134	7.76E-06	0.14433	0.04243	0.2474227	0.00016
SMAD2/SMAD3:SMAD4 heterotrimer regulates transcription	0.375	8.37E-06	0.125	0.30608	0.3125	0.00214
Interleukin-6 signaling	0.6363636	9.43E-06	0.636364	9.29E-06	0.7272727	3.29E-06
Regulated proteolysis of p75NTR	0.6363636	9.43E-06	0.272727	0.06483	0.5454545	0.00059
Transcriptional activity of SMAD2/SMAD3:SMAD4 heterotrimer	0.3181818	1.36E-05	0.159091	0.08584	0.2954545	0.00088
PIP3 activates AKT signaling	0.1692308	1.54E-05	0.142308	0.00211	0.1538462	0.02829
activated TAK1 mediates p38 MAPK activation	0.4166667	1.61E-05	0.166667	0.15326	0.3333333	0.00377
Diseases of Immune System	0.4166667	1.61E-05	0.333333	0.00069	0.4166667	0.00015
Diseases associated with the TLR signaling cascade	0.4166667	1.61E-05	0.333333	0.00069	0.4166667	0.00015
Interleukin-6 family signaling	0.4166667	1.61E-05	0.375	0.00011	0.4583333	2.40E-05
TRAF6 mediated NF-kB activation	0.4166667	1.61E-05	0.375	0.00011	0.375	0.00082

Interferon alpha/beta signaling	0.2608696	1.87E-05	0.188406	0.00624	0.2463768	0.00149
Signaling by TGF-beta family members	0.2254902	1.87E-05	0.147059	0.03159	0.245098	0.00014
RIPK1-mediated regulated necrosis	0.45	2.05E-05	0.3	0.00588	0.35	0.00491
Regulated Necrosis	0.45	2.05E-05	0.3	0.00588	0.35	0.00491
NRIF signals cell death from the nucleus	0.5	2.31E-05	0.125	0.41481	0.4375	0.00109
MAP3K8 (TPL2)-dependent MAPK1/3 activation	0.5	2.31E-05	0.3125	0.0098	0.5	0.00015
IKK complex recruitment mediated by RIP1	0.4	2.48E-05	0.2	0.06209	0.36	0.00115
Interleukin-12 family signaling	0.2758621	2.54E-05	0.258621	9.96E-05	0.362069	6.58E-07
TNFR1-induced NFkappaB signaling pathway	0.3666667	2.56E-05	0.233333	0.01311	0.3333333	0.00123
MAPK targets/ Nuclear events mediated by MAP kinases	0.3548387	3.66E-05	0.225806	0.01568	0.3548387	0.00038
ERKs are inactivated	0.5384615	4.19E-05	0.307692	0.02197	0.3846154	0.01108
p75NTR recruits signalling complexes	0.5384615	4.19E-05	0.230769	0.0987	0.5384615	0.00022
MAPK1/MAPK3 signaling	0.1653543	4.20E-05	0.137795	0.00466	0.1692913	0.00472
CLEC7A (Dectin-1) signaling	0.2178218	4.98E-05	0.138614	0.05651	0.2574257	4.32E-05
TNF signaling	0.3023256	5.08E-05	0.209302	0.01081	0.3023256	0.00069
IRAK2 mediated activation of TAK1 complex	0.6	6.92E-05	0.1	0.60013	0.4	0.01988
Regulation of TNFR1 signaling	0.3333333	7.10E-05	0.272727	0.00163	0.3333333	0.00071
Cytosolic sensors of pathogen-associated DNA	0.2539683	7.66E-05	0.206349	0.00275	0.2857143	0.00015
Membrane binding and targetting of GAG proteins	0.5	7.74E-05	0.071429	0.72294	0.2857143	0.06563
Synthesis And Processing Of GAG, GAGPOL Polyproteins	0.5	7.74E-05	0.071429	0.72294	0.2857143	0.06563
Downregulation of SMAD2/3:SMAD4 transcriptional activity	0.3913043	7.81E-05	0.173913	0.13661	0.3478261	0.00279
JNK (c-Jun kinases) phosphorylation and activation mediated by activated human TAK1	0.3913043	7.81E-05	0.086957	0.61044	0.2608696	0.0394
Circadian Clock	0.2428571	8.48E-05	0.142857	0.0827	0.2857143	6.73E-05
Fc epsilon receptor (FCERI) signaling	0.1940299	8.71E-05	0.119403	0.1252	0.2238806	0.00019
Signaling by Receptor Tyrosine Kinases	0.1406593	9.20E-05	0.116484	0.01872	0.1736264	6.71E-05
Budding and maturation of HIV virion	0.3448276	0.00011	0.137931	0.24578	0.4137931	3.60E-05
Downstream signal transduction	0.3448276	0.00011	0.275862	0.00272	0.3448276	0.00091
Interferon gamma signaling	0.2173913	0.00011	0.25	2.86E-06	0.2717391	2.25E-05
Diseases of signal transduction	0.1455026	0.00012	0.103175	0.15801	0.1560847	0.00681
IRAK1 recruits IKK complex	0.4666667	0.00013	0.133333	0.3829	0.4	0.00431
IRAK1 recruits IKK complex upon TLR7/8 or 9 stimulation	0.4666667	0.00013	0.133333	0.3829	0.4	0.00431
Intracellular signaling by second messengers	0.1535836	0.00014	0.129693	0.00895	0.1501706	0.03217
Interleukin-27 signaling	0.5454545	0.00014	0.454545	0.0015	0.5454545	0.00059

Signaling by TGF-beta Receptor Complex	0.2328767	0.00015	0.136986	0.10281	0.2876712	3.99E-05
Transcriptional Regulation by TP53	0.1452055	0.00016	0.117808	0.02703	0.1671233	0.0012
Nuclear Events (kinase and transcription factor activation)	0.36	0.00017	0.24	0.01845	0.36	0.00115
Activation of NF-kappaB in B cells	0.238806	0.00017	0.164179	0.02999	0.2835821	0.00011
TNFR2 non-canonical NF-kB pathway	0.2058824	0.00017	0.156863	0.01543	0.245098	0.00014
Interleukin-3, Interleukin-5 and GM-CSF signaling	0.2708333	0.00018	0.1875	0.02174	0.2708333	0.00211
Programmed Cell Death	0.1744186	0.0002	0.139535	0.01486	0.2151163	8.71E-05
MAPK6/MAPK4 signaling	0.2134831	0.00021	0.078652	0.67211	0.2134831	0.00471
FCERI mediated NF-kB activation	0.2195122	0.00021	0.109756	0.28881	0.2439024	0.00068
Assembly Of The HIV Virion	0.4375	0.00022	0.0625	0.76939	0.3125	0.02818
Regulation of TP53 Expression and Degradation	0.2972973	0.00023	0.162162	0.10001	0.2432432	0.02017
RAF/MAP kinase cascade	0.1572581	0.00023	0.125	0.02719	0.1572581	0.02195
TP53 Regulates Transcription of Death Receptors and Ligands	0.5	0.00026	0.5	0.00026	0.4166667	0.00751
Interleukin-35 Signalling	0.5	0.00026	0.416667	0.00239	0.5	0.00107
TGF-beta receptor signaling activates SMADs	0.3125	0.00028	0.15625	0.14343	0.40625	2.16E-05
TP53 Regulates Transcription of Cell Death Genes	0.2727273	0.00029	0.272727	0.00029	0.2954545	0.00088
Apoptosis	0.1715976	0.00033	0.136095	0.02208	0.2130178	0.00013
IL-6-type cytokine receptor ligand interactions	0.4117647	0.00035	0.352941	0.00236	0.4117647	0.00167
Signaling by MET	0.2151899	0.00041	0.151899	0.0412	0.2151899	0.0067
Signaling by Activin	0.4615385	0.00045	0.153846	0.31706	0.3846154	0.01108
Chemokine receptors bind chemokines	0.25	0.00069	0.291667	4.00E-05	0.2708333	0.00211
Interleukin-12 signaling	0.25	0.00069	0.25	0.00068	0.3541667	1.07E-05
Regulation of IFNG signaling	0.4285714	0.00073	0.571429	6.23E-06	0.5714286	4.36E-05
TRAF3-dependent IRF activation pathway	0.4285714	0.00073	0.357143	0.00521	0.5	0.0004
ER-Phagosome pathway	0.2048193	0.00074	0.072289	0.74532	0.2168675	0.00492
RHO GTPases activate IQGAPs	0.3	0.00078	0.266667	0.00342	0.3	0.00479
Regulation of TP53 Degradation	0.2777778	0.00079	0.138889	0.20344	0.2222222	0.0456
Cell junction organization	0.1978022	0.00081	0.131868	0.09866	0.1868132	0.0263
Transcriptional regulation of white adipocyte differentiation	0.202381	0.00085	0.130952	0.11468	0.202381	0.01245
Antigen processing-Cross presentation	0.1919192	0.00086	0.080808	0.64637	0.2222222	0.00144
MAPK3 (ERK1) activation	0.5	0.00089	0.6	6.84E-05	0.7	2.12E-05
Interleukin-20 family signaling	0.32	0.00095	0.32	0.00094	0.4	0.00023
Antiviral mechanism by IFN-stimulated genes	0.2051282	0.00103	0.179487	0.00721	0.2692308	0.00012
PTEN Regulation	0.1714286	0.00106	0.107143	0.24279	0.1571429	0.07187

TRAF6 mediated IRF7 activation in TLR7/8 or 9 signaling	0.4	0.00112	0.066667	0.74723	0.2666667	0.08174
Intrinsic Pathway for Apoptosis	0.25	0.00115	0.181818	0.03503	0.3409091	5.89E-05
Downregulation of TGF-beta receptor signaling	0.3076923	0.00127	0.153846	0.18863	0.4230769	5.98E-05
Signaling by PDGF	0.2241379	0.00127	0.206897	0.00386	0.2241379	0.01184
C-type lectin receptors (CLRs)	0.1690141	0.0013	0.119718	0.11471	0.2112676	0.00054
Signaling by ERBB4	0.2444444	0.0014	0.088889	0.56361	0.2444444	0.01036
Signaling by Leptin	0.4545455	0.00152	0.454545	0.0015	0.4545455	0.00483
NOTCH4 Activation and Transmission of Signal to the Nucleus	0.4545455	0.00152	0.181818	0.24972	0.3636364	0.02847
Spry regulation of FGF signaling	0.375	0.00167	0.125	0.41481	0.25	0.09959
ERK/MAPK targets	0.3181818	0.00206	0.181818	0.12074	0.3181818	0.00882
NOTCH2 Activation and Transmission of Signal to the Nucleus	0.3181818	0.00206	0.090909	0.58579	0.2272727	0.09635
Regulation of signaling by CBL	0.3181818	0.00206	0.136364	0.30215	0.2727273	0.03211
Signaling by PTK6	0.2222222	0.00209	0.12963	0.19044	0.2777778	0.00074
Signaling by Non-Receptor Tyrosine Kinases	0.2222222	0.00209	0.12963	0.19044	0.2777778	0.00074
Membrane Trafficking	0.1210191	0.00219	0.093949	0.29972	0.1544586	0.00087
Cell death signalling via NRAGE, NRIF and NADE	0.1973684	0.00219	0.105263	0.3471	0.2105263	0.01038
Caspase activation via Death Receptors in the presence of ligand	0.3529412	0.00238	0.294118	0.01291	0.2941176	0.03628
ISG15 antiviral mechanism	0.1971831	0.00306	0.183099	0.00798	0.2676056	0.00026
TNF receptor superfamily (TNFSF) members mediating non-canonical NF-kB pathway	0.3333333	0.00331	0.333333	0.00328	0.3888889	0.00247
TCR signaling	0.1680672	0.0034	0.092437	0.47175	0.1848739	0.01426
Regulation of lipid metabolism by Peroxisome proliferator-activated receptor alpha (PPARalpha)	0.1680672	0.0034	0.084034	0.60195	0.1764706	0.02655
Activation of BH3-only proteins	0.2666667	0.00347	0.2	0.04266	0.3333333	0.00123
Cellular responses to stress	0.1267606	0.00357	0.086854	0.54675	0.1690141	0.00032
Signaling by Erythropoietin	0.2916667	0.00358	0.25	0.01511	0.2083333	0.12914
Growth hormone receptor signaling	0.2916667	0.00358	0.25	0.01511	0.2916667	0.01463
Dissolution of Fibrin Clot	0.3846154	0.00364	0.384615	0.0036	0.4615385	0.0018
Cell-Cell communication	0.1627907	0.00403	0.131783	0.05745	0.1782946	0.01874
Activated NOTCH1 Transmits Signal to the Nucleus	0.2580645	0.00433	0.064516	0.76829	0.2258065	0.05527
Negative regulation of FGFR4 signaling	0.2580645	0.00433	0.096774	0.51804	0.1290323	0.4756
NOTCH3 Activation and Transmission of Signal to the Nucleus	0.28	0.0046	0.08	0.65634	0.24	0.05691

Antigen Presentation: Folding, assembly and peptide loading of class I MHC	0.28	0.0046	0.16	0.17062	0.24	0.05691
Vesicle-mediated transport	0.1169415	0.00477	0.095952	0.2325	0.1484258	0.00283
Downstream TCR signaling	0.1734694	0.00478	0.081633	0.63489	0.2040816	0.00645
Endosomal Sorting Complex Required For Transport (ESCRT)	0.25	0.00533	0.0625	0.78352	0.28125	0.00763
Gene and protein expression by JAK-STAT signaling after Interleukin-12 stimulation	0.2307692	0.00565	0.205128	0.01787	0.3589744	5.40E-05
PPARA activates gene expression	0.1623932	0.00621	0.08547	0.57993	0.1794872	0.02235
Regulation of TP53 Activity	0.15	0.00642	0.125	0.06608	0.1375	0.20034
Signaling by NOTCH2	0.2424242	0.00651	0.090909	0.56131	0.1818182	0.1657
Oncogene Induced Senescence	0.2424242	0.00651	0.090909	0.56131	0.1515152	0.31951
Negative regulation of FGFR1 signaling	0.2424242	0.00651	0.090909	0.56131	0.1212121	0.5263
Nuclear signaling by ERBB4	0.2592593	0.00728	0.111111	0.42523	0.2592593	0.02778
Caspase activation via extrinsic apoptotic signalling pathway	0.2592593	0.00728	0.222222	0.02657	0.1851852	0.18623
Signaling by NOTCH1 HD Domain Mutants in Cancer	0.3333333	0.00732	0.066667	0.74723	0.2666667	0.08174
Constitutive Signaling by NOTCH1 HD Domain Mutants	0.3333333	0.00732	0.066667	0.74723	0.2666667	0.08174
Regulation of innate immune responses to cytosolic DNA	0.3333333	0.00732	0.066667	0.74723	0.2666667	0.08174
Negative regulation of MET activity	0.2857143	0.00772	0.142857	0.27741	0.1904762	0.21032
Signaling by FGFR4	0.2195122	0.00797	0.073171	0.70886	0.1463415	0.32088
MASTL Facilitates Mitotic Progression	0.4	0.00804	0.2	0.2161	0.4	0.01988
Interleukin-21 signaling	0.4	0.00804	0.3	0.05033	0.3	0.09562
Cellular responses to external stimuli	0.1192843	0.00836	0.089463	0.46225	0.1689861	9.57E-05
Rho GTPase cycle	0.1521739	0.00882	0.108696	0.22592	0.1666667	0.03816
RAB geranylgeranylation	0.1875	0.00891	0.078125	0.67021	0.265625	0.0006
Transcriptional regulation by RUNX3	0.1666667	0.00898	0.083333	0.61133	0.2083333	0.00507
Pink/Parkin Mediated Mitophagy	0.2727273	0.00984	0.090909	0.58579	0.2727273	0.03211
TGF-beta receptor signaling in EMT (epithelial to mesenchymal transition)	0.3125	0.00989	0.0625	0.76939	0.375	0.00623
Glycogen storage diseases	0.3125	0.00989	0.125	0.41481	0.125	0.55763
Signaling by NOTCH1	0.1780822	0.01025	0.09589	0.45924	0.1780822	0.0664
Signaling by FGFR1	0.2	0.0104	0.06	0.82574	0.12	0.5097
Mitophagy	0.2413793	0.01097	0.103448	0.47257	0.2068966	0.10385
Negative regulation of FGFR3 signaling	0.2413793	0.01097	0.068966	0.73504	0.137931	0.42297
TRAF6 mediated IRF7 activation	0.2413793	0.01097	0.172414	0.10443	0.2413793	0.04

Signaling by NOTCH1 PEST Domain Mutants in Cancer	0.1896552	0.01103	0.068966	0.75939	0.1551724	0.20856
Signaling by NOTCH1 in Cancer	0.1896552	0.01103	0.068966	0.75939	0.1551724	0.20856
Constitutive Signaling by NOTCH1 PEST Domain Mutants	0.1896552	0.01103	0.068966	0.75939	0.1551724	0.20856
Signaling by NOTCH1 HD+PEST Domain Mutants in Cancer	0.1896552	0.01103	0.068966	0.75939	0.1551724	0.20856
Constitutive Signaling by NOTCH1 HD+PEST Domain Mutants	0.1896552	0.01103	0.068966	0.75939	0.1551724	0.20856
RAB GEFs exchange GTP for GDP on RABs	0.1666667	0.01119	0.088889	0.53633	0.1666667	0.08236
Rab regulation of trafficking	0.1532258	0.0115	0.08871	0.52817	0.1532258	0.10811
Endosomal/Vacuolar pathway	0.3636364	0.01176	0.181818	0.24972	0.2727273	0.12079
Regulation by c-FLIP	0.3636364	0.01176	0.363636	0.01167	0.2727273	0.12079
Myoclonic epilepsy of Lafora	0.3636364	0.01176	0.090909	0.63518	0.1818182	0.36087
Type I hemidesmosome assembly	0.3636364	0.01176	0.363636	0.01167	0.4545455	0.00483
SUMOylation of immune response proteins	0.3636364	0.01176	0.454545	0.0015	0.5454545	0.00059
CASP8 activity is inhibited	0.3636364	0.01176	0.363636	0.01167	0.2727273	0.12079
Dimerization of procaspase-8	0.3636364	0.01176	0.363636	0.01167	0.2727273	0.12079
VEGFA-VEGFR2 Pathway	0.1616162	0.01198	0.111111	0.24745	0.2222222	0.00144
Cyclin D associated events in G1	0.2045455	0.01274	0.136364	0.18366	0.1818182	0.12032
G1 Phase	0.2045455	0.01274	0.136364	0.18366	0.1818182	0.12032
Transport of connexons to the plasma membrane	0.2941176	0.01302	0.411765	0.00034	0.3529412	0.00872
Regulation of MECP2 expression and activity	0.2333333	0.01325	0.1	0.49556	0.1333333	0.44949
Dectin-1 mediated noncanonical NF-kB signaling	0.1833333	0.01415	0.116667	0.26983	0.2333333	0.00636
HSP90 chaperone cycle for steroid hormone receptors (SHR)	0.1886792	0.01556	0.150943	0.08838	0.2641509	0.00188
Regulation of PTEN gene transcription	0.1803279	0.01594	0.147541	0.08238	0.1147541	0.5496
Josephin domain DUBs	0.3333333	0.01644	NA	NA	0.3333333	0.03896
NF-kB activation through FADD/RIP-1 pathway mediated by caspase-8 and -10	0.3333333	0.01644	0.333333	0.01632	0.4166667	0.00751
Signaling by NTRK1 (TRKA)	0.1666667	0.01748	0.089744	0.53074	0.2307692	0.00242
RAF activation	0.24	0.01863	0.12	0.3765	0.24	0.05691
Constitutive Signaling by AKT1 E17K in Cancer	0.24	0.01863	0.2	0.06209	0.28	0.01838
Antigen activates B Cell Receptor (BCR) leading to generation of second messengers	0.21875	0.0188	0.21875	0.0186	0.1875	0.14898
Gap junction trafficking and regulation	0.1914894	0.01935	0.191489	0.0191	0.2765957	0.00172
Regulation of mRNA stability by proteins that bind AU-rich elements	0.1590909	0.02048	0.090909	0.51012	0.1931818	0.01939
Signaling by FGFR3	0.2	0.02092	0.05	0.87659	0.15	0.30005

Signaling by moderate kinase activity BRAF mutants	0.2	0.02092	0.125	0.27015	0.175	0.16293
Paradoxical activation of RAF signaling by kinase inactive BRAF	0.2	0.02092	0.125	0.27015	0.175	0.16293
Constitutive Signaling by Ligand-Responsive EGFR Cancer Variants	0.2631579	0.02114	NA	NA	0.2631579	0.05639
Signaling by EGFR in Cancer	0.2631579	0.02114	NA	NA	0.2631579	0.05639
Signaling by Ligand-Responsive EGFR Variants in Cancer	0.2631579	0.02114	NA	NA	0.2631579	0.05639
LDL clearance	0.2631579	0.02114	0.210526	0.0785	0.3684211	0.00353
PI3K/AKT Signaling in Cancer	0.1546392	0.02138	0.175258	0.0042	0.1443299	0.20904
Hemostasis	0.1112903	0.0221	0.119355	0.00338	0.1564516	0.00056
Initiation of Nuclear Envelope Reformation	0.3076923	0.02213	0.230769	0.0987	0.2307692	0.17709
Nuclear Envelope Reassembly	0.3076923	0.02213	0.230769	0.0987	0.2307692	0.17709
Triglyceride biosynthesis	0.3076923	0.02213	0.307692	0.02197	0.0769231	0.79197
Erythropoietin activates RAS	0.3076923	0.02213	0.153846	0.31706	0.3076923	0.05135
Cell-cell junction organization	0.171875	0.02233	0.0625	0.82329	0.15625	0.1863
Transcriptional activation of mitochondrial biogenesis	0.1785714	0.02242	0.089286	0.55012	0.1785714	0.09788
Other interleukin signaling	0.2307692	0.02248	0.192308	0.07157	0.3076923	0.00652
Signaling by VEGF	0.1495327	0.02389	0.102804	0.33422	0.2056075	0.00401
Degradation of beta-catenin by the destruction complex	0.1585366	0.02563	0.085366	0.58514	0.2073171	0.00981
Signaling by NOTCH4	0.1585366	0.02563	0.073171	0.73469	0.195122	0.02094
Negative regulation of FGFR2 signaling	0.2058824	0.02581	0.117647	0.3471	0.1470588	0.34302
Signaling by Hippo	0.25	0.0262	0.15	0.25289	0.2	0.18583
TP53 Regulates Transcription of Genes Involved in Cytochrome C Release	0.25	0.0262	0.3	0.00588	0.25	0.06842
Listeria monocytogenes entry into host cells	0.25	0.0262	0.1	0.53309	0.25	0.06842
Deactivation of the beta-catenin transactivating complex	0.1904762	0.02751	0.095238	0.50782	0.2142857	0.04332
Beta-catenin independent WNT signaling	0.137931	0.02812	0.075862	0.73427	0.1793103	0.01214
Deubiquitination	0.1212121	0.02878	0.087542	0.53094	0.1346801	0.14442
SEMA3A-Plexin repulsion signaling by inhibiting Integrin adhesion	0.2857143	0.02888	0.357143	0.00521	0.2142857	0.20749
TP53 regulates transcription of several additional cell death genes whose specific roles in p53-dependent apoptosis remain uncertain	0.2857143	0.02888	0.214286	0.1178	0.3571429	0.01565
Interleukin-15 signaling	0.2857143	0.02888	0.214286	0.1178	0.3571429	0.01565

EPH-Ephrin signaling	0.1521739	0.02903	0.163043	0.01335	0.2717391	2.25E-05
The role of GTSE1 in G2/M progression after G2 checkpoint	0.16	0.02931	0.093333	0.48821	0.2133333	0.00913
NIK-->noncanonical NF-kB signaling	0.1694915	0.03123	0.101695	0.41432	0.220339	0.01365
Signaling by SCF-KIT	0.1860465	0.0313	0.232558	0.00334	0.2790698	0.00235
Sphingolipid de novo biosynthesis	0.1860465	0.0313	0.162791	0.07776	0.2325581	0.02004
EPH-ephrin mediated repulsion of cells	0.1764706	0.03161	0.176471	0.03123	0.2352941	0.01038
CD209 (DC-SIGN) signaling	0.2380952	0.03198	0.190476	0.10573	0.2857143	0.02576
TP53 regulates transcription of additional cell cycle genes whose exact role in the p53 pathway remain uncertain	0.2380952	0.03198	0.238095	0.03172	0.2857143	0.02576
Interleukin-37 signaling	0.2380952	0.03198	0.238095	0.03172	0.2857143	0.02576
G2/M Transition	0.128866	0.03277	0.07732	0.73118	0.1443299	0.1094
RHO GTPases Activate WASPs and WAVEs	0.1944444	0.03441	0.111111	0.3882	0.2777778	0.00552
Cellular Senescence	0.1282051	0.03458	0.082051	0.64541	0.1282051	0.29105
Interleukin-2 family signaling	0.1818182	0.03542	0.136364	0.18366	0.2045455	0.05619
TP53 Regulates Metabolic Genes	0.1511628	0.03627	0.081395	0.63625	0.1976744	0.01562
Mitotic G2-G2/M phases	0.127551	0.03648	0.076531	0.74535	0.1428571	0.11992
Activation of BAD and translocation to mitochondria	0.2666667	0.03671	0.133333	0.3829	0.4	0.00431
YAP1- and WWTR1 (TAZ)-stimulated gene expression	0.2666667	0.03671	0.133333	0.3829	0.2	0.23893
phosphorylation site mutants of CTNNB1 are not targeted to the proteasome by the destruction complex	0.2666667	0.03671	0.133333	0.3829	0.2	0.23893
Misspliced GSK3beta mutants stabilize beta-catenin	0.2666667	0.03671	0.133333	0.3829	0.2	0.23893
S33 mutants of beta-catenin aren't phosphorylated	0.2666667	0.03671	0.133333	0.3829	0.2	0.23893
S37 mutants of beta-catenin aren't phosphorylated	0.2666667	0.03671	0.133333	0.3829	0.2	0.23893
S45 mutants of beta-catenin aren't phosphorylated	0.2666667	0.03671	0.133333	0.3829	0.2	0.23893
T41 mutants of beta-catenin aren't phosphorylated	0.2666667	0.03671	0.133333	0.3829	0.2	0.23893
The NLRP3 inflammasome	0.2666667	0.03671	0.266667	0.03646	0.3333333	0.02132
InlB-mediated entry of Listeria monocytogenes into host cell	0.2666667	0.03671	0.066667	0.74723	0.2	0.23893
N-Glycan antennae elongation	0.2666667	0.03671	0.066667	0.74723	0.3333333	0.02132
Signaling by NOTCH	0.1234043	0.03777	0.07234	0.8287	0.1489362	0.05687
Signaling by RAS mutants	0.1698113	0.03942	0.113208	0.31792	0.1509434	0.25069
Gap junction trafficking	0.1777778	0.0399	0.177778	0.03946	0.2666667	0.00356
Synthesis of active ubiquitin: roles of E1 and E2 enzymes	0.2	0.04304	0.066667	0.75215	0.3	0.00479
Molecules associated with elastic fibres	0.1842105	0.04472	0.210526	0.01534	0.1578947	0.25925

Microtubule-dependent trafficking of connexons from Golgi to the plasma membrane	0.25	0.04566	0.375	0.00165	0.3125	0.02818
Glycogen synthesis	0.25	0.04566	0.0625	0.76939	0.1875	0.27109
Rap1 signalling	0.25	0.04566	0.125	0.41481	0.3125	0.02818
Ub-specific processing proteases	0.1227273	0.04643	0.077273	0.74136	0.1272727	0.29036
Transcriptional Regulation by MECP2	0.1636364	0.04844	0.109091	0.34988	0.1090909	0.60631
Signaling by NTRKs	0.1414141	0.04986	0.10101	0.36646	0.2020202	0.00724
COPI-mediated anterograde transport	0.1414141	0.04986	0.10101	0.36646	0.1616162	0.09271
PI5P, PP2A and IER3 Regulate PI3K/AKT Signaling	0.1414141	0.04986	0.161616	0.01175	0.1313131	0.33388
NOTCH1 Intracellular Domain Regulates Transcription	0.1702128	0.04994	0.106383	0.39452	0.1702128	0.15902
Extracellular matrix organization	0.1162791	0.05142	0.156146	6.26E-05	0.1461794	0.04732
Non-integrin membrane-ECM interactions	0.1525424	0.07033	0.237288	0.00044	0.2542373	0.00198
Peptide ligand-binding receptors	0.1210526	0.07042	0.163158	0.0005	0.1210526	0.40743
cGMP effects	0.0555556	0.80878	0.388889	0.00052	NA	NA
Formation of tubulin folding intermediates by CCT/TriC	0.1304348	0.32822	0.304348	0.0027	0.2608696	0.0394
Nitric oxide stimulates guanylate cyclase	0.0416667	0.88991	0.291667	0.00353	NA	NA
Class A/1 (Rhodopsin-like receptors)	0.0987654	0.26473	0.12963	0.00626	0.0833333	0.97115
Prolactin receptor signaling	0.2	0.1388	0.333333	0.00726	0.2	0.23893
Gap junction assembly	0.1764706	0.0726	0.235294	0.00777	0.2647059	0.01159
Degradation of the extracellular matrix	0.1142857	0.16554	0.15	0.01011	0.15	0.11223
Negative regulation of the PI3K/AKT network	0.1320755	0.07928	0.160377	0.01033	0.1226415	0.43021
Erythropoietin activates Phosphoinositide-3-kinase (PI3K)	0.2727273	0.06517	0.363636	0.01167	0.1818182	0.36087
Vitamin B5 (pantothenate) metabolism	0.1176471	0.44701	0.294118	0.01291	0.2352941	0.11907
Cooperation of Prefoldin and TriC/CCT in actin and tubulin folding	0.1	0.49705	0.233333	0.01311	0.2	0.11801
GPCR ligand binding	0.0919037	0.39853	0.118162	0.01371	0.0853392	0.98177
Metabolism of vitamins and cofactors	0.0899471	0.49473	0.137566	0.01376	0.1322751	0.23855
Cytosolic tRNA aminoacylation	0.0833333	0.63517	0.25	0.01511	0.375	0.00082
Synthesis of very long-chain fatty acyl-CoAs	0.0833333	0.63517	0.25	0.01511	0.2083333	0.12914
Apoptotic cleavage of cellular proteins	0.1315789	0.2375	0.210526	0.01534	0.1578947	0.25925
Uptake and actions of bacterial toxins	0.1612903	0.13067	0.225806	0.01568	0.1935484	0.13306
Assembly of collagen fibrils and other multimeric structures	0.1311475	0.16286	0.180328	0.01571	0.1967213	0.0397
Caspase-mediated cleavage of cytoskeletal proteins	0.1666667	0.28434	0.333333	0.01632	0.25	0.14807
Constitutive Signaling by Aberrant PI3K in Cancer	0.1267606	0.16741	0.169014	0.01948	0.0985915	0.71132

Metabolism of water-soluble vitamins and cofactors	0.097561	0.39444	0.146341	0.02075	0.1463415	0.15725
Ephrin signaling	0.2105263	0.079	0.263158	0.02096	0.3157895	0.01569
Prefoldin mediated transfer of substrate to CCT/TriC	0.1153846	0.40239	0.230769	0.02226	0.1923077	0.16626
Post-chaperonin tubulin folding pathway	0.15	0.25393	0.25	0.02598	0.2	0.18583
Inflammasomes	0.2	0.09221	0.25	0.02598	0.25	0.06842
Syndecan interactions	0.1481481	0.2083	0.222222	0.02657	0.2222222	0.0784
Tryptophan catabolism	0.0714286	0.72375	0.285714	0.02867	0.1428571	0.48409
Chaperonin-mediated protein folding	0.0860215	0.57711	0.150538	0.03103	0.1290323	0.36646
Nuclear Envelope Breakdown	0.1372549	0.15609	0.176471	0.03123	0.1568627	0.21833
Signaling by BMP	0.1785714	0.09343	0.214286	0.03139	0.1785714	0.20701
Integrin cell surface interactions	0.1294118	0.12327	0.152941	0.03286	0.2	0.01397
Apoptotic execution phase	0.0961538	0.48479	0.173077	0.03495	0.1346154	0.37848
p130Cas linkage to MAPK signaling for integrins	0.2	0.1388	0.266667	0.03646	0.1333333	0.52181
Smooth Muscle Contraction	0.0810811	0.64187	0.189189	0.03895	0.1081081	0.61978
Fatty acyl-CoA biosynthesis	0.0540541	0.84799	0.189189	0.03895	0.1351351	0.41383
Triglyceride metabolism	0.1081081	0.41027	0.189189	0.03895	0.1621622	0.23944
Elastic fibre formation	0.1555556	0.09522	0.177778	0.03946	0.1333333	0.40554
Laminin interactions	0.1333333	0.26692	0.2	0.04266	0.2666667	0.0162
EPHA-mediated growth cone collapse	0.1	0.49705	0.2	0.04266	0.2666667	0.0162
Regulation of TP53 Activity through Acetylation	0.1666667	0.11759	0.2	0.04266	0.1666667	0.2506
Collagen formation	0.0888889	0.53886	0.144444	0.04904	0.1444444	0.21928
Platelet homeostasis	0.0777778	0.68579	0.144444	0.04904	0.0555556	0.98086
Protein folding	0.0808081	0.64882	0.141414	0.04912	0.1212121	0.45315
Response to elevated platelet cytosolic Ca2+	0.0970149	0.39463	0.119403	0.1252	0.2164179	0.00044
Platelet activation, signaling and aggregation	0.1145038	0.07868	0.091603	0.43925	0.1832061	0.00053
EPHB-mediated forward signaling	0.1666667	0.07079	0.142857	0.1574	0.3095238	0.00053
HIV Infection	0.1163793	0.07866	0.107759	0.16204	0.1853448	0.00079
Axon guidance	0.0998185	0.16996	0.094374	0.30193	0.1578947	0.0008
RHO GTPases activate CIT	0.1	0.5343	0.15	0.25289	0.4	0.00098
RHO GTPases Activate ROCKs	0.05	0.84092	0.1	0.53309	0.4	0.00098
Platelet degranulation	0.0930233	0.46055	0.116279	0.15695	0.2093023	0.00116
HIV Life Cycle	0.1192053	0.11188	0.119205	0.11023	0.1986755	0.00154
Regulation of actin dynamics for phagocytic cup formation	0.147541	0.08326	0.081967	0.62774	0.2459016	0.00282
PCP/CE pathway	0.1413043	0.05763	0.076087	0.70569	0.2173913	0.00304

Late Phase of HIV Life Cycle	0.1304348	0.05713	0.115942	0.15009	0.1956522	0.00323
Class I MHC mediated antigen processing & presentation	0.1105121	0.07213	0.097035	0.2807	0.1590296	0.00454
Sema4D in semaphorin signaling	0.16	0.17156	0.16	0.17062	0.32	0.005
Infectious disease	0.0994764	0.2282	0.094241	0.34491	0.1570681	0.00558
Oxygen-dependent proline hydroxylation of Hypoxia-inducible Factor Alpha	0.1515152	0.06067	0.045455	0.93653	0.2272727	0.00625
Sema4D induced cell migration and growth-cone collapse	0.1428571	0.27853	0.142857	0.27741	0.3333333	0.00666
Regulation of RUNX2 expression and activity	0.1506849	0.05244	0.082192	0.62519	0.2191781	0.00699
Fcgamma receptor (FCGR) dependent phagocytosis	0.1395349	0.07168	0.081395	0.63625	0.2093023	0.00725
BMAL1:CLOCK,NPAS2 activates circadian gene expression	0.1851852	0.0824	0.148148	0.20721	0.2962963	0.00835
Transcriptional regulation by RUNX2	0.1157025	0.1737	0.082645	0.6234	0.1900826	0.00893
Regulation of Hypoxia-inducible Factor (HIF) by oxygen	0.1466667	0.06168	0.04	0.96546	0.2133333	0.00913
Cellular response to hypoxia	0.1466667	0.06168	0.04	0.96546	0.2133333	0.00913
Gastrin-CREB signalling pathway via PKC and MAPK	0.2222222	0.06682	0.166667	0.20498	0.3333333	0.01185
Clathrin-mediated endocytosis	0.1214286	0.10576	0.1	0.34141	0.1785714	0.01443
RHO GTPases activate PAKs	0.0833333	0.63517	0.166667	0.15326	0.2916667	0.01463
PTK6 Regulates RHO GTPases, RAS GTPase and MAP kinases	0.2142857	0.11838	0.142857	0.35027	0.3571429	0.01565
Negative regulation of NOTCH4 signaling	0.1481481	0.09715	0.037037	0.95648	0.2222222	0.01632
Signaling by Rho GTPases	0.1013514	0.17035	0.083333	0.65123	0.1463964	0.01857
p75NTR regulates axonogenesis	0.1	0.60096	0.2	0.2161	0.4	0.01988
Formation of Incision Complex in GG-NER	0.0930233	0.52855	0.093023	0.52679	0.2325581	0.02004
Detoxification of Reactive Oxygen Species	0.1621622	0.10081	0.108108	0.40863	0.2432432	0.02017
Signaling by ERBB2	0.14	0.1449	0.02	0.98987	0.22	0.02255
Iron uptake and transport	0.0862069	0.58411	0.137931	0.13138	0.2068966	0.02784
CD28 co-stimulation	0.1212121	0.32801	0.151515	0.15765	0.2424242	0.02833
Plasma lipoprotein clearance	0.1818182	0.06434	0.121212	0.32655	0.2424242	0.02833
Formation of annular gap junctions	0.2727273	0.06517	0.090909	0.63518	0.3636364	0.02847
Calcineurin activates NFAT	0.2727273	0.06517	0.181818	0.24972	0.3636364	0.02847
Semaphorin interactions	0.1076923	0.34358	0.153846	0.05498	0.2	0.0293
Autodegradation of the E3 ubiquitin ligase COP1	0.1538462	0.08172	0.038462	0.94931	0.2115385	0.02959
Host Interactions of HIV factors	0.125	0.09409	0.085938	0.57185	0.171875	0.03095
RHO GTPase Effectors	0.0917722	0.42789	0.075949	0.79799	0.1487342	0.03198
Gap junction degradation	0.25	0.08142	0.083333	0.66715	0.3333333	0.03896

FCGR activation	0.0833333	0.66798	0.083333	0.66715	0.3333333	0.03896
PECAM1 interactions	0.1666667	0.28434	0.25	0.081	0.3333333	0.03896
Repression of WNT target genes	0.1666667	0.28434	0.25	0.081	0.3333333	0.03896
VEGFR2 mediated vascular permeability	0.137931	0.24701	0.137931	0.24578	0.2413793	0.04
Regulation of RAS by GAPs	0.1323529	0.13889	0.029412	0.9854	0.1911765	0.04079
Regulation of RUNX3 expression and activity	0.1454545	0.10544	0.054545	0.87142	0.2	0.04289
tRNA Aminoacylation	0.0714286	0.72573	0.142857	0.1574	0.2142857	0.04332
Regulation of PTEN stability and activity	0.1304348	0.1481	0.057971	0.8652	0.1884058	0.04524
COPI-independent Golgi-to-ER retrograde traffic	0.1632653	0.0615	0.163265	0.06086	0.2040816	0.04618
DAP12 signaling	0.1666667	0.11759	0.133333	0.26563	0.2333333	0.04725
SUMOylation of intracellular receptors	0.1333333	0.26692	0.033333	0.93624	0.2333333	0.04725
Interconversion of nucleotide di- and triphosphates	0.1	0.49705	0.066667	0.75215	0.2333333	0.04725
Antigen processing: Ubiquitination & Proteasome degradation	0.0906149	0.45796	0.090615	0.45319	0.1456311	0.04775
AUF1 (hnRNP D0) binds and destabilizes mRNA	0.1428571	0.11411	0.035714	0.96267	0.1964286	0.04812
Hh mutants that don't undergo autocatalytic processing are degraded by ERAD	0.125	0.21738	0.017857	0.99417	0.1964286	0.04812

Table S3A. Enriched Reactome pathways from up-regulated genes at 2.5h pi. Significant Reactome pathways using up-regulated DEGs from STM, T3SS-1^{mut} or T3SS-2^{mut}-infected HIOs at 2.5h.

Table S3B: Reactome Pathways Downregulated 2.5h pi

Description	STM		T3SS-1mut		T3SS-2mut	
	gene ratio	p-value	gene ratio	p-value	gene ratio	p-value
Mitochondrial translation initiation	0.264368	2.40E-08	0.126437	0.03474	0.091954	0.4138
Mitochondrial translation	0.247312	9.26E-08	0.11828	0.05248	0.086022	0.4893
Metabolism of RNA	0.126113	1.04E-07	0.0727	0.35054	0.074184	0.7803
Mitochondrial translation elongation	0.252874	1.15E-07	0.126437	0.03474	0.08046	0.5697
Mitochondrial translation termination	0.252874	1.15E-07	0.126437	0.03474	0.08046	0.5697
Mitotic Prometaphase	0.181818	1.45E-07	0.045455	0.93222	0.106061	0.1267
Cell Cycle	0.123245	7.87E-07	0.049922	0.98097	0.087363	0.3051
Cell Cycle, Mitotic	0.125	3.52E-06	0.052239	0.9516	0.080224	0.5646
Cell Cycle Checkpoints	0.143345	9.73E-06	0.054608	0.85977	0.071672	0.7617
AURKA Activation by TPX2	0.232877	1.05E-05	0.054795	0.74599	0.164384	0.0142
Cilium Assembly	0.160804	1.09E-05	0.055276	0.81059	0.140704	0.003
mRNA Splicing	0.162304	1.23E-05	0.078534	0.32912	0.099476	0.211
Processing of Capped Intronless Pre-mRNA	0.357143	1.28E-05	0.25	0.00228	0.178571	0.0725
tRNA processing	0.198113	1.50E-05	0.075472	0.44168	0.075472	0.6405
Processing of Capped Intron-Containing Pre-mRNA	0.148148	2.03E-05	0.065844	0.60323	0.090535	0.3318
Regulation of PLK1 Activity at G2/M Transition	0.204545	3.86E-05	0.045455	0.86172	0.147727	0.0252
Resolution of Sister Chromatid Cohesion	0.177419	5.67E-05	0.024194	0.9925	0.072581	0.6887
M Phase	0.124682	7.94E-05	0.035623	0.99848	0.07888	0.6013
Translation	0.134021	9.21E-05	0.09622	0.04248	0.054983	0.9682
mRNA Splicing - Major Pathway	0.153005	9.41E-05	0.081967	0.27197	0.092896	0.3201
G2/M Transition	0.149485	0.000108	0.056701	0.78415	0.103093	0.162
Mitotic Spindle Checkpoint	0.178571	0.000111	0.053571	0.78809	0.089286	0.4285
Organelle biogenesis and maintenance	0.132653	0.000115	0.061224	0.72516	0.132653	0.0016
Amplification of signal from the kinetochores	0.1875	0.000127	0.03125	0.96459	0.083333	0.5262
Amplification of signal from unattached kinetochores via a MAD2 inhibitory signal	0.1875	0.000127	0.03125	0.96459	0.083333	0.5262
Mitotic G2-G2/M phases	0.147959	0.00013	0.056122	0.79503	0.102041	0.1733
Recruitment of mitotic centrosome proteins and complexes	0.195122	0.000184	0.073171	0.49517	0.158537	0.0146
Centrosome maturation	0.195122	0.000184	0.073171	0.49517	0.158537	0.0146
Processing of Intronless Pre-mRNAs	0.368421	0.000214	0.315789	0.00127	0.210526	0.0632
SUMOylation	0.144385	0.00033	0.053476	0.83304	0.085561	0.4555
Loss of Nlp from mitotic centrosomes	0.2	0.000351	0.042857	0.86715	0.142857	0.0559

Loss of proteins required for interphase microtubule organization from the centrosome	0.2	0.000351	0.042857	0.86715	0.142857	0.0559
Anchoring of the basal body to the plasma membrane	0.173469	0.000506	0.05102	0.81034	0.132653	0.0538
tRNA modification in the nucleus and cytosol	0.232558	0.000704	0.069767	0.57263	0.046512	0.8756
Recruitment of NuMA to mitotic centrosomes	0.173913	0.000712	0.065217	0.60949	0.130435	0.069
RHO GTPases Activate Formins	0.152174	0.000735	0.036232	0.96425	0.057971	0.8832
Separation of Sister Chromatids	0.138298	0.000821	0.037234	0.97687	0.06383	0.8478
Intraflagellar transport	0.211538	0.000907	0.038462	0.88016	0.115385	0.2462
Mitotic Metaphase and Anaphase	0.135	0.000956	0.035	0.98625	0.07	0.7607
Mitotic Anaphase	0.130653	0.001897	0.035176	0.98563	0.065327	0.8332
G0 and Early G1	0.259259	0.002282	0.111111	0.28104	0.074074	0.6576
SUMO E3 ligases SUMOylate target proteins	0.132597	0.002286	0.055249	0.80251	0.082873	0.5105
Cleavage of Growing Transcript in the Termination Region	0.179104	0.002475	0.134328	0.03817	0.104478	0.3025
RNA Polymerase II Transcription Termination	0.179104	0.002475	0.134328	0.03817	0.104478	0.3025
Activation of E2F1 target genes at G1/S	0.25	0.002858	0.107143	0.3002	0.142857	0.1897
G1/S-Specific Transcription	0.25	0.002858	0.107143	0.3002	0.142857	0.1897
APC/C-mediated degradation of cell cycle proteins	0.162791	0.002861	0.046512	0.84947	0.093023	0.4011
Regulation of mitotic cell cycle	0.162791	0.002861	0.046512	0.84947	0.093023	0.4011
Peroxisomal lipid metabolism	0.241379	0.00354	0.206897	0.01236	0.137931	0.2069
Transcription of E2F targets under negative control by p107 (RBL1) and p130 (RBL2) in complex with HDAC1	0.3125	0.004083	0.125	0.30067	0.0625	0.7432
Peroxisomal protein import	0.174603	0.004526	0.174603	0.00335	0.126984	0.1375
Glyoxylate metabolism and glycine degradation	0.225806	0.005267	0.129032	0.15945	0.064516	0.7309
Beta-catenin phosphorylation cascade	0.294118	0.00545	0.235294	0.02537	0.235294	0.0441
Condensation of Prometaphase Chromosomes	0.363636	0.005628	NA	NA	0.181818	0.2239
SLBP Dependent Processing of Replication-Dependent Histone Pre-mRNAs	0.363636	0.005628	0.090909	0.54205	0.090909	0.6071
tRNA processing in the nucleus	0.178571	0.005652	0.071429	0.54037	0.089286	0.4835
TP53 Regulates Transcription of Genes Involved in G2 Cell Cycle Arrest	0.277778	0.007111	0.111111	0.35225	0.166667	0.1763
Activation of APC/C and APC/C:Cdc20 mediated degradation of mitotic proteins	0.157895	0.007126	0.052632	0.77382	0.078947	0.5922
Activation of the TFAP2 (AP-2) family of transcription factors	0.333333	0.007971	0.083333	0.57345	0.083333	0.6391
G2/M Checkpoints	0.125	0.00821	0.059524	0.72279	0.059524	0.8865

Transcription of E2F targets under negative control by DREAM complex	0.263158	0.009095	0.157895	0.13715	0.052632	0.801
Phosphorylation of the APC/C	0.263158	0.009095	0.157895	0.13715	0.157895	0.1976
DNA Repair	0.107595	0.010218	0.075949	0.32856	0.094937	0.2118
Regulation of APC/C activators between G1/S and early anaphase	0.15	0.010678	0.05	0.80711	0.075	0.6428
Chk1/Chk2(Cds1) mediated inactivation of Cyclin B:Cdk1 complex	0.307692	0.010874	0.153846	0.22241	0.076923	0.6686
SUMOylation of transcription cofactors	0.177778	0.013094	0.088889	0.37188	0.133333	0.1559
Mitochondrial tRNA aminoacylation	0.238095	0.014141	0.238095	0.01206	0.238095	0.024
Mitochondrial Fatty Acid Beta-Oxidation	0.189189	0.01423	0.108108	0.24593	0.027027	0.957
APC/C:Cdc20 mediated degradation of mitotic proteins	0.146667	0.016621	0.053333	0.76482	0.08	0.579
G2/M DNA damage checkpoint	0.136842	0.016864	0.063158	0.64089	0.063158	0.7959
RHO GTPase Effectors	0.10443	0.016989	0.041139	0.98596	0.03481	0.9998
Protein localization	0.125984	0.01817	0.125984	0.01293	0.086614	0.4609
mRNA decay by 5' to 3' exoribonuclease	0.266667	0.018526	0.066667	0.65534	0.066667	0.7204
mRNA 3'-end processing	0.155172	0.020609	0.137931	0.04292	0.103448	0.3328
TP53 Regulates Transcription of Cell Cycle Genes	0.163265	0.021351	0.081633	0.43506	0.163265	0.0426
Diseases associated with N-glycosylation of proteins	0.235294	0.028845	0.235294	0.02537	0.058824	0.7641
SUMOylation of DNA methylation proteins	0.235294	0.028845	0.117647	0.32659	0.235294	0.0441
Cyclin A/B1/B2 associated events during G2/M transition	0.2	0.029191	0.04	0.83072	0.08	0.6153
mRNA Splicing - Minor Pathway	0.153846	0.029578	0.019231	0.97526	0.076923	0.6207
SLBP independent Processing of Histone Pre-mRNAs	0.3	0.029619	0.1	0.50833	0.1	0.5723
Processing and activation of SUMO	0.3	0.029619	NA	NA	0.1	0.5723
APC:Cdc20 mediated degradation of cell cycle proteins prior to satisfation of the cell cycle checkpoint	0.136986	0.033427	0.054795	0.74599	0.068493	0.7192
SUMOylation of DNA replication proteins	0.159091	0.034503	0.022727	0.95624	0.045455	0.8836
Regulation of TP53 Activity	0.1125	0.035742	0.09375	0.13359	0.11875	0.0609
DNA Replication	0.11811	0.036225	0.070866	0.50814	0.062992	0.8216
Beta-oxidation of very long chain fatty acids	0.272727	0.038616	0.181818	0.1712	0.181818	0.2239
CDC6 association with the ORC:origin complex	0.272727	0.038616	0.090909	0.54205	0.090909	0.6071
Regulation of TP53 Degradation	0.166667	0.039893	0.083333	0.45191	0.083333	0.5704
Synthesis of DNA	0.117647	0.043252	0.067227	0.57455	0.058824	0.8612
Dual incision in TC-NER	0.136364	0.043416	0.106061	0.16414	0.060606	0.7962
Transcriptional Regulation by TP53	0.09589	0.043664	0.093151	0.04104	0.109589	0.0321
Activation of ATR in response to replication stress	0.162162	0.04486	0.162162	0.03812	0.081081	0.5894

Regulation of TP53 Expression and Degradation	0.162162	0.04486	0.081081	0.47009	0.081081	0.5894
RNA Polymerase III Transcription Initiation From Type 3 Promoter	0.178571	0.04534	0.107143	0.3002	0.107143	0.4015
G1/S Transition	0.114504	0.045722	0.061069	0.68279	0.068702	0.7492
Fatty acid metabolism	0.107345	0.047473	0.067797	0.55825	0.079096	0.5853
Kinesins	0.140351	0.047708	0.105263	0.19359	0.087719	0.4995
HDR through Homologous Recombination (HRR) or Single Strand Annealing (SSA)	0.113636	0.048348	0.060606	0.69099	0.106061	0.1856
Post-chaperonin tubulin folding pathway	0.2	0.049614	0.1	0.40244	0.05	0.8172
Metabolism of cofactors	0.2	0.049614	0.1	0.40244	0.05	0.8172
Asparagine N-linked glycosylation	0.072848	0.489383	0.115894	0.0015	0.109272	0.0498
N-glycan trimming in the ER and Calnexin/Calreticulin cycle	0.085714	0.459461	0.228571	0.00209	0.171429	0.0608
VEGFR2 mediated cell proliferation	0.142857	0.185177	0.285714	0.00225	0.095238	0.5191
Membrane Trafficking	0.055732	0.953318	0.095541	0.00501	0.074841	0.7532
Neddylation	0.098291	0.071906	0.115385	0.00526	0.128205	0.0085
Retrograde transport at the Trans-Golgi-Network	0.081633	0.46563	0.183673	0.00545	0.102041	0.3686
Translesion Synthesis by POLH	0.157895	0.149395	0.263158	0.00772	0.105263	0.4654
Rab regulation of trafficking	0.064516	0.666042	0.129032	0.0104	0.072581	0.6887
TBC/RABGAPs	0.130435	0.106304	0.173913	0.01194	0.086957	0.5218
Biosynthesis of the N-glycan precursor (dolichol lipid-linked oligosaccharide, LLO) and transfer to a nascent protein	0.089744	0.320034	0.141026	0.01675	0.115385	0.1818
Vesicle-mediated transport	0.052474	0.981945	0.089955	0.01696	0.073463	0.8001
Acyl chain remodelling of PS	0.086957	0.495233	0.217391	0.0178	NA	NA
Regulation of KIT signaling	0.0625	0.693816	0.25	0.02048	0.1875	0.1361
Activation of PPARGC1A (PGC-1alpha) by phosphorylation	0.2	0.156101	0.3	0.02671	0.3	0.0418
Base-Excision Repair, AP Site Formation	0.1	0.522653	0.3	0.02671	0.1	0.5723
Ketone body metabolism	0.1	0.522653	0.3	0.02671	NA	NA
Calnexin/calreticulin cycle	0.038462	0.854014	0.192308	0.0294	0.153846	0.1568
Glycerophospholipid biosynthesis	0.062016	0.708663	0.116279	0.03033	0.062016	0.8344
TP53 Regulates Metabolic Genes	0.116279	0.084355	0.127907	0.03226	0.104651	0.2645
Antigen processing: Ubiquitination & Proteasome degradation	0.097087	0.0512	0.097087	0.03312	0.116505	0.0179
Phospholipid metabolism	0.075472	0.443116	0.103774	0.03327	0.070755	0.7522
GAB1 signalosome	0.090909	0.556698	0.272727	0.0349	0.090909	0.6071
Acyl chain remodelling of PG	0.157895	0.149395	0.210526	0.03708	NA	NA
Receptor-type tyrosine-protein phosphatases	0.05	0.772306	0.2	0.04392	0.05	0.8172

Base Excision Repair	0.128205	0.141533	0.153846	0.04771	0.076923	0.6257
Resolution of Abasic Sites (AP sites)	0.128205	0.141533	0.153846	0.04771	0.076923	0.6257
DNA Damage Bypass	0.102041	0.268957	0.142857	0.04796	0.102041	0.3686
Translocation of SLC2A4 (GLUT4) to the plasma membrane	0.114286	0.123536	0.128571	0.04852	0.071429	0.6839
Reversible hydration of carbon dioxide	0.083333	0.588317	0.083333	0.57345	0.416667	0.0017
Deactivation of the beta-catenin transactivating complex	0.119048	0.176699	0.095238	0.3241	0.214286	0.0058
Fanconi Anemia Pathway	0.125	0.152896	0.125	0.13581	0.2	0.0138
Resolution of D-loop Structures through Holliday Junction Intermediates	0.121212	0.20578	0.090909	0.39599	0.212121	0.0152
Resolution of D-Loop Structures	0.117647	0.221103	0.088235	0.41484	0.205882	0.0178
Creation of C4 and C2 activators	NA	NA	NA	NA	0.285714	0.0225
CD28 dependent PI3K/Akt signaling	0.090909	0.471552	0.136364	0.18817	0.227273	0.029
Urea cycle	NA	NA	0.1	0.50833	0.3	0.0418
HDR through Homologous Recombination (HRR)	0.089552	0.342698	0.104478	0.17325	0.149254	0.0434

Table S3B. Enriched Reactome pathways from down-regulated genes at 2.5h pi. Significant Reactome pathways using down-regulated DEGs from STM, T3SS-1^{mut} or T3SS-2^{mut}-infected HIOs at 2.5h.
