# Supplementary material for: Salmonella enterica Serovar Typhimurium SPI-1 and SPI-2 Shape the Global Transcriptional Landscape in a Human Intestinal Organoid Model System
Source: mBio. 2021 May 18;12(3):e00399-21. doi: 10.1128/mBio.00399-21 (PMC8262845; doi:10.1128/mBio.00399-21)
Supplement: TABLE S4 [file mbio.00399-21-st004.pdf]

Table S4A: Reactome Pathways Upregulated 8h pi

Description	STM		T3SS-1^mut		T3SS-2^mut	
	gene ratio	p-value	gene ratio	p-value	gene ratio	p-value
Cytokine Signaling in Immune system	0.2688953	2.58E-28	0.206395	9.42E-25	0.22093	2.95E-23
Signaling by Interleukins	0.2835498	2.67E-22	0.218615	1.60E-19	0.238095	1.74E-19
Interleukin-4 and Interleukin-13 signaling	0.3888889	7.97E-13	0.314815	7.30E-12	0.277778	8.97E-08
Extracellular matrix organization	0.2392027	4.99E-09	0.192691	2.27E-09	0.099668	0.49371
Toll Like Receptor 3 (TLR3) Cascade	0.3333333	2.17E-08	0.20202	0.00022	0.252525	6.95E-06
MyD88-independent TLR4 cascade	0.33	2.87E-08	0.2	0.00025	0.25	8.41E-06
TRIF(TICAM1)-mediated TLR4 signaling	0.33	2.87E-08	0.2	0.00025	0.25	8.41E-06
Toll Like Receptor 4 (TLR4) Cascade	0.3	3.24E-08	0.207692	1.11E-05	0.230769	6.35E-06
Rab regulation of trafficking	0.2983871	8.49E-08	0.129032	0.0622	0.217742	5.45E-05
Interleukin-10 signaling	0.4255319	1.41E-07	0.489362	4.44E-13	0.382979	1.78E-07
Vesicle-mediated transport	0.1874063	1.62E-07	0.110945	0.01071	0.152924	2.58E-06
Interferon gamma signaling	0.326087	1.64E-07	0.217391	7.62E-05	0.23913	6.01E-05
Membrane Trafficking	0.1894904	1.78E-07	0.111465	0.01163	0.16242	1.41E-07
Signaling by Receptor Tyrosine Kinases	0.2021978	2.52E-07	0.18022	3.38E-11	0.156044	4.74E-05
Toll Like Receptor 7/8 (TLR7/8) Cascade	0.3191489	2.80E-07	0.180851	0.0023	0.234043	8.49E-05
MyD88 dependent cascade initiated on endosome	0.3191489	2.80E-07	0.180851	0.0023	0.234043	8.49E-05
Toll Like Receptor 10 (TLR10) Cascade	0.3294118	3.33E-07	0.2	0.00072	0.258824	1.60E-05
Toll Like Receptor 5 (TLR5) Cascade	0.3294118	3.33E-07	0.2	0.00072	0.258824	1.60E-05
MyD88 cascade initiated on plasma membrane	0.3294118	3.33E-07	0.2	0.00072	0.258824	1.60E-05
MyD88:MAL(TIRAP) cascade initiated on plasma membrane	0.3157895	3.62E-07	0.189474	0.001	0.242105	3.33E-05
Toll Like Receptor TLR6:TLR2 Cascade	0.3157895	3.62E-07	0.189474	0.001	0.242105	3.33E-05
MAPK family signaling cascades	0.221843	5.50E-07	0.139932	0.00107	0.177474	1.60E-05
Toll-like Receptor Cascades	0.2645161	6.55E-07	0.193548	1.59E-05	0.219355	5.20E-06
Interleukin-1 family signaling	0.2733813	6.86E-07	0.223022	4.97E-07	0.294964	5.11E-11
TRAF6 mediated induction of NFkB and MAP kinases upon TLR7/8 or 9 activation	0.311828	7.61E-07	0.182796	0.00204	0.236559	7.15E-05
Toll Like Receptor 9 (TLR9) Cascade	0.3061224	7.66E-07	0.173469	0.00362	0.22449	0.00016
Toll Like Receptor TLR1:TLR2 Cascade	0.3061224	7.66E-07	0.183673	0.00145	0.234694	5.64E-05
Toll Like Receptor 2 (TLR2) Cascade	0.3061224	7.66E-07	0.183673	0.00145	0.234694	5.64E-05
Clathrin-mediated endocytosis	0.2642857	2.33E-06	0.142857	0.01528	0.171429	0.00469
Interleukin-1 signaling	0.2912621	2.44E-06	0.23301	4.26E-06	0.300971	6.81E-09
DDX58/IFIH1-mediated induction of interferon-alpha/beta	0.3205128	2.49E-06	0.230769	7.59E-05	0.25641	4.46E-05

Death Receptor Signalling	0.2624113	2.80E-06	0.177305	0.00034	0.205674	8.77E-05
Interleukin-6 signaling	0.7272727	5.14E-06	0.727273	3.58E-07	0.727273	1.06E-06
MAPK1/MAPK3 signaling	0.2165354	8.75E-06	0.145669	0.00088	0.181102	2.90E-05
TBC/RABGAPs	0.3695652	1.22E-05	0.130435	0.19558	0.217391	0.01223
RAB GEFs exchange GTP for GDP on RABs	0.2888889	1.33E-05	0.133333	0.08015	0.233333	0.00013
TNFR2 non-canonical NF-kB pathway	0.2745098	1.80E-05	0.284314	3.70E-09	0.264706	1.12E-06
Syndecan interactions	0.4444444	2.73E-05	0.37037	4.29E-05	0.148148	0.27058
Interleukin-12 family signaling	0.3275862	2.79E-05	0.344828	2.77E-08	0.310345	6.17E-06
MAP kinase activation	0.3125	3.75E-05	0.171875	0.01859	0.234375	0.00108
RAF/MAP kinase cascade	0.2096774	3.86E-05	0.137097	0.00384	0.173387	0.00015
Adaptive Immune System	0.1679894	4.20E-05	0.145503	9.07E-09	0.154762	2.33E-07
Glycosaminoglycan metabolism	0.25	4.89E-05	0.153226	0.00872	0.096774	0.56557
RIP-mediated NFkB activation via ZBP1	0.5294118	5.13E-05	0.294118	0.01164	0.352941	0.00422
TAK1 activates NFkB by phosphorylation and activation of IKKs complex	0.3939394	5.94E-05	0.212121	0.0193	0.272727	0.00359
ZBP1(DAI) mediated induction of type I IFNs	0.4761905	6.27E-05	0.238095	0.02881	0.333333	0.00291
Interleukin-17 signaling	0.2916667	7.55E-05	0.166667	0.01805	0.222222	0.00138
Diseases of signal transduction	0.1878307	7.76E-05	0.142857	0.00011	0.15873	0.00011
Transcriptional regulation of white adipocyte differentiation	0.2738095	0.0001	0.154762	0.02516	0.142857	0.11824
Signaling by TGF-beta family members	0.254902	0.00014	0.205882	0.00012	0.186275	0.00449
Regulation of lipid metabolism by Peroxisome proliferator-activated receptor alpha (PPARalpha)	0.2436975	0.00014	0.092437	0.43773	0.151261	0.04175
Interferon Signaling	0.213198	0.00014	0.177665	2.27E-05	0.192893	3.44E-05
Circadian Clock	0.2857143	0.00015	0.142857	0.07245	0.214286	0.00281
Elastic fibre formation	0.3333333	0.00015	0.266667	0.00028	0.155556	0.14758
SUMOylation of transcription cofactors	0.3333333	0.00015	0.066667	0.75132	0.177778	0.06902
Integrin alphaIIb beta3 signaling	0.4074074	0.00015	0.259259	0.00627	0.444444	3.23E-06
Integrin signaling	0.4074074	0.00015	0.259259	0.00627	0.444444	3.23E-06
RAF-independent MAPK1/3 activation	0.4347826	0.00016	0.26087	0.01085	0.391304	0.00019
Nucleotide-binding domain, leucine rich repeat containing receptor (NLR) signaling pathways	0.3090909	0.00016	0.2	0.00602	0.254545	0.00066
NOD1/2 Signaling Pathway	0.3611111	0.00017	0.25	0.00264	0.277778	0.00187
p75NTR signals via NF-kB	0.5	0.00023	0.25	0.04189	0.3125	0.0157
L1CAM interactions	0.2393162	0.00025	0.205128	4.16E-05	0.205128	0.00036

Interleukin-6 family signaling	0.4166667	0.00025	0.458333	1.46E-06	0.416667	4.31E-05
TRAF6 mediated NF-kB activation	0.4166667	0.00025	0.291667	0.00306	0.291667	0.00667
Cargo recognition for clathrin-mediated endocytosis	0.25	0.00026	0.12	0.14385	0.15	0.06302
Signaling by NOTCH1	0.2739726	0.00028	0.164384	0.01998	0.178082	0.02419
Ovarian tumor domain proteases	0.3421053	0.00031	0.289474	0.00023	0.315789	0.00018
Platelet activation, signaling and aggregation	0.1946565	0.00032	0.164122	2.13E-05	0.148855	0.00536
TNF signaling	0.3255814	0.00034	0.209302	0.00923	0.209302	0.02164
Interleukin-12 signaling	0.3125	0.00034	0.270833	0.00013	0.270833	0.00053
Hemostasis	0.166129	0.00035	0.120968	0.00113	0.133871	0.00189
Interferon alpha/beta signaling	0.2753623	0.00037	0.26087	1.28E-05	0.231884	0.00085
Negative regulators of DDX58/IFIH1 signaling	0.3529412	0.00038	0.264706	0.00172	0.323529	0.00026
Axon guidance	0.168784	0.00039	0.137931	1.55E-05	0.141561	0.00051
Signal attenuation	0.6	0.00041	0.3	0.04722	0.3	0.06697
Non-integrin membrane-ECM interactions	0.2881356	0.00042	0.305085	1.09E-06	0.118644	0.35819
PIP3 activates AKT signaling	0.1923077	0.00049	0.119231	0.03556	0.176923	5.29E-05
Signaling by NTRKs	0.2424242	0.00055	0.161616	0.00935	0.171717	0.01549
Regulation of TP53 Activity through Association with Co-factors	0.5	0.00058	0.142857	0.33868	0.285714	0.0415
Regulation of IFNG signaling	0.5	0.00058	0.5	6.49E-05	0.642857	1.04E-06
PPARA activates gene expression	0.2307692	0.00059	0.094017	0.41528	0.153846	0.0361
RORA activates gene expression	0.4444444	0.00062	0.277778	0.01501	0.222222	0.09295
Cell-extracellular matrix interactions	0.4444444	0.00062	0.111111	0.4624	0.388889	0.00104
TNF receptor superfamily (TNFSF) members mediating non-canonical NF-kB pathway	0.4444444	0.00062	0.333333	0.00288	0.277778	0.02612
Assembly of collagen fibrils and other multimeric structures	0.2786885	0.00064	0.278689	8.55E-06	0.065574	0.86203
Signaling by NTRK1 (TRKA)	0.2564103	0.00072	0.192308	0.00219	0.179487	0.01856
Interleukin-27 signaling	0.5454545	0.00082	0.818182	1.09E-08	0.545455	0.00026
Integrin cell surface interactions	0.2470588	0.00091	0.235294	2.28E-05	0.176471	0.01754
Smooth Muscle Contraction	0.3243243	0.00092	0.081081	0.62281	0.297297	0.0006
NOTCH1 Intracellular Domain Regulates Transcription	0.2978723	0.00092	0.170213	0.04361	0.148936	0.1735
Cytosolic sensors of pathogen-associated DNA	0.2698413	0.00096	0.142857	0.08556	0.190476	0.01815
YAP1- and WWTR1 (TAZ)-stimulated gene expression	0.4666667	0.00097	0.133333	0.37068	0.333333	0.01172
IGF1R signaling cascade	0.2830189	0.0011	0.132075	0.16287	0.169811	0.07105
Regulation of TNFR1 signaling	0.3333333	0.00116	0.212121	0.0193	0.242424	0.01256
Chemokine receptors bind chemokines	0.2916667	0.00116	0.291667	3.02E-05	0.270833	0.00053

Molecules associated with elastic fibres	0.3157895	0.0012	0.236842	0.00392	0.157895	0.16407
Diseases of Immune System	0.375	0.00126	0.25	0.01345	0.166667	0.20465
Diseases associated with the TLR signaling cascade	0.375	0.00126	0.25	0.01345	0.166667	0.20465
Growth hormone receptor signaling	0.375	0.00126	0.291667	0.00306	0.375	0.00027
Signaling by Type 1 Insulin-like Growth Factor 1 Receptor (IGF1R)	0.2777778	0.00136	0.12963	0.17432	0.166667	0.0782
Semaphorin interactions	0.2615385	0.0014	0.138462	0.09962	0.123077	0.30463
TNFs bind their physiological receptors	0.3448276	0.00144	0.37931	1.36E-05	0.206897	0.05892
COPI-independent Golgi-to-ER retrograde traffic	0.2857143	0.00145	0.265306	0.00017	0.326531	9.73E-06
NOTCH2 intracellular domain regulates transcription	0.5	0.00147	NA	NA	0.166667	0.33255
Interleukin-35 Signalling	0.5	0.00147	0.666667	9.93E-07	0.5	0.00048
TICAM1-dependent activation of IRF3/IRF7	0.5	0.00147	0.25	0.07623	0.333333	0.02403
Intracellular signaling by second messengers	0.1808874	0.00148	0.122867	0.01648	0.163823	0.00025
Transcriptional activity of SMAD2/SMAD3:SMAD4 heterotrimer	0.2954545	0.00151	0.204545	0.01075	0.204545	0.02491
Negative regulation of the PI3K/AKT network	0.2264151	0.00153	0.141509	0.03521	0.141509	0.09392
Platelet Aggregation (Plug Formation)	0.3076923	0.00154	0.230769	0.00472	0.307692	0.00024
Gene and protein expression by JAK-STAT signaling after Interleukin-12 stimulation	0.3076923	0.00154	0.25641	0.00126	0.282051	0.00098
Regulation of cholesterol biosynthesis by SREBP (SREBF)	0.2727273	0.00167	0.109091	0.32803	0.218182	0.00616
Transcriptional Regulation by MECP2	0.2727273	0.00167	0.072727	0.7022	0.145455	0.16745
CLEC7A (Dectin-1) signaling	0.2277228	0.00175	0.207921	0.0001	0.237624	3.15E-05
Signaling by VEGF	0.2242991	0.00175	0.196262	0.00024	0.196262	0.00149
Nuclear Events (kinase and transcription factor activation)	0.36	0.00176	0.16	0.15995	0.24	0.03066
Recycling pathway of L1	0.2888889	0.0019	0.244444	0.00112	0.266667	0.00101
RHO GTPases activate IQGAPs	0.3333333	0.00193	0.266667	0.00293	0.4	1.21E-05
Regulation of MECP2 expression and activity	0.3333333	0.00193	0.066667	0.73912	0.133333	0.33951
Collagen formation	0.2333333	0.00198	0.244444	4.62E-06	0.044444	0.98116
p75 NTR receptor-mediated signalling	0.2268041	0.0023	0.14433	0.03541	0.185567	0.00583
NF-kB is activated and signals survival	0.4615385	0.00245	0.307692	0.02019	0.307692	0.03207
Dissolution of Fibrin Clot	0.4615385	0.00245	0.384615	0.00323	0.307692	0.03207
Erythropoietin activates RAS	0.4615385	0.00245	0.230769	0.09304	0.384615	0.00593
Metabolism of lipids	0.1546811	0.00247	0.097693	0.12147	0.12076	0.02119
HS-GAG biosynthesis	0.3225806	0.00254	0.193548	0.04429	0.16129	0.18295
RHO GTPases Activate WASPs and WAVEs	0.3055556	0.00256	0.194444	0.03021	0.25	0.00671

IRS-related events triggered by IGF1R	0.2692308	0.00268	0.115385	0.28199	0.153846	0.13308
Signaling by NOTCH1 PEST Domain Mutants in Cancer	0.2586207	0.00294	0.172414	0.02372	0.12069	0.34165
Signaling by NOTCH1 in Cancer	0.2586207	0.00294	0.172414	0.02372	0.12069	0.34165
Constitutive Signaling by NOTCH1 PEST Domain Mutants	0.2586207	0.00294	0.172414	0.02372	0.12069	0.34165
Signaling by NOTCH1 HD+PEST Domain Mutants in Cancer	0.2586207	0.00294	0.172414	0.02372	0.12069	0.34165
Constitutive Signaling by NOTCH1 HD+PEST Domain Mutants	0.2586207	0.00294	0.172414	0.02372	0.12069	0.34165
VEGFA-VEGFR2 Pathway	0.2222222	0.00302	0.212121	7.43E-05	0.20202	0.00132
PI5P, PP2A and IER3 Regulate PI3K/AKT Signaling	0.2222222	0.00302	0.131313	0.07771	0.121212	0.26334
Activation of gene expression by SREBF (SREBP)	0.2857143	0.0031	0.119048	0.28685	0.214286	0.01868
BMAL1:CLOCK,NPAS2 activates circadian gene expression	0.3333333	0.00322	0.259259	0.00627	0.185185	0.11909
Interleukin receptor SHC signaling	0.3333333	0.00322	0.296296	0.0014	0.259259	0.01321
Signaling by RAS mutants	0.2641509	0.00325	0.150943	0.07886	0.188679	0.03141
SMAD2/SMAD3:SMAD4 heterotrimer regulates transcription	0.3125	0.0033	0.21875	0.01638	0.1875	0.08775
FCERI mediated NF-kB activation	0.2317073	0.00345	0.207317	0.00047	0.268293	8.59E-06
Interleukin-3, Interleukin-5 and GM-CSF signaling	0.2708333	0.00355	0.229167	0.00197	0.208333	0.01642
Rho GTPase cycle	0.2028986	0.00368	0.123188	0.07905	0.130435	0.12903
MAPK3 (ERK1) activation	0.5	0.00377	0.5	0.00078	0.5	0.00149
adhesion	0.4285714	0.00385	0.142857	0.33868	0.214286	0.15199
RUNX3 regulates NOTCH signaling	0.4285714	0.00385	0.071429	0.71353	0.142857	0.40596
Interleukin-15 signaling	0.4285714	0.00385	0.285714	0.02639	0.357143	0.00849
TRAF3-dependent IRF activation pathway	0.4285714	0.00385	0.142857	0.33868	0.214286	0.15199
Insulin receptor signalling cascade	0.2592593	0.00391	0.148148	0.08601	0.166667	0.0782
Signaling by PTK6	0.2592593	0.00391	0.166667	0.03773	0.277778	0.00015
Signaling by Non-Receptor Tyrosine Kinases	0.2592593	0.00391	0.166667	0.03773	0.277778	0.00015
Signaling by BMP	0.3214286	0.00425	0.285714	0.00181	0.321429	0.00101
G-protein beta:gamma signalling	0.3214286	0.00425	0.142857	0.2131	0.214286	0.05077
Transcriptional regulation by RUNX3	0.21875	0.00451	0.1875	0.00113	0.270833	1.10E-06
Degradation of the extracellular matrix	0.2	0.00454	0.207143	5.57E-06	0.071429	0.89183
Neutrophil degranulation	0.1607516	0.00458	0.148225	2.39E-06	0.150313	0.00014
MHC class II antigen presentation	0.2066116	0.00458	0.165289	0.00303	0.198347	0.0006
Heparan sulfate/heparin (HS-GAG) metabolism	0.2545455	0.00468	0.127273	0.1861	0.090909	0.63871
Activation of NMDA receptors and postsynaptic events	0.2727273	0.00471	0.159091	0.07725	0.159091	0.13536
Interleukin-2 family signaling	0.2727273	0.00471	0.25	0.00092	0.227273	0.00891
Fc epsilon receptor (FCERI) signaling	0.2014925	0.00474	0.186567	0.00015	0.231343	4.23E-06

Signaling by TGF-beta Receptor Complex	0.2328767	0.00522	0.178082	0.00823	0.178082	0.02419
Signaling by MET	0.2278481	0.00526	0.278481	4.22E-07	0.189873	0.00913
activated TAK1 mediates p38 MAPK activation	0.3333333	0.00541	0.208333	0.04873	0.208333	0.07966
Signaling by Erythropoietin	0.3333333	0.00541	0.208333	0.04873	0.25	0.02534
Transcriptional activation of mitochondrial biogenesis	0.25	0.00556	0.107143	0.34361	0.178571	0.04401
C-type lectin receptors (CLRs)	0.1971831	0.00557	0.169014	0.00089	0.197183	0.00024
Regulated proteolysis of p75NTR	0.4545455	0.00623	0.454545	0.00134	0.363636	0.01734
Dermatan sulfate biosynthesis	0.4545455	0.00623	0.272727	0.06092	0.181818	0.29481
Signaling by Leptin	0.4545455	0.00623	0.272727	0.06092	0.272727	0.08558
SUMOylation of immune response proteins	0.4545455	0.00623	0.272727	0.06092	0.545455	0.00026
RHO GTPases activate KTN1	0.4545455	0.00623	0.454545	0.00134	0.636364	1.99E-05
Erythropoietin activates Phosphoinositide-3-kinase (PI3K)	0.4545455	0.00623	0.363636	0.01069	0.272727	0.08558
Signaling by moderate kinase activity BRAF mutants	0.275	0.00626	0.175	0.05029	0.225	0.01367
Paradoxical activation of RAF signaling by kinase inactive BRAF	0.275	0.00626	0.175	0.05029	0.225	0.01367
GPVI-mediated activation cascade	0.2857143	0.00672	0.314286	0.0001	0.257143	0.0055
NOTCH4 Intracellular Domain Regulates Transcription	0.35	0.0068	0.1	0.51902	0.1	0.59791
Signaling by the B Cell Receptor (BCR)	0.2053571	0.00688	0.232143	1.85E-06	0.232143	2.32E-05
Laminin interactions	0.3	0.00705	0.266667	0.00293	0.133333	0.33951
TNFR1-induced NFkappaB signaling pathway	0.3	0.00705	0.166667	0.10774	0.166667	0.16593
G beta:gamma signalling through PI3Kgamma	0.32	0.00714	0.16	0.15995	0.24	0.03066
TCR signaling	0.2016807	0.00738	0.168067	0.00248	0.201681	0.00047
Diseases associated with glycosaminoglycan metabolism	0.2682927	0.00764	0.146341	0.13294	0.073171	0.78103
MET promotes cell motility	0.2682927	0.00764	0.365854	6.33E-07	0.121951	0.37603
Collagen degradation	0.234375	0.00791	0.234375	0.00025	0.0625	0.88576
Oncogenic MAPK signaling	0.2236842	0.00797	0.131579	0.11114	0.157895	0.06582
MAPK targets/ Nuclear events mediated by MAP kinases	0.2903226	0.0089	0.129032	0.2703	0.258065	0.0085
Arachidonic acid metabolism	0.2372881	0.00904	0.186441	0.01031	0.118644	0.35819
Synthesis of Leukotrienes (LT) and Eoxins (EX)	0.3333333	0.00915	0.190476	0.09849	0.142857	0.34057
Signal transduction by L1	0.3333333	0.00915	0.190476	0.09849	0.238095	0.04858
SHC-mediated cascade:FGFR1	0.3333333	0.00915	0.190476	0.09849	0.190476	0.14451
TP53 Regulates Transcription of Death Receptors and Ligands	0.4166667	0.00963	0.166667	0.27339	0.333333	0.02403
Interleukin-2 signaling	0.4166667	0.00963	0.333333	0.01497	0.166667	0.33255
Signaling by Insulin receptor	0.2179487	0.01038	0.153846	0.032	0.166667	0.03935
PI3K/AKT Signaling in Cancer	0.2061856	0.01076	0.14433	0.03541	0.123711	0.24107

CREB phosphorylation through the activation of Ras	0.28125	0.01108	0.09375	0.52234	0.15625	0.20058
Neurotransmitter receptors and postsynaptic signal transmission	0.1858974	0.01113	0.108974	0.17719	0.102564	0.46574
IL-6-type cytokine receptor ligand interactions	0.3529412	0.01155	0.411765	0.00029	0.294118	0.02048
Metabolism of steroids	0.1866667	0.0118	0.106667	0.20944	0.146667	0.03593
Downstream TCR signaling	0.2040816	0.01203	0.173469	0.00362	0.214286	0.00045
ERK/MAPK targets	0.3181818	0.01205	0.136364	0.28863	0.181818	0.16374
Regulation of actin dynamics for phagocytic cup formation	0.2295082	0.01217	0.147541	0.07276	0.196721	0.0142
EPH-Ephrin signaling	0.2065217	0.0125	0.195652	0.00067	0.141304	0.11413
PI3K Cascade	0.25	0.01325	0.113636	0.32124	0.136364	0.26006
Signaling by NOTCH2	0.2727273	0.01365	0.060606	0.7858	0.121212	0.40891
Chondroitin sulfate/dermatan sulfate metabolism	0.24	0.01376	0.14	0.1307	0.1	0.55201
p75NTR recruits signalling complexes	0.3846154	0.01412	0.153846	0.30618	0.230769	0.12831
Loss of function of MECP2 in Rett syndrome	0.3846154	0.01412	0.153846	0.30618	0.153846	0.36968
Pervasive developmental disorders	0.3846154	0.01412	0.153846	0.30618	0.153846	0.36968
JNK (c-Jun kinases) phosphorylation and activation mediated by activated human TAK1	0.3043478	0.01557	0.130435	0.31278	0.217391	0.06834
FRS-mediated FGFR1 signaling	0.3043478	0.01557	0.130435	0.31278	0.130435	0.3962
Gastrin-CREB signalling pathway via PKC and MAPK	0.3333333	0.01559	0.111111	0.4624	0.277778	0.02612
Activation of IRF3/IRF7 mediated by TBK1/IKK epsilon	0.3333333	0.01559	0.111111	0.4624	0.222222	0.09295
EPH-ephrin mediated repulsion of cells	0.2352941	0.01608	0.196078	0.00995	0.078431	0.75097
Signaling by NOTCH4	0.2073171	0.01691	0.195122	0.00135	0.231707	0.00029
Post NMDA receptor activation events	0.25	0.01779	0.125	0.25322	0.125	0.3558
Costimulation by the CD28 family	0.2142857	0.018	0.157143	0.03406	0.157143	0.07813
Transport to the Golgi and subsequent modification	0.1748634	0.01885	0.098361	0.29893	0.147541	0.02041
Downstream signaling events of B Cell Receptor (BCR)	0.2048193	0.01895	0.216867	0.00018	0.253012	3.60E-05
Tryptophan catabolism	0.3571429	0.01983	0.357143	0.00467	0.428571	0.00132
PTK6 Regulates RHO GTPases, RAS GTPase and MAP kinases	0.3571429	0.01983	0.285714	0.02639	0.5	0.00016
RET signaling	0.2439024	0.02107	0.146341	0.13294	0.146341	0.21012
PI Metabolism	0.202381	0.02118	0.107143	0.28784	0.190476	0.00701
HSP90 chaperone cycle for steroid hormone receptors (SHR)	0.2264151	0.02158	0.207547	0.00449	0.264151	0.00044
Activation of BH3-only proteins	0.2666667	0.02241	0.266667	0.00293	0.366667	7.28E-05
Regulation of TP53 Activity through Acetylation	0.2666667	0.02241	0.033333	0.9315	0.133333	0.33951
Hedgehog 'off' state	0.1891892	0.02325	0.162162	0.00585	0.243243	6.46E-06

Diseases of glycosylation	0.1811594	0.0236	0.123188	0.07905	0.07971	0.80769
Signaling by high-kinase activity BRAF mutants	0.25	0.02398	0.138889	0.18951	0.194444	0.05758
Interleukin-21 signaling	0.4	0.02423	0.4	0.00729	0.3	0.06697
IRAK2 mediated activation of TAK1 complex	0.4	0.02423	0.2	0.20787	0.2	0.25677
ER to Golgi Anterograde Transport	0.1776316	0.02431	0.098684	0.31677	0.164474	0.00682
Signaling by BRAF and RAF fusions	0.2166667	0.02436	0.133333	0.13648	0.166667	0.06559
Sema4D in semaphorin signaling	0.28	0.02465	0.16	0.15995	0.24	0.03066
Interleukin-20 family signaling	0.28	0.02465	0.28	0.00395	0.2	0.09191
NOTCH3 Intracellular Domain Regulates Transcription	0.28	0.02465	0.12	0.36111	0.12	0.45048
Antigen Presentation: Folding, assembly and peptide loading of class I MHC	0.28	0.02465	0.16	0.15995	0.28	0.0085
G alpha (12/13) signalling events	0.2025316	0.02471	0.088608	0.51705	0.113924	0.37114
IRS-mediated signalling	0.2291667	0.02498	0.104167	0.39101	0.145833	0.18714
Signalling to RAS	0.3	0.0264	0.2	0.08521	0.2	0.12624
TICAM1, RIP1-mediated IKK complex recruitment	0.3	0.0264	0.2	0.08521	0.2	0.12624
Signaling by Hippo	0.3	0.0264	0.1	0.51902	0.3	0.01023
Receptor-type tyrosine-protein phosphatases	0.3	0.0264	NA	NA	0.05	0.87361
Unblocking of NMDA receptors, glutamate binding and activation	0.3	0.0264	0.15	0.2409	0.15	0.31256
TP53 Regulates Transcription of Genes Involved in Cytochrome C Release	0.3	0.0264	0.2	0.08521	0.35	0.00212
Activation of NF-kappaB in B cells	0.2089552	0.02677	0.208955	0.00133	0.268657	5.47E-05
Synthesis of bile acids and bile salts via 27-hydroxycholesterol	0.3333333	0.02686	0.333333	0.00652	0.066667	0.78794
GRB2:SOS provides linkage to MAPK signaling for Integrins	0.3333333	0.02686	0.2	0.13062	0.4	0.00203
p130Cas linkage to MAPK signaling for integrins	0.3333333	0.02686	0.266667	0.03362	0.466667	0.00027
MECP2 regulates neuronal receptors and channels	0.3333333	0.02686	0.066667	0.73802	0.2	0.17691
Trafficking of AMPA receptors	0.2580645	0.02711	0.16129	0.11994	0.16129	0.18295
Glutamate binding, activation of AMPA receptors and synaptic plasticity	0.2580645	0.02711	0.16129	0.11994	0.16129	0.18295
Uptake and actions of bacterial toxins	0.2580645	0.02711	0.096774	0.50055	0.129032	0.3627
Downstream signaling of activated FGFR1	0.2580645	0.02711	0.225806	0.01378	0.193548	0.07741
Defective B3GALTL causes Peters-plus syndrome (PpS)	0.2432432	0.02841	0.216216	0.01134	0.108108	0.49867
TP53 Regulates Transcription of Cell Cycle Genes	0.2244898	0.02882	0.061224	0.80104	0.142857	0.20121
Signaling by SCF-KIT	0.2325581	0.02889	0.232558	0.00278	0.209302	0.02164
Signaling by EGFR	0.2325581	0.02889	0.186047	0.02711	0.209302	0.02164

Other interleukin signaling	0.2692308	0.03033	0.230769	0.01989	0.192308	0.10507
Post-translational protein phosphorylation	0.1851852	0.03239	0.194444	0.00027	0.064815	0.91584
Thrombin signalling through proteinase activated receptors (PARs)	0.25	0.03246	0.125	0.28995	0.1875	0.08775
RUNX2 regulates bone development	0.25	0.03246	0.21875	0.01638	0.0625	0.8361
Nuclear Receptor transcription pathway	0.22	0.03307	0.12	0.25215	0.06	0.88099
Presynaptic function of Kainate receptors	0.2857143	0.0333	0.047619	0.84677	0.142857	0.34057
CD209 (DC-SIGN) signaling	0.2857143	0.0333	0.142857	0.26464	0.142857	0.34057
PI-3K cascade:FGFR1	0.2857143	0.0333	0.190476	0.09849	0.142857	0.34057
TP53 regulates transcription of additional cell cycle genes whose exact role in the p53 pathway remain uncertain	0.2857143	0.0333	0.142857	0.26464	0.238095	0.04858
Negative regulation of MET activity	0.2857143	0.0333	0.238095	0.02881	0.238095	0.04858
Interleukin-37 signaling	0.2857143	0.0333	0.285714	0.00676	0.380952	0.00053
O-glycosylation of TSR domain-containing proteins	0.2368421	0.03338	0.210526	0.01332	0.105263	0.52021
Intrinsic Pathway for Apoptosis	0.2272727	0.0335	0.204545	0.01075	0.340909	1.02E-05
Plasma lipoprotein assembly, remodeling, and clearance	0.2028986	0.03373	0.144928	0.06697	0.086957	0.68267
Regulation of gene expression by Hypoxia-inducible Factor	0.3636364	0.03452	0.090909	0.62547	0.181818	0.29481
Signaling by activated point mutants of FGFR1	0.3636364	0.03452	0.181818	0.24053	0.090909	0.67925
Type I hemidesmosome assembly	0.3636364	0.03452	0.363636	0.01069	0.181818	0.29481
MET activates RAP1 and RAC1	0.3636364	0.03452	0.363636	0.01069	0.363636	0.01734
Activated NTRK2 signals through FRS2 and FRS3	0.3636364	0.03452	NA	NA	0.272727	0.08558
Regulation of KIT signaling	0.3125	0.03529	0.1875	0.1511	0.1875	0.20284
FGFR1 ligand binding and activation	0.3125	0.03529	0.1875	0.1511	0.0625	0.80879
Microtubule-dependent trafficking of connexons from Golgi to the plasma membrane	0.3125	0.03529	0.25	0.04189	0.5	5.26E-05
Regulation of gene expression in late stage (branching morphogenesis) pancreatic bud precursor cells	0.3125	0.03529	NA	NA	0.0625	0.80879
Phospholipase C-mediated cascade: FGFR1	0.3125	0.03529	0.1875	0.1511	0.0625	0.80879
MAPK6/MAPK4 signaling	0.1910112	0.03544	0.157303	0.01811	0.224719	0.00031
XBP1(S) activates chaperone genes	0.2105263	0.03658	0.052632	0.87569	0.157895	0.10219
Endogenous sterols	0.2592593	0.03682	0.222222	0.02377	0.037037	0.93878
Intra-Golgi and retrograde Golgi-to-ER traffic	0.165	0.03696	0.13	0.02004	0.14	0.03422
Translocation of SLC2A4 (GLUT4) to the plasma membrane	0.2	0.03767	0.142857	0.07245	0.3	1.96E-06
CD28 co-stimulation	0.2424242	0.03847	0.242424	0.00552	0.272727	0.00359

Negative regulation of FGFR1 signaling	0.2424242	0.03847	0.090909	0.54359	0.090909	0.64182
Formation of Fibrin Clot (Clotting Cascade)	0.2307692	0.03892	0.205128	0.01553	0.179487	0.08241
RAB geranylgeranylation	0.203125	0.03951	0.140625	0.09243	0.171875	0.04562
Golgi-to-ER retrograde transport	0.1755725	0.04003	0.145038	0.0153	0.183206	0.00192
Beta-catenin independent WNT signaling	0.1724138	0.04045	0.144828	0.01135	0.165517	0.00733
Signaling by PDGF	0.2068966	0.04126	0.241379	0.00028	0.275862	9.95E-05
Constitutive Signaling by Aberrant PI3K in Cancer	0.1971831	0.04192	0.15493	0.03732	0.098592	0.55374
Cell junction organization	0.1868132	0.04276	0.153846	0.02165	0.153846	0.05957
Regulation of Insulin-like Growth Factor (IGF) transport and uptake by Insulin-like Growth Factor Binding Proteins (IGFBPs)	0.176	0.04287	0.168	0.00197	0.056	0.96811
G alpha (q) signalling events	0.1619048	0.04385	0.114286	0.08617	0.109524	0.32064
MAP2K and MAPK activation	0.225	0.04505	0.125	0.25322	0.175	0.09186
Transport of connexons to the plasma membrane	0.2941176	0.04518	0.294118	0.01164	0.470588	9.08E-05
Regulation of RUNX1 Expression and Activity	0.2941176	0.04518	0.058824	0.7809	NA	NA
TICAM1,TRAF6-dependent induction of TAK1 complex	0.2941176	0.04518	0.176471	0.17251	0.117647	0.50801
IRAK2 mediated activation of TAK1 complex upon TLR7/8 or 9 stimulation	0.2941176	0.04518	0.176471	0.17251	0.117647	0.50801
IRE1alpha activates chaperones	0.2033898	0.04634	0.050847	0.88997	0.152542	0.12027
PTEN Regulation	0.1714286	0.04659	0.107143	0.21393	0.214286	2.96E-05
FGFR1c ligand binding and activation	0.3333333	0.04696	0.166667	0.27339	0.083333	0.71077
Caspase-mediated cleavage of cytoskeletal proteins	0.3333333	0.04696	NA	NA	0.166667	0.33255
COPI-mediated anterograde transport	0.1818182	0.04782	0.141414	0.04115	0.181818	0.00724
Fcgamma receptor (FCGR) dependent phagocytosis	0.1860465	0.04992	0.162791	0.01364	0.186047	0.00881
Response to elevated platelet cytosolic Ca2+	0.1716418	0.04998	0.149254	0.00965	0.104478	0.44468
MET activates PTK2 signaling	0.2	0.14494	0.4	2.83E-06	0.033333	0.95514
Transcriptional regulation by RUNX2	0.1652893	0.08763	0.198347	7.33E-05	0.181818	0.0032
ECM proteoglycans	0.1842105	0.0683	0.210526	0.00057	0.052632	0.9486
Nicotinamide salvaging	0.2631579	0.06943	0.368421	0.00065	0.315789	0.0078
Nicotinate metabolism	0.1935484	0.16256	0.290323	0.00083	0.225806	0.02775
Transport of small molecules	0.1217151	0.47778	0.118949	0.00084	0.08852	0.8332
ABC-family proteins mediated transport	0.1165049	0.59567	0.184466	0.00102	0.145631	0.07747
Regulation of RUNX2 expression and activity	0.1232877	0.52474	0.205479	0.00109	0.219178	0.00161
Oxygen-dependent proline hydroxylation of Hypoxia-inducible Factor Alpha	0.1818182	0.09403	0.212121	0.00114	0.272727	4.39E-05

NIK-->noncanonical NF-kB signaling	0.1694915	0.16723	0.220339	0.00117	0.271186	0.00012
Dectin-1 mediated noncanonical NF-kB signaling	0.1833333	0.10112	0.216667	0.00138	0.266667	0.00015
Programmed Cell Death	0.1569767	0.08981	0.156977	0.0014	0.226744	4.39E-07
Regulation of Hypoxia-inducible Factor (HIF) by oxygen	0.1866667	0.06231	0.2	0.00145	0.253333	8.21E-05
Cellular response to hypoxia	0.1866667	0.06231	0.2	0.00145	0.253333	8.21E-05
Peptide ligand-binding receptors	0.1578947	0.07264	0.152632	0.00148	0.126316	0.11846
Negative regulation of NOTCH4 signaling	0.1481481	0.32322	0.222222	0.00166	0.277778	0.00015
Apoptosis	0.1538462	0.113	0.153846	0.00227	0.224852	7.69E-07
ABC transporters in lipid homeostasis	0.1111111	0.65671	0.333333	0.00288	NA	NA
Regulation of mRNA stability by proteins that bind AU-rich elements	0.1590909	0.16919	0.181818	0.00289	0.215909	0.00075
TNFR1-induced proapoptotic signaling	0.3076923	0.06156	0.384615	0.00323	0.307692	0.03207
Collagen chain trimerization	0.1818182	0.15331	0.227273	0.00333	0.045455	0.93895
Vpu mediated degradation of CD4	0.1538462	0.28608	0.211538	0.00384	0.269231	0.00035
The role of GTSE1 in G2/M progression after G2 checkpoint	0.1733333	0.11212	0.186667	0.00403	0.306667	4.13E-07
Antigen processing-Cross presentation	0.1717172	0.08284	0.171717	0.00403	0.191919	0.00319
ER-Phagosome pathway	0.1807229	0.0696	0.180723	0.00409	0.216867	0.00096
Degradation of GLI2 by the proteasome	0.15	0.29414	0.2	0.00421	0.25	0.00053
GLI3 is processed to GLI3R by the proteasome	0.15	0.29414	0.2	0.00421	0.25	0.00053
Antigen activates B Cell Receptor (BCR) leading to generation of second messengers	0.1875	0.18103	0.25	0.00451	0.15625	0.20058
PCP/CE pathway	0.1630435	0.13728	0.173913	0.00457	0.217391	0.0005
Retrograde neurotrophin signalling	0.1428571	0.51672	0.357143	0.00467	NA	NA
DCC mediated attractive signaling	0.2857143	0.07825	0.357143	0.00467	0.214286	0.15199
Disorders of transmembrane transporters	0.1388889	0.28278	0.152778	0.00519	0.111111	0.33873
FBXL7 down-regulates AURKA during mitotic entry and in early mitosis	0.1481481	0.32322	0.203704	0.00521	0.277778	0.00015
Activation of Matrix Metalloproteinases	0.1818182	0.20029	0.242424	0.00552	NA	NA
Metabolism of polyamines	0.1395349	0.33991	0.174419	0.00577	0.232558	0.00019
SCF-beta-TrCP mediated degradation of Emi1	0.1272727	0.50015	0.2	0.00602	0.254545	0.00066
Regulation of RUNX3 expression and activity	0.1454545	0.34211	0.2	0.00602	0.290909	4.91E-05
Anchoring fibril formation	0.2666667	0.09693	0.333333	0.00652	0.066667	0.78794
Platelet Adhesion to exposed collagen	0.2666667	0.09693	0.333333	0.00652	0.2	0.17691
Synthesis of bile acids and bile salts	0.1764706	0.22026	0.235294	0.00669	0.088235	0.66214

Hh mutants that don't undergo autocatalytic processing are degraded by ERAD	0.1607143	0.22786	0.196429	0.00693	0.267857	0.00023
EPHB-mediated forward signaling	0.2142857	0.05917	0.214286	0.00788	0.214286	0.01868
Degradation of DVL	0.1578947	0.24391	0.192982	0.00795	0.263158	0.00029
Hedgehog ligand biogenesis	0.1538462	0.25297	0.184615	0.00818	0.246154	0.00042
Cross-presentation of soluble exogenous antigens (endosomes)	0.14	0.39792	0.2	0.00864	0.28	0.00023
Regulation of activated PAK-2p34 by proteasome mediated degradation	0.14	0.39792	0.2	0.00864	0.28	0.00023
Sphingolipid de novo biosynthesis	0.1395349	0.41793	0.209302	0.00923	0.139535	0.24305
Downstream signal transduction	0.1724138	0.26686	0.241379	0.00949	0.310345	0.00133
VEGFR2 mediated vascular permeability	0.1724138	0.26686	0.241379	0.00949	0.206897	0.05892
Regulation of ornithine decarboxylase (ODC)	0.1372549	0.4186	0.196078	0.00995	0.27451	0.00028
Class I MHC mediated antigen processing & presentation	0.1428571	0.10491	0.121294	0.01	0.15903	0.00012
Hh mutants abrogate ligand secretion	0.1525424	0.27709	0.186441	0.01031	0.254237	0.00043
Collagen biosynthesis and modifying enzymes	0.1492537	0.28444	0.179104	0.01041	0.029851	0.99196
Autodegradation of the E3 ubiquitin ligase COP1	0.1538462	0.28608	0.192308	0.01141	0.288462	9.35E-05
Ubiquitin-dependent degradation of Cyclin D1	0.1346154	0.43921	0.192308	0.01141	0.269231	0.00035
Ubiquitin-dependent degradation of Cyclin D	0.1346154	0.43921	0.192308	0.01141	0.269231	0.00035
ABC transporter disorders	0.1447368	0.30579	0.171053	0.0115	0.197368	0.00634
Trafficking of GluR2-containing AMPA receptors	0.2352941	0.13973	0.294118	0.01164	0.176471	0.22957
SCF(Skp2)-mediated degradation of p27/p21	0.1166667	0.59612	0.183333	0.01168	0.233333	0.00165
Degradation of GLI1 by the proteasome	0.1666667	0.18045	0.183333	0.01168	0.266667	0.00015
Gap junction trafficking	0.2	0.08516	0.2	0.01245	0.2	0.02852
Regulation of Apoptosis	0.1320755	0.45971	0.188679	0.01302	0.264151	0.00044
Ubiquitin Mediated Degradation of Phosphorylated Cdc25A	0.1320755	0.45971	0.188679	0.01302	0.264151	0.00044
p53-Independent DNA Damage Response	0.1320755	0.45971	0.188679	0.01302	0.264151	0.00044
p53-Independent G1/S DNA damage checkpoint	0.1320755	0.45971	0.188679	0.01302	0.264151	0.00044
Regulation of PTEN stability and activity	0.1449275	0.31693	0.173913	0.01309	0.275362	2.34E-05
Platelet degranulation	0.1705426	0.05761	0.147287	0.01311	0.108527	0.38758
Signaling by Hedgehog	0.1632653	0.07424	0.142857	0.01315	0.217687	1.16E-05
Defective CFTR causes cystic fibrosis	0.1311475	0.45693	0.180328	0.01319	0.245902	0.00063
Synthesis of bile acids and bile salts via 7alpha-hydroxycholesterol	0.2083333	0.15472	0.25	0.01345	0.083333	0.69773
Synthesis of very long-chain fatty acyl-CoAs	0.1666667	0.32674	0.25	0.01345	0.166667	0.20465

Antigen processing: Ubiquitination & Proteasome degradation	0.1294498	0.33793	0.122977	0.01384	0.161812	0.00026
Vif-mediated degradation of APOBEC3G	0.1296296	0.48004	0.185185	0.01479	0.259259	0.00054
Crosslinking of collagen fibrils	0.2222222	0.16349	0.277778	0.01501	NA	NA
Gap junction trafficking and regulation	0.212766	0.05031	0.191489	0.01644	0.212766	0.01421
Regulation of Complement cascade	0.1489362	0.33605	0.191489	0.01644	0.148936	0.1735
Autodegradation of Cdh1 by Cdh1:APC/C	0.1111111	0.6489	0.174603	0.01664	0.238095	0.00091
Degradation of AXIN	0.1272727	0.50015	0.181818	0.01674	0.272727	0.00019
AUF1 (hnRNP D0) binds and destabilizes mRNA	0.1428571	0.36115	0.178571	0.01887	0.267857	0.00023
LDL clearance	0.2105263	0.18858	0.263158	0.01897	0.105263	0.56938
Regulation of IFNA signaling	0.1923077	0.19713	0.230769	0.01989	0.192308	0.10507
CLEC7A (Dectin-1) induces NFAT activation	0.1538462	0.47626	0.307692	0.02019	0.153846	0.36968
Transport of inorganic cations/anions and amino acids/oligopeptides	0.1388889	0.31913	0.148148	0.02058	0.092593	0.62535
Degradation of beta-catenin by the destruction complex	0.1341463	0.40104	0.158537	0.02097	0.243902	9.57E-05
Stabilization of p53	0.1403509	0.38029	0.175439	0.0212	0.263158	0.00029
Gap junction assembly	0.1764706	0.22026	0.205882	0.02257	0.235294	0.01505
Cyclin E associated events during G1/S transition	0.1325301	0.41718	0.156627	0.023	0.216867	0.00096
Cell surface interactions at the vascular wall	0.1386861	0.29165	0.138686	0.02359	0.116788	0.26847
Netrin-1 signaling	0.18	0.14138	0.18	0.02406	0.12	0.36682
APC/C:Cdc20 mediated degradation of Securin	0.1044776	0.71252	0.164179	0.02548	0.223881	0.00178
Cell-Cell communication	0.1627907	0.0925	0.139535	0.02562	0.139535	0.07971
Activation of RAC1	0.1428571	0.51672	0.285714	0.02639	0.285714	0.0415
CDT1 association with the CDC6:ORC:origin complex	0.1186441	0.57765	0.169492	0.02646	0.237288	0.00138
Bile acid and bile salt metabolism	0.1860465	0.13922	0.186047	0.02711	0.093023	0.62057
Cyclin A:Cdk2-associated events at S phase entry	0.1411765	0.32492	0.152941	0.02747	0.211765	0.00129
Cellular responses to stress	0.1361502	0.17415	0.112676	0.0278	0.147887	0.00056
Regulation of RAS by GAPs	0.1470588	0.30057	0.161765	0.02814	0.25	0.00023
Immunoregulatory interactions between a Lymphoid and a non-Lymphoid cell	0.1363636	0.3246	0.136364	0.03143	0.090909	0.65417
Activation of BAD and translocation to mitochondria	0.2	0.26759	0.266667	0.03362	0.466667	0.00027
Synthesis of Prostaglandins (PG) and Thromboxanes (TX)	0.2666667	0.09693	0.266667	0.03362	0.2	0.17691
WNT5A-dependent internalization of FZD4	0.2	0.26759	0.266667	0.03362	NA	NA
Intrinsic Pathway of Fibrin Clot Formation	0.2272727	0.11668	0.227273	0.03474	0.090909	0.65065
Orc1 removal from chromatin	0.0985915	0.76792	0.15493	0.03732	0.197183	0.00828

Regulation of APC/C activators between G1/S and early anaphase	0.0875	0.86317	0.15	0.03803	0.1875	0.01025
Signalling to ERKs	0.2333333	0.06152	0.2	0.03838	0.2	0.0678
DAP12 signaling	0.2	0.14494	0.2	0.03838	0.2	0.0678
CDO in myogenesis	0.1333333	0.49664	0.2	0.03838	0.133333	0.33951
Myogenesis	0.1333333	0.49664	0.2	0.03838	0.133333	0.33951
APC/C:Cdh1 mediated degradation of Cdc20 and other APC/C:Cdh1 targeted proteins in late mitosis/early G1	0.0972222	0.78047	0.152778	0.04079	0.208333	0.00374
Cdc20:Phospho-APC/C mediated degradation of Cyclin A	0.0972222	0.78047	0.152778	0.04079	0.208333	0.00374
CDK-mediated phosphorylation and removal of Cdc6	0.0972222	0.78047	0.152778	0.04079	0.208333	0.00374
Class A/1 (Rhodopsin-like receptors)	0.1481481	0.07437	0.114198	0.04127	0.108025	0.2985
Initial triggering of complement	0.2173913	0.1351	0.217391	0.04138	0.217391	0.06834
Role of LAT2/NTAL/LAB on calcium mobilization	0.25	0.11748	0.25	0.04189	0.375	0.00297
Rap1 signalling	0.25	0.11748	0.25	0.04189	0.1875	0.20284
MAP3K8 (TPL2)-dependent MAPK1/3 activation	0.25	0.11748	0.25	0.04189	0.1875	0.20284
Asymmetric localization of PCP proteins	0.109375	0.66555	0.15625	0.04357	0.265625	0.0001
APC:Cdc20 mediated degradation of cell cycle proteins prior to satisfation of the cell cycle checkpoint	0.0958904	0.79252	0.150685	0.04449	0.205479	0.00429
Ion channel transport	0.1236559	0.48139	0.123656	0.04521	0.096774	0.56237
Cellular responses to external stimuli	0.1371769	0.13513	0.107356	0.04544	0.147117	0.00022
Prolonged ERK activation events	0.1	0.72331	0.3	0.04722	0.2	0.25677
p75NTR regulates axonogenesis	0.2	0.34377	0.3	0.04722	0.2	0.25677
Thyroxine biosynthesis	0.3	0.1097	0.3	0.04722	0.1	0.6443
Regulation of commissural axon pathfinding by SLIT and ROBO	0.3	0.1097	0.3	0.04722	0.1	0.6443
RUNX1 and FOXP3 control the development of regulatory T lymphocytes (Tregs)	0.2	0.34377	0.3	0.04722	NA	NA
Cytosolic sulfonation of small molecules	0.1666667	0.32674	0.208333	0.04873	0.125	0.42356
RUNX2 regulates osteoblast differentiation	0.2083333	0.15472	0.208333	0.04873	0.041667	0.91647
Formation of tubulin folding intermediates by CCT/TriC	0.2173913	0.1351	0.173913	0.12766	0.434783	2.76E-05
G2/M Transition	0.1597938	0.0606	0.108247	0.15346	0.190722	5.60E-05
Mitotic G2-G2/M phases	0.1581633	0.06773	0.107143	0.16465	0.188776	7.02E-05
Hedgehog 'on' state	0.1529412	0.21977	0.141176	0.05649	0.235294	0.00016
Post-chaperonin tubulin folding pathway	0.25	0.08377	0.2	0.08521	0.4	0.00036

Cooperation of Prefoldin and TriC/CCT in actin and tubulin folding	0.2	0.14494	0.133333	0.2509	0.333333	0.00038
Prefoldin mediated transfer of substrate to CCT/TriC	0.2307692	0.08433	0.153846	0.1771	0.346154	0.00055
RHO GTPase Effectors	0.1297468	0.32975	0.088608	0.44703	0.155063	0.0008
Protein folding	0.1414141	0.30309	0.090909	0.47324	0.20202	0.00132
Chaperonin-mediated protein folding	0.1505376	0.22608	0.096774	0.39843	0.204301	0.0015
p53-Dependent G1 DNA Damage Response	0.1212121	0.54974	0.151515	0.05214	0.227273	0.00151
p53-Dependent G1/S DNA damage checkpoint	0.1212121	0.54974	0.151515	0.05214	0.227273	0.00151
mRNA Splicing - Major Pathway	0.0983607	0.85255	0.027322	0.99967	0.169399	0.00174
Cholesterol biosynthesis	0.24	0.07187	0.08	0.64221	0.32	0.00198
G1/S DNA Damage Checkpoints	0.1176471	0.58503	0.147059	0.06175	0.220588	0.00208
RHO GTPases activate CIT	0.2	0.21478	0.15	0.2409	0.35	0.00212
Signaling by NOTCH	0.1574468	0.05259	0.114894	0.06866	0.157447	0.00255
Signaling by Rho GTPases	0.1418919	0.09235	0.087838	0.45023	0.13964	0.00261
TP53 Regulates Transcription of Cell Death Genes	0.2045455	0.07585	0.159091	0.07725	0.25	0.00284
mRNA Splicing	0.0942408	0.89541	0.026178	0.99981	0.162304	0.00344
Mitotic Anaphase	0.0954774	0.88883	0.100503	0.2536	0.160804	0.00346
Mitotic Metaphase and Anaphase	0.095	0.89353	0.1	0.26086	0.16	0.00374
Processing of Capped Intron-Containing Pre-mRNA	0.0946502	0.91568	0.020576	1	0.152263	0.00453
Separation of Sister Chromatids	0.0904255	0.92267	0.095745	0.34026	0.159574	0.0051
APC/C:Cdc20 mediated degradation of mitotic proteins	0.0933333	0.81509	0.146667	0.05255	0.2	0.00559
Assembly of the pre-replicative complex	0.1029412	0.72715	0.147059	0.06175	0.205882	0.00559
Signaling by cytosolic FGFR1 fusion mutants	0.1111111	0.65671	0.166667	0.19471	0.333333	0.00581
Activation of APC/C and APC/C:Cdc20 mediated degradation of mitotic proteins	0.0921053	0.82564	0.144737	0.05692	0.197368	0.00634
Host Interactions of HIV factors	0.125	0.47851	0.125	0.07813	0.171875	0.0064
HIV Infection	0.1077586	0.75593	0.099138	0.25507	0.150862	0.00655
RHO GTPases activate PAKs	0.2083333	0.15472	0.125	0.33697	0.291667	0.00667
Kinesins	0.1403509	0.38029	0.122807	0.2106	0.210526	0.00828
Signaling by NTRK2 (TRKB)	0.24	0.07187	0.12	0.36111	0.28	0.0085
Deubiquitination	0.1144781	0.65424	0.10101	0.18937	0.141414	0.00968
FCERI mediated MAPK activation	0.21875	0.0825	0.125	0.28995	0.25	0.01038
Constitutive Signaling by EGFRvIII	0.2666667	0.09693	0.2	0.13062	0.333333	0.01172
Signaling by EGFRvIII in Cancer	0.2666667	0.09693	0.2	0.13062	0.333333	0.01172

TCF dependent signaling in response to WNT	0.1030043	0.82373	0.081545	0.61843	0.145923	0.01195
Transcriptional Regulation by TP53	0.1260274	0.39553	0.068493	0.90104	0.134247	0.01428
STING mediated induction of host immune responses	0.25	0.11748	0.1875	0.1511	0.3125	0.0157
Metabolism of RNA	0.074184	0.99998	0.043027	1	0.123145	0.01644
Recruitment of NuMA to mitotic centrosomes	0.1630435	0.13728	0.086957	0.53243	0.173913	0.01651
MET activates RAS signaling	0.1818182	0.38952	0.181818	0.24053	0.363636	0.01734
APC/C-mediated degradation of cell cycle proteins	0.0813953	0.90683	0.139535	0.06079	0.174419	0.01939
Regulation of mitotic cell cycle	0.0813953	0.90683	0.139535	0.06079	0.174419	0.01939
Regulation of TP53 Degradation	0.1666667	0.26199	0.083333	0.60391	0.222222	0.02106
Carboxyterminal post-translational modifications of tubulin	0.1388889	0.44121	0.111111	0.36979	0.222222	0.02106
Regulation of TP53 Expression and Degradation	0.1891892	0.15045	0.081081	0.62281	0.216216	0.02464
Ub-specific processing proteases	0.0909091	0.93357	0.095455	0.32799	0.140909	0.02488
Synthesis of PIPs at the Golgi membrane	0.2777778	0.05656	0.055556	0.79963	0.277778	0.02612
FGFR1 mutant receptor activation	0.1935484	0.16256	0.16129	0.11994	0.225806	0.02775
RHO GTPases Activate Formins	0.1449275	0.22094	0.086957	0.51778	0.152174	0.0279
Switching of origins to a post-replicative state	0.0777778	0.9288	0.133333	0.08015	0.166667	0.02827
Signaling by FGFR1 in disease	0.1578947	0.30554	0.131579	0.22066	0.210526	0.02861
Synthesis of PIPs at the plasma membrane	0.1886792	0.09872	0.075472	0.67388	0.188679	0.03141
IRF3-mediated induction of type I IFN	0.2307692	0.2002	0.153846	0.30618	0.307692	0.03207
Chk1/Chk2(Cds1) mediated inactivation of Cyclin B:Cdk1 complex	NA	NA	0.153846	0.30618	0.307692	0.03207
Signaling by WNT	0.1242424	0.44236	0.093939	0.31346	0.130303	0.03239
SUMOylation of RNA binding proteins	0.1086957	0.66515	0.021739	0.98366	0.195652	0.03248
Constitutive Signaling by Ligand-Responsive EGFR Cancer Variants	0.2105263	0.18858	0.157895	0.21755	0.263158	0.03267
Signaling by EGFR in Cancer	0.2105263	0.18858	0.157895	0.21755	0.263158	0.03267
Signaling by Ligand-Responsive EGFR Variants in Cancer	0.2105263	0.18858	0.157895	0.21755	0.263158	0.03267
Regulation of TLR by endogenous ligand	0.1052632	0.68619	0.105263	0.49121	0.263158	0.03267
Neddylation	0.1153846	0.62691	0.115385	0.06587	0.136752	0.03337
Mitotic G1-G1/S phases	0.114094	0.63378	0.120805	0.08376	0.147651	0.03366
Factors involved in megakaryocyte development and platelet production	0.1566265	0.09646	0.078313	0.66923	0.144578	0.03418
Signaling by ROBO receptors	0.0963303	0.89035	0.110092	0.1177	0.137615	0.03575
DNA Replication Pre-Initiation	0.0823529	0.90051	0.117647	0.18697	0.164706	0.03644

Antiviral mechanism by IFN-stimulated genes	0.1153846	0.60787	0.102564	0.34796	0.166667	0.03935
UCH proteinases	0.1176471	0.58154	0.127451	0.09331	0.156863	0.03969
RHO GTPases Activate ROCKs	0.2	0.21478	0.1	0.51902	0.25	0.04015
Organelle biogenesis and maintenance	0.1326531	0.28368	0.061224	0.95169	0.129252	0.04691
Deactivation of the beta-catenin transactivating complex	0.1904762	0.12582	0.071429	0.70782	0.190476	0.04888
Cooperation of PDCL (PhLP1) and TRiC/CCT in G-protein beta folding	0.1190476	0.58256	0.047619	0.88454	0.190476	0.04888

Table S4A. Enriched Reactome pathways from up-regulated genes at 8h pi. Significant Reactome pathways using up-regulated DEGs from STM, T3SS-1^{mut} or T3SS-2^{mut}-infected HIOs at 8h.

Table S4B: Reactome Pathways Downregulated 8h pi

Description	STM		T3SS-1^{mut}		T3SS-2^{mut}	
	gene ratio	p-value	gene ratio	p-value	gene ratio	p-value
Translation	0.4261168	2.59E-39	0.1237113	4.07E-05	0.2061856	8.84E-11
Metabolism of RNA	0.268546	4.24E-26	0.0771513	0.04784	0.0919881	0.2981
Influenza Viral RNA Transcription and Replication	0.4962406	5.92E-26	0.1578947	5.39E-05	0.2556391	4.15E-09
Eukaryotic Translation Termination	0.5806452	7.68E-26	0.1827957	4.17E-05	0.311828	3.79E-10
Influenza Life Cycle	0.4755245	2.58E-25	0.1468531	0.00016	0.2447552	8.44E-09
Selenoamino acid metabolism	0.5169492	2.61E-25	0.1949153	5.95E-07	0.2881356	1.30E-10
Eukaryotic Translation Elongation	0.5698925	7.64E-25	0.1935484	1.10E-05	0.3225806	7.31E-11
rRNA processing in the nucleus and cytosol	0.4102564	2.72E-24	0.1282051	0.00035	0.1846154	8.13E-06
SRP-dependent cotranslational protein targeting to membrane	0.5178571	3.57E-24	0.1517857	0.00044	0.3035714	2.65E-11
Peptide chain elongation	0.5730337	4.19E-24	0.2022472	5.77E-06	0.3258427	1.15E-10
Viral mRNA Translation	0.5730337	4.19E-24	0.1910112	2.30E-05	0.3258427	1.15E-10
Selenocysteine synthesis	0.5591398	7.28E-24	0.1935484	1.10E-05	0.3333333	1.34E-11
Major pathway of rRNA processing in the nucleolus and cytosol	0.4108108	3.57E-23	0.1297297	0.00038	0.1891892	6.18E-06
Influenza Infection	0.4415584	5.39E-23	0.1428571	0.00017	0.2272727	6.38E-08
rRNA processing	0.3902439	1.28E-22	0.1219512	0.00074	0.1756098	2.55E-05
Cell Cycle	0.2605304	1.62E-22	0.1060842	4.63E-06	0.0904836	0.35377
Nonsense Mediated Decay (NMD) independent of the Exon Junction Complex (EJC)	0.5368421	2.44E-22	0.1789474	5.53E-05	0.3052632	6.67E-10
Formation of a pool of free 40S subunits	0.5148515	1.20E-21	0.1881188	9.71E-06	0.2871287	3.27E-09
L13a-mediated translational silencing of Ceruloplasmin expression	0.4864865	6.21E-21	0.1711712	3.90E-05	0.2702703	8.42E-09
GTP hydrolysis and joining of the 60S ribosomal subunit	0.4821429	1.06E-20	0.1696429	4.44E-05	0.2678571	1.06E-08
Nonsense-Mediated Decay (NMD)	0.4608696	3.20E-19	0.1565217	0.0002	0.2608696	2.06E-08
Nonsense Mediated Decay (NMD) enhanced by the Exon Junction Complex (EJC)	0.4608696	3.20E-19	0.1565217	0.0002	0.2608696	2.06E-08
Eukaryotic Translation Initiation	0.4537815	3.55E-19	0.1596639	0.0001	0.2605042	1.24E-08
Cap-dependent Translation Initiation	0.4537815	3.55E-19	0.1596639	0.0001	0.2605042	1.24E-08
Cell Cycle, Mitotic	0.255597	9.29E-18	0.1100746	6.66E-06	0.0895522	0.39979
Cell Cycle Checkpoints	0.3105802	9.72E-18	0.1194539	0.00011	0.0853242	0.54404
Metabolism of amino acids and derivatives	0.2864865	1.22E-17	0.1324324	2.16E-07	0.2	2.46E-12
S Phase	0.3664596	1.66E-15	0.2049689	5.47E-10	0.1552795	0.00259

Activation of the pre-replicative complex	0.7272727	1.80E-15	0.4545455	2.00E-10	0.3333333	5.81E-05
Activation of ATR in response to replication stress	0.6756757	7.25E-15	0.4594595	9.71E-12	0.3243243	3.66E-05
DNA Repair	0.2816456	1.73E-14	0.1234177	2.06E-05	0.1107595	0.07013
G1/S Transition	0.3816794	3.80E-14	0.2290076	1.97E-10	0.1679389	0.00168
DNA strand elongation	0.6875	1.79E-13	0.53125	4.61E-13	0.40625	9.35E-07
DNA Replication	0.3779528	1.91E-13	0.2125984	9.03E-09	0.1732283	0.00111
Regulation of expression of SLITs and ROBOs	0.3333333	5.45E-13	0.1111111	0.0084	0.1754386	0.00012
Mitotic G1-G1/S phases	0.3489933	7.54E-13	0.2214765	6.29E-11	0.1677852	0.00085
Synthesis of DNA	0.3697479	4.69E-12	0.2016807	1.74E-07	0.1680672	0.00264
G2/M Checkpoints	0.3214286	1.13E-11	0.1428571	8.52E-05	0.1011905	0.2737
Chromosome Maintenance	0.3636364	8.01E-11	0.1181818	0.01665	0.1090909	0.23341
Homology Directed Repair	0.3333333	9.67E-11	0.1304348	0.00185	0.0869565	0.52576
HDR through Homologous Recombination (HRR) or Single Strand Annealing (SSA)	0.3333333	2.47E-10	0.1287879	0.0028	0.0909091	0.46216
HDR through Homologous Recombination (HRR)	0.4328358	2.64E-10	0.1791045	0.00067	0.1492537	0.05857
Mitochondrial translation	0.3763441	4.11E-10	0.0645161	0.50791	0.1827957	0.00217
Infectious disease	0.2356021	5.79E-10	0.0890052	0.01761	0.1151832	0.02673
Extension of Telomeres	0.6	8.48E-10	0.3	5.09E-05	0.3	0.00067
Signaling by ROBO receptors	0.2752294	9.67E-10	0.0917431	0.04545	0.1513761	0.00092
The citric acid (TCA) cycle and respiratory electron transport	0.2931034	1.68E-09	0.0804598	0.17917	0.1436782	0.00724
DNA Double-Strand Break Repair	0.2951807	2.71E-09	0.1204819	0.00278	0.0843373	0.56869
Homologous DNA Pairing and Strand Exchange	0.5	3.15E-09	0.2380952	0.00017	0.1904762	0.0245
Presynaptic phase of homologous DNA pairing and strand exchange	0.5128205	4.24E-09	0.2307692	0.00046	0.2051282	0.01602
Mitochondrial translation elongation	0.3678161	4.49E-09	0.0689655	0.44286	0.1954023	0.00101
Mitochondrial translation termination	0.3678161	4.49E-09	0.0689655	0.44286	0.183908	0.00271
DNA Replication Pre-Initiation	0.3647059	9.90E-09	0.2	1.22E-05	0.1529412	0.02858
HDR through Single Strand Annealing (SSA)	0.5135135	1.01E-08	0.2702703	5.30E-05	0.2162162	0.0117
Telomere C-strand (Lagging Strand) Synthesis	0.625	1.02E-08	0.375	6.49E-06	0.2916667	0.00316
Processing of Capped Intron-Containing Pre-mRNA	0.255144	1.27E-08	0.0534979	0.73237	0.0411523	0.99802
Respiratory electron transport	0.34	1.53E-08	0.1	0.08486	0.15	0.02307
Mitochondrial translation initiation	0.3563218	1.88E-08	0.0689655	0.44286	0.183908	0.00271
Activation of E2F1 target genes at G1/S	0.5714286	2.00E-08	0.4642857	2.39E-09	0.3928571	9.62E-06
G1/S-Specific Transcription	0.5714286	2.00E-08	0.4642857	2.39E-09	0.3928571	9.62E-06

tRNA Aminoacylation	0.4761905	2.18E-08	0.2380952	0.00017	0.1428571	0.14735
Processing of DNA double-strand break ends	0.3367347	3.26E-08	0.122449	0.01619	0.0918367	0.46761
Formation of the ternary complex, and subsequently, the 43S complex	0.4313725	4.13E-08	0.1960784	0.00089	0.2156863	0.00342
Unwinding of DNA	0.8333333	4.32E-08	0.6666667	7.51E-08	0.5	0.00023
Telomere Maintenance	0.3536585	6.50E-08	0.1219512	0.0275	0.1341463	0.09087
Mitotic Spindle Checkpoint	0.3125	1.06E-07	0.0982143	0.08086	0.0625	0.85671
Translation initiation complex formation	0.3965517	1.35E-07	0.1724138	0.00248	0.2068966	0.00329
Ribosomal scanning and start codon recognition	0.3965517	1.35E-07	0.1724138	0.00248	0.2068966	0.00329
G2/M DNA damage checkpoint	0.3263158	1.90E-07	0.1157895	0.03012	0.0842105	0.57631
Activation of the mRNA upon binding of the cap-binding complex and eIFs, and subsequent binding to 43S	0.3898305	1.95E-07	0.1694915	0.00283	0.220339	0.00123
CDC6 association with the ORC:origin complex	0.8181818	2.93E-07	0.5454545	1.82E-05	0.4545455	0.00137
Respiratory electron transport, ATP synthesis by chemiosmotic coupling, and heat production by uncoupling proteins.	0.2926829	4.33E-07	0.0813008	0.22037	0.1300813	0.061
Dual Incision in GG-NER	0.4390244	5.14E-07	0.195122	0.00298	0.1707317	0.05777
Resolution of Sister Chromatid Cohesion	0.2903226	5.38E-07	0.0806452	0.22764	0.0403226	0.98479
Lagging Strand Synthesis	0.6	6.05E-07	0.45	1.04E-06	0.35	0.00096
Transcriptional Regulation by TP53	0.2136986	6.58E-07	0.1041096	0.00089	0.1178082	0.02016
Amplification of signal from the kinetochores	0.3125	8.53E-07	0.09375	0.13254	0.0416667	0.97001
Amplification of signal from unattached kinetochores via a MAD2 inhibitory signal	0.3125	8.53E-07	0.09375	0.13254	0.0416667	0.97001
M Phase	0.2086514	9.54E-07	0.0508906	0.83564	0.043257	0.99971
Mitotic Prometaphase	0.2474747	1.13E-06	0.0707071	0.3271	0.0454545	0.99054
Transcription-Coupled Nucleotide Excision Repair (TC-NER)	0.3291139	1.52E-06	0.1265823	0.02178	0.164557	0.01637
Mitotic Metaphase and Anaphase	0.245	1.55E-06	0.065	0.45272	0.045	0.99149
Dual incision in TC-NER	0.3484848	1.99E-06	0.1363636	0.0185	0.1666667	0.02386
mRNA Splicing	0.2460733	2.21E-06	0.052356	0.73988	0.0366492	0.9979
Resolution of D-loop Structures through Holliday Junction Intermediates	0.4545455	2.70E-06	0.1818182	0.01378	0.1515152	0.14863
tRNA processing	0.2924528	2.75E-06	0.1226415	0.01247	0.1037736	0.30022
Mitochondrial Fatty Acid Beta-Oxidation	0.4324324	2.83E-06	0.1891892	0.00641	0.3243243	3.66E-05
Mitotic Anaphase	0.241206	3.16E-06	0.0653266	0.44544	0.0452261	0.99103

Resolution of D-loop Structures through Synthesis-Dependent Strand Annealing (SDSA)	0.5	3.35E-06	0.2307692	0.00412	0.1923077	0.06686
Regulation of TP53 Activity through Phosphorylation	0.3043478	3.52E-06	0.1413043	0.00382	0.1195652	0.16385
mRNA Splicing - Major Pathway	0.2459016	3.69E-06	0.0491803	0.79619	0.0382514	0.99659
Resolution of D-Loop Structures	0.4411765	4.29E-06	0.1764706	0.01589	0.1470588	0.16275
Gap-filling DNA repair synthesis and ligation in TC-NER	0.3384615	5.73E-06	0.1384615	0.01686	0.1846154	0.00853
Recognition of DNA damage by PCNA-containing replication complex	0.4516129	6.42E-06	0.2258065	0.00225	0.1935484	0.04531
Nucleotide Excision Repair	0.2792793	7.89E-06	0.1171171	0.01784	0.1261261	0.09278
Separation of Sister Chromatids	0.2393617	7.90E-06	0.0638298	0.48343	0.0425532	0.99323
Fanconi Anemia Pathway	0.4	9.61E-06	0.15	0.03334	0.15	0.12413
Mitochondrial tRNA aminoacylation	0.5238095	1.09E-05	0.2857143	0.00126	0.1904762	0.10004
PCNA-Dependent Long Patch Base Excision Repair	0.5238095	1.09E-05	0.2857143	0.00126	0.2380952	0.02942
Mismatch Repair	0.6	1.67E-05	0.2666667	0.01104	0.2	0.13224
Processive synthesis on the lagging strand	0.6	1.67E-05	0.4666667	1.30E-05	0.3333333	0.00667
Global Genome Nucleotide Excision Repair (GG-NER)	0.297619	1.78E-05	0.1309524	0.01306	0.1071429	0.29336
E2F mediated regulation of DNA replication	0.5	1.94E-05	0.2727273	0.00165	0.2272727	0.03547
Switching of origins to a post-replicative state	0.2888889	2.19E-05	0.1555556	0.00107	0.1333333	0.08277
Resolution of AP sites via the multiple-nucleotide patch replacement pathway	0.4615385	2.27E-05	0.2307692	0.00412	0.1923077	0.06686
Metabolism of nucleotides	0.2772277	2.48E-05	0.1584158	0.00039	0.1683168	0.00526
snRNP Assembly	0.3461538	2.84E-05	0.0961538	0.21124	0.0576923	0.83549
Metabolism of non-coding RNA	0.3461538	2.84E-05	0.0961538	0.21124	0.0576923	0.83549
Branched-chain amino acid catabolism	0.4782609	3.31E-05	0.2173913	0.01132	0.3043478	0.00242
Interactions of Rev with host cellular proteins	0.4	3.47E-05	0.0857143	0.36296	0.0571429	0.8152
Protein localization	0.2519685	5.47E-05	0.0629921	0.51915	0.1338583	0.0436
Complex I biogenesis	0.3272727	6.65E-05	0.0545455	0.66288	0.1272727	0.18972
SUMOylation	0.2245989	7.74E-05	0.0855615	0.10938	0.0695187	0.82449
Rev-mediated nuclear export of HIV RNA	0.3939394	7.97E-05	0.0909091	0.32877	0.0606061	0.78847
Methylation	0.5714286	8.20E-05	0.2142857	0.0501	0.1428571	0.3412
Mismatch repair (MMR) directed by MSH2:MSH6 (MutSalpha)	0.5714286	8.20E-05	0.2857143	0.0085	0.2142857	0.11264
Mismatch repair (MMR) directed by MSH2:MSH3 (MutSbeta)	0.5714286	8.20E-05	0.2142857	0.0501	0.2142857	0.11264
Removal of the Flap Intermediate	0.5714286	8.20E-05	0.4285714	0.0001	0.2857143	0.02688

Gap-filling DNA repair synthesis and ligation in GG-NER	0.44	8.61E-05	0.2	0.01616	0.2	0.05792
Viral Messenger RNA Synthesis	0.3571429	8.67E-05	0.0952381	0.25437	0.1190476	0.29081
SUMOylation of DNA damage response and repair proteins	0.2894737	8.89E-05	0.1052632	0.0922	0.0526316	0.90109
rRNA modification in the nucleus and cytosol	0.3064516	0.00011	0.0967742	0.1774	0.0645161	0.79085
NEP/NS2 Interacts with the Cellular Export Machinery	0.4	0.00013	0.1	0.27751	0.0666667	0.742
Base Excision Repair	0.3589744	0.00014	0.2307692	0.00046	0.2307692	0.00489
Resolution of Abasic Sites (AP sites)	0.3589744	0.00014	0.2307692	0.00046	0.2307692	0.00489
Regulation of HSF1-mediated heat shock response	0.2941176	0.00014	0.0588235	0.60514	0.0882353	0.5345
Assembly of the pre-replicative complex	0.2941176	0.00014	0.1911765	0.00021	0.1617647	0.02916
Peroxisomal protein import	0.3015873	0.00015	0.047619	0.7501	0.2063492	0.00231
Nucleobase biosynthesis	0.5333333	0.00016	0.3333333	0.00152	0.3333333	0.00667
SUMO E3 ligases SUMOylate target proteins	0.2209945	0.00016	0.0828729	0.14292	0.0718232	0.78849
DNA Damage Bypass	0.3265306	0.00017	0.1836735	0.00258	0.1428571	0.12336
Deposition of new CENPA-containing nucleosomes at the centromere	0.2837838	0.00017	0.0405405	0.83941	0.027027	0.98981
Nucleosome assembly	0.2837838	0.00017	0.0405405	0.83941	0.027027	0.98981
Meiosis	0.2457627	0.00019	0.0508475	0.73669	0.059322	0.89002
G0 and Early G1	0.4074074	0.0002	0.2962963	0.00015	0.1481481	0.19751
DNA Damage/Telomere Stress Induced Senescence	0.275	0.0002	0.0375	0.8754	0.0625	0.82789
TP53 Regulates Transcription of DNA Repair Genes	0.2923077	0.00023	0.1230769	0.04382	0.1384615	0.10224
Pyruvate metabolism and Citric Acid (TCA) cycle	0.3090909	0.00023	0.1090909	0.1182	0.1636364	0.0431
Transport of Mature mRNAs Derived from Intronless Transcripts	0.3414634	0.00026	0.0731707	0.46298	0.0487805	0.87815
Export of Viral Ribonucleoproteins from Nucleus	0.375	0.00026	0.09375	0.31165	0.09375	0.5262
Nuclear import of Rev protein	0.375	0.00026	0.09375	0.31165	0.0625	0.77386
Termination of translesion DNA synthesis	0.375	0.00026	0.1875	0.01187	0.15625	0.13505
Orc1 removal from chromatin	0.2816901	0.00027	0.1690141	0.00114	0.1267606	0.1527
RHO GTPases Activate Formins	0.2318841	0.00029	0.0652174	0.47187	0.0362319	0.99367
tRNA processing in the nucleus	0.3035714	0.00029	0.0892857	0.25716	0.1071429	0.34846
Cellular response to heat stress	0.2613636	0.00033	0.0568182	0.63248	0.0795455	0.63958
mRNA Splicing - Minor Pathway	0.3076923	0.00037	0.0576923	0.62497	0.0769231	0.66342
Translesion synthesis by POLI	0.4705882	0.00047	0.2941176	0.00282	0.2352941	0.05208
Transport of the SLBP Dependant Mature mRNA	0.3529412	0.0005	0.0882353	0.34589	0.0588235	0.80223
Nuclear Pore Complex (NPC) Disassembly	0.3529412	0.0005	0.0882353	0.34589	0.0588235	0.80223

Transport of Ribonucleoproteins into the Host Nucleus	0.3666667	0.00059	0.1	0.27751	0.0666667	0.742
Regulation of Glucokinase by Glucokinase Regulatory Protein	0.3666667	0.00059	0.1	0.27751	0.0666667	0.742
Calnexin/calreticulin cycle	0.3846154	0.00067	0.0384615	0.80684	0.1153846	0.38857
N-glycan trimming in the ER and Calnexin/Calreticulin cycle	0.3428571	0.00067	0.0571429	0.64067	0.1428571	0.17739
Polymerase switching on the C-strand of the telomere	0.5	0.00069	0.3571429	0.00106	0.2857143	0.02688
Polymerase switching	0.5	0.00069	0.3571429	0.00106	0.2857143	0.02688
Leading Strand Synthesis	0.5	0.00069	0.3571429	0.00106	0.2857143	0.02688
Transport of Mature mRNA Derived from an Intronless Transcript	0.325	0.00073	0.075	0.44672	0.05	0.86925
Defects in vitamin and cofactor metabolism	0.4090909	0.00074	0.1363636	0.14852	0.1818182	0.11439
Pyruvate metabolism	0.3548387	0.00081	0.0645161	0.57396	0.1935484	0.04531
Diseases of metabolism	0.2380952	0.00085	0.1333333	0.00467	0.152381	0.01685
Glutathione conjugation	0.3333333	0.0009	0.1666667	0.02076	0.25	0.00274
Mitotic Prophase	0.2198582	0.00094	0.0283688	0.97641	0.035461	0.99478
Processive synthesis on the C-strand of the telomere	0.5454545	0.00095	0.4545455	0.00029	0.3636364	0.0109
SeMet incorporation into proteins	0.5454545	0.00095	0.2727273	0.02602	0.1818182	0.24253
mitochondrial fatty acid beta-oxidation of saturated fatty acids	0.5454545	0.00095	0.3636364	0.00325	0.4545455	0.00137
Nuclear Envelope Breakdown	0.2941176	0.00095	0.0784314	0.38084	0.0588235	0.82557
Regulation of TP53 Activity	0.2125	0.00103	0.09375	0.06535	0.09375	0.39985
Vpr-mediated nuclear import of PICs	0.34375	0.00109	0.09375	0.31165	0.0625	0.77386
Translesion Synthesis by POLH	0.4210526	0.00116	0.2631579	0.00479	0.2631579	0.01939
Transcription of E2F targets under negative control by DREAM complex	0.4210526	0.00116	0.4210526	7.80E-06	0.2105263	0.07409
Transport of the SLBP independent Mature mRNA	0.3333333	0.00146	0.0909091	0.32877	0.0606061	0.78847
SUMOylation of SUMOylation proteins	0.3333333	0.00146	0.0909091	0.32877	0.0606061	0.78847
Meiotic recombination	0.244186	0.00153	0.0465116	0.78019	0.0581395	0.87164
Cytosolic tRNA aminoacylation	0.375	0.00154	0.1666667	0.05567	0.0833333	0.62285
Formation of Incision Complex in GG-NER	0.3023256	0.00155	0.1395349	0.04544	0.0930233	0.51079
Purine ribonucleoside monophosphate biosynthesis	0.5	0.0017	0.3333333	0.00463	0.3333333	0.01525
Metabolism of cofactors	0.4	0.00173	0.2	0.03074	0.3	0.00534
SUMOylation of chromatin organization proteins	0.2571429	0.00174	0.0571429	0.62818	0.0714286	0.72907
Formation of TC-NER Pre-Incision Complex	0.2777778	0.00181	0.0740741	0.42307	0.1481481	0.08817
Translesion synthesis by REV1	0.4375	0.00183	0.25	0.01401	0.25	0.04262

Transcription of E2F targets under negative control by p107 (RBL1) and p130 (RBL2) in complex with HDAC1	0.4375	0.00183	0.3125	0.0021	0.125	0.40484
Transcriptional regulation by small RNAs	0.2285714	0.00195	0.0380952	0.89182	0.0571429	0.89691
Translesion synthesis by Y family DNA polymerases bypasses lesions on DNA template	0.3076923	0.00197	0.1538462	0.02982	0.1282051	0.24028
Transport of Mature Transcript to Cytoplasm	0.2409639	0.00233	0.060241	0.58012	0.0361446	0.9778
Interactions of Vpr with host cellular proteins	0.3142857	0.00249	0.0857143	0.36296	0.0571429	0.8152
ER Quality Control Compartment (ERQC)	0.3809524	0.0025	NA	NA	0.0952381	0.54891
Deadenylation-dependent mRNA decay	0.2678571	0.00267	NA	NA	0.1071429	0.34846
Reproduction	0.2083333	0.00272	0.0416667	0.88205	0.0555556	0.93513
Translesion synthesis by POLK	0.4117647	0.00278	0.2352941	0.01746	0.2352941	0.05208
HIV Life Cycle	0.205298	0.00296	0.0794702	0.21401	0.0794702	0.65351
Phase II - Conjugation of compounds	0.2201835	0.0033	0.1192661	0.01552	0.1559633	0.01126
Cellular responses to stress	0.1690141	0.00341	0.0446009	0.9464	0.0633803	0.96672
RNA Polymerase I Transcription Initiation	0.2765957	0.00372	0.0851064	0.32414	0.0638298	0.78061
SUMOylation of ubiquitinylation proteins	0.2972973	0.00404	0.0810811	0.39687	0.0540541	0.83887
Removal of the Flap Intermediate from the C-strand	0.5	0.00427	0.4	0.00217	0.3	0.04789
RNA Polymerase I Transcription	0.2142857	0.00475	0.0446429	0.82354	0.0446429	0.96885
tRNA modification in the nucleus and cytosol	0.2790698	0.00483	0.1860465	0.00406	0.1162791	0.30807
Cellular Senescence	0.1897436	0.00509	0.0512821	0.7626	0.0666667	0.8651
Processing of Capped Intronless Pre-mRNA	0.3214286	0.00513	0.0357143	0.82982	NA	NA
Fatty acid metabolism	0.1920904	0.0058	0.0960452	0.04323	0.1581921	0.00111
SUMOylation of DNA replication proteins	0.2727273	0.00591	0.0909091	0.28198	0.0681818	0.74095
NS1 Mediated Effects on Host Pathways	0.2820513	0.00628	0.1025641	0.21415	0.0512821	0.85976
mRNA decay by 5' to 3' exoribonuclease	0.4	0.00663	NA	NA	0.1333333	0.37335
RNA Polymerase I Promoter Clearance	0.2110092	0.00685	0.0366972	0.90771	0.0366972	0.98697
Meiotic synapsis	0.2278481	0.00703	0.0379747	0.86994	0.0506329	0.91661
Establishment of Sister Chromatid Cohesion	0.4545455	0.00703	0.0909091	0.50099	0.0909091	0.6276
Condensation of Prometaphase Chromosomes	0.4545455	0.00703	NA	NA	0.0909091	0.6276
SLBP Dependent Processing of Replication-Dependent Histone Pre-mRNAs	0.4545455	0.00703	NA	NA	NA	NA
Gene Silencing by RNA	0.2	0.00762	0.037037	0.92238	0.0444444	0.97881
Epigenetic regulation of gene expression	0.1959459	0.00781	0.0472973	0.80903	0.0337838	0.99669

Inhibition of the proteolytic activity of APC/C required for the onset of anaphase by mitotic spindle checkpoint components	0.35	0.00792	0.15	0.12005	0.2	0.08659
Inactivation of APC/C via direct inhibition of the APC/C complex	0.35	0.00792	0.15	0.12005	0.2	0.08659
Transport of Mature mRNA derived from an Intron-Containing Transcript	0.2297297	0.00795	0.0675676	0.47696	0.0405405	0.95905
APC-Cdc20 mediated degradation of Nek2A	0.32	0.00844	0.12	0.19469	0.16	0.16224
Interconversion of nucleotide di- and triphosphates	0.3	0.00846	0.2	0.00863	0.2	0.03929
SUMOylation of RNA binding proteins	0.2608696	0.00863	0.0869565	0.31002	0.0652174	0.76798
Nonhomologous End-Joining (NHEJ)	0.2318841	0.00899	0.057971	0.61677	0.0289855	0.98494
Metabolism of vitamins and cofactors	0.1851852	0.00925	0.0793651	0.18202	0.1587302	0.00071
Apoptotic execution phase	0.25	0.00933	0.0384615	0.83622	0.0769231	0.66342
Host Interactions with Influenza Factors	0.2682927	0.00939	0.097561	0.24078	0.0487805	0.87815
Formation of Senescence-Associated Heterochromatin Foci (SAHF)	0.375	0.00952	0.0625	0.63627	0.0625	0.76239
Positive epigenetic regulation of rRNA expression	0.2075472	0.00981	0.0377358	0.896	0.0377358	0.98415
Late Phase of HIV Life Cycle	0.1956522	0.01022	0.0652174	0.47187	0.0724638	0.75723
Glyoxylate metabolism and glycine degradation	0.2903226	0.01064	0.1612903	0.03829	0.1612903	0.12205
Tetrahydrobiopterin (BH4) synthesis, recycling, salvage and regulation	0.4166667	0.01084	0.25	0.03315	0.3333333	0.01525
Ethanol oxidation	0.4166667	0.01084	0.3333333	0.00463	0.1666667	0.27558
Cyclin E associated events during G1/S transition	0.2168675	0.01183	0.1084337	0.0664	0.0481928	0.93391
RNA Polymerase I Chain Elongation	0.2111111	0.01314	0.0333333	0.9197	0.0444444	0.95655
Metabolism of nitric oxide	0.3529412	0.01319	0.1764706	0.0818	0.2941176	0.01191
eNOS activation and regulation	0.3529412	0.01319	0.1764706	0.0818	0.2941176	0.01191
RNA Polymerase I Transcription Termination	0.28125	0.01321	0.0625	0.59145	0.0625	0.77386
RNA Pol II CTD phosphorylation and interaction with CE during HIV infection	0.2962963	0.01384	0.0740741	0.49868	0.1481481	0.19751
RNA Pol II CTD phosphorylation and interaction with CE	0.2962963	0.01384	0.0740741	0.49868	0.1481481	0.19751
Citric acid cycle (TCA cycle)	0.3181818	0.01397	0.1363636	0.14852	0.0909091	0.57465
Cyclin A:Cdk2-associated events at S phase entry	0.2117647	0.01505	0.1058824	0.07485	0.0470588	0.94129
Metabolism of water-soluble vitamins and cofactors	0.195122	0.01534	0.0569106	0.63355	0.1544715	0.00847
Cleavage of Growing Transcript in the Termination Region	0.2238806	0.01546	0.0447761	0.78642	0.0149254	0.9976
RNA Polymerase II Transcription Termination	0.2238806	0.01546	0.0447761	0.78642	0.0149254	0.9976

Biological oxidations	0.1748879	0.01582	0.1210762	0.00052	0.1524664	0.00068
Apoptosis induced DNA fragmentation	0.3846154	0.01584	NA	NA	NA	NA
The activation of arylsulfatases	0.3846154	0.01584	0.3846154	0.00072	0.3846154	0.00331
eNOS activation	0.3846154	0.01584	0.2307692	0.04118	0.3076923	0.02057
Activation of DNA fragmentation factor	0.3846154	0.01584	NA	NA	NA	NA
Synthesis of PC	0.2857143	0.01732	0.1785714	0.02572	0.2142857	0.02883
Synthesis of IP3 and IP4 in the cytosol	0.2857143	0.01732	0.2142857	0.00607	0.25	0.008
Condensation of Prophase Chromosomes	0.2162162	0.01749	NA	NA	0.0135135	0.99873
RNA Polymerase III Chain Elongation	0.3333333	0.01776	0.0555556	0.6795	0.1666667	0.19693
Protein folding	0.2020202	0.01781	0.0505051	0.73264	0.0707071	0.75686
Gamma carboxylation, hypusine formation and arylsulfatase activation	0.2564103	0.0179	0.1794872	0.00861	0.2307692	0.00489
Association of TriC/CCT with target proteins during biosynthesis	0.2564103	0.0179	0.0512821	0.69888	0.0512821	0.85976
APC/C:Cdc20 mediated degradation of Cyclin B	0.3043478	0.01798	0.0869565	0.41541	0.1304348	0.31586
BBSome-mediated cargo-targeting to cilium	0.3043478	0.01798	0.0869565	0.41541	0.0869565	0.59929
Mitochondrial calcium ion transport	0.3043478	0.01798	0.0434783	0.76644	NA	NA
RHO GTPase Effectors	0.164557	0.01898	0.0348101	0.9879	0.0379747	0.99979
NoRC negatively regulates rRNA expression	0.1981132	0.01904	0.0377358	0.896	0.0377358	0.98415
Metabolism of carbohydrates	0.1672727	0.02011	0.1127273	0.00071	0.1781818	5.89E-07
Peroxisomal lipid metabolism	0.2758621	0.02139	0.1034483	0.26057	0.2068966	0.0338
Mitophagy	0.2758621	0.02139	0.0689655	0.53738	0.0689655	0.72467
mRNA Capping	0.2758621	0.02139	0.0689655	0.53738	0.137931	0.23483
Glycogen metabolism	0.2758621	0.02139	0.1724138	0.02957	0.2413793	0.00978
Defects in cobalamin (B12) metabolism	0.3571429	0.02218	NA	NA	0.1428571	0.3412
TP53 Regulates Transcription of Genes Involved in G1 Cell Cycle Arrest	0.3571429	0.02218	0.1428571	0.20994	0.1428571	0.3412
Recruitment and ATM-mediated phosphorylation of repair and signaling proteins at DNA double strand breaks	0.2105263	0.02225	0.0526316	0.69198	0.0526316	0.90109
Glycolysis	0.2142857	0.02267	0.0714286	0.42844	0.1	0.39507
Phosphorylation of the APC/C	0.3157895	0.02329	0.1052632	0.32578	0.1578947	0.21996
Protein methylation	0.3157895	0.02329	0.2105263	0.02581	0.1052632	0.49424
Transcriptional Regulation by E2F6	0.2571429	0.02369	0.1714286	0.01822	0.1428571	0.17739
DNA Double Strand Break Response	0.2077922	0.02496	0.0519481	0.70182	0.0519481	0.90652

Negative epigenetic regulation of rRNA expression	0.1926606	0.02544	0.0366972	0.90771	0.0366972	0.98697
RNA Polymerase I Promoter Escape	0.2666667	0.0261	0.0666667	0.55594	0.0666667	0.742
SLBP independent Processing of Histone Pre-mRNAs	0.4	0.02662	NA	NA	NA	NA
Cohesin Loading onto Chromatin	0.4	0.02662	0.1	0.46842	NA	NA
Folding of actin by CCT/TriC	0.4	0.02662	NA	NA	NA	NA
HDR through MMEJ (alt-NHEJ)	0.4	0.02662	0.2	0.12152	0.1	0.59259
Ketone body metabolism	0.4	0.02662	0.3	0.01981	0.4	0.00743
Pentose phosphate pathway	0.3333333	0.02995	0.2	0.05987	0.4	0.001
Neutrophil degranulation	0.1524008	0.03375	0.0542797	0.76838	0.1210856	0.0044
TP53 Regulates Transcription of Cell Cycle Genes	0.2244898	0.03454	0.122449	0.07697	0.1020408	0.41318
Cellular responses to external stimuli	0.1510934	0.03657	0.0437376	0.96671	0.0596421	0.99003
Biotin transport and metabolism	0.3636364	0.03782	0.2727273	0.02602	0.1818182	0.24253
Apoptotic cleavage of cell adhesion proteins	0.3636364	0.03782	0.0909091	0.50099	0.0909091	0.6276
Organelle biogenesis and maintenance	0.1598639	0.03881	0.0544218	0.72379	0.0680272	0.89016
Apoptotic cleavage of cellular proteins	0.2368421	0.03904	0.0526316	0.68511	0.1052632	0.41387
mRNA decay by 3' to 5' exoribonuclease	0.3125	0.03925	NA	NA	0.125	0.40484
Processing of SMDT1	0.3125	0.03925	NA	NA	NA	NA
ERCC6 (CSB) and EHMT2 (G9a) positively regulate rRNA expression	0.1973684	0.04425	0.0394737	0.8523	0.0394737	0.96419
Formation of the Early Elongation Complex	0.2424242	0.04439	0.0606061	0.6084	0.1212121	0.31342
Formation of the HIV-1 Early Elongation Complex	0.2424242	0.04439	0.0606061	0.6084	0.1212121	0.31342
Pink/Parkin Mediated Mitophagy	0.2727273	0.04643	0.0909091	0.3935	0.0454545	0.86147
Mitochondrial protein import	0.203125	0.04786	0.078125	0.35435	0.0625	0.81106
Signaling by Nuclear Receptors	0.1658291	0.04846	0.0351759	0.96422	0.0753769	0.73902
Glucose metabolism	0.1888889	0.0488	0.0777778	0.31243	0.1222222	0.14738
Cyclin D associated events in G1	0.2045455	0.08714	0.2272727	0.00026	0.1590909	0.07907
G1 Phase	0.2045455	0.08714	0.2272727	0.00026	0.1590909	0.07907
Neurotransmitter clearance	0.2	0.357	0.4	0.00217	0.1	0.59259
Nitric oxide stimulates guanylate cyclase	0.0416667	0.95838	0.25	0.00267	0.125	0.34019
Glycosphingolipid metabolism	0.1956522	0.10867	0.173913	0.00622	0.2391304	0.00142
HS-GAG degradation	0.0909091	0.77649	0.2272727	0.00932	0.1818182	0.11439
Nuclear Receptor transcription pathway	0.12	0.59818	0.16	0.01032	0.16	0.0612
Diseases of carbohydrate metabolism	0.1764706	0.23979	0.1764706	0.01589	0.2058824	0.02319
Amino acid synthesis and interconversion (transamination)	0.1764706	0.23979	0.1764706	0.01589	0.2647059	0.00178

Base-Excision Repair, AP Site Formation	0.2	0.357	0.3	0.01981	0.4	0.00743
cGMP effects	NA	NA	0.2222222	0.02138	0.0555556	0.80149
Glycogen breakdown (glycogenolysis)	0.2777778	0.06254	0.2222222	0.02138	0.3333333	0.00297
Calmodulin induced events	0.1428571	0.46369	0.1785714	0.02572	0.1071429	0.43604
CaM pathway	0.1428571	0.46369	0.1785714	0.02572	0.1071429	0.43604
PKA activation	0.2105263	0.20225	0.2105263	0.02581	0.1578947	0.21996
Creatine metabolism	0.1818182	0.40371	0.2727273	0.02602	0.3636364	0.0109
Glycerophospholipid biosynthesis	0.1705426	0.07355	0.1085271	0.02607	0.1472868	0.01384
Phase I - Functionalization of compounds	0.1308411	0.4575	0.1121495	0.03015	0.1495327	0.01989
PKA-mediated phosphorylation of CREB	0.2	0.22978	0.2	0.03074	0.15	0.2435
Inositol phosphate metabolism	0.2	0.08355	0.14	0.03143	0.16	0.0612
Miscellaneous substrates	NA	NA	0.25	0.03315	0.1666667	0.27558
Repression of WNT target genes	0.0833333	0.7958	0.25	0.03315	0.0833333	0.6596
Ca-dependent events	0.1333333	0.51969	0.1666667	0.03376	0.1	0.48205
TP53 Regulates Metabolic Genes	0.1744186	0.10669	0.1162791	0.03668	0.1162791	0.2012
Inhibition of replication initiation of damaged DNA by RB1/E2F1	0.3076923	0.06706	0.2307692	0.04118	0.2307692	0.09426
alpha-linolenic (omega3) and linoleic (omega6) acid metabolism	0.1538462	0.49176	0.2307692	0.04118	0.0769231	0.68886
alpha-linolenic acid (ALA) metabolism	0.1538462	0.49176	0.2307692	0.04118	0.0769231	0.68886
Sphingolipid metabolism	0.1797753	0.07925	0.1123596	0.04484	0.2022472	0.00047
Metabolism of lipids	0.1316147	0.27221	0.0719132	0.12086	0.1248304	0.00013
Synthesis of bile acids and bile salts	0.1764706	0.23979	0.0882353	0.34589	0.2647059	0.00178
Bile acid and bile salt metabolism	0.1395349	0.44534	0.0697674	0.49484	0.2325581	0.00289
O-linked glycosylation of mucins	0.140625	0.39699	0.109375	0.09499	0.1875	0.00753
SLC-mediated transmembrane transport	0.1097561	0.77984	0.0731707	0.24862	0.1300813	0.01146
Metabolism of folate and pterines	0.2352941	0.15062	0.1176471	0.27951	0.2941176	0.01191
Diseases associated with N-glycosylation of proteins	0.2941176	0.0501	0.0588235	0.65857	0.2941176	0.01191
Defective C1GALT1C1 causes Tn polyagglutination syndrome (TNPS)	0.1176471	0.6411	0.1176471	0.27951	0.2941176	0.01191
Defective GALNT12 causes colorectal cancer 1 (CRCS1)	0.1176471	0.6411	0.1176471	0.27951	0.2941176	0.01191
Synthesis of bile acids and bile salts via 7alpha-hydroxycholesterol	0.125	0.58591	0.0833333	0.43691	0.25	0.0138
Diseases of glycosylation	0.0942029	0.88772	0.057971	0.61472	0.1449275	0.01393

Keratan sulfate degradation	0.2307692	0.21208	0.0769231	0.56027	0.3076923	0.02057
Asparagine N-linked glycosylation	0.1390728	0.23221	0.0397351	0.96259	0.1192053	0.02697
Sulfur amino acid metabolism	0.2142857	0.12451	0.1428571	0.08876	0.2142857	0.02883
Cellular hexose transport	0.0952381	0.75361	0.0952381	0.37123	0.2380952	0.02942
Biosynthesis of the N-glycan precursor (dolichol lipid-linked oligosaccharide, LLO) and transfer to a nascent protein	0.1410256	0.37211	0.0641026	0.52403	0.1538462	0.03321
Diseases associated with glycosylation precursor biosynthesis	0.1333333	0.57114	0.0666667	0.61252	0.2666667	0.03423
Acetylcholine regulates insulin secretion	0.1	0.73386	0.1	0.46842	0.3	0.04789
Cytosolic sulfonation of small molecules	0.0833333	0.81673	0.1666667	0.05567	0.2083333	0.04971

Table S4B. Enriched Reactome pathways from down-regulated genes at 8h pi. Significant Reactome pathways using down-regulated DEGs from STM, T3SS-1^{mut} or T3SS-2^{mut}-infected HIOs at 8h.
