# Supplementary material for: Salmonella enterica Serovar Typhimurium SPI-1 and SPI-2 Shape the Global Transcriptional Landscape in a Human Intestinal Organoid Model System
Source: mBio. 2021 May 18;12(3):e00399-21. doi: 10.1128/mBio.00399-21 (PMC8262845; doi:10.1128/mBio.00399-21)
Supplement: TABLE S5 [file mbio.00399-21-st005.pdf]

Bacterial strains used in this study:

Name	Strain	Genotype
Salmonella enterica serovar Typhimurium (STM)	SL1344	
STM T3SS-1^{mut}	SL1344	*orgA*::Tn5lacZY
STM T3SS-2^{mut}	SL1344	*ssaV*::*mudJ*
STM-DsRed	SL1344	pGEN plasmid expressing Ds-Red

Table S5: List of bacterial strains used in this study, previously described in (44, 45).
